# Unveiling the Potential of *p*‑Block Bismuth in Homogeneous Electrocatalytic Proton Reduction: Secondary Coordination Sphere Control of Catalytic Activity

**DOI:** 10.1021/jacs.6c06029

**Published:** 2026-07-10

**Authors:** Yashdeep Maurya, Parul Bishnoi, Rishabh Sharma, Vineet Jhamb, Akhilesh Sharma, Swetha Vasanthdamodar Sivapreetha, Arya Singh, Puneet Gupta, Sayanti Chatterjee

**Affiliations:** † Department of Chemistry, 30112 Indian Institute of Technology Roorkee, Roorkee, Uttarakhand PIN-247667, India; ‡ Max Planck Institute for Chemical Energy Conversion, Stiftstrasse 34-36, Mülheim an der Ruhr 45470, Germany

## Abstract

The development of sustainable hydrogen evolution reaction (HER) based on earth-abundant molecular electrocatalysts requires strategies that enable controlled access to reactive low-valent intermediates while overcoming conventional activity-overpotential scaling relationships. Although transition metal complexes have been extensively explored, the potential of main-group systems remains largely untapped due to challenges in stabilizing reactive low-valent states and the lack of predictive catalyst design frameworks integrating electronics, geometry, and secondary sphere effects. Herein, we report a series of NCN pincer-supported Bi­(III) organometallic catalysts [(L^1^)­BiCl_2_] **(1)**, [(L^2^)­BiCl_2_] **(2)**, [(L^3^)­BiCl_2_] **(3)**, [(L^4^)­BiCl_2_] **(4)**, [(L^5^)­BiCl_2_] **(5)**, [(L^6^)­BiCl_2_] **(6)**, [(L^7^)­BiCl_2_] **(7)**) that promote HER through in situ electrochemical access of active Bi­(I) intermediates. Mechanistic investigations combining potential–p*K*_a_ analysis, Tafel slope measurements, in situ electrochemical studies, detailed electrokinetic study exploring foot-of-the-wave analysis (FOWA), controlled potential electrolysis, and density functional theory calculations support a Bi^I^/Bi^III^ redox cycle-mediated proton-coupled electron transfer (PCET) pathway involving transient bismuth hydride intermediates that are challenging to isolate under conventional chemical conditions. Systematic modulation of ligand electronics, geometry, and secondary coordination sphere features reveals a prominent role in modulating reactivity and further highlights that incorporation of a pendant −*NH* functionality acts as an intramolecular proton-assistance that significantly enhances catalytic activity, relative to analogues lacking pendant −*NH* proton functionality in the ligand backbone. Notably, secondary-sphere interactions from the ligand backbone enable these catalysts to circumvent traditional activity-overpotential scaling relationships, exhibiting enhanced performance at low overpotential that rivals state-of-the-art transition metal systems. These findings establish fundamental design principles for redox-active main-group electrocatalysis and expand the accessible redox space of the periodic table by leveraging *p*-block redox chemistry toward practical applications.

## Graphical abstract

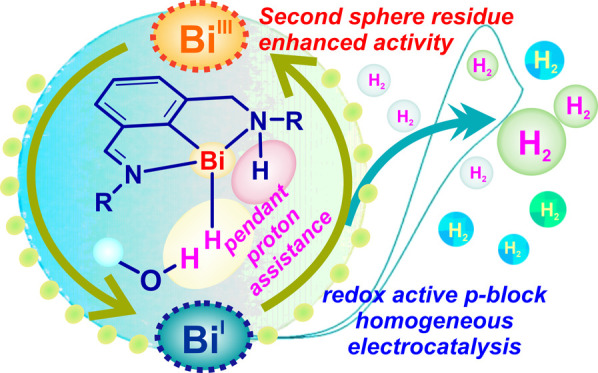

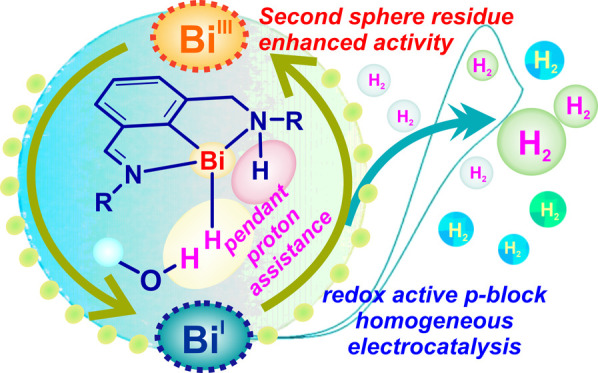

## 1 Introduction

The transition toward sustainable energy technologies requires transformative advances in catalytic small molecule activation. Among these processes, the electrochemical reduction of protons to molecular hydrogen (H_2_) occupies a central position, as hydrogen is a high-energy-density (142 MJ kg^–1^), carbon-neutral energy carrier^1–3^ whose combustion yields only water.^4^ While platinum-based materials remain the state-of-the-art electrocatalysts for the hydrogen evolution reaction (HER), their scarcity and cost present significant barriers to widespread implementation.^5–8^ The development of efficient, earth-abundant molecular alternatives capable of operating at low overpotential and high selectivity along with well-designed mechanistic control is therefore both scientifically compelling and technologically urgent. Exquisite control over the local environment surrounding the metal active sites is central to advancing catalytic performance.^7^ In enzymatic systems, such control is achieved through directed evolution of the amino acid scaffold, yielding engineered enzymes with remarkable activity and selectivity.^9^ Analogously, in molecular catalysis based on earth-abundant metals, systematic modification of ligand steric and electronic properties has enabled the development of catalysts whose performance rivals or even surpasses that of conventional synthetic precious-metal systems.^8,10–12^ Beyond primary coordination sphere effects, secondary-sphere interactions in small-molecule catalysts exert a profound influence on reactivity and selectivity, often unveiling previously inaccessible reaction pathways.^11,13–19^ Nature provides a powerful blueprint for proton reduction through hydrogenase enzymes, which mediate the reversible interconversion of protons and dihydrogen under ambient conditions with extraordinary rates and minimal energetic input.^20^ The active sites of [NiFe] and [FeFe] hydrogenases as well as mononuclear Fe hydrogenases integrate redox-active first-row transition metals within finely tuned coordination environments that orchestrate multielectron and multiproton transfer steps in a cooperative manner. For example, the [NiFe] hydrogenases are often more active in H_2_ oxidation and the [FeFe] hydrogenases are involved in the production of molecular hydrogen.^20–23^ In fact, the “Iron only” hydrogenase (Fe–Fe hydrogenase) facilitates hydrogen evolution from water with a turnover frequency (TOF) as high as 9000 s^–1^ at an ambient temperature of 30 °C.^20–23^ Inspired by these systems, extensive efforts have been devoted to the design of molecular HER catalysts based on first-row transition metals, preferably nickel, cobalt, and iron complexes.^11,19,24–50^ In particular, nickel diphosphine platforms incorporating pendant amine proton relays have demonstrated the importance of secondary coordination sphere effects in promoting proton-coupled electron transfer (PCET).^11,51–53^ These studies establish that beyond first-sphere coordination effects, secondary-sphere interactions such as suitably designed pendant proton assistance from the ligand backbone can profoundly influence catalytic pathways by lowering activation barriers, facilitating PCET, and stabilizing reactive intermediates.^54^ Moreover, there are reports exploring through-space electrostatic effects on electrocatalytic reactions, particularly HER showing inhibition and promoting through cationic and anionic units, respectively.^18^ However, these systems remain intrinsically dependent on transition-metal-centered *d*-orbital redox chemistry.

Expanding catalysis beyond the transition metal paradigm represents an emerging frontier in molecular electrocatalysis.^55–62^ Heavier *p*-block elements (*n* ≥ 3), when embedded within appropriately engineered ligand frameworks, can exhibit redox flexibility and bond activation reactivity that challenge traditional electronic classifications. However, unlocking catalytic redox cycles in main-group systems requires a fundamental rethinking of design principles. In contrast to transition metals, where closely spaced *d*-orbitals facilitate reversible access to multiple oxidation states, *p*-block elements possess frontier orbitals of predominantly *s* and *p* character separated by significantly larger energy gaps. Consequently, redox processes at main-group centers are frequently thermodynamically driven and proceed in a unidirectional, stoichiometric fashion, with the products acting as thermodynamic sinks. Achieving reversible redox cycling and sustained catalytic turnover in such systems therefore remains a central challenge in advancing main-group redox catalysis.^55,63–65^

Within this context, bismuth has recently emerged as a particularly intriguing platform.^55,65^ As the heaviest stable element in the periodic table, bismuth uniquely combines accessible multivalent oxidation states with increasing evidence of transition-metal-like reactivity under appropriate ligand constraints. Recent advances have demonstrated that rationally designed bismuth complexes can engage in redox-mediated bond activation processes previously considered exclusive to *d*-block chemistry.^55,65^ In particular, the Bi­(I)/Bi­(III) redox manifold has been effectively exploited by Cornella and coworkers for small-molecule activation in organic synthesis. This platform enables diverse chemical transformations such as transfer hydrogenation of azoarenes and nitroarenes using ammonia borane,^66,67^ hydrodefluorination reactions,^68^ and catalytic activation of N_2_O, demonstrating that low-valent bismuth complexes can engage in catalytic redox cycles analogous to those traditionally dominated by transition metals.^69^ However, despite the expanding accessibility of Bi­(I)/Bi­(III) redox manifolds, their widespread practical catalytic applications remain scarce. This limited development arises largely from the synthetic challenges associated with generating and stabilizing low-valent bismuth species, which typically require air- and moisture-sensitive techniques and the use of harsh or stoichiometric chemical reductants and thereby restrict their alignment with the principles of atom-economic and sustainable catalysis.

In this regard, the development of alternative strategies that enable controlled access to reactive low-valent bismuth redox states under mild and sustainable conditions represents a critical unmet challenge. Addressing this gap would not only expand the synthetic utility of bismuth redox catalysis but also establish a viable paradigm for main-group elements to emulate and potentially surpass traditional transition metal redox catalysis if possible. Electrochemical strategies provide a compelling alternative by enabling controlled, in situ access to reactive oxidation states under mild conditions, thereby eliminating the need to isolate reactive (low-valent) intermediates/precursors. Thus, integrating catalyst activation directly into the catalytic cycle, electrochemical approaches would allow transient Bi­(I) species to be accessed under operationally simple conditions while minimizing thermodynamic trapping and decomposition pathways and offering a practical and sustainable route to catalytically competent low-valent main-group species.^57–62^

Despite this promise, electrochemical exploration of homogeneous bismuth redox catalysis remains in its infancy. To date, bismuth has mainly been explored as a dopant in heterogeneous electrocatalysis rather than as an active catalytic center.^70^ Beyond an isolated preliminary report,^62^ unveiling the potential of bismuth as a homogeneous active electrocatalyst has remained largely underdeveloped. Systematic structure–activity relationship investigations of redox-flexible bismuth complexes are scarce. In particular, the influence of ligand backbone electronics, geometric constraints, and secondary coordination sphere engineering effects toward small-molecule bond activation processes has not been rigorously examined in the main-group regime. Moreover, the deliberate incorporation of a secondary-sphere functionality designed to serve as an intramolecular proton assistance, an approach that has proven transformative to modulate the electrochemical reactivity and selectivity in metalloenzymes and synthetic transition metal catalysis,^11,18,19,51–53^ has not yet been demonstrated in a main-group Bi-based molecular electrocatalyst platform. We envision that in situ electrochemical access to rationally designed low-valent Bi­(I) species from Bi­(III) precursors and the formation of reactive bismuth intermediates under electrochemical conditions would open new possibilities for small-molecule activation, including efficient proton reduction, and expand the reactivity landscape of main-group elements.

Herein, we explore the potential of a series of ligand-constrained redox-active bismuth NCN pincer complexes as homogeneous electrocatalysts for HER. By systematically modulating ligand architecture and redox accessibility, we examine how electronic structure, geometric constraint, and secondary sphere design influence catalytic efficiency and mechanistic pathways. These studies establish foundational design principles for enabling reversible redox cycling at a *p*-block center and advance molecular bismuth as a viable platform for main-group energy-relevant electrocatalysis.

## 2 Experimental Results

### 2.1 Synthesis and Characterization of Ligands and Complexes

The pincer-based bromo-substituted ligands L^1^Br, L^2^Br, L^5^Br, L^6^Br, and L^7^Br reported in this work were synthesized starting from 2-bromoisophthaldehyde in a multistep process for L^1^Br, L^6^Br, and L^7^Br and in a one-step procedure for L^2^Br, L^5^Br (see Experimental Results section for details) in high yield and purity and characterized by various spectroscopic techniques (Schemes S1–S5, Scheme S10, Scheme S12, and Scheme S14, SI). Ligands L^3^Br and L^4^Br were synthesized with slight modifications of the literature procedure.^66,67^

The corresponding organometallic Bi­(III) complexes ([(L^1^)­BiCl_2_] **(1)**, [(L^2^)­BiCl_2_] **(2)**, [(L^3^)­BiCl_2_] **(3)**, [(L^4^)­BiCl_2_] **(4)**, [(L^5^)­BiCl_2_] **(5)**, [(L^6^)­BiCl_2_] **(6)**, [(L^7^)­BiCl_2_] **(7)**), supported by carbanionic ligands L^1^, L^2^, L^3^, L^4^, L^5^, L^6^, L^7^ respectively were synthesized using modification of literature report^66,67^ (Schemes S6–S9, Scheme S11, Scheme S13 and Scheme S15 SI, for detailed synthesis and characterization). An analogous redox-inactive Al­(III) complex ([(L^2^)­AlCl_2_] **(8)**) was also synthesized and characterized for a control study (see SI for detailed characterization, Scheme S16, Figures S43–S47).

#### 2.1.1 Synthesis of Dichlorobismuthine [L^1^BiCl_2_] **(1)** and [L^2^BiCl_2_] **(2)**

An oven-dried 100 mL two-necked round-bottomed flask equipped with a magnetic stir bar was charged with ligand (L^1^Br) (1.12 g, 3.8 mmol, 1 equiv) or L^2^Br (1 g, 3.4 mmol, 1 equiv) for the corresponding complexes **[(L^1^)­BiCl_2_]** **(1)** or **[(L** ^**2**^ **)­BiCl**_**2**_ **] (2)**, respectively, in 50 mL of dry tetrahydrofuran (THF) under N_2_ and was cooled to −78 °C. Then, *n*-BuLi (4.18 mL, 8.32 mmol, 2.2 equiv, 2 M in hexane) was added dropwise to the precooled mixture. After complete addition, the solution turned a dark red color. The temperature was maintained at −78 °C for an additional period of 2 h under stirring conditions. After 2 h, a solution of BiCl_3_ (1.1 g, 3.8 mmol, 1.0 equiv) dissolved in dry tetrahydrofuran (THF) (around 10 mL) under N_2_ was added to the precooled reaction mixture and stirred for 10–15 min before being brought to room temperature. The resulting brown mixture thus formed was stirred and allowed to reach at ambient temperature overnight. The subsequent workup was carried out under air. All volatiles were removed under vacuum. The resulting solids were redissolved in dichloromethane (CH_2_Cl_2_) and filtered through Celite prewetted with dichloromethane. The filtrate was concentrated under vacuum to 5 mL, and hexane was added to precipitate the product, which was filtered using a frit filter and further washed with 100 mL of hexane to yield the corresponding dichlorobismuthine [(L^1^)­BiCl_2_] **(1)** (51%, off-white) and [(L^2^)­BiCl_2_] **(2)** (65%, pale yellow), respectively, as solid powders. The catalysts [(L^1^)­BiCl_2_] **(1)** and [(L^2^)­BiCl_2_] **(2)** were characterized by ^1^H NMR, HRMS, IR and UV–vis spectroscopy. Single crystals suitable for X-ray diffraction studies for [(L^1^)­BiCl_2_] **(1)** and [(L^2^)­BiCl_2_] **(2)** were grown from a 1:1 dichloromethane:ethyl acetate solvent mixture via the layering method at room temperature and data collected at 100 K (Figures S11–S24, Figure S48, Figures S50–S52, Tables S1–S6, see SI for complete spectroscopic and analytical characterization including crystallographic tables). Catalysts [(L^3^)­BiCl_2_] **(3)** and [(L^4^)­BiCl_2_] **(4)** as well as other Bi­(III) catalysts [(L^5^)­BiCl_2_] **(5)** to [(L^7^)­BiCl_2_] **(7)** were synthesized following a modification of a literature procedure^66,67^ and characterized by various spectroscopic and analytical techniques (Figures S25–S30, Figure S33, Figure S34, Figure S37, Figure S38, Figure S41, Figure S42, and Figures S48–S49, SI).

### 2.2 Electrochemistry

All electroanalytical experiments were performed using a Metrohm DropSens μStat-i 400 potentiostat, and appropriate iR_drop_ compensation was applied during data acquisition/analysis. Glassy carbon was used as the working electrode (3 mm), Ag/AgCl as the pseudoreference electrode, and Pt wire was used as the counter electrode unless otherwise stated. As a control, the Pt wire counter electrode was replaced by an inert glassy carbon electrode, keeping glassy carbon as the working electrode (3 mm), and Ag/AgCl as the pseudoreference electrode, to rule out any Pt nanoparticle contribution from the counter electrode in the electrochemical reactivity. All CV experiments were performed in a modified scintillation vial (20 mL volume) as a single-chamber cell with a cap modified with ports for all electrodes and a sparging needle. Controlled potential electrolysis experiments were performed in a four-port 50 mL European-style flask with a glassy carbon rod working electrode (Metrohm, type 1, 3 mm diameter), platinum wire counter electrode (Metrohm, spectrographic grade, 0.5 mm diameter), a custom silver/silver chloride pseudoreference electrode (Metrohm, 99.9%, 1.0 mm diameter), and ports for headspace sampling and gas sparging via needles through septa. Tetrabutylammonium hexafluorophosphate (TBAPF_6_) was purified and dried in a vacuum oven before being stored in a desiccator. All data were referenced to an internal ferrocene standard (ferrocene/ferrocenium (Fc/Fc^+^) reduction potential under stated conditions) unless otherwise specified. Voltammograms were plotted according to polarographic or US convention, and the initial direction of the scan was cathodic. All experiments were conducted at 25 °C. For an individual run, the jar was charged with a known amount of catalyst, a known amount of proton source (Scheme S24, SI), a stir bar, and an electrolyte solution (0.1 M TBAPF_6_/Acetonitrile) before being sparged to saturation with N_2_ (depending on the experiment). After a run was completed, the headspace was sampled via an airtight syringe and characterized by GC-TCD (HP 7890A with TCD and Agilent 8890 with TCD) such that Faradaic efficiency could be determined by using calibration curves.

### 2.3 Spectroelectrochemical Studies

UV–vis spectroelectrochemical experiments were performed using a SEC-CT thin-layer quartz spectroelectrochemical cell equipped with a platinum grid mesh working electrode (3 cm × 0.95 mm square; height 6 cm; rod thickness 1 mm), a platinum wire counter electrode (1 mm diameter), and an Ag/AgCl (Vycor frit) pseudoreference electrode. All measurements were conducted under an inert N_2_ atmosphere in acetonitrile containing 0.1 M TBAPF_6_ as the supporting electrolyte. The complexes were studied at a concentration of 0.5 mM. Electrochemical control was achieved using a Metrohm DropSens μStat-i 400 potentiostat operated in amperometric mode and coupled to an Agilent Cary 60 UV–vis spectrophotometer. Prior to spectroelectrochemical measurements, cyclic voltammograms were recorded to verify the electrical integrity of the setup. A potential of −1.25 V vs Ag/AgCl was then applied to access the Bi^III^/Bi^I^ redox couple for complexes [(L^1^)­BiCl_2_] **(1)** and [(L^2^)­BiCl_2_] **(2)** (Figures S201–S208, SI). The corresponding Al­(III) complex [(L^2^)­AlCl_2_] **(8)** were also subjected to a similar spectroelectrochemical study as a control to monitor the formation of any ligand-based radical anion during the spectroelectrochemical redox study (Figure S47, SI).

### 2.4 Computational Methods

Density functional theory (DFT) calculations were conducted by using the ORCA 5.0.3 ^71,72^ quantum chemical software package. All the calculations were carried out using the PBE0 density functional^73^ incorporating Grimme’s D3 dispersion correction with Becke–Johnson damping.^74,75^ Solvent effects were modeled using the SMD solvation mode^76^ with acetonitrile as the implicit solvent. To shed light on electron transfer events, intrinsic bonding orbital (IBO) analysis was undertaken using IBOView software^77^ (Section XVII, Figures S231–S232, SI).

## 3 Results and Discussion

### 3.1 Synthesis and Electronic Structure of [(L^1^)­BiCl_2_] **(1)** and [(L^2^)­BiCl_2_] **(2)**

The bromo ligand precursors L^1^Br–L^7^Br were synthesized in good yields and fully characterized by ^1^H and ^13^C NMR spectroscopy and HRMS (Schemes S1–S5, Scheme S10, Scheme S12 and Scheme S14, Figures S1–S10, Figures S31–S32, Figures S35–S36 and Figures S39–S40, see SI for details). All ligand precursors (L^1^Br–L^7^Br) were subsequently subjected to metalation reactions. In a typical procedure, lithiation of the ligand precursors with *n*-BuLi at −78 °C in THF was followed by in situ salt metathesis with one equivalent of BiCl_3_ at low temperature, to afford the corresponding dichlorobismuthine complexes [(L)­BiCl_2_] **(1–7)**. Complexes [(L^1^)­BiCl_2_] **(1)** and [(L^2^)­BiCl_2_] **(2)** were isolated as off-white and pale-yellow solids, respectively (Schemes S6–S7, see Supporting Information for details). Other bismuth catalysts [(L^3^)­BiCl_2_] **(3)** to [(L^7^)­BiCl_2_] **(7)** were also synthesized and characterized for comparative catalytic studies. The molecular structures of complexes [(L^1^)­BiCl_2_] **(1)** and [(L^2^)­BiCl_2_] **(2)** were unambiguously established by single-crystal X-ray diffraction (scXRD).

Single crystals of the complexes [(L^1^)­BiCl_2_] **(1)** and [(L^2^)­BiCl_2_] **(2)** were obtained by layering ethyl acetate into a dichloromethane solution of the respective complexes [(L^1^)­BiCl_2_] **(1)** and [(L^2^)­BiCl_2_] **(2)** at room temperature. Colorless for [(L^1^)­BiCl_2_] **(1)** to pale yellow for [(L^2^)­BiCl_2_] **(2)** block-shaped crystals thus obtained were analyzed at low temperature by single-crystal X-ray diffraction studies (scXRD). The solid-state structures of both [(L^1^)­BiCl_2_] **(1)** and [(L^2^)­BiCl_2_] **(2)** reveal neutral, five-coordinate Bi­(III) centers supported by carbanionic N–C–N pincer ligands bound in a pseudomeridional fashion (Figure 1a and 1b) with N1–Bi–N2 bond angles of 141.2° (6) for complex [(L^1^)­BiCl_2_] **(1)** and 142.7° (9) for complex [L^2^BiCl_2_] **(2)**. Coordination of two chloride ligands completes the coordination sphere, resulting in a distorted square-pyramidal geometry for complex [(L^1^)­BiCl_2_] **(1)** (*τ* = 0.45), whereas complex [(L^2^)­BiCl_2_] (**2)** adopts a geometry intermediate between square-pyramidal and trigonal-bipyramidal (*τ* = 0.50). Interestingly, the solid-state molecular structures of both the compounds [(L^1^)­BiCl_2_] **(1)** and [(L^2^)­BiCl_2_] **(2)** exhibit a T-shaped geometry of the CBiCl_2_ core, with trans halogen atoms having Cl1–Bi–Cl2 bond angles of around 168.19° (17) for complex [(L^1^)­BiCl_2_] **(1)** and 172.70° (3) for complex [(L^2^)­BiCl_2_] **(2)**. In complex [(L^2^)­Bi^III^Cl_2_] **(2)**, which features two symmetrically substituted imine arms bearing *N*-isopropyl substituents, the Bi–N bond lengths are nearly identical (≈2.49 Å (3), 2.47 Å (3)) and are comparable to those reported for the related N-*t*Bu substituted analogue ([(L^4^)­BiCl_2_] **(4)**).^78^ The Bi–N bond distances of 2.47 Å (3) to 2.49 Å (3) in complex [(L^2^)­BiCl_2_] **(2)**, when compared with the Σ_cov_(Bi,N) = 2.22 Å ^79–81^ and van der Waals radii (Σ_vdW_(Bi,N) = 3.94 Å), indicate the presence of intramolecular N → Bi interactions.^82,83^ As a result of the intramolecular coordination of the nitrogen to the bismuth atom, two five-membered BiC3N rings are formed. Interestingly, the *trans* Bi–Cl bond lengths in compound [(L^2^)­BiCl_2_] **(2)** (Bi–Cl1 = 2.69 Å (11) and Bi–Cl2 = 2.66 Å (11)) are considerably longer than the characteristic Bi–Cl covalent bond (Σ_cov_(Bi,Cl) = 2.51) in agreement with the 3c–4e bonding description commonly invoked for hypervalent bismuth compounds.^79–81^ For the symmetrically coordinated imine complex [(L^2^)­BiCl_2_] **(2)**, along with the comparable Bi–N bond distances, the symmetric imine character of the ligand backbone is further corroborated by almost equivalent N–C bond lengths of both the imine arms (N(1)–C(7) = 1.27 Å (4) and N(2)–C(8) = 1.28 Å (4)). The *N*-isopropyl substituents provide an optimal balance of steric and electronic effects around the T-shaped Bi center, intimating potential relevance for catalytic applications. In contrast, complex [(L^1^)­BiCl_2_] **(1)** features an asymmetric N–C–N pincer ligand, comprising one imine arm (C7–N1 = 1.22 Å (3)) and one reduced amine arm (C8–N2 = 1.48 Å (3)). This asymmetry is reflected in the Bi–N bond distances: the imine nitrogen exhibits a longer Bi–N1 distance (2.56 Å (2)) relative to the more strongly donating amine nitrogen (Bi–N2 = 2.46 Å (18)). These Bi–N distances are significantly longer than the sum of the covalent radii (Σ_cov_(Bi,N) = 2.22 Å) yet substantially shorter than the sum of the van der Waals radii (Σ_vdW_(Bi,N) = 3.94 Å), consistent with intramolecular N → Bi donor interactions.^82,83^ Despite this asymmetry, the Bi–C bond distances in both complexes [(L^1^)­BiCl_2_] **(1)** and [(L^2^)­BiCl_2_] **(2)** remain essentially identical (≈2.20 Å), in good agreement with reported values for carbanionic bismuth pincer complexes.^82^ Analogous to complex [(L^2^)­BiCl_2_] **(2)**, the Bi–Cl bonds in complex [(L^1^)­BiCl_2_] **(1)** are also elongated (Bi–Cl1 = 2.67 Å (5), Bi–Cl2 = 2.78 Å (5)), consistent with hypervalent bonding interactions.^79–81^ Additional evidence for ligand asymmetry in complex [(L^1^)­Bi^III^Cl_2_] **(1)** is provided by the C–C bond lengths adjacent to the carbanionic carbon: the C2–C8 bond associated with the amine arm in [L^1^BiCl_2_] **(1)** is elongated (1.46 Å (3)) relative to the C6–C7 bond of the imine arm (1.42 Å (4)), indicative of *sp* ^3^ and *sp* ^2^ hybridization, respectively, for C8 and C7 carbon atoms. The two hydrogen atoms attached to the *sp* ^3^-hybridized C8 atom are noncoplanar, as observed crystallographically. This asymmetry also extends to the *N*-alkyl substituents, with the isopropyl groups residing in distinct planes due to the differing geometries of the amine and imine donors (Figure 1c). Overall, complex [(L^1^)­BiCl_2_] **(1)** is distinguished by its mixed amine–imine pincer coordination and asymmetric ligand framework, whereas complex [(L^2^)­BiCl_2_] **(2)** features a fully symmetric bis-imine N–C–N pincer scaffold. These structural differences impart distinct coordination environments around the Bi­(III) center and are expected to have important implications for their reactivity and catalytic behavior (Figure 1). The geometric preferences of *p*-block element compounds are traditionally rationalized by valence shell electron pair repulsion (VSEPR) theory, which in most of the cases could explain the structures of a wide range of main-group molecules.^84^ However, increasing evidence suggests that these preferences can be deliberately overridden through ligand-imposed constraints, forcing *p*-block centers into non-VSEPR geometries.^85^ Such enforced distortions can lead to profound changes in the energies, compositions, and spatial distributions of frontier molecular orbitals, thereby altering fundamental bonding characteristics (vide infra). Exploiting these electronically perturbed states has recently emerged as a powerful strategy for unlocking unconventional reactivities and properties in *p*-block chemistry, offering new opportunities for main-group compounds in areas traditionally dominated by transition metals, which is a main focus of the work presented here.^55,61,65,78,86,87^

**Figure 1:**
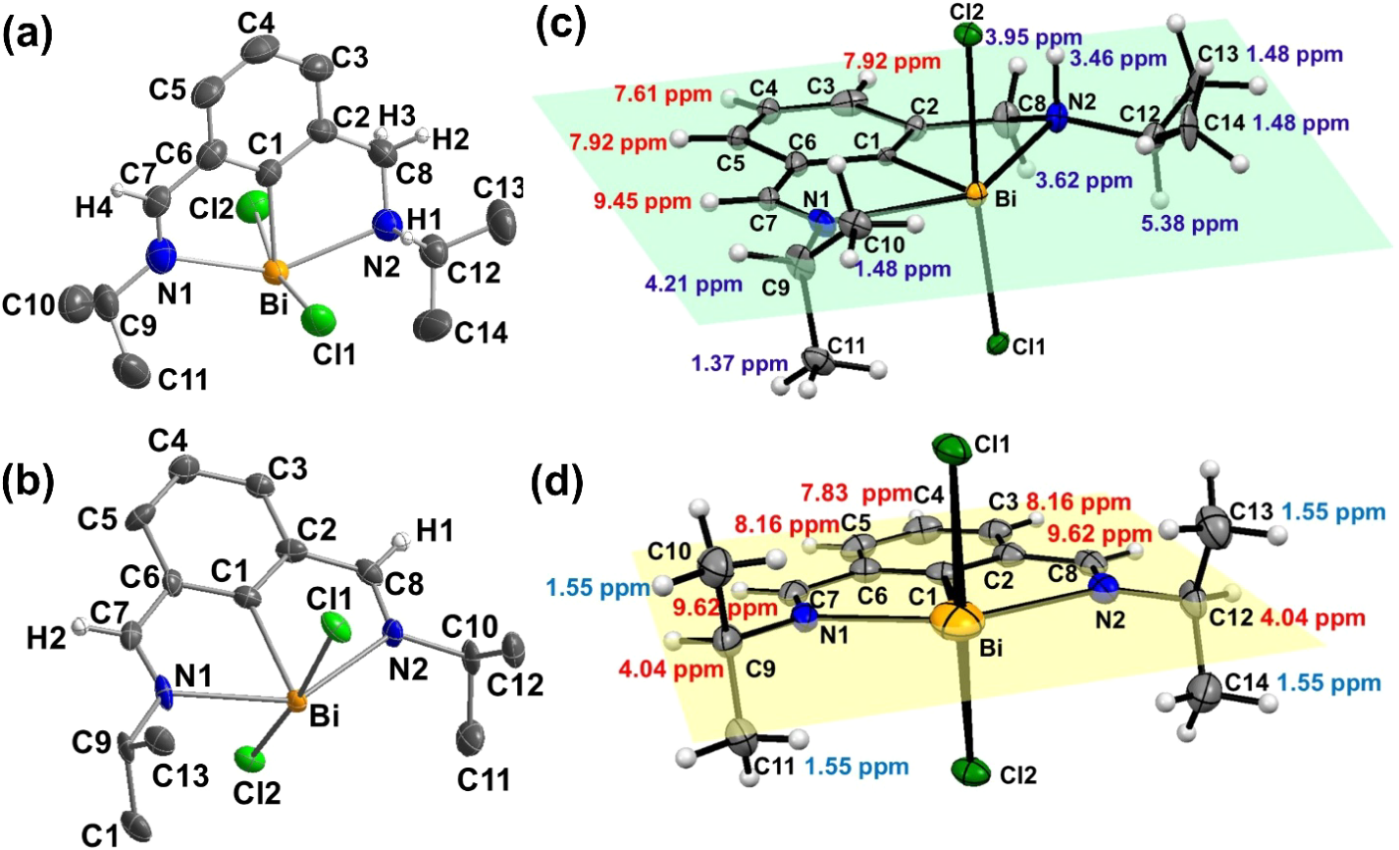
ORTEP plots of (a) [(L^1^)­BiCl_2_] **(1)** and (b) [(L^2^)­BiCl_2_] **(2)** with 50% ellipsoid probability. All hydrogen atoms except those on C7, C8, and N2 for complex [L^1^BiCl_2_] **(1)** and C7 and C8 for complex [L^2^BiCl_2_] **(2)** have been removed for clarity. Three-dimensional views of proton environments in (c) [(L^1^)­BiCl_2_] **(1)** and (d) [(L^2^)­BiCl_2_] **(2)**, along with the assignment of corresponding ^1^H NMR chemical shifts. Protons positioned out of the molecular plane are highlighted in blue, while in-plane protons are shown in red.

The ^1^H and ^13^C­{^1^H} NMR spectra measured in deuterated chloroform (CDCl_3_) confirm that complexes [(L^1^)­BiCl_2_] **(1)** and [(L^2^)­BiCl_2_] **(2)**, as well as other Bi­(III) catalysts [(L^3^)­BiCl_2_] **(3)** to [(L^4^)­BiCl_2_] **(7)**, are diamagnetic on the NMR time scale and demonstrate their high purity and solution stability (Figures S26–S30, Figures S33–S34 and Figures S37–S40, SI). The ^1^H NMR spectrum of complex [(L^2^)­BiCl_2_] **(2)** (Figure S18, SI) displays two imine C–H resonances at *δ* 9.62 ppm (2H), which are significantly downfield relative to the free ligand L^2^ (*δ* 8.74 ppm), indicating coordination of both imine donors to the Bi center. The methine protons (C–H) of the isopropyl groups attached to each imine nitrogen appear as a multiplet at *δ* 4.04 ppm, while the isopropyl methyl protons (−CH_3_) resonate as a doublet at *δ* 1.55 ppm (12H), consistent with a symmetric ligand environment (Figure 1d). In contrast, the ^1^H NMR spectrum of complex [(L^1^)­BiCl_2_] **(1)** (Figure S11, SI) exhibits a single imine C–H resonance at *δ* 9.46 ppm, downfield from that of the free ligand L^1^ (*δ* 8.72 ppm), indicating coordination of only one imine to the Bi center. The methylene protons appear as two resonances at *δ* 3.60 and 3.95 ppm, each split into doublets due to coupling with the N–H proton (*δ* 3.46 ppm, ^3^ *J* = 12.6 Hz) (Figure 1c). The methine proton (C–H) of the isopropyl group bound to the amine nitrogen resonates further downfield at *δ* 5.37 ppm compared to that of the methine proton (C–H) of the isopropyl group attached to the imine nitrogen (*δ* 4.24 ppm). The isopropyl methyl protons give rise to two distinct doublets at *δ* 1.50 and 1.37 ppm due to diverse planar deployment (Figure 1c). This pronounced asymmetry in complex [L^1^BiCl_2_] **(1)**, in contrast to the symmetric complex [(L^2^)­BiCl_2_] **(2)**, is clearly evidenced by the presence of distinct sets of isopropyl resonances as well as two chemically nonequivalent protons associated with the *sp* ^3^-hybridized carbon of the amine arm (Figure 1c and 1d). In general, the aromatic protons of the L^2^ ligand backbone in complex [(L^2^)­BiCl_2_] **(2)** are downfield (*δ* 8.17 ppm and *δ* 7.83 ppm) as compared to the aromatic protons of complex [(L^1^)­BiCl_2_] **(1)** (*δ* 7.91 and 7.67 ppm), suggesting the electron-deficient nature of the bis-imine complex [(L^2^)­BiCl_2_] **(2)** as compared to the imine-amine Bi pincer complex [(L^1^)­BiCl_2_] **(1)**. To gain further insight into the solution-phase composition and connectivity of the Bi­(III) complexes, and to correlate the solid-state structures with their behavior in solution, detailed two-dimensional NMR experiments (COSY and NOESY) were performed (Figures S13–S14 and Figures S20–S21, SI). The analyses confirm that the solution-phase compositions of the complexes are consistent with their solid-state structures. The NOESY experiment also confirms the presence of the N–H proton in the amine arm of complex [(L^1^)­BiCl_2_] **(1)**. The distinct differentiation of proton environments in complex [(L^1^)­BiCl_2_] **(1)** clearly reflects its asymmetric structural arrangement, in contrast to the comparatively symmetric environment observed for complex [(L^2^)­BiCl_2_] (**2)**. The ^1^H NMR spectra of complexes [(L^3^)­BiCl_2_] **(3)** and [(L^4^)­BiCl_2_] **(4)** match with the previous literature reports (Figures S26–S27, Figures S29–S30, SI). High-resolution mass spectrometry (HRMS) analysis of all Bi­(III) complexes ([(L^1^)­BiCl_2_] **(1)** to [(L^4^)­BiCl_2_] **(4)**) further confirms their compositions (Schemes S6–S9, Figure S15 and Figure S22, SI). For catalyst [(L^1^)­BiCl_2_] **(1)**, the observed peak at *m*/*z* 461.1255 with the isotopic distribution corresponds to the cationic fragment **[1-Cl]** ^**+**^. Similarly, for [(L^2^)­BiCl_2_] **(2)**, the peak at *m*/*z* 459.1039 with the isotopic distribution is consistent with the formation of **[2-Cl]** ^**+**^, supporting the proposed molecular formulations (see Figure S15 and Figure S22, SI).

### 3.2 Electrochemical Characterization of Catalysts

Electrochemical studies of catalysts [(L^1^)­BiCl_2_] **(1)**, [(L^2^)­BiCl_2_] **(2)**, [(L^3^)­BiCl_2_] **(3)**, and [(L^4^)­BiCl_2_] **(4)** were conducted by cyclic voltammetry (CV) referenced to the Fc/Fc^+^ couple (Tables S8–S9 and Figures S71–S77, SI). All complexes display a quasi-reversible redox event centered at approximately −1.0 V vs Fc/Fc^+^. In addition, full-range CV measurements reveal a second redox feature at more negative potentials (around –2.5 V vs Fc/Fc^+^), which could be assigned to ligand-centered electron transfer processes arising from the redox noninnocent NCN pincer scaffold. For catalyst [(L^2^)­BiCl_2_] **(2)** bearing the symmetrical NCN bis-aldimino pincer ligand (L^2^), an anodic peak at −0.86 V vs Fc/Fc^+^ is attributed to oxidation at the Bi center, Bi­(III), while a quasi-reversible cathodic wave at −1.33 V vs Fc/Fc^+^ corresponds to reduction to Bi­(I). The resulting *E*_red_ value of −1.09 V vs Fc/Fc^+^ is thus assigned to the Bi­(I)/Bi­(III) redox couple for catalyst [L^2^BiCl_2_] **(2)** (Figure 2 red graph). Comparison of the integrated peak currents with the Fc/Fc^+^ standard confirms a two-electron process, consistent with a Bi­(I)/Bi­(III) two-electron redox manifold operating through an electrochemical pathway. The large peak-to-peak separation (Δ*E*_p_) of 0.47 V for catalyst [(L^2^)­BiCl_2_] (**2**) with a pseudoreversible cyclic voltammogram peak is likely indicative of some structural reorganization between the oxidized and reduced species. One such possibility could be the chloride dissociation in the putative bismuthidine intermediate [(L^2^)­Bi^I^] **(2′)**, formed electrochemically. For catalyst [(L^1^)­BiCl_2_] **(1)**, with one of the pendant imine arms reduced with an amine, a more electron-rich Bi catalyst is formed as evident with a more negative *E*_red_ value of −1.14 V vs Fc/Fc^+^ (Figure 2 blue graph). For catalyst [(L^1^)­BiCl_2_] **(1)**, an anodic peak at −1.09 V vs Fc/Fc^+^ is attributed to oxidation at the Bi center, Bi­(III), while a quasi-reversible cathodic wave at −1.19 V vs Fc/Fc^+^ corresponds to reduction to Bi­(I). The lower potential for the cathodic peak current at −1.19 V for catalyst [(L^1^)­BiCl_2_] **(1)** (vs −1.33 V for catalyst [(L^2^)­BiCl_2_] **(2)**) as well as the enhanced current density (*J*_pc_ = 2.77 × 10^–4^ A/cm^2^ for catalyst [L^1^BiCl_2_] **(1)**, whereas *J*_pc_ = 1.42 × 10^–4^ A/cm^2^ for catalyst [(L^2^)­BiCl_2_] **(2)**) implies a more electron-rich Bi catalyst in [(L^1)^BiCl_2_] **(1)**, and easy access of the corresponding bismuthidine [(L^1^)­Bi^I^] **(1′)** as compared to [(L^2^)­Bi^I^] **(2′)** (Figure 2).

**Figure 2:**
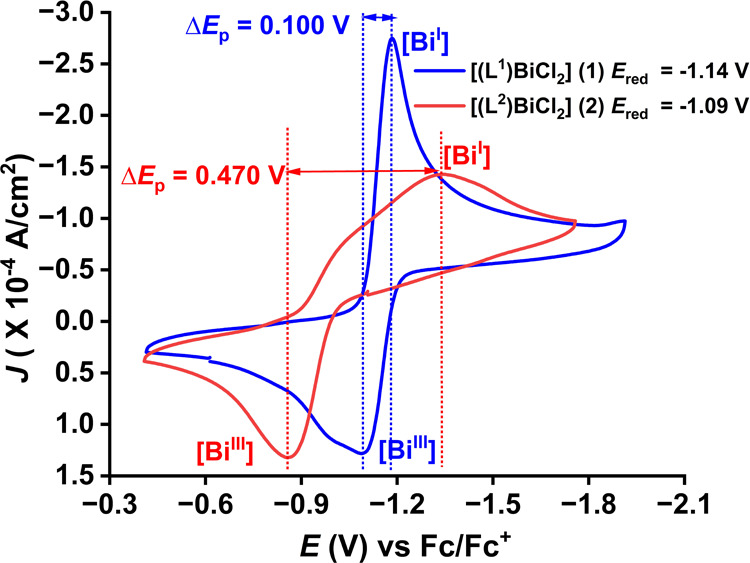
Cyclic voltammograms of 0.5 mM [(L^1^)­BiCl_2_] **(1)** (blue) and [(L^2^)­BiCl_2_] **(2)** (red) in CH_3_CN with 0.1 M TBAPF_6_ supporting electrolyte under N_2_; scan rate is 100 mV/s. The working electrode is glassy carbon (3 mm diameter), the counter electrode is platinum, and the pseudoreference electrode is Ag/AgCl wire with ferrocene (Fc) added as an internal reference.

The diminished quasireversibility of catalyst [(L^1^)­BiCl_2_] **(1)** relative to catalyst [(L^2^)­BiCl_2_] **(2)** could be attributed to subtle structural reorganization, as discussed in the previous section (Figure 1c and 1d). One such reorganization could relate to chloride dissociation from catalyst [(L^1^)­BiCl_2_] **(1)**, to form bismuthidine [L^1^Bi^I^] **(1′)** which likely retains sufficient conformational flexibility owing to the orientation of the isopropyl substituents within its asymmetric scaffold as depicted in Figure 1c, thereby minimizing steric reorganization at the metal center. In contrast, chloride loss in [L^2^Bi^I^] **(2′)** could induce more significant structural reorganization, leading to the increased irreversibility observed in the cyclic voltammogram of [L^2^BiCl_2_] **(2)**. The *E*_red_ vs Fc/Fc^+^ for the corresponding catalysts **[(L** ^**3**^ **)­BiCl**_**2**_ **] (3)** and **[(L** ^**4**^ **)­BiCl**_**2**_ **] (4)**, under the standard electrochemical conditions of the present study, were calculated to be −1.27 V and −1.12 V, respectively, vs Fc/Fc^+^ (Table S8, Figures S73–S74, SI). Analyses of the current density vs square root of scan rate plot for all the complexes [(L^1^)­BiCl_2_] **(1)**, [(L^2^)­BiCl_2_] **(2)**, [(L^3^)­BiCl_2_] **(3)**, and [(L^4^)­BiCl_2_] **(4)** gave a straight line obeying the Randles–Ševčík equation (eq 1)^88^ indicating that the electrocatalytic redox phenomenon is under a diffusion-controlled process (see Figures S71–S77, SI).

1 $$i_{p}=0.446nFAC^{0}{\left(nF\nu D_{0}/RT\right)}^{1/2}$$

where *i*_p_ is the peak current (A), ν is the scan rate (V/s), *n* is the number of electrons transferred in the redox event, *A* is the electrode surface area (cm^2^), *D*_0_ is the diffusion coefficient of the oxidized analyte, and *C* ^0^ is the bulk analyte concentration. From eq 1, the diffusion coefficients of all the catalysts [(L^1^)­BiCl_2_] **(1)**, [(L^2^)­BiCl_2_] **(2)**, [(L^3^)­BiCl_2_] **(3)**, and [(L^4^)­BiCl_2_] **(4)** were calculated (Figure S78, Table S10, SI), indicating the mobility of the catalysts and the mass transport-limited current.^88,89^

The Bi-based electrocatalysts with designed electronics and sterics ([(L^1^)­BiCl_2_] **(1)**, [(L^2^)­BiCl_2_] **(2)**, [(L^3^)­BiCl_2_] **(3)**, and [(L^4^)­BiCl_2_] **(4)**) were subsequently evaluated for the HER. In molecular HER catalysis, the generation of H_2_ formally requires the coupling of two electrons and two protons at the active site, and the elementary steps are commonly described in terms of PCET processes. PCET is classically depicted using a square scheme, wherein stepwise electron transfer (ET) and proton transfer (PT) pathways compete with a concerted proton–electron transfer (CPET) route.^4,90–94^ The stepwise pathways often proceed through high-energy charged intermediates. While the mechanistic landscape of PCET has been extensively delineated for transition metal complexes,^27,95–97^ analogous investigations in redox-active main-group systems remain largely underdeveloped. In particular, systematic studies addressing PCET manifolds in *p*-block-based HER catalysts are scarce. Establishing a detailed mechanistic framework for PCET in Bi-centered systems is therefore essential for advancing rational catalyst design. Such insights will not only enable the optimization of main-group redox platforms for HER but also broaden the fundamental understanding of multielectron/multiproton reactivity beyond the traditional transition metal paradigm.

### 3.3 Electrocatalytic Proton Reduction

We studied the catalytic activity of the synthesized bismuth­(III) catalysts [(L^1^)­BiCl_2_] **(1)**, [(L^2^)­BiCl_2_] **(2)**, [(L^3^)­BiCl_2_] **(3)**, and [(L^4^)­BiCl_2_] **(4)** for proton reduction in the presence of various external proton sources (Scheme 1). Since external acid strength can have a significant influence on the preferred catalytic pathway, the reactions of different acids (HX) of varying p*K*_a_ values in acetonitrile^90^ were used to check their effect on the catalytic proton reduction activity. Starting from a p*K*_a_ range of 6.10 in acetonitrile (dimethylformamidium triflate),^90^ around 11 different protic sources were explored for the proton reduction activity (Scheme 1).^90^

**Scheme 1:**
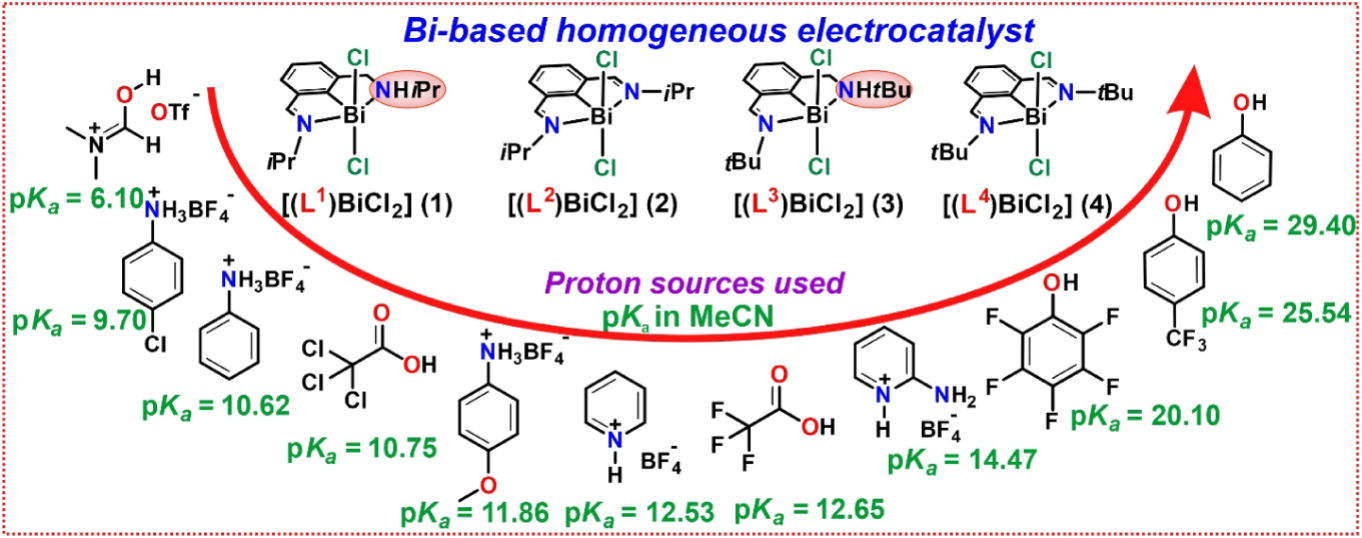
Catalysts and Proton Sources Used in This Study

Comprehensive electrochemical studies, including cyclic voltammetry (CV) and controlled potential electrolysis (CPE), were performed to assess key catalytic parameters (TOF, Faradaic efficiency, and overpotential) of the Bi-based catalysts, as well as to extract the plausible mechanistic steps operating in the catalytic cycles, for all the proton sources used as substrates for HER (Scheme 1). Adding different equivalents of acid to 0.5 mM solutions of catalysts [(L^1^)­BiCl_2_] **(1)**, [(L^2^)­BiCl_2_] **(2)**, [(L^3^)­BiCl_2_] **(3)**, and [(L^4^)­BiCl_2_] **(4)** and recording cyclic voltammograms (CVs) in acetonitrile at a scan rate of 100 mV/s resulted in a gradual enhancement of the cathodic current near the Bi­(I)/Bi­(III) redox couple for the different proton sources in acetonitrile^90^ (p*K*_a_ 6.10 to p*K*_a_ 29.40) upon increasing their concentration (Scheme 1 and Figures S58–S96, SI). The proton sources used in this study in acetonitrile were Dimethylformamidium triflate: p*K*_a_ = 6.10, *p*-chloroanilinium tetrafluoroborate: p*K*_a_ = 9.70, Anilinium tetrafluoroborate: p*K*_a_ = 10.62, Trichloroacetic acid: p*K*_a_ = 10.75, 4-methoxyanilinium tetrafluoroborate: p*K*_a_ = 11.86, Pyridinium tetrafluoroborate: p*K*_a_ = 12.53, Trifluoroacetic acid: p*K*_a_ = 12.65, 2-aminopyridinium tetrafluoroborate: p*K*_a_ = 14.47, Pentafluorophenol: p*K*_a_ = 20.10, 4-trifluoromethylphenol: p*K*_a_ = 25.54, and Phenol: p*K*_a_ = 29.40 (Scheme 1). The enhancement of the cathodic current suggested a catalytic proton reduction reaction toward the evolution of H_2_ gas, which was identified by gas chromatographic (GC) analysis with a thermal conductivity detector (TCD) (vide infra). The experimental results in all the cases reveal a linear dependence of current density on varying the concentration of proton sources giving insights into the order of the reaction components (vide infra). The TOF for each catalyst under different proton sources was calculated using the formula shown in eq 2:^98,99^

2 $$TOF=F\nu {n_{p}}^{3}{\left(\left(0.4463/n_{cat}\right)\left(i_{cat}/i_{p}\right)\right)}^{2}/RT$$

where ν is the scan rate in V/s, *n*_p_ is the number of electrons transferred between the catalyst redox couple (*n*_p_ = 2 for Bi^III^/Bi^I^ couple), *n*_cat_ is the number of electrons required for the conversion of the substrate (here *n*_cat_ = 2, as two electrons are required to convert two protons to give one H_2_), *i*_cat_ is the catalytic peak current, and *i*_p_ is the inherent current before the addition of the substrate (acid, here) (Note: *i*_cat_/*i*_p_ = *J*_cat_/*J*_p_, the ratio of current density). The results of TOF for different catalysts and different proton sources are summarized in the tables of SI (Tables S11–S20, SI). The S-shaped CV obtained for the Bi catalysts under proton reduction catalytic conditions as well as scan rate-independent catalytic current (Figures S153–S154, SI) suggests that the catalytic reaction is operating under pure kinetic conditions where the concentration of acid present on the electrode surface is equal to the concentration in the bulk solution.^100,101^ Thus, at a scan rate of 100 mV/s with an acid concentration of 100 mM in acetonitrile, the catalytic plateau current and thus the practical kinetic condition regime were reached for all the catalysts [(L^1^)­BiCl_2_] **(1)**, [(L^2^)­BiCl_2_] **(2)**, [(L^3^)­BiCl_2_] **(3)**, and [(L^4^)­BiCl_2_] **(4)**. In the presence of 100 mM *p*-Cl-anilinium tetrafluoroborate (p*K*_a_ = 9.70 in acetonitrile), with catalyst [(L^1^)­BiCl_2_] **(1)**, a catalytic wave for the reduction of protons was observed with a half-wave potential (*E*_cat/2_) of −1.41 V vs Fc/Fc^+^, corresponding to an overpotential of 0.94 V (Figure 3a, Table 1). Using 100 mM of 2-aminopyridinium (p*K*_a_ = 14.47 in acetonitrile), catalyst [(L^1^)­BiCl_2_] **(1)** exhibited an *E*_cat/2_ value of −1.75 vs Fc^+^/Fc, with an overall decrease in overpotential value to 0.86 V (Figure 3b, Table 1). For a weaker acid, like Pentafluorophenol (p*K*_a_ = 20.10 in acetonitrile), the *E*_cat/2_ value shifted further cathodically to −1.87 V vs Fc^+^/Fc, with an overpotential of 0.65 V observed for the hydrogen evolution reaction (Figure 3c, Table 1).

**Figure 3:**
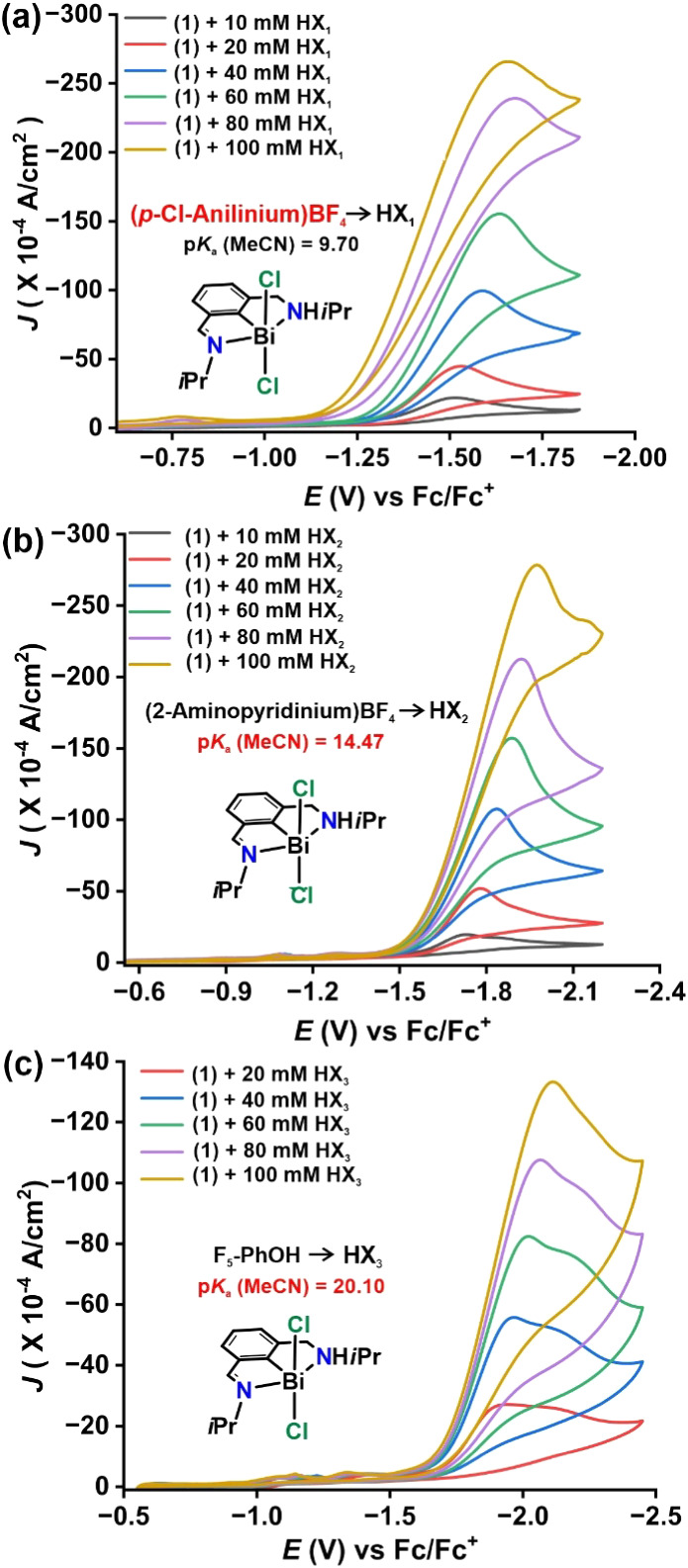
Cyclic voltammograms of 0.5 mM [(L^1^)­BiCl_2_] **(1)** in CH_3_CN with 0.1 M TBAPF_6_ supporting electrolyte under N_2_. Varying conc. of acid (a) *p*-Cl-anilinium tetrafluoroborate (p*K*_a_ = 9.70 in acetonitrile), (b) 2-aminopyridinium tetrafluoroborate (p*K*_a_ = 14.47 in acetonitrile), and (c) pentafluorophenol (p*K*_a_ = 20.10 in acetonitrile). Scan rate is 100 mV/s. The working electrode is glassy carbon (3 mm diameter), the counter electrode is platinum, and the pseudoreference electrode is Ag/AgCl wire with ferrocene (Fc) added as an internal reference.

**Table 1:** Representative Activity of Catalyst [(L^1^)­BiCl_2_] **(1)** and [(L^2^)­BiCl_2_] **(2)** at Different p*K*_a_ Ranges

Entry	Catalyst	p*K*_a_ in Acetonitrile^90^	*E*_cat/2_ (V vs Fc/Fc^+^)	Overpotential *η* (V)	TOF (s^–1^)
1.	[(L^1^)Bi^III^Cl_2_] **(1)**	6.10 (DMFH^+^OTf^–^)	–1.33	0.94	3.1 × 10^6^
2	[(L^2^)Bi^III^Cl_2_] **(2)**	6.10 (DMFH^+^OTf^–^)	–1.49	1.10	1.3 × 10^6^
3.	[(L^1^)Bi^III^Cl_2_] **(1)**	9.70 (*p*-Cl-Anilinium^+^BF_4_^–^)	–1.41	0.80	2.6 × 10^6^
4	[(L^2^)Bi^III^Cl_2_] **(2)**	9.70 (*p*-Cl-Anilinium^+^BF_4_^–^)	–1.46	0.86	7.4 × 10^5^
5.	[(L^1^)Bi^III^Cl_2_] **(1)**	12.65 (TFA)	–1.15	0.37	3.0 × 10^5^
6.	[(L^2^)Bi^III^Cl_2_] **(2)**	12.65 (TFA)	–1.43	0.65	6.3 × 10^4^
7.	[(L^1^)Bi^III^Cl_2_] **(1)**	14.47 (2-Aminopyridinium^+^BF_4_^–^)	–1.75	0.86	2.8 × 10^6^
8.	[(L^2^)Bi^III^Cl_2_] **(2)**	14.47 (2-Aminopyridinium^+^BF_4_^–^)	–1.83	0.95	8.8 × 10^5^
9.	[(L^1^)Bi^III^Cl_2_] **(1)**	20.10 (Pentafluorophenol)	–1.87	0.65	7.1 × 10^5^
10.	[(L^2^)Bi^III^Cl_2_] **(2)**	20.10 (Pentafluorophenol)	–1.99	0.76	1.5 × 10^5^

Compared to catalyst [(L^1^)­BiCl_2_] **(1)**, which features a mixed imine–amine ligand backbone, the bis-imine ligand-coordinated Bi­(III) catalyst [(L^2^)­BiCl_2_] **(2)** having a slightly more positive standard reduction potential (*E*_red_ value −1.09 V for [L^2^BiCl_2_] **(2)** vs −1.14 V vs Fc/Fc^+^ for [(L^1^)­BiCl_2_] **(1)**) exhibited significantly reduced catalytic activity (Figure 4, Table 1). In the presence of 100 mM of different acids (*p*-Cl-anilinium tetrafluoroborate: p*K*_a_ = 9.70 in acetonitrile, 2-aminopyridinium: p*K*_a_ = 14.47 in acetonitrile, pentafluorophenol: p*K*_a_ = 20.10 in acetonitrile) (Figure 4a, 4b, 4c), with catalyst [(L^2^)­BiCl_2_] **(2)**, an increase in half-wave potential (*E*_cat/2_) and overpotential (*η*) as compared to that of catalyst [(L^1^)­BiCl_2_] **(1)** was observed (Figure 4, Table 1). Interestingly, unlike reports on transition metal systems, where HER activity has been reported to be boosted by added water to the proton sources through water-containing networks,^19^ no such effects were observed for the HER activity of catalysts **(1)** and **(2)**.

**Figure 4:**
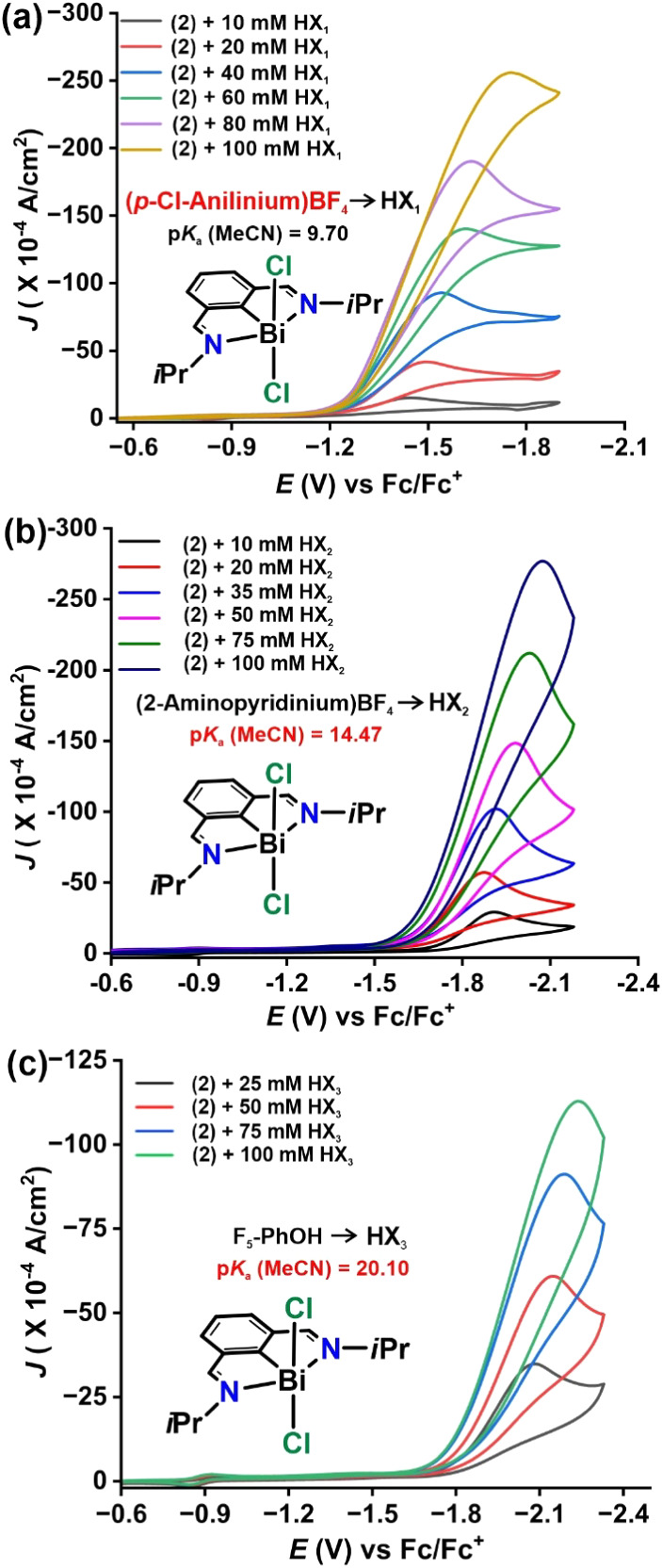
Cylic voltammograms of 0.5 mM [(L^2^)­BiCl_2_] **(2)** in CH_3_CN with 0.1 M TBAPF_6_ supporting electrolyte under N_2_. Varying conc. of acid (a) *p*-Cl-anilinium tetrafluoroborate (p*K*_a_ = 9.70 in acetonitrile), (b) 2-aminopyridinium tetrafluoroborate (p*K*_a_ = 14.47 in acetonitrile), and (c) pentafluorophenol (p*K*_a_ = 20.10 in acetonitrile). Scan rate is 100 mV/s. The working electrode is glassy carbon (3 mm diameter), the counter electrode is platinum, and the pseudoreference electrode is Ag/AgCl wire with ferrocene (Fc) added as an internal reference.

However, despite a slightly lower overpotential trend for catalyst [(L^1^)­BiCl_2_] **(1)** across the p*K*_a_ range as compared to catalyst [(L^2^)­BiCl_2_] **(2)**, TOF calculations using eq 2 revealed consistently higher catalytic activity for catalyst [(L^1^)­BiCl_2_] **(1)** relative to catalyst [(L^2^)­BiCl_2_] **(2)** (Table 1 and Figure 5). This enhanced performance of catalyst [(L^1^)­BiCl_2_] **(1)** prompted further investigation into the structural, electronic, and mechanistic factors responsible for its superior catalytic efficiency. A similar trend of enhanced activity for the *t*-Butyl-substituted imine-amine catalyst ([(L^3^)­BiCl_2_] **3**) was observed when compared with the analogous *t*-Butyl-substituted bis-imine catalyst [(L^4^)­BiCl_2_] **(4)** (Tables S13, S14 and Figure S149, SI). Among catalysts [(L^1^)­BiCl_2_] **(1)**, [(L^2^)­BiCl_2_] **(2)**, [(L^3^)­BiCl_2_] **(3)**, and [(L^4^)­BiCl_2_] **(4)**, catalyst [(L^1^)­BiCl_2_] **(1)** showed the highest TOF (or *k*_obs_) of 3.10 × 10^6^ s^–1^ (at p*K*_a_ 6.10 in acetonitrile) across the Bi­(III) catalyst series, which surpasses the activity to some of the best-reported, well-known transition metal HER catalysts^11^ as well as the natural iron–hydrogenase enzyme.^21^

**Figure 5:**
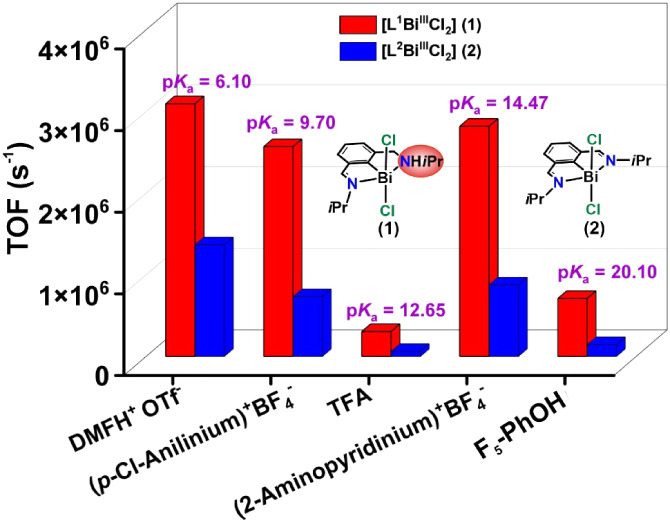
Comparative plot of TOF (s^–1^) values for catalyst [(L^1^)­BiCl_2_] **(1)** (red bar) and catalyst [(L^2^)­BiCl_2_] **(2)** (blue bar) (0.5 mM in acetonitrile) toward electrochemical proton reduction activity using 0.1 M of external proton sources of varied p*K*_a_ range in acetonitrile (only five representative proton sources are shown here).

### 3.4 Effect of Potential–p*K*_a_ Study

Aqueous potential–pH diagrams, commonly called Pourbaix diagrams, have proven to be of great practical and fundamental utility for benchmarking and understanding the mechanisms of hydrogen evolution catalysts.^90^ However, though water should ideally be used as the solvent and proton source, most molecular electrocatalysts are commonly employed in organic solvents due to their limited solubility or poor stability in water. The thermodynamics of proton transfer in nonaqueous solvents are dictated by p*K*_a_ differences, rather than the pH; thus, the free energy axis for a nonaqueous Pourbaix diagram should also depend on the p*K*_a_. PCET in dry nonaqueous solvents is generally viewed to occur through the direct interaction of the proton donor with the proton acceptor.^90^ Construction of potential–p*K*_a_ diagrams enables direct visualization of the interplay between reduction potential and protonation equilibria, allowing extraction of thermodynamic parameters relevant to catalytic intermediates. While extensive studies have elucidated PCET mechanisms in transition-metal-based hydrogen evolution catalysts,^27,91,95–97^ analogous systematic analyses of main-group systems remain comparatively underexplored. This distinction is mechanistically significant: main-group frameworks lack accessible *d*-orbital manifolds and often exhibit distinct charge localization, bond polarization, and proton affinity profiles. As a result, the coupling between electron transfer and proton transfer steps may differ fundamentally from that observed in transition metal systems. For HER catalysis, potential–p*K*_a_ diagrams would provide a unified thermochemical framework to evaluate catalytic trends across varying acid strengths and applied potentials. Analysis of onset potentials as a function of acid p*K*_a_ allows identification of regimes in which catalysis is limited by electron transfer, proton transfer, or concerted PCET.^90^ Importantly, such mapping enables the identification of optimal acidity windows that balance favorable protonation equilibria with minimal overpotential. Accordingly, potential–p*K*_a_ analysis would offer quantitative insight into driving forces, rate-limiting PCET steps, and hydride formation energetics. This approach is expected to establish design principles for main-group HER catalysts aimed at maximizing TOF while minimizing effective overpotential (*η*) under electrocatalytic conditions. Our analyses over a wide range of external proton source p*K*_a_ values for the main-group bismuth-based homogeneous electrocatalysts ([(L^1^)­BiCl_2_] **(1)**, [(L^2^)­BiCl_2_] **(2)**, [(L^3^)­BiCl_2_] **(3)**, [(L^4^)­BiCl_2_] **(4)**) reveal a clear and systematic trend: the catalytic half-wave potential (*E*_cat/2_) shifts to more negative values with increasing p*K*_a_ of the external proton source in acetonitrile (Figure 6a and also Figure S152 and Tables S11–S14, SI). In contrast, no clear correlation was observed between overpotential *η* vs p*K*_a_ profile across the range of acids examined for all the catalysts ([(L^1^)­BiCl_2_] **(1)**, [(L^2^)­BiCl_2_] **(2)**, [(L^3^)­BiCl_2_] **(3)**, [(L^4^)­BiCl_2_] **(4)**) (Figure 6b, and also see Tables S11–S14, SI for other catalysts, Tables S15–S20) indicating that factors beyond simple proton acidity, including structural and electronic effects of the proton donor, also contribute significantly to catalytic energetics (Figure 6b).

**Figure 6:**
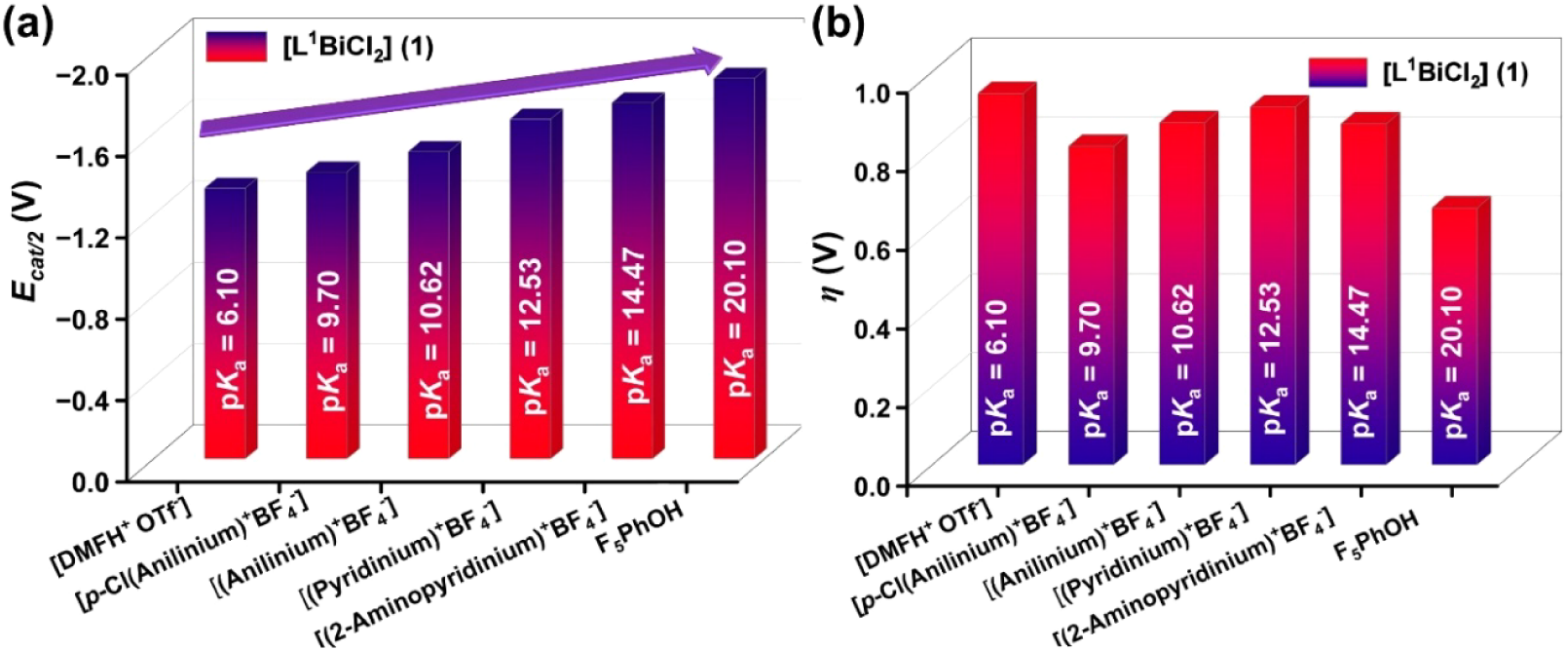
(a) Plot of catalytic half wave potential (*E*_cat/2_, V) for catalyst [(L^1^)­BiCl_2_] **(1)** vs p*K*_a_ for different external proton sources in acetonitrile toward HER. (b) Plot of overpotential^a^ (*η*, V) vs p*K*_a_ in acetonitrile for different external proton sources using the catalyst [(L^1^)­Bi^III^Cl_2_] **(1)** for electrochemical HER. ^a^Overpotential was calculated using *E*_cat/2_.

In homogeneous catalysis, Hammett-type linear free energy relationships correlating reaction rates or equilibrium constants with the p*K*_a_ or acidity of reactants or intermediates are widely employed to elucidate reaction mechanisms and rationalize reactivity trends of molecular complexes.^102^ Such relationships rely on the premise that a linear correlation exists between the activation free energy (Δ*G* ^‡^) and the reaction free energy (Δ*G*_rxn_), implying that the kinetics of the reaction depends linearly on the thermodynamic driving force of the reaction. In this study, we have tried to develop such a Hammett-type linear scaling relation. In this context, we sought to establish a Hammett-type linear scaling relationship for HER mediated by the catalyst [(L^1^)­BiCl_2_] **(1)**. To interrogate the p*K*_a_ dependence of external proton sources used for the catalyst [(L^1^)­BiCl_2_] **(1)** toward HER, and the associated modulation of the effective overpotential (*η*), we examined the electronic influence of the proton source in a systematic manner. A series of *para*-X-substituted anilinium tetrafluoroborate salts (*p*-X–C_6_H_4_NH_3_^+^BF_4_^–^) was employed, incorporating both electron-donating (X = OMe) and electron-withdrawing substituents (X = Cl), thereby enabling controlled variation of proton donor electronics within a conserved structural framework. As the electron density on the *para*-X-substituted anilinium backbone increases, the corresponding p*K*_a_ in acetonitrile increases (*p*-Cl–C_6_H_4_NH_3_^+^BF_4_^–^, p*K*_a_ = 9.70; *p*-H–C_6_H_4_NH_3_^+^BF_4_^–^, p*K*_a_ = 10.62; *p*-OMe–C_6_H_4_NH_3_^+^BF_4_^–^, p*K*_a_ = 11.86), accompanied by an increase in η for HER activity by the catalyst [L^1^BiCl_2_] **(1)**. Accordingly, a linear correlation is observed between *η* and p*K*_a_ in the electrochemical proton reduction by the catalyst [L^1^BiCl_2_] **(1)** (Figure 7a). Consistent with a Hammett-type description, *η* exhibits an inverse linear dependence on the *σ*_p_ substituent parameter, thereby establishing a direct electronic scaling relationship between proton donor substituent effects (*p*-X–C_6_H_4_NH_3_^+^BF_4_^–^) and the energetic requirements of catalysis within a conserved structural framework (Figure 7b). The Hammett correlation is indicative of the fact that more acidic proton donors would likely facilitate the hydrogen release step from any Bi–H intermediate formed in the reaction pathway more effectively (vide infra). This trend further suggests that the rate-determining transition state likely involves the development of partial positive charge on the proton donor (or conversely, increasing hydridic character at the putative Bi–H moiety) during H–H bond formation. Electron-withdrawing substituents, which increase proton acidity, thus stabilize this charge distribution and thereby lower the energetic barrier for hydrogen evolution. Such behavior is consistent with a transition state involving concerted proton transfer and hydride coupling from the Bi–H intermediate, rather than a simple protonation event. Notably, this trend is also supported by DFT-calculated energetics discussed later (Figure S232). Collectively, these Hammett analyses implicate a mechanistic pathway in which the electronic properties of the proton donor directly modulate the energetics of the H_2_-release transition state, thereby providing a framework for the rational design of HER catalysts operating at lower energetic cost.

**Figure 7:**
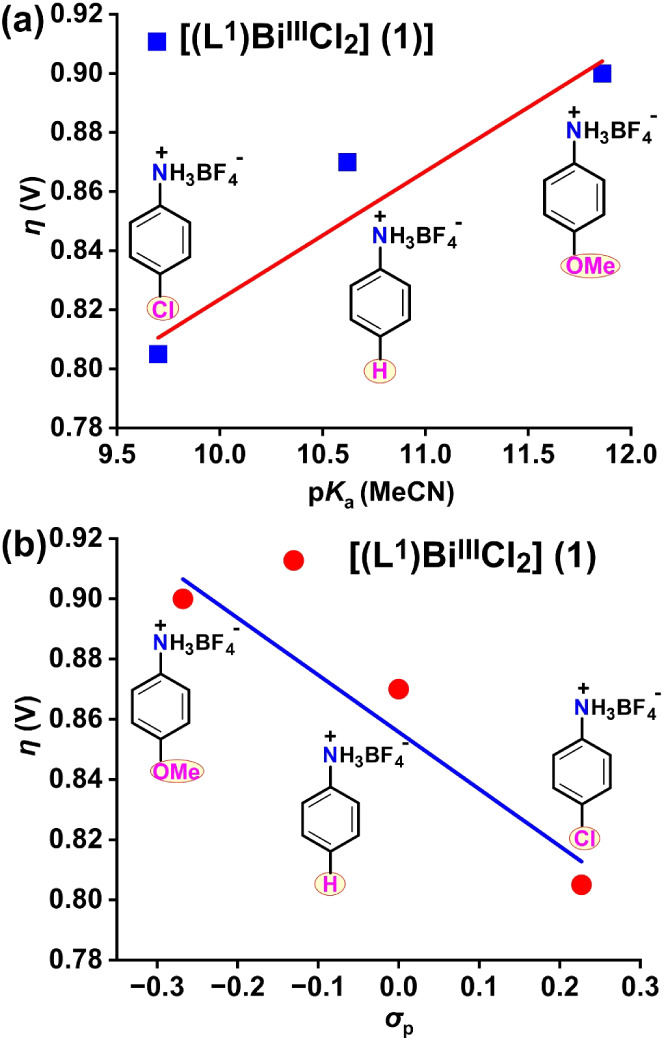
(a) Plot of overpotential vs p*K*_a_ in acetonitrile for different *p*-X-substituted anilinium tetrafluoroborate salts as external proton sources for electrochemical HER by catalyst [(L^1^)­BiCl_2_] **(1)**. (b) Hammett-type linear scale relationship in the plot of overpotential vs electronic factor of the external proton source (*σ*_p_) for the proton reduction activity by catalyst [(L^1^)­BiCl_2_] **(1)** with different *p*-X-substituted anilinium tetrafluoroborate salts (X = Cl, H, OMe).

An analogous overpotential–p*K*_a_ dependence was observed for the remaining Bi-based catalysts [(L^2^)­BiCl_2_] **(2)**, [(L^3^)­BiCl_2_] **(3)**, [(L^4^)­BiCl_2_] **(4)**, each displaying systematic modulation of catalytic response with proton donor acidity (Figure S152, SI). Notably, catalysts [(L^1^)­BiCl_2_] **(1)** and [(L^3^)­BiCl_2_] **(3)** each having a pendant amine arm along with the imine arm consistently exhibited higher activity relative to their bis-imine analogues [(L^2^)­BiCl_2_] **(2)** and [(L^4^)­BiCl_2_] **(4)**, respectively, under comparable conditions (Table S11, Table S13 and Figure S151, SI). The enhanced performance of [(L^1^)­BiCl_2_] **(1)** and [(L^3^)­BiCl_2_] **(3)** thus prompted further mechanistic investigation to elucidate the structural and electronic origins of the superior catalytic efficiency of [(L^1^)­BiCl_2_] **(1)** and [(L^3^)­BiCl_2_] **(3)** (vide infra).

### 3.5 Effect of Potential-Activity Study: Scaling Relations and Tafel Slope

Efficient catalytic systems are expected to deliver high TOFs at minimal overpotentials (*η*), ideally operating near the thermodynamic equilibrium potential under the specified experimental conditions. Achieving such efficiency in PCET catalysis remains a significant challenge due to the intricate interplay between electron transfer, proton transfer, and intermediate stability.^94^ Molecular electrocatalysts based on transition metals have harnessed considerable attention in this regard, as they offer precise structural tunability and facilitate detailed mechanistic insights relative to heterogeneous electrocatalysts.^27,95–97^ In contrast, systematic evaluation and benchmarking of redox-active main-group electrocatalysts, particularly Bi-based homogeneous systems, have remained largely unexplored. Establishing such benchmarks is anticipated to expand the scope of main-group redox catalysis, positioning it as a viable and potentially complementary alternative to conventional *d-*block catalysis, with the prospect of accessing reactivity profiles that may surpass those of rare and expensive transition metals or enable new transformations that remained challenging within traditional frameworks.^55,65^

In order to address the challenge in molecular PCET catalysis to increase the TOF without compensating changes in the *η*, we correlated *η* with the log­(TOF) values for the series of Bi-based homogeneous electrocatalysts used in our study ([(L^1^)­BiCl_2_] **(1)**, [(L^2^)­BiCl_2_] **(2)**, [(L^3^)­BiCl_2_] **(3)**, [(L^4^)­BiCl_2_] **(4)**). For the representative acids having p*K*_a_ = 10.75 (TCA = Trichloroacetic acid), p*K*_a_ = 11.86 (*p*-Anisidinium tetrafluoroborate), and p*K*_a_ = 12.65 (TFA = Trifluoroacetic acid) in acetonitrile, the activity vs overpotential plot for all four Bi catalysts was analyzed. For each acid examined, plots of log­(TOF) vs *η* for catalysts ([(L^1^)­BiCl_2_] **(1)**, [(L^2^)­BiCl_2_] **(2)**, [(L^3^)­BiCl_2_] **(3)**, [(L^4^)­BiCl_2_] **(4)**) exhibit a linear correlation with a positive slope (Figure 8). This trend is consistent with a molecular scaling relationship, wherein enhanced catalytic rates are attained at the expense of increased overpotential.^103^ Such linear dependence highlights the inherent trade-off between turnover frequency and thermodynamic driving force within this series of Bi-based homogeneous electrocatalysts. However, in the linear free-energy relationship (LFER) analysis, catalyst [(L^1^)­BiCl_2_] **(1)** with a pendant amine arm and *i*Pr substitution and catalyst [(L^3^)­BiCl_2_] **(3)** with a pendant amine arm and *t*Bu substitution in the ligand framework deviate from the established scaling relationship and exhibit an enhanced TOF than predicted from the linear correlation (Figure 8a, 8b, 8c, 8d, also Figures S155–S156). This positive deviation suggests that [(L^1^)­BiCl_2_] **(1)** and [(L^3^)­BiCl_2_] **(3)** likely benefit from an additional kinetic advantage beyond that dictated solely by thermodynamic driving force, indicative of a distinct mechanistic contribution arising from ligand architecture, most likely associated with secondary coordination sphere (SCS) effects that facilitate proton transfer and enhance catalytic efficiency^104^ (vide infra). Notably, for a given catalyst, namely [(L^1^)­BiCl_2_] **(1)** or [(L^2^)­BiCl_2_] **(2)**, plots of log­(TOF) vs overpotential (*η*) obtained using proton sources of varying p*K*_a_ exhibited a clear linear relationship, with catalytic activity increasing at higher overpotentials (Figure S157, SI).

**Figure 8:**
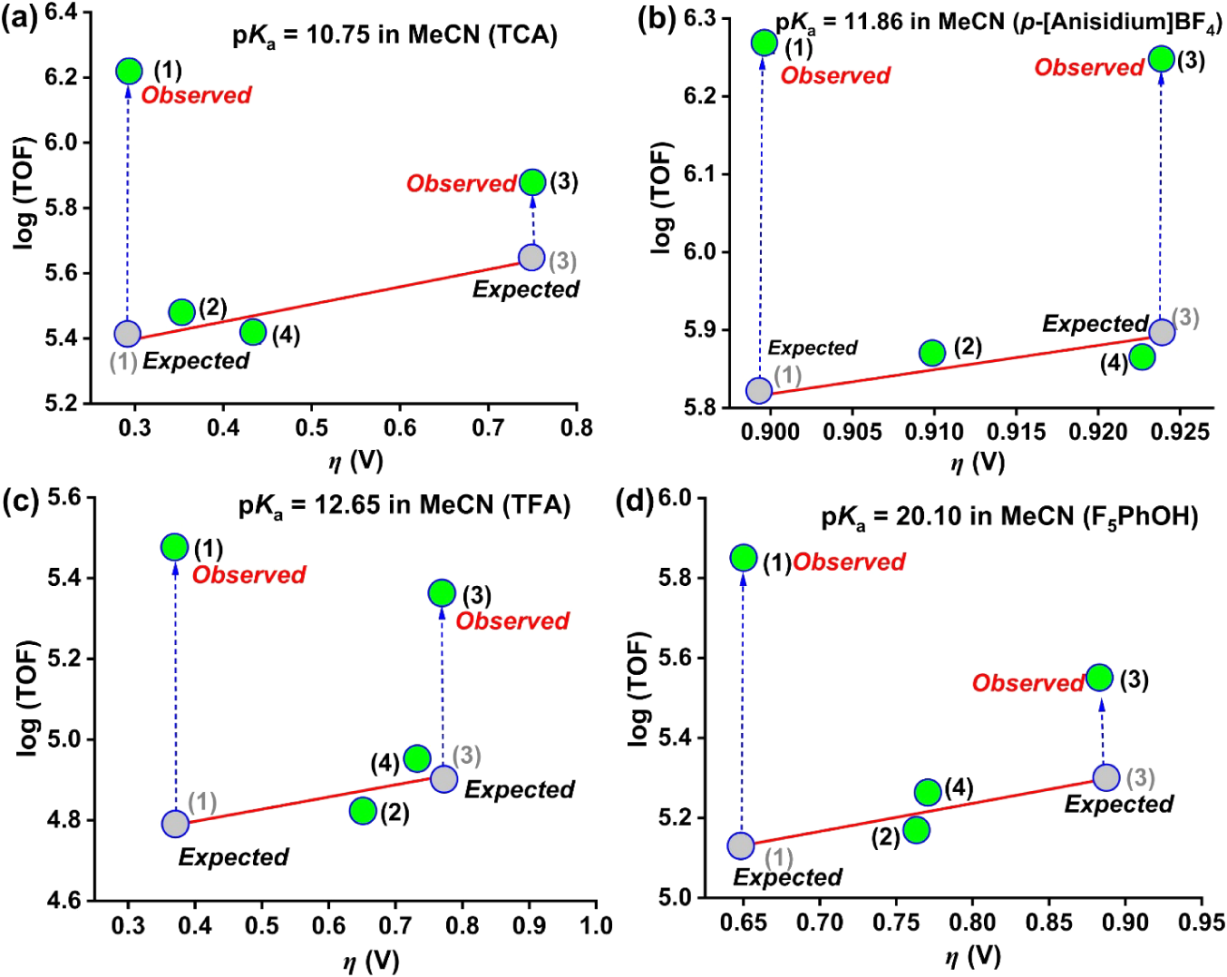
Scaling of log­(TOF) with η (V) for HER in acetonitrile catalyzed by Bi-based homogeneous catalysts: [(L^1^)­BiCl_2_] **(1)**, [(L^2^)­BiCl_2_] **(2)**, [(L^3^)­BiCl_2_] **(3)**, and [(L^4^)­BiCl_2_] **(4)** under nitrogen with different proton sources (0.1 M); (a) trichloroacetic acid (p*K*_a_ = 10.75 in acetonitrile); (b) *p*-Anisidinium tetrafluoroborate (p*K*_a_ = 11.86 in acetonitrile); (c) trifluoroacetic acid (p*K*_a_ = 12.65 in acetonitrile); (d) pentafluorophenol (p*K*_a_ = 20.10 in acetonitrile). Conditions: [Complex] = 0.5 mM, [nBu_4_NPF_6_] = 0.1 M, and scan rate = 100 mV/s were used to determine the TOF and *η* values (*η* was calculated based on *E*_cat/2_).

Another important parameter for evaluating electrochemical catalytic efficiency is the Tafel slope, defined as the overpotential required to increase the current density by 1 order of magnitude and typically expressed in mV dec^–1^.^105^ A lower Tafel slope therefore indicates more favorable catalytic kinetics, as a smaller increase in overpotential is required to achieve higher current densities. Under appropriate conditions, the Tafel slope can also provide mechanistic insight into the rate-determining step.^105^ Comparative Tafel slope analyses were performed for catalysts ([(L^1^)­BiCl_2_] **(1)**, [(L^2^)­BiCl_2_] **(2)**, [(L^3^)­BiCl_2_] **(3)**, [(L^4^)­BiCl_2_] **(4)**) under similar experimental conditions using anilinium tetrafluoroborate (p*K*_a_ = 10.62) and 2-aminopyridinium tetrafluoroborate (p*K*_a_ = 14.47) in acetonitrile (Figure 9). In both cases, catalyst [(L^1^)­BiCl_2_] **(1)** and catalyst [(L^3^)­BiCl_2_] **(3)** exhibit lower Tafel slopes compared to the analogue lacking −*NH* pendant arm, indicating more favorable catalytic kinetics. This observation further strengthens our proposal of the involvement of proton assistance from the pendant amine arm of the pincer ligand, which facilitates and enhances HER activity (vide infra). Together, these results highlight the beneficial role of the second-sphere pendant −*NH* proton in catalysts [(L^1^)­BiCl_2_] **(1)** (or **(3)**) which facilitates proton mobility and leads to faster and more energy-efficient catalysis relative to their congeners lacking such functionality in the ligand backbone (catalyst [(L^2^)­BiCl_2_] **(2) or (4)**).

**Figure 9:**
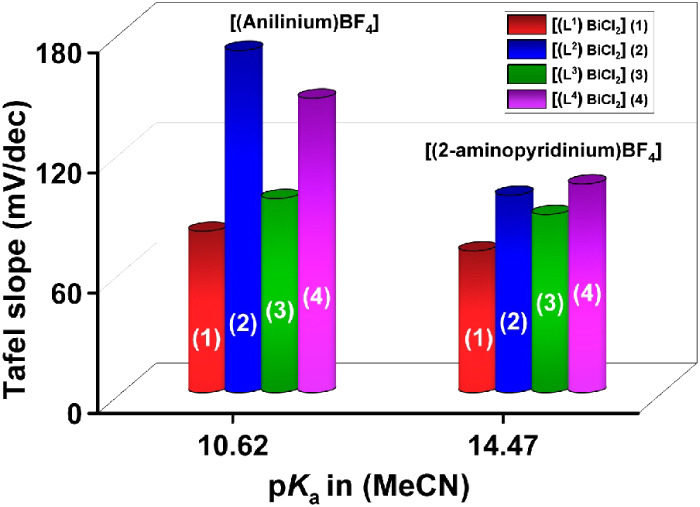
Tafel slope comparison of the bismuth-based electrocatalysts [(L^1^)­BiCl_2_] **(1)**, [(L^2^)­BiCl_2_] **(2)**, [(L^3^)­BiCl_2_] **(3)**, and [(L^4^)­BiCl_2_] **(4)** using anilinium tetrafluoroborate (p*K*_a_ = 10.62 in acetonitrile) and 2-aminopyridinium tetrafluoroborate (p*K*_a_ = 14.47 in acetonitrile).

Overall, the experimental results reveal that although enhanced HER rates were obtained for both catalysts [(L^1^)­BiCl_2_] **(1)** and [(L^3^)­BiCl_2_] **(3)**, having −NH functionality, catalyst [(L^1^)­BiCl_2_] **(1)** consistently exhibited higher activity than catalyst [(L^3^)­BiCl_2_] **(3)**. Similarly, catalyst [(L^2^)­BiCl_2_] **(2)** displayed slightly higher activity than catalyst [(L^3^)­BiCl_2_] **(3)**, albeit less prominently. To further probe the influence of substituent effects (Me, *i*Pr, and *t*Bu) in both bis-imine as well as mixed amine-imine ligand frameworks of Bi catalysts on HER reactivity and to gain mechanistic insight into the catalytic pathway, corresponding methyl-substituted analogues, bis-imine [(L^5^)­BiCl_2_] **(5)** and amine-imine [(L^6^)­BiCl_2_] **(6)**, were synthesized and evaluated for proton reduction activity (Scheme 1, Scheme S11, Scheme S13, Table S15, Tables S19–S20, SI). Experimentally, the activity trend among bis-imine catalysts [(L^2^)­BiCl_2_] **(2**), [(L^4^)­BiCl_2_] **(4**), and [(L^5^)­BiCl_2_] (**5**) showed only marginal variation, suggesting that steric effects do not significantly influence catalytic activity within this series (Tables S19–S20). Likewise, within the amine–imine series, only modest differences in activity were observed upon varying the substituent from Me to *i*Pr or *t*Bu (catalysts [(L^1^)­BiCl_2_] **(1)**, [(L^3^)­BiCl_2_] **(3)**, and [(L^6^)­BiCl_2_] **(6)**) (Table S11, Table S13, Table S19, Table S20 also Figures S236–S237 for DFT details). Interestingly, though the role of steric effects is not very prominent, in all these cases, catalysts bearing pendant −*NH* functionality consistently exhibited enhanced HER activity compared to analogous systems lacking −*NH* assistance. Collectively, these results support the conclusion that proton-assisted kinetics mediated by the pendant −*NH* group constitute the dominant factor responsible for overcoming the conventional activity–overpotential scaling relationship, whereas steric effects contribute only marginally to the modulation of catalytic efficiency (Figure 8 and also Figures S155–S156, SI).

### 3.6 Direct Experimental Probe of −*NH* Functionality in Modulating HER Activity

From the above experimental results, it is evident that catalysts supported by mixed amine–imine ligands having −*NH* functionality as a second sphere residue (catalysts [(L^1^)­BiCl_2_] **(1)**, [(L^3^)­BiCl_2_] **(3)**, and [(L^6^)­BiCl_2_] **(6)**) exhibit enhanced TOF values irrespective of the substituent effects (Me, *i*Pr, and *t*Bu). This observation identifies the −*NH*-containing catalysts as more promising HER electrocatalysts compared to their bis-imine congeners (catalysts [(L^2^)­BiCl_2_] **(2)**, [(L^4^)­BiCl_2_] **(4)**, and [(L^5^)­BiCl_2_] **(5)**), thereby implicating the pivotal role of the pendant *–* *NH* functionality in modulating catalytic activity. However, a comparison between mixed amine–imine systems with and without *–* *NH* functionality would provide a more comprehensive understanding of the role of the pendant *–* *NH* group, beyond the comparison with the bis-imine analogues discussed thus far. Accordingly, we synthesized a mixed amine–imine ligand featuring an *N*-methylated amine arm to block the *–* *NH* proton functionality, while largely preserving the electronic properties of the parent system (Scheme S15, Figures S41–S42, Figure S49, Figure S77). The corresponding complex, [(L^7^)­BiCl_2_] **(7)**, exhibited a reduction potential (*E*_red_) of −1.11 V vs Fc/Fc^+^ closely comparable to that of catalyst [(L^1^)­BiCl_2_] **(1)** (*E*_red_ = −1.14 V vs Fc/Fc^+^) indicating similar electronic characteristics for the two systems. To directly probe the role of the *–*NH proton assistance, comparative HER studies were performed for catalysts [(L^1^)­BiCl_2_] **(1)** and [(L^7^)­BiCl_2_] **(7)** using both pentafluorophenol (p*K*_a_ = 20.10 in acetonitrile) as well as TFA (p*K*_a_ = 12.65 in acetonitrile) as proton sources. In both cases, catalyst [(L^1^)­BiCl_2_] **(1)**, possessing the pendant amine −NH group, exhibited higher TOF values and lower overpotentials than the *N*-methylated analogue [(L^7^)­BiCl_2_] **(7)** (Figure 10a and 10b, Tables S17–S18, SI). These observations provide direct experimental evidence for the beneficial role of the secondary coordination sphere *–*NH proton assistance in enhancing catalytic HER efficiency.

**Figure 10:**
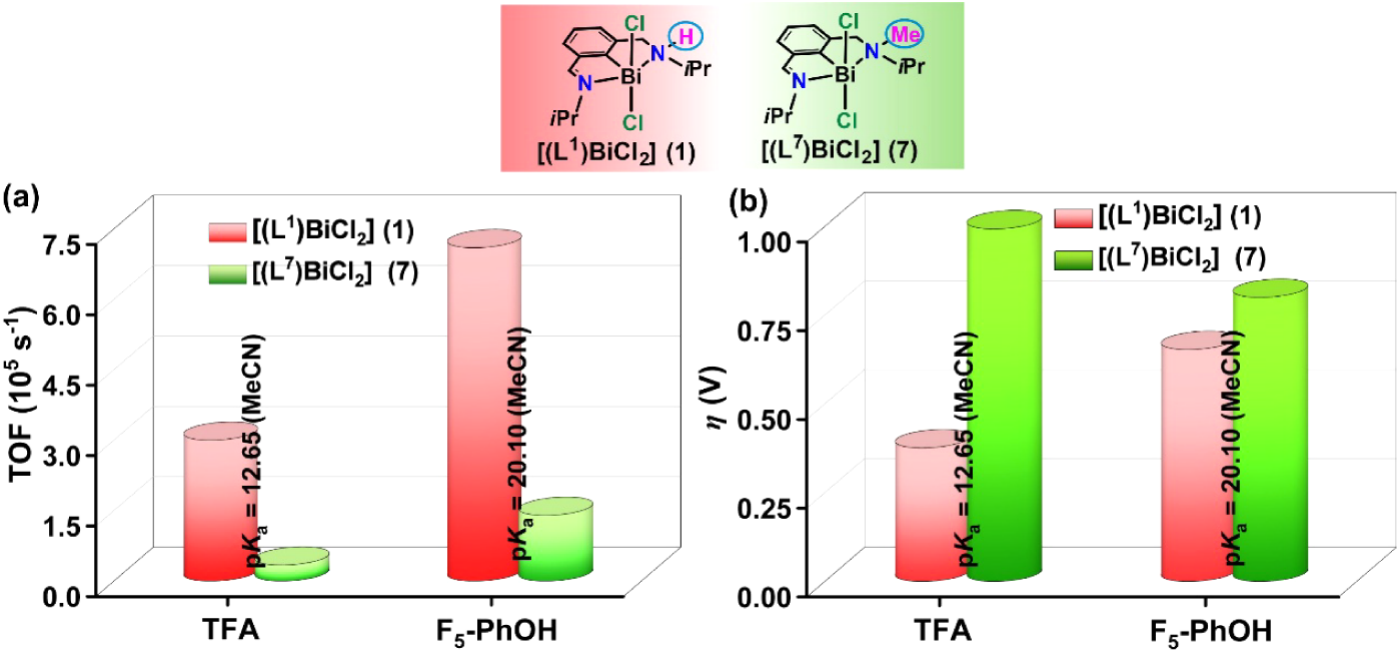
Comparative activity of bismuth-based electrocatalysts [(L^1^)­BiCl_2_] **(1)** having *N–H* functionality (red bar) and [(L^7^)­BiCl_2_] **(7)** having *N–Me* substitution (green bar), showing a plot of (a) TOF (s^–1^) for electrochemical HER and (b) corresponding overpotential η (V) using proton sources TFA (p*K*_a_ = 10.65 in acetonitrile) and pentafluorophenol (p*K*_a_ = 20.10 in acetonitrile) (η was calculated based on *E*_cat/2_).

### 3.7 FOWA and Kinetic Insights

As discussed above, the performance of homogeneous electrocatalysts is often evaluated by comparing the catalytic plateau currents observed in cyclic voltammograms (eq 2).^88,99^ However, side phenomena such as substrate depletion, catalyst deactivation, or product inhibition during electrocatalysis can prevent the catalytic currents from reaching an ideal plateau.^106^ Consequently, the accurate determination of turnover frequencies based solely on catalytic peak currents using the Randles–Ševčík equation (eqs 1 and 2) may lead to unreliable kinetic estimates.^88^ These limitations can be addressed by applying foot-of-the-wave analysis (FOWA) to cyclic voltammetry data.^107^ FOWA extracts kinetic information from the onset, or “foot”, of the catalytic wave—a region where the current–potential response follows near-ideal behavior and is minimally influenced by secondary processes such as catalyst degradation or substrate depletion. By analyzing this region, reliable kinetic parameters such as the apparent rate constant (*k*_obs_) and the maximum turnover frequency (TOF_max_) can be determined.^33,100,106^ Accordingly, FOWA was employed to estimate the TOF_max_ values for all four catalysts [(L^1^)­BiCl_2_] **(1)**, [(L^2^)­BiCl_2_] **(2)**, [(L^3^)­BiCl_2_] **(3)**, [(L^4^)­BiCl_2_] **(4)** for different external proton sources spanning a wide p*K*_a_ range in acetonitrile^90^ under comparable experimental conditions (Anisidinium, p*K*_a_ = 11.86; TFA, p*K*_a_ = 12.65; 2-Aminopyridinium, p*K*_a_ = 14.47; Pentafluorophenol, p*K*_a_ = 20.10) (Figures S158–S179, SI). Notably, the scan rate (ν)-independent behavior of the FOWA slopes over the range (ν = 20 mV s^–1^, 50 mV s^–1^, 100 mV s^–1^, and 200 mV s^–1^) indicated that the catalytic response originates from a purely kinetically controlled regime before mass-transport and substrate depletion effects become significant^107^ (Figures S180–S183, SI). Furthermore, the extracted TOF values remained consistent across the examined scan rate range, further validating the reliability and robustness of the kinetic parameters obtained from the FOWA (Tables S21–S24, SI). In all cases, the FOWA-derived results revealed enhanced HER rates for catalysts [(L^1^)­BiCl_2_] **(1)** (and [(L^3^)­BiCl_2_] **(3)**) compared to their bis-imine analogues [(L^2^)­BiCl_2_] **(2)** (and [(L^4^)­BiCl_2_] **(4)**) across the investigated p*K*_a_ range in acetonitrile. This observation clearly highlights the beneficial role of the pendant −*NH* group in the secondary coordination sphere of the ligand framework in [(L^1^)­BiCl_2_] **(1)** (and [(L^3^)­BiCl_2_] **(3)**), which likely assists through a proton-assisted mechanism to enhance the catalytic efficiency (vide infra). To the best of our knowledge, this study represents the first application of FOWA to evaluate the kinetics of a redox-active main-group electrocatalyst, providing direct access to intrinsic catalytic parameters such as TOF_max_ (or *k*_obs_). These findings further demonstrate that appropriately designed main-group catalysts can achieve efficient electrocatalytic performance, highlighting the emerging potential of *p*-block systems as viable alternatives to traditional transition-metal-based HER electrocatalysts.

In order to get insight into the reaction mechanism and determine the dependence of the reaction on individual components, the rate dependence on both catalyst and acid concentration was evaluated.^88^ Rate dependence studies using 0.1 M *p*-anisidinium tetrafluoroborate as the external proton source (p*K*_a_ = 11.86 in acetonitrile) with the Bi-based homogeneous electrocatalysts [(L^1^)­BiCl_2_] **(1)** and [(L^2^)­BiCl_2_] **(2)** revealed a first-order dependence on catalyst concentration (Figures S130–S131, SI). Furthermore, a linear dependence of the catalytic current density on the concentration of a wide range of proton sources (11 acids spanning a broad p*K*_a_ range in acetonitrile, Scheme 1) indicates a second-order dependence on the proton source concentration for all Bi-based homogeneous catalysts ([(L^1^)­BiCl_2_] **(1)**, [(L^2^)­BiCl_2_] **(2)**, [(L^3^)­BiCl_2_] **(3)**, [(L^4^)­BiCl_2_] **(4)**) (Figures S79–S125, SI). Such kinetic behavior contrasts with many transition metal HER systems^108^ but closely resembles that reported for main-group Sb–corrole electrocatalysts, where a second-order dependence on the proton source and an ECEC-type mechanism has been proposed.^57^

Based on the experimental observations, we hypothesize an ECEC mechanism (vide infra) to be operative for the Bi-based catalysts and calculated reaction rates *k*_1_ and *k*_2_.^28,53,100,108,109^ FOWA was applied to estimate the rate constant for the first chemical step (*k*_1_) of hydrogen evolution using TFA as the proton source (p*K*_a_ = 12.65 in acetonitrile)^90^ by catalysts [(L^1^)­BiCl_2_] **(1)** and [(L^2^)­BiCl_2_] **(2)**. A linear dependence of *k*_FOWA_ on acid concentration was observed, yielding second-order rate constant *k*_1_ value of 2.64 × 10^10^ M^–1^ s^–1^ for catalyst [(L^1^)­BiCl_2_] **(1)** and 7.87 × 10^9^ M^–1^ s^–1^ for catalyst [(L^2^)­BiCl_2_] **(2)** (Figure S190a–S190b, SI). The rate constant for the second protonation step *k*_2_ for catalyst [(L^1^)­BiCl_2_] **(1)** and [(L^2^)­BiCl_2_] **(2)** was subsequently estimated from TOF_max_ values derived from FOWA at varying acid concentrations using the equation *k*_obs_ = *k*_2_[H^+^], which afforded *k*_2_ values of 3.1 × 10^9^ M^–1^ s^–1^ and 9.8 × 10^8^ M^–1^ s^–1^ for catalysts [(L^1^)­BiCl_2_] **(1)** and [(L^2^)­BiCl_2_] **(2)**, respectively. The lower *k*_2_ values relative to *k*_1_ indicate that the second protonation step is likely the rate-determining step of the catalytic HER cycle (Figure S191a and Figure S191b, SI). The significantly higher rate constants (*k*_1_ and *k*_2_) observed for [(L^1^)­BiCl_2_] **(1)** relative to [(L^2^)­BiCl_2_] **(2)** highlight the key role of the pendant amine in the secondary coordination sphere of [(L^1^)­BiCl_2_] **(1)** in modulating catalytic efficiency. This pendant −*NH* site likely assists the reactivity, facilitating rapid proton transfer from solution to the Bi center and thereby promoting efficient H–H bond formation during the HER process. These findings emphasize the importance of secondary-sphere engineering in enhancing catalytic activity in main-group electrocatalysts, a design strategy that remained largely underexplored. Further mechanistic insight was obtained from kinetic isotope effect (KIE) measurements. Reactions performed with CF_3_COOH and CF_3_COOD afforded *k*_H_/*k*_D_ values of 2.1 for catalyst [(L^1^)­Bi^III^Cl_2_] **(1)** and 1.5 for [(L^2^)­BiCl_2_] **(2)** (Figure S186 and Figure S188, Figure S233, SI). These moderate KIE values differ from the inverse KIE values previously reported by systems studied by Gray and Fukuzumi, as well as the much larger KIE values reported by Grapperhaus for certain transition metal HER catalysts.^110–113^ Instead, the observed KIE values are consistent with the moderate isotope effects reported for cobaloxime and related systems by Sun and coworkers,^114^ as well as Co-based *N*-heterocyclic carbene-supported catalysts described by Mandal and coworkers.^33^ Collectively, these experimental observations, along with computational calculations for the O–H vs O–D pathway (Figure S233, SI), support the involvement of proton transfer from the external acid source to a putative Bi–H intermediate, in the rate-determining step of the catalytic cycle for hydrogen evolution (vide infra).

To exclude the possibility of heterogeneous catalysis arising from catalyst adsorption on the electrode surface, rinse tests were performed using the same glassy carbon working electrode following catalytic experiments in the presence of the various proton sources employed. The absence of any significant increase in catalytic current after rinsing ruled out the involvement of electrode-adsorbed species in the HER activity (Figures S135–S138, SI). Furthermore, control experiments performed with the different proton sources under identical experimental conditions using polished glassy carbon electrodes but in the absence of the Bi catalysts showed negligible catalytic current (Figures S139–S148, SI). These results confirm that the observed catalytic activity originates from the molecular Bi complexes operating homogeneously in solution and highlight the essential role of the Bi catalysts in promoting efficient HER at significantly reduced overpotentials.

### 3.8 Controlled Potential Electrolysis (CPE) Analysis

Controlled-potential electrolysis (CPE) experiments were conducted to verify and quantify hydrogen generation by the Bi-based electrocatalysts (Figures S209–S228, SI). Bulk electrolysis for the Bi-catalyzed HER was performed in a four-neck glass cell containing 0.2 M pentafluorophenol (p*K*_a_ = 20.10 in acetonitrile) using a glassy carbon working electrode (surface area = 0.071 cm^2^) in the presence of 1.0 mM catalyst ([(L^1^)­BiCl_2_] **(1)**, [(L^2^)­BiCl_2_] **(2)**, [(L^3^)­BiCl_2_] **(3)**, [(L^4^)­BiCl_2_] **(4)**) and 0.1 M tetrabutylammonium hexafluorophosphate as the supporting electrolyte in 35 mL of acetonitrile. The applied potentials were selected based on the catalytic wave observed in cyclic voltammetry (see Table S33, SI). The total charge passed during electrolysis corresponded to Faradaic efficiencies of ∼98–100% across the catalyst series ([(L^1^)­BiCl_2_] **(1)**, [(L^2^)­BiCl_2_] **(2)**, [(L^3^)­BiCl_2_] **(3)**, [(L^4^)­BiCl_2_] **(4)**). The evolved hydrogen was detected and quantified by gas chromatography with a thermal conductivity detector via headspace injection using a standard calibration curve (Figure S211, Figure S214, Figure S217, Figure S220, Figure S223 and Figure S226, SI). The structural integrity of the catalysts remained unchanged after 2 h of bulk electrolysis in the presence of the proton source, as confirmed by ultraviolet–visible (UV–vis) spectroscopy (Figure S212, Figure S215, Figure S218, Figure S221, Figure S224 and Figure S228, SI) as well as ^1^H NMR spectroscopy in CDCl_3_ (Figure S227). Collectively, the Bi-based catalysts exhibited excellent stability and high Faradaic efficiencies, underscoring their robustness and potential relevance for energy-related transformations. Rinse experiments performed with the same glassy carbon electrode following CPE displayed negligible current response and no detectable hydrogen evolution, ruling out electrode-adsorbed species as the active catalyst (Figure S210, Figure S213, Figure S216, Figure S219, Figure S222 and Figure S225, SI). Control experiments conducted in the absence of catalyst with the proton sources produced no measurable hydrogen, further underscoring the essential role of the designed Bi homogeneous catalysts in promoting efficient HER (Figure S210, Figure S213, Figure S216, Figure S219, Figure S222 and Figure S225, SI).

### 3.9 Mechanistic Investigation

Our experimental results indicate that in situ electrochemical access to low-valent Bi­(I) species plays a key role in initiating the proton reduction activity. To obtain direct experimental evidence for the participation of Bi­(I) intermediates in the electrocatalytic cycle, UV–vis spectroelectrochemical (UV–vis SEC) experiments were performed to correlate changes in absorption spectral features under electrochemical conditions (Figure 11 and Figures S201–S208, SI). Based on previous literature precedence and DFT calculations,^62,115^ as well as our experimental results, it is evident that while Bi­(III) [(L^1^)­BiCl_2_] **(1)** or [(L^2^)­BiCl_2_] **(2)** supported by the NCN pincer backbone used in this study have optical absorption features primarily in the ultraviolet region (Figures S48–S49, SI), the Bi­(I)-based intermediates [(L^1^)­Bi^I^] **(1′)** or [(L^2^)­Bi^I^] **(2′)** show prominent absorption features in the visible range assigned to Bi­(I) → (N,C,N) metal-to-ligand charge transfer (MLCT) transitions (*λ*_max_ = 600 nm for **(1′)** and *λ*_max_ = 630 nm for **(2′)**) which account for the characteristic dark green color observed in acetonitrile solutions (Figure S205, SI). Such spectral features have previously been associated with low-valent Bi­(I) pincer complexes.^62,115^ An analogous redox-inactive Al­(III) complex [(L^2^)­AlCl_2_] **(8)** when subjected to similar spectroelectrochemical conditions (Figure S45 and Figure S47, SI) ruled out the formation of any ligand radical anion species to be responsible for the 600 to 630 nm chromophore assigned to Bi­(I) → (N,C,N) MLCT transitions. To probe the in situ-formed Bi­(I)-active species under electrochemical conditions, UV–vis spectroelectrochemical experiments were carried out using a SEC-CT thin-layer quartz spectroelectrochemical cell equipped with a platinum grid mesh working electrode, a platinum wire counter electrode, and an Ag/AgCl (Vycor frit) pseudoreference electrode connected to a potentiostat operating in amperometric mode and coupled to a UV–vis spectrophotometer (Section XV, SI). A potential of −1.25 V vs Ag/AgCl was then applied to access the Bi^III^/Bi^I^ redox couple for complexes [(L^1^)­BiCl_2_] **(1)** and [(L^2^)­BiCl_2_] **(2)** (Figure 11 and Figures S201–S208, SI). For [(L^1^)­BiCl_2_] **(1)**, the initial colorless solution with an absorption band at 295 nm turned dark green along with the formation of new absorption features at 388 nm and 488 nm along with the prominent 600 nm band (Figure S201, SI). This band is attributed to a Bi­(I) → L^1^ MLCT transition, consistent with the formation of the electrochemically reduced species [(L^1^)­Bi^I^] **(1′)**. Notably, the reduced species [(L^1^)­Bi] **(1′)** thus formed was highly reactive and a rapid decay of the 600 nm absorption band was observed, indicating the transient nature of the Bi­(I) intermediate under catalytic conditions (Figure S202, SI). A similar spectroelectrochemical response was observed for [(L^2^)­BiCl_2_] **(2)**, which initially exhibits a UV–vis absorption maximum at 285 nm (Figure S48). Upon applying −1.25 V vs Ag/AgCl, new absorption bands appeared at 403, 476, and 630 nm, corresponding to the formation of the reduced species [(L^2^)­Bi] **(2′)** (Figure 11a). The time-resolved evolution and decay of species [(L^2^)­Bi] **(2′)** are shown in Figure 11a and 11b. Collectively, these UV–vis spectroelectrochemical studies provide direct experimental evidence for the in situ generation of Bi­(I) intermediates under electrochemical conditions, supporting their involvement as key reactive species in the proton reduction catalytic cycle. Further attempts to detect any putative Bi–H species thus formed under spectroelectrochemical conditions revealed a UV–vis peak at around 430 nm (Figure S203, SI), tentatively attributable to a Bi–H intermediate based on previous literature reports.^62^ The intensity of this feature gradually decays under the applied potential over a period of 1 h (Figure S204, SI). However, detailed study and isolation of such species are currently underway in our group.

**Figure 11:**
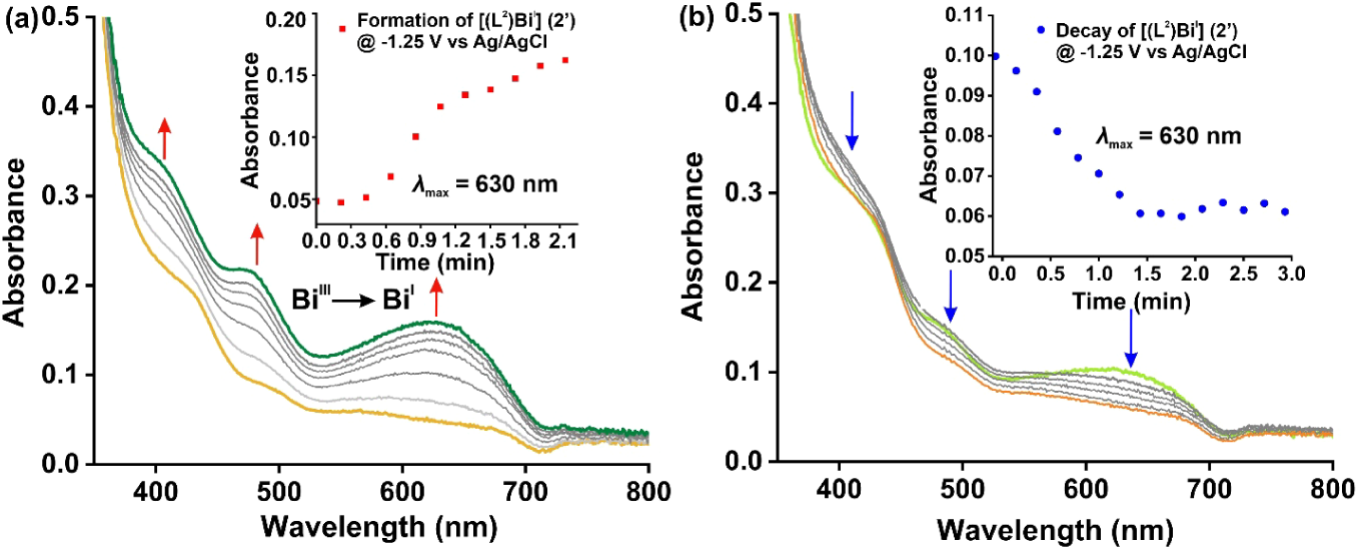
(a) UV–vis absorption spectrum for the formation of bismuthinidene [(L^2^)­Bi^I^] **(2′)** in acetonitrile from the corresponding [(L^2^)­Bi^III^Cl_2_] **(2)** catalyst under electrochemical conditions with an applied potential of −1.25 V vs Ag/AgCl (inset shows the time profile of formation of the peak at 630 nm). (b) UV–vis absorption spectrum for the decay of bismuthinidene [(L^2^)­Bi^I^] **(2′)** in acetonitrile (inset shows the time profile of decay of the peak at 630 nm).

### 3.10 Computational Calculations and Insight into Electronic Structure

The interesting spectroscopic features observed for the reduced bismuthidine species ([(L^1^)­Bi] **(1′)** or [(L^2^)­Bi] **(2′)**) prompted us to further examine their electronic structures to understand the unique Bi–NCN pincer bonding interactions and their potential role in catalysis. Quasi-restricted orbital (QRO) analysis was employed to examine the frontier molecular orbitals of the Bi­(III) precatalysts [(L^1^)­BiCl_2_] **(1)** and [(L^2^)­BiCl_2_] **(2)** and the active Bi­(I) catalysts [(L^1^)­Bi] **(1′)** and [(L^2^)­Bi] **(2′)** to understand their electronic structure and possible reactive intermediates (for more details, see Supporting Information; Section XVII, Figures S230–S231). QRO analysis of Bi­(III) precatalysts [(L^1^)­BiCl_2_] **(1)** and [(L^2^)­BiCl_2_] **(2)** revealed that the HOMO is primarily localized on the chloride ligands, with only a minor contribution from the central Bi­(III) center (Figure S230–S231, SI), whereas the LUMO is predominantly localized on the NCN pincer ligand frameworks (**L** ^**1**^ and **L** ^**2**^) (Figures S230–S231, SI). However, the active Bi­(I) catalysts ([(L^1^)­Bi^I^] **(1′)** and [(L^2^)­Bi^I^] **(2′)**) revealed that the frontier occupied orbital predominantly resides on the *p*-type lone pair of the Bi­(I) center, consistent with the presence of a nucleophilic bismuth center, thereby providing an open axial site for proton coordination, as illustrated in Figure S230, SI. Similar electronic characteristics have been reported by Luo and coworkers for related low-valent bismuth systems generated in situ.

Interestingly, though the nature of HOMO in [(L^1^)­Bi^I^] **(1′)** is comparable to that of [(L^2^)­Bi^I^] **(2′)**, the LUMO of the bismuthidine [(L^1^)­Bi^I^] **(1′)** exhibits partial localization on the Bi center, unlike [(L^2^)­Bi^I^] **(2′)** which is mainly ligand-based, indicating enhanced participation of Bi­(I) in electron-accepting processes in [L^1^Bi^I^] **(1′)**. Such metal–ligand orbital mixing often leads to facilitated redox processes and improved catalytic activity consistent with the experimentally observed superior HER performance for catalyst [(L^1^)­Bi] **(1′)**. In addition, the HOMO of [(L^1^)­Bi] **(1′)** lies at higher energy relative to that of [(L^2^)­Bi] **(2′)**, indicating a more electron-rich Bi center in [(L^1^)­Bi] **(1′)**. Calculations also reveal a higher LUMO energy for [(L^1^)­Bi] **(1′)** compared to [(L^2^)­Bi] **(2′)**, suggesting that subtle electronic effects arising from the pendant amine functionality in the ligand framework modulate the frontier orbital energetics and thereby influence catalytic reactivity. These electronic features are expected to facilitate proton-coupled electron transfer steps and promote efficient formation of reactive Bi–H intermediates during the hydrogen evolution reaction. The UV–vis absorption band corresponding to the Bi­(I) to NCN pincer MLCT transition for the bismuthidine derived from catalyst [(L^1^)­BiCl_2_] **(1)**, i.e., [(L^1^)­Bi^I^] **(1′)**, appears blue-shifted (*λ*_max_ = 600 nm) relative to the MLCT transition observed for the corresponding bismuthidine derived from catalyst [(L^2^)­BiCl_2_] **(2)**, i.e., [(L^2^)­Bi^I^] **(2′)** (Figure S205). This spectral shift suggests a higher HOMO–LUMO gap in [(L^1^)­Bi^I^] **(1′)** as compared to [(L^2^)­Bi^I^] **(2′)** (Table S34, SI). This trend is further supported by Löwdin charge analysis of the corresponding Bi­(III) precatalysts [(L^1^)­BiCl_2_] **(1)** and [(L^2^)­BiCl_2_] **(2)** and their reduced bismuthidines [(L^1^)­Bi^I^] **(1′)** and [(L^2^)­Bi^I^] **(2′)** (Table S37, SI). While the calculated Löwdin charge on the Bi centers in the Bi­(III) catalysts [(L^1^)­BiCl_2_] **(1)** and [(L^2^)­BiCl_2_] **(2)** are nearly identical (Table S37, SI), the charge on the bismuthidine Bi­(I) center in [(L^1^)­Bi^I^] **(1′)** is significantly lower than that in [(L^2^)­Bi^I^] **(2′)**, indicating a more electron-rich Bi­(I) center in [L^1^Bi] **(1′)**. These computational observations are consistent with the experimentally probed electrochemical behavior as well as ^1^H NMR spectroscopic studies, both of which indicate an electron-rich Bi center for catalyst [(L^1^)­BiCl_2_] **(1)** relative to [(L^2^)­BiCl_2_] **(2)**. However, considering the magnitude of the experimentally observed catalytic rate enhancement for catalyst [(L^1^)­BiCl_2_] **(1)** relative to catalyst [(L^2^)­BiCl_2_] **(2)** or *N*-methylated version of catalyst **(1)**, i.e., catalyst [(L^7^)­BiCl_2_] **(7)**, it is unlikely that electronic effects alone account for the improved catalytic performance. Instead, the pendant amine functionality present in catalyst [(L^1^)­BiCl_2_] **(1)** is likely to provide an additional kinetic advantage through a pendant *N–H*-assisted secondary-sphere interaction effect, facilitating more efficient proton transfer during catalysis. To further validate this hypothesis and gain deeper insight into the proton reduction pathway mediated by these main-group redox-active catalysts, plausible reaction pathways and key catalytic intermediates were investigated computationally for catalysts [(L^1^)­BiCl_2_] **(1)** and [(L^2^)­BiCl_2_] **(2)** (also for the comparative pathway of catalyst **(1)** with catalyst **(7)** and also catalysts **(1)**, **(3)**, and **(6)**, see SI Figures S234–S240).

A detailed computational calculation correlated to the experimental observations presented a holistic understanding of the intriguing reaction pathway (Figures 12 and 13). DFT calculations were conducted by using the ORCA 5.0.3 quantum chemical software package^71,72^ (for details, see the Computational Methods section and also Section XVII, SI).

**Figure 12:**
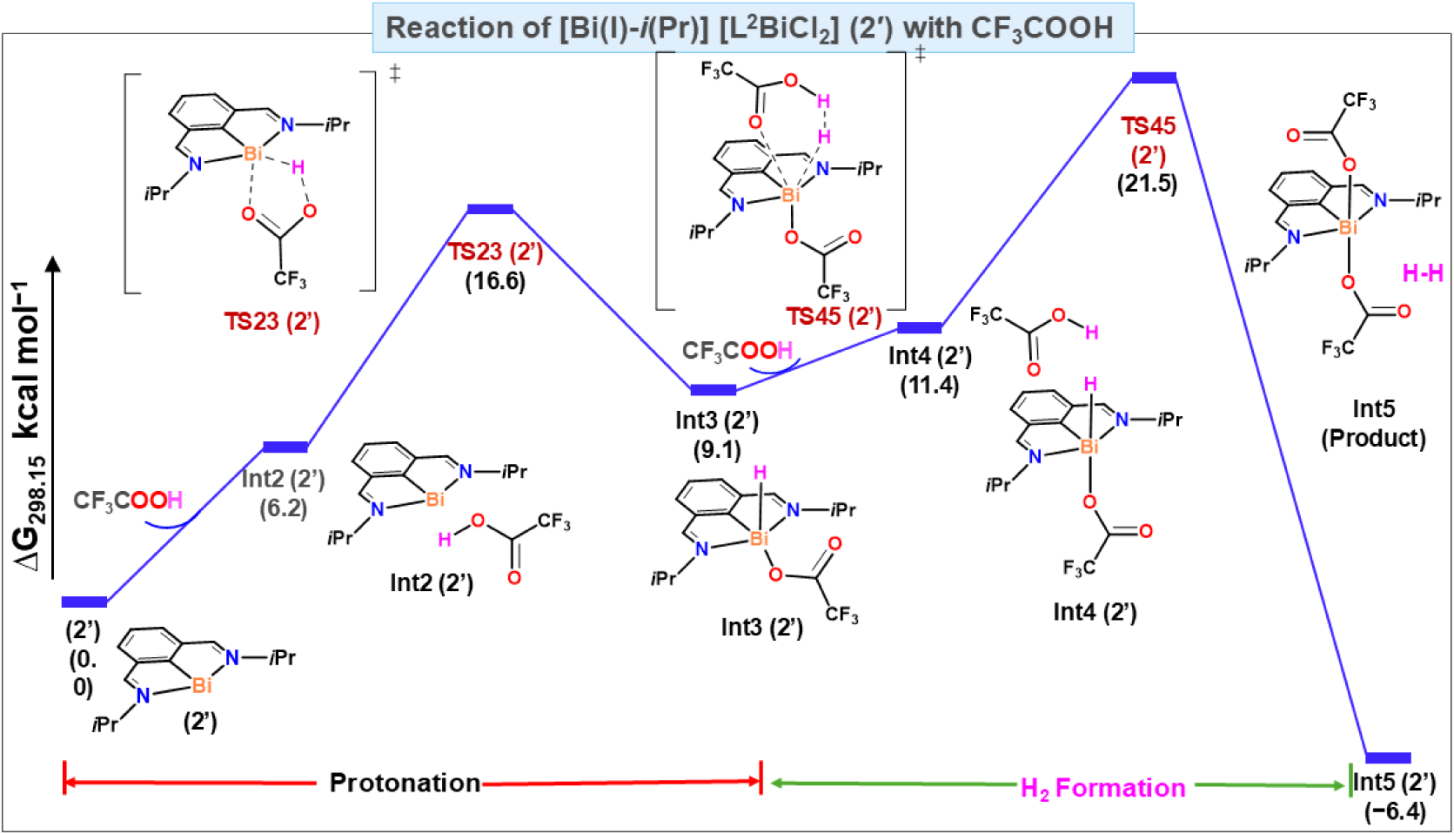
Computed Gibbs free energy profile for the hydrogen evolution reaction catalyzed by Bi­(I)–*i*Pr [**L^2^Bi^I^**] **(2′)** in the presence of CF_3_COOH, illustrating the relative stability of intermediates and transition states.

**Figure 13:**
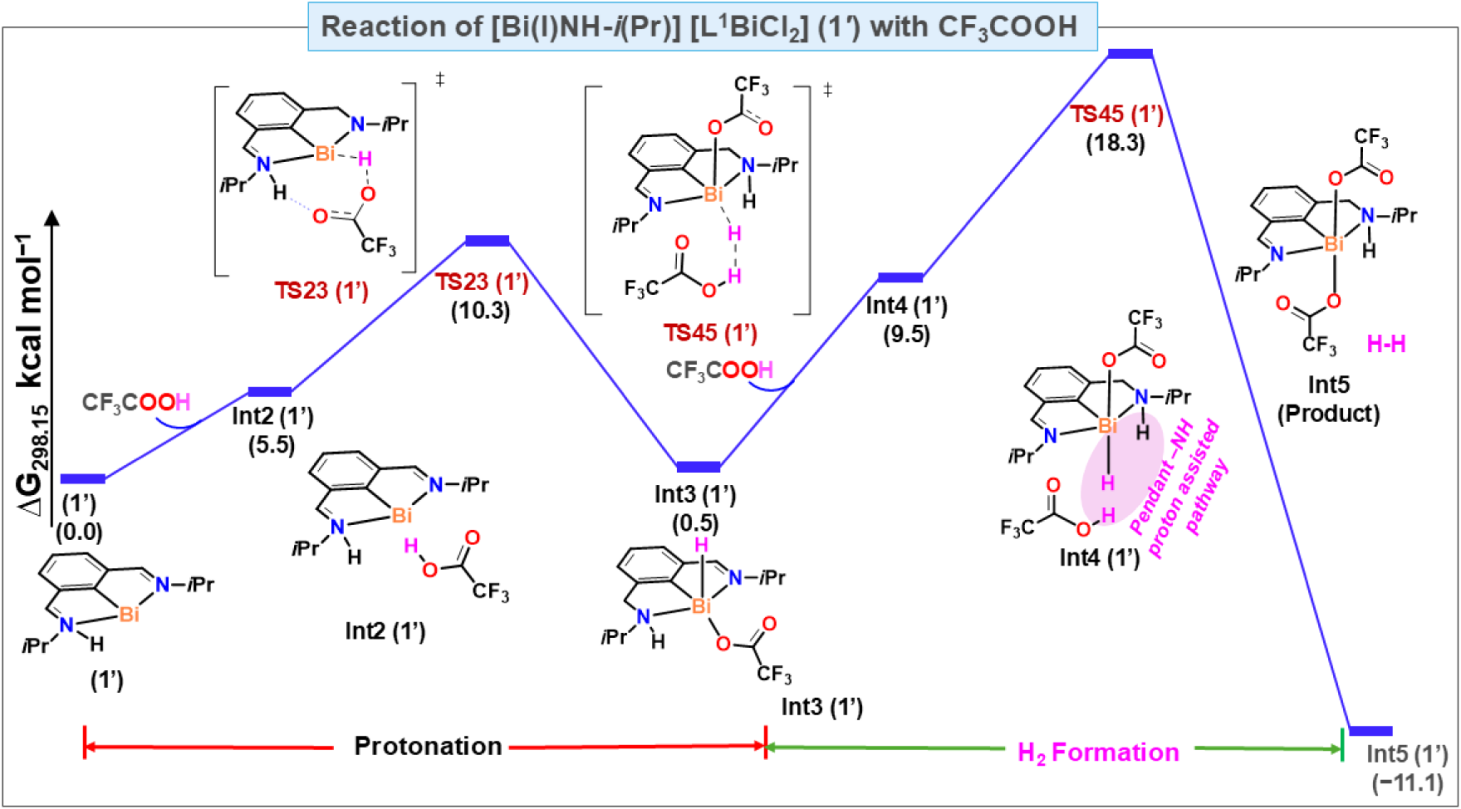
Computed Gibbs free energy profile for the hydrogen evolution reaction catalyzed by Bi­(I)–NH-*i*Pr [**L^1^Bi^I^**] **(1′)** in the presence of CF_3_COOH illustrating the relative stability of intermediates and transition states.

For computational calculations, TFA (CF_3_COOH) (p*K*_a_ = 12.65 in acetonitrile)^90^ was used as the proton source. The catalytic cycle begins with an electrochemical reduction step, wherein the Bi­(III) precatalyst [L^2^BiCl_2_] **(2)** (Figure 12) is reduced to generate the catalytically active Bi­(I)–*i*Pr species [(L^2^)­Bi^I^] **(2′).** Subsequently, the O–H bond of CF_3_COOH undergoes heterolytic cleavage through a proton transfer process via transition state **TS23­(2′)** with an activation barrier of Δ*G* ^‡^ = 16.6 kcal mol^–1^, leading to the formation of a bismuth­(III) hydride intermediate **(Bi** ^**III**^ **–H; Int3(2′))**, as illustrated in Figure 12. Thus, the overall transformation from **[L** ^**2**^ **Bi** ^**I**^ **] (2′)** to **Int3­(2′)** can be considered as a proton-coupled electron transfer step that generates the key Bi­(III)–H intermediate **Int3­(2′)**. The proton transfer is further supported by Intrinsic Bonding Orbital (IBO) analysis (Figure S231, SI). In the subsequent step, hydrogen evolution proceeds via the reaction of the hydride from the Bi^III^–H intermediate **Int3­(2′)** with a proton derived from another CF_3_COOH molecule. This step occurs through a heterolytic H–H bond formation pathway involving intermediate **Int4­(2′)** via transition state **TS45­(2′)** with an activation barrier of 21.5 kcal mol^–1^. This process results in the formation of molecular hydrogen (H_2_) along with Bi­(III)–trifluoroacetate species **(Int5­(2′))** as the product (Figure 12).

An analogous computational investigation was carried out for the modified catalyst [(L^1^)­Bi^I^] **(1′)** bearing a pendant amine–imine arm to evaluate the influence of ligand variation on the reaction energetics (Figure 13). Although the overall mechanistic pathway remains similar for both catalysts, notable differences in the activation barriers are observed. As depicted in Figure 13 (also Figure S234) the first protonation step for catalyst [(L^1^)­Bi^I^] **(1′)** proceeds via **TS23­(1′)** with an activation barrier of Δ*G* ^‡^ = 10.3 kcal mol^–1^, which is 6.3 kcal mol^–1^ lower than that calculated for the corresponding step in catalyst [(L^2^)­Bi^I^] **(2′)**, leading to the formation of the bismuth­(III) hydride intermediate (Bi^III^–H; **Int3­(1′)**). Subsequently, protonation of the Bi^III^–H species results in H_2_ evolution through **TS45­(1′)** with an activation free energy of Δ*G* ^‡^ = 18.3 kcal mol^–1^, ultimately affording the product **Int5­(1′)**. This barrier is lower than that computed for the analogous step in catalyst [L^2^Bi^I^] **(2′)** (Δ*G* ^‡^ = 21.5 kcal mol^–1^), indicating a more favorable hydrogen evolution step in the presence of one of the pendant amine functionalities in the ligand backbone of catalyst [L^1^Bi^I^] **(1′)**. The catalytic cycle is completed by an electrochemical step that regenerates the active Bi­(I) species. Consistent with the experimental observations, the computational analysis identifies the second protonation/H–H bond formation step as the rate-determining step for HER catalyzed by these NCN pincer-based Bi homogeneous electrocatalysts (Figure S233, SI). Interestingly, examination of the transition state structures from **(1′)** reveals that **TS23­(1′)** is stabilized by a hydrogen-bonding interaction involving the pendant amine proton of the reduced amine–imine pincer ligand (**L** ^**1**^) (Figure 14a). Furthermore, the transition state responsible for hydrogen evolution, **TS45­(1′)**, also benefits from the presence of a properly designed pendant amine group in the secondary coordination sphere (Figure 14b and Figure S241, NCI analyses) that likely assists and lowers the activation barriers for promoting extremely fast H–H bond formation and accelerates proton transfer during catalysis. In addition, the role of pendant −*NH* proton assistance was further established through experimental results (Figure 10) as well as computational calculations for the hydrogen-forming step of *N*-methylated analogue [(L^7^)­BiCl_2_] **(7)** (Figure S239, SI) where the −*NH* functionality was blocked while maintaining the electronics. Apparently, though a slightly lower energy barrier (∼2 kcal/mol) for **TS45­(7)** was obtained for [(L^7^)­BiCl_2_] **(7)**, when compared to **TS45­(1)** derived from [(L^1^)­BiCl_2_] **(1)** (Figure S239, SI), the overall product from [(L^7^)­BiCl_2_] **(7)** was at higher energy making the hydrogen release process thermodynamically less efficient from [(L^7^)­BiCl_2_] **(7)** as compared to catalyst [(L^1^)­BiCl_2_] **(1)**. DFT predicts an elongated bond length of the Bi–N amine bond in [(L^7^)­BiCl_2_] **(7)** that destabilizes and makes the transition state **TS45­(7)** unstable (Figure S239, SI). To provide additional mechanistic insight into the role of the pendant *N–H* functionality, the geometries of the rate-determining transition states, **TS45­(1)** and **TS45­(7)**, were examined along the imaginary vibrational mode associated with H_2_ formation. In **TS45­(1)**, the N–H hydrogen atom is positioned in close proximity to the hydrogen atom bound to bismuth. In contrast, this structural feature was absent in **TS45­(7)**, where the *N–H* group is replaced by an *N–CH_3_* substituent. Consistent with this observation, noncovalent interaction (NCI) analyses of **TS45­(1)** revealed an attractive interaction (shown in green in Figure S241) between the N–H and Bi–H hydrogen atoms, whereas no analogous interaction was present in **TS45­(7)**. These results further suggest that **TS45­(1)** benefits from an additional secondary-sphere stabilizing interaction that is absent in **TS45­(7)** which contributes to the experimentally observed enhancement in the catalytic performance of catalyst [(L^1^)­BiCl_2_] **(1)** and provides further support for the beneficial role of the pendant *N–H* functionality.

**Figure 14:**
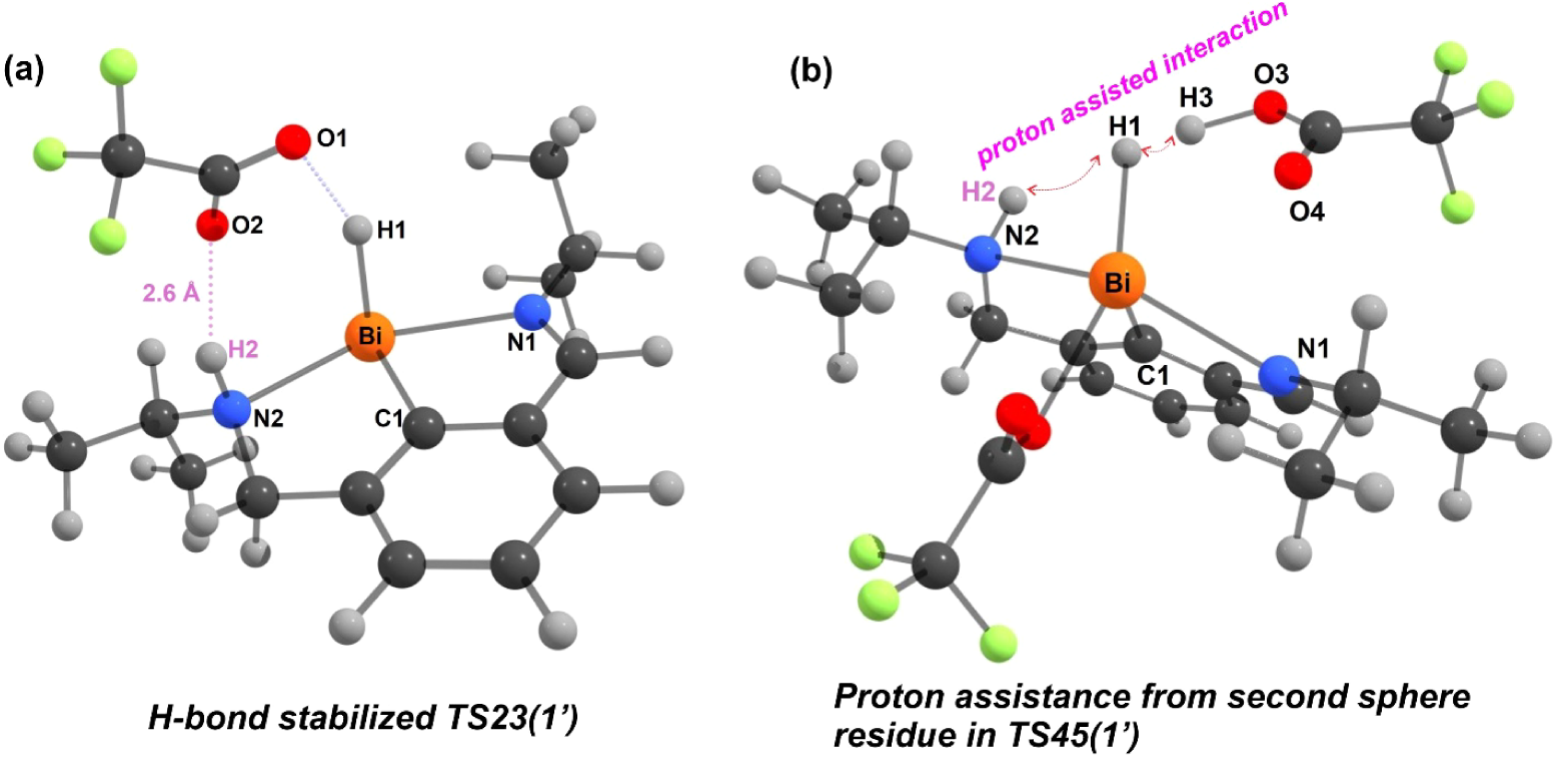
(a) Computed H-bond-stabilized transition state **TS23­(1′)** obtained from the first protonation step by catalyst [(L^1^)­Bi^I^] **(1′)** in the HER pathway. (b) Pendant *N–H*-assisted secondary-sphere interaction from the ligand backbone of [(L^1^)­Bi^I^] **(1′)** as evident in **TS45­(1′)** for hydrogen evolution step.

### 3.11 Proposed Catalytic Mechanism for Hydrogen Evolution

Based on the combined electrochemical, spectroscopic, kinetic, and computational investigations, a plausible catalytic mechanism for hydrogen evolution mediated by the NCN pincer-supported bismuth-based homogeneous electrocatalyst is proposed (Scheme 2). The catalytic cycle is initiated by an electrochemical reduction step, wherein the Bi­(III) precatalysts [(L^1^)­BiCl_2_] **(1)** or [(L^2^)­BiCl_2_] **(2)** undergo two-electron reduction to generate the catalytically active Bi­(I) species ([(L^1^)­Bi^I^] **(1′)** or [(L^2^)­Bi^I^] **(2′)**). Spectroelectrochemical UV–vis studies confirm the in situ formation of these putative low-valent species as monitored through the appearance of characteristic metal-to-ligand charge transfer bands in the visible region (Figure 11). The resulting Bi­(I) intermediate then interacts with the external proton source in acetonitrile (H–X e.g., H–X = H–OOCF_3_), leading to a PCET step that forms a bismuth­(III) hydride intermediate (Bi^III^–H) [**IntH­(1′)**] (Scheme 2). Density functional theory calculations indicate that this step proceeds through heterolytic cleavage of the O–H bond of the acid (H–X) via a transition state associated with moderate activation barriers. The hydride intermediate thus generated represents a key catalytic species in the hydrogen evolution pathway. Notably, Bi–H intermediates are extremely rare and difficult to access under conventional chemical conditions, and to date, only a single well-defined Bi–H complex has been isolated, stabilized by extremely bulky ligands.^116^ In contrast, the strategy presented in this work enables electrochemical access to these otherwise elusive Bi–H intermediates under mild conditions, thereby providing a practical alternative pathway to generate such reactive species in situ (Figure 11 and Figures S201–S208, SI). This approach opens new opportunities to explore the reactivity of low-valent bismuth hydride intermediates, potentially enabling a broader range of transformations and expanding the scope of main-group redox catalysis. Subsequently, the Bi^III^–H intermediate [**IntH­(1′)**] undergoes a second protonation step [**IntH-2­(1′)**], in which a proton from another acid molecule (H–X) reacts with the hydride ligand to generate molecular hydrogen (H_2_) through heterolytic H–H bond formation (Scheme 2). Computational analysis identifies this step as the rate-determining step of the catalytic cycle (Figures 12 and 13), consistent with the experimentally observed kinetic results (*k*_2_ < *k*_1_). This step simultaneously forms a Bi­(III) intermediate, [**IntX-2­(1′)**] which is subsequently reduced electrochemically to regenerate the active Bi­(I) species **(1′)**, thereby completing the catalytic cycle. Importantly, for catalysts containing a pendant amine group in the secondary coordination sphere (e.g., catalyst [(L^1^)­Bi^I^] **(1′)**), the amine functionality participates in hydrogen-bonding interactions with the proton donor, stabilizing key transition states and facilitating proton transfer to the metal center (Figure 14). This pendant N–H-assisted secondary-sphere interaction lowers the activation barriers for both the hydride formation and H–H bond formation steps, resulting in significantly enhanced catalytic rates relative to analogous catalysts lacking this functionality. Collectively, these findings demonstrate how secondary-sphere residue engineering can significantly enhance catalytic efficiency in main-group electrocatalysts and establish a mechanistic framework for the rational design of next-generation redox-active bismuth catalysts for efficient hydrogen evolution reactions.

**Scheme 2:**
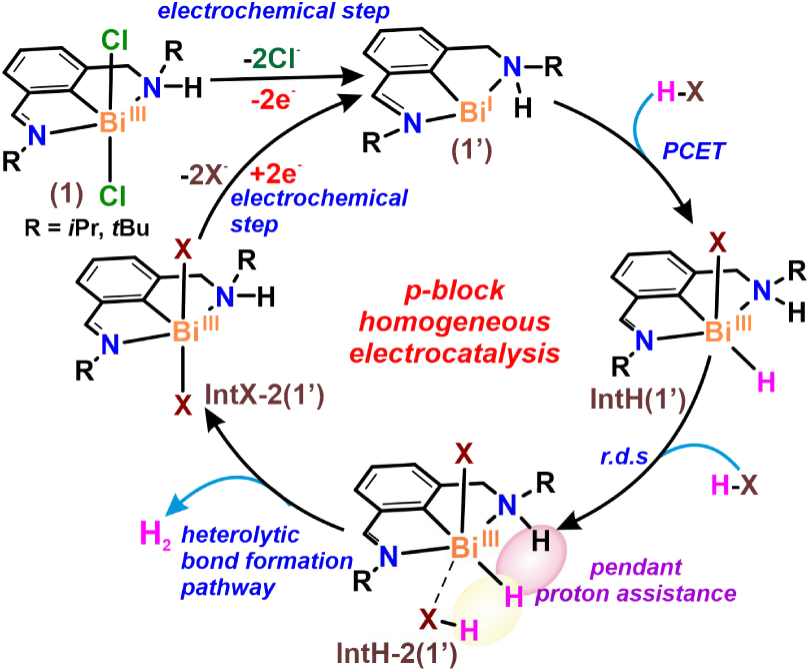
Proposed Mechanism for Bi-Catalyzed Homogeneous Electrochemical HER Harnessing Redox Accessible Bi^I^/Bi^III^ Catalytic Cycle

## 4 Conclusion

In summary, we establish NCN pincer-supported bismuth complexes as a new class of homogeneous main-group electrocatalysts for the hydrogen evolution reaction, addressing key challenges in accessing reactive low-valent intermediates and overcoming conventional activity–overpotential scaling relationships. Electrochemical, kinetic, and spectroelectrochemical studies reveal the in situ generation of catalytically active Bi­(I) species, while foot-of-the-wave analysis (FOWA), potential–p*K*_a_ study, Tafel analysis, and kinetic isotope effects identify a proton-involved rate-determining step. Complementary density functional theory calculations support a Bi­(I)/Bi­(III) catalytic cycle involving a transient Bi–H intermediate and proton-coupled electron transfer pathways that are inaccessible under conventional conditions. Systematic tuning of ligand electronics, geometry, and secondary coordination sphere features highlights their decisive role in modulating catalytic reactivity. In particular, incorporation of a pendant amine functionality operates as an intramolecular proton-assistance, lowering activation barriers by stabilizing key intermediates and transition states through hydrogen-bonding and local proton-environment modulation and enhancing catalytic rates. As a result, the catalyst ([(L^1^)­BiCl_2_] **(1)**) deviates from traditional activity–overpotential scaling relationships, delivering high HER performance at low overpotential and rivaling state-of-the-art transition metal systems.

This work represents the first comprehensive electrokinetic and structure–function–activity study of redox-active bismuth complexes for homogeneous proton reduction and the first application of FOWA to a main-group electrocatalyst. More broadly, it establishes a general design paradigm for main-group redox electrocatalysis, demonstrating how secondary-sphere proton assistance and electrochemical activation can unlock previously inaccessible *p*-block reactivity and enable efficient small-molecule transformations.

## Supplementary Material



## Data Availability

The data supporting the findings of this study are available within the article and its Supporting Information.

## Notes

The experimental details, analytical and spectroscopic data, crystallographic data, and computational calculations (PDF)

## Notes

This work is dedicated to pay tribute to Acharya Prafulla Chandra Ray, the Father of Indian Chemistry.
