# Supplementary material for: Unveiling the Potential of p‑Block Bismuth in Homogeneous Electrocatalytic Proton Reduction: Secondary Coordination Sphere Control of Catalytic Activity
Source: J Am Chem Soc. 2026 Jul 10;148(28):29997–30018. doi: 10.1021/jacs.6c06029 (PMC13397559; doi:10.1021/jacs.6c06029)
Supplement: Supplementary file 1 [file ja6c06029_si_001.pdf]

Unveiling the Potential of *p*-Block Bismuth in Homogeneous Electrocatalytic Proton Reduction: Secondary Coordination Sphere Control of Catalytic Activity

Yashdeep Maurya [a], Parul Bishnoi[a], Rishabh Sharma[a], Vineet Jhamb[a], Akhilesh Sharma[a], Swetha Vasanthdamodar Sivapreetha [a], Arya Singh[a], Puneet Gupta[a], and Sayanti Chatterjee*[a,b]

[a] Dr. S. Chatterjee*, Y. Maurya, P. Bishnoi, R. Sharma, V. Jhamb, A. Sharma, V.S. Swetha , A. Singh, P. Gupta Department of Chemistry, Indian Institute of Technology Roorkee, Roorkee, Uttarakhand, PIN-247667, India

[b] Dr. S. Chatterjee, Max Planck Institute for Chemical Energy Conversion, Stiftstrasse 34-36, 45470 Mülheim an der Ruhr, Germany.

*E-mail: sayanti.chatterjee@cy.iitr.ac.in
sayanti.chatterjee@cec.mpg.de

Supporting Information

Table of Content

1) Abbreviations……………………………………………………………………………….3

2) Experimental Section: Materials and Methods………………………………………..6

3) Synthesis and Characterization of Ligands and Complexes………………………….....8

5) UV-Vis Spectroscopy of Catalysts …………………………………………………..56

6) Single Crystal XRD of Catalysts ……………………………………………………57

7) Synthesis and characterization of Protic acid …………………………...............65

8) Electrochemical Studies of Catalyst………………………………………….........83

9) Diffusion Coefficient of Catalyst……………………………………………………..89

10) Variation in pK_a of proton sources with Catalysts ……………………………….90

11) Order of Catalyst…………..……………………………………………...................117

12) Rinse tests and control study………………………………………….....................121

13) Comparison study of complexes………………………………………………………..129

a) Plot of TOF vs pK_a ……………………………………………………………...138

b) Plot of $E_{cat/2}$ vs pK_a ……………………………………………………………..…140

c) Scan rate independent data ………………………………………………… ….141

d) Linear Free Energy Relationship (LFER) …………………..…………………….…142

14) Foot-Of-The Wave Analysis (FOWA)………………………………………….........144

a) Plot of TOF vs pK_a ……………………………………………………………...155

b) Variable scan rate FOWA analysis…………..……………………………………157

15) Electrochemical experiment for Kinetics and Kinetic Isotope Effect…………………..…162

16) Tafel Analysis………..……………………………………………………….……...170

17) UV-Vis Spectroelectrochemical Studies…………..………………….……………176

18) CPE Experiment……………………..…………………………………………….…182

19) Computational Study…………………………………………………..………………..195

Abbreviations

Technical terms

equiv.	equivalent
r.t.	room temperature
J	coupling constant (cm^{-1})
λ	*wavelength*

Techniques

IR	infrared spectroscopy
NMR	nuclear magnetic spectroscopy
UV- Vis	ultraviolet- visible spectroscopy

Units

cm	centimetre
h	hour
°C	degree Celsius
M	molar
mM	millimolar
*μ*L	microlitre
min	minute
m	metre
dec	Decade

Latin expressions

et al.	and co-workers
e.g.	for example,
i.e.	namely
tert	tertiary
ipr	isopropyl

Solvents and reagents

CH_2Cl_2/ DCM	dichloromethane
$CHCl_3$	chloroform
Et_2O	diethyl ether
EtOAc	ethylacetate
MeCN	acetonitrile
DMSO	dimethyl sulfoxide
DMF	Dimethylformamide
THF	tetrahydrofuran
MeOH	methanol
NEt_3	triethylamine
*t*Bu	*tert*-Butyl
*i*Pr	*iso*-Propyl
HCl	hydrogen chloride
HBr	hydrogen bromide
H_2SO_4	sulfuric acid
NaCl	sodium chloride
Na_2SO_4	sodium sulfate
$NaHCO_3$	sodium bicarbonate
$BiCl_3$	Bismuth trichloride
$NaBH_4$	Sodium Borohydride
KOH	Potassium hydroxide
MS	Molecular sieves
n-BuLi	Butyl lithium
$TBAPF_6$	Tetrabutylammonium Hexafluorophosphate

Electrochemical terms

i_{cat}	Maximum current observed at plateau shape
i_p	Catalyst current at potential where i_{cat} observed
i_{pc}	Cathodic peak current of catalyst
i_{pa}	Anodic peak current of catalyst
υ	Scan rate (V/s)
n	Number of electrons transferred in the redox event
A	Electrode surface area (cm^2)

D_0	Diffusion coefficient
C^0	Bulk analyte concentration
V	Potential
E_{red}	Reduction Potential
$E_{\mathrm{p,c}}$	Cathodic peak potential
$E_{\mathrm{p,a}}$	Anodic peak potential
$E_{\mathrm{cat/2}}$	Potential at half of the maximum catalytic current
j	*Current density*
j_{cat}	*Peak current density*
K	*Rate* constant
η	*Overpotential*
TOF	*Turnover Frequency*
TON	*Turnover Number*
E_{onset}	*Approximate potential at which TOF >0*
k_1	Rate constant for the first chemical step
k_2	Rate constant for the second protonation step

Experimental Details Materials and Methods

All chemicals and reagents were obtained from commercial sources and were used without further purification unless otherwise noted. $BiCl_3$, Phenol, Pentafluorophenol, 4-methoxy aniline, 4-Trifluoromethylphenol, tert-butylamine, were purchased from TCI, *p*-Chloroaniline, Aniline, Pyridine, 2-Aminopyridine, Dimethylformamide, Isopropylamine, purchased from Sisco Research laboratories Pvt. Ltd. (SRL), Trifluoroacetic acid, Trichloroacetic acid were purchased from GLR Innovations, 2-bromoisopthaldehyde was purchased from BLD, Tetrafluoroboric acid-ether complex and *n*-BuLi (specified concentration) were purchased from Merck (*n*-Butyllithium (*n*-BuLi) is highly reactive and pyrophoric, and it may catch fire if exposed to air or moisture. The reagent was handled behind a shield in a chemical fume hood. Safety goggles, chemical-resistant gloves, and other protective clothing were worn). Solvents were purchased from commercial sources and used as it is. For reactions under dry solvent, solvents were distilled and degassed using standard procedure.[1] All chemicals used in the research were purchased from commercial sources and used without further purification unless otherwise mentioned. The catalysts which were synthesized has been included in the experimental portion.

NMR spectroscopy: Nuclear Magnetic Resonance spectra were measured using a Jeol spectrometer (500 MHz). ^{1}H-NMR was reported as follows: chemical shift, multiplicity (s = singlet, d = doublet, t = triplet, q = quadruplet, m = multiplet and br = broad), coupling constant (*J* values) in Hz and integration. Chemical shifts (δ) were reported with respect to the corresponding solvent residual peak at 7.26 ppm for $CDCl_3$, 1.94 for CD_3CN for ^{1}H-NMR. ^{13}C-NMR spectra (^{1}H-broadband decoupled) were reported in ppm using the central peak of $CDCl_3$ (77.16 ppm) and CD_3CN (118.26). Spectra were processed with MestReNova 16.0.0 and coupling constants are reported as observed.

UV-Vis Absorption Spectroscopy [UV-Vis ABS] - The Agilent Cary 60 UV-Vis Spectrophotometer instrument was used to record UV- Visible spectra using a pair of quartz cuvette of pathlength 1 cm at 25 °C.

IR Spectroscopy – The Bruker ALPHA II FT-IR spectrometer was used to measure IR spectra at 25 °C. FT-IR Spectrum reported as follows: frequency, (vs = very strong, s = strong, m = medium, w= weak, br = broad).

Mass Spectrometry: High resolution mass spectrometry (HR-MS) was performed on an Agilent 6545 LC/Q-TOF in ESI mode. The ionization method and mode of detection employed

is indicated for the corresponding experiment and all masses are reported in atomic units per elementary charge (m/z) with an intensity normalized to the most intense peak.

Gas Chromatography with TCD: The gas chromatographic analysis was performed using Agilent GC (8890) Thermal conductivity detector (TCD) and Shimadzu GC (HP7890A with TCD). The following chromatographic conditions were used: Carrier gas: Argon, Column temperature: 60 °C, TCD temperature: 200 °C, and injection temperature: 150 °C with flow rate 20 mL/min and run time 15 min.

Chromatography - Thin Layer Chromatography analyses were performed on silica gel coated glass plates (0.25 mm) with fluorescence-indicator UV254 (Macherey-Nagel, TLC plates SIL G25 UV254). For detection of spots, irradiation of UV light at 254 nm, and oxidative staining.

Single Crystal X-Ray Diffractometer: Single Crystal X-Ray Diffraction Data collection was collected in Bruker-D8 QUEST diffractometer (See crystallographic section for details). The structure was solved and refined by Software package APEX-4 and Olex2-1.5. Crystal structures were drawn by Diamond.

Electrochemistry: All electroanalytical experiments were performed using a Metrohm DropSens *μ*Stat-i 400 potentiostat with glassy carbon working (3 mm), Ag/AgCl as pseudoreference electrodes and the counter electrode was Pt wire.

Synthesis of Ligands(I)

A) Synthesis of Ligand L^1Br

Step 1: Synthesis of 2-bromo-3-(bromomethyl) benzaldehyde (A) (L^1Br)

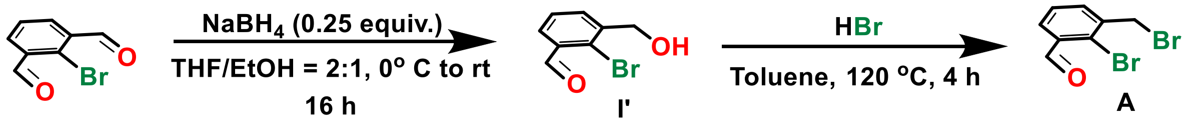

Scheme S1. Synthesis of 2-bromo-3-(bromomethyl) benzaldehyde (A)

A 250 mL round bottomed flask equipped with a magnetic stir bar was charged with 2-bromoisopthaldehyde (2 g, 9.3 mmol, 1 equiv.), 30 mL dry tetrahydrofuran (THF) and 15 mL ethanol (EtOH). The contents were stirred until dissolved. The solution was placed in an ice bath after which sodium borohydride (86.9 mg, 2.3 mmol, 0.25 equiv.) was added at once to the stirring mixture. The reaction mixture was stirred overnight and warmed to room temperature. A pale yellow-brown solid was obtained after filtering and evaporating the filtrate under vacuum. This product (I') (crude) was used for the next step without further purification.

A 250 mL round bottomed flask containing the crude product (I') equipped with a magnetic stir bar, was charged with 30 mL toluene. To the mixture was added an aqueous HBr solution (47%, 2.8 mL). The mixture was heated to reflux for 4 h after which it was cooled to ambient temperature and diluted with 12 mL water. The organic phase was separated, and the aqueous layer was extracted with diethyl ether (3 x 10 mL). The combined organic layer was washed with brine, dried over Na_2SO_4 and concentrated under vacuum. Solid product was obtained after refrigeration. The crude product was purified using column chromatography with 50% dichloromethane-hexane as the eluent to give 0.94 g (36.5% yield over two steps) of 2-bromo-3-(bromomethyl) benzaldehyde as white solid (A).[2]

NMR of 2-bromo-3-(bromomethyl) benzaldehyde

^{1}H NMR (500 MHz, $CDCl_3$): δ 10.44 (s, 1H), 7.86 (d, J = 9.5 Hz, 1H), 7.70 (d, J = 7.6 Hz, 1H), 7.43 (t, J = 7.6 Hz, 1H), 4.67 (s, 2H)

^{13}C NMR (125 MHz, $CDCl_3$): δ 191.92, 138.92, 136.69, 134.85, 130.14, 128.99, 128.21, 32.39

HR-MS (ESI$^+$) (L: $C_8H_6Br_2O$, M.W = 277.8765**)** calculated for [**L**−Br]$^+$196.9602; found 196.9601.

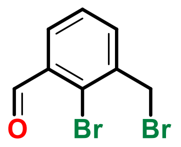

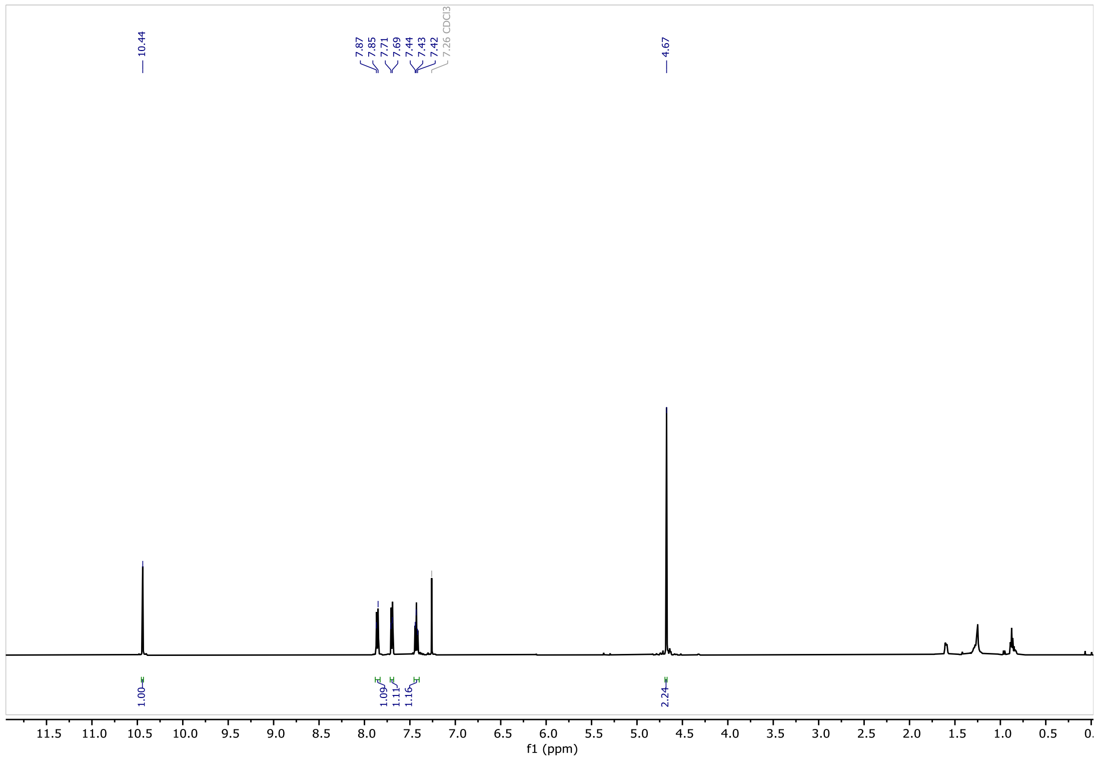

Figure S1. ^{1}H NMR (500 MHz) of **A** in $CDCl_3$ at 298 K.

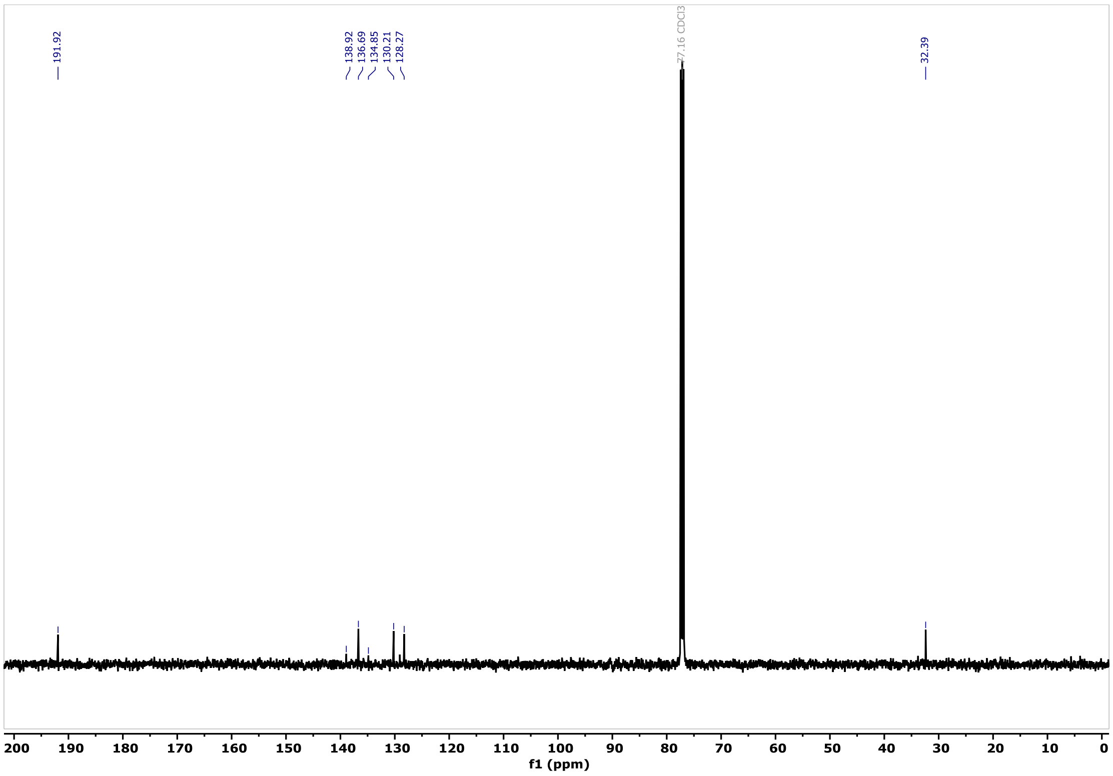

Figure S2. ^{13}C NMR (126 MHz) of **A** in $CDCl_3$ at 298 K.

Step 2: Synthesis of (E)-N-(2-bromo-3-((iso-propylimino)methyl)benzyl)-2-methylpropan-2-amine (L^1)

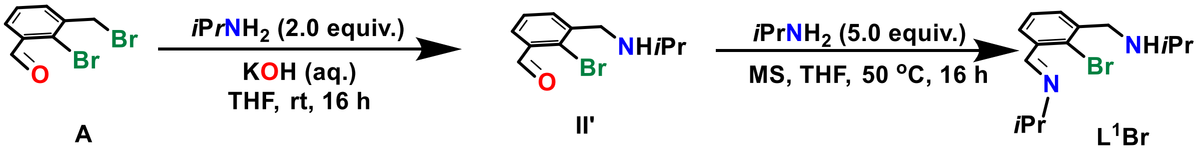

Scheme S2. Synthesis of (E)-N-(2-bromo-3-((iso-propylimino)methyl)benzyl)-2-methylpropan-2-amine (L^1Br)

A 25 mL round-bottomed flask equipped with a magnetic stir bar was charged with **A** (0.94 g, 3.4 mmol, 1.0 equiv.), obtained from the previous step and isopropyl amine (*iPr*NH_2) (0.579 mL, 6.8 mmol, 2.0 equiv.), and tetrahydrofuran (THF) (20 mL). An aqueous KOH solution (10 M, 2 mL) was then added, and the mixture was stirred overnight at ambient temperature. The organic phase was separated, dried over Na_2SO_4, and concentrated to afford a light-yellow solid product. This product (II') was used in the next step without further purification.

A 100 mL two-necked round-bottomed flask with a stir bar was charged with the product (II') obtained in step 2, *iPr*NH_2 (1.4 mL, 17 mmol, 5.0 equiv.), Molecular Sieves (4 Å) and 12 mL

dry tetrahydrofuran (THF). The reaction was stirred for 48 hours at 50 °C and the completion of the reaction was monitored by NMR. The mixture was filtered through celite and eluted with dichloromethane and ethyl acetate. The filtrate was concentrated under reduced pressure to afford a 1.12 g yellow semi-viscous oil L^1 (89% over two steps).[2]

NMR of L^1Br

^{1}H NMR (500 MHz, $CDCl_3$): δ 8.72 (s, 1H), 7.86 (dd, J = 7.8, 1.8 Hz, 1H), 7.42 (dd, J = 7.5, 2.0 Hz, 1H), 7.28 (t, J = 7.5 Hz, 1H), 3.89 (s, 2H), 3.64 – 3.59 (m, 1H), 2.83 (p, J = 6.3 Hz, 1H), 1.26 (d, J = 6.3 Hz, 6H), 1.10 (d, J = 6.3 Hz, 7H).

^{13}C NMR (125 MHz, $CDCl_3$): δ 158.19, 140.15, 136.01, 132.35, 128.17, 127.73, 61.99, 51.96, 48.28, 24.48, 23.25.

HR-MS (ESI$^+$) (L^1: $C_{14}H_{22}BrN_2$, M.W = 297.0888**)** calculated for $[L^1+H]^+$ 297.0966; found 297.0965.

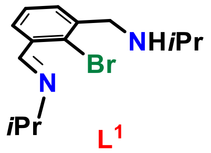

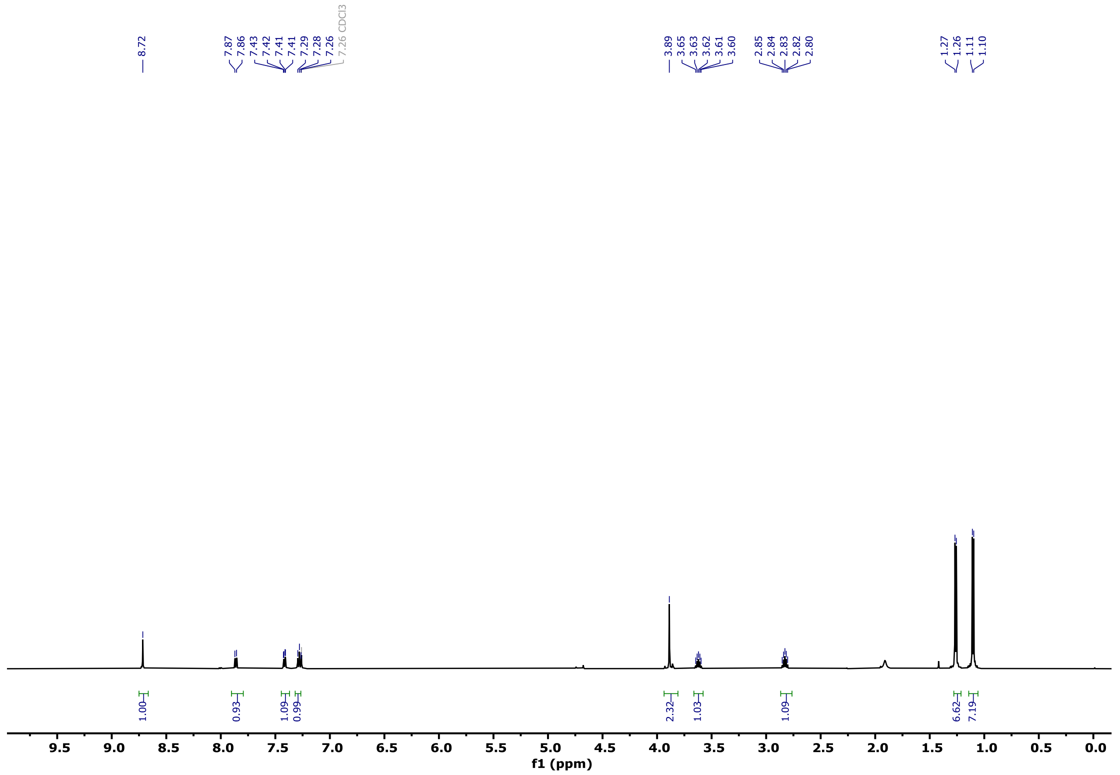

Figure S3. ^{1}H NMR (500 MHz) of (L^1Br) in $CDCl_3$ at 298 K.

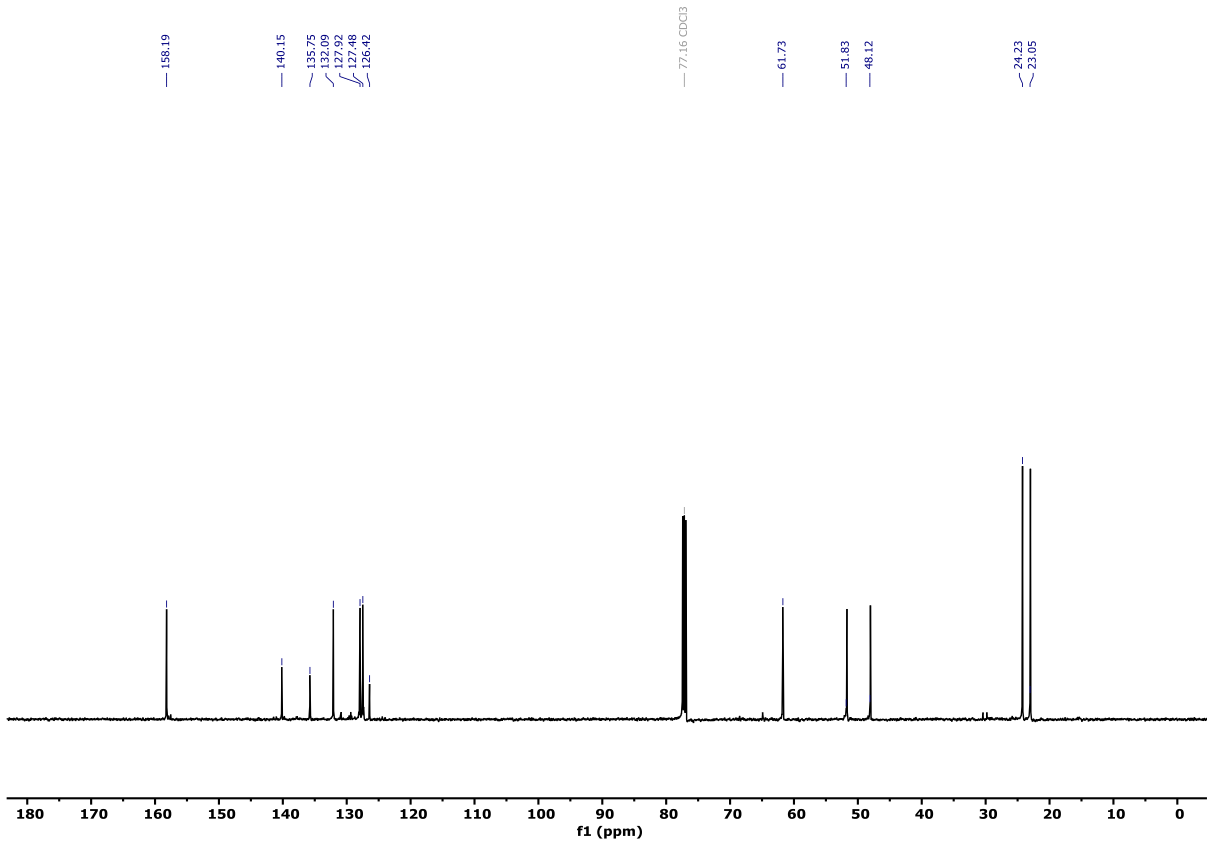

Figure S4. ^{13}C NMR (126 MHz) of (L^1Br) in $CDCl_3$ at 298 K.

B) Synthesis of Ligand L^3Br

2-bromo-3-(bromomethyl) benzaldehyde (A) was prepared following the procedure discussed in the previous section Step 1.

Step 2: Synthesis of (E)-N-(2-bromo-3-((tert-butylimino)methyl)benzyl)-2-methylpropan-2-amine (L^3Br)

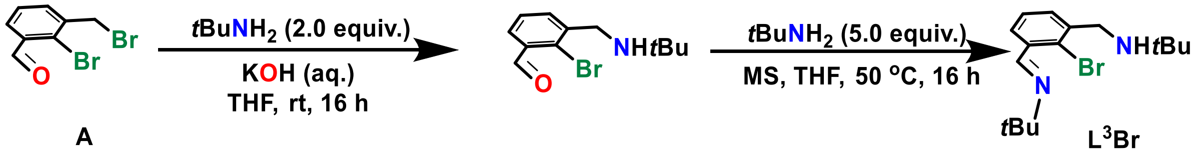

Scheme S3. Synthesis of (E)-N-(2-bromo-3-((tert-butylimino)methyl)benzyl)-2-methylpropan-2-amine (L^3Br)

A 25 mL round-bottomed flask equipped equipped with a magnetic stir bar was charged with **A** obtained in previous step (550 mg, 2 mmol, 1.0 equiv.), *t*BuNH$_2$ (0.42 mL, 4 mmol, 2.0 equiv.), and THF (5 mL). An aqueous KOH solution (5 M, 2mL) was then added, and the mixture was stirred overnight at ambient temperature. The organic phase was separated, dried over Na$_2$SO$_4$, and concentrated to afford a yellow white solid product. This product was used in the next step without further purification.

A 100 mL two-necked round-bottomed flask with a stir bar was charged with product obtained in step 3, *t*BuNH$_2$ (1.1 mL, 10.4 mmol, 5.0 equiv.), MS (4 Å) and 10 mL dry THF (first distilled) under N$_2$. The reaction was stirred for 48 hours at 50°C and the completion of the reaction was monitored by NMR. The mixture was filtered through celite and eluted with dichloromethane and ethyl acetate. The filtrate was concentrated under reduced pressure to afford a 530 mg yellow semi-viscous oil.[2]

NMR of L^{3}Br

^{1}H NMR (500 MHz, CDCl$_3$): *δ* 8.68 (s, 1H), 7.84 (dd, *J* = 7.6, 1.8 Hz, 1H), 7.49 (dd, *J* = 7.5, 1.8 Hz, 1H), 7.29 (t, *J* = 7.6 Hz, 1H), 3.85 (s, 2H), 1.32 (s, 9H), 1.21 (s, 9H)

^{13}C NMR (125 MHz, CDCl$_3$): *δ* 155.87, 140.94, 136.26, 136.08, 132.00, 127.63, 127.51, 127.06, 126.71, 58.04, 51.04, 47.56, 29.81, 29.21

HR-MS (ESI$^+$) (**L^3:** C$_{16}$H$_{26}$BrN$_2$, M.W = 325.1201) calculated for [**L^3+H**]$^+$ 324.1290; found 324.1301.

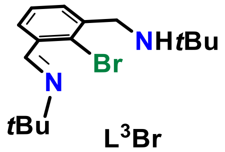

L^{3}Br

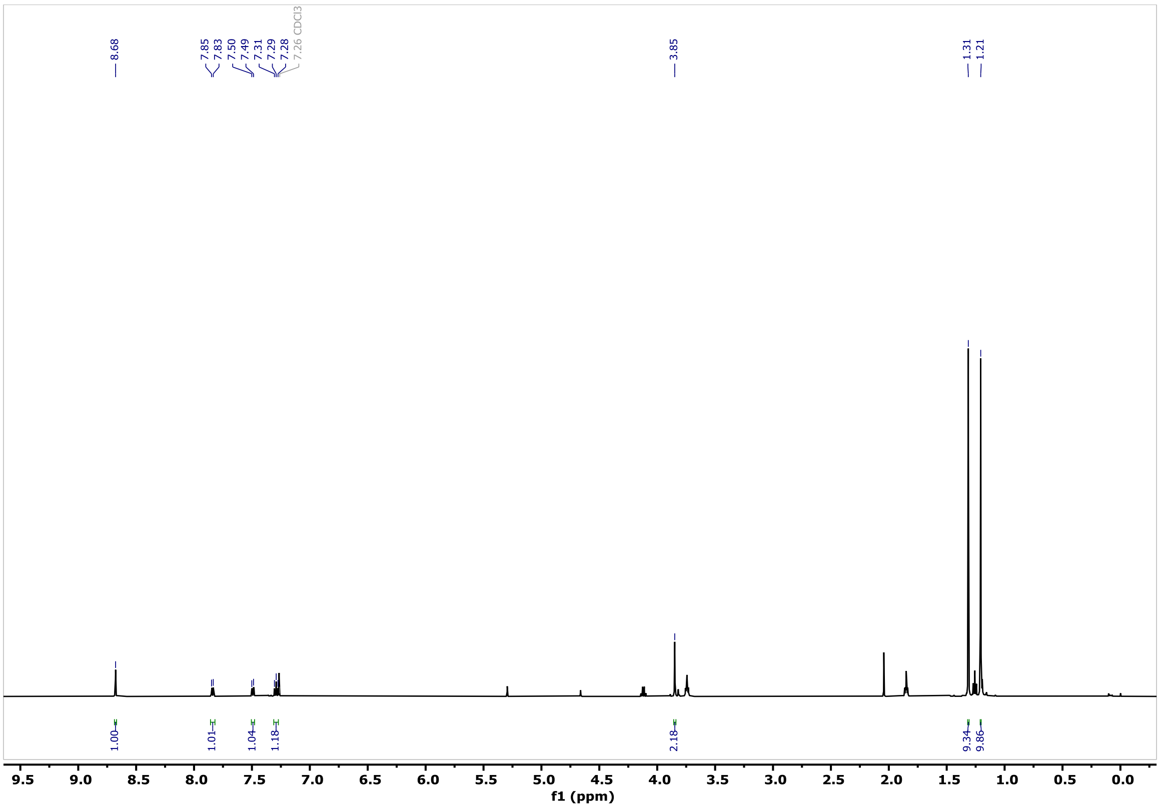

Figure S5. ^{1}H NMR (500 MHz) of ($L^{3}Br$) in $CDCl_3$ at 298 K.

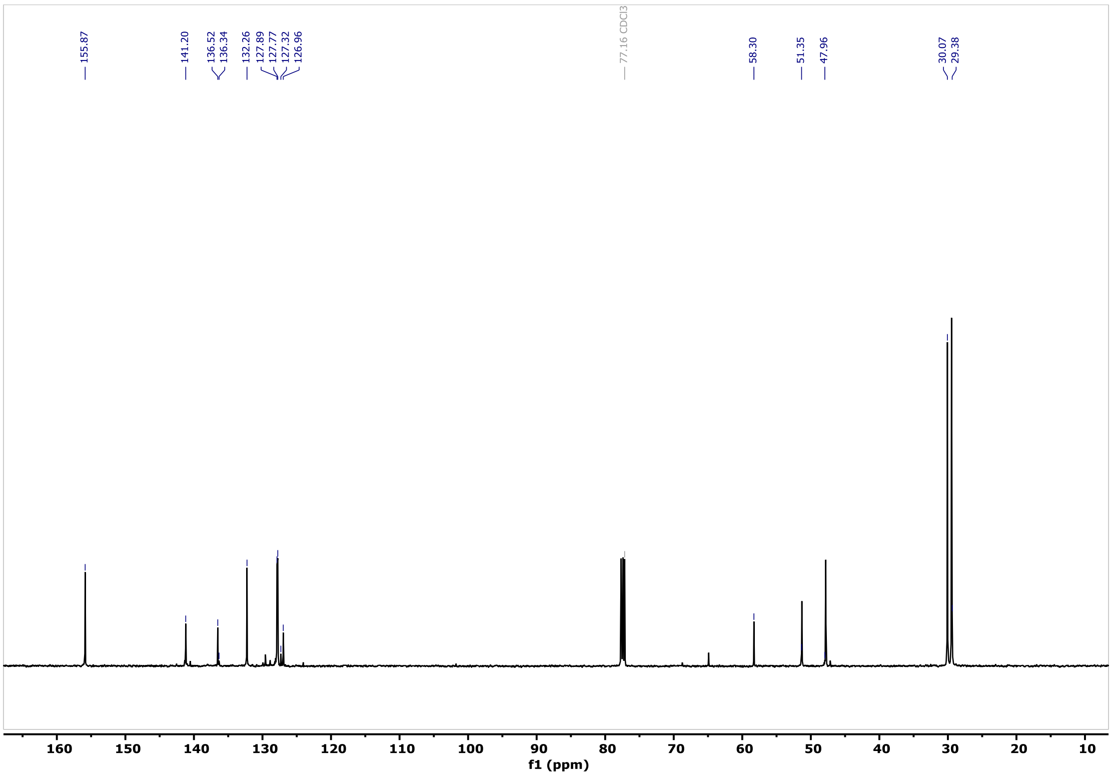

Figure S6. ^{13}C NMR (126 MHz) of ($L^{3}Br$) in $CDCl_3$ at 298 K.

C) Synthesis of Ligand L^2Br

Step 1: Synthesis of (1E,1'E)-1,1'-(2-Bromo-1,3-phenylene) bis(N-*iso*-propyl methanamine)

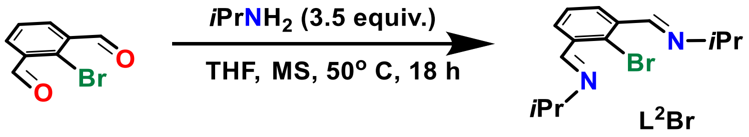

Scheme S4. Synthesis of (1E,1'E)-1,1'-(2-Bromo-1,3-phenylene) bis(N-*iso*-propyl methanamine) (L^2Br)

A 100 mL two-necked round bottom flask equipped with a magnetic stir bar and a reflux condenser was charged with 2-bromoisophthalaldehyde (1.00 g, 4.7 mmol, 1.00 equiv.) and activated 4 Å molecular sieves under argon. To this mixture, dry tetrahydrofuran (THF) (40 mL) and *iso*-propyl amine $^{i}PrNH_2$ (1.4 mL, 16.4 mmol, 3.50 equiv.) were added, and the resulting mixture was stirred at 50 °C for 20 h under reflux. After cooling down to room temperature, the mixture was filtered through a pad of Celite and washed with tetrahydrofuran (THF) (2 × 20 mL). After removing the solvent and drying the resulting solid in high vacuum for 4 h (to remove residual *tert*-butylamine), 1.29 g (93 %) of the desired bisimine (L^2Br) was obtained as a yellow solid.[3]

NMR of (L^2Br)

^{1}H NMR (500 MHz, $CDCl_3$): δ 8.74 (s, 2H), 8.01 (d, J = 7.6 Hz, 2H), 7.33 (t, J = 7.6 Hz, 1H), 3.63 (hept, J = 6.4 Hz, 2H), 1.27 (d, J = 6.4 Hz, 18H).

^{13}C NMR (125 MHz, $CDCl_3$): δ 157.78, 135.88, 131.14, 127.89, 127.08, 62.02, 24.46.

HR-MS (ESI$^+$) (**L^2:** $C_{14}H_{19}BrN_2$, M.W = 295.2240) calculated for $[L^2+H]^+$ 295.0810; found 295.0812.

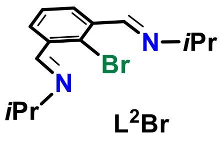

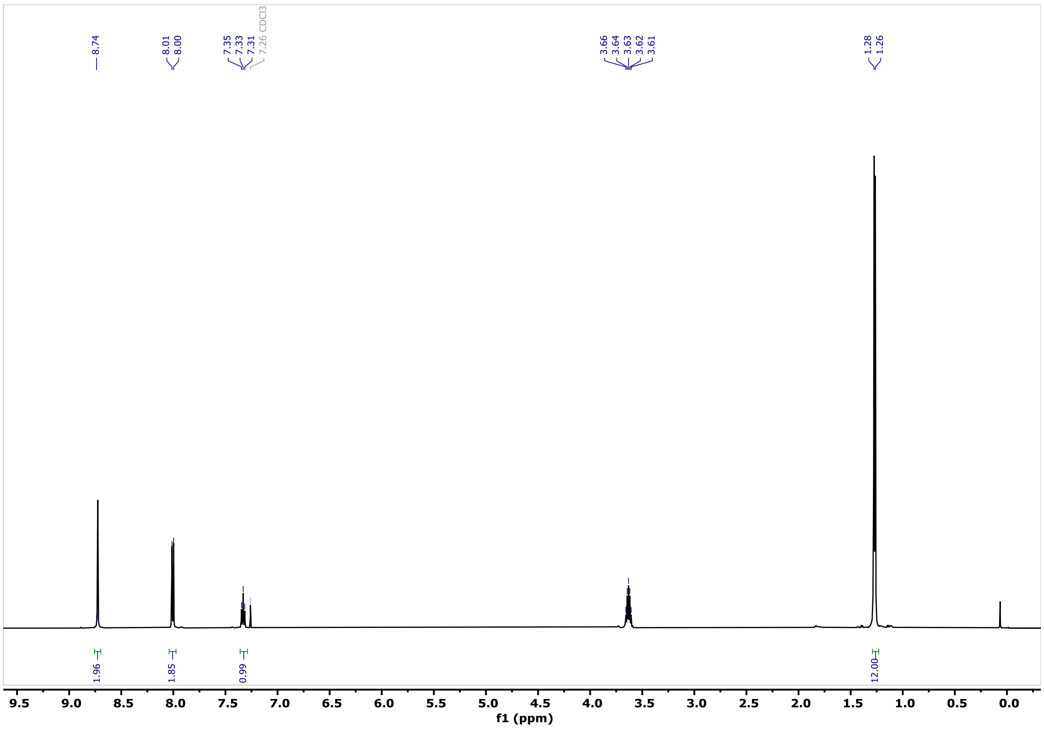

Figure S7. ^{1}H NMR (500 MHz) of (L^2Br) in $CDCl_3$ at 298 K.

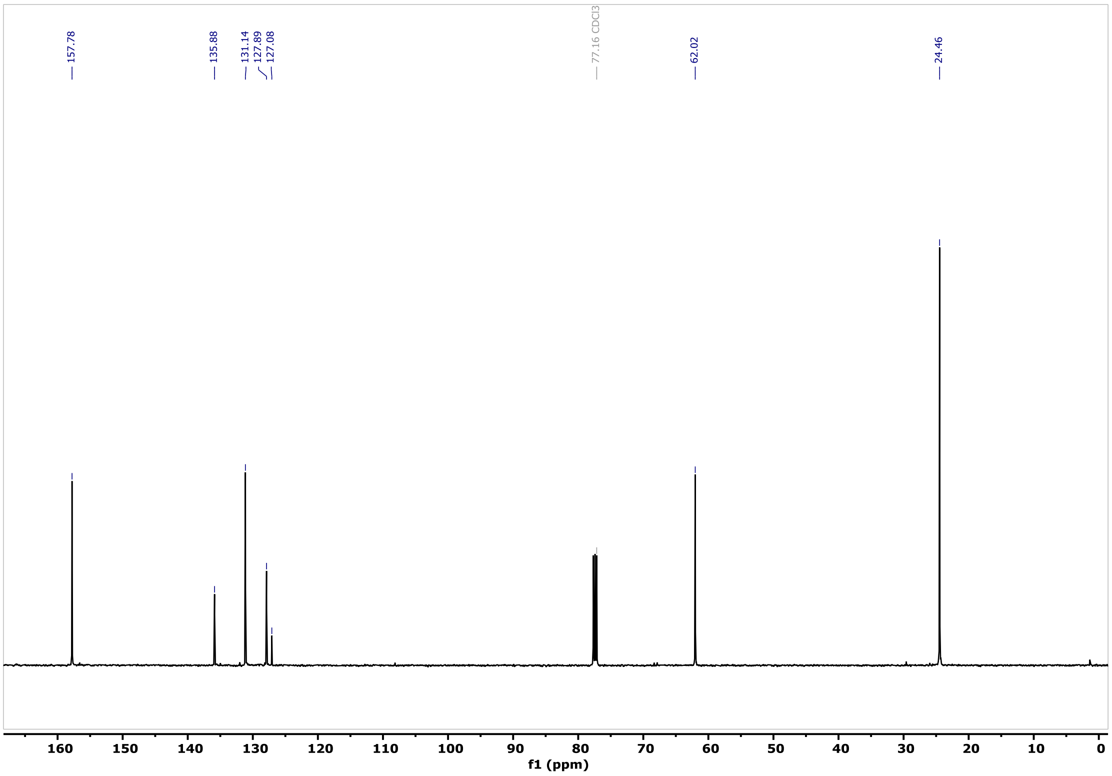

Figure S8. ^{13}C NMR (126 MHz) of (L^2Br) in $CDCl_3$ at 298 K.

D) Synthesis of Ligand L⁴Br

Step 1: (1E,1'E)-1,1'-(2-Bromo-1,3-phenylene) bis(N-*tert*-butyl methanamine)

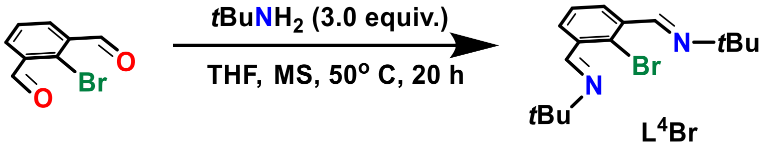

Scheme S5. Synthesis of (1E,1'E)-1,1'-(2-Bromo-1,3-phenylene) bis(N-*tert*-butyl methanamine) (L^4Br)

A 100 mL two-necked round bottom flask equipped with a magnetic stir bar and a reflux condenser was charged with 2-bromoisophthalaldehyde (3.5 g, 16.4 mmol, 1.00 equiv.) and activated 4 Å molecular sieves under argon. To this mixture, dry tetrahydrofuran (THF) (40 mL) and *tert*-butylamine *tBu*NH_2 (4.9 mL, 57.5 mmol, 3.50 equiv.) were added, and the resulting mixture was stirred at 50 °C for 20 h under reflux. After cooling down to room temperature, the mixture was filtered through a pad of Celite and washed with tetrahydrofuran (THF) (2 X 20 mL). After removing the solvent and drying the resulting solid in high vacuum for 4 h (to remove residual *tert*-butylamine), 5.25 g (99 %) of the desired bisimine (L^4Br) was obtained.[3]

NMR of (L^4Br)

^{1}H NMR (500 MHz, $CDCl_3$): δ 8.68 (s, 2H), 7.99 (d, J = 7.6 Hz, 2H), 7.35 (t, J = 7.6 Hz, 1H), 1.33 (s, 18H).

^{13}C NMR (125 MHz, $CDCl_3$): δ 155.12, 136.35, 130.56, 127.89, 127.59, 58.41, 29.97.

HR-MS (ESI$^+$) (**L^4:** $C_{16}H_{23}BrN_2$, M.W = 323.2780) calculated for $[L^4+H]^+$ 323.1123; found 323.1203.

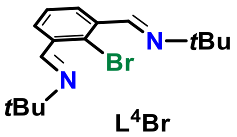

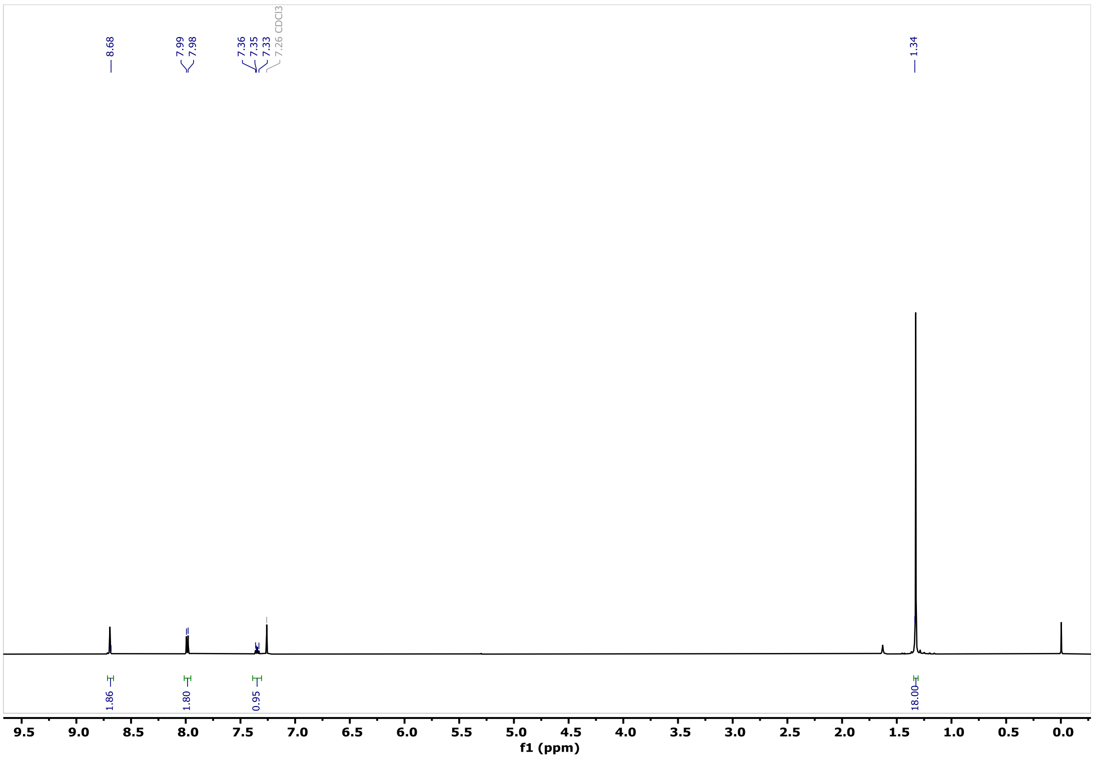

Figure S9. ^1H NMR (500 MHz) of (L^4Br) in $CDCl_3$ at 298 K

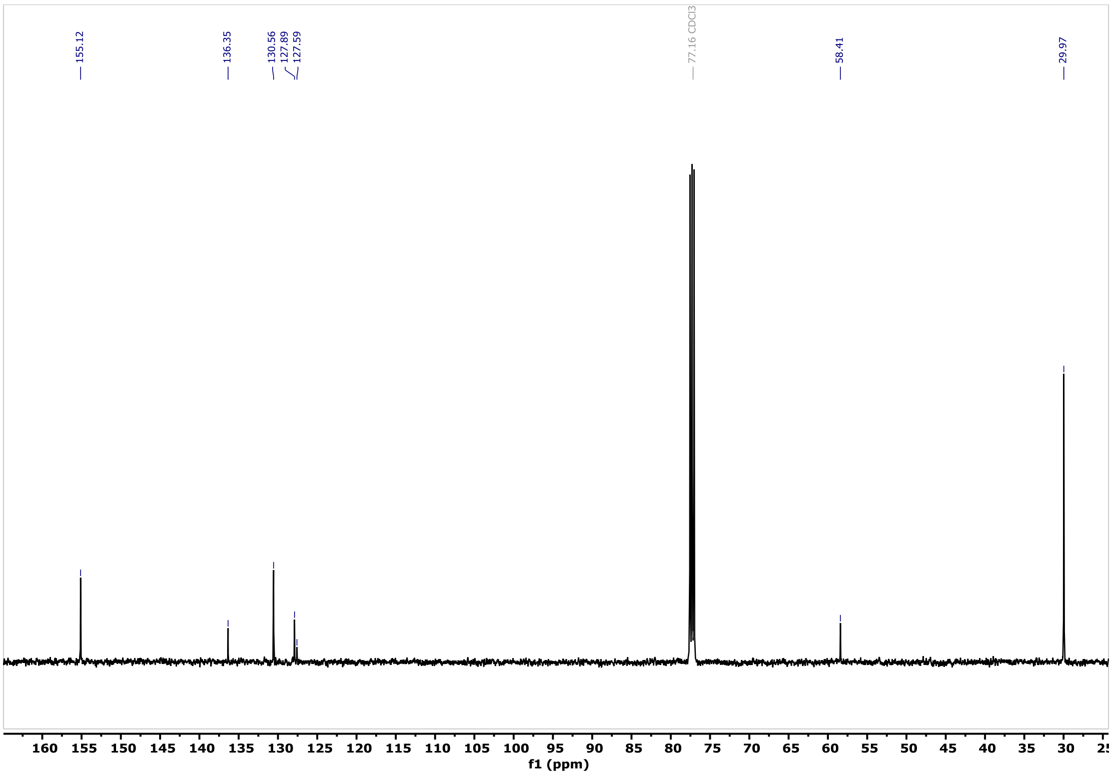

Figure S10. ^{13}C NMR (126 MHz) of (L^4Br) in $CDCl_3$ at 298 K.

Synthesis of Bi(III)- Complexes (II)

A) Synthesis of Dichlorobismuthine [(L[1])BiCl2] (1)

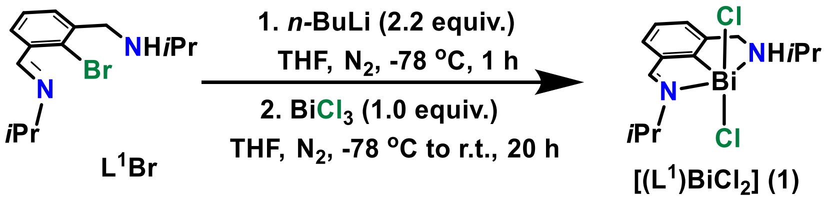

Scheme S6. Synthesis of Dichlorobismuthine [(L^1)$BiCl_2$] **(1)**

An oven dried 100 mL two-necked round-bottomed flask equipped with a magnetic stir bar was charged with previously prepared ligand (L^1Br) (1.12 g, 3.8 mmol, 1 equiv.) in 50 mL dry tetrahydrofuran (THF) under N_2 and was cooled to − 78 °C. Then *n*-BuLi (4.18 mL, 8.32 mmol, 2.2 equiv., 2 M in cyclohexane) was added dropwise to the pre-cooled mixture. After complete addition, the solution turned into dark red color. The temperature was maintained at − 78 °C for a period of 2 hours under stirring. After 2 hours a solution of $BiCl_3$ (1.1 g, 3.8 mmol, 1.0 equiv.) dissolved in dry tetrahydrofuran (THF) (around 10 mL) was added to the pre-cooled reaction mixture and stirred for 10 - 15 minutes before being brought to room temperature. The resulting brown mixture was stirred and allowed to reach at ambient temperature overnight. The following workup was carried out under air. All volatiles were removed under vacuum. The resulting solids were redissolved in dichloromethane and filtered through celite. The filtrate was concentrated under vacuum to 5 mL, and hexane was used to precipitate the product which was filtered with a filter-frit and washed with 100 mL of hexane to yield (0.97 g, 51%) of Dichlorobismuthine [(L^1)$BiCl_2$] (**1**) as yellow solid. Crystals suitable for single crystal X-Ray diffraction studies were grown from 1:1 dichloromethane: ethyl acetate solvent mixture via layering method at room temperature and diffracted at 100 K.[2]

NMR of Dichlorobismuthine [(L^1)$BiCl_2$] (1)

^{1}H NMR (500 MHz, $CDCl_3$): δ 9.46 (s, 1H), 7.91 (t, *J* = 7.4 Hz, 2H), 7.67 (t, *J* = 7.5 Hz, 1H), 5.37 (dd, *J* = 14.7, 5.4 Hz, 1H), 4.26 – 4.16 (m, 1H), 3.96 – 3.91 (m, 1H), 3.62 – 3.55 (m, 1H), 1.53 – 1.47 (m, 12H), 1.37 (s, 3H).

^{13}C NMR (125 MHz, CD_3CN): δ 196.36, 170.41, 151.75, 146.90, 132.44, 132.14, 129.27, 60.52, 54.56, 51.45, 24.80, 21.92.

Note : Due to the high quadrupole moment of the ^{209}Bi nucleus (100%, I = 9/2, quadrupole moment −0.4 x 10^{-28} m^{-2}), which broaden the peaks corresponding to atoms bonded to the Bi center to such an extent that they are not observable under standard conditions, so here

carbon corresponding to the C-Bi bond is also not observable prominently in ^{13}C NMR spectroscopy. (See Figure S12)[4]

HR-MS (ESI$^+$) (1: $C_{14}H_{21}BiCl_2N_2$, M.W = 497.2164**)** calculated for [**1**–Cl]$^+$ 461.1197; found 461.1266

IR: 2966 (br), 2916(m), 1622 (s), 1568 (m),1388 (s),1136 (m), 789 (vs) cm^{-1}

UV-Visible Spectroscopy: Complex **[(L^1)BiCl$_2$] (1)** (1.6 x 10^{-5} M) in CH_2Cl_2 shows λ_{max} 232 nm (ε_{232} = 21625 M^{-1} cm^{-1}), 300 nm (ε_{300}= 7500 M^{-1} cm^{-1}), measured at 298 K in quartz cuvette with path length 1 cm.

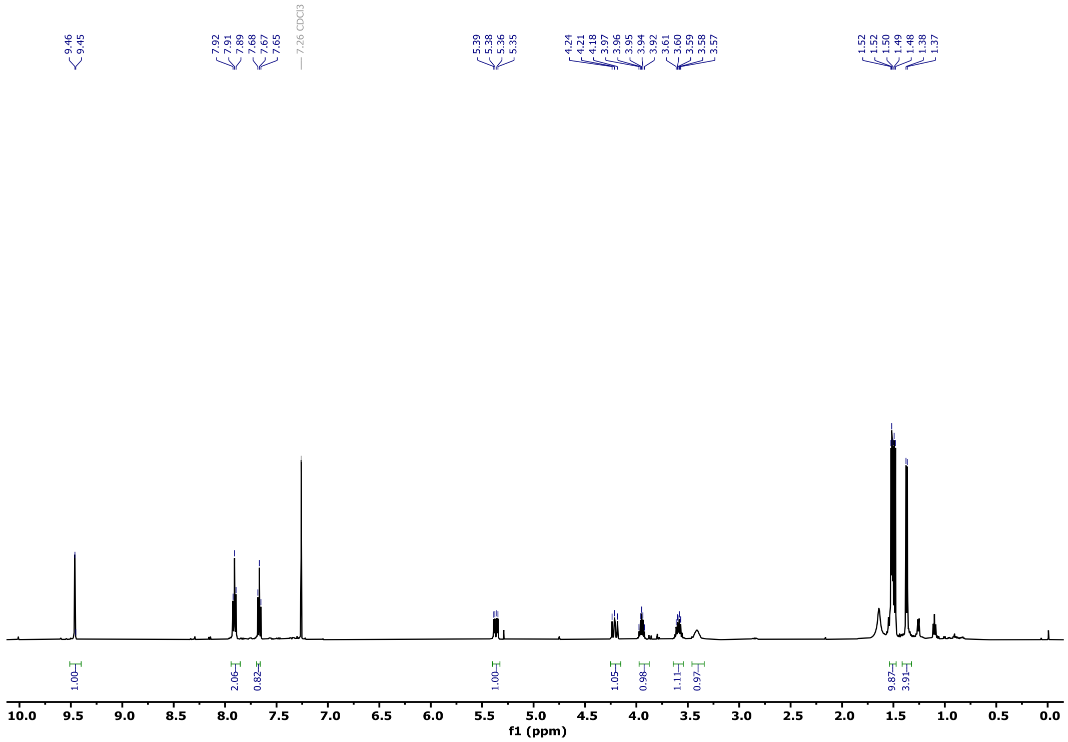

Figure S11. ^1H NMR (500 MHz) of Dichlorobismuthine [(L^1)BiCl$_2$] (**1**) in $CDCl_3$ at 298 K.

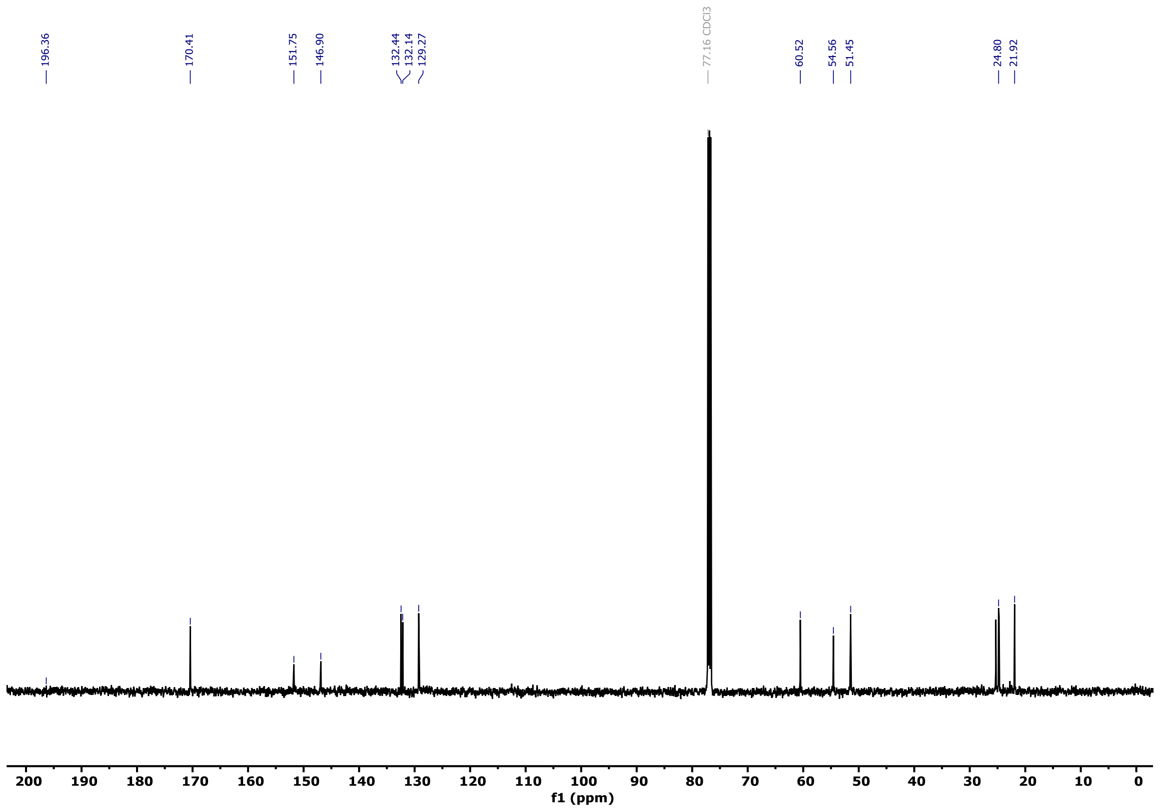

Figure S12. ^{13}C NMR (126 MHz) of Dichlorobismuthine [(L[1])BiCl$_2$] (**1**) in CDCl$_3$ at 298 K.

2-D COSY NMR

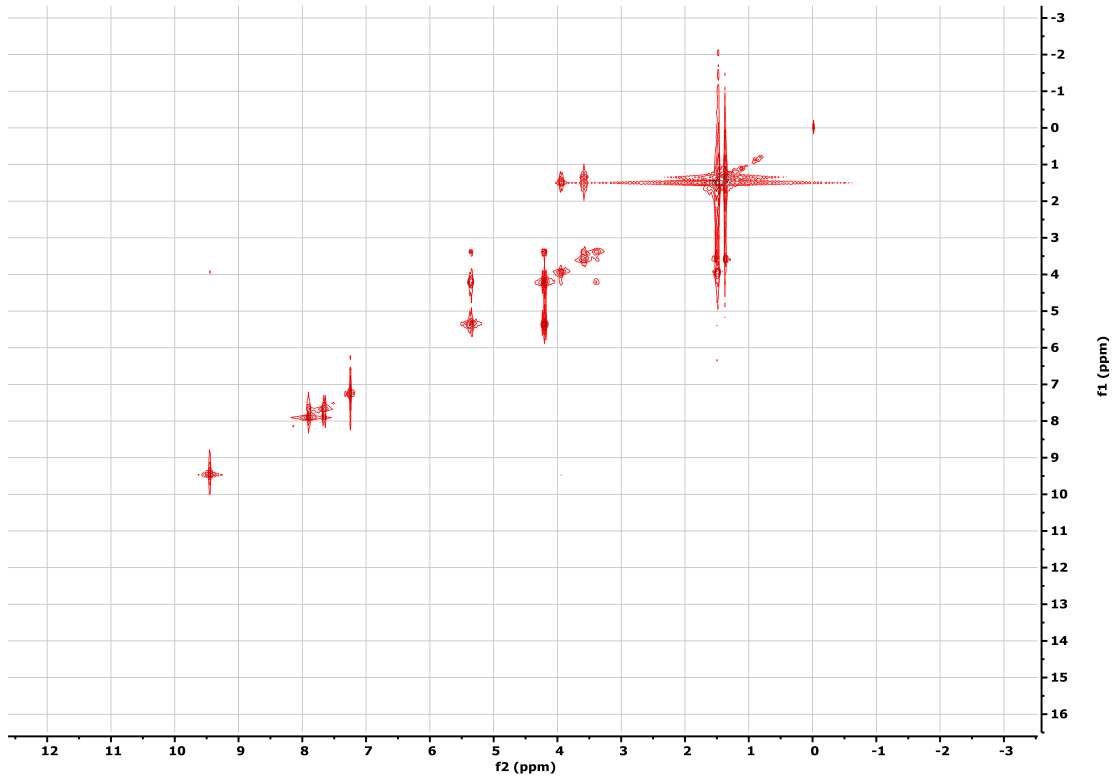

Figure S13. COSY NMR (500 MHz) of Dichlorobismuthine [(L[1])BiCl$_2$] (**1**) in CDCl$_3$ at 298 K.

2D-NOESY NMR

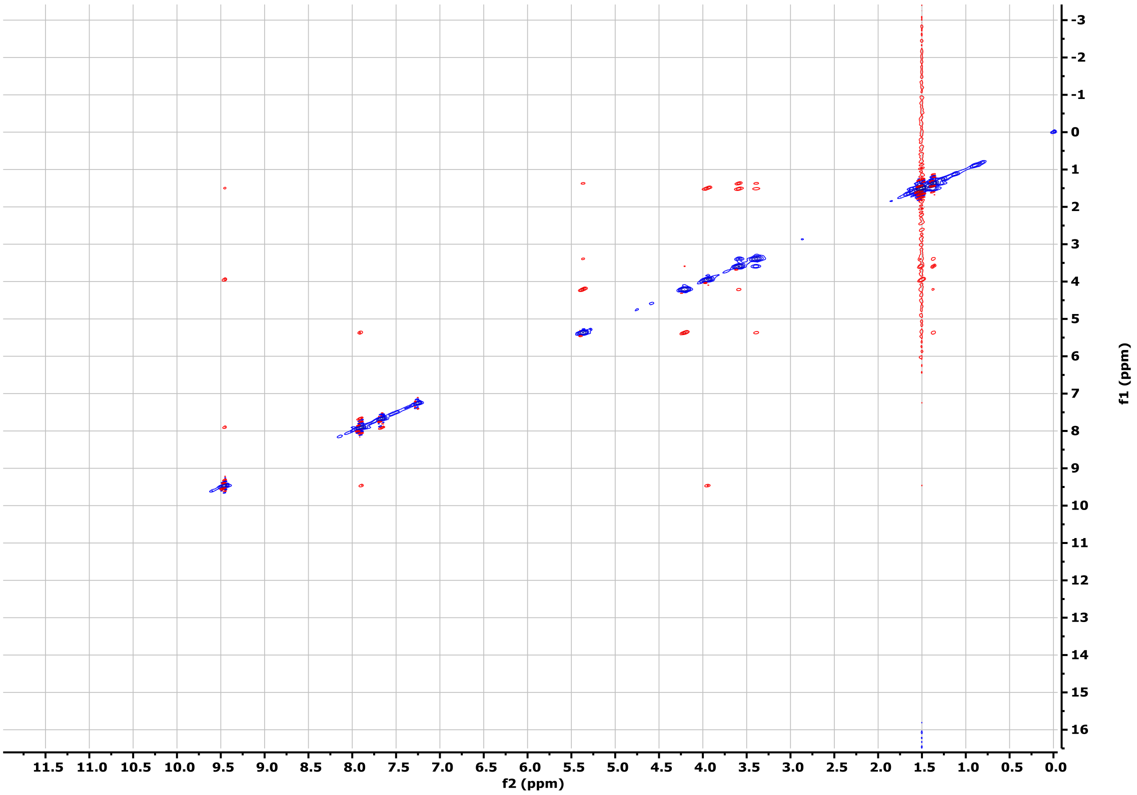

Figure S14. NOESY NMR (500 MHz) of Dichlorobismuthine [(L^1)$BiCl_2$] (**1**) in $CDCl_3$ at 298 K.

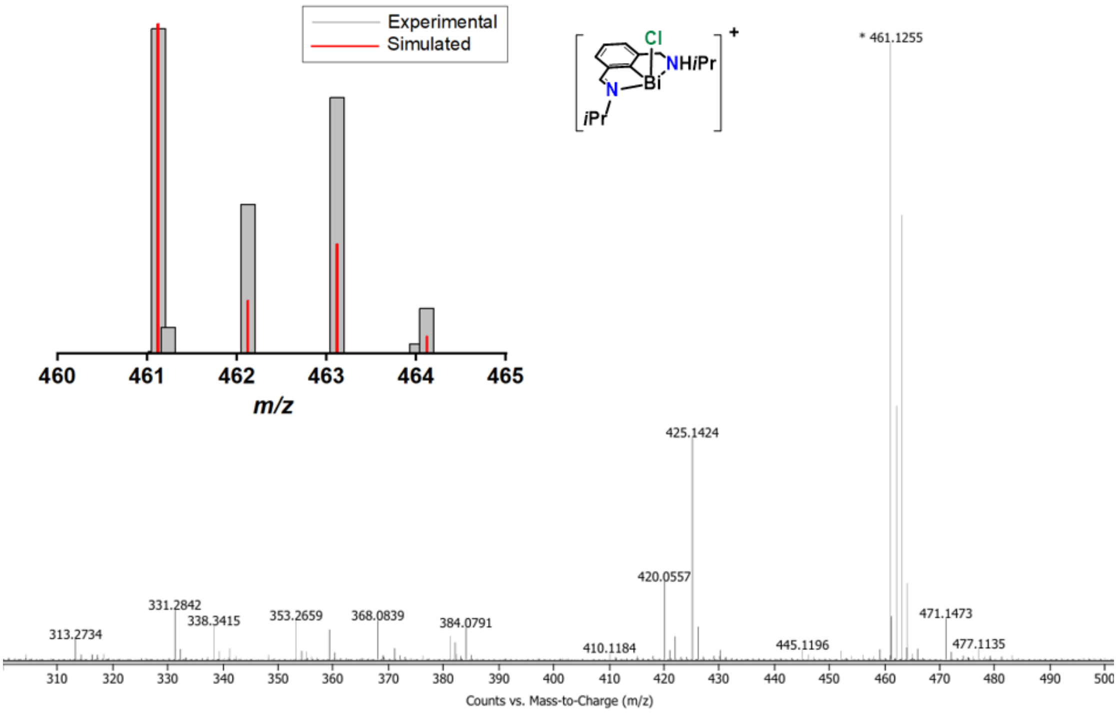

Figure S15. HR-MS (ESI$^+$) in MeOH of **(1)** $C_{14}H_{21}BiCl_2N_2$, (M.W = 497.2164**)** calculated for [**1**–Cl]$^+$ 461.1197; found 461.1255.

UV of Bismuth [(L^1)BiCl$_2$] (1) Complex at different concentrations

UV-Vis spectroscopic measurements were taken at room temperature (298 K) in quartz cuvette with path length = 1 cm. CH_2Cl_2 was used as the solvent, varying concentration of the [(L^1)BiCl$_2$] **(1)**.

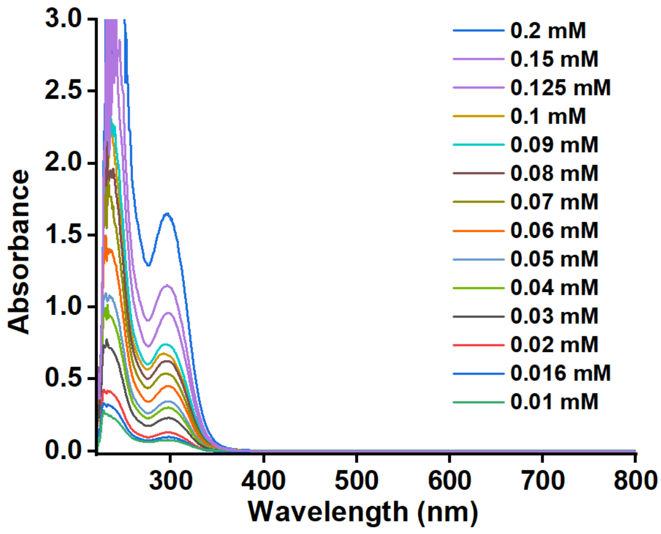

Figure S16: UV-Vis spectra of various concentration of catalyst [(L^1)BiCl$_2$] **(1)** in dichloromethane at 298 K

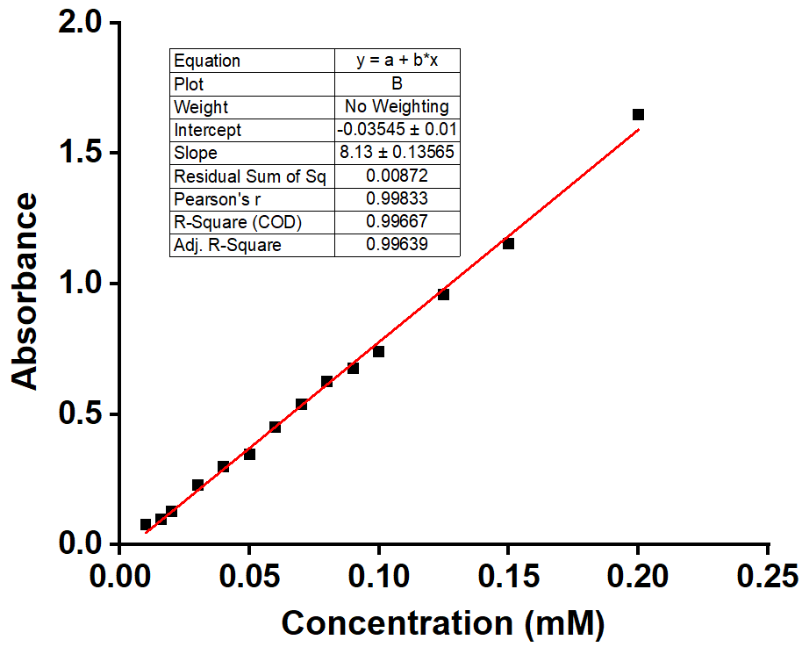

Figure S17. Absorbance vs concentration plot of catalyst [(L[1])BiCl$_2$] **(1)** at λ_{max} 295 nm (slope =8.13; epsilon (ε) = 8.13 x 10^3 L mol^{-1} cm^{-1}).

B) Synthesis of Dichlorobismuthine [(L^2)$BiCl_2$] (2)

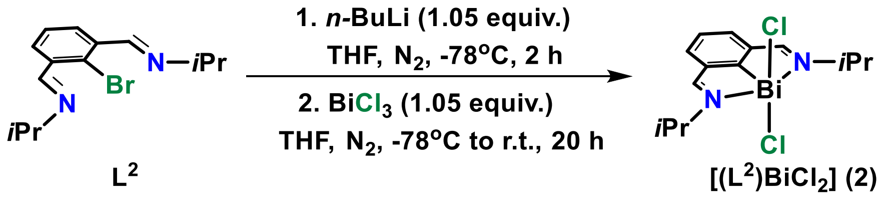

Scheme S7. Synthesis of Dichlorobismuthine [(L^2)$BiCl_2$] **(2)**

An oven dried 100 mL two-necked round-bottomed flask equipped with a magnetic stir bar was charged with previously prepared ligand (L^2) (1 g, 3.4 mmol, 1 equiv.) in 50 mL dry tetrahydrofuran (THF) under N_2 and was cooled to −78 °C. Then *n*-BuLi (2.25 mL, 3.5 mmol, 1.05 equiv., 1.6 M in hexane) was added dropwise to the pre-cooled mixture. After complete addition, the solution turned into dark red. The temperature was maintained at −78 °C for a period of 2 hours under stirring. After 2 hours a solution of $BiCl_3$ (1.12 g, 3.57 mmol, 1.05 equiv.) dissolved in dry tetrahydrofuran (THF) (around 15 mL) was added to the pre-cooled reaction mixture and stirred for 10 - 15 minutes before being brought to room temperature. The reaction mixture turned to a yellow color. Stirring continued at room temperature for another 20 hours. Tetrahydrofuran (THF) was removed in vacuum and the residue obtained was resuspended in 40 - 50 mL CH_2Cl_2. It was then filtered using a celite pad leaving yellow-colored filtrate. The filtrate was then concentrated to 20 mL of dichloromethane and hexane was added to it. A yellow precipitate was formed which was filtered with a filter-frit and washed with 200 mL of hexane to yield (1.14 g, 65%) of Dichlorobismuthine [(L^2)$BiCl_2$] **(2)** as a yellow solid. Crystals suitable for single crystal X-Ray diffraction studies were grown from 1:1 dichloromethane: ethyl acetate solvent mixture via layering method at room temperature and diffracted at 100 K.[3]

NMR of Dichlorobismuthine [(L^2)$BiCl_2$] (2)

^{1}H NMR (500 MHz, $CDCl_3$): δ 9.62 (d, J = 1.2 Hz, 2H), 8.16 (d, J = 7.5 Hz, 2H), 7.83 (t, J = 7.6 Hz, 1H), 4.04 (hept, J = 6.6 Hz, 2H), 1.55 (d, J = 6.4 Hz, 12H).

^{13}C NMR (125 MHz, $CDCl_3$): δ 196.31, 169.93, 147.15, 136.39, 129.67, 61.30, 25.24.

HR-MS (ESI$^+$) (**2:** $C_{14}H_{19}BiCl_2N_2$, M.W = 495.2004) calculated for $[2{-}Cl]^+$ 459.1039; found 459.1041

IR: 2963 (vs), 2923 (w), 1625 (s), 1568 (s), 753 (vs) cm^{-1}

UV-Visible Spectroscopy: [(L^2)$BiCl_2$] (2) (1.6 x 10^{-5} M) in CH_2Cl_2 shows λ_{max} 249 nm (ε_{249} = 20000 M^{-1} cm^{-1}), 284 nm (ε_{284}= 9063 M^{-1} cm^{-1}), measured at 298 K in quartz cuvette with path length 1 cm.

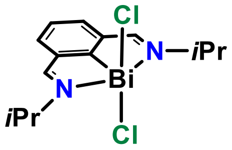

[(L^2)$BiCl_2$] (2)

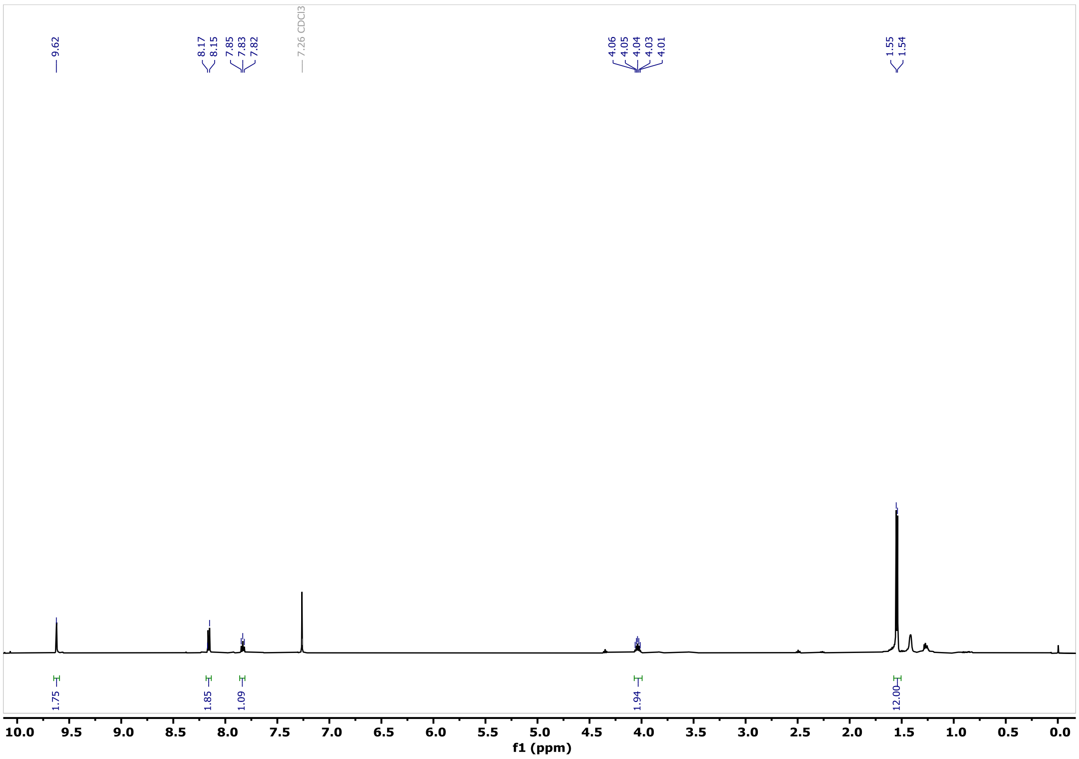

Figure S18. ^1H NMR (500 MHz) of Dichlorobismuthine [(L^2)$BiCl_2$] (**2**) in $CDCl_3$ at 298 K.

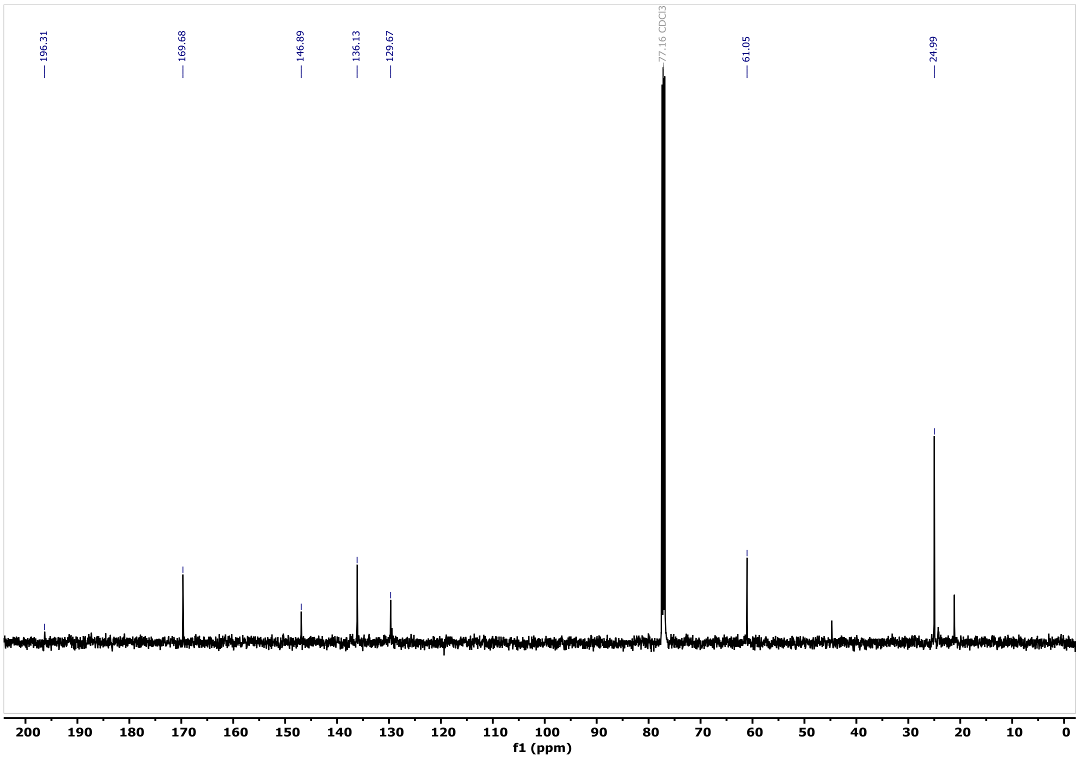

Figure S19. ^{13}C NMR (126 MHz) of Dichlorobismuthine [(L^2)$BiCl_2$] (**2**) in $CDCl_3$ at 298 K

2D-NMR COSY DATA

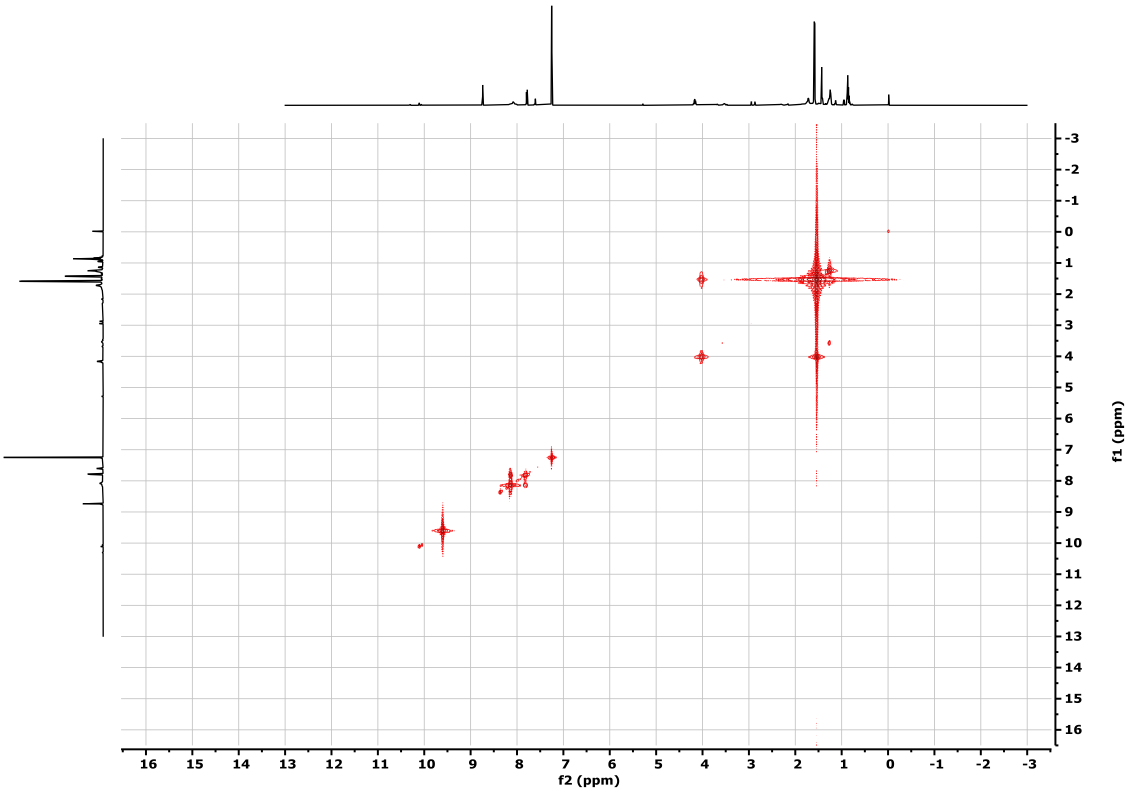

Figure S20. COSY NMR (500 MHz) of Dichlorobismuthine [(L^2)$BiCl_2$] (**2**) in $CDCl_3$ at 298 K

2D-NOESY DATA

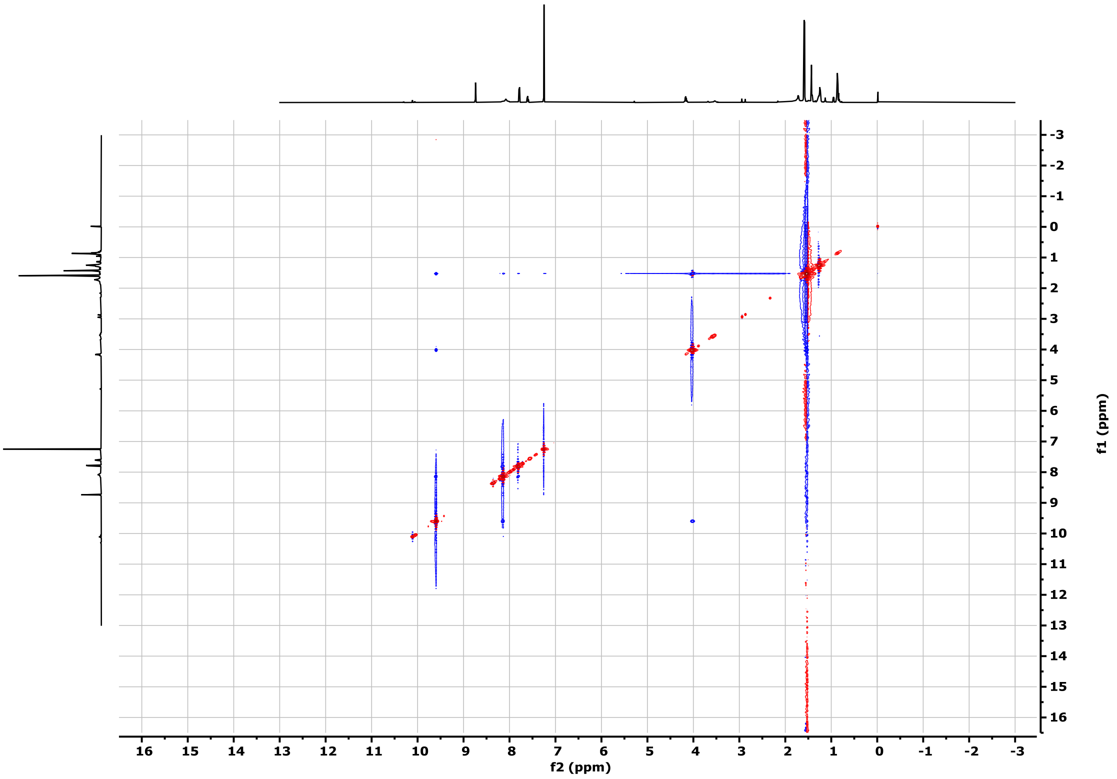

Figure S21. NOESY NMR (500 MHz) of Dichlorobismuthine [(L^2)BiCl$_2$] (**2**) in $CDCl_3$ at 298 K.

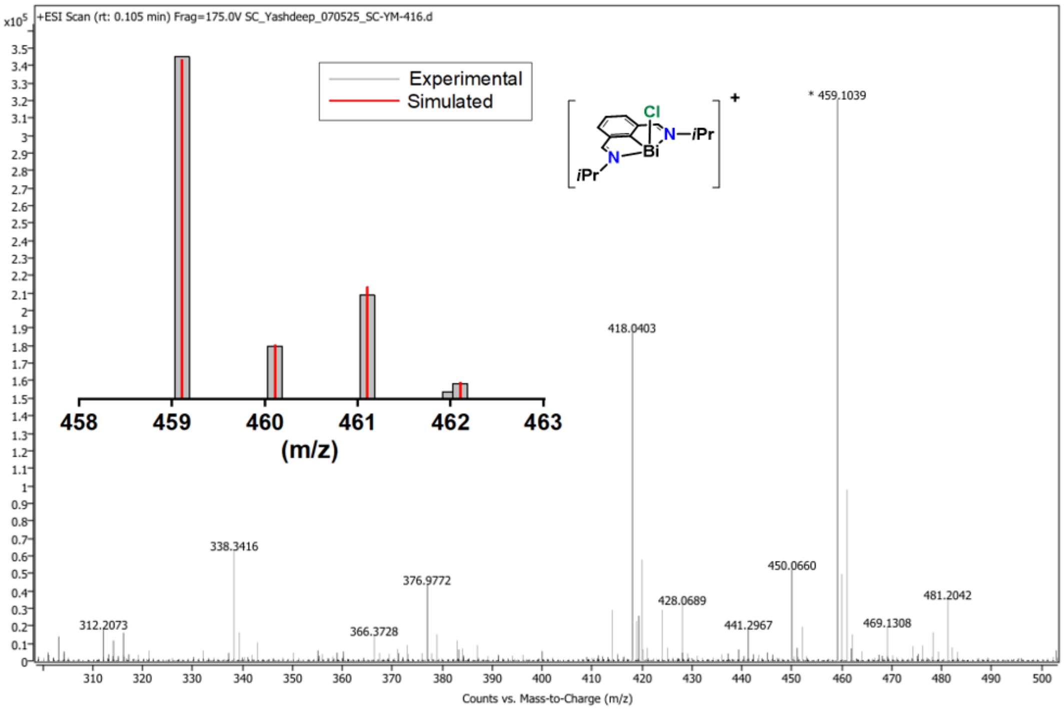

Figure 22. HR-MS (ESI$^+$) in MeOH for [(L^2)BiCl$_2$] **(2)** $C_{14}H_{19}BiCl_2N_2$, (M.W = 495.2004) calculated for [**2**–Cl]$^+$ 459.1039; found 459.1039.

UV of Bismuth [(L²)BiCl₂] (2) Complex at different concentrations

UV-vis spectroscopic measurements were taken at room temperature (298 K) in quartz cuvette with path length = 1 cm. CH_2Cl_2 was used as the solvent, varying concentration of the bismuth isopropyl complex.

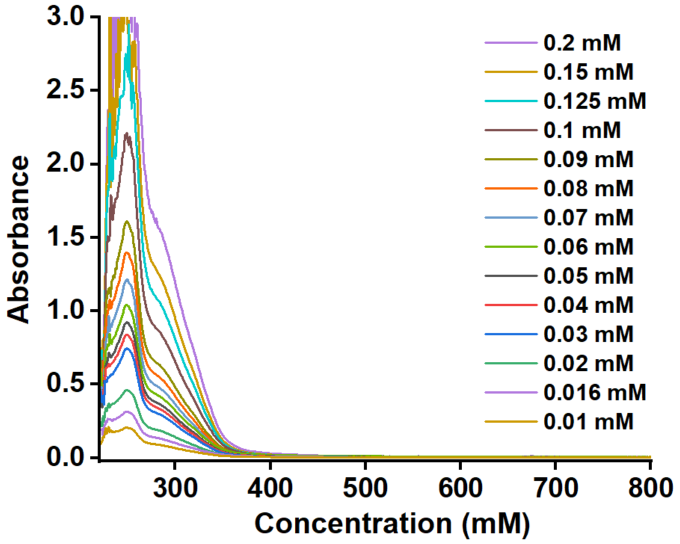

Figure S23: UV-vis spectra of various concentration of catalyst **$[(L^2)BiCl_2]$ (2)** in dichloromethane at 298 K

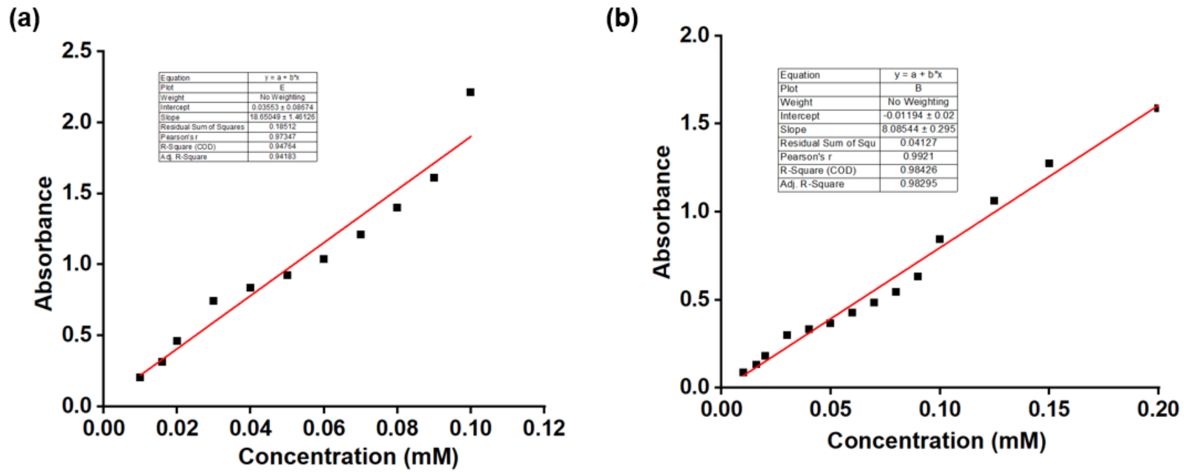

Figure S24: Absorbance vs Concentration plot of **$[(L^2)BiCl_2]$ (2)** at (a) λ_{max} 285 nm shows slope of 8.085 and epsilon (ε) value = 8.085 x 10^3 L mol^{-1} cm^{-1}. (b) λ_{max} 250 nm gives slope = 18.65 epsilon (ε) = 18.650 x 10^3 L mol^{-1} cm^{-1}

C) Synthesis of Dichlorobismuthine [(L³)BiCl₂] (3)

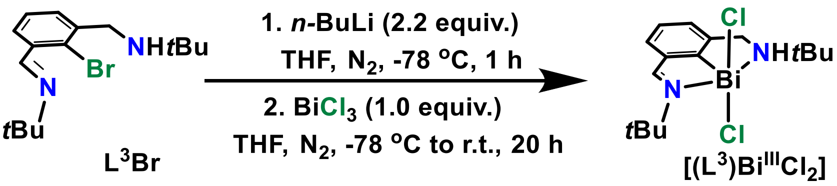

Scheme S8. Synthesis of Dichlorobismuthine [(L³)BiCl₂] **(3)**

An oven dried 100 mL two-necked round-bottomed flask equipped with a magnetic stir bar was charged with previously prepared ligand (L³) (400.0 mg, 1.23 mmol, 1 equiv.) in 20 mL dry tetrahydrofuran (THF) under N_2 and was cooled to −78 °C. Then *n*-BuLi (1.8 mL, 2.7 mmol, 2.2 equiv., 1.6 M in hexane) was added dropwise to the pre-cooled mixture. After complete addition, the solution turned into dark red color. The temperature was maintained at −78 °C for a period of 2 hours under stirring. After 2 hours a solution of $BiCl_3$ (387.8 mg, 1.23 mmol, 1.0 equiv.) dissolved in dry tetrahydrofuran (THF) (around 10 mL) was added to the pre-cooled reaction mixture and stirred for 10 - 15 minutes before being brought to room temperature. The resulting brown mixture was stirred and allowed to reach to ambient temperature overnight. The following workup was carried out under air. All volatiles were removed under vacuum. The resulting solids were redissolved in DCM and filtered through celite. The filtrate was concentrated under vacuum to 5 mL, and hexane was used to precipitate the product. Which was filtered with a filter-frit and washed with 100 mL of hexane to yield (360 mg, 55%) of Dichlorobismuthine [(L³)BiCl₂] (**3**) as white solid.[2]

NMR of Dichlorobismuthine [(L³)BiCl₂] (3)

^{1}H NMR (500 MHz, $CDCl_3$): *δ* 9.60 (s, 1H), 7.95 (t, *J* = 7.2 Hz, 2H), 7.68 (t, *J* = 7.6 Hz, 1H), 5.14 (dd, *J* = 14.4, 4.7 Hz, 1H), 4.27 (t, *J* = 13.5 Hz, 1H), 3.46 (d, *J* = 8.3 Hz, 1H), 1.58 (s, 9H), 1.58 (s, 9H).

^{13}C NMR (125 MHz, CD_3CN): *δ* 207.16, 168.68, 151.81, 148.45, 132.88, 132.84, 129.40, 61.79, 55.58, 50.32, 30.90, 29.77.

HR-MS (ESI$^+$) (3: $C_{16}H_{25}BiCl_2N_2$, M.W = 525.2704**)** calculated for [**3–Cl**]$^+$ 489.1510; found 489.1521

IR: 3398 (br), 2967 (s), 2926 (w), 1622 (s), 1456 (m), 1199 (m), 763 (s) cm^{-1}

UV-Visible Spectroscopy: Complex **[(L³)BiCl₂] (3)** (1.6×10^{-5} M) in CH_2Cl_2 shows λ_{max} 239 nm (ε_{239} = 16875 M^{-1} cm^{-1}), 300 nm (ε_{300} = 5500 M^{-1} cm^{-1}) measured at 298 K in quartz cuvette with path length 1 cm.

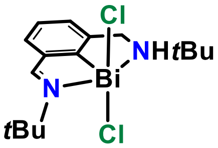

[(L[3])Bi[III]Cl2]

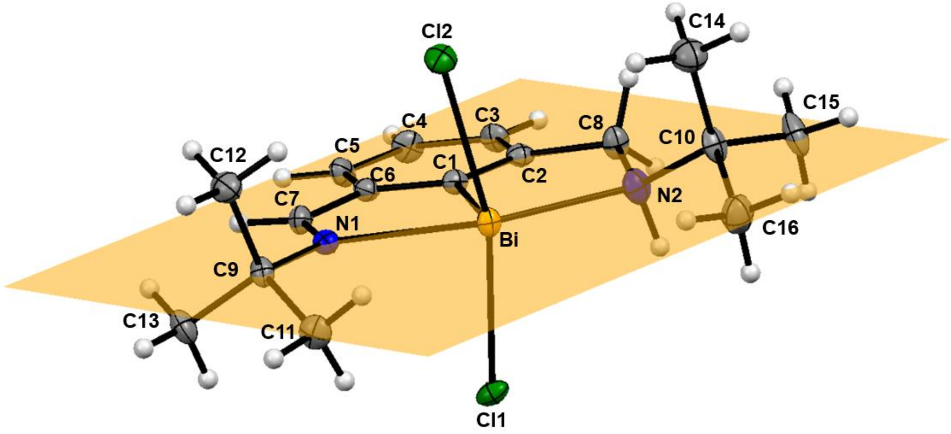

Figure S25: Structure showing non-symmetric and non-planar structure of catalyst **[(L[3])BiCl2] (3)**

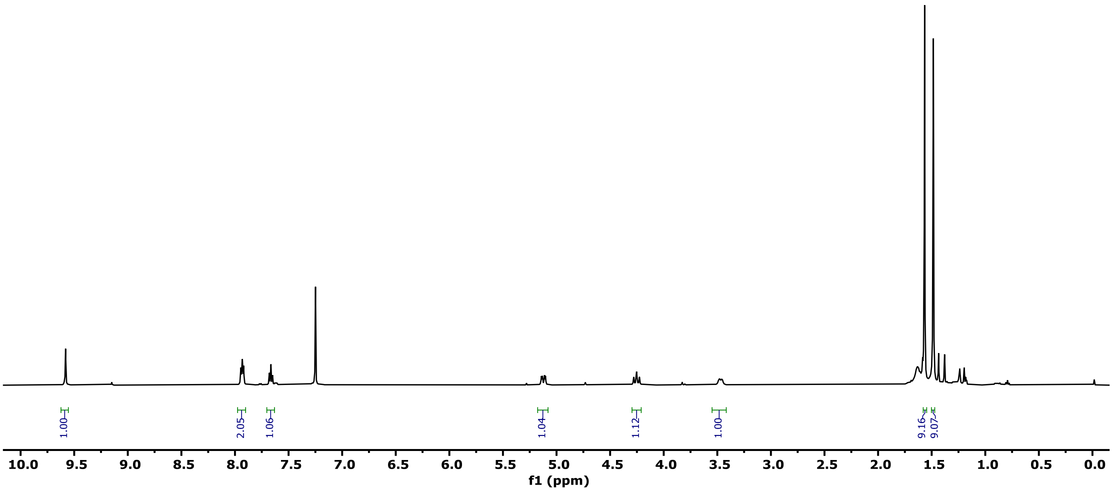

Figure S26. ^{1}H NMR (500 MHz) of Dichlorobismuthine [(L[3])BiCl2] (**3**) in $CDCl_3$ at 298 K.

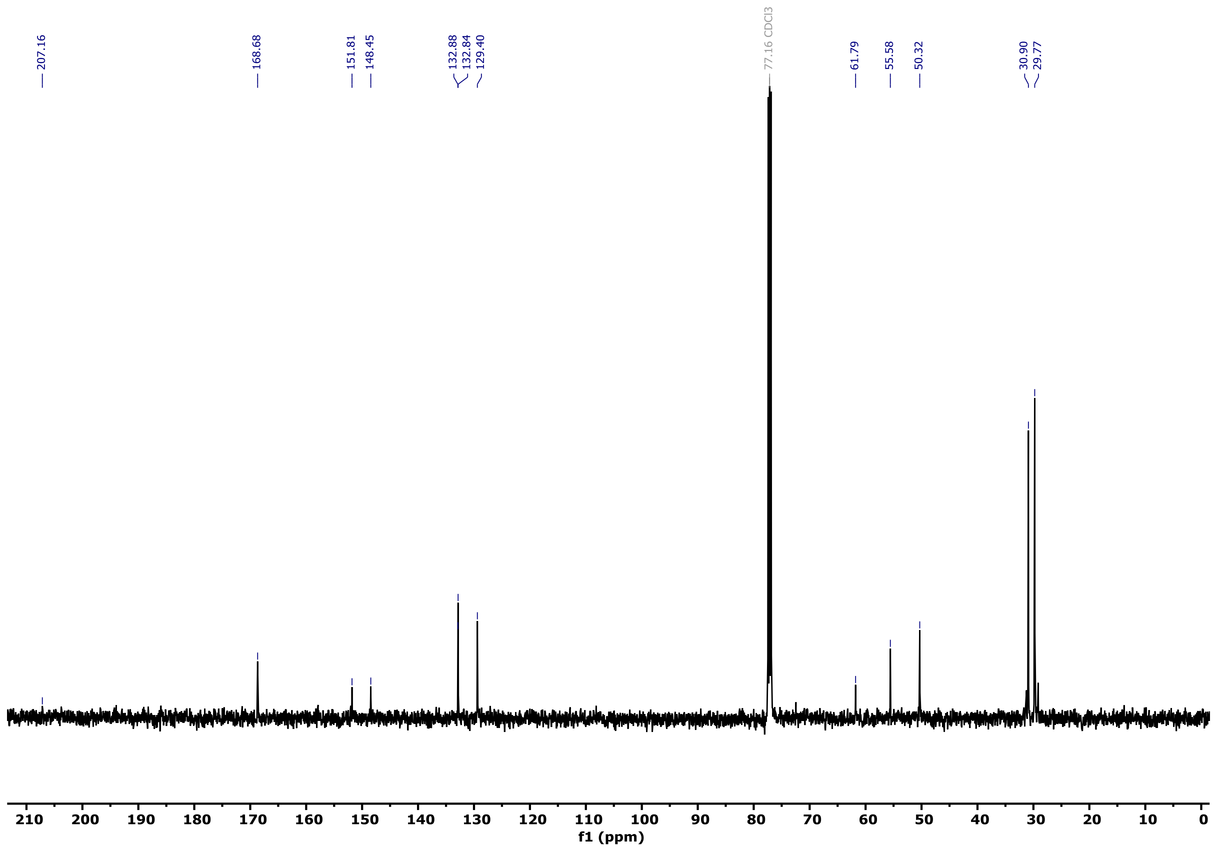

Figure S27. ^{13}C NMR (126 MHz) of Dichlorobismuthine [(L^3)$BiCl_2$] (**3**) in $CDCl_3$ at 298 K.

D) Synthesis of Dichlorobismuthine [(L^4)BiCl$_2$] (4)

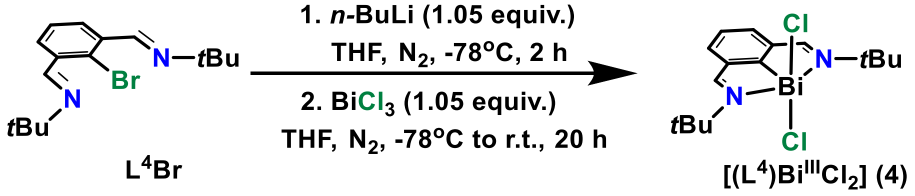

Scheme S9. Synthesis of Dichlorobismuthine [(L^4)BiCl$_2$] **(4)**

An oven dried 100 mL two-necked round-bottomed flask equipped with a magnetic stir bar and was charged with previously prepared ligand (L^1) (2 g, 6.18 mmol, 1 equiv.) in 60 mL dry tetrahydrofuran (THF) under N_2 and was cooled to −78 °C. Then *n*-BuLi (4.05 mL, 6.49 mmol, 1.05 equiv. 1.6 M in hexane) was added dropwise to the pre-cooled mixture. After complete addition, the solution turned into dark red color. The temperature was maintained at −78 °C for a period of 2 hours under stirring. After 2 hours a solution of $BiCl_3$ (2.04 g. 6.18 mmol, 1.05 equiv.) dissolved in dry THF (around 15 mL) was added to the precooled reaction mixture and stirred for 10 - 15 minutes before being brought to room temperature. The reaction mixture turned to a leaf-green color. Stirring continued at room temperature for another 20 hours. THF was removed in vacuum and the residue obtained was resuspended in 80 -100mL CH_2Cl_2. It was then filtered using a celite pad leaving orange-colored filtrate. The filtrate was then concentrated to 20 mL of CH_2Cl_2 and hexane (around 4/3 times the CH_2Cl_2 present) was added to it. A white precipitate was formed which was filtered with a filter-frit and washed with 200 mL of hexane to yield 2.1 g (65%) of Dichlorobismuthine [(L^4)BiCl$_2$] (**4**) as an off white solid.[3]

NMR of Dichlorobismuthine [(L^4)BiCl$_2$] (4)

^{1}H NMR (500 MHz, $CDCl_3$): δ 9.61 (s, 2H), 8.17 (d, *J* = 7.5 Hz, 2H), 7.85 (t, *J* = 7.6 Hz, 1H), 1.59 (s, 18H)

^{13}C NMR (125 MHz, $CDCl_3$): δ 192, 154.99, 136.22, 130.42, 127.76, 58.27, 29.83.

HR-MS (ESI$^+$) (4: $C_{16}H_{23}BiCl_2N_2$, M.W = 523.2544**)** calculated for $[4{-}Cl]^+$ 487.1354; found 487.1418

IR: 2970 (s), 2877 (m), 1621 (s), 1465 (m), 1236 (s), 753 (s) cm^{-1}

UV-Visible Spectroscopy: Complex **[(L[4])BiCl$_2$] (4)** (1.6 x 10^{-5} M) in CH_2Cl_2 shows λ_{max} 248 nm (ε_{248} = 19875 M^{-1} cm^{-1}), 284 nm (ε_{284} = 5750 M^{-1} cm^{-1}), 320 nm (ε_{320} = 2250 M^{-1} cm^{-1}) measured at 298 K in quartz cuvette with path length 1 cm.

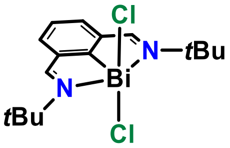

[(L[4])Bi^{III}Cl$_2$] (4)

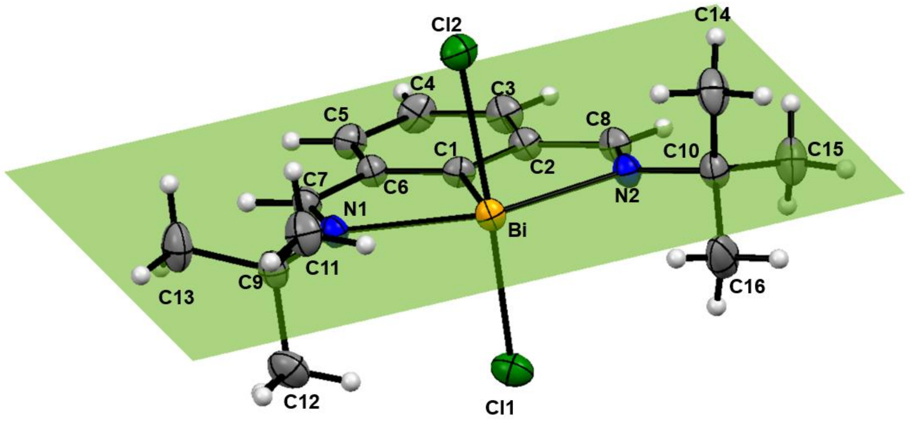

Figure S28: Structure showing symmetric and planar structure of catalyst **[(L[4])BiCl$_2$] (4)**

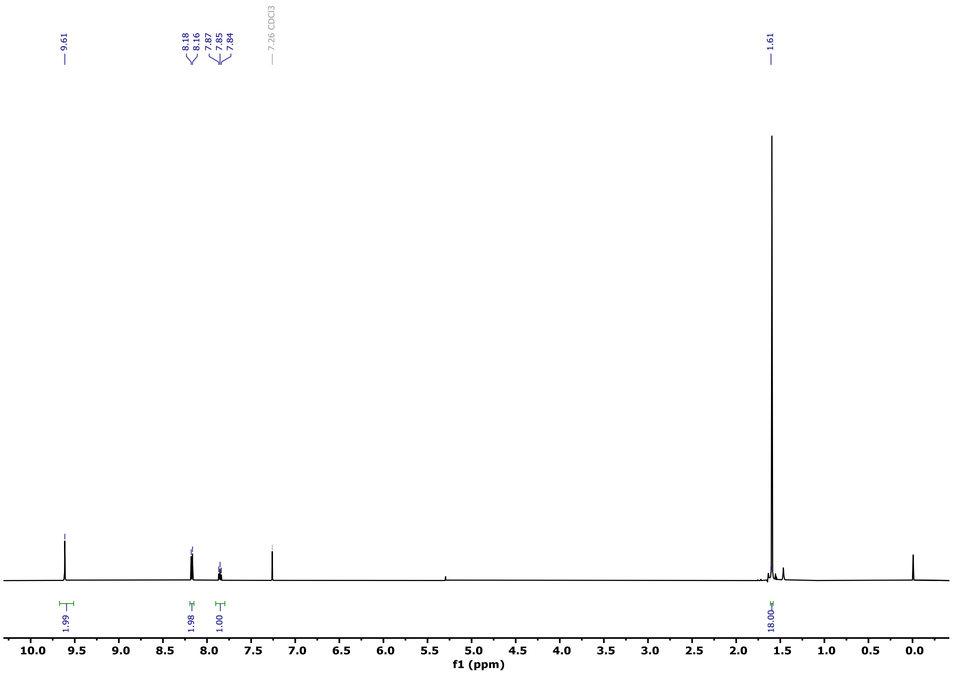

Figure S29. ^{1}H NMR (500 MHz) of Dichlorobismuthine [(L[4])BiCl$_2$] (**4**) in $CDCl_3$ at 298 K.

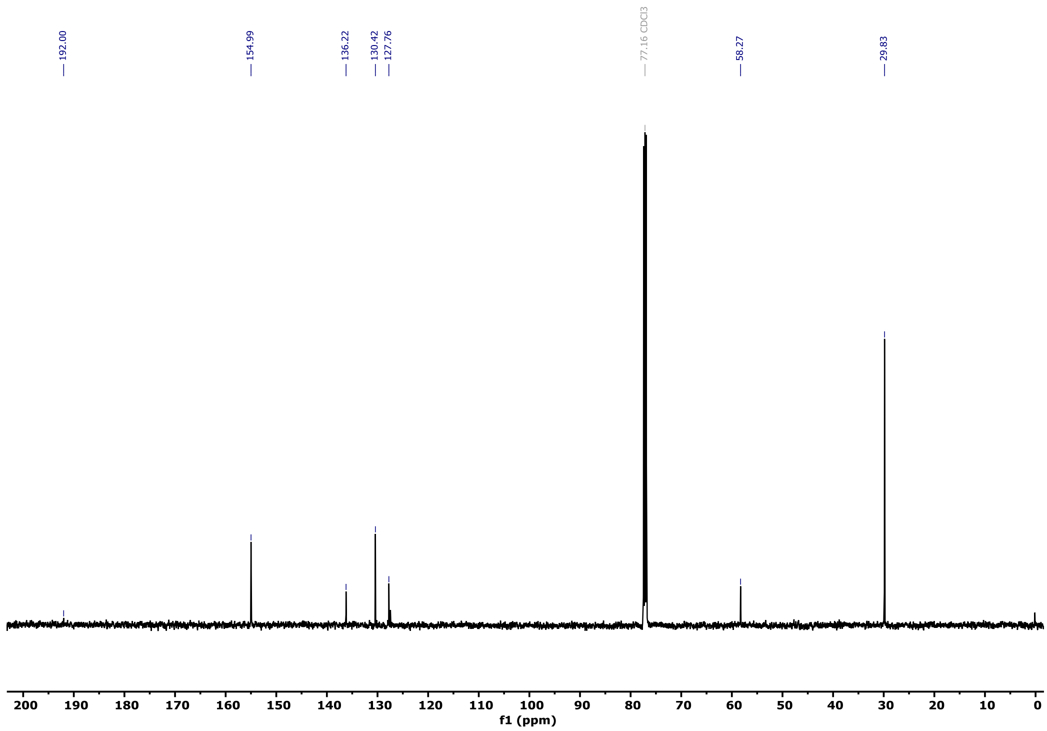

Figure S30. ^{13}C NMR (126 MHz) of Dichlorobismuthine [(L^4)$BiCl_2$] (**4**) in $CDCl_3$ at 298 K.

E) Synthesis of Dichlorobismuthine [(L^5)$BiCl_2$]

Step 1: Synthesis of L^5Br

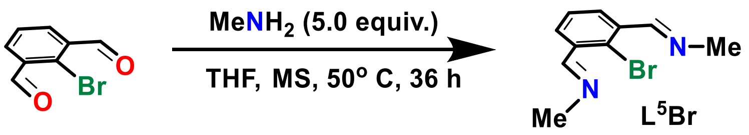

Scheme S10. Synthesis of (1E,1’E)-1,1’-(2-Bromo-1,3-phenylene) bis(N-methyl methanamine) (L^5Br)

To a 100 mL round-bottomed flask equipped with a magnetic stir bar and molecular sieves, 2-bromoisopthalaldehyde (1 g, 4.6 mmol, 1 equiv.) was added. After this, 15 - 20 mL of dry THF and methyl amine (1.1 mL, 23 mmol, 5 equiv.) were added. The dark yellow mixture was refluxed at 50 °C for 36 hours. The contents were passed through a filter-frit with a Celite Pad and washed with THF. The filtrate was collected and was evaporated under vacuum (and refrigeration for 1 day). Subsequent refrigeration for one day yielded 1.09 g (96%) of the required bisimine (L^5Br) as a yellow solid.

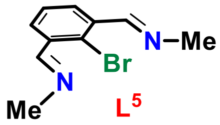

NMR of the L^5Br

^{1}H NMR (500 MHz, $CDCl_3$): δ 8.72 (s, 2H), 7.98 (d, J = 7.6 Hz, 2H), 7.34 (t, J = 7.6 Hz, 1H), 3.55 (s, 6H)

^{13}C NMR (125 MHz, $CDCl_3$): δ 161.48, 135.42, 130.65, 127.68, 126.80, 48.49

HR-MS (**L^5:** $C_{10}H_{11}BrN_2$, M.W = 239.1160) calculated for [**L^5**+H]$^+$ 239.0184; found 239.0186

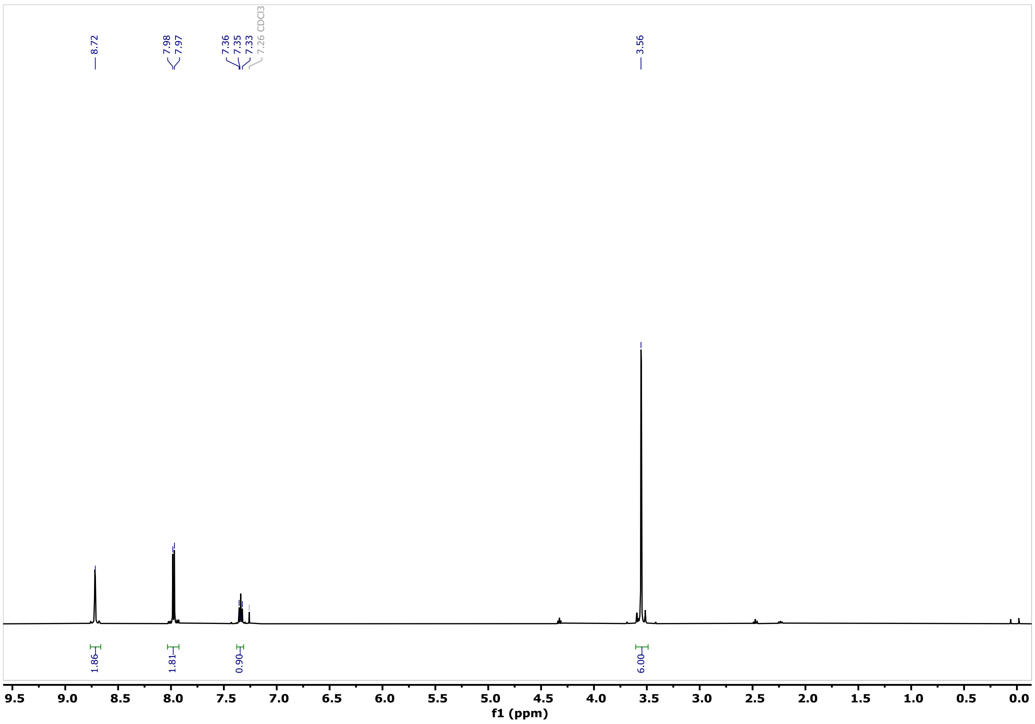

Figure S31. ^{1}H NMR (500 MHz) of ($L^{5}Br$) in $CDCl_3$ at 298 K.

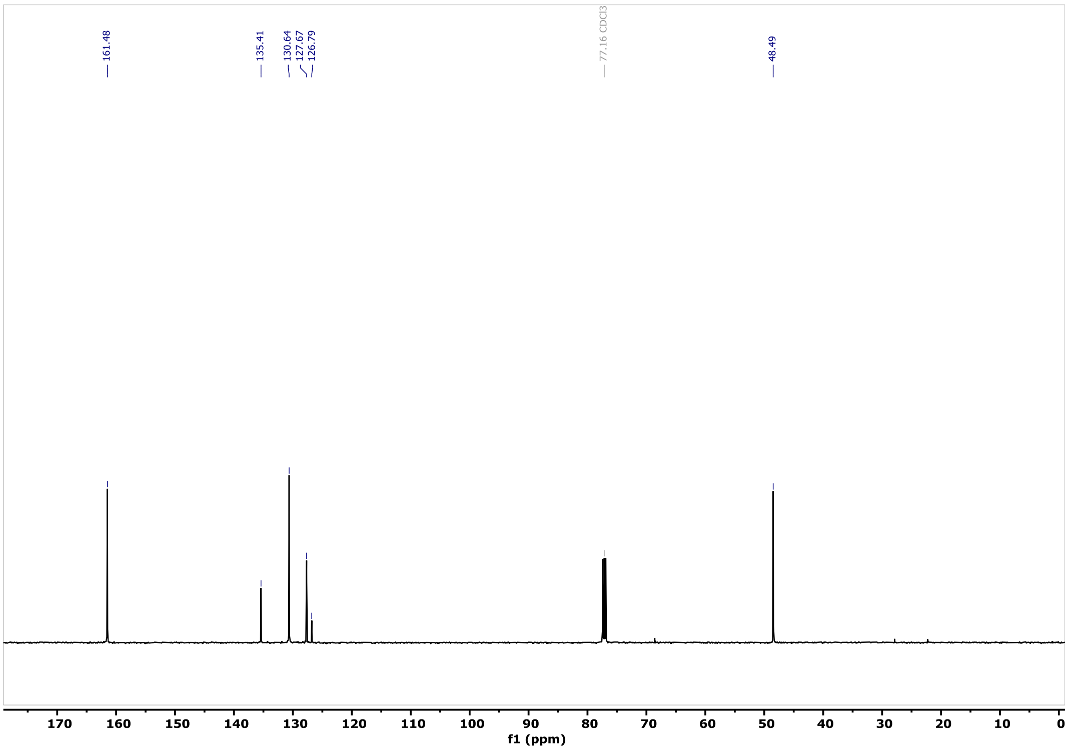

Figure S32. ^{13}C NMR (126 MHz) of ($L^{5}Br$) in $CDCl_3$ at 298 K.

Step 2: Synthesis of Dichlorobismuthine [(L^5)$BiCl_2$]

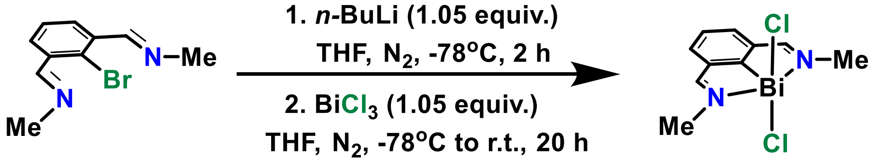

L5Br

[(L5)BiCl2] (5)

Scheme S11. Synthesis of Dichlorobismuthine [(L^5)$BiCl_2$] **(5)**

An oven dried 100 mL two-necked round-bottomed flask equipped with a magnetic stir bar was charged with previously prepared ligand (L^5Br) (0.476 g, 2 mmol, 1 equiv.) in 25 mL dry THF under N_2 and was cooled to −78 °C. Then *n*-BuLi (1.3 mL, 2.1 mmol, 1.05 equiv., 1.6 M in hexane) was added dropwise to the pre-cooled mixture. After complete addition, the solution turned into permanent dark red. The temperature was maintained at −78 °C for a period of 2 hours under stirring. After 2 hours a solution of $BiCl_3$ (0.662 g, 1 mmol, 1.05 equiv.) dissolved in dry THF (around 15 mL) was added to the precooled reaction mixture and stirred for 10 - 15 minutes before being brought to room temperature. The reaction mixture turned to a leaf-green color. Stirring continued at room temperature for another 20 hours. THF was removed in vacuum and the residue obtained was resuspended in 40 - 50 mL CH_2Cl_2. It was then filtered using a celite pad leaving orange-colored filtrate. The filtrate was then concentrated to 15 mL of CH_2Cl_2 and hexane (20 mL) was added to it. A white precipitate was formed which was filtered with a filter-frit and washed with 200 mL of hexane to yield 39.5 mg (4.5 %) of Dichlorobismuthine [(L^5)$BiCl_2$] **(5)** as light yellow solid.

NMR of Dichlorobismuthine [(L^5)$BiCl_2$] (5)

^{1}H NMR (500 MHz, $CDCl_3$): δ 9.54 (s, 2H), 8.18 (d, *J* = 7.5 Hz, 2H), 7.85 (t, *J* = 7.6 Hz, 1H), 3.77 (s, 6H)

^{13}C NMR (125 MHz, $CDCl_3$): δ 195.54, 173.86, 147.29, 136.02, 129.88, 46.09.

HR-MS (ESI$^+$) (5: $C_{10}H_{11}BiCl_2N_2$, M.W = 439.0924**)** calculated for **[5−Cl]**$^+$ 403.0415; found 403.0415.

IR: 2962 (s), 2865 (w), 1632 (m), 1568 (m),1456 (s),1163 (m), 792 (vs) cm^{-1}

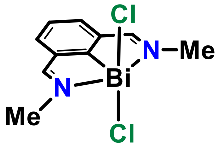

[(L⁵)BiCl₂] (5)

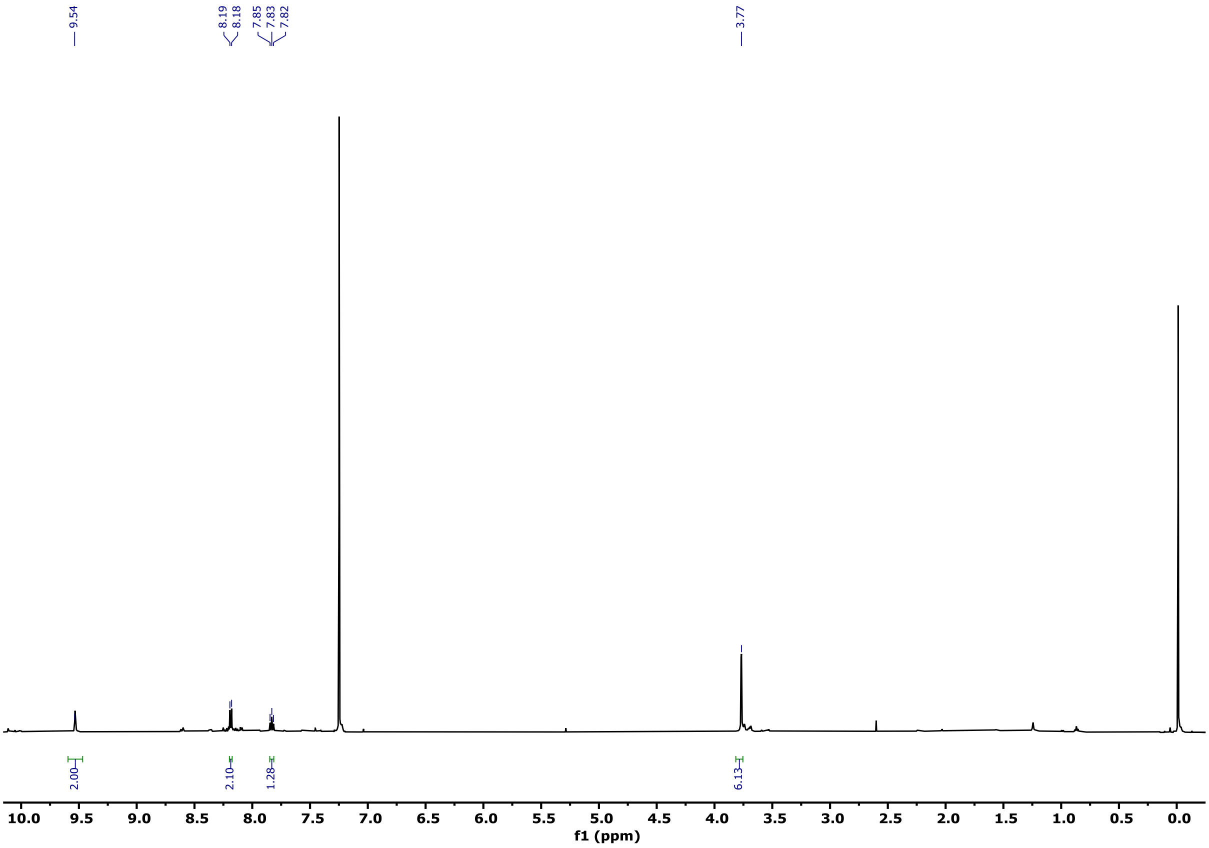

Figure S33. ^{1}H NMR (500 MHz) of Dichlorobismuthine [(L**⁵**)BiCl₂] **(5)** in $CDCl_3$ at 298 K.

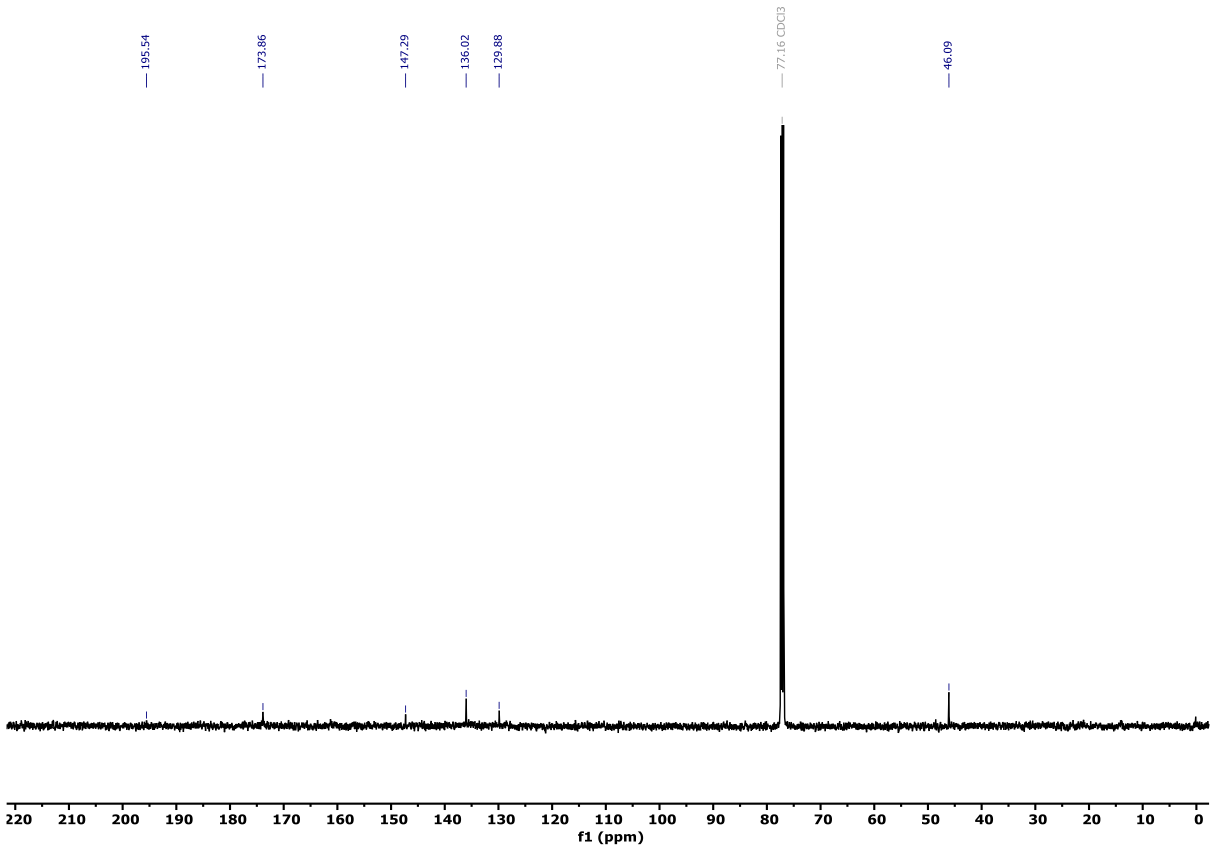

Figure S34. ^{13}C NMR (126 MHz) of Dichlorobismuthine [(L^5)$BiCl_2$] **(5)** in $CDCl_3$ at 298 K.

F) Synthesis of Dichlorobismuthine [(L^6)$BiCl_2$] (6)

Step 1: Synthesis of L^6Br

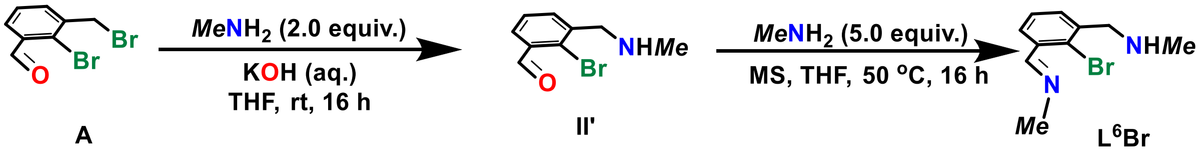

Scheme S12. Synthesis of (L^6Br)

A 25 mL round-bottomed flask equipped with a magnetic stir bar was charged with **A** (0.5 g, 1.8 mmol, 1.0 equiv.), obtained from the previous step and methylamine (*Me*NH_2 40% in MeOH, 9.8 mol/L) (0.367 mL, 3.6 mmol, 2.0 equiv.), and tetrahydrofuran (THF) (20 mL). An aqueous KOH solution (10 M, 2 mL) was then added, and the mixture was stirred overnight at ambient temperature. The organic phase was separated, dried over Na_2SO_4, and concentrated to afford a light-yellow solid product. This product (II') was used in the next step without further purification.

A 100 mL two-necked round-bottomed flask with a stir bar was charged with the product (II') obtained in step 2, methylamine (*Me*NH_2 40% in MeOH, 9.8 mol/L) (0.918 mL, 18 mmol, 5.0 equiv.), Molecular Sieves (4 Å) and 12 mL dry tetrahydrofuran (THF). The reaction was stirred for 16 hours at 50 °C and the completion of the reaction was monitored by NMR. The mixture was filtered through celite and eluted with dichloromethane and ethyl acetate. The filtrate was concentrated under reduced pressure to afford a 0.40 g yellow semi-viscous oil L^6Br (92% over two steps).[2]

NMR of L^6Br

^1H NMR (500 MHz, $CDCl_3$): δ 8.72 (s, 1H), 7.84 (dd, J = 7.8, 1.8 Hz, 1H), 7.41 (dd, J = 7.5, 2.0 Hz, 1H), 7.28 (t, J = 7.5 Hz, 1H), 3.86 (s, 2H), 33.55 (m, 1H), 2.45 (s, 1H).

^{13}C NMR (125 MHz, $CDCl_3$): δ 162, 139.63, 135.58, 132.16, 127.70, 127.47, 217.32, 55.96, 48.48, 36.07.

HR-MS (ESI$^+$) (L^6: $C_{10}H_{13}BrN_2$, M.W = 240.0262**)** calculated for [**L**^{6}+H]$^+$ 241.0340; found 241.0342.

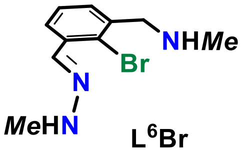

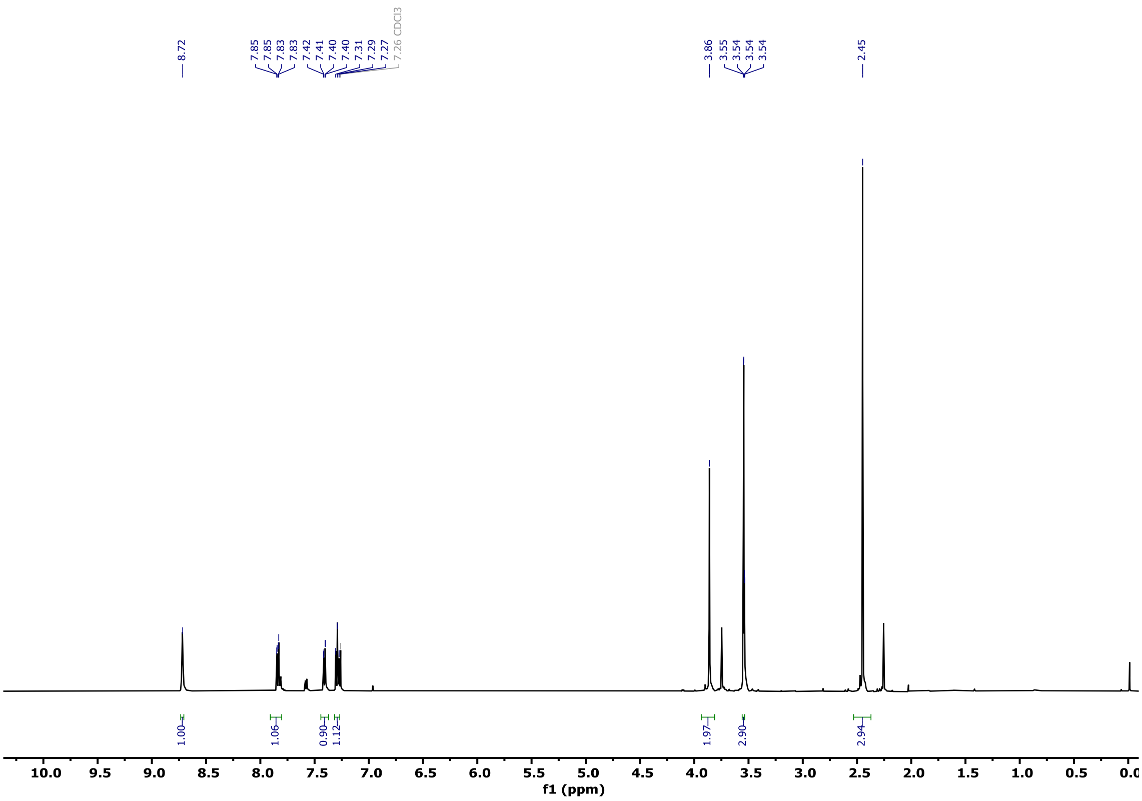

Figure S35. ^{1}H NMR (500 MHz) of ($L^{6}Br$) in $CDCl_3$ at 298 K.

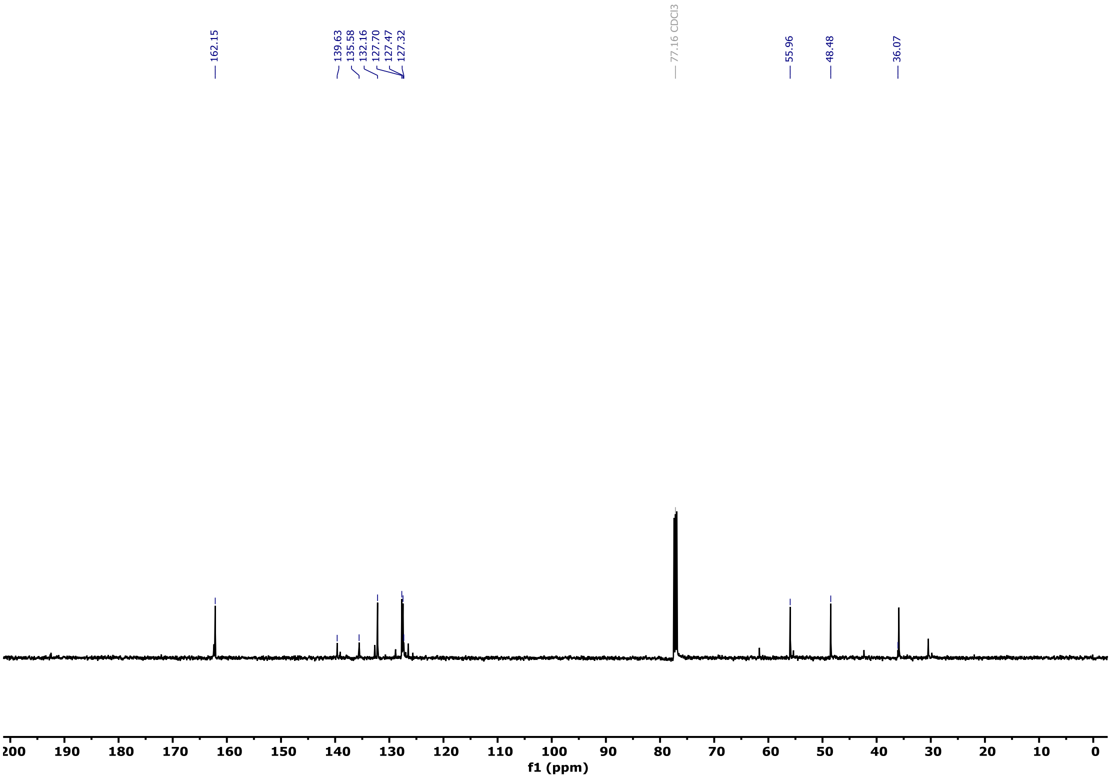

Figure S36. ^{13}C NMR (126 MHz) of ($L^{6}Br$) in $CDCl_3$ at 298 K.

Step 2 :- Synthesis of Dichlorobismuthine [(L[6])BiCl₂] (6)

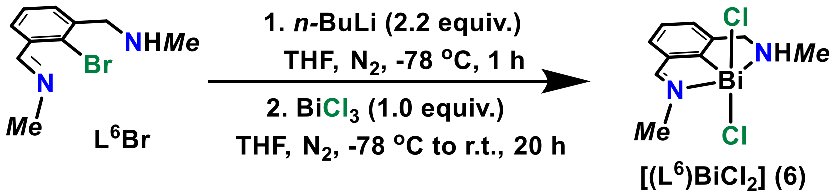

Scheme S13. Synthesis of Dichlorobismuthine [$(L^6)BiCl_2$] **(6)**

An oven dried 100 mL two-necked round-bottomed flask equipped with a magnetic stir bar was charged with previously prepared ligand (L^6Br) (0.400 g, 1.65 mmol, 1 equiv.) in 50 mL dry tetrahydrofuran (THF) under N_2 and was cooled to −78 °C. Then *n*-BuLi (1.81 mL, 3.63 mmol, 2.2 equiv., 2 M in cyclohexane) was added dropwise to the pre-cooled mixture. After complete addition, the solution turned into dark red color. The temperature was maintained at −78 °C for a period of 2 hours under stirring. After 2 hours a solution of $BiCl_3$ (0.52 g, 1.65 mmol, 1.0 equiv.) dissolved in dry tetrahydrofuran (THF) (around 10 mL) was added to the pre-cooled reaction mixture and stirred for 10 - 15 minutes before being brought to room temperature. The resulting brown mixture was stirred and allowed to reach at ambient temperature overnight. The following workup was carried out under air. All volatiles were removed under vacuum. The resulting solids were redissolved in dichloromethane and filtered through celite. The filtrate was concentrated under vacuum to 5 mL, and hexane was used to precipitate the product which was filtered with a filter-frit and washed with 100 mL of hexane to yield (0.2 g, 51%) of Dichlorobismuthine [$(L^6)BiCl_2$] (**6**) as light yellow solid.[2]

NMR of Dichlorobismuthine [$(L^6)BiCl_2$] (6)

^{1}H NMR (500 MHz, $CDCl_3$): δ 9.38 (q, J = 1.6 Hz, 1H), 7.95 (dd, J = 7.5, 5.0 Hz, 2H), 7.69 (t, J = 7.5 Hz, 1H), 5.35 (dd, J = 14.9, 5.7 Hz, 1H), 4.29 (dd, J = 14.8, 10.5 Hz, 1H), 3.72 (s, 3H), 3.13 (d, J = 6.2 Hz, 3H).

^{13}C NMR (125 MHz, $CDCl_3$): δ 206.44, 161.83, 152.07, 132.62, 132.32, 129.63, 59.31, 45.99, 37.07.

Note : Due to the high quadrupole moment of the ^{209}Bi nucleus (100%, I = 9/2, quadrupole moment −0.4 x 10^{-28} m^{-2}), which broaden the peaks corresponding to atoms bonded to the Bi center to such an extent that they are not observable under standard conditions, so here carbon corresponding to the C-Bi bond is also not observable prominently by ^{13}C NMR spectroscopy. (See Figure S12)[4]

HR-MS (ESI$^+$) (6: $C_{10}H_{13}BiCl_2N_2$, M.W = 441.1084**)** calculated for [**6**−Cl]$^+$ 405.0571; found 405.0573

UV-Visible Spectroscopy: Complex **[(L[6])BiCl$_2$] (6)** (1.6 x 10^{-5} M) in CH_2Cl_2 shows λ_{max} 247 nm (ε_{247} = 18750 M^{-1} cm^{-1}), 300 nm (ε_{300}= 7500 M^{-1} cm^{-1}), measured at 298 K in quartz cuvette with path length 1 cm.

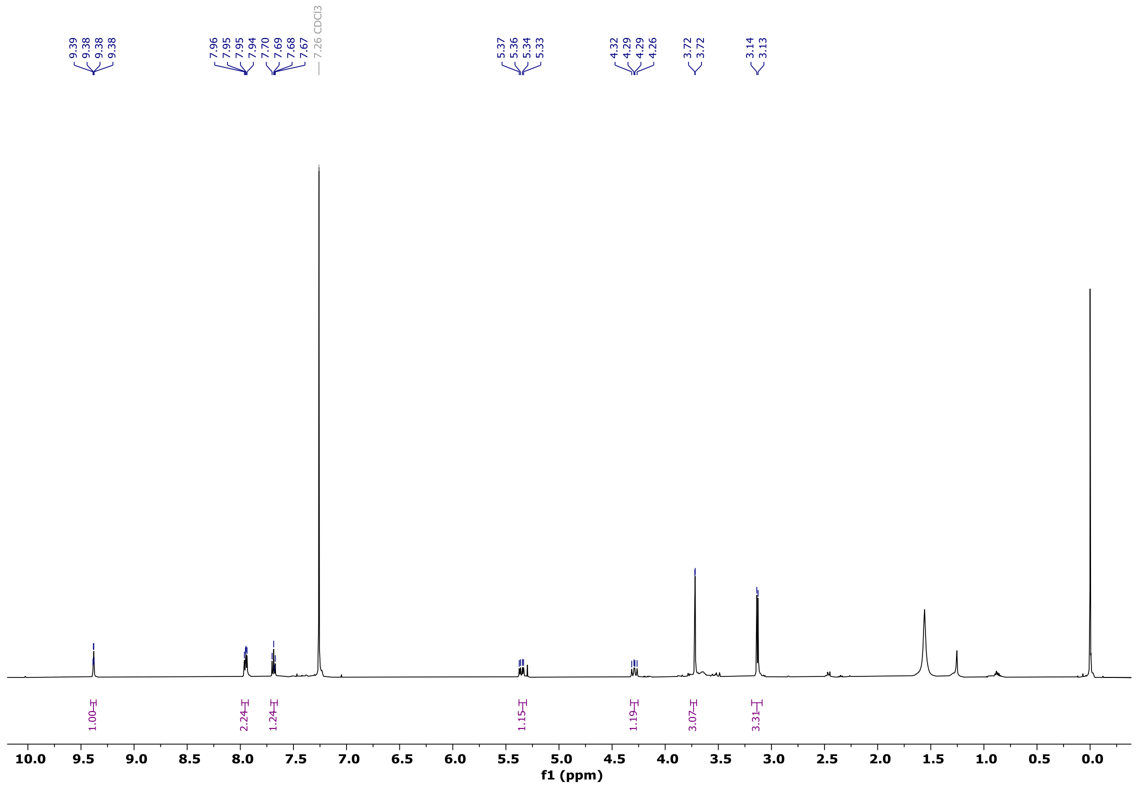

Figure S37. ^1H NMR (500 MHz) of Dichlorobismuthine [(L[6])BiCl$_2$] (**6**) in $CDCl_3$ at 298 K.

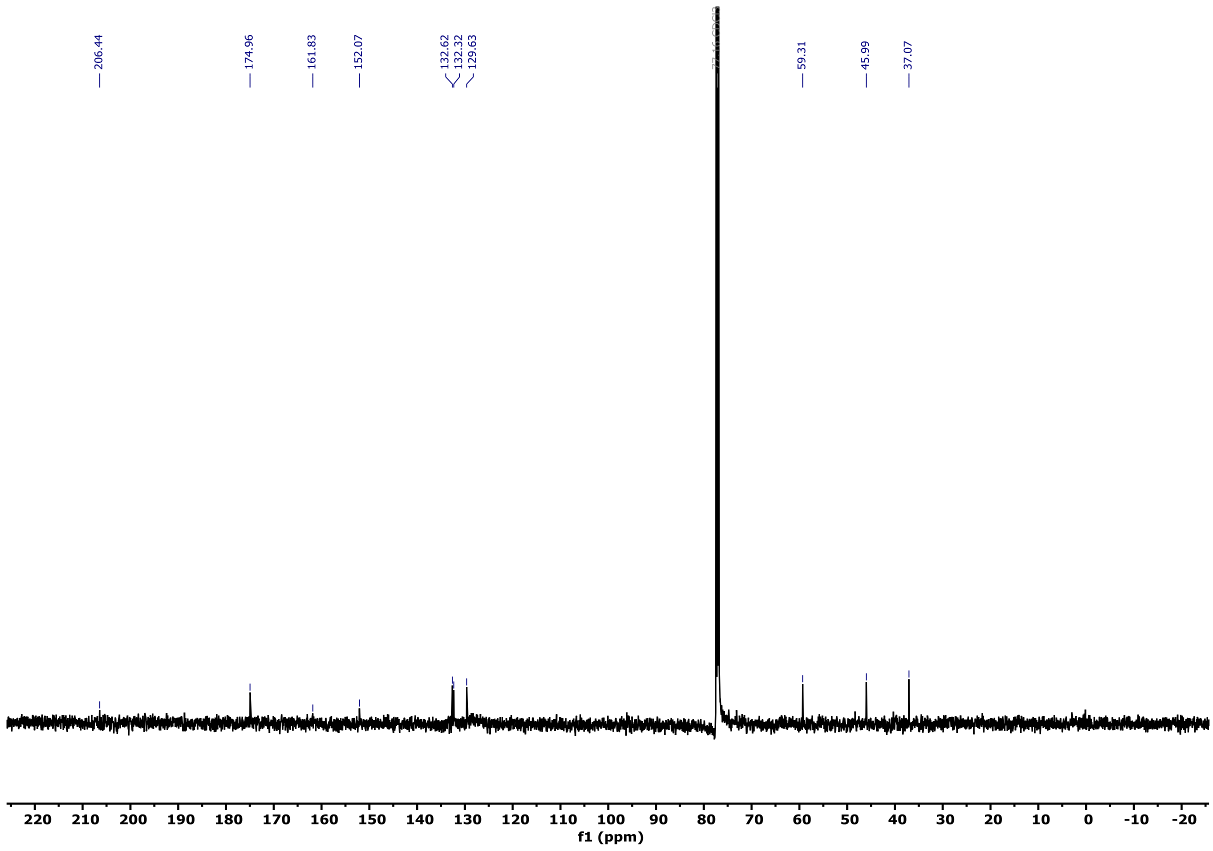

Figure S38. ^{13}C NMR (126 MHz) of Dichlorobismuthine [(L^6)$BiCl_2$] (**6**) in $CDCl_3$ at 298 K.

G) Synthesis of Dichlorobismuthine [(L^7)BiCl$_2$] (7)

Step 1: Synthesis of Ligand L^{7}Br

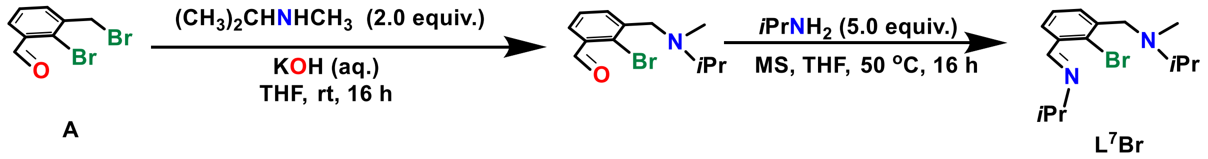

Scheme S14. Synthesis of Dichlorobismuthine [(L^7)BiCl$_2$] **(7)**

A 25 mL round-bottomed flask equipped with a magnetic stir bar was charged with A (500 mg, 2 mmol, 1.0 equiv.), $(CH_3)_2CHNHCH_3$ (0.42 mL, 4 mmol, 2.0 equiv.), and THF (5 mL). An aqueous KOH solution (10 M, 2 mL) was then added, and the mixture was stirred overnight at ambient temperature. The organic phase was separated, dried over Na_2SO_4, and concentrated to afford a yellow white solid product. This product was used in the next step without further purification.

A 100 mL two-necked round-bottomed flask with a stir bar was charged with product obtained in step 3, *i*PrNH$_2$ (1.1 mL, 10 mmol, 5.0 equiv.), MS (4 Å) and 10 mL dry THF (first distilled) under N_2. The reaction was stirred for 16 hours at 50 °C and the completion of the reaction was monitored by NMR. The mixture was filtered through celite and eluted with DCM and ethyl acetate. The filtrate was concentrated under reduced pressure to afford a 550 mg yellow semi-viscous oil.

NMR of L^{7}Br

^{1}H NMR (500 MHz, $CDCl_3$): δ 8.74 (s, 1H), 7.84 (d, J = 7.6 Hz, 1H), 7.55 (d, J = 8.3 Hz, 1H), 7.29 (t, J = 7.6 Hz, 1H), 3.60 (s, 2H), 3.58 (m, 1H), 2.95 (s, J = 13.1 Hz, 3H), 1.27 (d, J = 6.3 Hz, 2H), 1.10 (d, J = 6.3 Hz, 2H).

^{13}C NMR (125 MHz, $CDCl_3$): δ 158.51, 139.87, 135.64,132.52, 127.67, 127.25, 126.89, 61.70, 57.63, 54.01, 36.64, 24.28, 18.14.

HR-MS (ESI$^+$) (L^7: $C_{15}H_{23}BrN_2$, M.W = 311.2670**)** calculated for **[L^7+H]$^+$** 311.1123; found 311.1125.

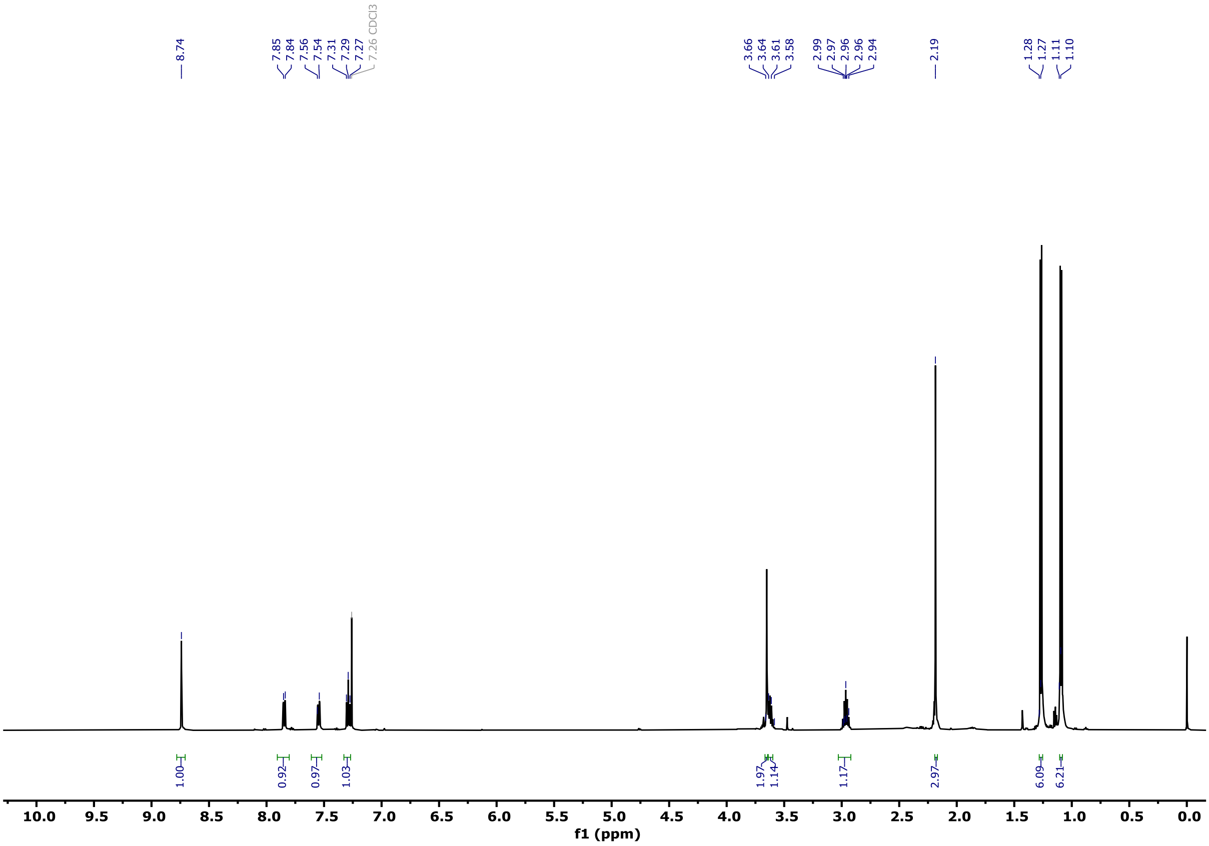

Figure S39. ^{1}H NMR (500 MHz) of (L^7Br) in $CDCl_3$ at 298 K.

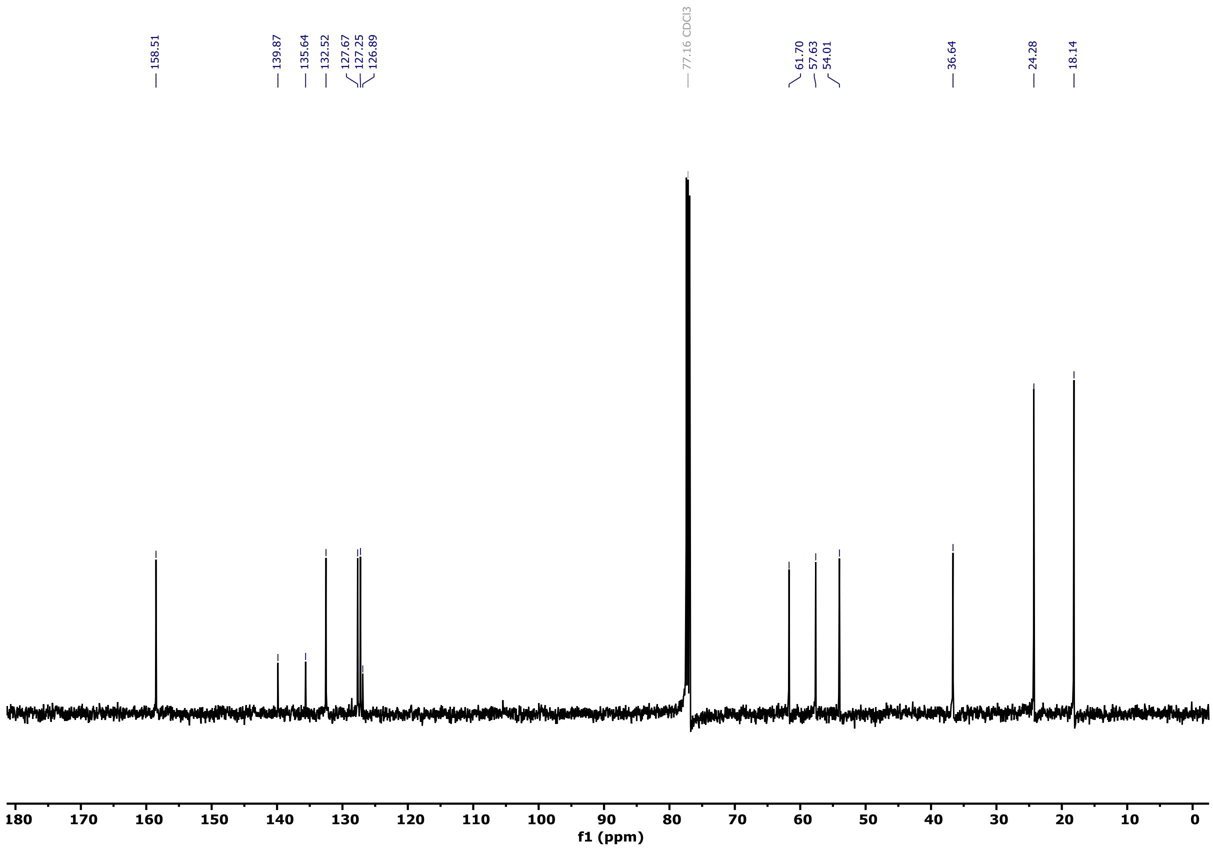

Figure S40. ^{13}C NMR (126 MHz) of (L^7Br) in $CDCl_3$ at 298 K.

E) Synthesis of Dichlorobismuthine [(L[7])$BiCl_2$] (7)

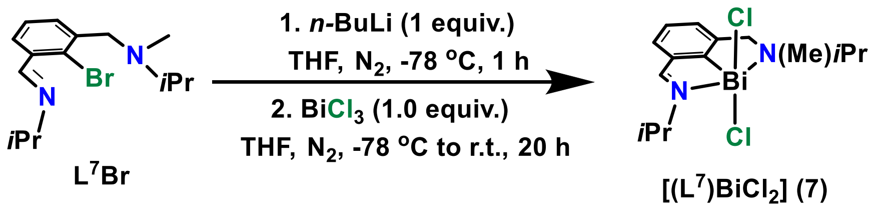

Scheme S15. Synthesis of Dichlorobismuthine [(L^7)$BiCl_2$] **(7)**

An oven dried 100 mL two-necked round-bottomed flask equipped with a magnetic stir bar was charged with previously prepared ligand (L^7Br) (0.5 g, 1.6 mmol, 1 equiv.) in 50 mL dry tetrahydrofuran (THF) under N_2 and was cooled to −78 °C. Then *n*-BuLi (0.8 mL, 1.6 mmol, 1 equiv., 2 M in cyclohexane) was added dropwise to the pre-cooled mixture. After complete addition, the solution turned into dark red color. The temperature was maintained at −78 °C for a period of 2 hours under stirring. After 2 hours a solution of $BiCl_3$ (0.505 g, 1.6 mmol, 1.0 equiv.) dissolved in dry tetrahydrofuran (THF) (around 10 mL) was added to the pre-cooled reaction mixture and stirred for 10 - 15 minutes before being brought to room temperature. The resulting brown mixture was stirred and allowed to reach at ambient temperature overnight. The following workup was carried out under air. All volatiles were removed under vacuum. The resulting solids were redissolved in dichloromethane and filtered through celite. The filtrate was concentrated under vacuum to 5 mL, and hexane was used to precipitate the product which was filtered with a filter-frit and washed with 100 mL of hexane to yield (0.2 g, 21%) of Dichlorobismuthine [(L^7)$BiCl_2$] (**7**) as yellow solid.

NMR of Dichlorobismuthine [(L^7)$BiCl_2$] (7)

^{1}H NMR (500 MHz, $CDCl_3$): δ 9.46 (s, 1H), 7.91 (t, *J* = 7.4 Hz, 2H), 7.67 (t, *J* = 7.5 Hz, 1H), 5.37 (dd, *J* = 14.7, 5.4 Hz, 1H), 4.26 – 4.16 (m, 1H), 3.96 – 3.91 (m, 1H), 3.62 – 3.55 (m, 1H), 1.53 – 1.47 (m, 12H), 1.37 (s, 3H).

^{13}C NMR (125 MHz, CD_3CN): δ 196.36, 170.41, 151.75, 146.90, 132.44, 132.14, 129.27, 60.52, 54.56, 51.45, 24.80, 21.92.

HR-MS (ESI$^+$) (7: $C_{15}H_{23}BiCl_2N_2$, M.W = 511.2434**)** calculated for [**7**−Cl]$^+$ 475.1354; found 475.1353.

UV-Visible Spectroscopy: Complex **[(L^7)$BiCl_2$] (7)** (1.6 x 10^{-5} M) in CH_2Cl_2 shows λ_{max} 247 nm (ε_{247} = 22937 M^{-1} cm^{-1}), 285 nm (ε_{285}= 1875 M^{-1} cm^{-1}), measured at 298 K in quartz cuvette with path length 1 cm.

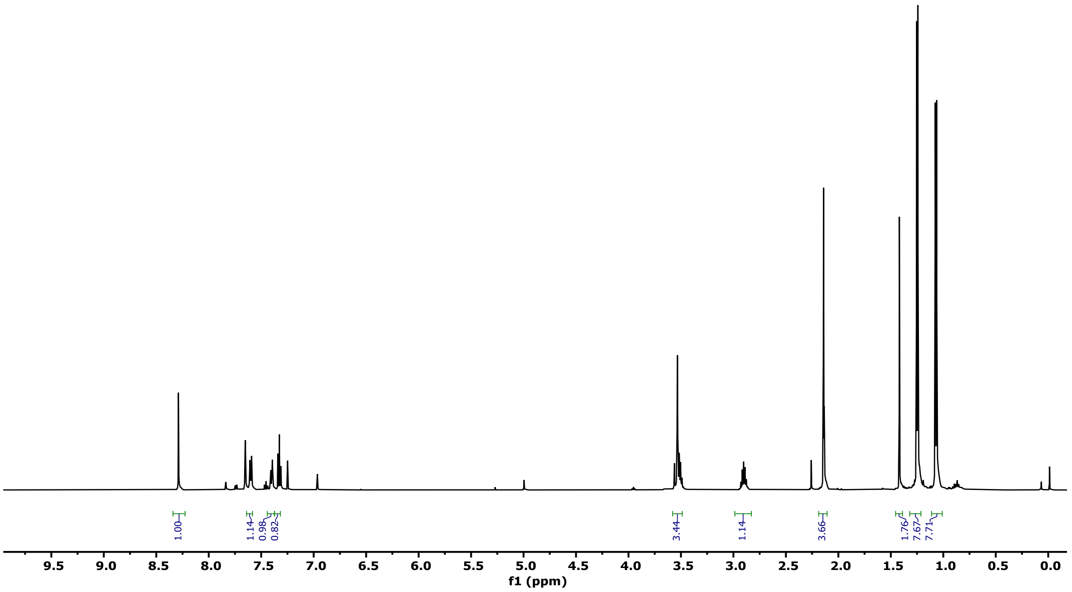

Figure S41. ^{1}H NMR (500 MHz) of Dichlorobismuthine [(L^{7})$BiCl_2$] (**7**) in $CDCl_3$ at 298 K.

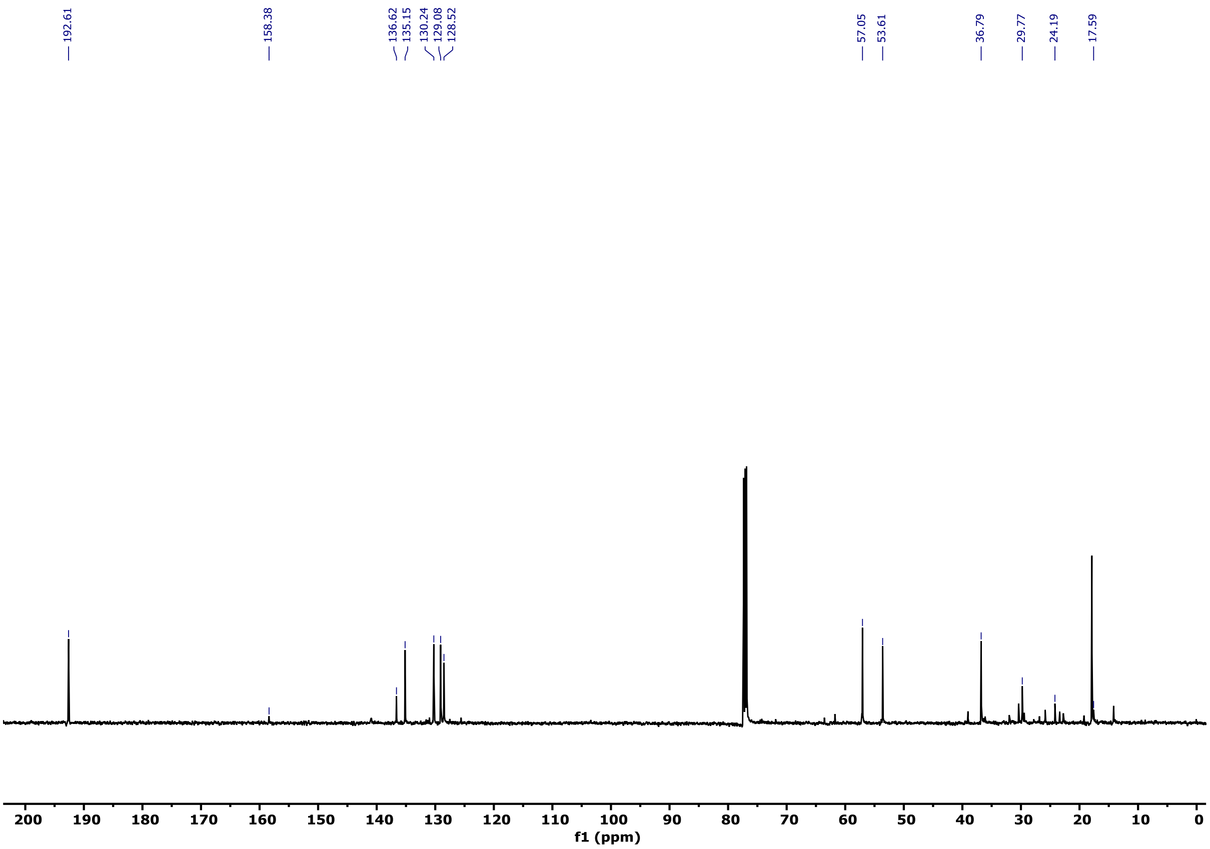

Figure S42. ^{13}C NMR (126 MHz) of Dichlorobismuthine [(L^{7})$BiCl_2$] (**7**) in $CDCl_3$ at 298 K.

Synthesis of Aluminium NCN pincer complex [(L^2)$AlCl_2$] (8)

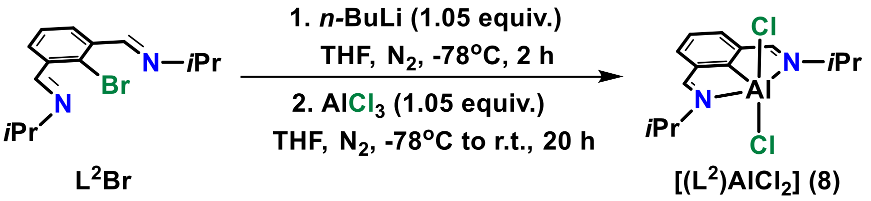

Scheme S16. Synthesis of Aluminium pincer complex [(L^2)$AlCl_2$] (8)

An oven dried 100 mL two-necked round-bottomed flask equipped with a magnetic stir bar was charged with previously prepared ligand (L^2) (1 g, 3.4 mmol, 1 equiv.) in 50 mL dry tetrahydrofuran (THF) under N_2 and was cooled to −78 °C. Then *n*-BuLi (2.25 mL, 3.5 mmol, 1.05 equiv., 1.6 M in hexane) was added dropwise to the pre-cooled mixture. After complete addition, the solution turned into dark red. The temperature was maintained at −78° C for a period of 30 min under stirring. After 30 hours a solution of $AlCl_3$ (476 g, 3.57 mmol, 1.05 equiv.) dissolved in dry tetrahydrofuran (THF) (around 15 mL) was added to the pre-cooled reaction mixture and stirred for 10 - 15 minutes before being brought to room temperature. The reaction mixture turned to a yellow color. Stirring continued at room temperature for another 20 hours. Tetrahydrofuran (THF) was removed in vacuum and the residue obtained was resuspended in 40 - 50 mL CH_2Cl_2. It was then filtered using a celite pad leaving off-white filtrate. The filtrate was then concentrated to 20 mL of dichloromethane and hexane was added to it. An off-white precipitate was formed which was filtered with a filter-frit and washed with 200 mL of hexane to yield (0.7 g, 65%) of Aluminium pincer complex [(L^2)$AlCl_2$] (**8**) as an off-white solid.

NMR of Dichlorobismuthine [(L^2)$AlCl_2$] (8)

^{1}H NMR (500 MHz, $CDCl_3$): δ 8.73 (s, 2H), 8.01 (s, 2H), 7.48 – 7.29 (m, 1H), 3.64 (p, J = 6.3 Hz, 2H), 1.28 (d, J = 6.3 Hz, 12H).

^{13}C NMR (125 MHz, $CDCl_3$): δ 191.82, 157.52, 135.70, 130.90, 127.65, 24.24.

HR-MS (ESI$^+$) (**8**: $C_{14}H_{19}AlCl_2N_2$, M.W = 313.2015) calculated for [**8**−Cl]$^+$ 277.1052; found 277.1054.

UV-Visible Spectroscopy: [(L^2)$AlCl_2$] (8) (5 x 10^{-4} M) in CH_2Cl_2 shows λ_{max} 300 nm (ε_{300} = 780 M^{-1} cm^{-1}), 320 nm (ε_{320}= 1340 M^{-1} cm^{-1}), measured at 298 K in quartz cuvette with path length 1 cm.

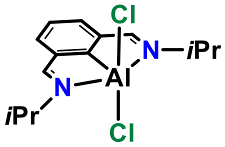

[(L^2)AlCl$_2$] (8)

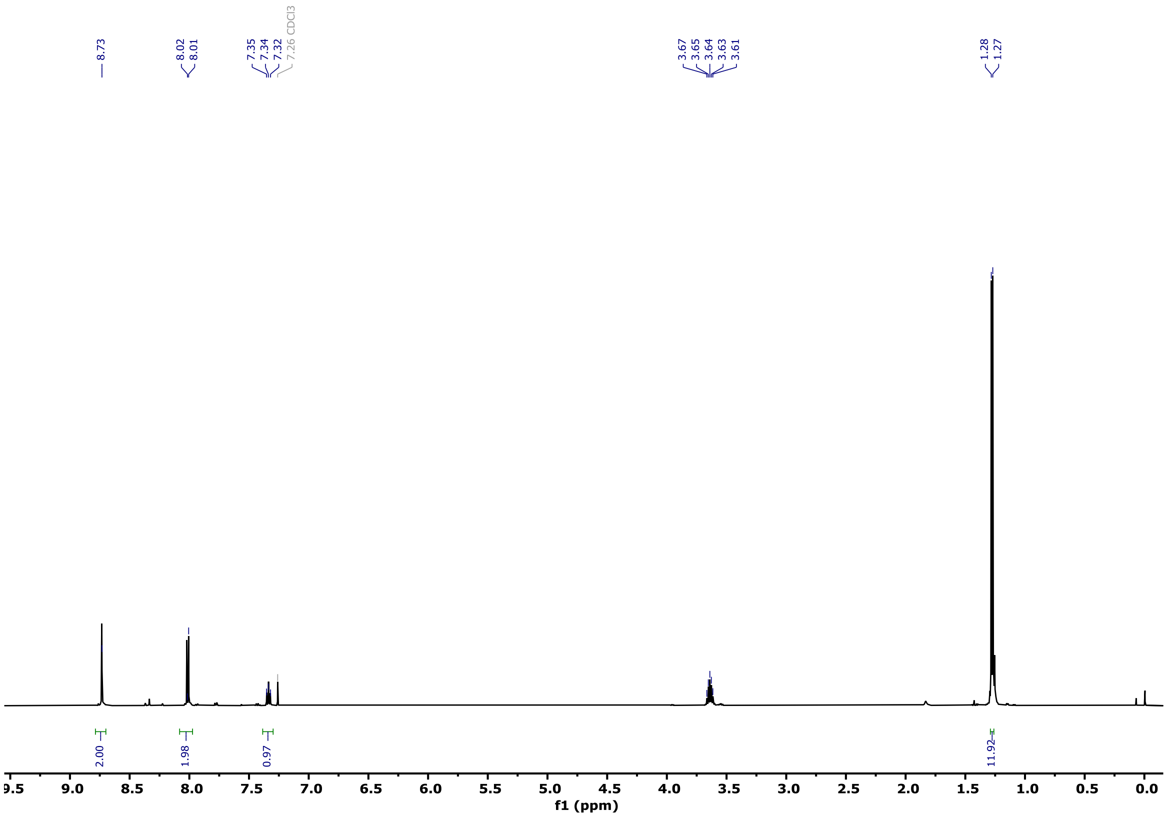

Figure S43. ^{1}H NMR (500 MHz) of Dichloroaluminium [(L^2)AlCl$_2$] (**8**) in $CDCl_3$ at 298 K.

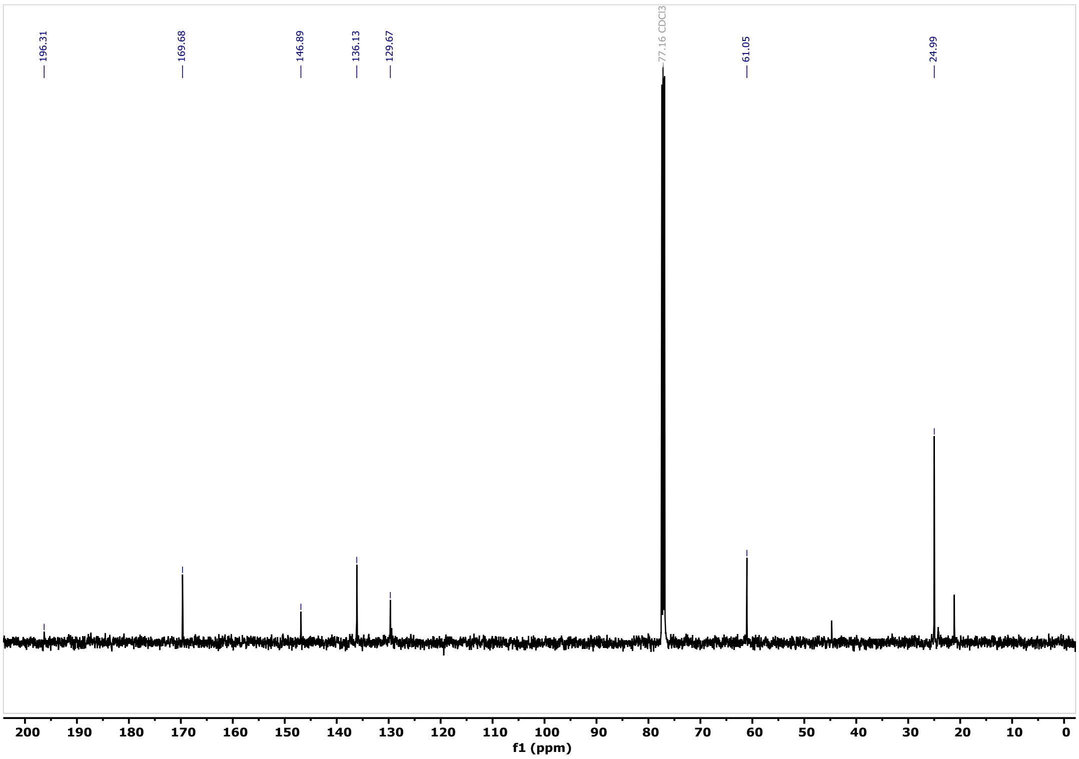

Figure S44. ^{13}C NMR (126 MHz) of Dichloroaluminium [(L^2)$AlCl_2$] (**8**) in $CDCl_3$ at 298 K

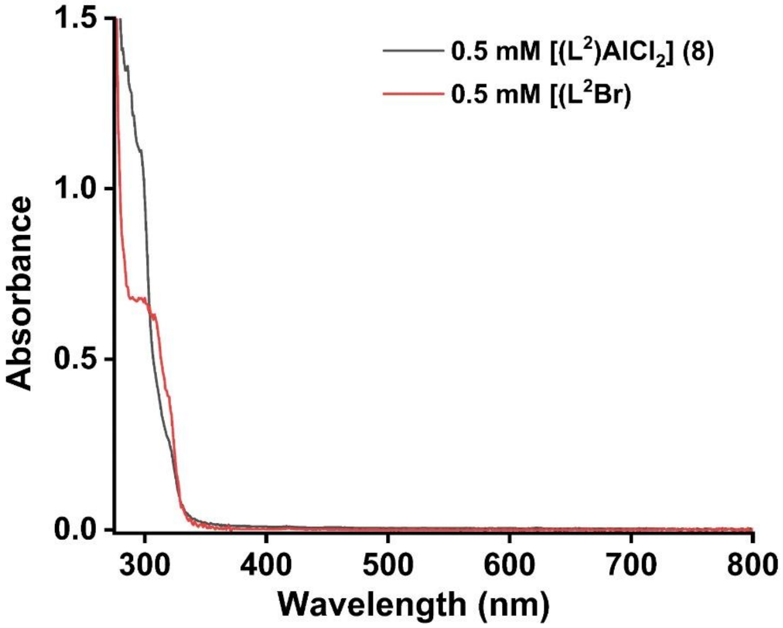

Figure S45. UV-Vis spectra of 0.5 mM of a) [(L^2)$AlCl_2$] (**8**) (black) and ligand L^2Br (red) in dichloromethane (path length = 1 cm, temperature 298 K)

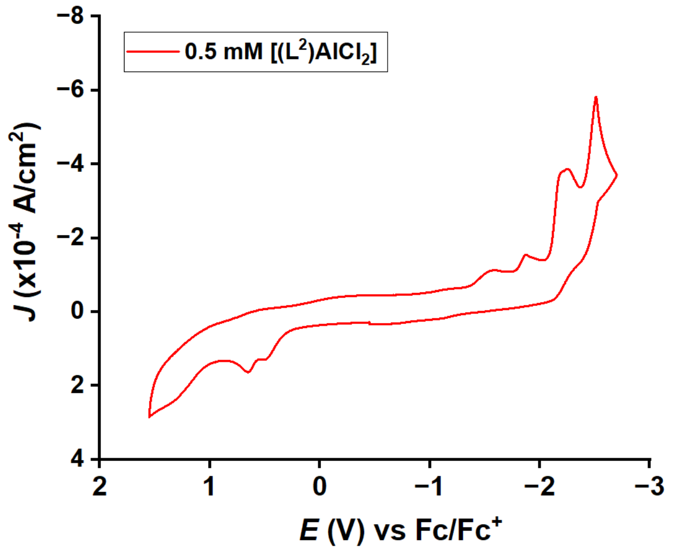

Figure S46. Cyclic Voltammogram of 0.5 mM [(L^2)AlCl$_2$] **(8)** with 0.1 M TBAPF$_6$ as a supporting electrolyte in acetonitrile. Condition: Working electrode glassy carbon (3 mm diameter), counter electrode Pt wire, and reference is Ag/AgCl. 100 mV/s scan rate. Data referenced to Fc/Fc$^+$ couple showing only ligand-based oxidation and reduction peaks.

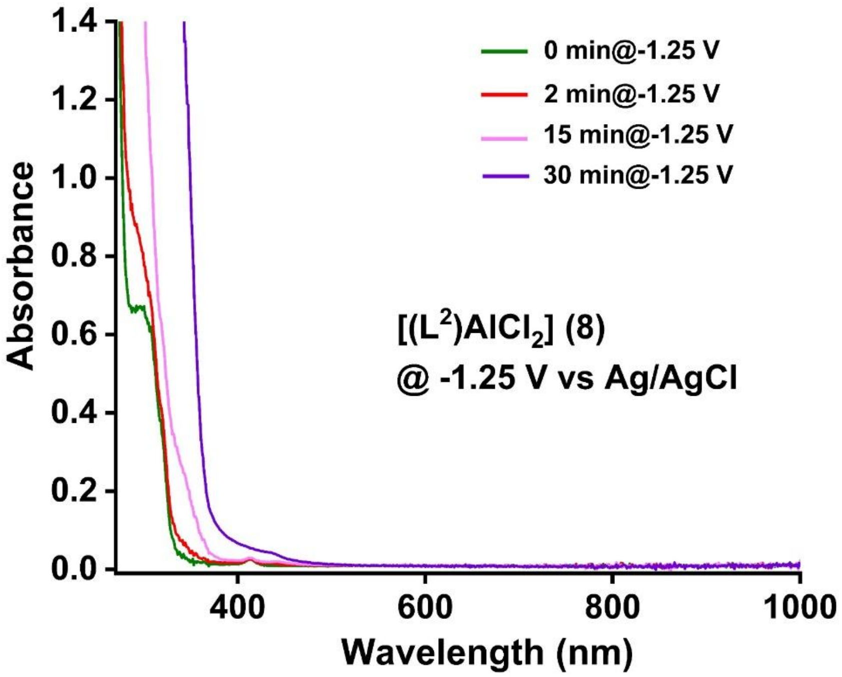

Figure S47. UV-Vis absorption spectrum for [(L^2)AlCl$_2$] **(8)** in acetonitrile under spectroelectrochemical condition with an applied potential of −1.25 V vs Ag/AgCl.

UV-Vis Spectroscopy of all Metal Catalyst [(L^1)BiCl$_2$] **(1) -** [(L^6)BiCl$_2$] **(6) (II)**

UV-Vis spectroscopic measurements were taken at room temperature (298 K) in quartz cuvette with path length = 1 cm. CH_2Cl_2 was used as the solvent, and the concentration of the bismuth complexes was 1.6 x 10^{-5} M.

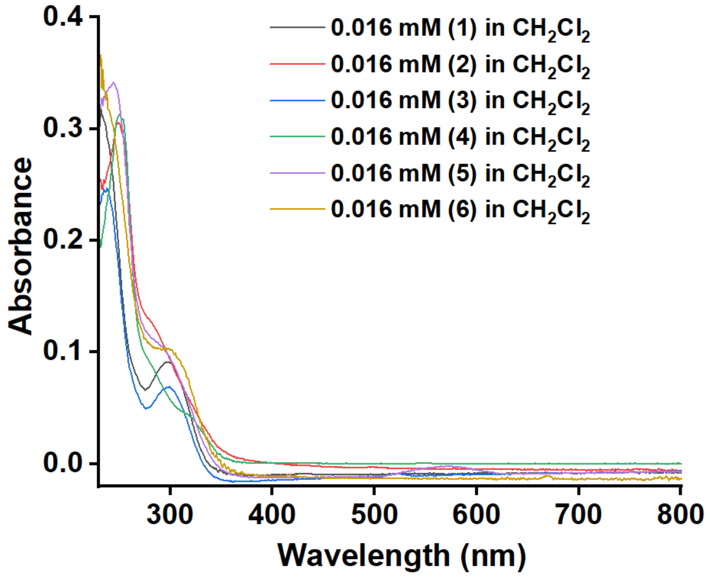

Figure S48. UV-Vis spectra of 0.016 mM of **(a)** [(L^1)BiCl$_2$] **(1), (b)** [(L^2)BiCl$_2$] **(2), (c)** [(L^3)BiCl$_2$] **(3)**, **(d)** [(L^4)BiCl$_2$] **(4), (e)** [(L^5)BiCl$_2$] **(5) , (f)** [(L^6)BiCl$_2$] **(6),** in dichloromethane at 298 K.

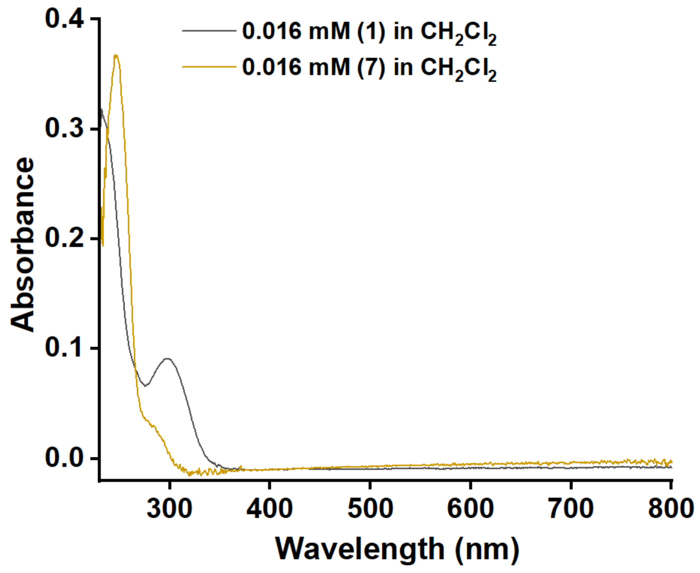

Figure S49. UV-Vis spectra of 0.016 mM of **(a)** [(L^1)BiCl$_2$] **(1), (b)** [(L^7)BiCl$_2$] **(7),** in dichloromethane at 298 K.

Single Crystal XRD (III)

X-ray crystallographic data collection and refinement for complex [(L^1)$BiCl_2$] (1):

X-ray single-crystal data for complexes were collected at 100 K using Mo Kα (λ = 0.7107Å) radiation on a Bruker D8 QUEST. Data collection, data reduction, structure solution and refinement were carried out using the software package of APEX 4. The structure was solved by difference vectors and fast Fourier transform methods.

Single crystals of complex were grown by layering (1:1) mixture of EtOAc and DCM. Complex crystallizes in a monoclinic system with Pc space group (Table S1).

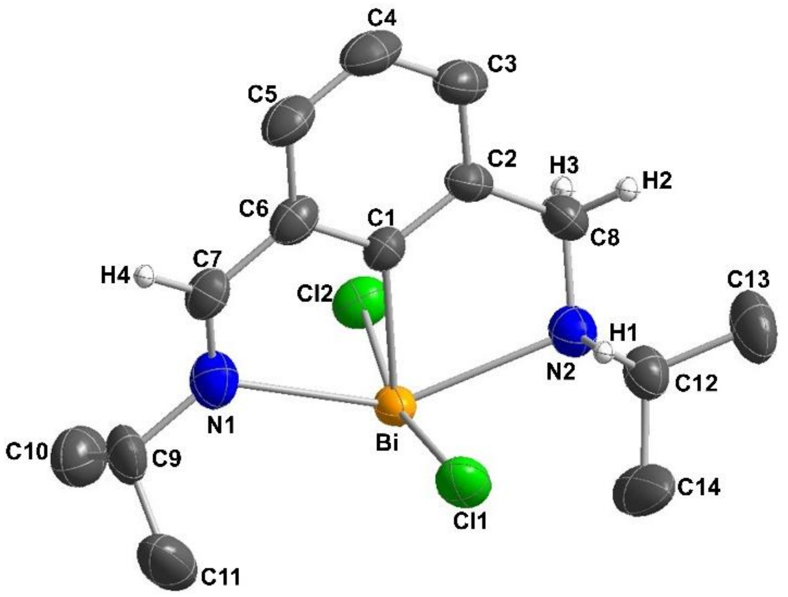

Figure S50. Crystal structure of **[(L^1)$BiCl_2$] (1)**. All H atoms except N2 and C7 and C8 have been removed for clarity and ellipsoids are drawn at 50% probability level.

Table S1. Crystal data and structure refinement of **[(L[1])BiCl$_2$] (1).**

Empirical formula	$C_{14}H_{21}BiCl_2N_2$
Formula weight	497.21 g·mol^{-1}
Temperature/K	100
Crystal system	monoclinic
Space group	Pc
a/Å	8.074
b/Å	23.791
c/Å	9.340
α/°	90
β/°	105.52
γ/°	90
Volume/Å^3	1728.7
Z	17
ρ_{calc} g/cm^3	1.910
μ/mm^{-1}	10.498
F(000)	944.0
Crystal size/mm^3	0.164 × 0.116 × 0.064
Radiation	MoKα (λ = 0.71073)
2Θ range for data collection/°	4.84 to 52.028
Index ranges	−9 ≤ h ≤ 9, −29 ≤ k ≤ 29, −11 ≤ l ≤ 11
Reflections collected	52427
Independent reflections	6738 [R_{int} = 0.0463, R_{sigma} = 0.0290]
Data/restraints/parameters	6738/2/351
Goodness-of-fit on F^2	1.090
Final R indexes [I>=2σ (I)]	R_1 = 0.0322, wR_2 = 0.0999
Final R indexes [all data]	R_1 = 0.0380, wR_2 = 0.1035
CCDC	2538081

Table S2. Bond distances (Å) for **[(L[1])BiCl$_2$] (1).**

Atoms	Bond Length in Å
Bi(1)-Cl(2)	2.783(5)
Bi(1)-Cl(1)	2.673(5)
Bi(1)-N(2)	2.468(18)
Bi(1)-N(1)	2.56(2)
Bi(1)-C(1)	2.172(18)
N(1)-C(7)	1.22(3)
N(2)-C(8)	1.48(3)
N(1)-C(9)	1.53(3)
N(2)-C(10)	1.55(3)
C(2)-C(1)	1.37(3)
C(2)- C(3)	1.46(3)
C(6)-C(7)	1.42(4)
C(2)- C(8)	1.46(3)
C(5)- C(6)	1.42(3)
C(5)- C(4)	1.38(3)
C(1)- C(6)	1.45(3)
C(3)- C(4)	1.28(3)

The compound (**1**) was found to be a racemic mixture of two enantiomers within the asymmetric unit.

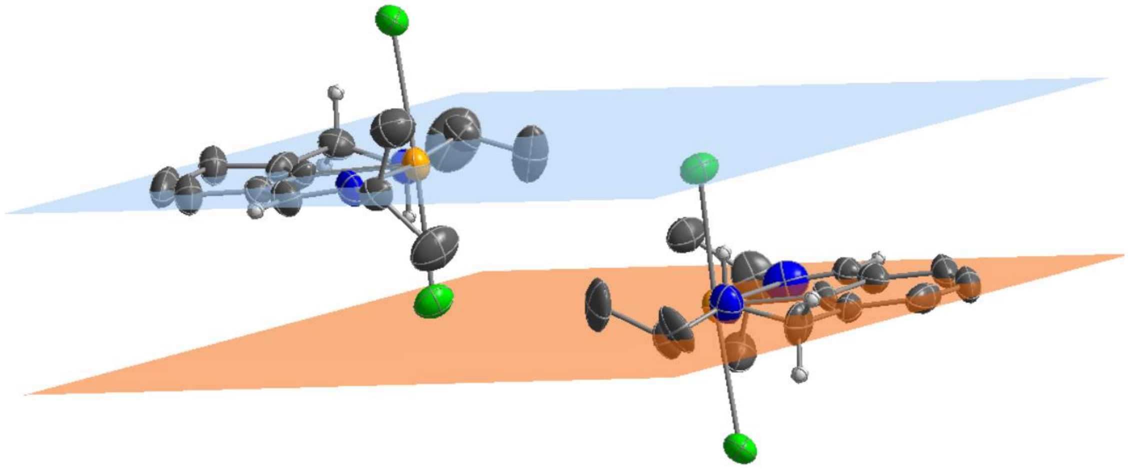

Figure S51. Out of plane orientation of protonated amine nitrogen atoms N indicating true enantiomers in the asymmetric unit of each **[(L[1])BiCl$_2$] (1)** moiety.

Table S3. Bond angles (°) for **[(L¹)BiCl₂] (1).**

Atom	Bond Angle
Cl(1)-Bi(1)-Cl(2)	168.19(17)
N(2)-Bi(1)-Cl(2)	86.9(5)
N(2)-Bi(1)-Cl(1)	81.4(5)
N(1)-Bi(1)-Cl(2)	97.9(5)
N(1)-Bi(1)-Cl(1)	90.1(5)
N(1)-Bi(1)-N(2)	141.2(6)
C(1)-Bi(1)-Cl(2)	84.7(5)
C(1)-Bi(1)-Cl(1)	89.8(5)
C(1)-Bi(1)-N(2)	72.0(6)
C(1)-Bi(1)-N(1)	70.2(7)
C(7)-N(1)-C(9)	123(2)
C(7)-N(1)-Bi(1)	113.2(15)
C(9)-N(1)-Bi(1)	123.3(16)
C(8)- N(2)-Bi(1)	108.4(13)
C(8)-N(2)-C(10)	116.0(180
C(10)-N(2)-Bi(10)	117.2(14)
C(2)-C(1)-Bi(1)	121.0(13)
C(6)-C(1)-Bi(1)	118.0(16)

X-ray crystallographic data collection and refinement for complex [(L^1)$BiCl_2$] (2):

X-ray single-crystal data for complex were collected at 100 K using Mo Kα (λ = 0.7107Å) radiation on a Bruker D8 QUEST. Data collection, data reduction, structure solution and refinement were carried out using the software package of APEX 4. The structure was solved by difference vectors and fast Fourier transform methods.

Single crystals of complex were grown by layering (1:1) mixture of EtOAc and DCM. Complex crystallizes in a monoclinic system with $P2_1/n$ space group (Table S4).

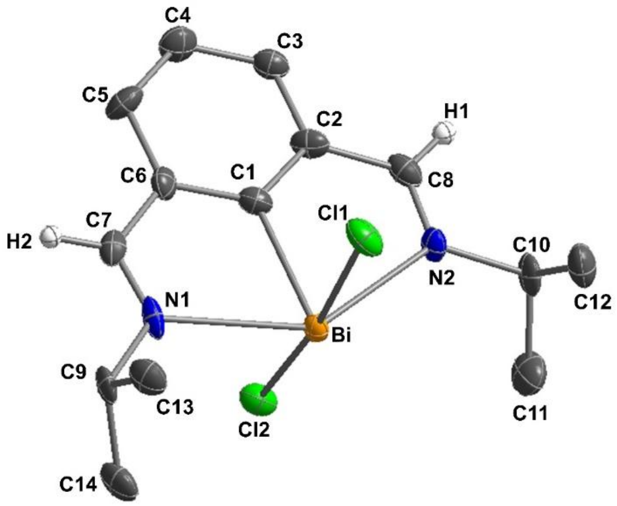

Figure S52. Crystal structure of complex **[(L^2BiCl_2] (2)**. All H atoms except those on C7 and C8 have been removed for clarity and ellipsoids are drawn at 50% probability level.

Table S4. Crystal data and structure refinement of complex **[(L^2BiCl_2] (2)**:

Empirical formula	$C_{14}H_{19}BiCl_2N_2$
Formula weight	495.19
Temperature/K	100.00
Crystal system	monoclinic
Space group	$P2_1/n$
a/Å	10.555(2)
b/Å	10.900(2)
c/Å	14.757(3)
α/°	90
β/°	98.180(7)
γ/°	90
Volume/Å^3	1680.5(6)
Z	4
ρ_{calc}g/cm^3	1.957
μ/mm^{-1}	10.799
F(000)	936.0
Crystal size/mm^3	0.16 × 0.151 × 0.068
Radiation	MoKα (λ = 0.71073)
2Θ range for data collection/°	4.458 to 52.054
Index ranges	−13 ≤ h ≤ 13, −13 ≤ k ≤ 13, −18 ≤ l ≤ 18
Reflections collected	52334
Independent reflections	3303 [R_{int} = 0.0660, R_{sigma} = 0.0268]
Data/restraints/parameters	3303/0/185
Goodness-of-fit on F^2	1.049
Final R indexes [I>=2σ (I)]	R_1 = 0.0195, wR_2 = 0.0443
Final R indexes [all data]	R_1 = 0.0229, wR_2 = 0.0456
CCDC	2538082

Table S5. Bond distances (Å) for **[(L^2)$BiCl_2$] (2)**:

**Atoms**	**Bond Length in Å**
Bi(1)-Cl(1)	2.69(11)
Bi(1)-Cl(2)	2.66(11)
Bi(1)-N(2)	2.49(3)
Bi(1)-N(1)	2.47(3)
Bi(1)-C(1)	2.20(3)
N(1)-C(7)	1.27(4)
N(2)-C(8)	1.28(4)
N(2)-C(10)	1.46(4)
N(1)-C(9)	1.47(4)
C(1)-C(2)	1.38(5)
C(1)- C(6)	1.38(5)
C(3)- C(4)	1.38(5)
C(3)- C(2)	1.40(5)
C(5)- C(4)	1.38(5)
C(5)- C(6)	1.40(5)
C(7)- C(6)	1.46(5)
C(8)- C(2)	1.47(5)
C(9)- C(13)	1.50(5)
C(9)- C(14)	1.52(5)
C(11)- C(10)	1.52(5)
C(10)- C(12)	1.52(5)

Table S6. Bond angles (°) for **[(L[2])BiCl$_2$] (2)**:

Atom	Bond Angle
Cl(1)-Bi(1)-Cl(2)	172.70(3)
N(2)-Bi(1)-Cl(2)	88.51(6)
N(2)-Bi(1)-Cl(1)	88.69(6)
N(1)-Bi(1)-Cl(2)	90.76(6)
N(1)-Bi(1)-Cl(1)	87.38(6)
N(1)-Bi(1)-N(2)	142.65(9)
C(1)-Bi(1)-Cl(2)	88.90(9)
C(1)-Bi(1)-Cl(1)	83.82(9)
C(1)-Bi(1)-N(2)	71.14(11)
C(1)-Bi(1)-N(1)	71.51(11)
C(7)-N(1)-C(9)	121.00(3)
C(7)-N(1)-Bi(1)	112.7(2)
C(9)-N(1)-Bi(1)	126.3(2)
C(8)- N(2)-Bi(1)	112.60(2)
C(8)-N(2)-C(10)	121.5(3)
C(10)-N(2)-Bi(1)	125.9(2)
C(2)-C(1)-Bi(1)	119.3(2)
C(6)-C(1)-Bi(1)	118.2(2)

Synthesis of different protic source acids (IV)[5]

a) Synthesis of dimethylformamidium triflate:

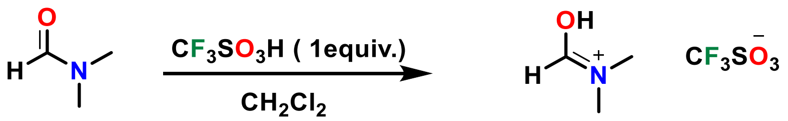

Scheme S17. Synthesis of dimethylformamidium triflate

A 50 mL round bottomed flask charged with a magnetic stir bar, then dry dimethyl formamide (DMF) (5 mL, 0.0642 moles) and dichloromethane (DCM) (8 mL) were added. To the stirring reaction mixture, triflic acid (5.7 mL, 0.064 moles, 1 equiv.) was added dropwise. The reaction mixture was allowed to stir for 5 minutes after that DCM was removed by rotary evaporation giving 14 g (98%) of dimethylformamidium triflate as a white solid whose NMR match with the literature report.[5]

NMR of dimethylformamidium triflates

^{1}H NMR (500 MHz, $CDCl_3$): δ 10.59 (s, 1H), 8.46 (s, 1H), 3.31 (s, 3H), 3.14 (s, 3H).

^{13}C NMR (125 MHz, $CDCl_3$): δ 165.65, 118.78, 40.66, 34.99.

^{19}F NMR (471 MHz, $CDCl_3$): δ −78.45, −79.11.

HR-MS (ESI$^+$) calculated for $[M]^+$ 74.0601; found 74.0600.

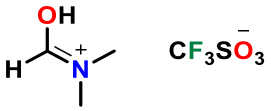

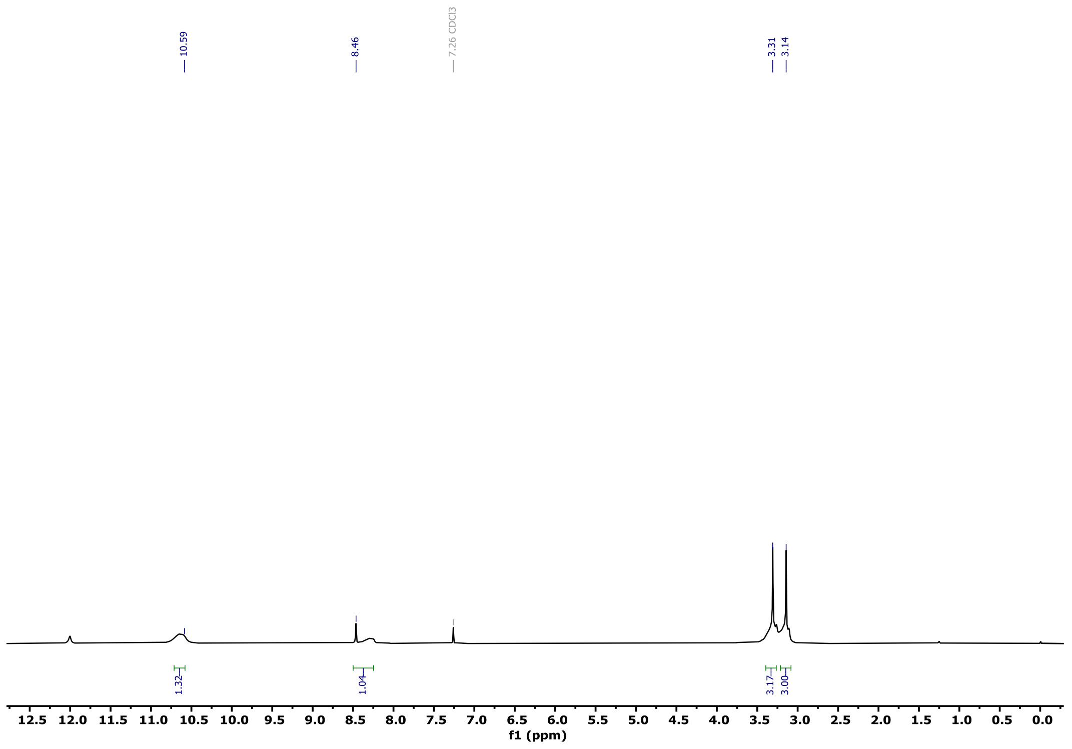

Figure S53. ^{1}H NMR (500 MHz) of dimethylformamidium triflate in $CDCl_3$ at 298 K

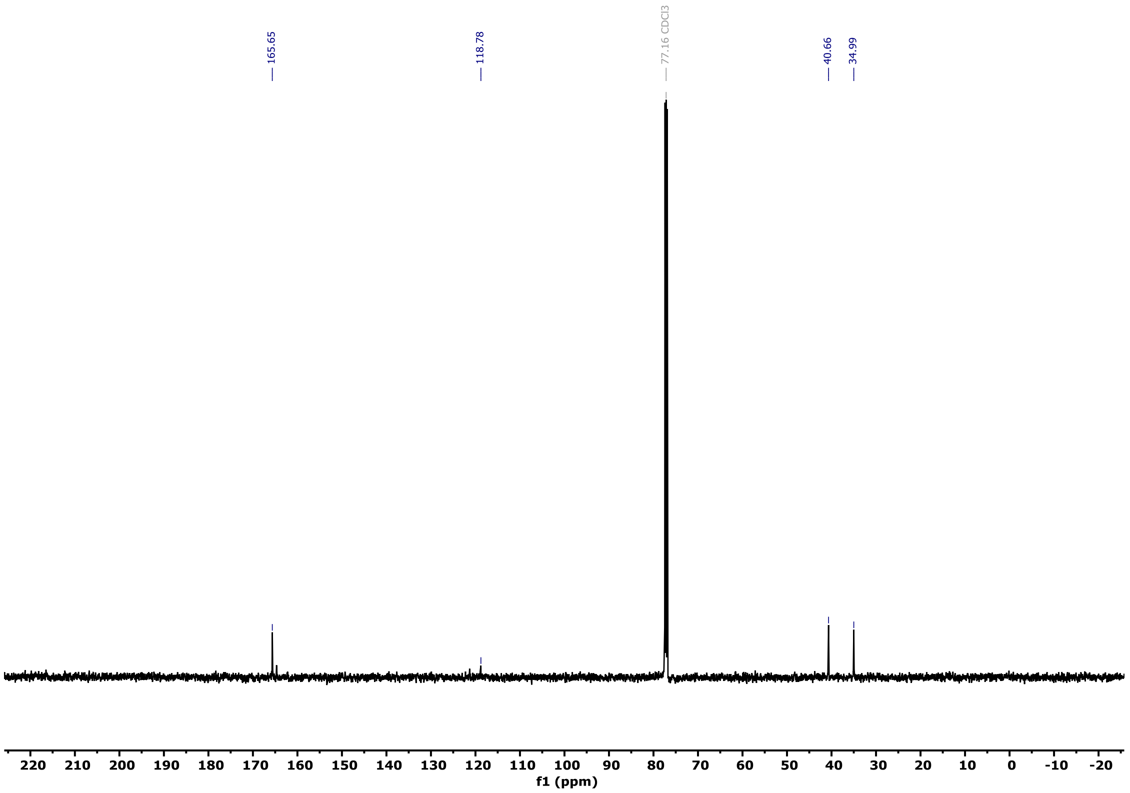

Figure S54. ^{13}C NMR (125 MHz) of dimethylformamidium triflate in $CDCl_3$ at 298 K

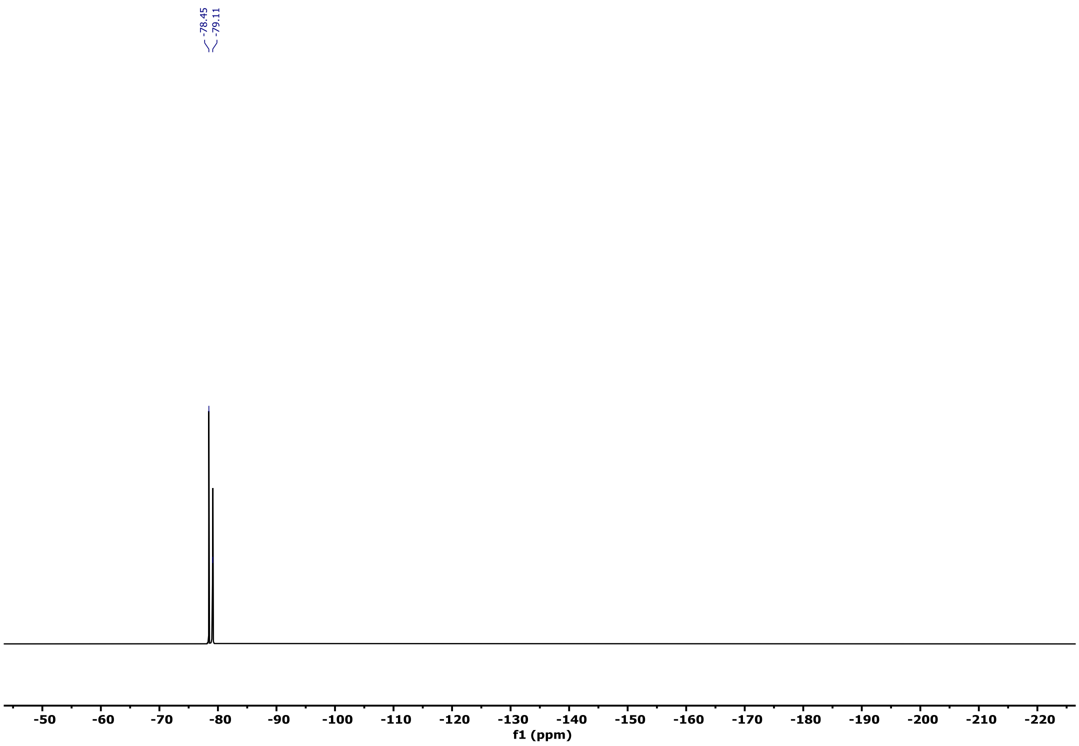

Figure S55. ^{19}F NMR (471 MHz) of dimethylformamidium triflate in $CDCl_3$ at 298 K

b) Synthesis of *p*-Chloroanilinium tetrafluoroborate:

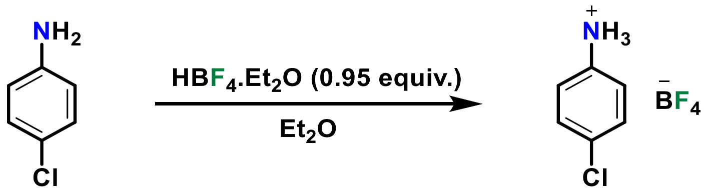

Scheme S18. Synthesis of *p*-Chloroanilinium tetrafluoroborate

A 50 mL round bottomed flask Charged with a magnetic stir bar, then *p*-Chloroaniline (1g, 0.0078 mol, 1 equiv.,) and diethyl ether (5 mL) was added. To the stirring reaction mixture, tetrafluoroboric acid-ether complex (1.01, 0.95 equiv., 0.03 mol) was added in dropwise manner. (A cool water bath was used during this addition as a precaution as the reaction was reported to be exothermic). After stirring mixture for around 20 - 25 minutes, small amount of ether was added further to obtain white solid product. The leftover ether was removed under vacuum. The white product was washed with two 4 mL portions of diethyl ether and was dried to yield a 2.16 g (78%) para-Chloroanilinium tetrafluoroborate as a white solid.

NMR of *p*-Chloroanilinium tetrafluoroborate

^{1}H NMR (500 MHz, $CDCl_3$): δ 8.25 (s, 1H), 7.56 – 7.53 (m, 2H), 7.43 – 7.38 (m, 2H).

^{13}C NMR (125 MHz, $CDCl_3$): δ 136.01, 130.97, 128.98, 126.18.

^{19}F NMR (471 MHz, $CDCl_3$): δ −150.91

HR-MS (ESI$^+$) calculated for $[M]^+$ 128.0262; found 128.0266.

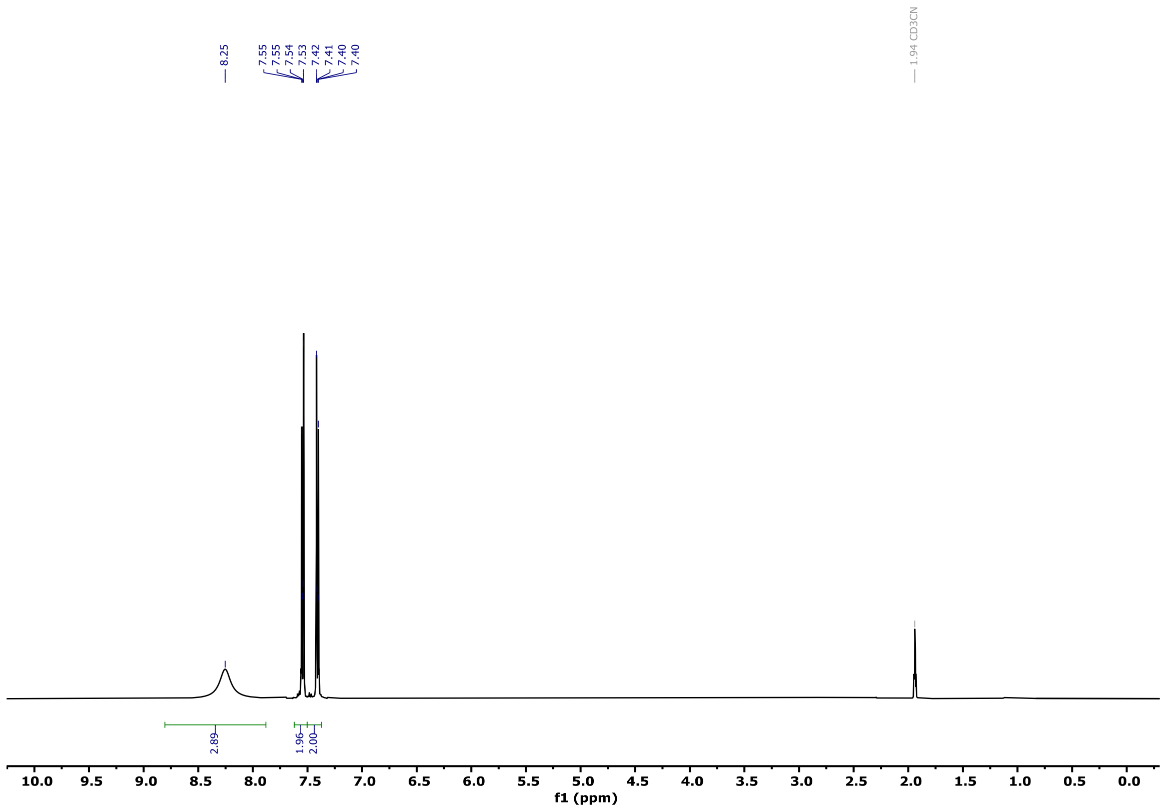

Figure S56. ^{1}H NMR (500 MHz) of *p*-Chloroanilinium tetrafluoroborate in CD_3CN at 298 K.

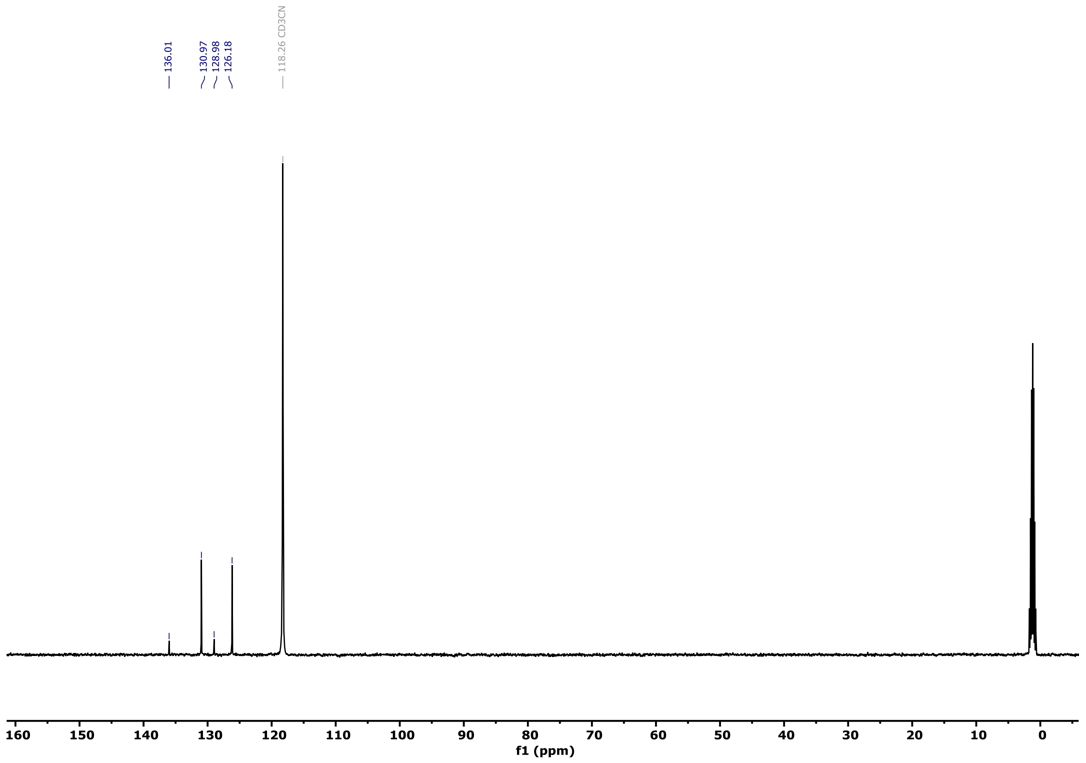

Figure S57. ^{13}C NMR (125 MHz) of *p*-Chloroanilinium tetrafluoroborate in CD_3CN at 298 K.

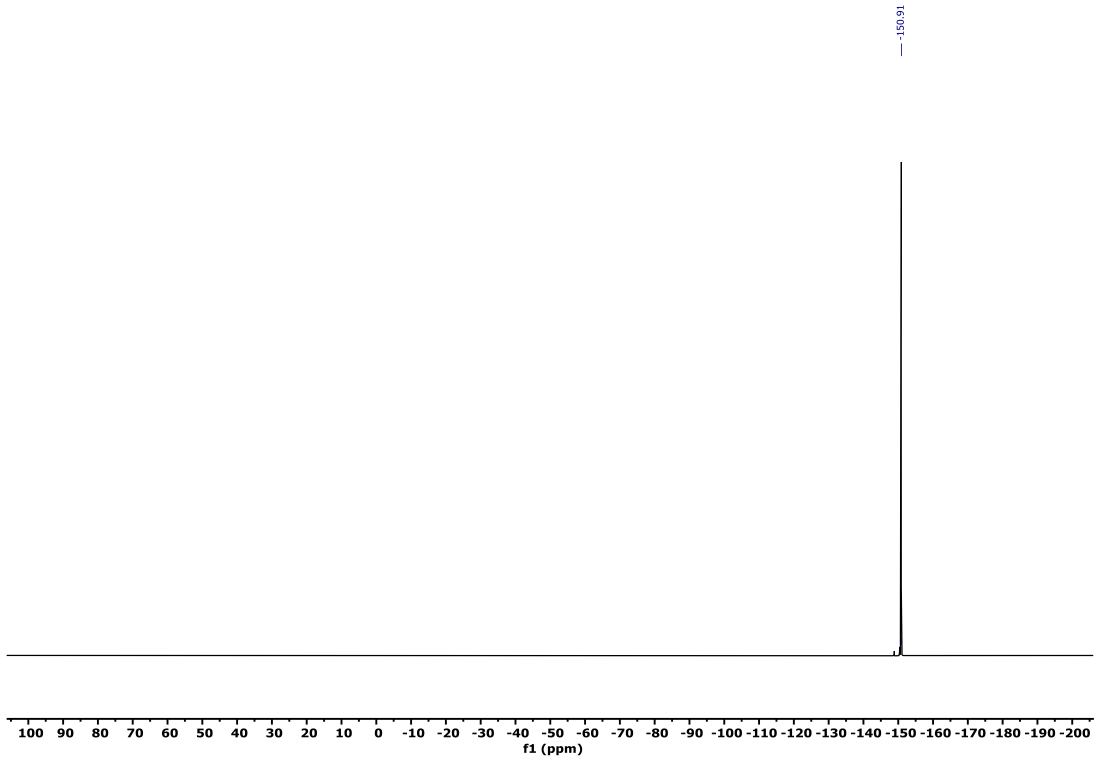

Figure S58. ^{19}F NMR (471 MHz) of *p*-Chloroanilinium tetrafluoroborate in CD_3CN at 298 K.

c) Synthesis of anilinium tetrafluoroborate:

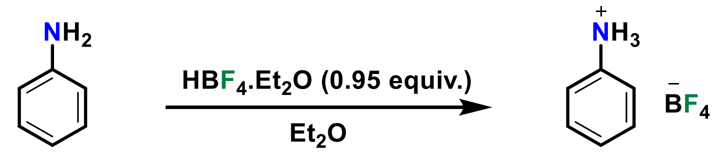

Scheme S19. Synthesis of anilinium tetrafluoroborate

A 50 mL round bottomed flask Charged with a magnetic stir bar, then aniline (3 mL, 0.032 mol, 1 equiv.,) and diethyl ether (5 mL) was added. To the stirring reaction mixture, tetrafluoroboric acid-ether complex (4.2 mL, 0.95 equiv., 0.030 mol) was added in dropwise manner. (A cool water bath was used during this addition as a precaution as the reaction was reported to be exothermic). After stirring mixture for around 20 - 25 minutes, small amount of ether was added further to obtain white solid product. The leftover ether was removed under vacuum. The white product was washed with two 4 mL portions of diethyl ether and was dried to yield a 5.2 g (84.5%) anilinium tetrafluoroborate as a white solid.

NMR of Anilinium tetrafluoroborate

^{1}H NMR (500 MHz, CD_3CN): δ 8.22 (s, 3H), 7.60 – 7.52 (m, 3H), 7.42 (dd, J = 7.8, 2.0 Hz, 2H).

^{13}C NMR (125 MHz, CD_3CN): δ 125.49, 125.09, 124.61, 99.99.

^{19}F NMR (471 MHz, $CDCl_3$): δ −151.06.

HR-MS (ESI$^+$) calculated for $[M]^+$ 94.0652; found 94.0650.

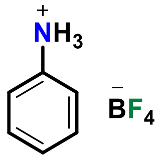

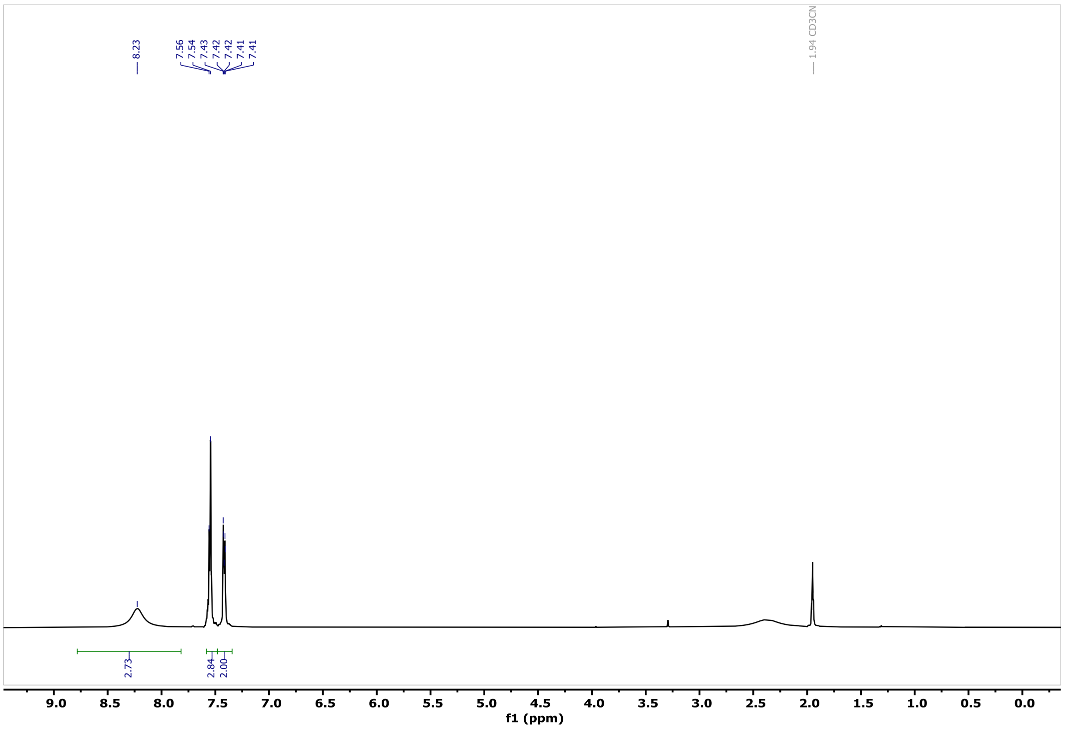

Figure S59. ^{1}H NMR (500 MHz) of anilinium tetrafluoroborate in CD_3CN at 298 K.

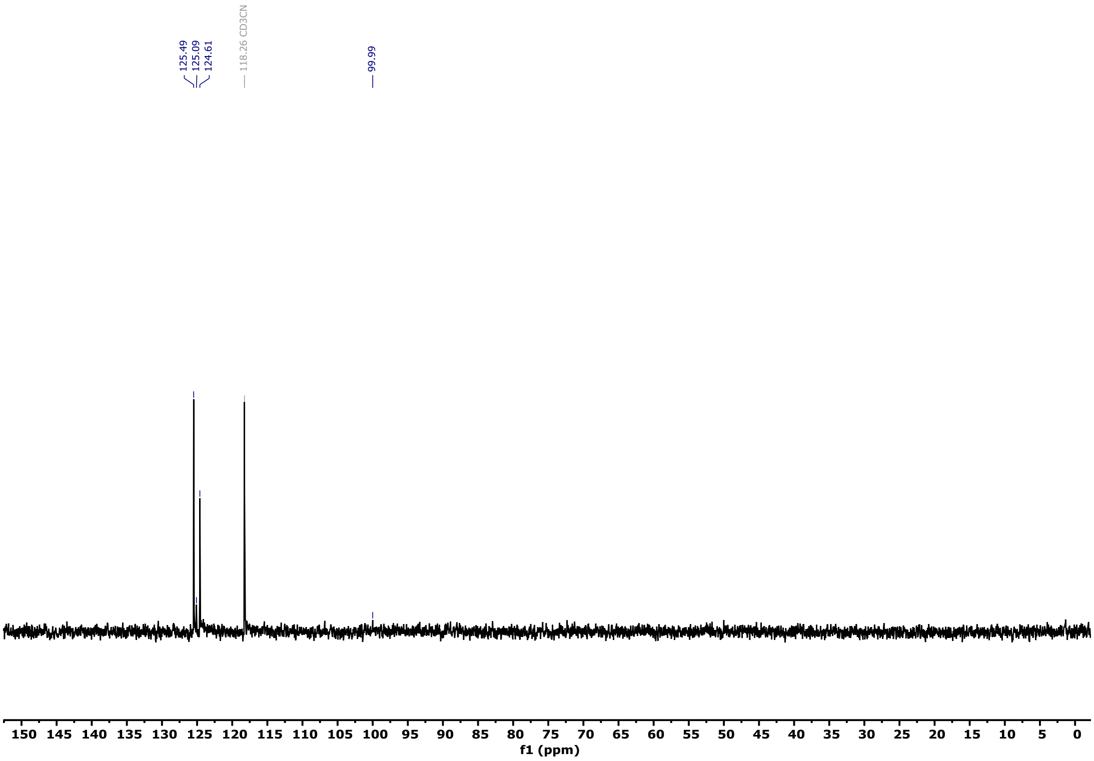

Figure S60. ^{13}C NMR (125 MHz) of anilinium tetrafluoroborate in CD_3CN at 298 K.

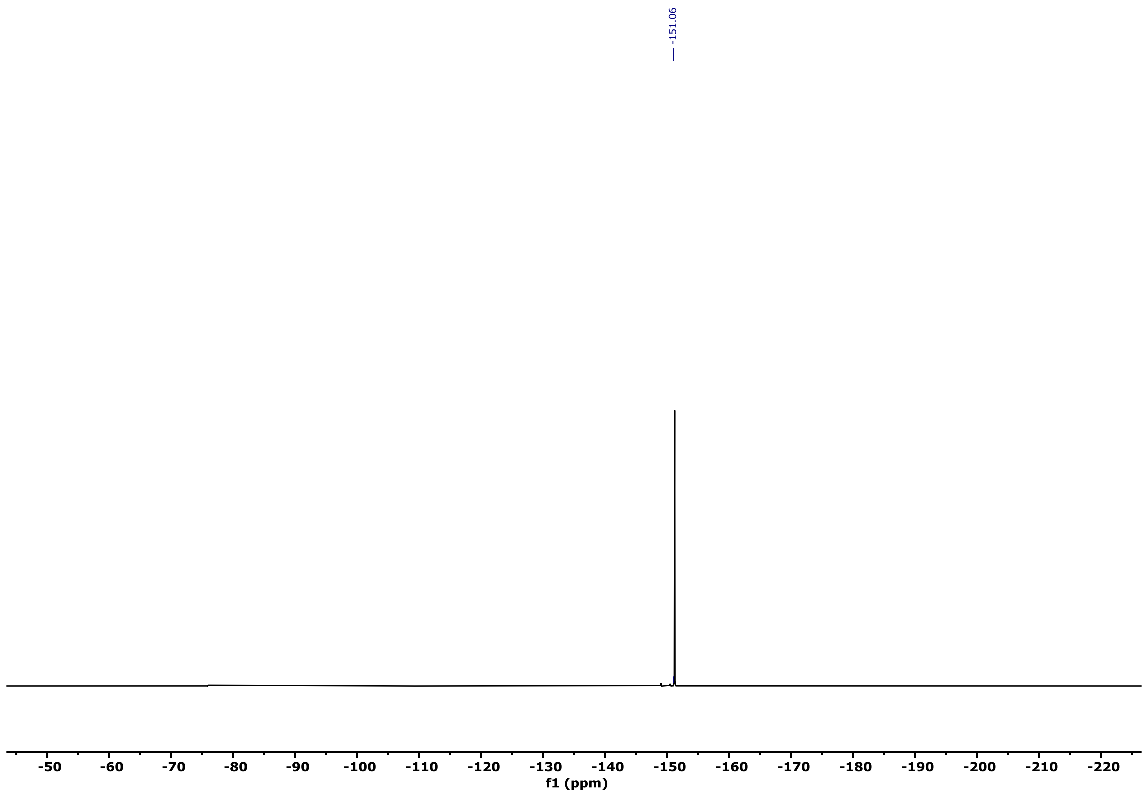

Figure S61. ^{19}F NMR (471 MHz) of anilinium tetrafluoroborate in CD_3CN at 298 K.

d) Synthesis of *p*-anisidium tetrafluoroborate:

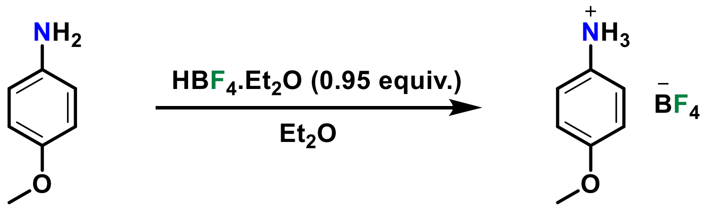

Scheme S20. Synthesis of *p*-anisidium tetrafluoroborate

A 50 mL round bottomed flask charged with a magnetic stir bar, then 4-methoxyaniline (3g, 0.024 mol, 1 equiv.) and diethyl ether (5 mL) was added. To the stirring reaction mixture, tetrafluoroboric acid-ether complex (4.2 mL, 0.95 equiv., 0.0306 mol) was added dropwise. (A cool water bath was used during this addition as a precaution as the reaction was reported to be exothermic). After stirring for around 20 - 25 minutes, small amount of ether was added further to obtain white solid product which was scratched. The excess ether was removed under vacuum. The product was washed with two 4 mL portions of diethyl ether and was dried to yield a 5.4 g (80%) of *p*-anisidium tetrafluoroborate as white solid.

NMR of *p*-anisidium tetrafluoroborate

^{1}H NMR (500 MHz, CD_3CN): δ 7.34 – 7.32 (m, 2H), 7.05 – 7.03 (m, 2H), 3.82 (s, 3H).

^{13}C NMR (125 MHz, CD_3CN): δ 161.23, 125.57, 122.71, 116.07, 56.39.

^{19}F NMR (471 MHz, $CDCl_3$): δ −150.74

HR-MS (ESI$^+$) calculated for $[M]^+$ 124.0757; found 124.0791.

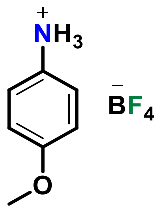

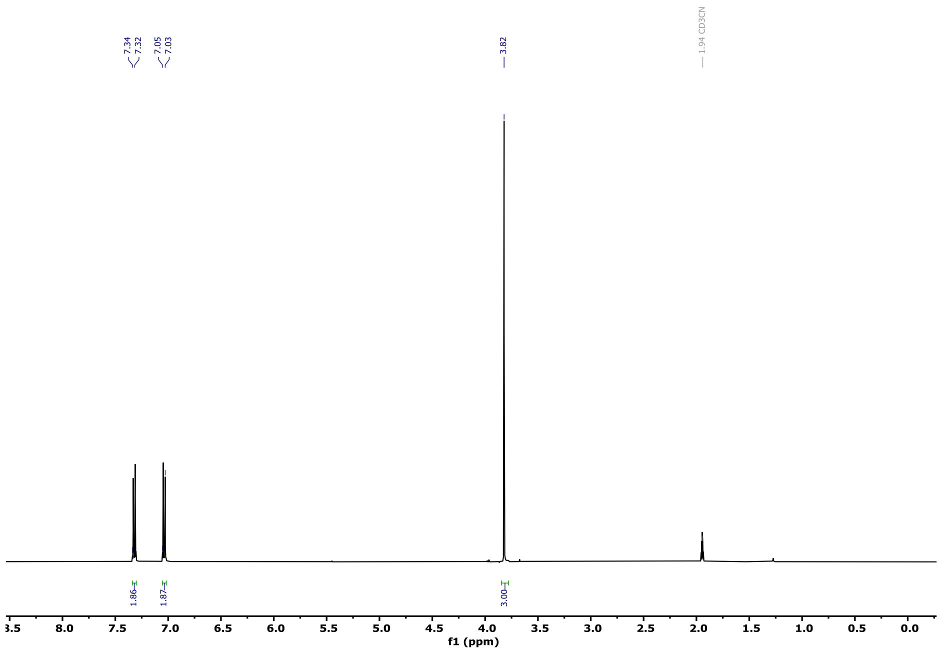

Figure S62. ^{1}H NMR (500 MHz) of *p*-anisidium tetrafluoroborate in CD_3CN at 298 K.

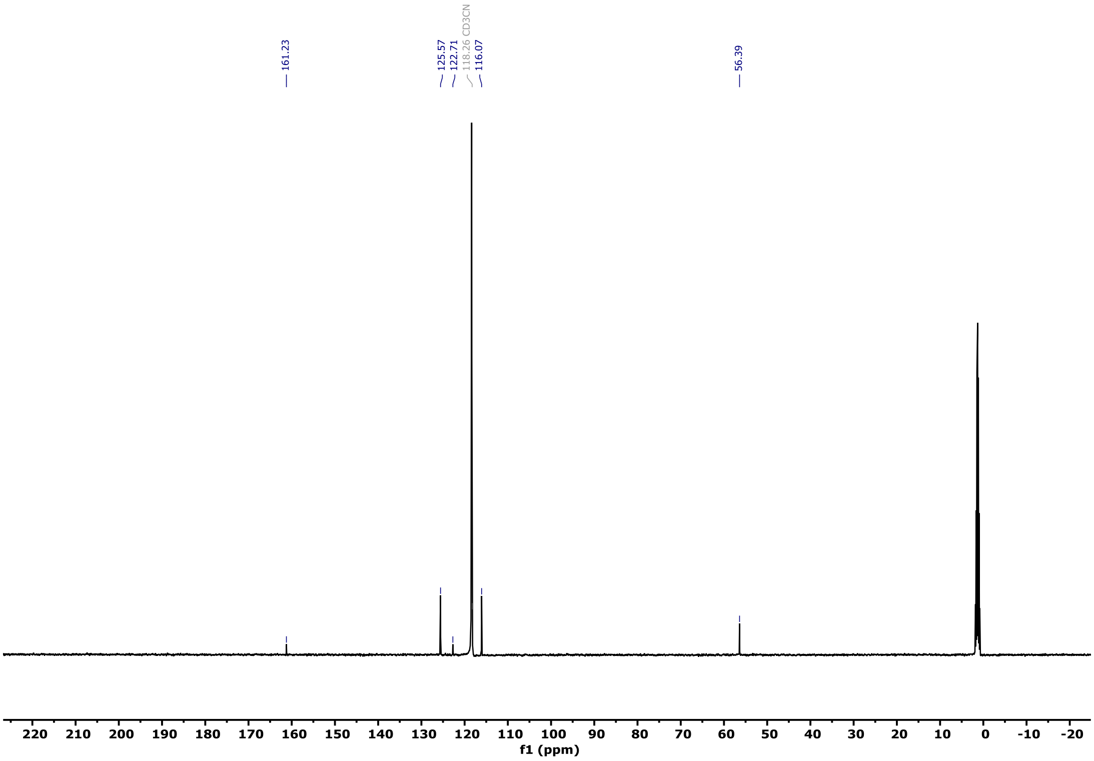

Figure S63. ^{13}C NMR (125 MHz) of *p*-anisidium tetrafluoroborate in CD_3CN at 298 K

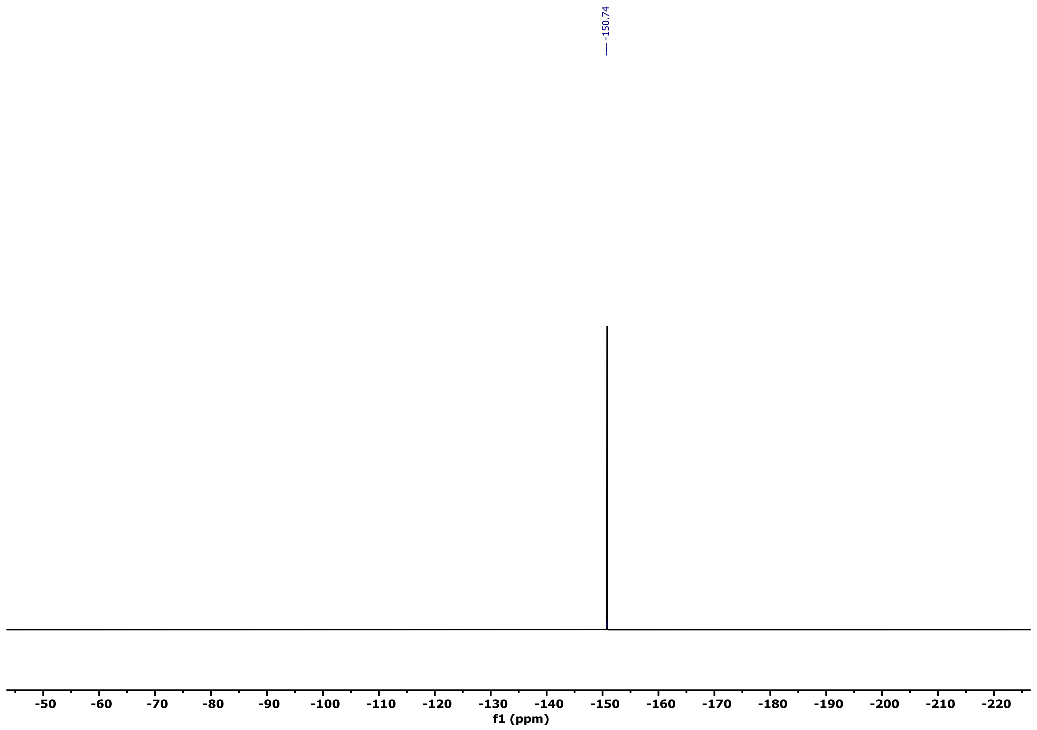

Figure S64. ^{19}F NMR (471 MHz) of pyridinium tetrafluoroborate in CD_3CN at 298 K

e) Synthesis of Pyridinium Tetrafluoroborate:

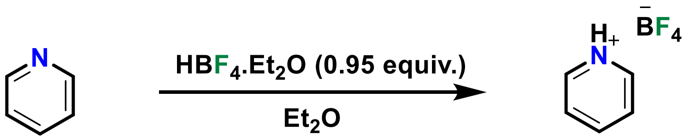

Scheme S21. Synthesis of pyridinium tetrafluoroborate

A 50 mL round bottomed flask charged with a magnetic stir bar, then pyridine (1 g, 0.0126 mol, 1 equiv.) was added in diethyl ether (10 mL) under nitrogen filled ballon. To the stirring reaction mixture 1.97 mL of $HBF_4.Et_2O$ (0.95 mol. equiv.) was added with vigorous agitation. Within 5 min white solid precipitate appears to this 5 mL of Et_2O was added again and the supernatant decanted. The obtained solids were washed 3 times with 10 mL Et_2O each time followed by drying under vacuum at room temperature for about 1 hours and after final washing it was dried under high vacuum for 8 - 10 h to yield 1.99 g (94.6%) of pyridinium tetrafluoroborate as a pure off-white solid.

NMR of pyridinium tetrafluoroborate

^{1}H NMR (500 MHz, CD_3CN): δ 8.75 (d, J = 5.2 Hz, 2H), 8.62 (tt, J = 7.9, 1.5 Hz, 1H), 8.07 (t, J = 7.3 Hz, 2H)

^{13}C NMR (125 MHz, CD_3CN): δ 148.66, 142.49, 128.56.

^{19}F NMR (471 MHz, $CDCl_3$): δ −151.26.

HR-MS (ESI$^+$) calculated for $[M]^+$ 80.0495; found 80.0492.

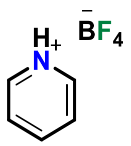

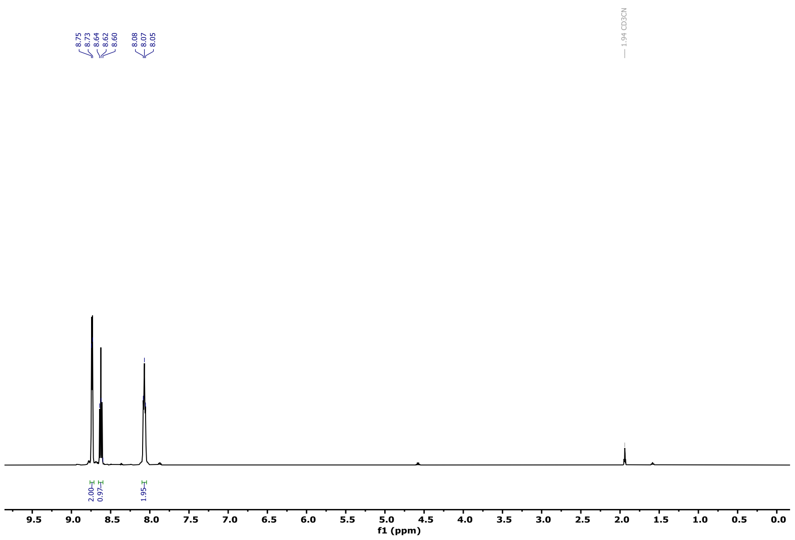

Figure S65. ^{1}H NMR (500 MHz) of pyridinium tetrafluoroborate in CD_3CN at 298 K.

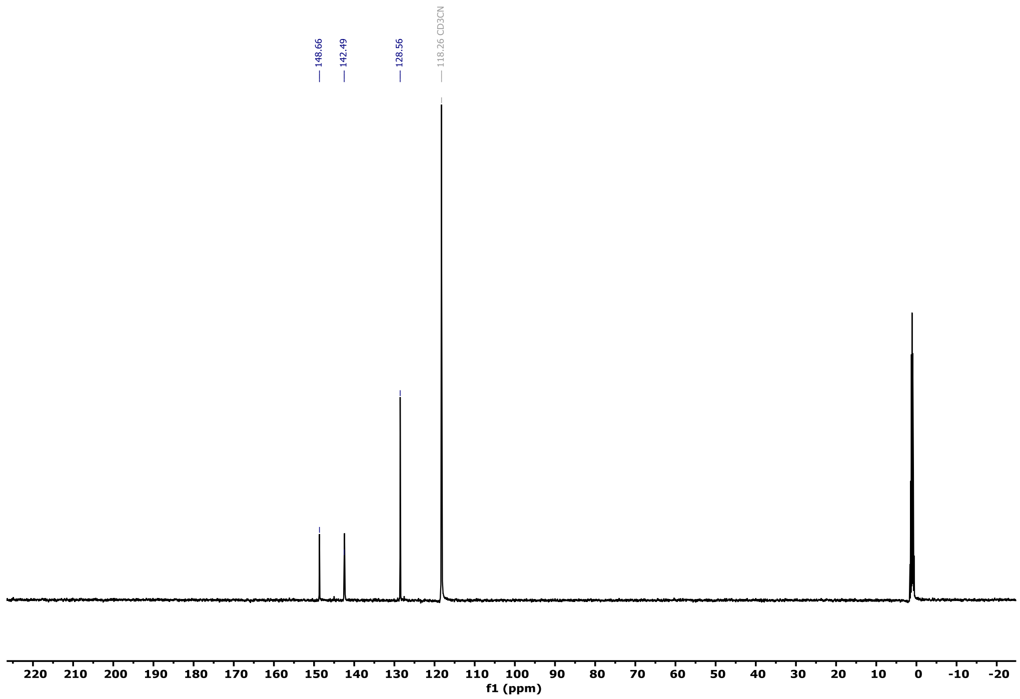

Figure S66. ^{13}C NMR (125 MHz) of pyridinium tetrafluoroborate in CD_3CN at 298 K

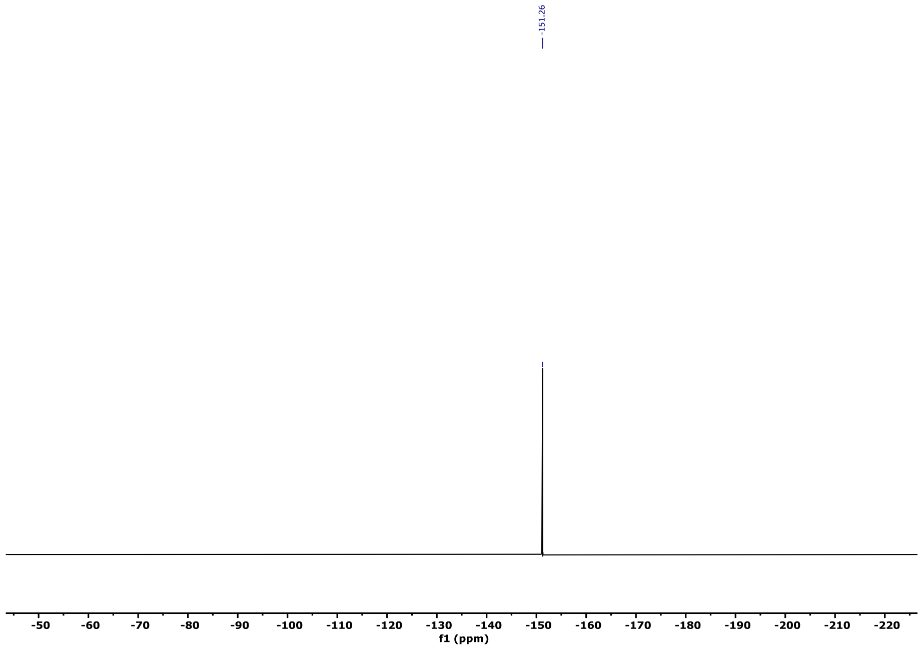

Figure S67. ^{19}F NMR (471 MHz) of pyridinium tetrafluoroborate in CD_3CN at 298 K

f) Synthesis of 2-Aminopyridinium Tetrafluoroborate:

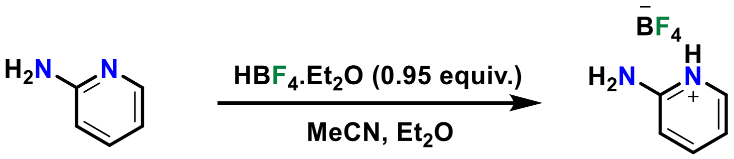

Scheme S22. Synthesis of 2-aminopyridinium tetrafluoroborate

A 50 mL round bottomed flask charged with a magnetic stir bar, then 2-aminopyridine (1 g, 0.0106 mol, 1 equiv.) was added in diethyl ether (10 mL) under nitrogen filled ballon. To the stirring reaction mixture 1.97 mL of $HBF_4.Et_2O$ (0.95 mol. equiv.) was added with vigorous agitation. Within 5 min white solid precipitate appears to this 5 mL of Et_2O was added again and the supernatant decanted. The obtained solids were washed 3 times with 10 mL Et_2O each time followed by drying under vacuum at room temperature for about 1 hours and after final washing it was dried under high vacuum for 8 - 10 h to yield 1.35 g (70.3%) of 2-aminopyridinium tetrafluoroborate pure off-white solid.

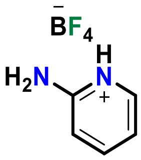

NMR of 2-aminopyridinium tetrafluoroborate

^{1}H NMR (500 MHz, CD_3CN): δ 11.14 (s, 1H), 7.91 (ddd, J = 8.9 Hz, 1H), 7.76 (t, J = 5.6 Hz, 1H), 7.05 – 7.00 (m, 1H), 6.89 (t, J = 6.8 Hz, 1H), 6.63 (s, 2H).

^{13}C NMR (125 MHz, CD_3CN): δ 154.82, 145.89, 136.12, 114.76, 114.15.

^{19}F NMR (471 MHz, $CDCl_3$): δ −150.57.

HR-MS (ESI$^+$) calculated for $[M]^+$ 95.0604; found 95.0602.

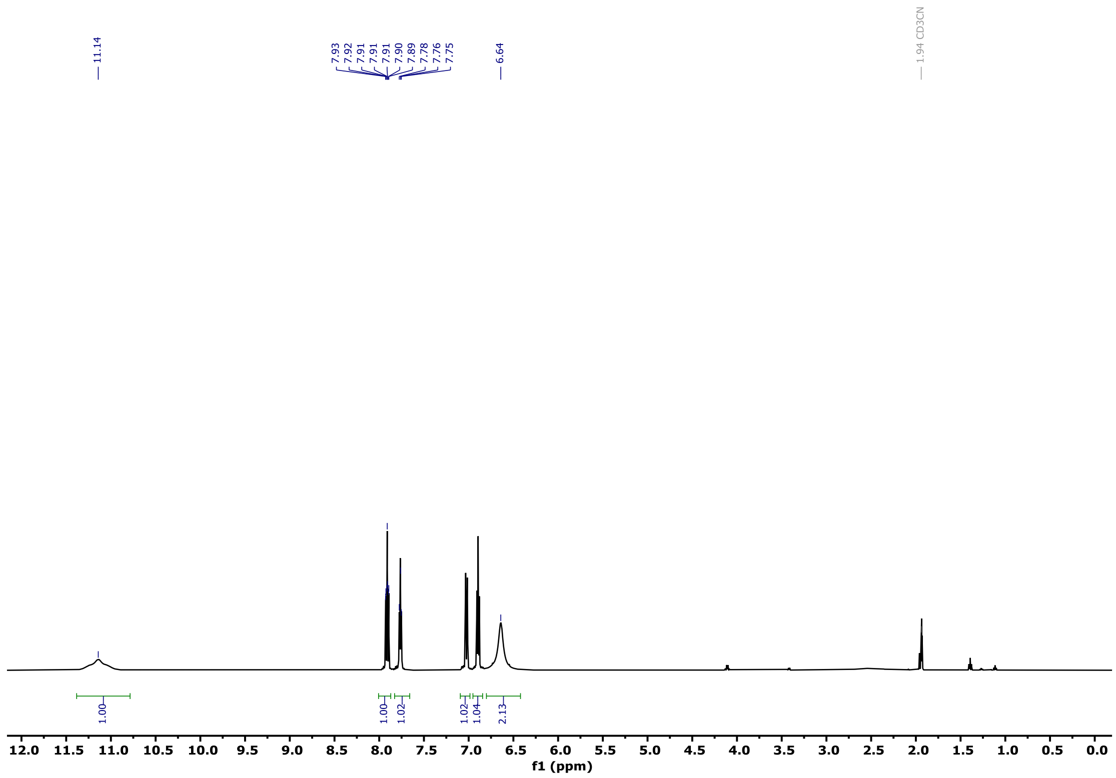

Figure S68. ^{1}H NMR (500 MHz) of 2-aminopyridinium tetrafluoroborate in CD_3CN at 298 K.

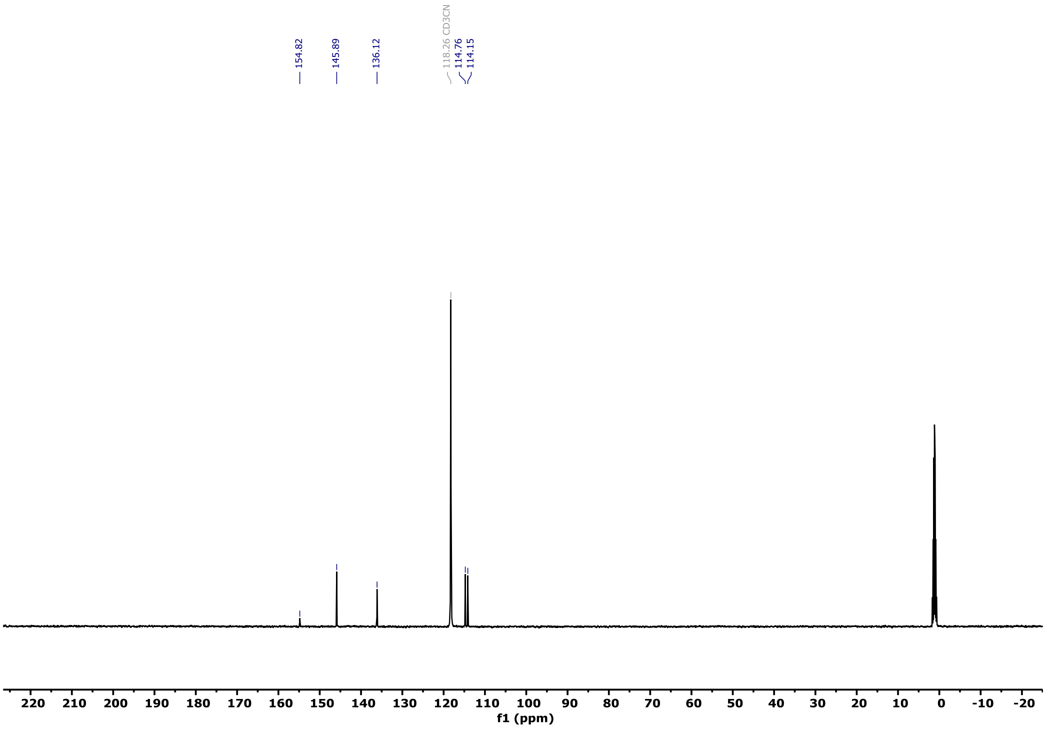

Figure S69. ^{13}C NMR (125 MHz) of 2-aminopyridinium tetrafluoroborate in CD_3CN at 298 K

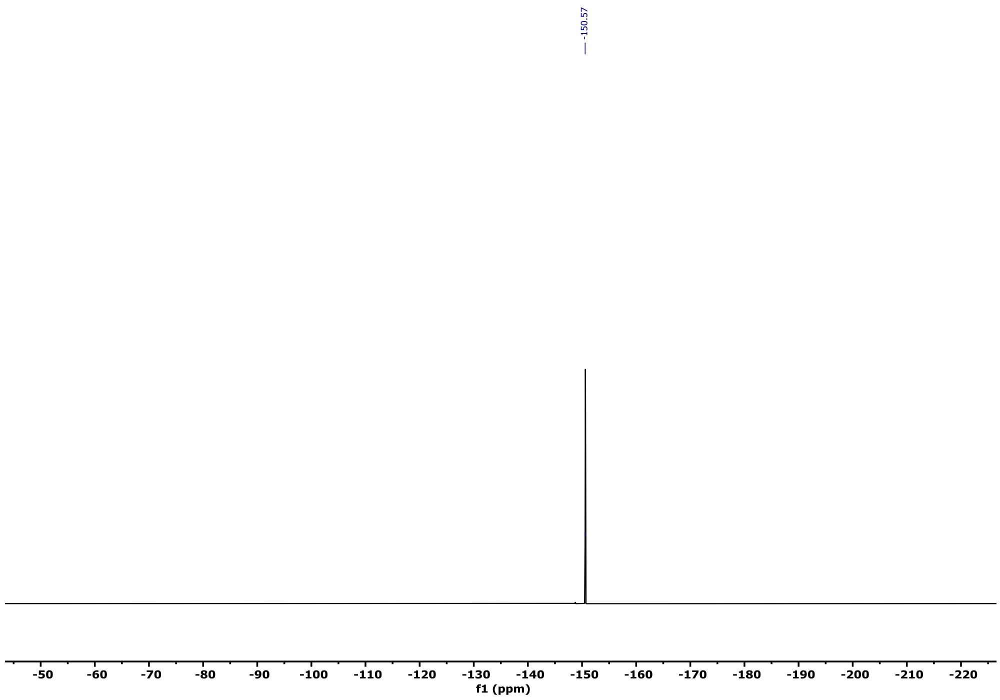

Figure S70. ^{19}F NMR (471 MHz) of 2-aminopyridinium tetrafluoroborate in CD_3CN at 298 K.

Table S7. List of proton sources used for proton reduction studies and their corresponding pK_a values in Acetonitrile.[5]

S.No.	Proton source	pK_a in Acetonitrile[5]
1.	Dimethylformamidium triflate	6.1
2.	*p*-chloroanilinium tetrafluoroborate	9.7
3.	Anilinium tetrafluoroborate	10.62
4.	Trichloroacetic acid	10.75
5.	4-methoxyanilinium tetafluoroborate	11.86
6.	Pyridinium tetrafluoroborate	12.53
7.	Trifluoroacetic acid	12.65
8.	2-aminopyridinium tetrafluoroborate	14.47
9.	Pentafluorophenol	20.1
10.	4-trifluoromethylphenol	25.54
11.	Phenol	29.4

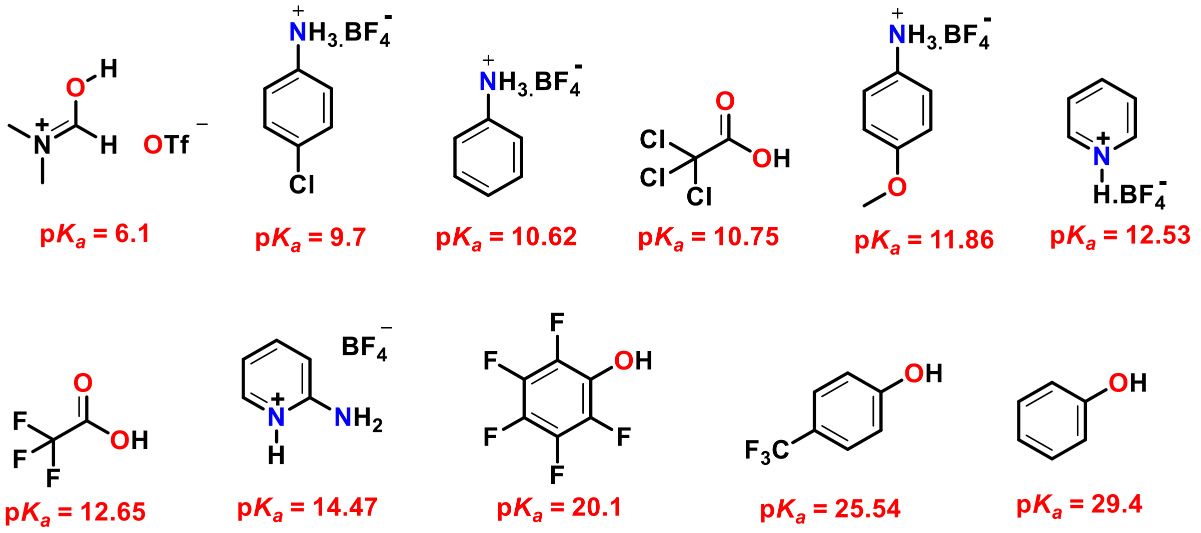

Scheme S23. Proton sources used for the present work and their corresponding pK_a values in acetonitrile.

Electrochemical studies of Complexes (V)

Electrochemical measurements were recorded with glassy carbon electrode as the working electrode, platinum electrode as the counter electrode and Ag/AgCl being the pseudo reference electrode. The solvent was Acetonitrile. Tetrabutylammonium hexafluorophosphate ($TBAPF_6$) was purified by recrystallization from ethanol and dried 5 - 6 hr in high vacuum in a vacuum before being stored in a desiccator and 0.1 M $TBAPF_6$ acted as an electrolyte in all the measurements. All data were referenced to an internal ferrocene standard (ferrocenium/ferrocene (Fc/Fc^+) redox potential under stated conditions). Other than scan rate variation studies all the cyclic voltametric measurements for proton reduction titrations were performed at a scan rate of 100 mV/s. The acids with their corresponding pK_a values in acetonitrile are listed in Table S7. All titrations were performed in presence of 0.5 mM concentration of the respective catalyst till the acid concentration reached 0.1 M (200 equiv.). Control studies in the absence of any catalyst were also carried out. To perform rinse test, a catalytic CV with 0.1 M proton source was recorded followed by a CV measurement with only the electrolyte and the solvent after rinsing the electrode with the solvent.

Electrochemical Data

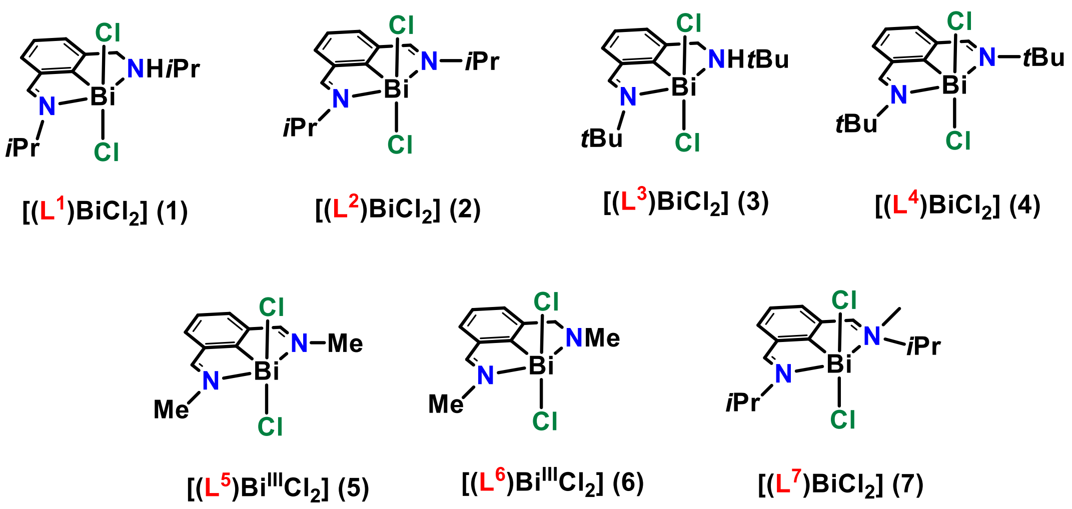

Scheme S24. Bismuth complexes studied in this work

Preliminary scan rate variation studies and E_{red} determination: (VI)

Electrochemical analyses were performed for all the catalysts. The catalysts exhibited a pseudo reversible oxidation and reduction peak implicating some structural reorganisation between the oxidised and reduced species (vide infra). The E_{red} vs Fc/Fc^+ for all the catalysts $[(L^1)BiCl_2]$ **(1),** $[(L^2)BiCl_2]$ **(2),** $[(L^3)BiCl_2]$ **(3)**, $[(L^4)BiCl_2]$ **(4),** $[(L^5)BiCl_2]$ **(5) ,** $[(L^6)BiCl_2]$ **(6),** $[(L^7)BiCl_2]$ **(7)** were calculated for the Bi^I/Bi^{III} redox couple which shows values of −1.14 V, −1.09 V, −1.25 V, and −1.11 V, −0.95 V, −1.06 V, −1.11 V respectively vs Fc/Fc^+ in acetonitrile.

The E_{red} values for all the catalysts that was calculated by taking the average of $E_{p,c}$ (cathodic peak potential) and $E_{p,a}$ (anodic peak potential) from the catalyst CVs is summarised in the table below.

Table S8. E_{red} (V) vs Fc/Fc^+ for catalysts $[(L^1)BiCl_2]$ **(1),** $[(L^2)BiCl_2]$ **(2),** $[(L^3)BiCl_2]$ **(3)**, $[(L^4)BiCl_2]$ **(4),** $[(L^5)BiCl_2]$ **(5) ,** $[(L^6)BiCl_2]$ **(6),** $[(L^7)BiCl_2]$ **(7)** in Acetonitrile,

**Catalyst**	**1**	**2**	**3**	**4**	**5**	**6**	**7**
**E_{red} (V) vs Fc/Fc^+**	−1.14	−1.09	−1.25	−1.11	−0.95	−1.06	−1.11

Table S9. **ΔEp** (V) vs Fc/Fc^+ for catalysts $[(L^1)BiCl_2]$ **(1),** $[(L^2)BiCl_2]$ **(2),** $[(L^3)BiCl_2]$ **(3)**, $[(L^4)BiCl_2]$ **(4)** in Acetonitrile

Catalyst	Epc	Epa	ΔE_p
1	−1.19	−1.09	0.100
2	−1.33	−0.86	0.470
3	−1.57	−0.93	0.63
4	−1.46	−0.77	0.69

Analyses of the current density versus square root of scan rate plot for all the complexes ([(L^1)$BiCl_2$] **(1),** [(L^2)$BiCl_2$] **(2),** [(L^3)$BiCl_2$] **(3)**, [(L^4)$BiCl_2$] **(4),** [(L^5)$BiCl_2$] **(5),** [(L^6)$BiCl_2$] **(6),** [(L^7)$BiCl_2$] **(7)**) gave a straight line obeying the Randles-Sevcik equation (eq. (i)) indicating that the catalytic phenomenon is under diffusion-controlled process.[6] (Figure S71-S77)

$$i_p = 0.446nFAC^0\,(n\mathrm{F}\upsilon D_0/\mathrm{RT})^{1/2}$$

where i_p is the peak current(A), υ is the scan rate (V/s), n is the number of electrons transferred in the redox event, A is the electrode surface area (cm^2), D_0 is the diffusion coefficient of the oxidised analyte and C^0 is bulk analyte concentration.

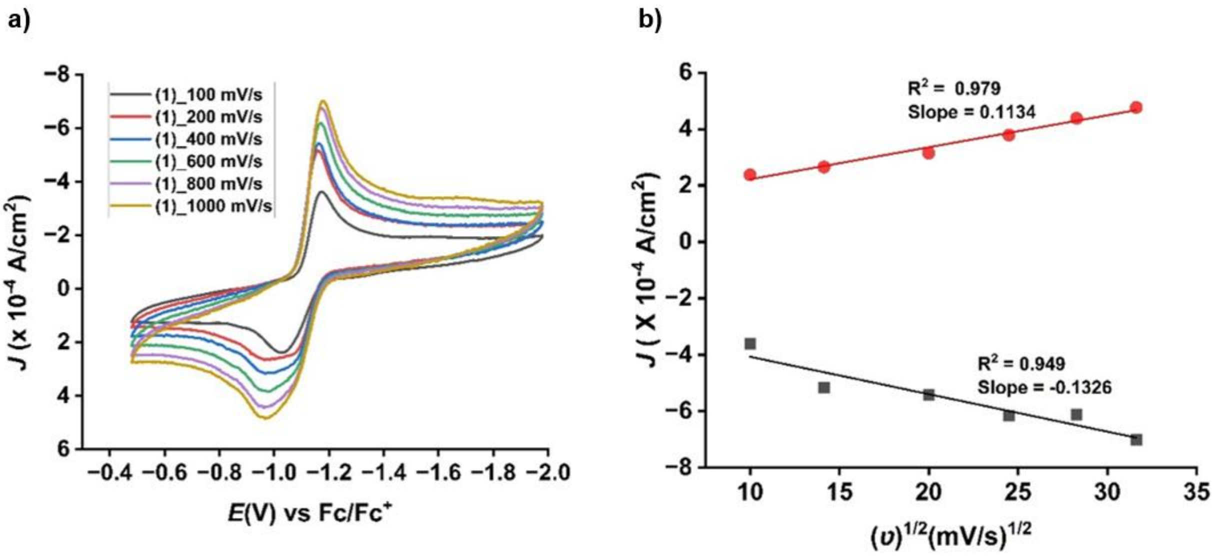

Figure S71. a) Cyclic Voltammogram of 0.5 mM [(L^1)$BiCl_2$] **(1)** varying scan rate with 0.1 M $TBAPF_6$ as a supporting electrolyte in acetonitrile. Condition: Working electrode glassy carbon (3 mm diameter), counter electrode Pt wire, and reference is Ag/AgCl. Data referenced to Fc/Fc^+ couple. **b)** Current density vs square root of scan rate plot for cathodic (black) and anodic (red) peaks in Cyclic Voltammogram of **[(L^1)$BiCl_2$] (1)**.

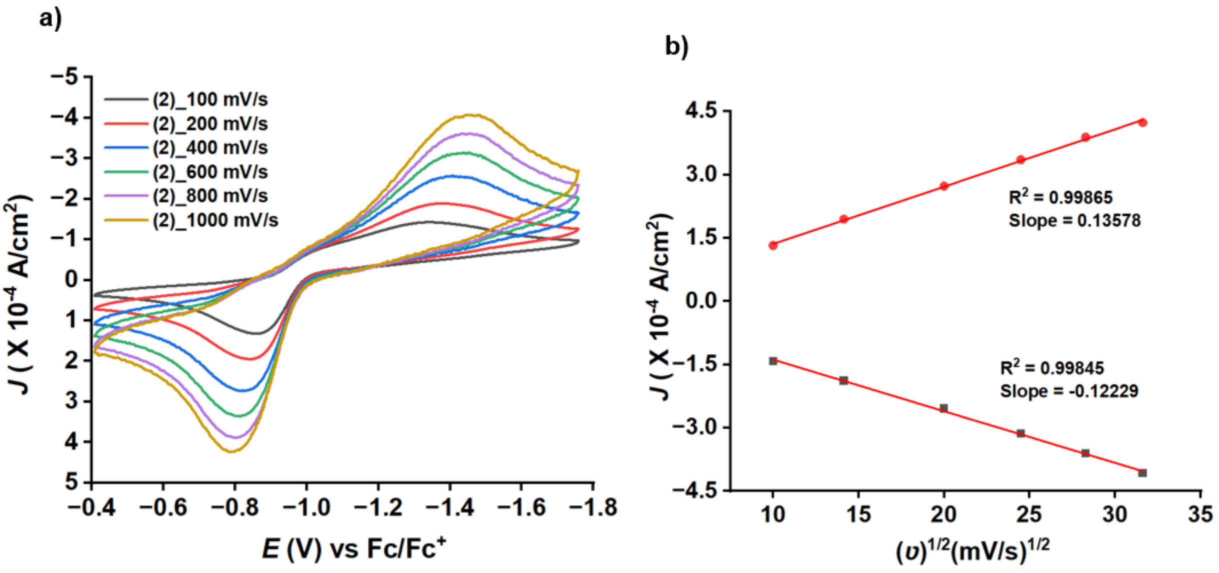

Figure S72. a) Cyclic Voltammogram of 0.5 mM [(L^2)BiCl$_2$] **(2)** varying scan rate with 0.1 M TBAPF$_6$ as a supporting electrolyte in acetonitrile. Condition: Working electrode glassy carbon (3 mm diameter), counter electrode Pt wire, and reference is Ag/AgCl. Data referenced to Fc/Fc$^+$ couple. **b)** Current density vs square root of scan rate plot for cathodic (black) and anodic (red) peaks in Cyclic Voltammogram of **[(L^2)BiCl$_2$] (2)**.

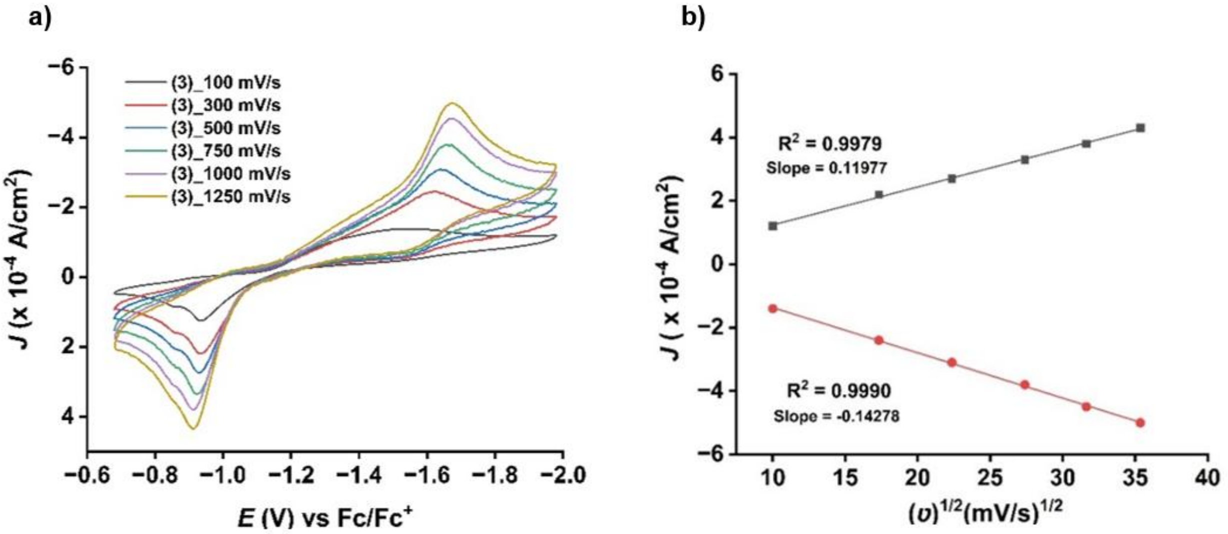

Figure S73. a) Cyclic Voltammogram of 0.5 mM [(L^3)BiCl$_2$] **(3)** varying scan rate with 0.1 M TBAPF$_6$ as a supporting electrolyte in acetonitrile. Condition: Working electrode glassy carbon (3 mm diameter), counter electrode Pt wire, and reference is Ag/AgCl. Data referenced to Fc/Fc$^+$ couple. **b)** Current density vs square root of scan rate plot for cathodic (red) and anodic (black) peaks in Cyclic Voltammogram of **[(L^3)BiCl$_2$] (3)**.

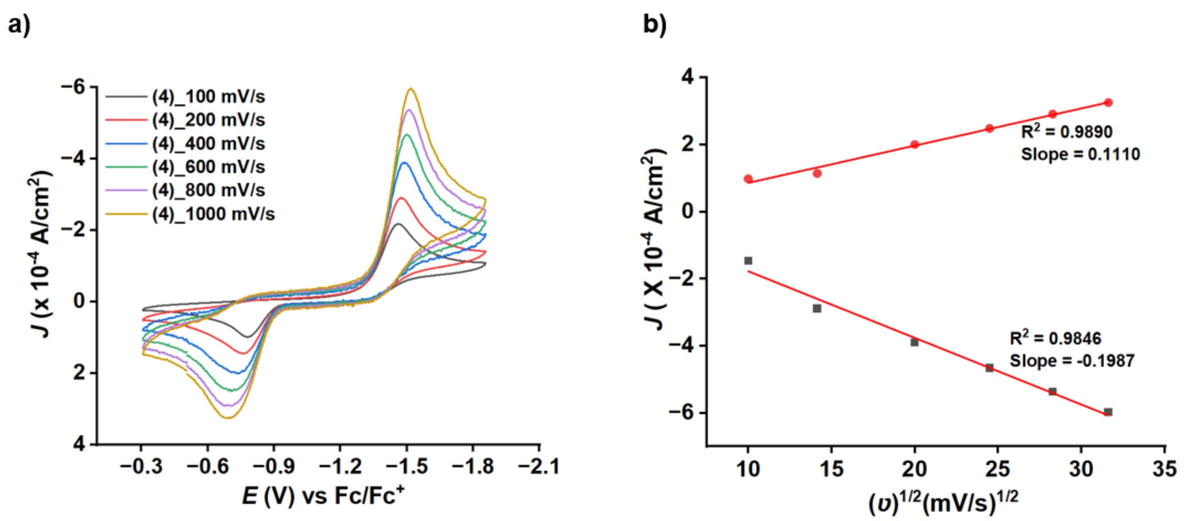

Figure S74. a) Cyclic Voltammogram of 0.5 mM [(L^4)$BiCl_2$] **(4)** varying scan rate with 0.1 M $TBAPF_6$ as a supporting electrolyte in acetonitrile. Condition: Working electrode glassy carbon (3 mm diameter), counter electrode Pt wire, and reference is Ag/AgCl. Data referenced to Fc/Fc^+ couple. **b)** Current density vs square root of scan rate plot for cathodic (black) and anodic (red) peaks in Cyclic Voltammogram of **[(L^4)$BiCl_2$] (4)**.

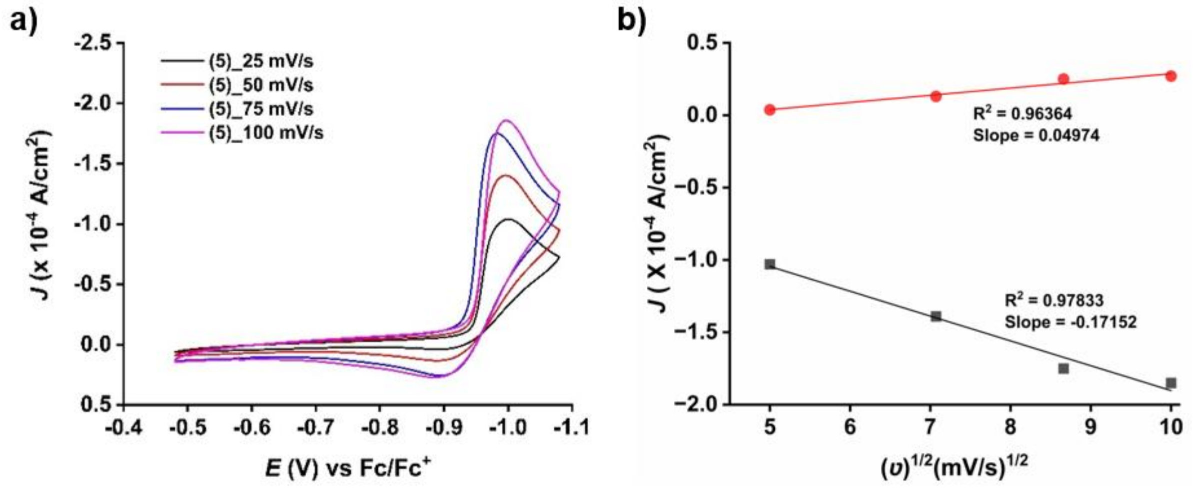

Figure S75. a) Cyclic Voltammogram of 0.5 mM [(L^5)$BiCl_2$] **(5)** varying scan rate with 0.1 M $TBAPF_6$ as a supporting electrolyte in acetonitrile. Condition: Working electrode glassy carbon (3 mm diameter), counter electrode Pt wire, and reference is Ag/AgCl. Data referenced to Fc/Fc^+ couple. **b)** Current density vs square root of scan rate plot for cathodic (black) and anodic (red) peaks in Cyclic Voltammogram of **[(L^5)$BiCl_2$] (5)**.

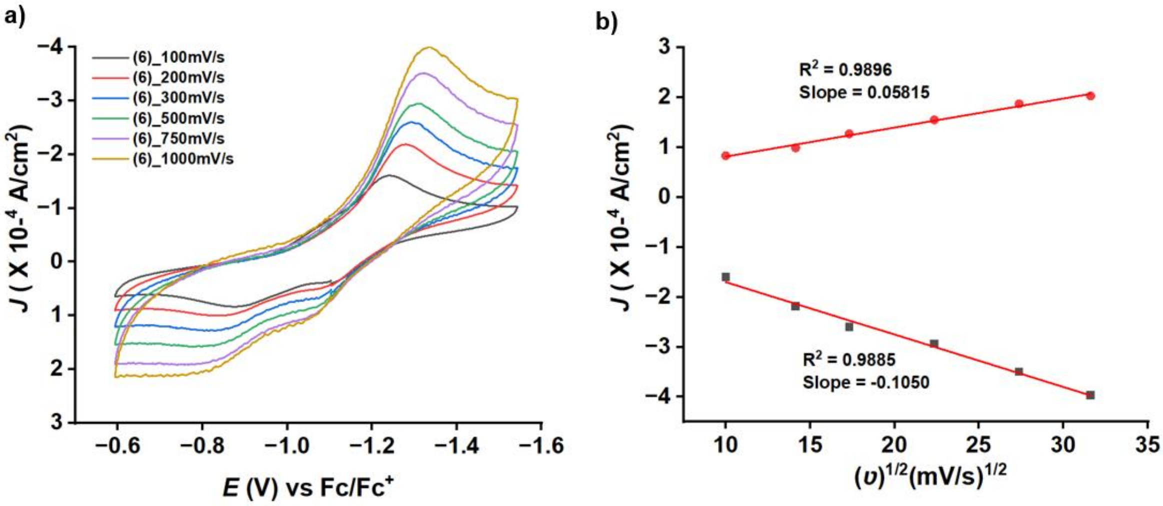

Figure S76. a) Cyclic Voltammogram of 0.5 mM $[(L^6)BiCl_2]$ **(6)** varying scan rate with 0.1 M $TBAPF_6$ as a supporting electrolyte in acetonitrile. Condition: Working electrode glassy carbon (3 mm diameter), counter electrode Pt wire, and reference is Ag/AgCl. Data referenced to Fc/Fc^+ couple. **b)** Current density vs square root of scan rate plot for cathodic (black) and anodic (red) peaks in Cyclic Voltammogram of **$[(L^6)BiCl_2]$ (6)**.

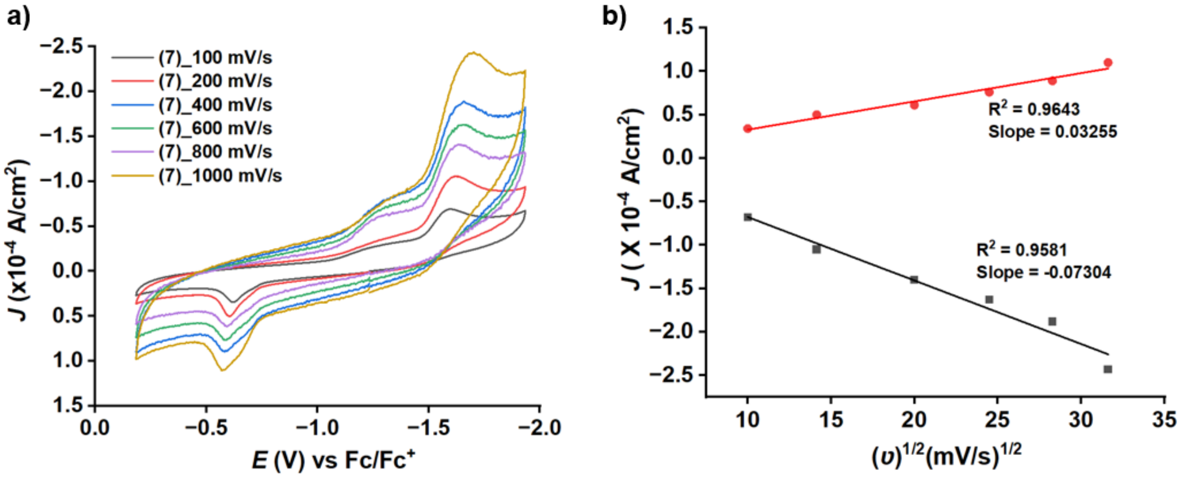

Figure S77. a) Cyclic Voltammogram of 0.5 mM $[(L^7)BiCl_2]$ **(7)** varying scan rate with 0.1 M $TBAPF_6$ as a supporting electrolyte in acetonitrile. Condition: Working electrode glassy carbon (3 mm diameter), counter electrode Pt wire, and reference is Ag/AgCl. Data referenced to Fc/Fc^+ couple. **b)** Current density vs square root of scan rate plot for cathodic (black) and anodic (red) peaks in Cyclic Voltammogram of **$[(L^7)BiCl_2]$ (7)**.

Diffusion Coefficient of Catalyst (VI)

For electrochemically reversible electron transfer processes involving freely diffusing redox species, the Randles–Sevcik equation describes how the peak current i_p (A) increases linearly with the square root of the scan rate υ (V s^{-1}), where n is the number of electrons transferred in the redox event, A (cm^2) is the electrode surface area (usually treated as the geometric surface area), D_o (cm^2 s^{-1}) is the diffusion coefficient of the oxidized analyte, and C^0 (mol cm^{-3}) is the bulk concentration of the analyte.[6] [7]

$$i_p = 0.446 nFAC^0 (nF\upsilon D_0/RT)^{1/2}$$

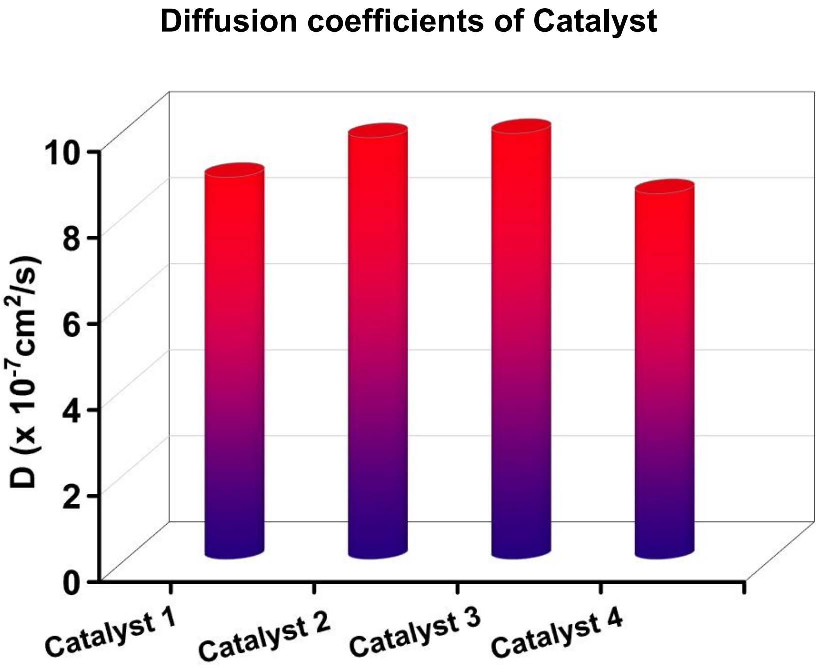

Figure S78. Plot of diffusion coefficient of catalyst (in Acetonitrile) at 0.1 M concentration of the proton sources in presence of 0.5 mM of catalyst [(L^1)$BiCl_2$] (**1**) b) [(L^2)$BiCl_2$] (**2**) c) [(L^3)$BiCl_2$] (**3**) d) [(L^4)$BiCl_2$] (**4**) at 298 K.

Table S10. Diffusion coefficient of catalysts

Catalyst	Diffusion Coefficient
[(L^1)$BiCl_2$] **(1)**	8.8 x 10^{-7}
[(L^2)$BiCl_2$] **(2)**	9.8 x 10^{-7}
[(L^3)$BiCl_2$] **(3)**	9.9 x 10^{-7}
[(L^4)$BiCl_2$] **(4)**	8.5 x 10^{-7}

Variation in pK_a of proton sources (VII)

The CVs for proton reduction studies of different acids with catalyst [(L[1])BiCl$_2$] (**1**) and the corresponding current density (*J*) vs concentration plots are shown in Figure S79 to S89.

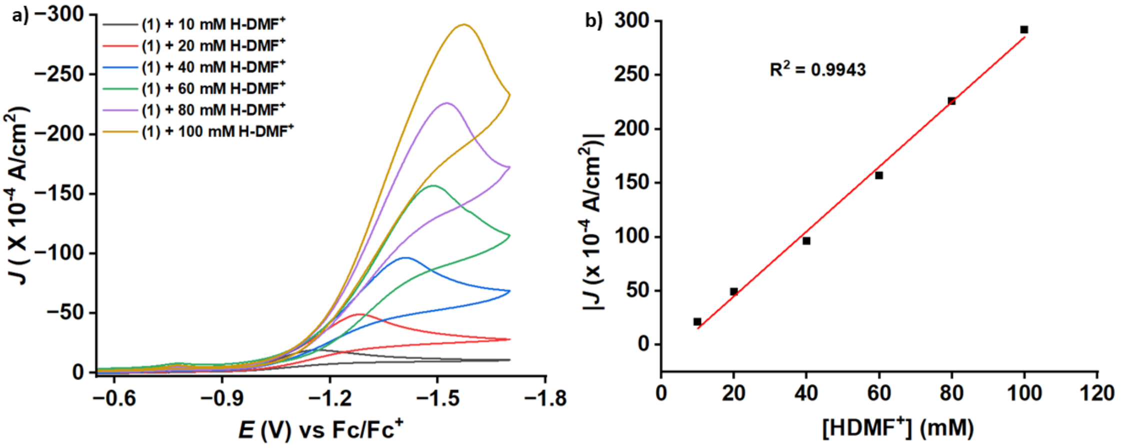

Figure S79. **a)** Cyclic Voltammogram of 0.5 mM [(L[1])BiCl$_2$] **(1)** with varying concentration of dimethylformamidium triflate (pK_a 6.1 in acetonitrile) with 0.1 M TBAPF$_6$ as a supporting electrolyte in acetonitrile; Scan rate = 100 mV/s. Condition: Working electrode glassy carbon (3 mm diameter), counter electrode Pt wire, and reference is Ag/AgCl. Data referenced to Fc/Fc$^+$ couple. **b)** Current density vs varying concentration of dimethylformamidium triflate plot for 0.5 mM [(L[1])BiCl$_2$] **(1)**.

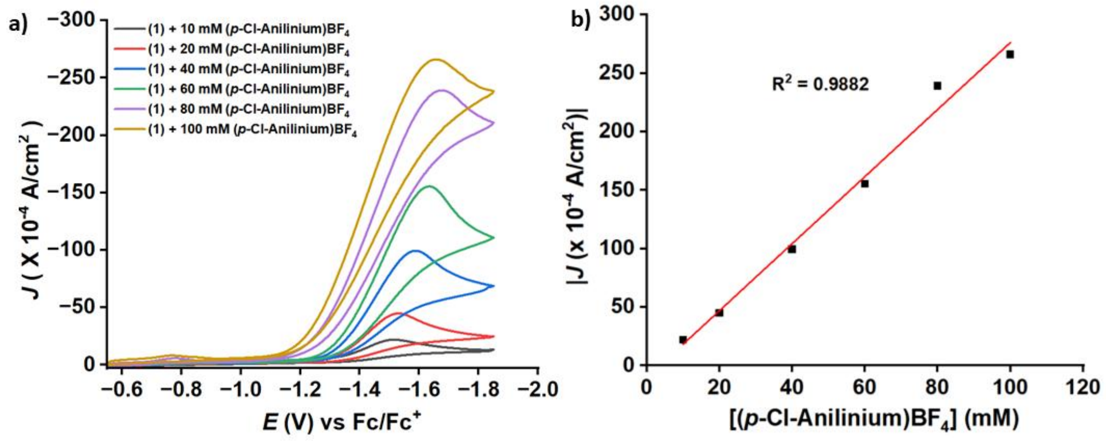

Figure S80. a) Cyclic Voltammogram of 0.5 mM [(L[1])BiCl$_2$] **(1)** with varying concentration of *p*-Chloroanilinium tetrafluoroborate (pK_a 9.7 in acetonitrile) with 0.1 M TBAPF$_6$ as a supporting electrolyte in acetonitrile; Scan rate = 100 mV/s. Condition: Working electrode glassy carbon (3 mm diameter), counter electrode Pt wire, and reference is Ag/AgCl. Data referenced to Fc/Fc$^+$ couple. **b)** Current density vs varying concentration of *p*-Chloroanilinium tetrafluoroborate plot for 0.5 mM [(L[1])BiCl$_2$] **(1)**.

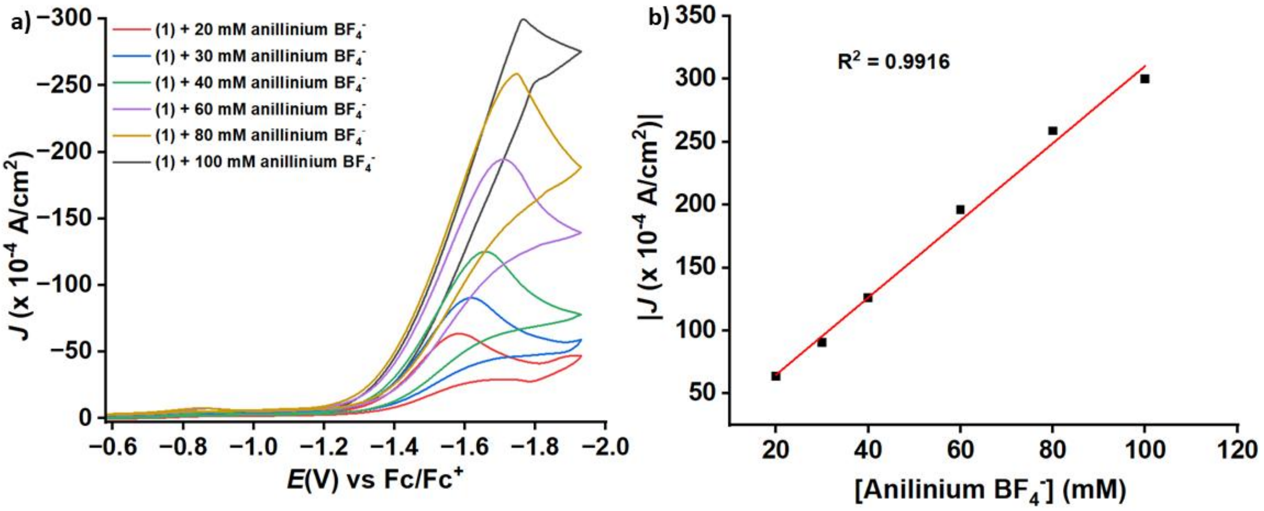

Figure S81. a) Cyclic Voltammogram of 0.5 mM [(L[1])BiCl$_2$] **(1)** with varying concentration of aniliniumBF$_4^-$ (pK_a 10.62 in acetonitrile) with 0.1 M TBAPF$_6$ as a supporting electrolyte in acetonitrile; Scan rate = 100 mV/s. Condition: Working electrode glassy carbon (3 mm diameter), counter electrode Pt wire, and reference is Ag/AgCl. Data referenced to Fc/Fc$^+$ couple. **b)** Current density vs varying concentration of anilinium BF$_4^-$ plot for 0.5 mM [(L[1])BiCl$_2$] **(1)**.

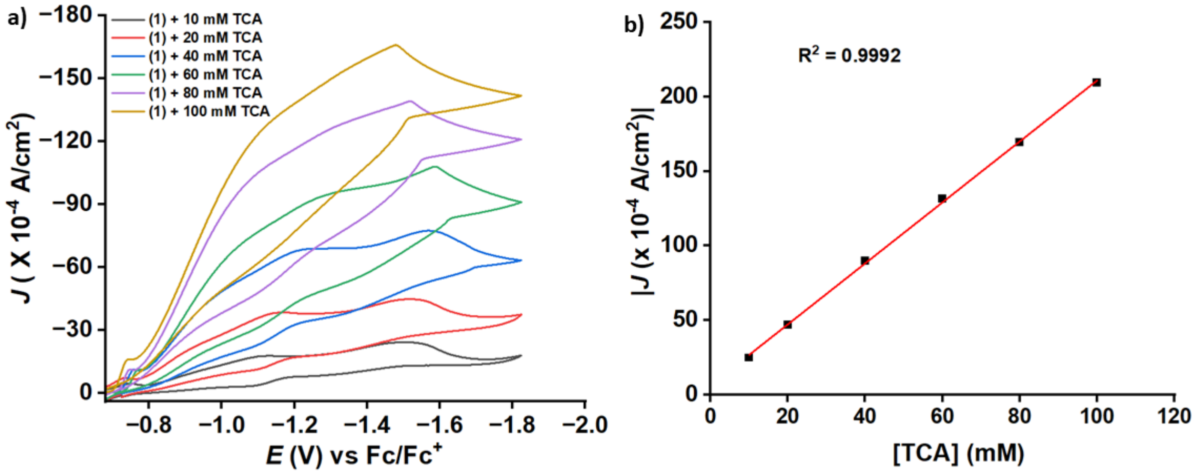

Figure S82. a) Cyclic Voltammogram of 0.5 mM [(L[1])BiCl$_2$] **(1)** with varying concentration of TCA (pK_a 10.75 in acetonitrile) with 0.1 M TBAPF$_6$ as a supporting electrolyte in acetonitrile; Scan rate = 100 mV/s. Condition: Working electrode glassy carbon (3 mm diameter), counter electrode Pt wire, and reference is Ag/AgCl. Data referenced to Fc/Fc$^+$ couple. **b)** Current density vs varying concentration of TCA plot for 0.5 mM [(L[1])BiCl$_2$] **(1)**.

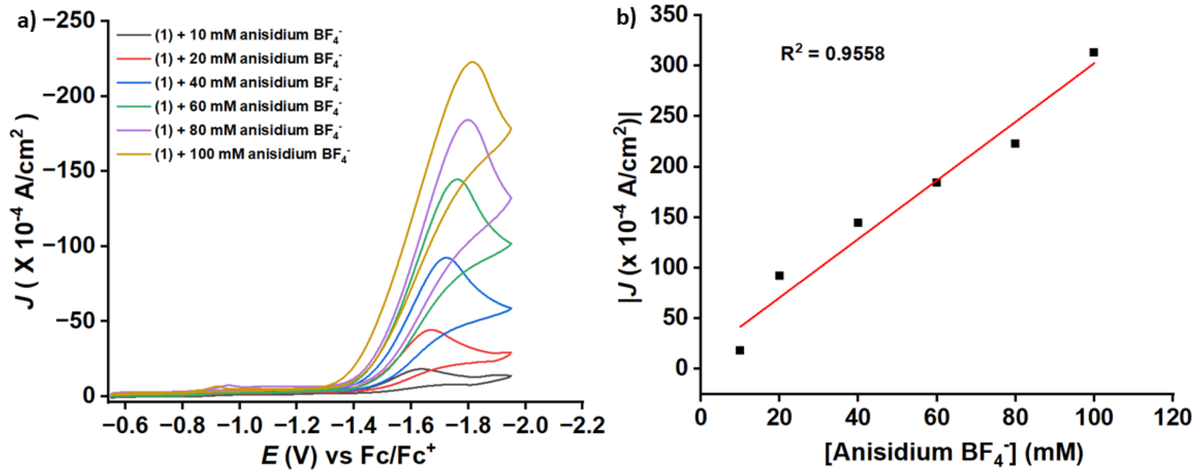

Figure S83. a) Cyclic Voltammogram of 0.5 mM [(L^1)BiCl$_2$] **(1)** with varying concentration of anisidium BF_4^- (pK_a 11.86 in acetonitrile) with 0.1 M $TBAPF_6$ as a supporting electrolyte in acetonitrile; Scan rate = 100 mV/s. Condition: Working electrode glassy carbon (3 mm diameter), counter electrode Pt wire, and reference is Ag/AgCl. Data referenced to Fc/Fc^+ couple. **b)** Current density vs varying concentration of anisidium BF_4^- plot for 0.5 mM [(L^1)BiCl$_2$] **(1)**.

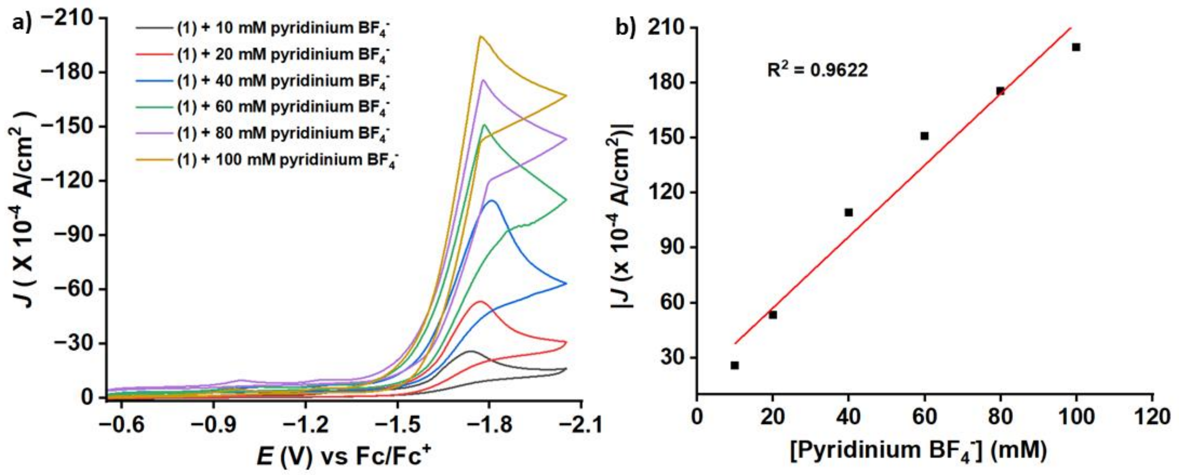

Figure 84. a) Cyclic Voltammogram of 0.5 mM [(L^1)BiCl$_2$] **(1)** with varying concentrations of Pyridinium BF_4^- (pK_a 12.53 in acetonitrile) with 0.1 M $TBAPF_6$ as a supporting electrolyte in acetonitrile; Scan rate = 100 mV/s. Condition: Working electrode glassy carbon (3 mm diameter), counter electrode Pt wire, and reference is Ag/AgCl. Data referenced to Fc/Fc^+ couple. **b)** Current density vs varying concentration of Pyridinium BF_4^- plot for 0.5 mM [(L^1)BiCl$_2$] **(1)**.

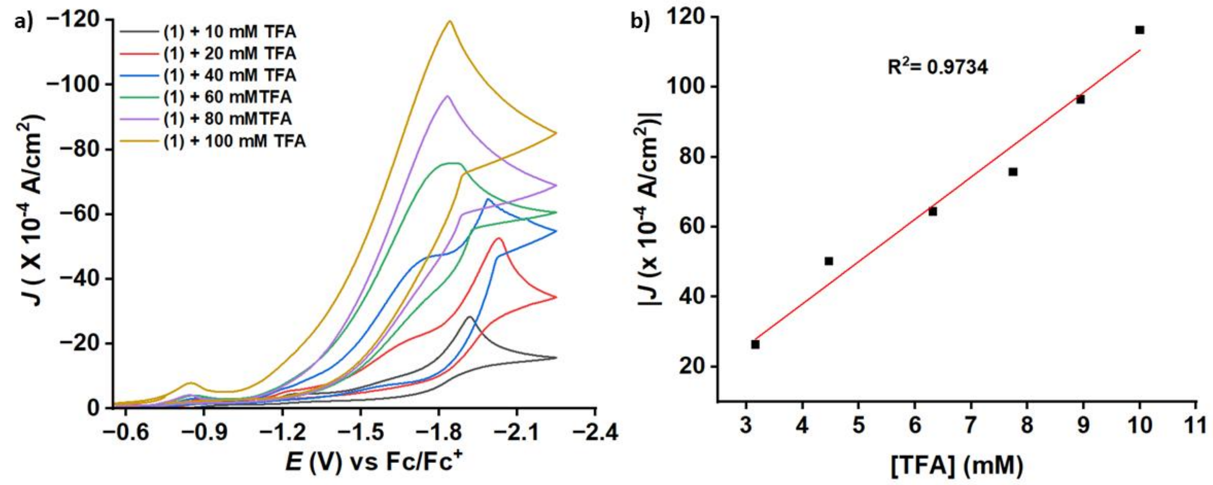

Figure S85. a) Cyclic Voltammogram of 0.5 mM [(L^1)BiCl$_2$] **(1)** with varying concentration of TFA (pK_a 12.65 in acetonitrile) with 0.1 M TBAPF$_6$ as a supporting electrolyte in acetonitrile; Scan rate = 100 mV/s. Condition: Working electrode glassy carbon (3 mm diameter), counter electrode Pt wire, and reference is Ag/AgCl. Data referenced to Fc/Fc$^+$ couple. **b)** Current density vs varying concentration of TFA plot for 0.5 mM [(L^1)BiCl$_2$] **(1)**.

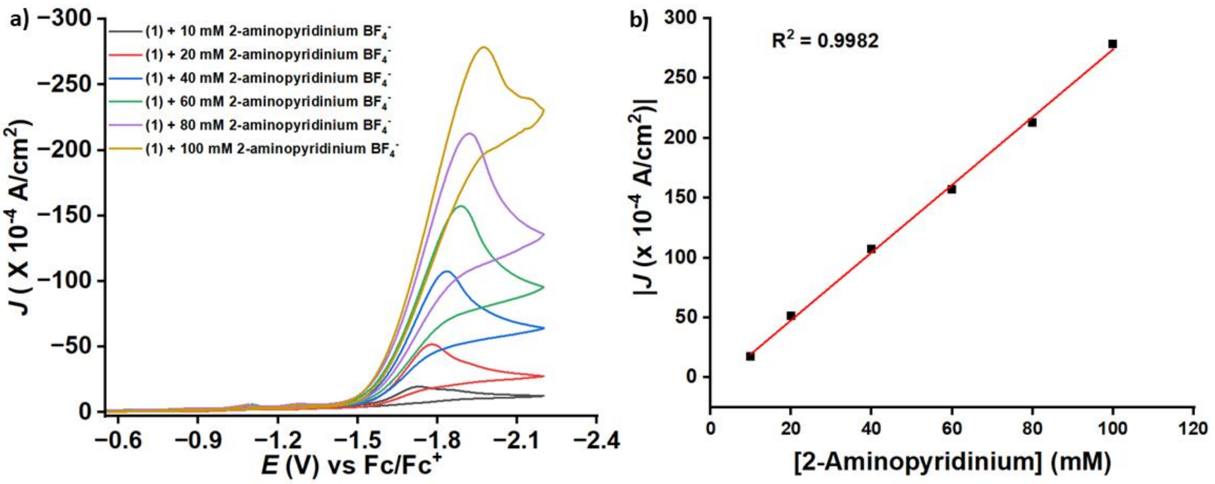

Figure S86. a) Cyclic Voltammogram of 0.5 mM [(L^1)BiCl$_2$] **(1)** with varying concentration of 2-aminopyridinium BF$_4^-$ (pK_a 14.47 in acetonitrile) with 0.1 M TBAPF$_6$ as a supporting electrolyte in acetonitrile; Scan rate = 100 mV/s. Condition: Working electrode glassy carbon (3 mm diameter), counter electrode Pt wire, and reference is Ag/AgCl. Data referenced to Fc/Fc$^+$ couple. **b)** Current density vs varying concentration of 2-aminopyridinium BF$_4^-$ plot for 0.5 mM [(L^1)BiCl$_2$] **(1)**.

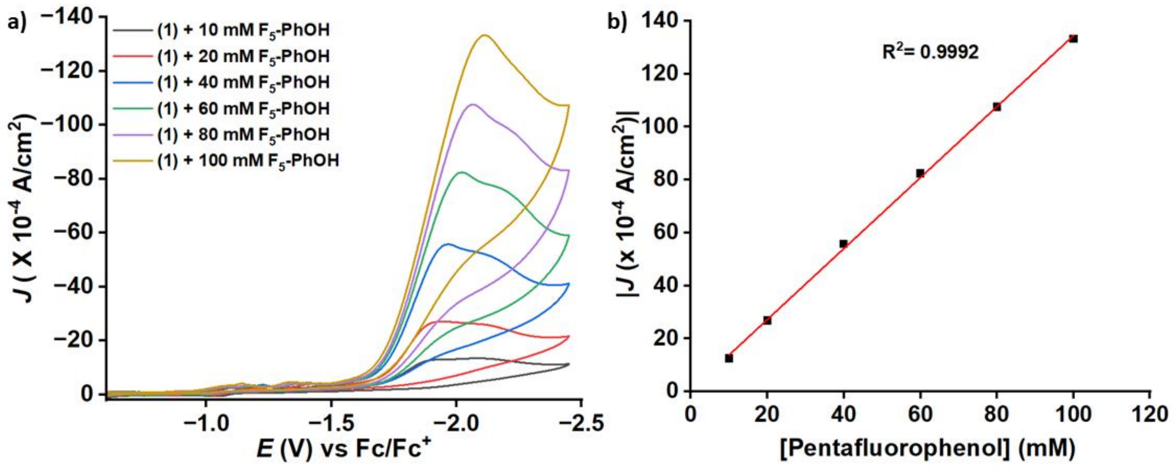

Figure S87. a) Cyclic Voltammogram of 0.5 mM [(L[1])BiCl$_2$] **(1)** with varying concentration of pentafluorophenol (pK_a 20.1 in acetonitrile) with 0.1 M TBAPF$_6$ as a supporting electrolyte in acetonitrile; Scan rate = 100 mV/s. Condition: Working electrode glassy carbon (3 mm diameter), counter electrode Pt wire, and reference is Ag/AgCl. Data referenced to Fc/Fc$^+$ couple. **b)** Current density vs varying concentration of pentafluorophenol plot for 0.5 mM [(L[1])BiCl$_2$] **(1)**.

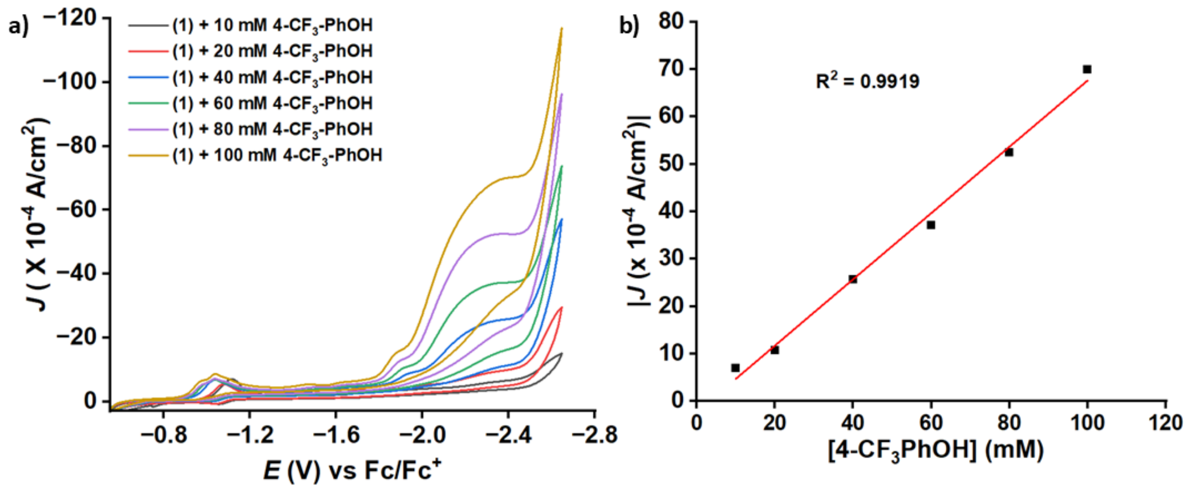

Figure S88. a) Cyclic Voltammogram of 0.5 mM [(L[1])BiCl$_2$] **(1)** with varying concentration of 4-trifluoromethylphenol (pK_a 25.54 in acetonitrile) with 0.1 M TBAPF$_6$ as a supporting electrolyte in acetonitrile; Scan rate = 100 mV/s. Condition: Working electrode glassy carbon (3 mm diameter), counter electrode Pt wire, and reference is Ag/AgCl. Data referenced to Fc/Fc$^+$ couple. **b)** Current density vs varying concentration of 4-trifluoromethylphenol plot for 0.5 mM [(L[1])BiCl$_2$] **(1)**.

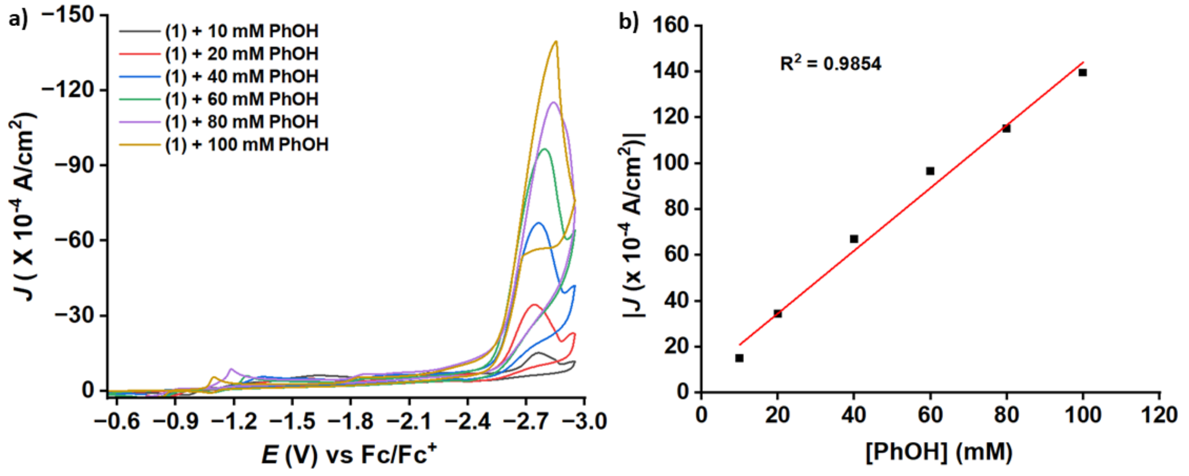

Figure S89. a) Cyclic Voltammogram of 0.5 mM [(L[1])BiCl$_2$] **(1)** with varying concentration of Phenol (pK_a 29.4 in acetonitrile) with 0.1 M TBAPF$_6$ as a supporting electrolyte in acetonitrile; Scan rate = 100 mV/s. Condition: Working electrode glassy carbon (3 mm diameter), counter electrode Pt wire, and reference is Ag/AgCl. Data referenced to Fc/Fc$^+$ couple. **b)** Current density vs varying concentration of Phenol plot for 0.5 mM [(L[1])BiCl$_2$] **(1)**.

The CVs for proton reduction studies of different acids with catalyst **[(L^2)BiCl$_2$] (2)** and the corresponding current density (J) vs concentration plots are shown in Figure S90 to S100.

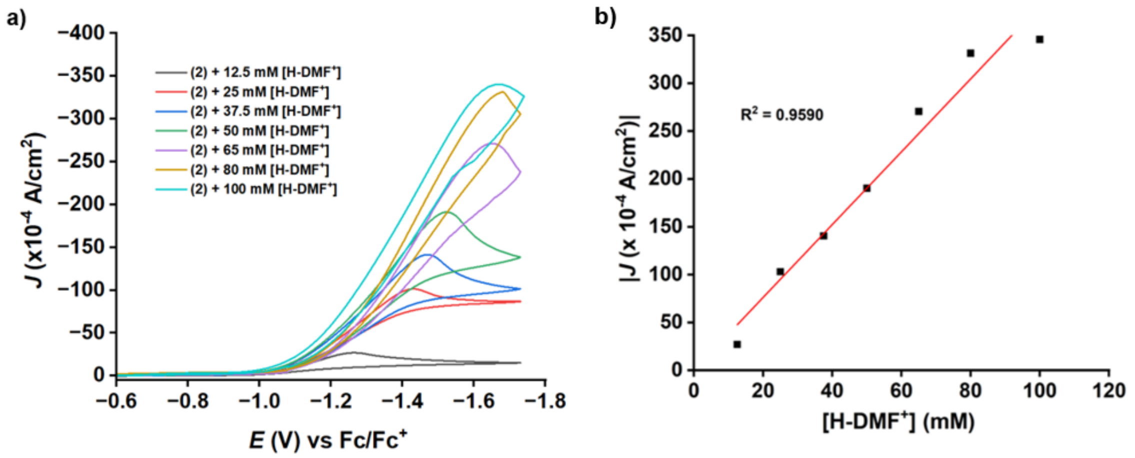

Figure S90. a) Cyclic Voltammogram of 0.5 mM [(L^2)BiCl$_2$] **(2)** with varying concentration of dimethylformamidium triflate (pK_a 6.1 in acetonitrile) with 0.1 M TBAPF$_6$ as a supporting electrolyte in acetonitrile; Scan rate = 100 mV/s. Condition: Working electrode glassy carbon (3 mm diameter), counter electrode Pt wire, and reference is Ag/AgCl. Data referenced to Fc/Fc$^+$ couple. **b)** Current density vs varying concentration of dimethylformamidium triflate plot for 0.5 mM [(L^2)BiCl$_2$] **(2)**.

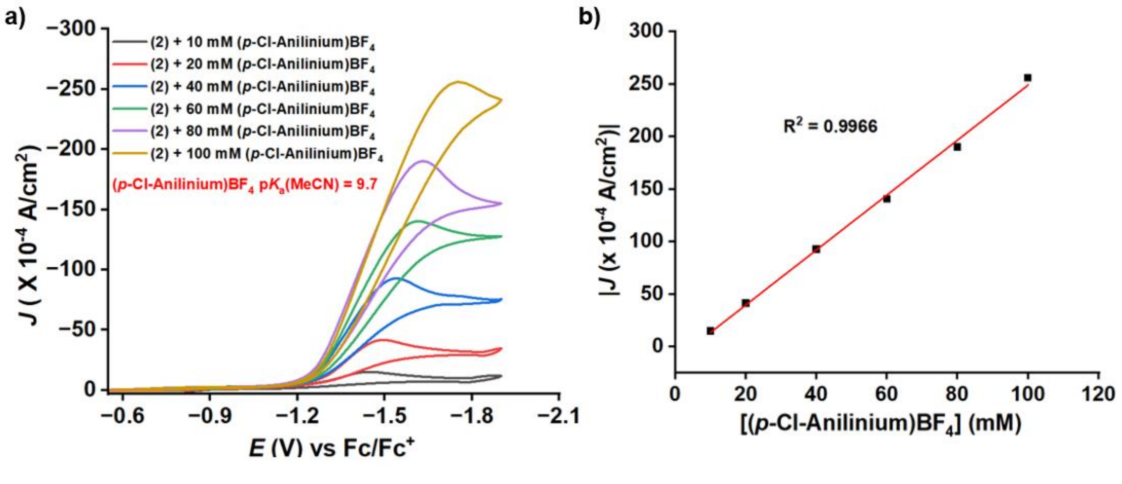

Figure S91. a) Cyclic Voltammogram of 0.5 mM [(L^2)BiCl$_2$] **(2)** with varying concentration of para-Chloroanilinium tetrafluoroborate (pK_a 9.7 in acetonitrile) with 0.1 M TBAPF$_6$ as a supporting electrolyte in acetonitrile; Scan rate = 100 mV/s. Condition: Working electrode glassy carbon (3 mm diameter), counter electrode Pt wire, and reference is Ag/AgCl. Data referenced to Fc/Fc$^+$ couple. **b)** Current density vs varying concentration of para-Chloroanilinium tetrafluoroborate plot for 0.5 mM [(L^2)BiCl$_2$] **(2)**.

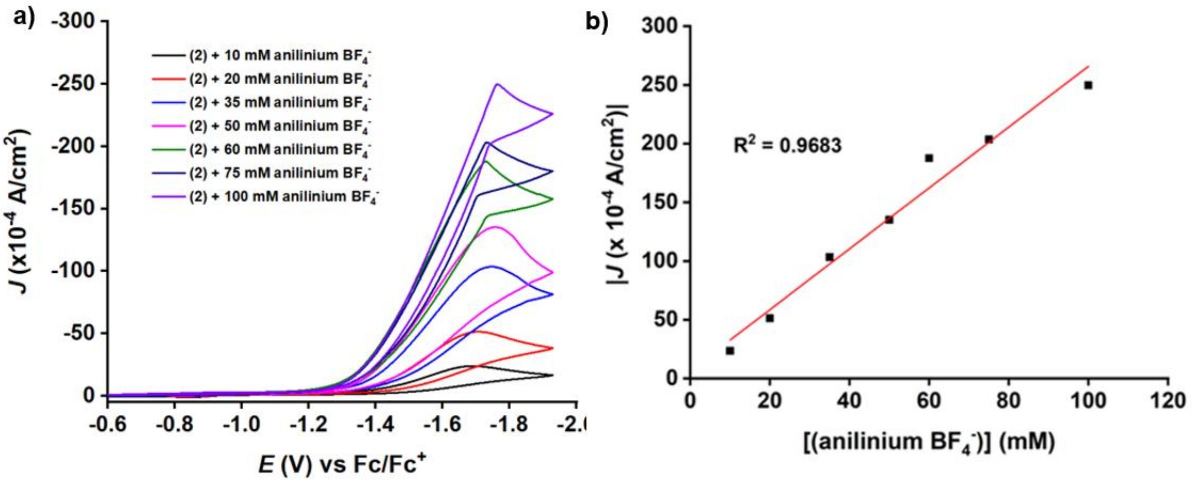

Figure S92. a) Cyclic Voltammogram of 0.5 mM [(L^2)$BiCl_2$] **(2)** with varying concentration of anilinium BF_4^- (pK_a 10.62 in acetonitrile) with 0.1 M $TBAPF_6$ as a supporting electrolyte in acetonitrile; Scan rate = 100 mV/s. Condition: Working electrode glassy carbon (3 mm diameter), counter electrode Pt wire, and reference is Ag/AgCl. Data referenced to Fc/Fc^+ couple. **b)** Current density vs varying concentration of anilinium BF_4^- plot for 0.5 mM [(L^2)$BiCl_2$] **(2)**.

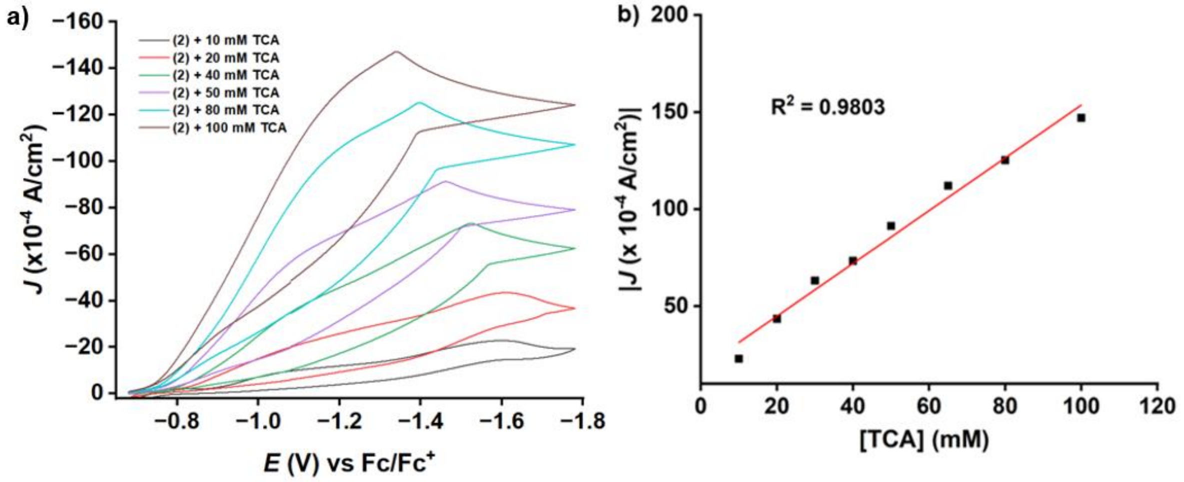

Figure S93. a) Cyclic Voltammogram of 0.5 mM [(L^2)$BiCl_2$] **(2)** with varying concentration of TCA (pK_a 10.75 in acetonitrile) with 0.1 M $TBAPF_6$ as a supporting electrolyte in acetonitrile; Scan rate = 100 mV/s. Condition: Working electrode glassy carbon (3 mm diameter), counter electrode Pt wire, and reference is Ag/AgCl. Data referenced to Fc/Fc^+ couple. **b)** Current density vs varying concentration of TCA plot for 0.5 mM [(L^2)$BiCl_2$] **(2)**.

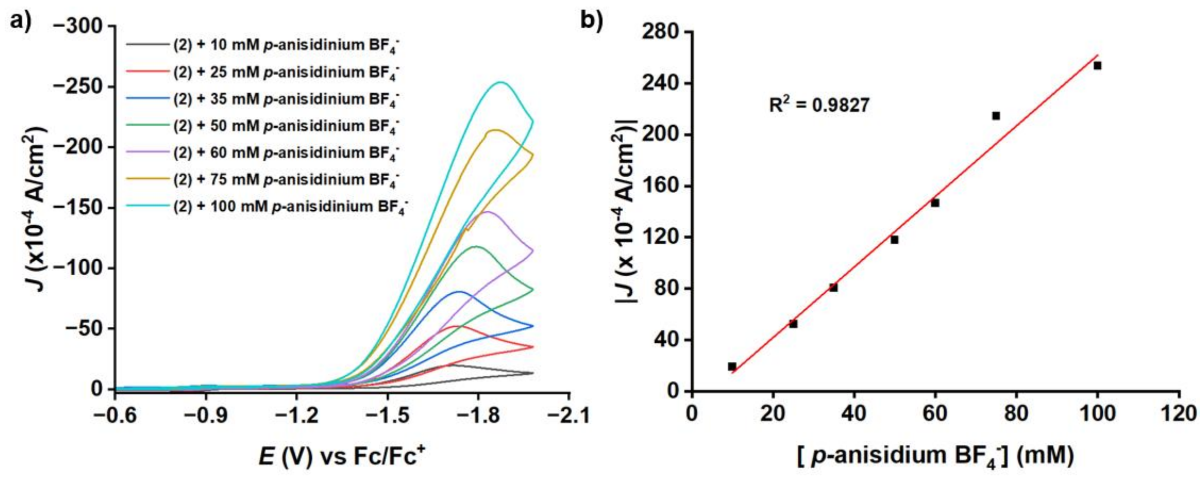

Figure S94. a) Cyclic Voltammogram of 0.5 mM [(L^2)$BiCl_2$] **(2)** with varying concentration of *p*-anisidium BF_4^- (pK_a 11.86 in acetonitrile) with 0.1 M $TBAPF_6$ as a supporting electrolyte in acetonitrile; Scan rate = 100 mV/s. Condition: Working electrode glassy carbon (3 mm diameter), counter electrode Pt wire, and reference is Ag/AgCl. Data referenced to Fc/Fc^+ couple. **b)** Current density vs varying concentration of anisidium BF_4^- plot for 0.5 mM [(L^2)$BiCl_2$] **(2)**.

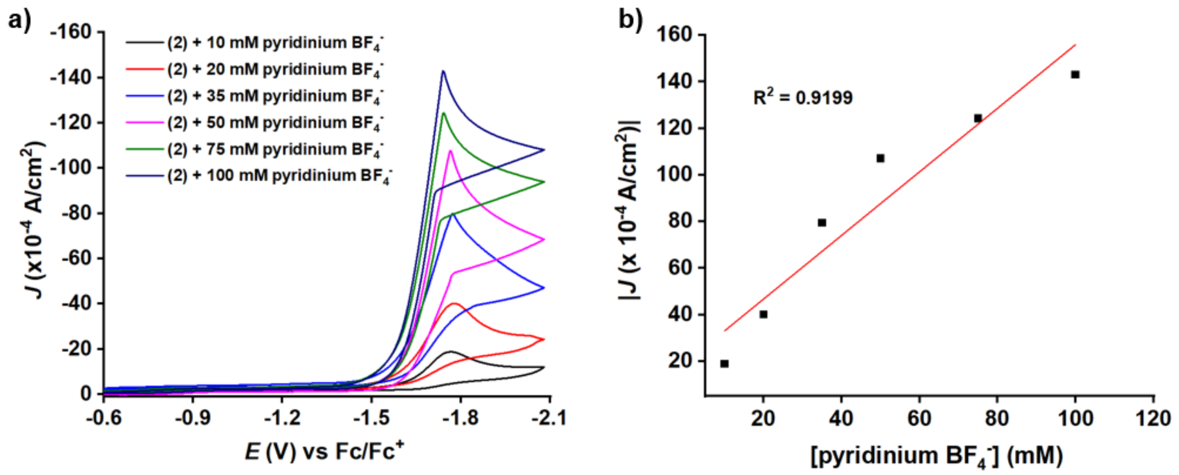

Figure S95. a) Cyclic Voltammogram of 0.5 mM [(L^2)$BiCl_2$] **(2)** with varying concentrations of Pyridinium BF_4^- (pK_a 12.53 in acetonitrile) with 0.1 M $TBAPF_6$ as a supporting electrolyte in acetonitrile; Scan rate = 100 mV/s. Condition: Working electrode glassy carbon (3 mm diameter), counter electrode Pt wire, and reference is Ag/AgCl. Data referenced to Fc/Fc^+ couple. **b)** Current density vs varying concentration of Pyridinium BF_4^- plot for 0.5 mM [(L^2)$BiCl_2$] **(2)**.

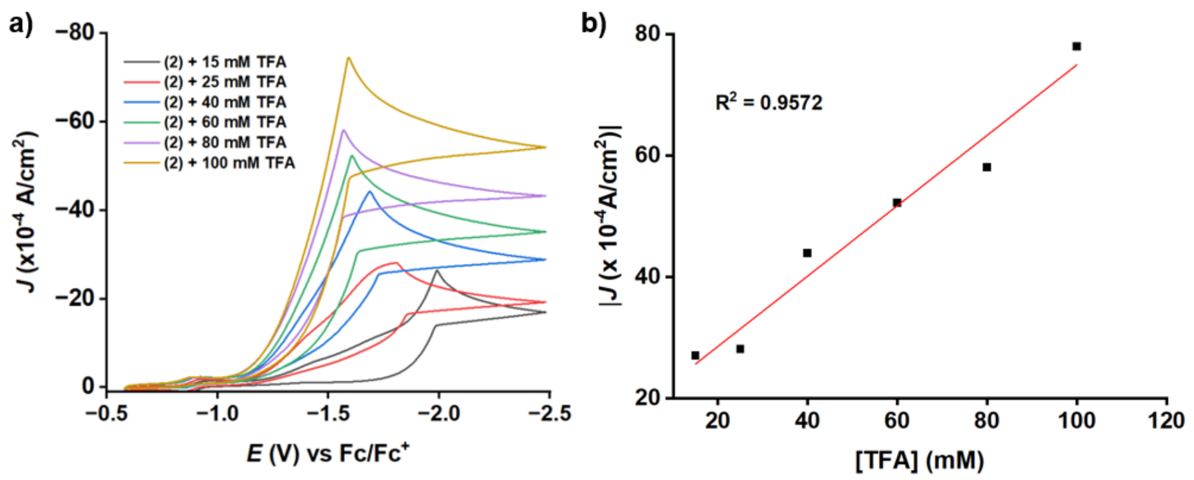

Figure S96. a) Cyclic Voltammogram of 0.5 mM [(L^2)BiCl$_2$] **(2)** with varying concentration of TFA (pK_a 12.65 in acetonitrile) with 0.1 M TBAPF$_6$ as a supporting electrolyte in acetonitrile; Scan rate = 100 mV/s. Condition: Working electrode glassy carbon (3 mm diameter), counter electrode Pt wire, and reference is Ag/AgCl. Data referenced to Fc/Fc$^+$ couple. **b)** Current density vs varying concentration of TFA plot for 0.5 mM [(L^2)BiCl$_2$] **(2)**.

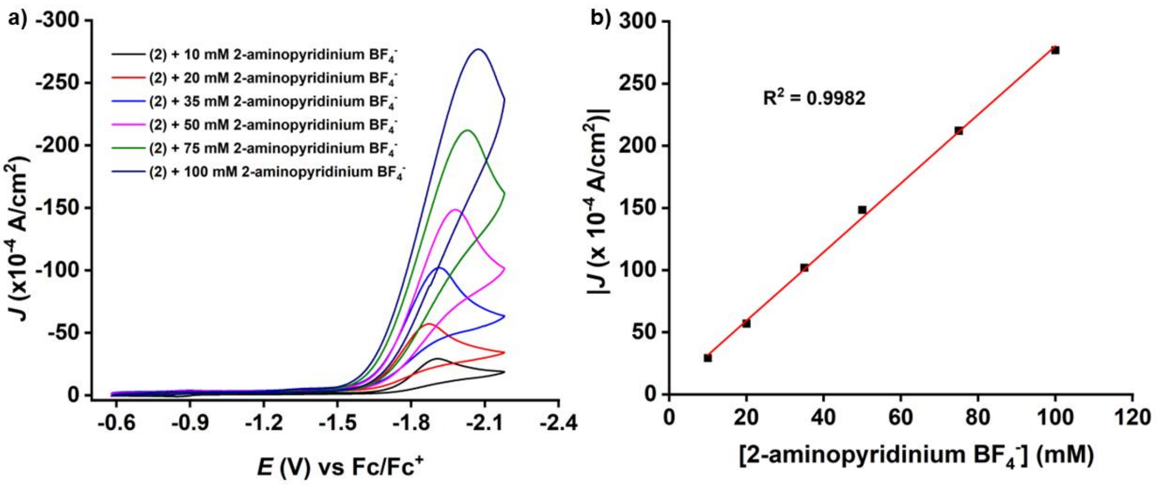

Figure S97. a) Cyclic Voltammogram of 0.5 mM [(L^2)BiCl$_2$] **(2)** with varying concentration of 2-aminopyridinium BF_4^- (pK_a 14.47 in acetonitrile) with 0.1 M TBAPF$_6$ as a supporting electrolyte in acetonitrile; Scan rate = 100 mV/s. Condition: Working electrode glassy carbon (3 mm diameter), counter electrode Pt wire, and reference is Ag/AgCl. Data referenced to Fc/Fc$^+$ couple. **b)** Current density vs varying concentration of 2-aminopyridinium BF_4^- plot for 0.5 mM [(L^2)BiCl$_2$] **(2)**.

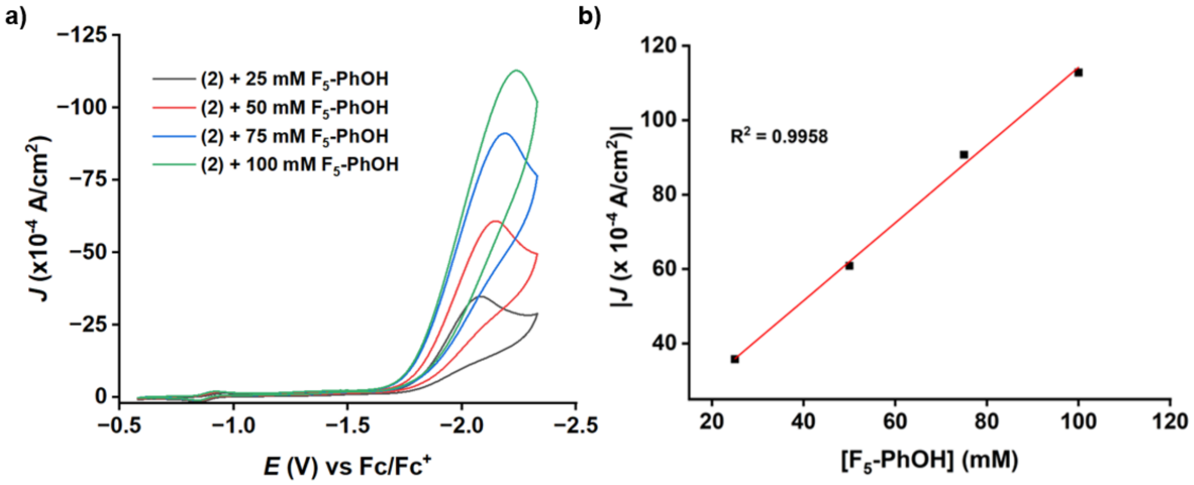

Figure S98. a) Cyclic Voltammogram of 0.5 mM [(L^2)$BiCl_2$] **(2)** with varying concentration of pentafluorophenol (pK_a 20.1 in acetonitrile) with 0.1 M $TBAPF_6$ as a supporting electrolyte in acetonitrile; Scan rate = 100 mV/s. Condition: Working electrode glassy carbon (3 mm diameter), counter electrode Pt wire, and reference is Ag/AgCl. Data referenced to Fc/Fc^+ couple. **b)** Current density vs varying concentration of pentafluorophenol plot for 0.5 mM [(L^2)$BiCl_2$] **(2)**.

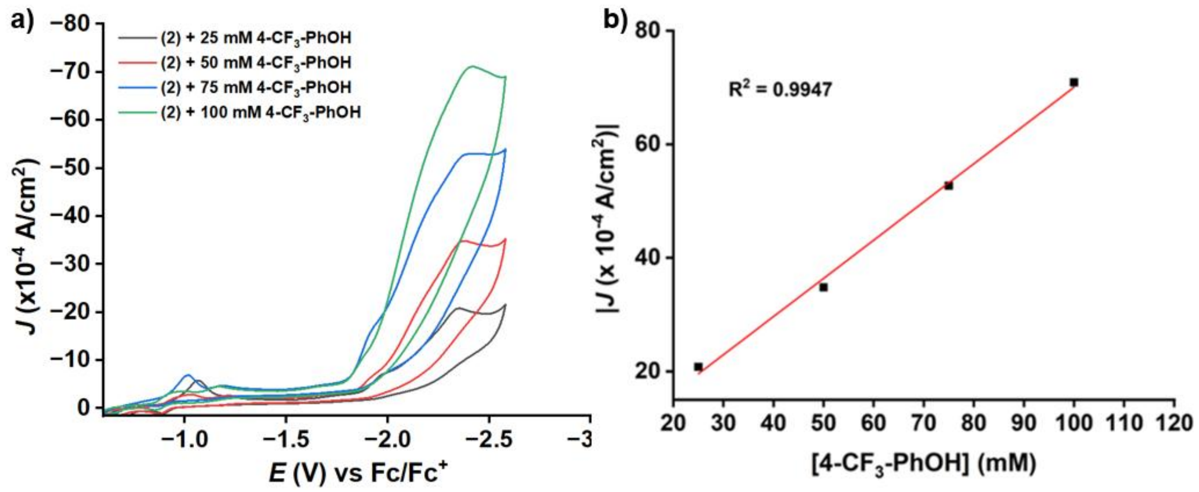

Figure S99. a) Cyclic Voltammogram of 0.5 mM [(L^2)$BiCl_2$] **(2)** with varying concentration of 4-trifluoromethylphenol (pK_a 25.54 in acetonitrile) with 0.1 M $TBAPF_6$ as a supporting electrolyte in acetonitrile; Scan rate = 100 mV/s. Condition: Working electrode glassy carbon (3 mm diameter), counter electrode Pt wire, and reference is Ag/AgCl. Data referenced to Fc/Fc^+ couple. **b)** Current density vs varying concentration of 4-trifluoromethylphenol plot for 0.5 mM [(L^2)$BiCl_2$] **(2)**.

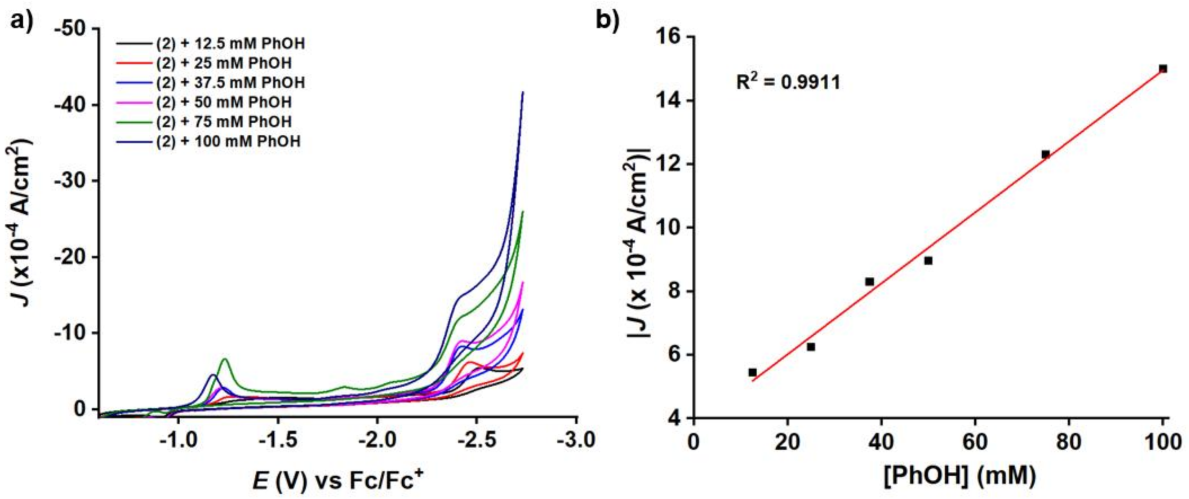

Figure S100. a) Cyclic Voltammogram of 0.5 mM [(L^2)BiCl$_2$] **(2)** with varying concentration of Phenol (pK_a 29.4 in acetonitrile) with 0.1 M $TBAPF_6$ as a supporting electrolyte in acetonitrile; Scan rate = 100 mV/s. Condition: Working electrode glassy carbon (3 mm diameter), counter electrode Pt wire, and reference is Ag/AgCl. Data referenced to Fc/Fc$^+$ couple. **b)** Current density vs varying concentration of Phenol plot for 0.5 mM [(L^2)BiCl$_2$] **(2)**.

The CVs for proton reduction studies of acids with catalyst **[(L^3)$BiCl_2$] (3)** the corresponding current density (*J*) vs concentration plots are shown in Fig. S103 to S111.

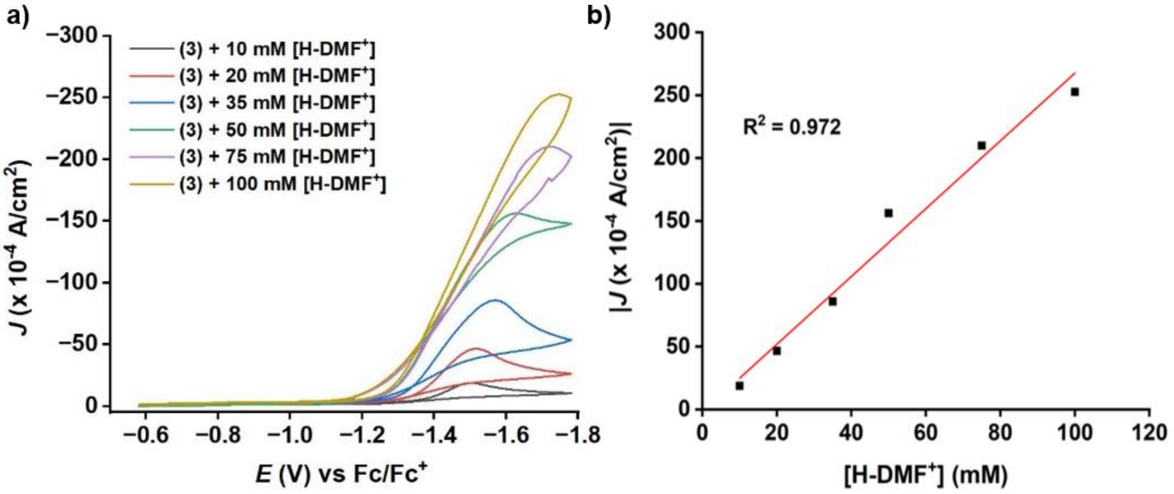

Figure S101. a) Cyclic Voltammogram of 0.5 mM [(L^3)$BiCl_2$] **(3)** with varying concentration of dimethylformamidium triflate (pK_a 6.1 in acetonitrile) with 0.1 M $TBAPF_6$ as a supporting electrolyte in acetonitrile; Scan rate = 100 mV/s. Condition: Working electrode glassy carbon (3 mm diameter), counter electrode Pt wire, and reference is Ag/AgCl. Data referenced to Fc/Fc^+ couple. **b)** Current density vs varying concentration of dimethylformamidium triflate plot for 0.5 mM [(L^3)$BiCl_2$] **(3)**.

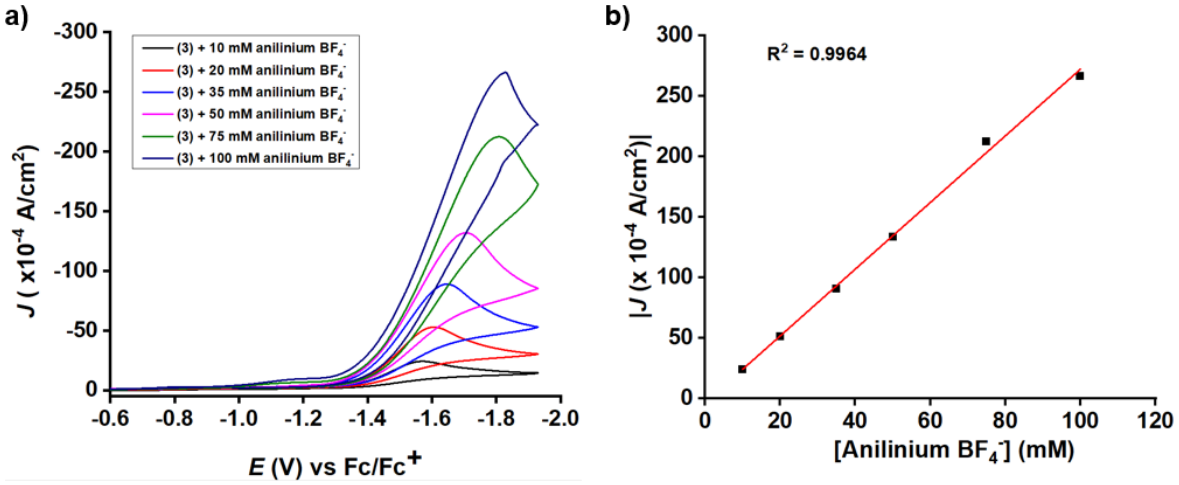

Figure S102. a) Cyclic Voltammogram of 0.5 mM [(L^3)$BiCl_2$] **(3)** with varying concentration of anilinium BF_4^- (pK_a 10.62 in acetonitrile) with 0.1 M $TBAPF_6$ as a supporting electrolyte in acetonitrile; Scan rate = 100 mV/s. Condition: Working electrode glassy carbon (3 mm diameter), counter electrode Pt wire, and reference is Ag/AgCl. Data referenced to Fc/Fc^+ couple. **b)** Current density vs varying concentration of anilinium BF_4^- plot for 0.5 mM [(L^3)$BiCl_2$] **(3)**.

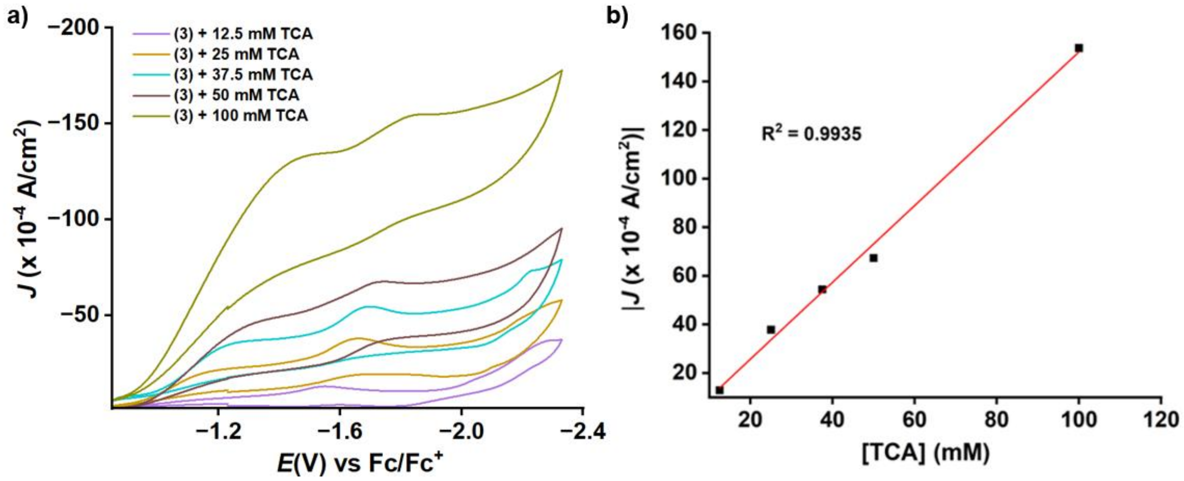

Figure S103. a) Cyclic Voltammogram of 0.5 mM [(L^3)$BiCl_2$] **(3)** with varying concentration of TCA (pK_a 10.75 in acetonitrile) with 0.1 M $TBAPF_6$ as a supporting electrolyte in acetonitrile; Scan rate = 100 mV/s. Condition: Working electrode glassy carbon (3 mm diameter), counter electrode Pt wire, and reference is Ag/AgCl. Data referenced to Fc/Fc^+ couple. **b)** Current density vs varying concentration of TCA plot for 0.5 mM [(L^3)$BiCl_2$] **(3)**.

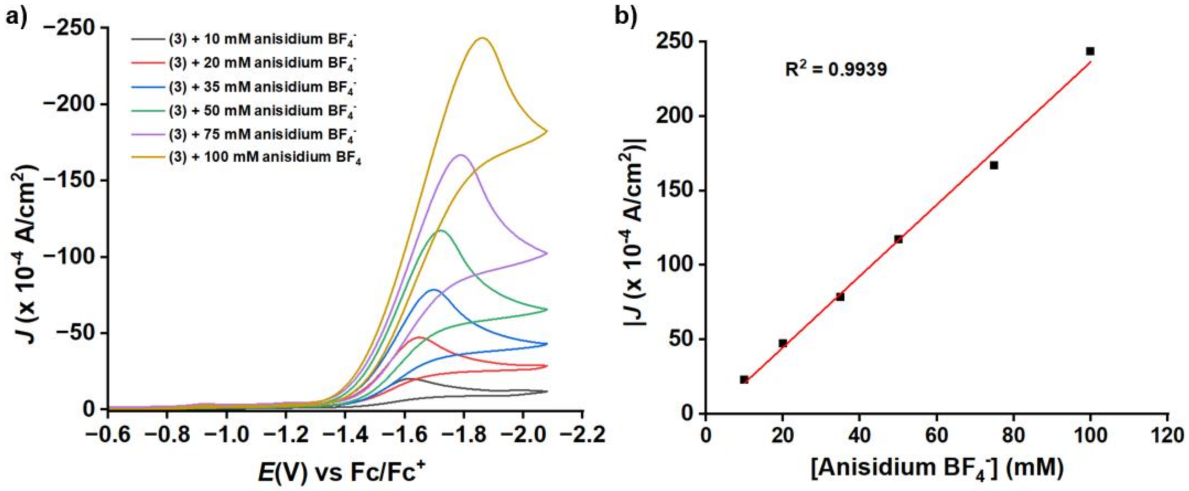

Figure S104 a) Cyclic Voltammogram of 0.5 mM [(L^3)$BiCl_2$] **(3)** with varying concentration of *p*-anisidium BF_4^- (pK_a 11.86 in acetonitrile) with 0.1 M $TBAPF_6$ as a supporting electrolyte in acetonitrile; Scan rate = 100 mV/s. Condition: Working electrode glassy carbon (3 mm diameter), counter electrode Pt wire, and reference is Ag/AgCl. Data referenced to Fc/Fc^+ couple. **b)** Current density vs varying concentration of anisidium BF_4^- plot for 0.5 mM [(L^3)$BiCl_2$] **(3)**.

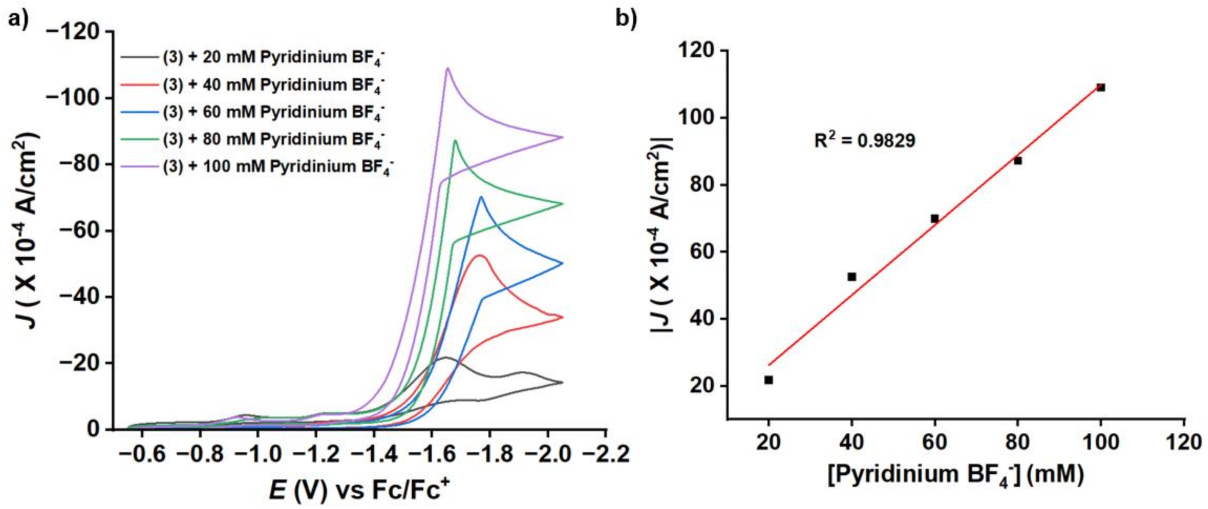

Figure S105. a) Cyclic Voltammogram of 0.5 mM [(L^3)$BiCl_2$] **(3)** with varying concentrations of Pyridinium BF_4^- (pK_a 12.53 in acetonitrile) with 0.1 M $TBAPF_6$ as a supporting electrolyte in acetonitrile; Scan rate = 100 mV/s. Condition: Working electrode glassy carbon (3 mm diameter), counter electrode Pt wire, and reference is Ag/AgCl. Data referenced to Fc/Fc^+ couple. **b)** Current density vs varying concentration of Pyridinium BF_4^- plot for 0.5 mM [(L^3)$BiCl_2$] **(3)**.

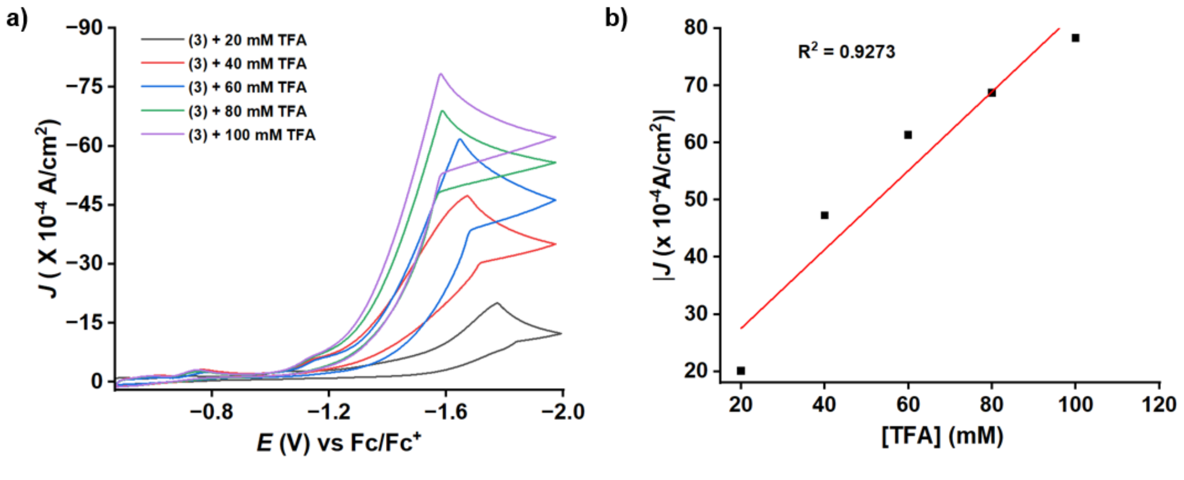

Figure S106. a) Cyclic Voltammogram of 0.5 mM [(L^3)$BiCl_2$] **(3)** with varying concentration of TFA (pK_a 12.65 in acetonitrile) with 0.1 M $TBAPF_6$ as a supporting electrolyte in acetonitrile; Scan rate = 100 mV/s. Condition: Working electrode glassy carbon (3 mm diameter), counter electrode Pt wire, and reference is Ag/AgCl. Data referenced to Fc/Fc^+ couple. **b)** Current density vs varying concentration of TFA plot for 0.5 mM [(L^3)$BiCl_2$] **(3)**.

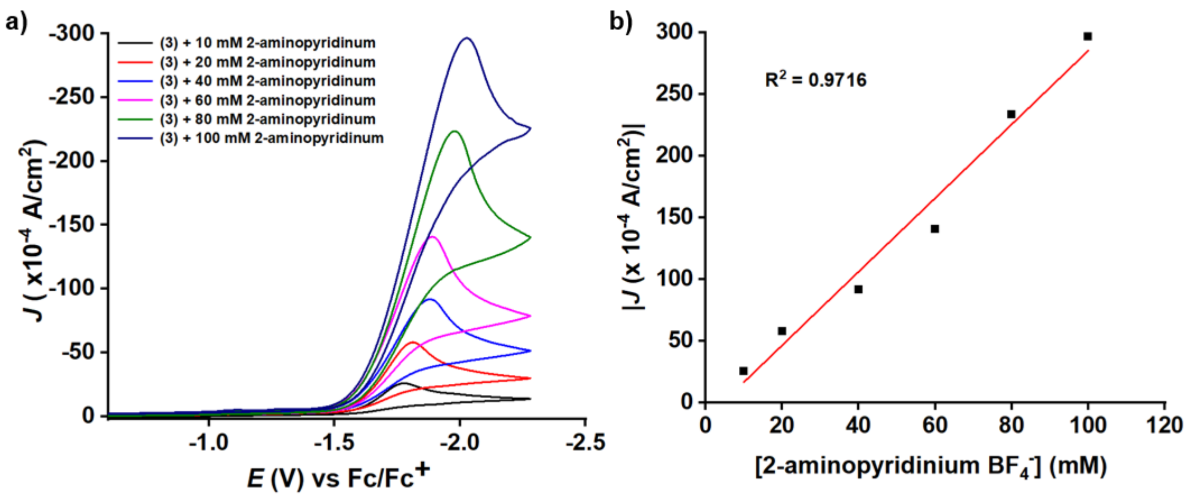

Figure S107. a) Cyclic Voltammogram of 0.5 mM [(L^3)$BiCl_2$] **(3)** with varying concentration of 2-aminopyridinium BF_4^- (pK_a 14.47 in acetonitrile) with 0.1 M $TBAPF_6$ as a supporting electrolyte in acetonitrile; Scan rate = 100 mV/s. Condition: Working electrode glassy carbon (3 mm diameter), counter electrode Pt wire, and reference is Ag/AgCl. Data referenced to Fc/Fc^+ couple. **b)** Current density vs varying concentration of 2-aminopyridinium BF_4^- plot for 0.5 mM [(L^3)$BiCl_2$] **(3)**.

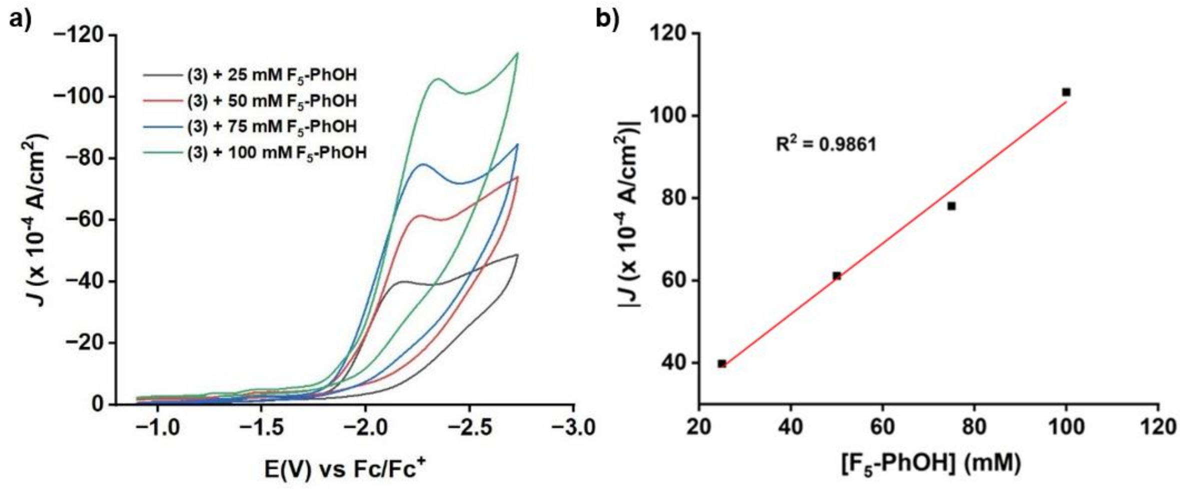

Figure S108. a) Cyclic Voltammogram of 0.5 mM [(L^3)$BiCl_2$] **(3)** with varying concentration of pentafluorophenol (pK_a 20.1 in acetonitrile) with 0.1 M $TBAPF_6$ as a supporting electrolyte in acetonitrile; Scan rate = 100 mV/s. Condition: Working electrode glassy carbon (3 mm diameter), counter electrode Pt wire, and reference is Ag/AgCl. Data referenced to Fc/Fc^+ couple. **b)** Current density vs varying concentration of pentafluorophenol plot for 0.5 mM [(L^3)$BiCl_2$] **(3)**.

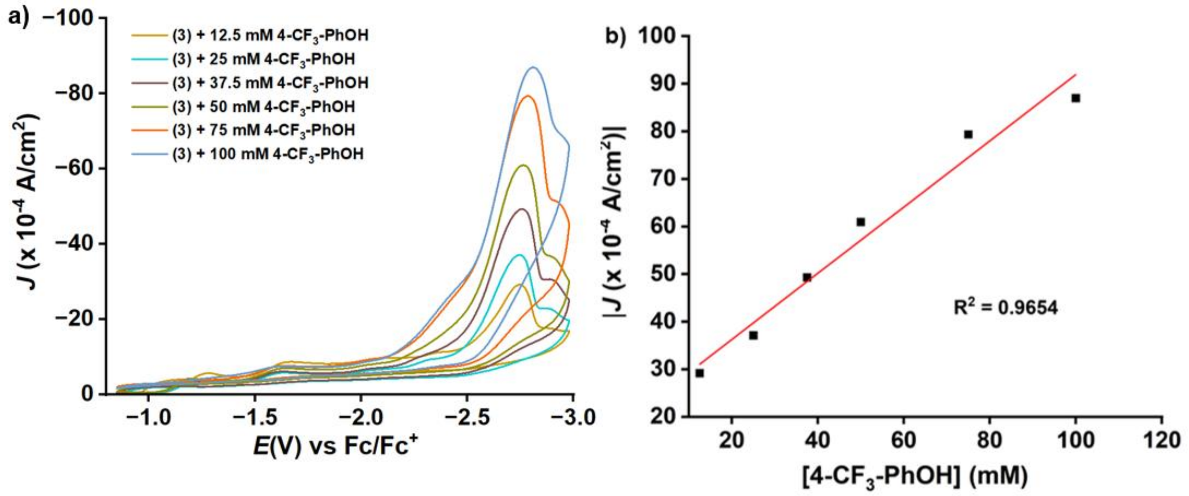

Figure S109. a) Cyclic Voltammogram of 0.5 mM [(L^3)BiCl_2] **(3)** with varying concentration of 4-trifluoromethylphenol (pK_a 25.54 in acetonitrile) with 0.1 M TBAPF_6 as a supporting electrolyte in acetonitrile; Scan rate = 100 mV/s. Condition: Working electrode glassy carbon (3 mm diameter), counter electrode Pt wire, and reference is Ag/AgCl. Data referenced to Fc/Fc^+ couple. **b)** Current density vs varying concentration of 4-trifluoromethylphenol plot for 0.5 mM [(L^3)BiCl_2] **(3)**.

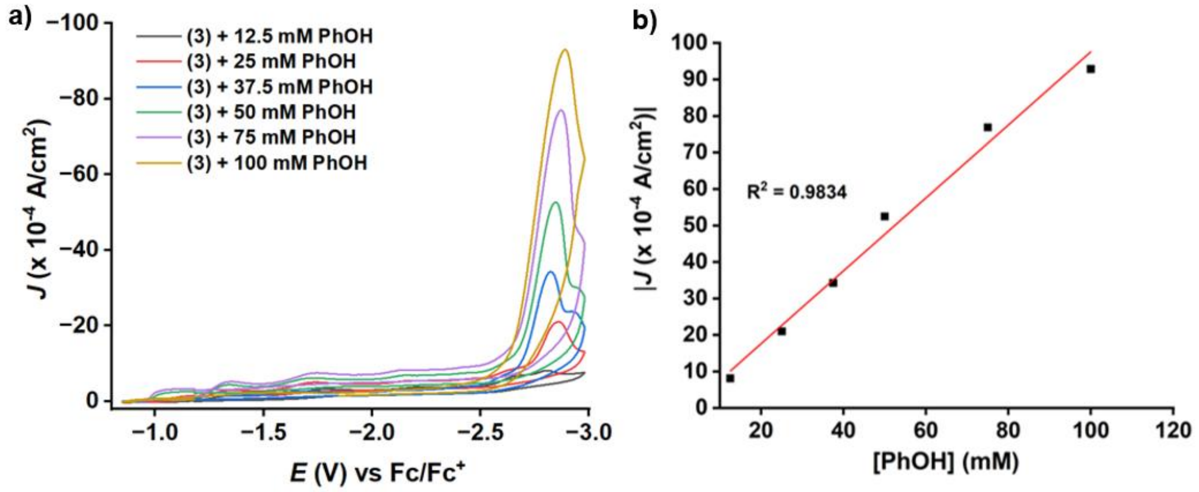

Figure S110. a) Cyclic Voltammogram of 0.5 mM [(L^3)BiCl_2] **(3)** with varying concentration of Phenol (pK_a 29.4 in acetonitrile) with 0.1 M TBAPF_6 as a supporting electrolyte in acetonitrile; Scan rate = 100 mV/s. Condition: Working electrode glassy carbon (3 mm diameter), counter electrode Pt wire, and reference is Ag/AgCl. Data referenced to Fc/Fc^+ couple. **b)** Current density vs varying concentration of Phenol plot for 0.5 mM [(L^3)BiCl_2] **(3)**.

The CVs for proton reduction studies of acids with catalyst **[(L⁴)BiCl₂] (4)** the corresponding current density (*J*) vs concentration plots are shown in Figure S111 to S120.

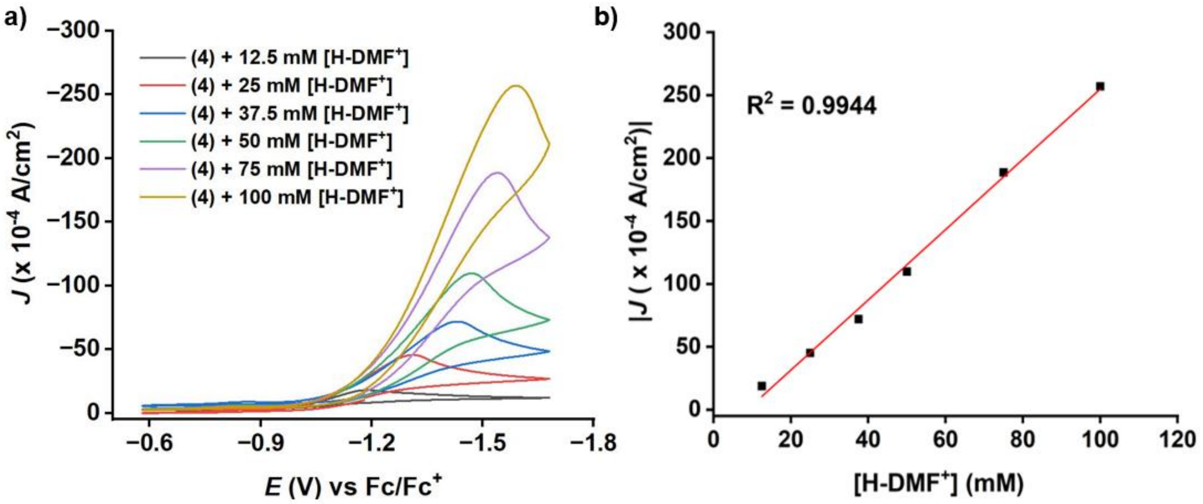

Figure S111. a) Cyclic Voltammogram of 0.5 mM [(L[4])BiCl$_2$] **(4)** with varying concentration of dimethylformamidium triflate (pK_a 6.1 in acetonitrile) with 0.1 M TBAPF$_6$ as a supporting electrolyte in acetonitrile; Scan rate = 100 mV/s. Condition: Working electrode glassy carbon (3 mm diameter), counter electrode Pt wire, and reference is Ag/AgCl. Data referenced to Fc/Fc$^+$ couple. **b)** Current density vs varying concentration of dimethylformamidium triflate plot for 0.5 mM [(L[4])BiCl$_2$] **(4)**.

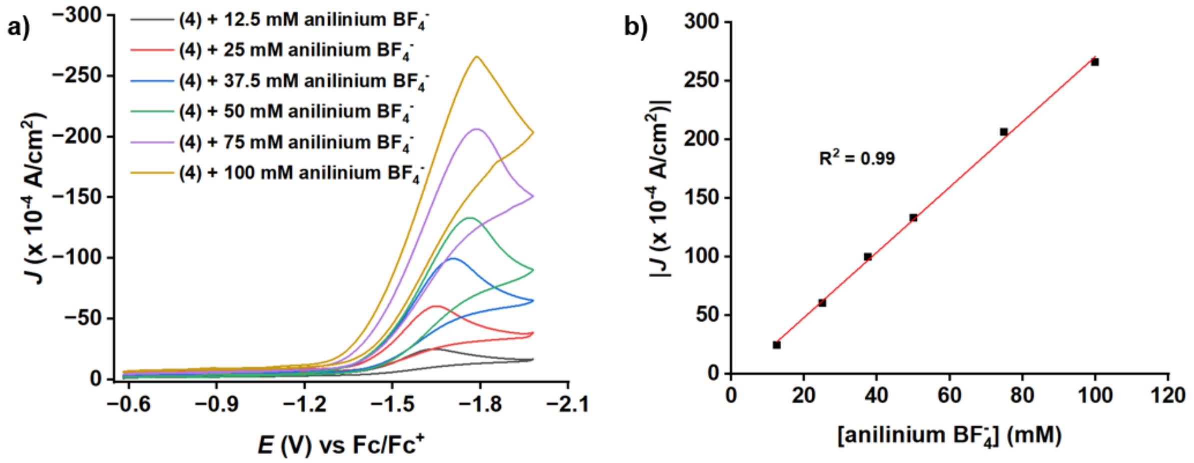

Figure S112. a) Cyclic Voltammogram of 0.5 mM [(L[4])BiCl$_2$] **(4)** with varying concentration of anilinium BF$_4^-$ (pK_a 10.62 in acetonitrile) with 0.1 M TBAPF$_6$ as a supporting electrolyte in acetonitrile; Scan rate = 100 mV/s. Condition: Working electrode glassy carbon (3 mm diameter), counter electrode Pt wire, and reference is Ag/AgCl. Data referenced to Fc/Fc$^+$ couple. **b)** Current density vs varying concentration of anilinium BF$_4^-$ plot for 0.5 mM [(L[4])BiCl$_2$] **(4)**.

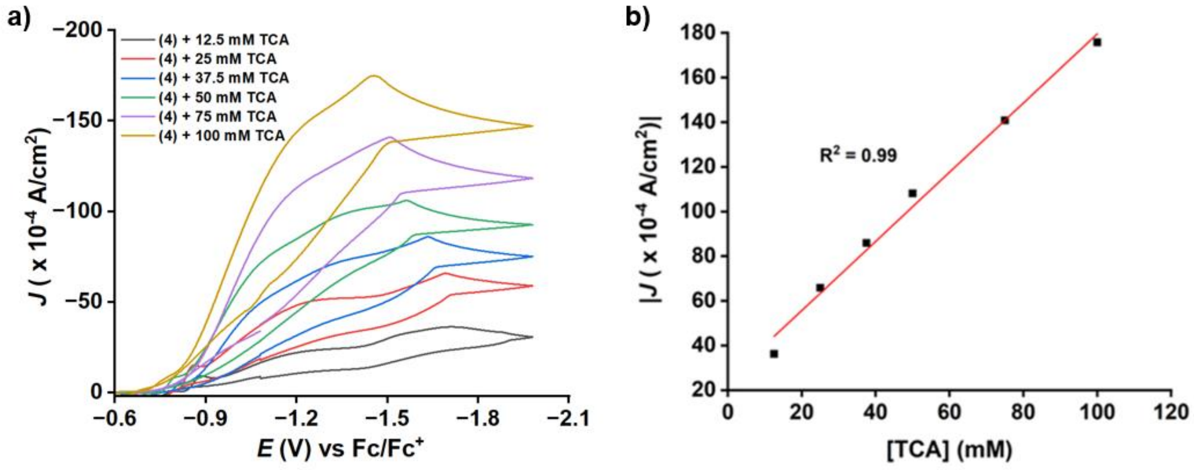

Figure S113. a) Cyclic Voltammogram of 0.5 mM [(L^4)$BiCl_2$] **(4)** with varying concentration of TCA (pK_a 10.75 in acetonitrile) with 0.1 M $TBAPF_6$ as a supporting electrolyte in acetonitrile; Scan rate = 100 mV/s. Condition: Working electrode glassy carbon (3 mm diameter), counter electrode Pt wire, and reference is Ag/AgCl. Data referenced to Fc/Fc^+ couple. **b)** Current density vs varying concentration of TCA plot for 0.5 mM [(L^4)$BiCl_2$] **(4)**.

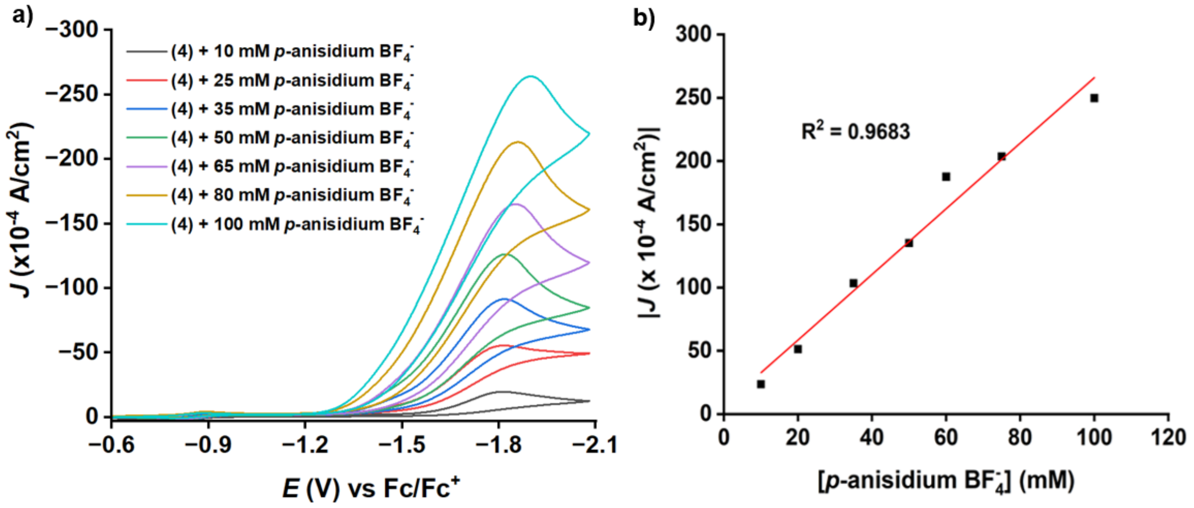

Figure S114. a) Cyclic Voltammogram of 0.5 mM [(L^4)$BiCl_2$] **(4)** with varying concentration of *p*-anisidium BF_4^- (pK_a 11.86 in acetonitrile) with 0.1 M $TBAPF_6$ as a supporting electrolyte in acetonitrile; Scan rate = 100 mV/s. Condition: Working electrode glassy carbon (3 mm diameter), counter electrode Pt wire, and reference is Ag/AgCl. Data referenced to Fc/Fc^+ couple. **b)** Current density vs varying concentration of *p*-anisidium BF_4^- plot for 0.5 mM [(L^4)$BiCl_2$] **(4)**.

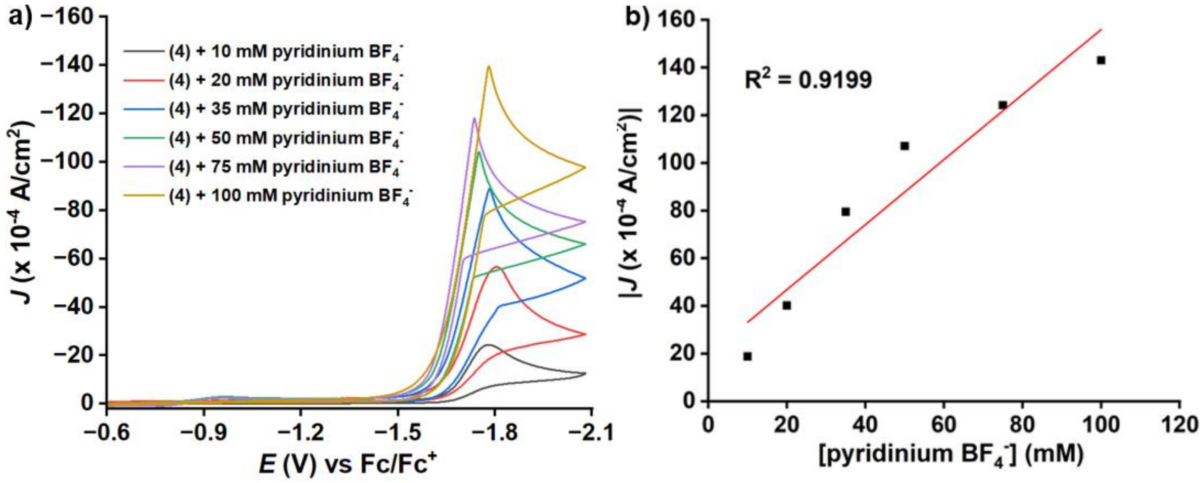

Figure S115. a) Cyclic Voltammogram of 0.5 mM [(L^4)$BiCl_2$] **(4)** with varying concentration of pyridinium BF_4^- (pK_a 12.53 in acetonitrile) with 0.1 M $TBAPF_6$ as a supporting electrolyte in acetonitrile; Scan rate = 100 mV/s. Condition: Working electrode glassy carbon (3 mm diameter), counter electrode Pt wire, and reference is Ag/AgCl. Data referenced to Fc/Fc^+ couple. **b)** Current density vs varying concentration of pyridinium BF_4^- plot for 0.5 mM [(L^4)$BiCl_2$] **(4)**.

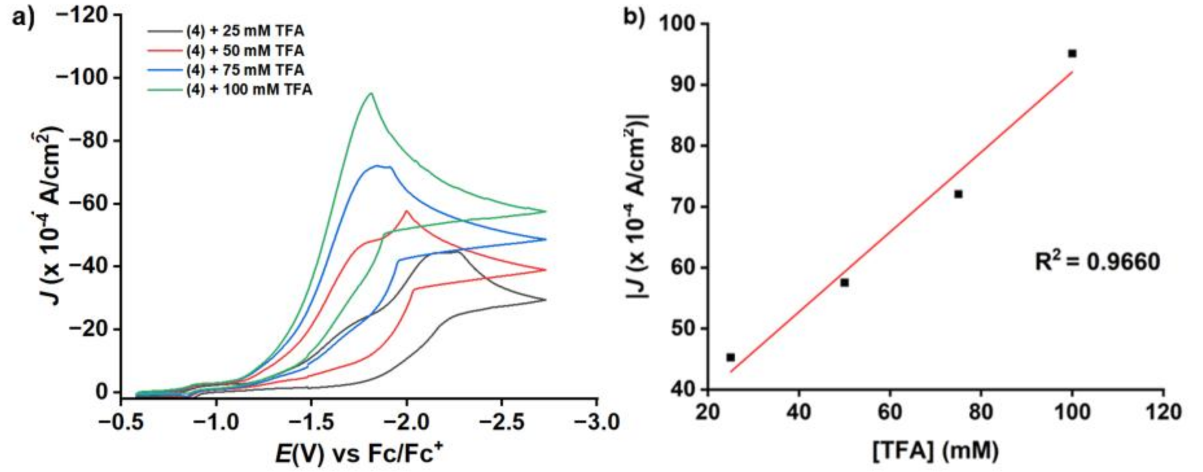

Figure S116. a) Cyclic Voltammogram of 0.5 mM [(L^4)$BiCl_2$] **(4)** with varying concentration of TFA (pK_a 12.65 in acetonitrile) with 0.1 M $TBAPF_6$ as a supporting electrolyte in acetonitrile; Scan rate = 100 mV/s. Condition: Working electrode glassy carbon (3 mm diameter), counter electrode Pt wire, and reference is Ag/AgCl. Data referenced to Fc/Fc^+ couple. **b)** Current density vs varying concentration of TFA plot for 0.5 mM [(L^4)$BiCl_2$] **(4)**.

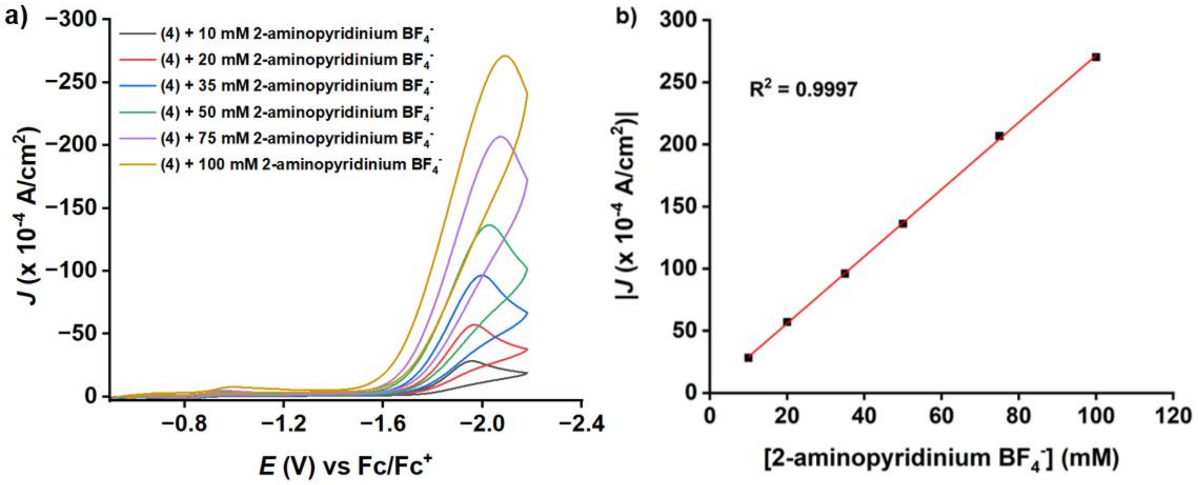

Figure S117. a) Cyclic Voltammogram of 0.5 mM [(L^4)$BiCl_2$] **(4)** with varying concentration of 2-aminopyridinium BF_4^- (pK_a 14.47 in acetonitrile) with 0.1 M $TBAPF_6$ as a supporting electrolyte in acetonitrile; Scan rate = 100 mV/s. Condition: Working electrode glassy carbon (3 mm diameter), counter electrode Pt wire, and reference is Ag/AgCl. Data referenced to Fc/Fc^+ couple. **b)** Current density vs varying concentration of 2-aminopyridinium BF_4^- plot for 0.5 mM [(L^4)$BiCl_2$] **(4)**.

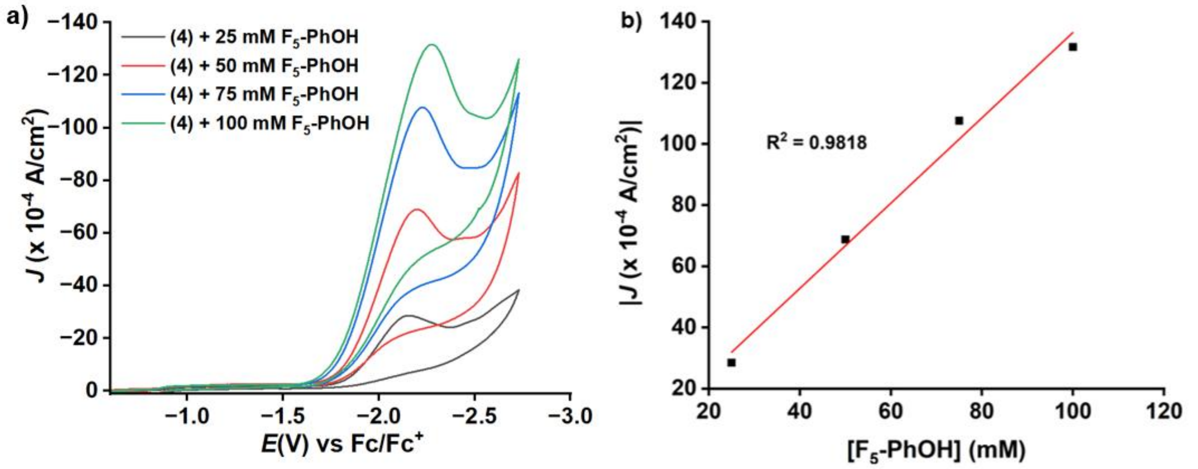

Figure S118. a) Cyclic Voltammogram of 0.5 mM [(L^4)$BiCl_2$] **(4)** with varying concentration of pentafluorophenol (pK_a 20.1 in acetonitrile) with 0.1 M $TBAPF_6$ as a supporting electrolyte in acetonitrile; Scan rate = 100 mV/s. Condition: Working electrode glassy carbon (3 mm diameter), counter electrode Pt wire, and reference is Ag/AgCl. Data referenced to Fc/Fc^+ couple. **b)** Current density vs varying concentration of pentafluorophenol plot for 0.5 mM [(L^4)$BiCl_2$] **(4)**.

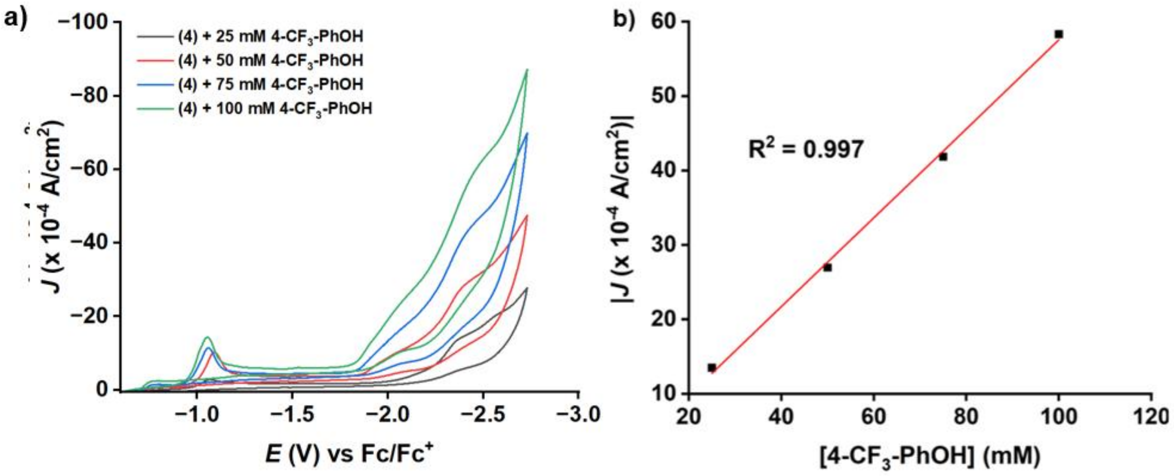

Figure S119. a) Cyclic Voltammogram of 0.5 mM [(L^4)$BiCl_2$] **(4)** with varying concentration of 4-trifluoromethylphenol (pK_a 25.54 in acetonitrile) with 0.1 M $TBAPF_6$ as a supporting electrolyte in acetonitrile; Scan rate = 100 mV/s. Condition: Working electrode glassy carbon (3 mm diameter), counter electrode Pt wire, and reference is Ag/AgCl. Data referenced to Fc/Fc^+ couple. **b)** Current density vs varying concentration of 4-trifluoromethylphenol plot for 0.5 mM [(L^4)$BiCl_2$] **(4)**.

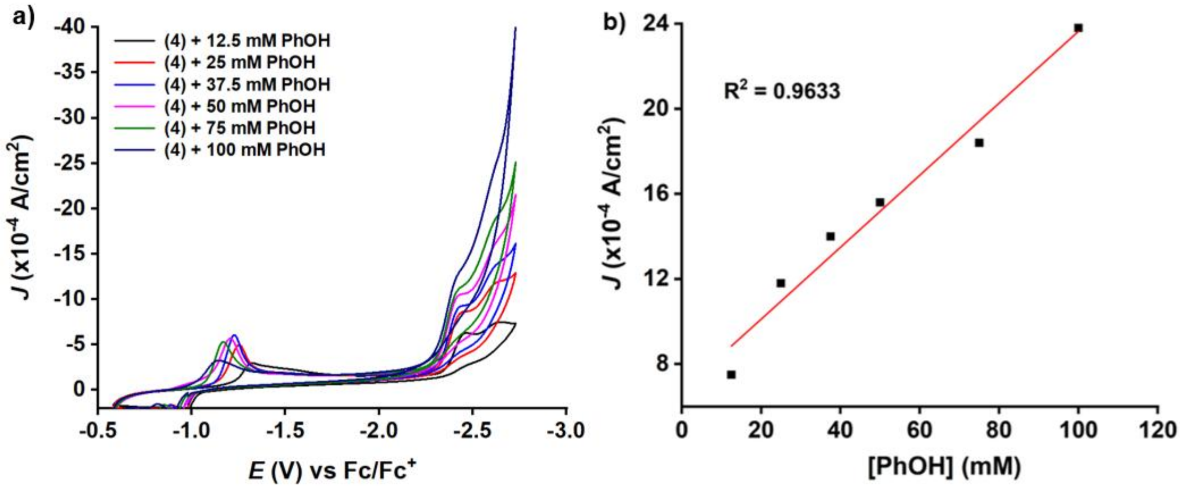

Figure S120. a) Cyclic Voltammogram of 0.5 mM [(L^4)$BiCl_2$] **(4)** with varying concentration of Phenol (pK_a 29.4 in acetonitrile) with 0.1 M $TBAPF_6$ as a supporting electrolyte in acetonitrile; Scan rate = 100 mV/s. Condition: Working electrode glassy carbon (3 mm diameter), counter electrode Pt wire, and reference is Ag/AgCl. Data referenced to Fc/Fc^+ couple. **b)** Current density vs varying concentration of Phenol plot for 0.5 mM [(L^4)$BiCl_2$] **(4)**.

The CVs for proton reduction studies of acids with catalyst **[(L^5)BiCl$_2$] (5)** the corresponding current density (J) vs concentration plots are shown in Figure S121 to S126.

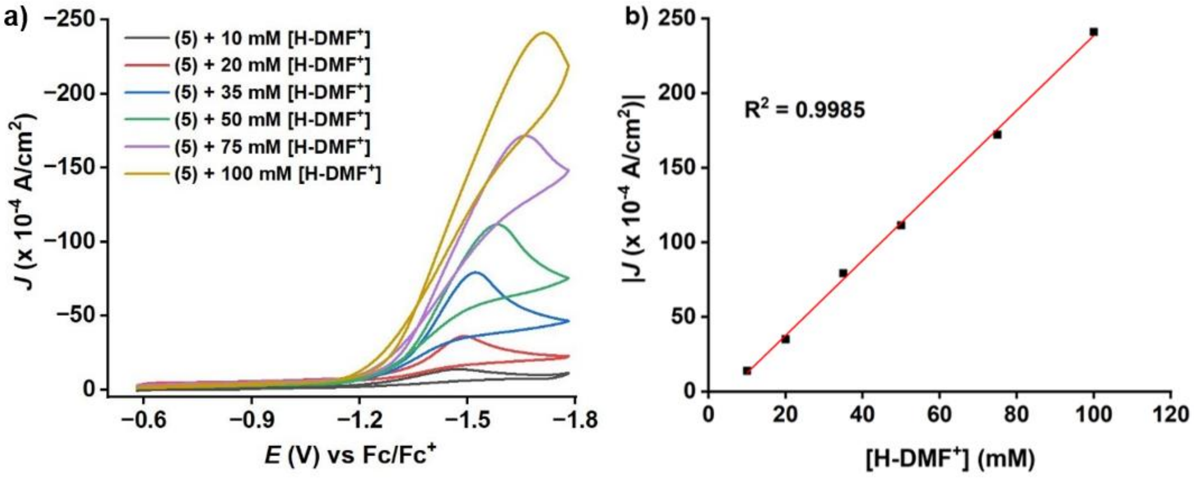

Figure S121. a) Cyclic Voltammogram of 0.5 mM [(L^5)BiCl$_2$] **(5)** with varying concentration of dimethylformamidium triflate (pK_a 6.1 in acetonitrile) with 0.1 M TBAPF$_6$ as a supporting electrolyte in acetonitrile; Scan rate = 100 mV/s. Condition: Working electrode glassy carbon (3 mm diameter), counter electrode Pt wire, and reference is Ag/AgCl. Data referenced to Fc/Fc$^+$ couple. **b)** Current density vs varying concentration of dimethylformamidium triflate plot for 0.5 mM [(L^5)BiCl$_2$] **(5)**.

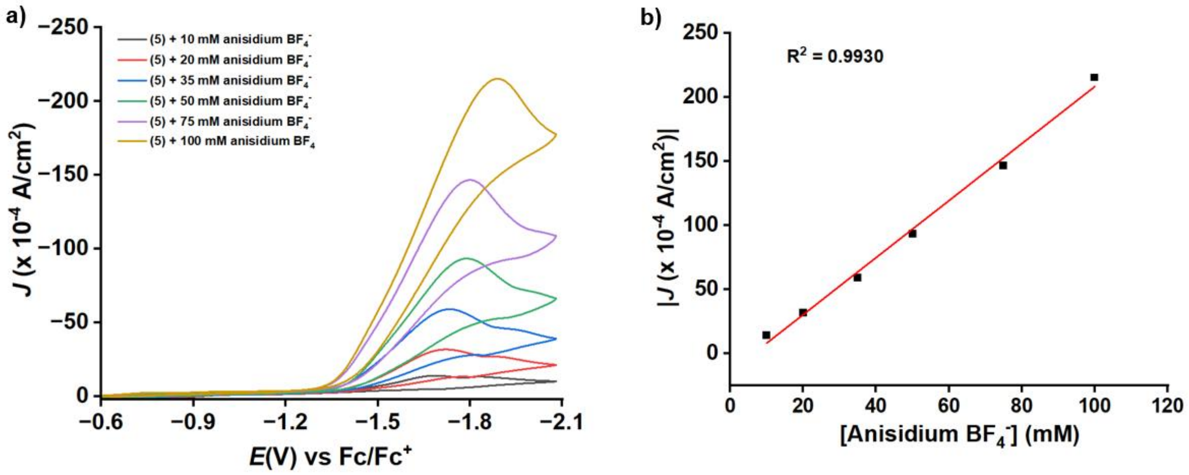

Figure S122. a) Cyclic Voltammogram of 0.5 mM [(L^5)BiCl$_2$] **(5)** with varying concentration of *p*-anisidium BF_4^- (pK_a 11.86 in acetonitrile) with 0.1 M TBAPF$_6$ as a supporting electrolyte in acetonitrile; Scan rate = 100 mV/s. Condition: Working electrode glassy carbon (3 mm diameter), counter electrode Pt wire, and reference is Ag/AgCl. Data referenced to Fc/Fc$^+$ couple. **b)** Current density vs varying concentration of *p*-anisidium BF_4^- plot for 0.5 mM [(L^5)BiCl$_2$] **(5)**.

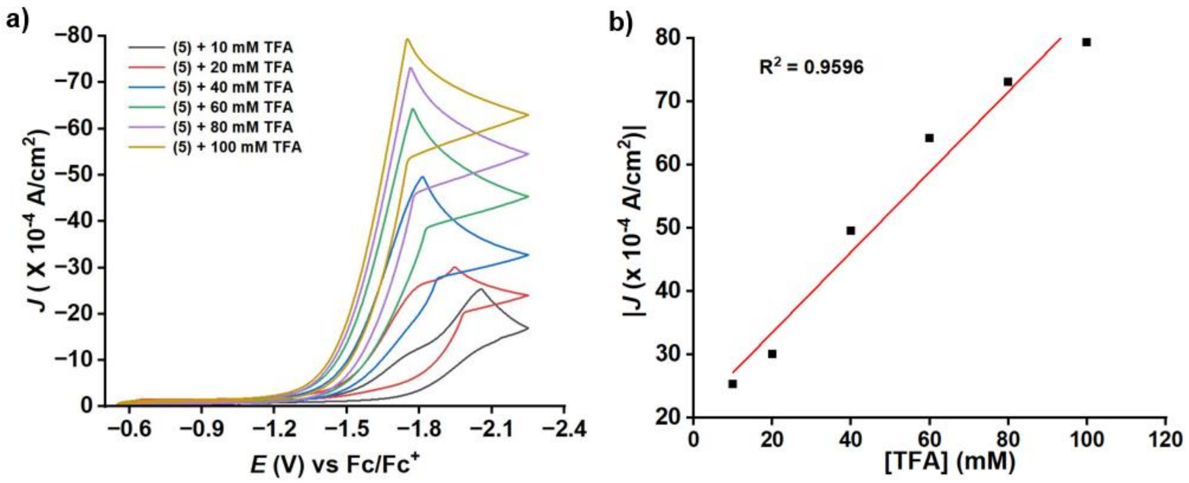

Figure S123. a) Cyclic Voltammogram of 0.5 mM [(L^5)BiCl$_2$] **(5)** with varying concentration of TFA (pK_a 12.65 in acetonitrile) with 0.1 M TBAPF$_6$ as a supporting electrolyte in acetonitrile; Scan rate = 100 mV/s. Condition: Working electrode glassy carbon (3 mm diameter), counter electrode Pt wire, and reference is Ag/AgCl. Data referenced to Fc/Fc$^+$ couple. **b)** Current density vs varying concentration of TFA plot for 0.5 mM [(L^5)BiCl$_2$] **(5)**.

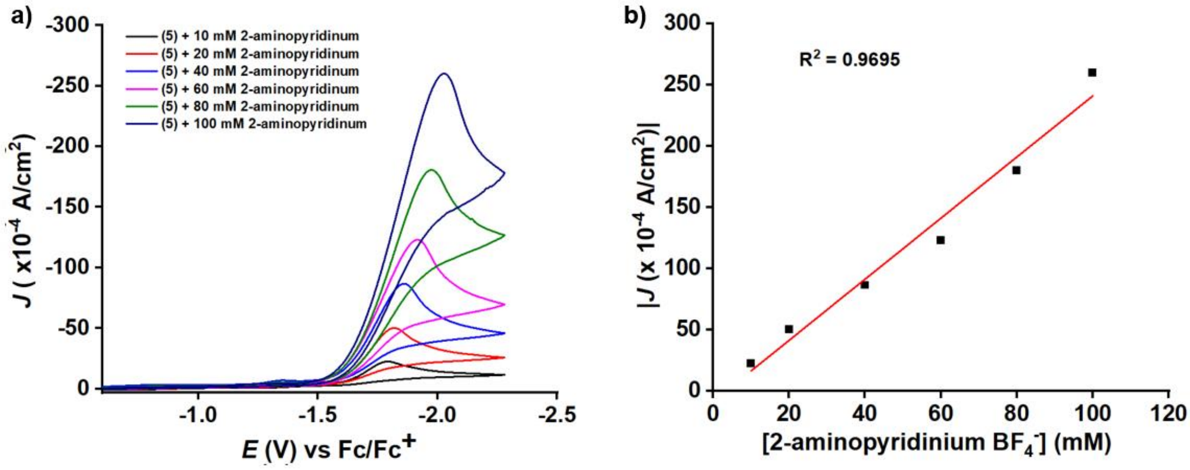

Figure S124. a) Cyclic Voltammogram of 0.5 mM [(L^5)BiCl$_2$] **(5)** with varying concentration of 2-aminopyridinium BF$_4^-$ (pK_a 14.47 in acetonitrile) with 0.1 M TBAPF$_6$ as a supporting electrolyte in acetonitrile; Scan rate = 100 mV/s. Condition: Working electrode glassy carbon (3 mm diameter), counter electrode Pt wire, and reference is Ag/AgCl. Data referenced to Fc/Fc$^+$ couple. **b)** Current density vs varying concentration of 2-aminopyridinium BF$_4^-$ plot for 0.5 mM [(L^5)BiCl$_2$] **(5)**

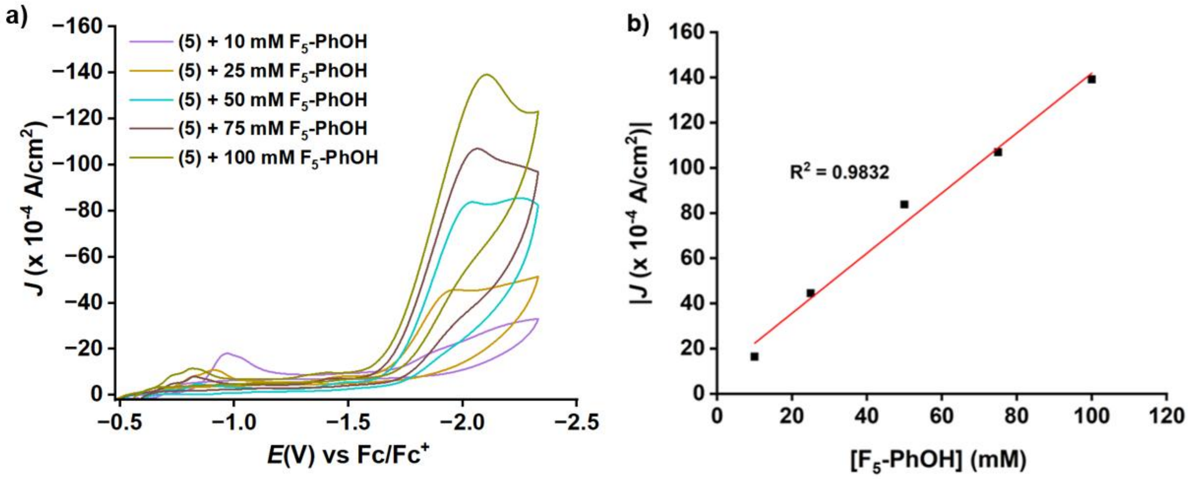

Figure S125. a) Cyclic Voltammogram of 0.5 mM [(L^5)$BiCl_2$] **(5)** with varying concentration of pentafluorophenol (pK_a 20.1 in acetonitrile) with 0.1 M $TBAPF_6$ as a supporting electrolyte in acetonitrile; Scan rate = 100 mV/s. Condition: Working electrode glassy carbon (3 mm diameter), counter electrode Pt wire, and reference is Ag/AgCl. Data referenced to Fc/Fc^+ couple. **b)** Current density vs varying concentration of pentafluorophenol plot for 0.5 mM [(L^5)$BiCl_2$] **(5)**.

The CVs for proton reduction study of catalyst [(L^6)$BiCl_2$] (6)

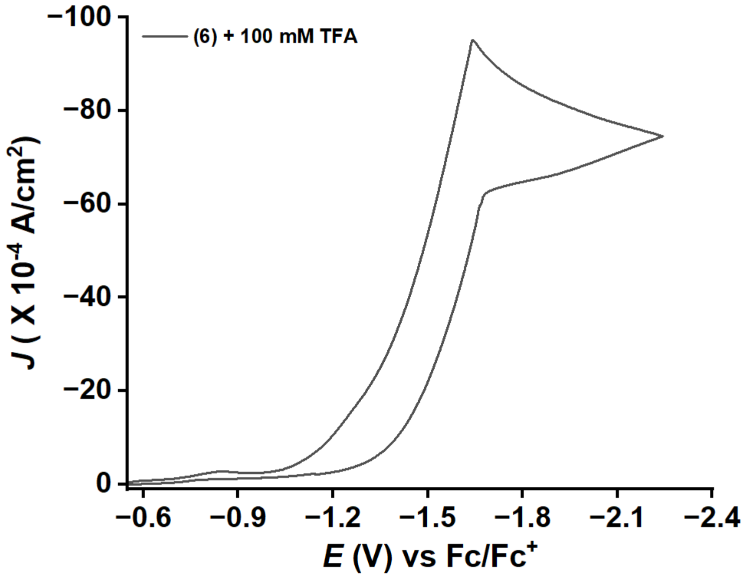

Figure S126. a) Cyclic Voltammogram of 0.5 mM [(L^6)$BiCl_2$] **(6)** at 0.1 M of TFA (pK_a 12.65 in acetonitrile) with 0.1 M $TBAPF_6$ as a supporting electrolyte in acetonitrile; Scan rate = 100 mV/s. Condition: Working electrode glassy carbon (3 mm diameter), counter electrode Pt wire, and reference is Ag/AgCl. Data referenced to Fc/Fc^+ couple.

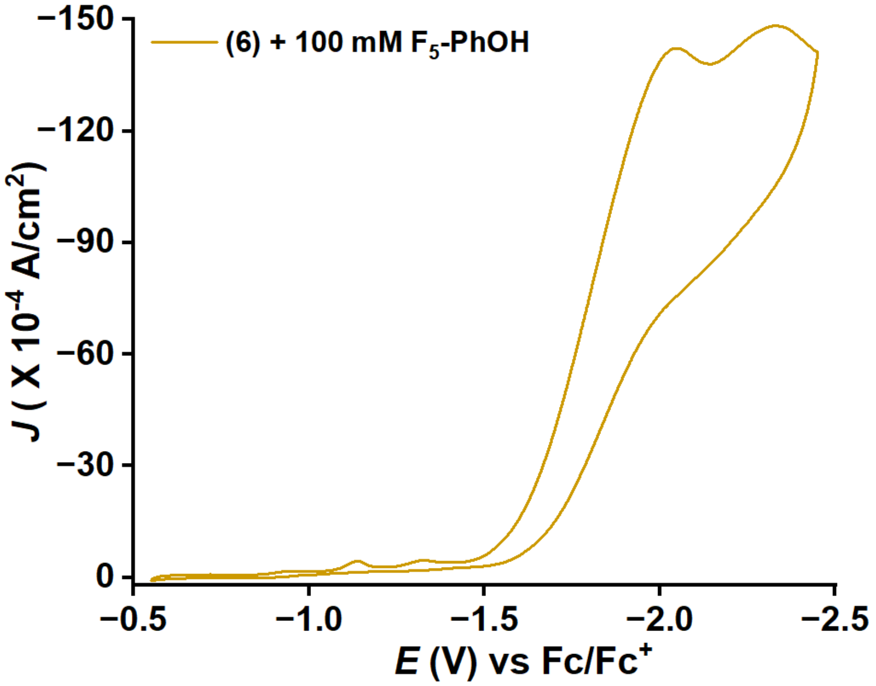

Figure S127. Cyclic Voltammogram of 0.5 mM [(L^6)BiCl$_2$] **(6)** at 0.1 M of F_5-PhOH (pK_a 20.1 in acetonitrile) with 0.1 M TBAPF$_6$ as a supporting electrolyte in acetonitrile; Scan rate = 100 mV/s. Condition: Working electrode glassy carbon (3 mm diameter), counter electrode Pt wire, and reference is Ag/AgCl. Data referenced to Fc/Fc$^+$ couple.

The CVs for proton reduction studies of acids with catalyst **[(L^7)$BiCl_2$] (7)** the corresponding current density (*J*) vs concentration plots are shown in Figure S128 and S129.

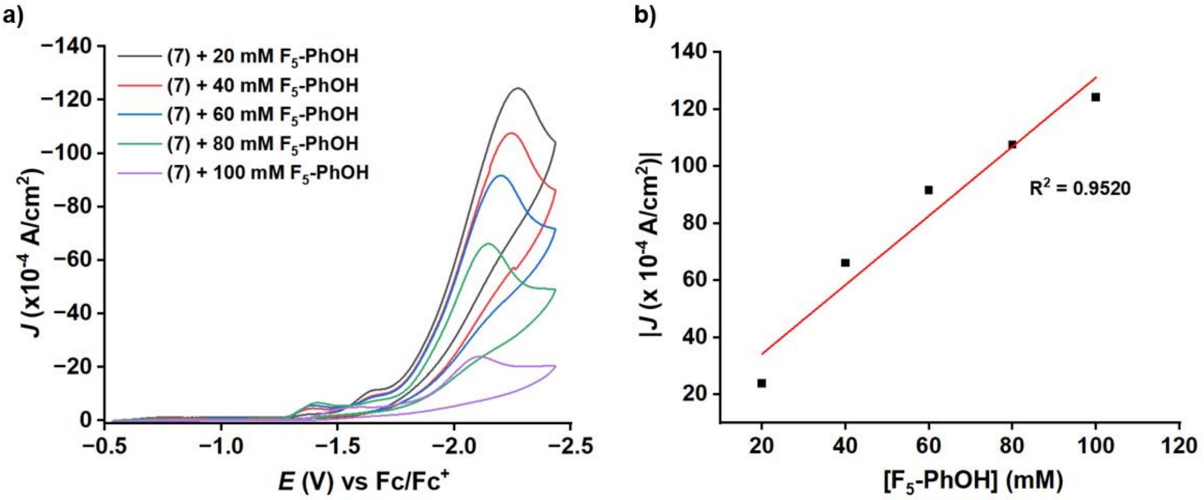

Figure S128 a) Cyclic Voltammogram of 0.5 mM [(L^7)$BiCl_2$] **(7)** with varying concentration of pentafluorophenol (pK_a 20.1 in acetonitrile) with 0.1 M $TBAPF_6$ as a supporting electrolyte in acetonitrile; Scan rate = 100 mV/s. Condition: Working electrode glassy carbon (3 mm diameter), counter electrode Pt wire, and reference is Ag/AgCl. Data referenced to Fc/Fc^+ couple. **b)** Current density vs varying concentration of pentafluorophenol plot for 0.5 mM [(L^7)$BiCl_2$] **(7)**.

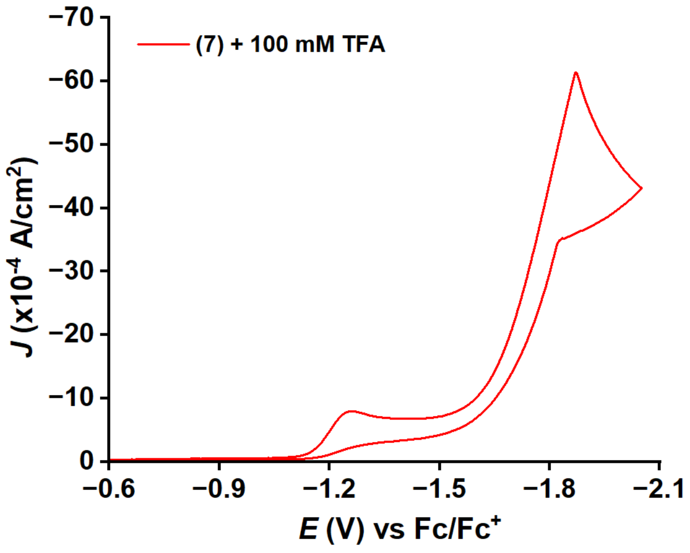

Figure S129. a) Cyclic Voltammogram of 0.5 mM [(L^7)$BiCl_2$] **(7)** at 0.1 M of TFA (pK_a 12.65 in acetonitrile) with 0.1 M $TBAPF_6$ as a supporting electrolyte in acetonitrile; Scan rate = 100 mV/s. Condition: Working electrode glassy carbon (3 mm diameter), counter electrode Pt wire, and reference is Ag/AgCl. Data referenced to Fc/Fc^+ couple.

Order of catalyst (VIII)

A study for dependence on the catalyst concentration against 0.1 M *p*-anisidium tetrafluoroborate with **[(L[1])BiCl$_2$] (1)** and [**(L[2])BiCl$_2$] (2)** (Figure S130, S131) portrays a first order dependence on the catalyst concentration as i_{cat} is directly proportional to catalyst concentration from eq. below

$$i_{cat} = nFA\,[cat]\,(Dk[Q]^y)^{1/2}$$

where [Q] is the concentration of the acid, A is the cross-sectional area of the electrode, k is the rate constant of outer-sphere electron transfer from the reduced catalyst to the substrate (here acid), n is the number of electrons transferred, D is the diffusion coefficient for the catalyst, [cat] is the bulk concentration of the catalyst.

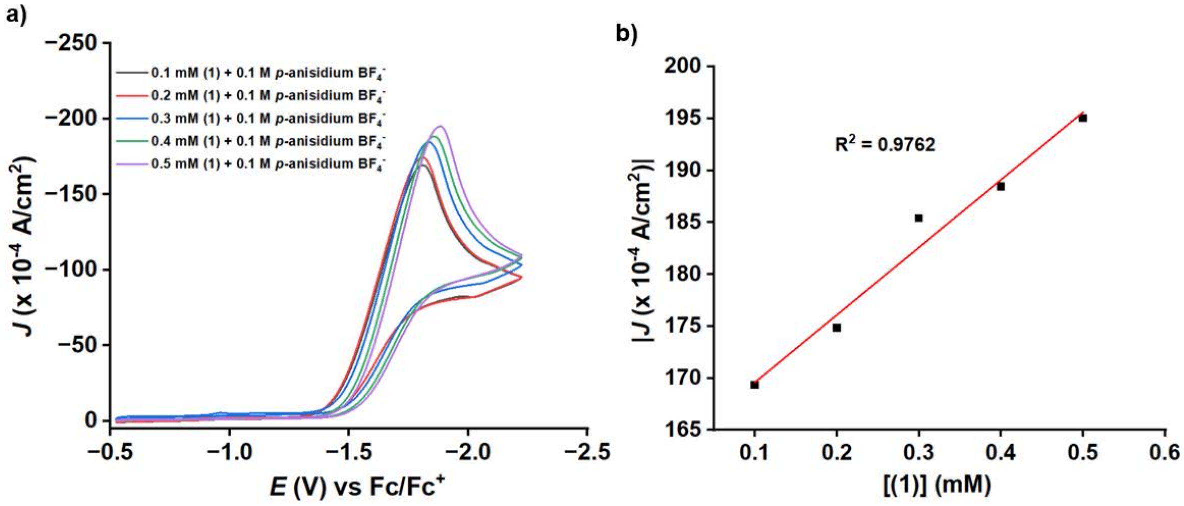

Figure S130 a) Cyclic Voltammogram of 0.1 M *p*-anisidium BF_4^- (pK_a 11.86 in acetonitrile) with varying concentration of [(L[1])BiCl$_2$] **(1)** with 0.1 M TBAPF$_6$ as a supporting electrolyte in acetonitrile; Scan rate = 100 mV/s. Condition: Working electrode glassy carbon (3 mm diameter), counter electrode Pt wire, and reference is Ag/AgCl. Data referenced to Fc/Fc$^+$ couple. **b)** Current density vs varying concentration of **[(L[1])BiCl$_2$] (1)** plot for 0.1 M *p*-anisidium BF_4^- (pK_a 11.86 in acetonitrile).

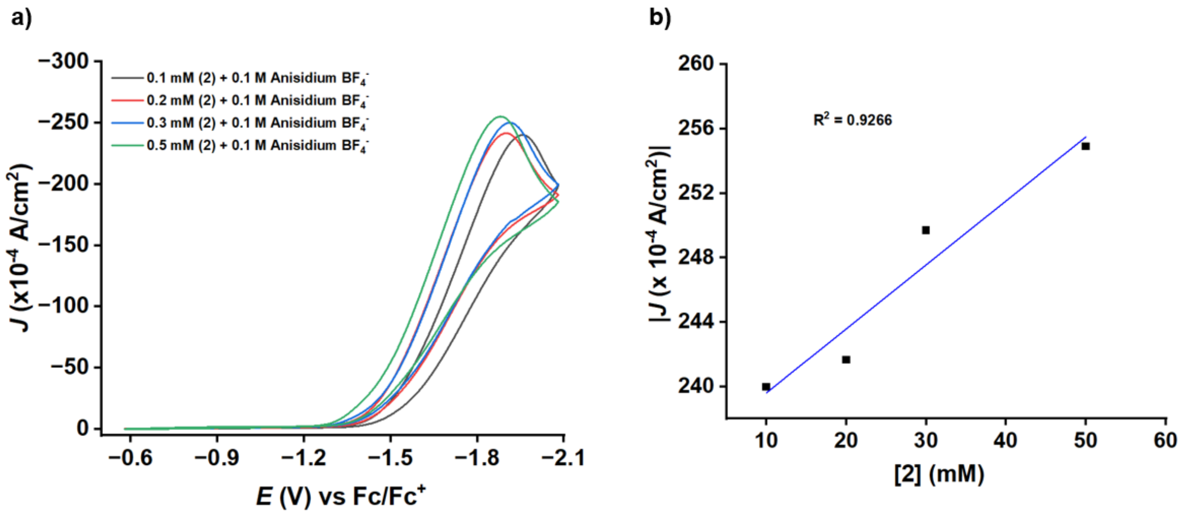

Figure S131. a) Cyclic Voltammogram of 0.1 M *p*-anisidium BF_4^- (pK_a 11.86 in acetonitrile) with varying concentration of $[(L^2)BiCl_2]$ **(2)** with 0.1 M $TBAPF_6$ as a supporting electrolyte in acetonitrile; Scan rate = 100 mV/s. Condition: Working electrode glassy carbon (3 mm diameter), counter electrode Pt wire, and reference is Ag/AgCl. Data referenced to Fc/Fc^+ couple. **b)** Current density vs varying concentration of **$[(L^2)BiCl_2]$ (2)** plot for 0.1 M *p*-anisidium BF_4^- (pK_a 11.86 in acetonitrile).

Electrochemical measurement using glassy carbon as an inert counter electrode (IX)

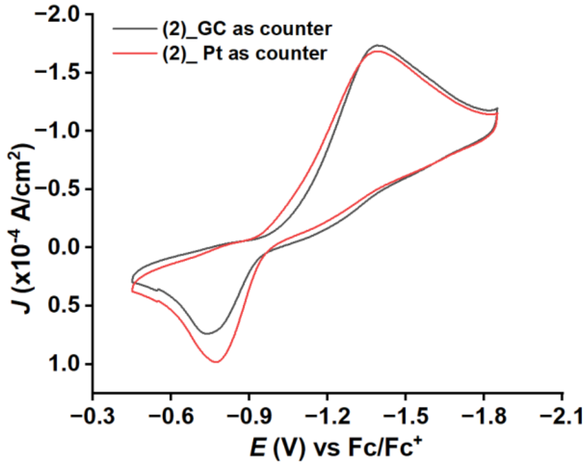

Figure S132. a) Cyclic Voltammogram of 0.5 mM [(L^2)$BiCl_2$] **(2)** and 0.1 M $TBAPF_6$ as a supporting electrolyte in acetonitrile; Scan rate = 100 mV/s using glassy carbon as counter (black) and using platinum as counter (red) (Working electrode glassy carbon (3 mm diameter), and reference is Ag/AgCl). Data referenced to Fc/Fc^+ couple.

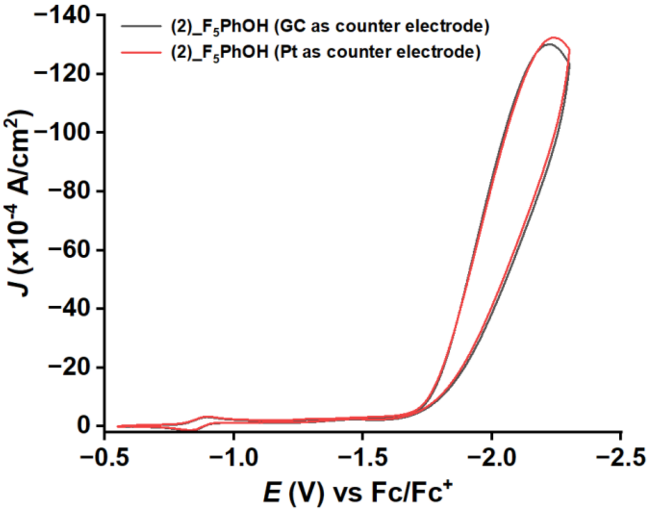

Figure S133. Cyclic Voltammogram of 0.5 mM [(L^2)$BiCl_2$] **(2)** with 0.1M of pentafluorophenol (pK_a 20.1 in acetonitrile) and 0.1 M $TBAPF_6$ as a supporting electrolyte in acetonitrile; Scan rate = 100 mV/s using glassy carbon as counter (black) and using platinum as counter(red) (Working electrode glassy carbon (3 mm diameter), and reference is Ag/AgCl). Data referenced to Fc/Fc^+ couple.

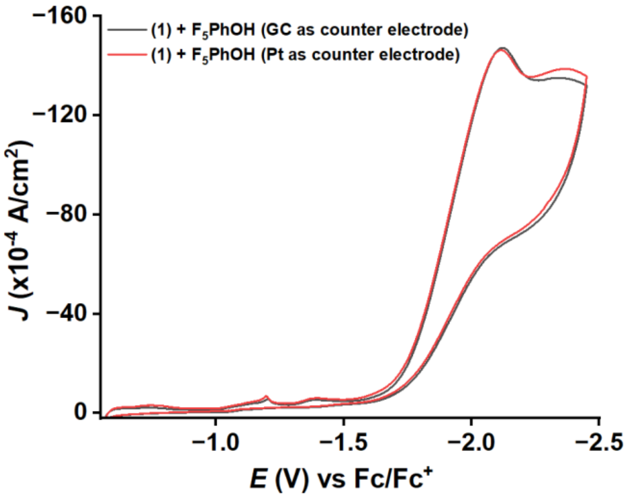

Figure S134. Cyclic Voltammogram of 0.5 mM [(L[1])$BiCl_2$] **(1)** with 0.1 M of pentafluorophenol (pK_a 20.1 in acetonitrile) and 0.1 M $TBAPF_6$ as a supporting electrolyte in acetonitrile; Scan rate = 100 mV/s using glassy carbon as counter (black) and using platinum as counter(red) (Working electrode glassy carbon (3 mm diameter), and reference is Ag/AgCl). Data referenced to Fc/Fc^+ couple.

Rinse tests (Homogeneity test) and control studies (X)

Rinse test was done to ascertain whether an analyte has been adsorbed to the electrode surface. Absence of any electrochemical characteristics in the rinse test rules out any significant adsorption and conveys that the catalysis is purely homogenous. Rinse test CVs of [(L^1)$BiCl_2$] **(1),** [(L^2)$BiCl_2$] **(2),** [(L^3)$BiCl_2$] **(3)**, [(L^4)$BiCl_2$] **(4)** are shown in Figure S135-S138.[6]

Also control studies were done for all the proton sources in the absence of any catalyst. A lower current density in the absence of catalyst emphasises the role of catalyst in this study for electrochemical proton reduction. Figure S139-S148 show performed with all eleven acid as proton sources with catalyst [(L^1)$BiCl_2$] **(1).**

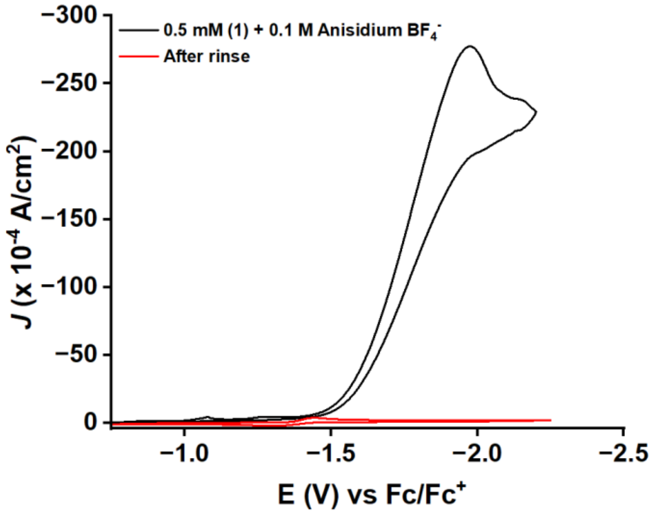

Figure S135. Cyclic Voltammogram of 0.5 mM [(L^1)$BiCl_2$] **(1)** at 0.1 M of (Anisidium)BF_4^- (pK_a 11.86 in acetonitrile) (black) and Cyclic Voltammogram after rinsing (red) with 0.1 M $TBAPF_6$ as a supporting electrolyte in acetonitrile; Scan rate = 100 mV/s. Condition: Working electrode glassy carbon (3 mm diameter), counter electrode Pt wire, and reference is Ag/AgCl. Data referenced to Fc/Fc^+ couple.

.

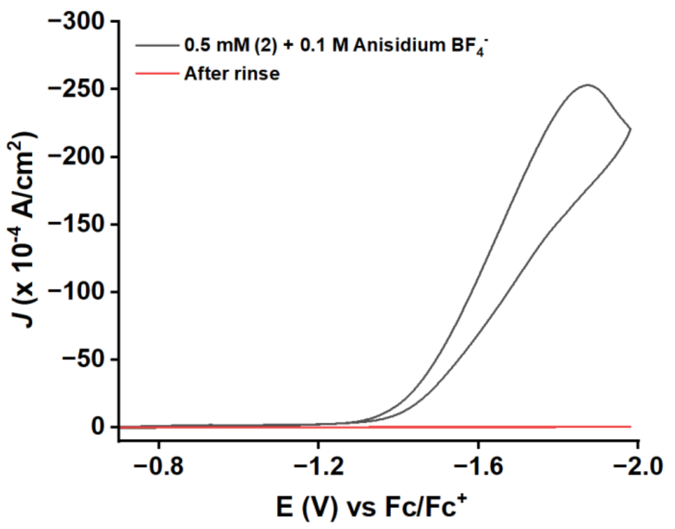

Figure S136. Cyclic Voltammogram of 0.5 mM [(L^2)$BiCl_2$] **(2)** at 0.1 M of (Anisidium)BF_4^- (pK_a 11.86 in acetonitrile) (black) and Cyclic Voltammogram after rinsing (red) with 0.1 M $TBAPF_6$ as a supporting electrolyte in acetonitrile; Scan rate = 100 mV/s. Condition: Working electrode glassy carbon (3 mm diameter), counter electrode Pt wire, and reference is Ag/AgCl. Data referenced to Fc/Fc^+ couple.

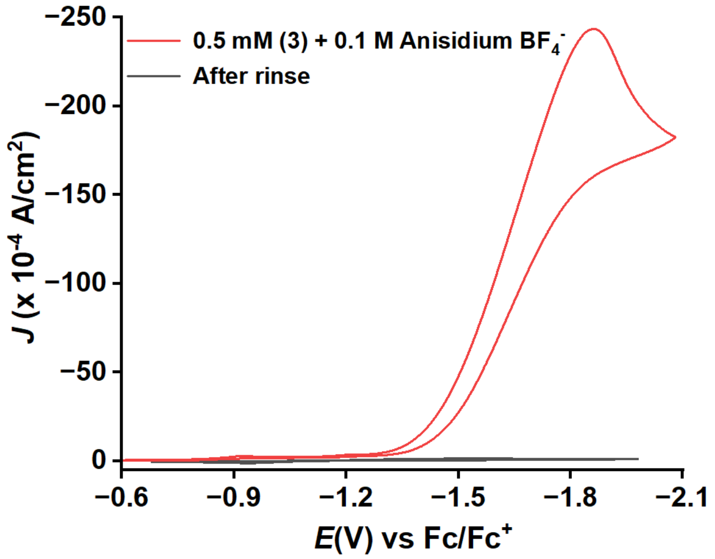

Figure S137. Cyclic Voltammogram of 0.5 mM [(L^3)$BiCl_2$] **(3)** at 0.1 M of (Anisidium)BF_4^- (pK_a 11.86 in acetonitrile) (black) and Cyclic Voltammogram after rinsing (red) with 0.1 M $TBAPF_6$ as a supporting electrolyte in acetonitrile; Scan rate = 100 mV/s. Condition: Working electrode glassy carbon (3 mm diameter), counter electrode Pt wire, and reference is Ag/AgCl. Data referenced to Fc/Fc^+ couple.

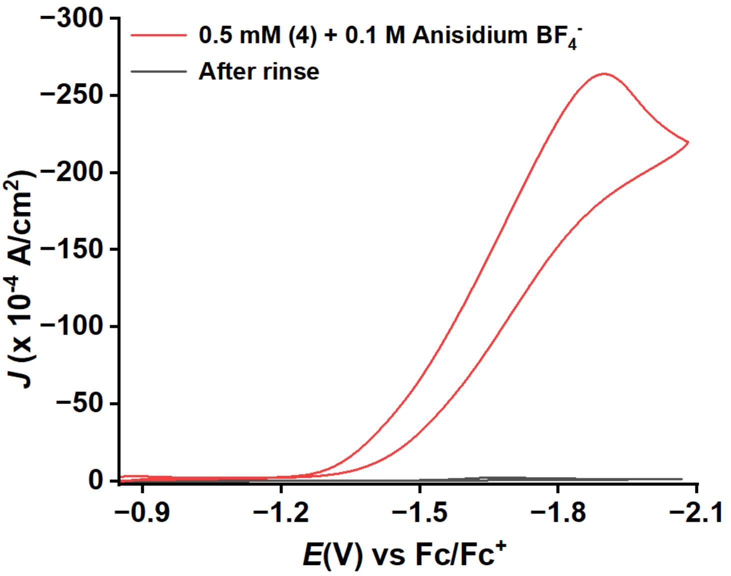

Figure S138. Cyclic Voltammogram of 0.5 mM [(L[4])$BiCl_2$] **(4)** at 0.1 M of (Anisidium)BF_4^- (pK_a 11.86 in acetonitrile) (black) and Cyclic Voltammogram after rinsing (red) with 0.1 M $TBAPF_6$ as a supporting electrolyte in acetonitrile; Scan rate = 100 mV/s. Condition: Working electrode glassy carbon (3 mm diameter), counter electrode Pt wire, and reference is Ag/AgCl. Data referenced to Fc/Fc^+ couple.

Control study of acids with glassy carbon electrode vs catalyst [(L[1])$BiCl_2$] (1) toward HER

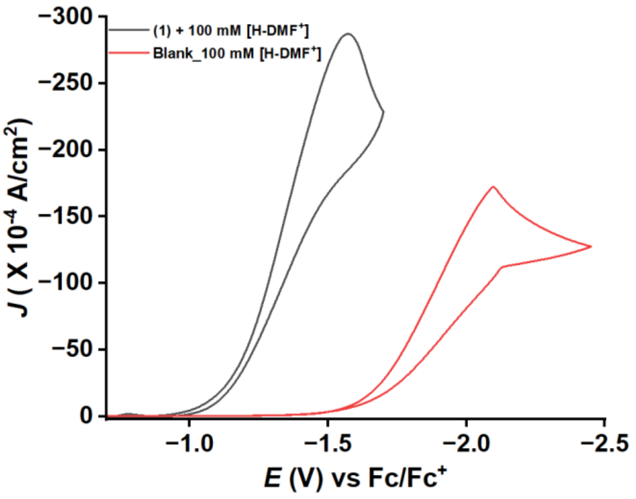

Figure S139. Cyclic Voltammogram of 0.5 mM [(L[1])$BiCl_2$] **(1)** at 0.1 M of dimethylformamidium triflate (pK_a 6.1 in acetonitrile) (black) and blank 0.1 M of dimethylformamidium triflate (pK_a 6.1 in acetonitrile) (red) with 0.1 M $TBAPF_6$ as a supporting electrolyte in acetonitrile; Scan rate =

100 mV/s. Condition: Working electrode glassy carbon (3 mm diameter), counter electrode Pt wire, and reference is Ag/AgCl. Data referenced to Fc/Fc^+ couple.

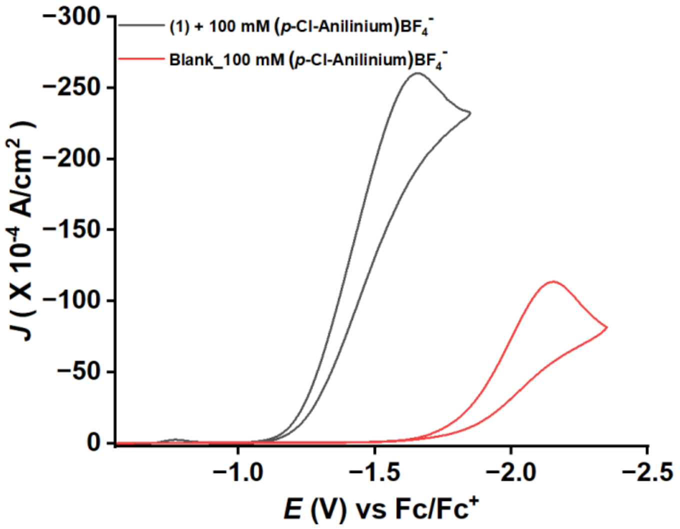

Figure S140. Cyclic Voltammogram of 0.5 mM [(L[1])$BiCl_2$] **(1)** at 0.1 M of *p*-Cl-Anilinium BF_4^- (pK_a 9.7 in acetonitrile) (black) and blank 0.1 M of *p*-Cl-Anilinium BF_4^- (pK_a 9.7 in acetonitrile) (red) with 0.1 M $TBAPF_6$ as a supporting electrolyte in acetonitrile; Scan rate = 100 mV/s. Condition: Working electrode glassy carbon (3 mm diameter), counter electrode Pt wire, and reference is Ag/AgCl. Data referenced to Fc/Fc^+ couple.

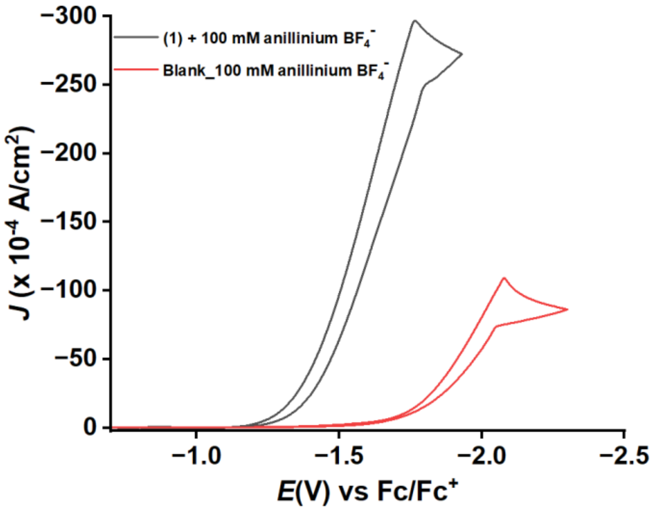

Figure S141. Cyclic Voltammogram of 0.5 mM [(L[1])$BiCl_2$] **(1)** at 0.1 M of Anilinium BF_4^- (pK_a 10.62 in acetonitrile) (black) and blank 0.1 M of Anilinium BF_4^- (pK_a 10.62 in acetonitrile) (red) with 0.1 M $TBAPF_6$ as a supporting electrolyte in acetonitrile; Scan rate = 100 mV/s. Condition: Working electrode glassy carbon (3 mm diameter), counter electrode Pt wire, and reference is Ag/AgCl. Data referenced to Fc/Fc^+ couple.

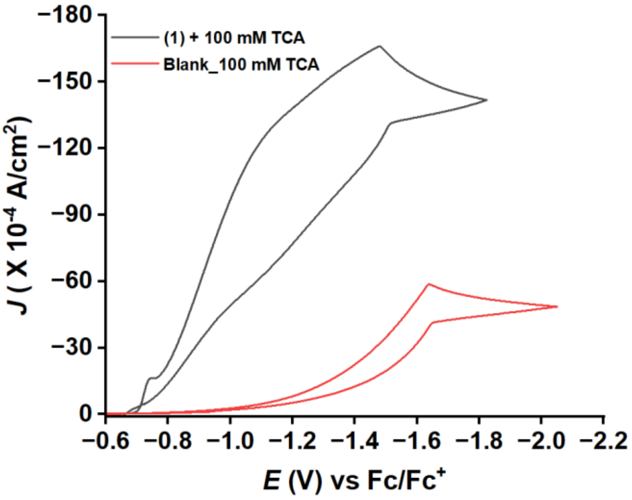

Figure S142. Cyclic Voltammogram of 0.5 mM [(L[1])BiCl$_2$] **(1)** at 0.1 M of TCA (pK_a 10.75 in acetonitrile) (black) and blank 0.1 M of TCA (pK_a 10.75 in acetonitrile) (red) with 0.1 M TBAPF$_6$ as a supporting electrolyte in acetonitrile; Scan rate = 100 mV/s. Condition: Working electrode glassy carbon (3 mm diameter), counter electrode Pt wire, and reference is Ag/AgCl. Data referenced to Fc/Fc$^+$ couple.

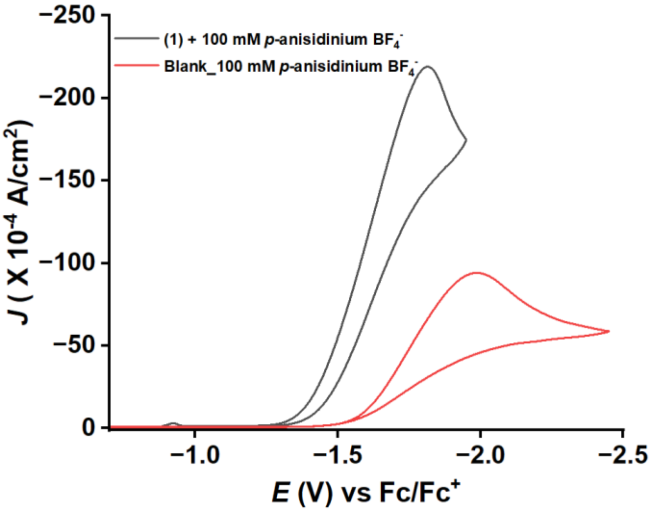

Figure S143. Cyclic Voltammogram of 0.5 mM [(L[1])BiCl$_2$] **(1)** at 0.1 M of (Anisidium)BF$_4^-$ (pK_a 11.86 in acetonitrile) (black) and blank 0.1 M of (Anisidium)BF$_4^-$ (pK_a 11.86 in acetonitrile) (red) with 0.1 M TBAPF$_6$ as a supporting electrolyte in acetonitrile; Scan rate = 100 mV/s. Condition: Working electrode glassy carbon (3 mm diameter), counter electrode Pt wire, and reference is Ag/AgCl. Data referenced to Fc/Fc$^+$ couple.

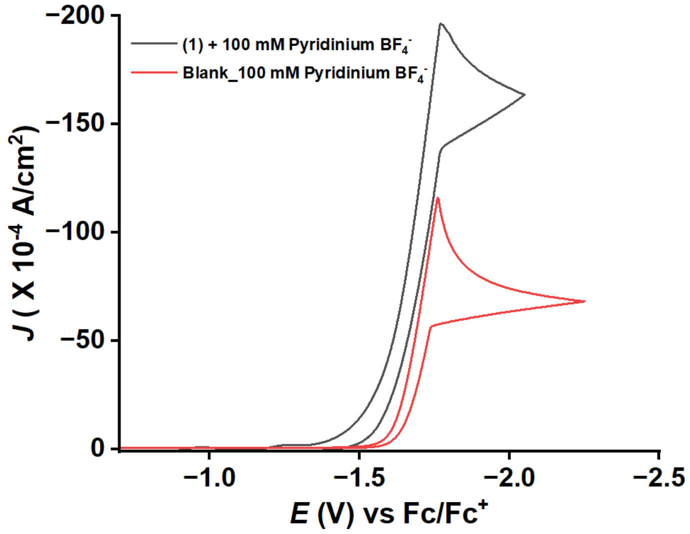

Figure S144a. Cyclic Voltammogram of 0.5 mM [(L[1])BiCl$_2$] **(1)** at 0.1 M of Pyridinium BF_4^- (pK_a 12.53 in acetonitrile) (black) and blank 0.1 M of Pyridinium BF_4^- (pK_a 12.53 in acetonitrile) (red) with 0.1 M TBAPF$_6$ as a supporting electrolyte in acetonitrile; Scan rate = 100 mV/s. Condition: Working electrode glassy carbon (3 mm diameter), counter electrode Pt wire, and reference is Ag/AgCl. Data referenced to Fc/Fc$^+$ couple.

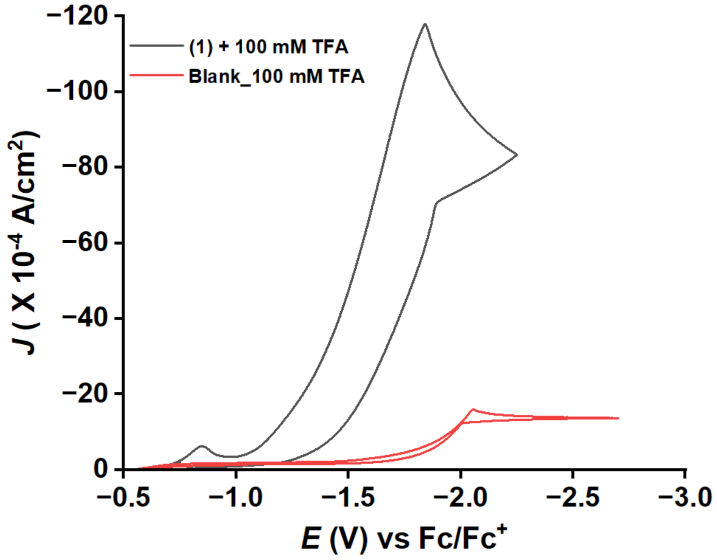

Figure S144b. Cyclic Voltammogram of 0.5 mM [(L[1])BiCl$_2$] **(1)** at 0.1 M of TFA (pK_a 12.65 in acetonitrile) (black) and blank 0.1 M of Pyridinium BF_4^- (pK_a 12.65 in acetonitrile) (red) with 0.1 M TBAPF$_6$ as a supporting electrolyte in acetonitrile; Scan rate = 100 mV/s. Condition: Working electrode glassy carbon (3 mm diameter), counter electrode Pt wire, and reference is Ag/AgCl. Data referenced to Fc/Fc$^+$ couple.

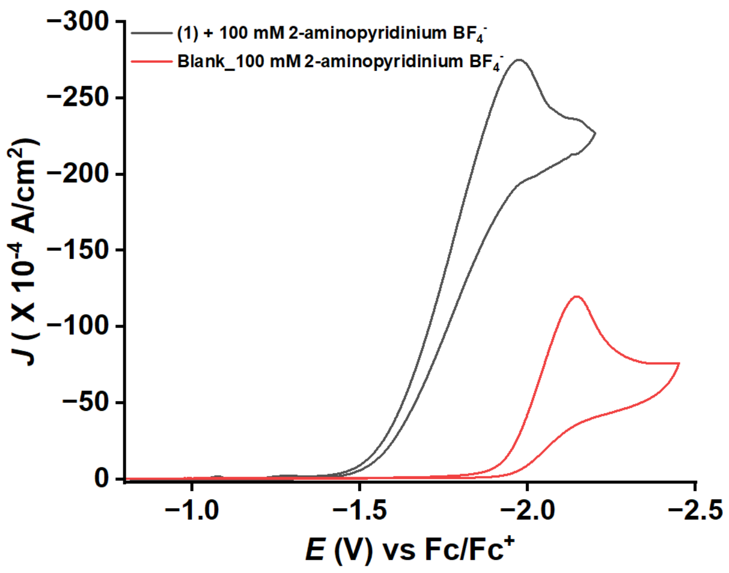

Figure S145. Cyclic Voltammogram of 0.5 mM [(L[1])BiCl$_2$] **(1)** at 0.1 M of 2-aminopyridinium BF_4^- (pK_a 14.47 in acetonitrile) (black) and blank 0.1 M of 2-aminopyridinium BF_4^- (pK_a 14.47 in acetonitrile) (red) with 0.1 M $TBAPF_6$ as a supporting electrolyte in acetonitrile; Scan rate = 100 mV/s. Condition: Working electrode glassy carbon (3 mm diameter), counter electrode Pt wire, and reference is Ag/AgCl. Data referenced to Fc/Fc^+ couple.

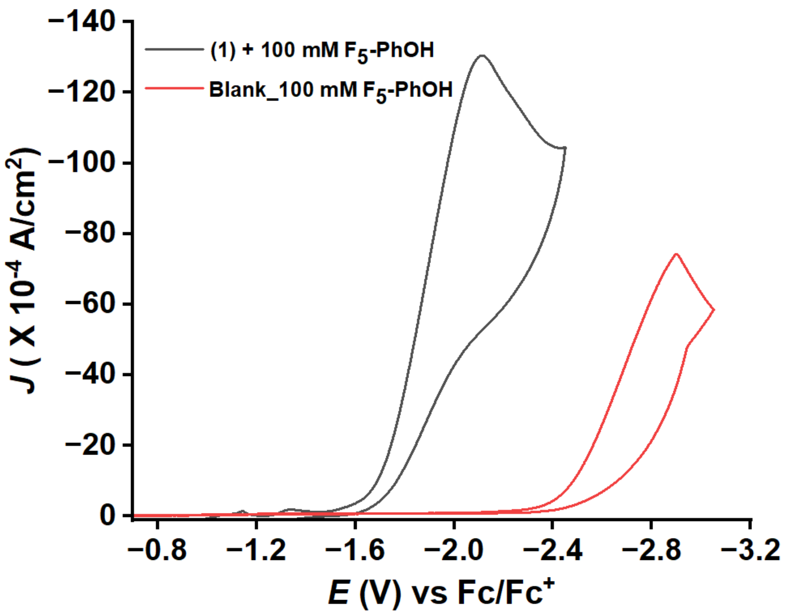

Figure S146. Cyclic Voltammogram of 0.5 mM [(L[1])BiCl$_2$] **(1)** at 0.1 M of F_5-PhOH (pK_a 20.1 in acetonitrile) (black) and blank 0.1 M of F_5-PhOH (pK_a 20.1 in acetonitrile) (red) with 0.1 M $TBAPF_6$ as a supporting electrolyte in acetonitrile; Scan rate = 100 mV/s. Condition: Working electrode glassy carbon (3 mm diameter), counter electrode Pt wire, and reference is Ag/AgCl. Data referenced to Fc/Fc^+ couple.

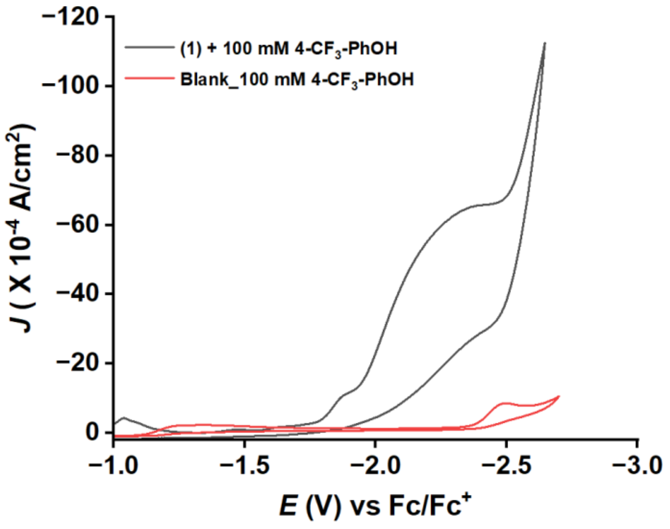

Figure S147. Cyclic Voltammogram of 0.5 mM [(L^1)$BiCl_2$] **(1)** at 0.1 M of 4-CF_3-PhOH (pK_a 25.54 in acetonitrile) (black) and blank 0.1 M of 4-CF_3-PhOH (pK_a 25.54 in acetonitrile) (red) with 0.1 M $TBAPF_6$ as a supporting electrolyte in acetonitrile; Scan rate = 100 mV/s. Condition: Working electrode glassy carbon (3 mm diameter), counter electrode Pt wire, and reference is Ag/AgCl. Data referenced to Fc/Fc^+ couple.

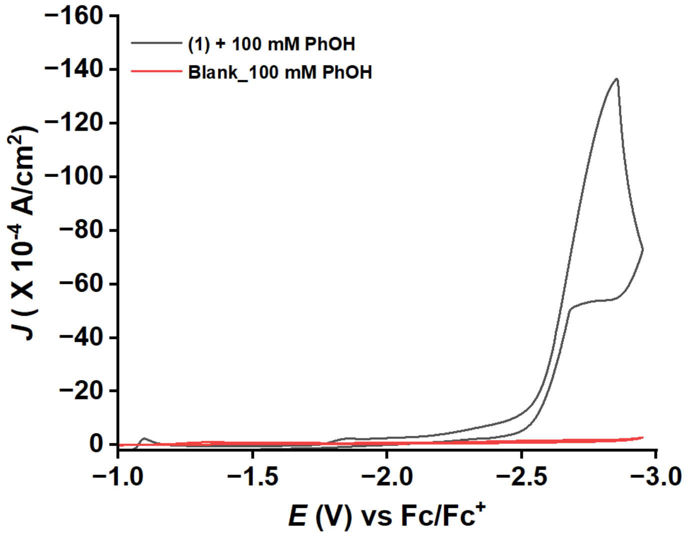

Figure S148. Cyclic Voltammogram of 0.5 mM [(L^1)$BiCl_2$] **(1)** at 0.1 M of PhOH (pK_a 29.4 in acetonitrile) (black) and blank 0.1 M of PhOH (pK_a 29.4 in acetonitrile) (red) with 0.1 M $TBAPF_6$ as a supporting electrolyte in acetonitrile; Scan rate = 100 mV/s. Condition: Working electrode glassy carbon (3 mm diameter), counter electrode Pt wire, and reference is Ag/AgCl. Data referenced to Fc/Fc^+ couple.

Comparative reactivity study of complexes (XI)

Table S11-S14 show the data recorded for proton reduction studies with different proton sources using catalyst $[(L^1)BiCl_2]$ **(1),** $[(L^2)BiCl_2]$ **(2),** $[(L^3)BiCl_2]$ **(3)**, $[(L^4)BiCl_2]$ **(4),** $[(L^5)BiCl_2]$ **(5),** $[(L^6)BiCl_2]$ **(6),** $[(L^47BiCl_2]$ **(7)** respectively. The turnover frequency (TOF) of all the catalysts used under different proton sources was calculated using the formula.[8]

$$\mathrm{TOF} = F\upsilon n_p^3((0.4463/n_{cat})(i_{cat}/i_p))^2/RT$$

where υ is the scan rate in V/s, n_p is the number of electrons transferred between the catalyst redox couple (n_p =2 for Bi^I/Bi^{III} couple), n_{cat} is the number of electrons required for the conversion of the substrate (here n_{cat} = 2, two electrons are required to convert 2 protons to give one H_2), i_{cat} is catalytic peak current, i_p is inherent current before the addition of substrate (acid, here) (Note: i_{cat}/i_p = J_{cat}/J_p). The results of TOF for different catalysts (1-4) are summarised in the tables (Table S11 to S14) below.[8]

The overpotential (η) for a half-cell reaction is defined as the additional potential (in excess of the thermodynamic requirement) needed to reach a specific current density or activity.

Calculating the Thermodynamic Potential for Proton Reduction

$$E_{H^+} = E^{\circ}{}_{H^+} + \frac{RT}{nF}\ln\frac{[H^+]}{P_{H_2}}$$

$$E_{H^+} = E^{\circ}{}_{H^+} - 0.05916\ \mathrm{V} \times \mathrm{pH}$$

Overpotential = $|E_{H^+} - E_X|$.[9]

E_X is $E_{cat/2}$ *for* $\boldsymbol{\eta_1}$ **(by $\boldsymbol{E_{cat/2}}$)**

E_X is E_{red} *for* $\boldsymbol{\eta_2}$ **(by $\boldsymbol{E_{red}}$)**

In the manuscript overpotential η_1 (by $E_{cat/2}$) has been used.

Table S11. Results of proton reduction studies with various proton sources using catalyst **[(L¹)BiCl₂] (1)**

Proton source	$E_{cat/2}$ (V)	E_{red} (V)	Onset potential (V)	TOF (s^{-1})	$E°_{H+/H2}$ (V)	**η_1 (by $E_{cat/2}$) (V)**	**η_2 (by E_{red}) (V)**
[H-DMF⁺] (pK_a = 6.1)	−1.33	−1.14	−0.97	3.1 x 10^6	−0.39	0.945	0.750
4-Cl-Anilinium BF_4^- (pK_a = 9.7)	−1.41	−1.14	−1.11	2.6 x 10^6	−0.601	0.81	0.538
Anilinium BF_4^- (pK_a = 10.62)	−1.51	−1.14	−1.21	3.3 x 10^6	−0.640	0.87	0.500
TCA (pK_a = 10.75)	−0.96	−1.14	−0.72	1.6 x 10^6	−0.664	0.30	0.475
p-anisidium BF_4^- (pK_a = 11.86)	−1.60	−1.14	−0.90	1.8 x 10^6	−0.702	0.90	0.437
Pyridinium BF_4^- (pK_a = 12.53)	−1.67	−1.14	−1.36	1.4 x 10^6	−0.769	0.91	0.371
TFA (pK_a = 12.65)	−1.15	−1.14	−0.68	3.0 x 10^5	−0.776	0.37	0.364
2-aminopyridinium BF_4^- (pK_a = 14.47)	−1.75	−1.14	−1.41	2.8 x 10^6	−0.884	0.87	0.255
F_5-PhOH (pK_a = 20.1)	−1.87	−1.14	−1.51	7.1 x 10^5	−1.217	0.66	0.0770
4- CF_3-PhOH (pK_a = 25.54)	−2.04	−1.14	−1.61	1.8 x 10^5	−1.534	0.50	0.394
PhOH (pK_a = 29.4)	−2.68	−1.14	−2.19	7.1 x 10^5	−1.768	0.91	0.628

Table S12. Results of proton reduction studies with various proton sources using catalyst **(L^2)BiCl$_2$] (2)**

Proton source	$E_{cat/2}$ (V)	E_{red} (V)	Onset potential (V)	TOF (s^{-1})	$E°_{H+/H2}$(V)	**η_1 (by $E_{cat/2}$) (V)**	**η_1 (by E_{red}) (V)**
[H-DMF$^+$] (pK_a = 6.1)	−1.49	−1.09	−1.15	1.3 x 10^6	−0.39	1.10	0.700
4-Cl-Anilinium BF_4^- (pK_a = 10.62	−1.46	−1.09	−1.102	7.4 x 10^5	−0.601	0.86	0.489
Anilinium BF_4^- (pK_a = 10.62)	−1.57	−1.09	−1.33	7.1 x 10^5	−0.64	0.93	0.450
TCA (pK_a = 10.75)	−0.99	−1.09	−0.76	2.4 x 10^5	−0.664	0.43	0.425
p-anisidium BF_4^- (pK_a = 11.86)	−1.62	−1.09	−1.38	7.4 x 10^5	−0.702	0.91	0.388
Pyridinium BF_4^- (pK_a = 12.53)	−1.67	−1.09	−1.48	2.3 x 10^5	−0.769	0.89	0.321
TFA (pK_a = 12.65)	−1.43	−1.09	−1.12	6.3 x 10^4	−0.776	0.65	0.313
2-aminopyridinium BF_4^- (pK_a = 14.47)	−1.83	−1.09	−1.55	8.8 x 10^5	−0.884	0.95	0.2054
F_5-PhOH (pK_a = 20.1)	−1.98	−1.09	−1.7	1.5 x 10^5	−1.217	0.76	0.127
4- CF_3-PhOH (pK_a = 25.54)	−2.08	−1.09	−1.81	5.8 x 10^4	−1.534	0.54	0.444
PhOH (pK_a = 29.4)	−2.31	−1.09	−2.13	1.2 x 10^5	−1.768	0.55	0.678

Table S13. Results of proton reduction studies with various proton sources using catalyst **[(L^3)$BiCl_2$] (3)**

Proton source	$E_{cat/2}$ (V)	E_{red} (V)	Onset potential (V)	TOF (s^{-1})	$E°_{H+/H2}$(V)	***η₁*** **(by** $E_{cat/2}$**) (V)**	***η₁*** **(by** E_{red}**) (V)**
[H-DMF$^+$] (pK_a = 6.1)	−1.48	−1.27	−1.18	1.9 x 10^6	−0.390	1.09	0.880
Anilinium BF_4^- (pK_a = 10.62)	−1.59	−1.27	−1.02	2.1 x 10^6	−0.640	0.96	0.320
TCA (pK_a = 10.75)	−1.41	−1.27	−0.89	7.2 x 10^5	−0.664	0.74	0.605
p-anisidium BF_4^- (pK_a = 11.86)	−1.62	−1.27	−1.31	1.8 x 10^6	−0.702	0.92	0.570
Pyridinium BF_4^- (pK_a = 12.53)	−1.42	−1.27	−1.42	3.3 x 10^6	−0.769	0.90	0.501
TFA (pK_a = 12.65)	−1.54	−1.27	−1.04	2.3 x 10^5	−0.776	0.77	0.490
2-aminopyridinium BF_4^- (pK_a = 14.47)	−1.798	−1.27	−1.42	2.7 x 10^6	−0.884	0.91	0.385
F_5-PhOH (pK_a = 20.1)	−2.10	−1.27	−1.72	3.4 x 10^5	−1.217	0.89	0.060
4- CF_3-PhOH (pK_a = 25.54)	−2.59	−1.27	−1.61	2.2 x 10^5	−1.534	1.05	0.264
PhOH (pK_a = 29.4)	−2.74	−1.27	−2.24	2.6 x 10^5	−1.768	0.97	0.498

Table S14. Results of proton reduction studies with various proton sources using catalyst **[(L^4)$BiCl_2$] (4).**

Proton source	$E_{cat/2}$ (V)	E_{red} (V)	Onset potential (V)	TOF (s^{-1})	$E°_{H+/H2}$(V)	***η₁* (by $E_{cat/2}$) (V)**	***η₁* (by E_{red}) (V)**
[H-DMF$^+$] (pK_a = 6.1)	−1.366	−1.12	−1.07	6.9 x 10^5	−0.390	0.98	0.730
Anilinium BF_4^- (pK_a = 10.62)	−1.574	−1.12	−1.29	7.3 x 10^5	−0.640	0.92	0.470
TCA (pK_a = 10.75)	−1.016	−1.12	−0.79	3.2 x 10^5	−0.664	0.35	0.455
p-anisidium BF_4^- (pK_a = 11.86)	−1.626	−1.12	−1.32	7.2 x 10^5	−0.702	0.92	0.417
Pyridinium BF_4^- (pK_a = 12.53)	−1.702	−1.12	−1.52	2 x 10^5	−0.769	0.93	0.350
TFA (pK_a = 12.65)	−1.56	−1.12	−1.17	9.3 x 10^4	−0.776	0.73	0.343
2-aminopyridinium BF_4^- (pK_a = 14.47)	−1.842	−1.12	−1.54	7.6 x 10^5	−0.884	0.96	0.235
F_5-PhOH (pK_a = 20.1)	−1.996	−1.12	−1.69	1.8 x 10^5	−1.217	0.77	0.097
4- CF_3-PhOH (pK_a = 25.54)	−2.178	−1.12	−1.84	3.7 x 10^4	−1.534	0.64	0.414
PhOH (pK_a = 29.4)	−2.394	−1.12	−2.32	5.4 x 10^3	−1.768	0.62	0.648

Table S15. Results of proton reduction studies with various proton sources using catalyst **[(L^5)$BiCl_2$] (5).**

Proton source	$E_{cat/2}$ (V)	E_{red} (V)	Onset potential (V)	TOF (s^{-1})	$E°_{H+/H2}$ (V)	η_1 (by $E_{cat/2}$) (V)	η_2 (by E_{red}) (V)
[H-DMF$^+$] (pK_a = 6.1)	−1.46	−0.95	−1.16	5.4 x 10^5	−0.39	1.07	0.560
p-anisidium BF_4^- (pK_a = 11.86)	−1.622	−0.95	−1.28	4.3 x 10^5	−0.7021	0.92	0.247
TFA (pK_a = 12.65)	−1.606	−0.95	−1.16	5.8 x 10^4	−0.7768	0.82	0.173
2-aminopyridinium BF_4^- (pK_a = 14.47)	−1.834	−0.95	−1.44	6.2 x 10^5	−0.8846	0.95	0.065
F_5-PhOH (pK_a = 20.1)	−1.85	−0.95	−1.51	1.7 x 10^5	−1.217	0.63	0.260

Table S16. Comparison of Results of TOF and overpotential with various proton sources using catalyst **[(L^1)$BiCl_2$] (1) and [(L^3)$BiCl_2$] (3).**

Proton source	[(L^1)$BiCl_2$] (1) TOF (s^{-1})	[(L3)$BiCl_2$] (3) TOF (s^{-1})	[(L^1)$BiCl_2$] (1) η_1 (by $E_{cat/2}$) (V)	[(L^3)$BiCl_2$] (3) η_1 (by $E_{cat/2}$) (V)
[H-DMF$^+$] (pK_a = 6.1)	3.1 x 10^6	1.9 x 10^6	0.945	1.09
Anilinium BF_4^- (pK_a = 10.62)	3.3 x 10^6	7.2 x 10^5	0.903	0.956
TCA (pK_a = 10.75)	1.6 x 10^6	1.8 x 10^6	0.301	0.7476
p-anisidium BF_4^- (pK_a = 11.86)	1.8 x 10^6	3.3 x 10^6	0.806	0.924
Pyridinium BF_4^- (pK_a = 12.53)	1.4 x 10^6	2.3 x 10^5	0.906	0.897
TFA (pK_a = 12.65)	3.0 x 10^5	2.7 x 10^6	0.313	0.7672
2-aminopyridinium BF_4^- (pK_a = 14.47)	2.8 x 10^6	3.4 x 10^5	0.867	0.9134
F_5-PhOH (pK_a = 20.1)	7.1 x 10^5	2.2 x 10^5	0.656	0.883
4- CF_3-PhOH (pK_a = 25.54)	1.8 x 10^5	2.6 x 10^5	0.497	1.056
PhOH (pK_a = 29.4)	7.1 x 10^5	1.9 x 10^6	0.907	0.972

Table S17. Comparison of results of proton reduction studies with F_5-PhOH (pK_a = 20.1 in acetonitrile) as proton source using catalyst **[(L^1)$BiCl_2$] (1), [(L^2)$BiCl_2$] (2) and [(L^7)$BiCl_2$] (7).**

Proton source	**TOF (s^{-1})**	***E*$_{cat/2}$ (V)**	**[(η_1 (by $E_{cat/2}$) (V)**
**[(L^1)$BiCl_2$] (1)**	7.1 x 10^5	−1.87	0.65
**[(L^2)$BiCl_2$] (2)**	1.5 x 10^5	−1.98	0.76
**[(L^7)$BiCl_2$] (7)**	1.4 x 10^5	−2.01	0.80

Table S18. Comparison of Results of proton reduction studies with TFA (pK_a = 12.65 in acetonitrile) as proton source using catalyst **[(L^1)$BiCl_2$] (1), [(L^2)$BiCl_2$] (2) and [(L^7)$BiCl_2$] (7).**

Proton source	**TOF (s^{-1})**	***E*$_{cat/2}$ (V)**	**[(η_1 (by $E_{cat/2}$) (V)**
**[(L^1)$BiCl_2$] (1)**	3.0 x 10^5	−1.15	0.38
**[(L^2)$BiCl_2$] (2)**	6.3 x 10^4	−1.43	0.65
**[(L^7)$BiCl_2$] (7)**	3.4 x 10^4	−1.17	0.99

Table S19. Comparison of Results of proton reduction studies with F_5-PhOH (pK_a = 20.1 in acetonitrile) as proton source using catalyst [(L^1)$BiCl_2$] **(1),** [(L^2)$BiCl_2$] **(2),** [(L^3)$BiCl_2$] **(3),** [(L^4)$BiCl_2$] **(4),** [(L^5)$BiCl_2$] **(5),** [(L^6)$BiCl_2$] **(6).**

Proton source	**TOF (s^{-1})**	***E*$_{cat/2}$ (V)**	**[(η_1 (by $E_{cat/2}$) (V)**
**[(L^1)$BiCl_2$] (1)**	7.1 x 10^5	−1.87	0.65
**[(L^2)$BiCl_2$] (2)**	1.5 x 10^5	−1.98	0.76
**[(L^3)$BiCl_2$] (3)**	3.4 x 10^5	−1.55	0.88
**[(L^4)$BiCl_2$] (4)**	1.8 x 10^5	−1.99	0.77
**[(L^5)$BiCl_2$] (5)**	1.7 x 10^5	−1.85	0.63
**[(L^6)$BiCl_2$] (6)**	2.5 x 10^5	−1.79	0.58

Table S20. Comparison of Results of proton reduction studies with TFA (pK_a = 12.65 in acetonitrile) as proton source using catalyst [(L^1)BiCl$_2$] **(1),** [(L^2)BiCl$_2$] **(2),** [(L^3)BiCl$_2$] **(3)**, [(L^4)BiCl$_2$] **(4),** [(L^5)BiCl$_2$] **(5)** , [(L^6)BiCl$_2$] **(6).**

Proton source	**TOF (s^{-1})**	**$E_{cat/2}$ (V)**	**[(η_1 (by $E_{cat/2}$) (V)**
**[(L^1)BiCl$_2$] (1)**	3 x 10^5	−1.15	0.376
**[(L^2)BiCl$_2$] (2)**	6.3 x 10^4	−1.43	0.653
**[(L^3)BiCl$_2$] (3)**	2.3 x 10^5	−2.10	0.767
**[(L^4)BiCl$_2$] (4)**	9.3 x 10^4	−1.56	0.738
**[(L^5)BiCl$_2$] (5)**	5.8 x 10^4	−1.60	0.829
**[(L^6)BiCl$_2$] (6)**	9.3 x 10^4	−1.47	0.698

To validate the effect of alkyl substituent on the pincer backbone we summarised the proton reduction activity of catalyst catalyst [(L^1)BiCl$_2$] **(1),** [(L^2)BiCl$_2$] **(2),** [(L^3)BiCl$_2$] **(3)**, [(L^4)BiCl$_2$] **(4),** [(L^5)BiCl$_2$] **(5)**.

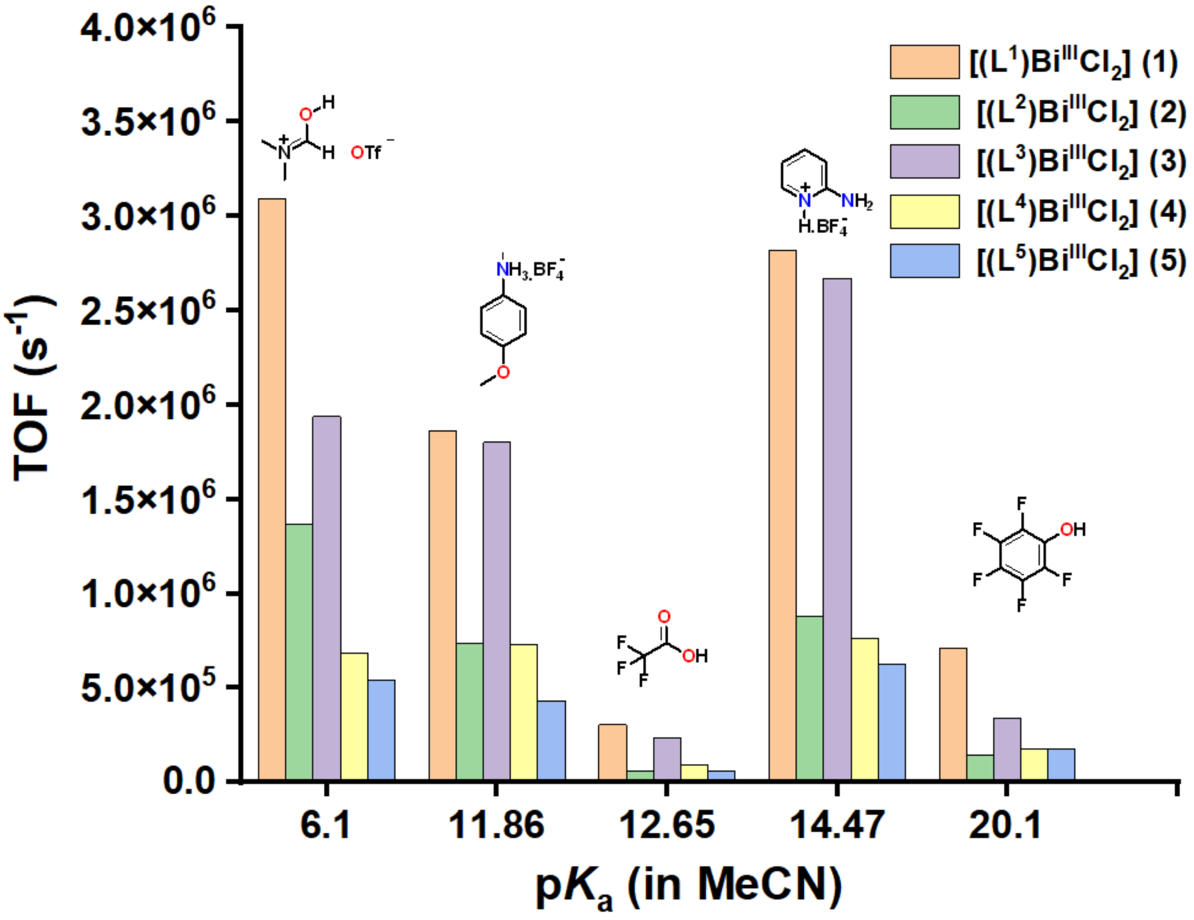

Figure S149. Plot of TOF for proton reduction vs pK_a (in acetonitrile) at 0.1 M concentration of the proton sources in presence of 0.5 mM of catalyst [(L^1)BiCl$_2$] **(1),** [(L^2)BiCl$_2$] **(2),** [(L^3)BiCl$_2$] **(3)**, [(L^4)BiCl$_2$] **(4),** [(L^5)BiCl$_2$] **(5).**

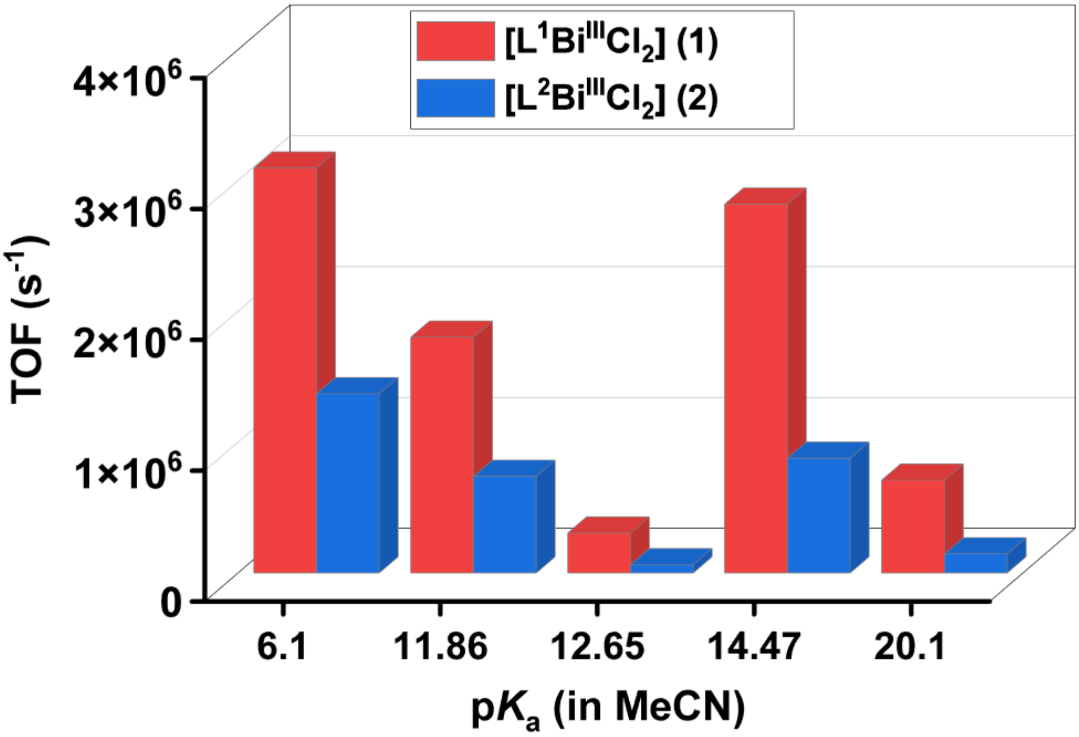

Figure S150. Complied plot of TOF values for 0.1 M of five acid as external proton sources in acetonitrile vs catalyst [(L^1)BiCl$_2$] **(1)** and [(L^2)BiCl$_2$] **(2)**.

Figure below summarises the activity trend of all the catalysts used in this study, where a plot of TOF vs pK_a is shown separately for each catalyst.

Plot of TOF vs pK_a

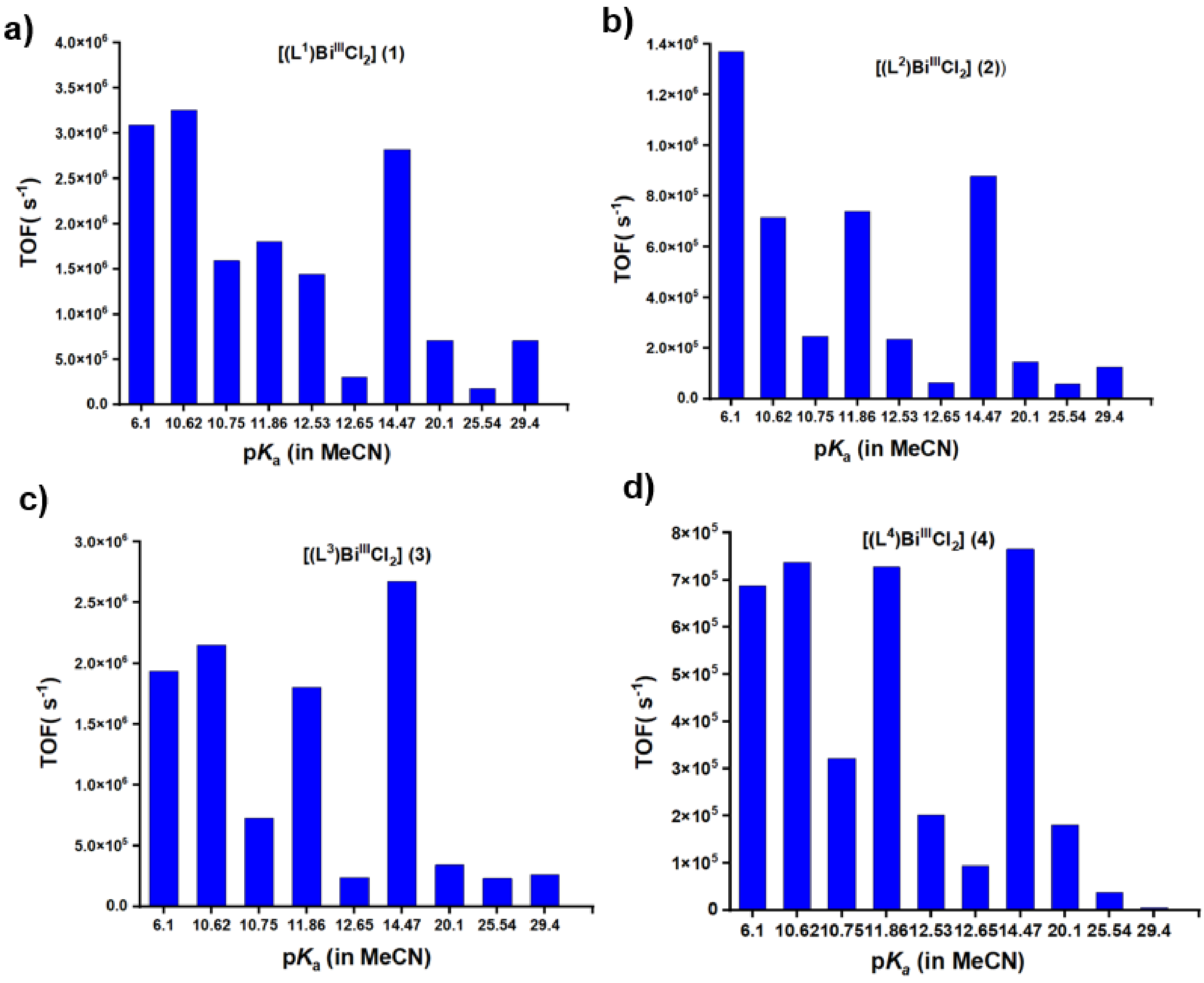

Figure S151. Plot of TOF vs pK_a for all four catalysts (in acetonitrile) at 0.1 M concentration of the proton sources in presence of 0.5 mM of a) $[(L^1)BiCl_2]$ **(1)** b) $[(L^2)BiCl_2]$ **(2)** c) $[(L^3)BiCl_2]$ **(3)** d) $[(L^4)BiCl_2]$ **(4)**, for all acid.

Plot of catalytic half wave potential ($E_{cat/2}$, V) for catalyst ([L^1BiCl_2] **(1),** [(L^2)$BiCl_2$] **(2),** [(L^3)$BiCl_2$] **(3),** [(L^4)$BiCl_2$] **(4))** vs pK_a for different external proton sources for HER

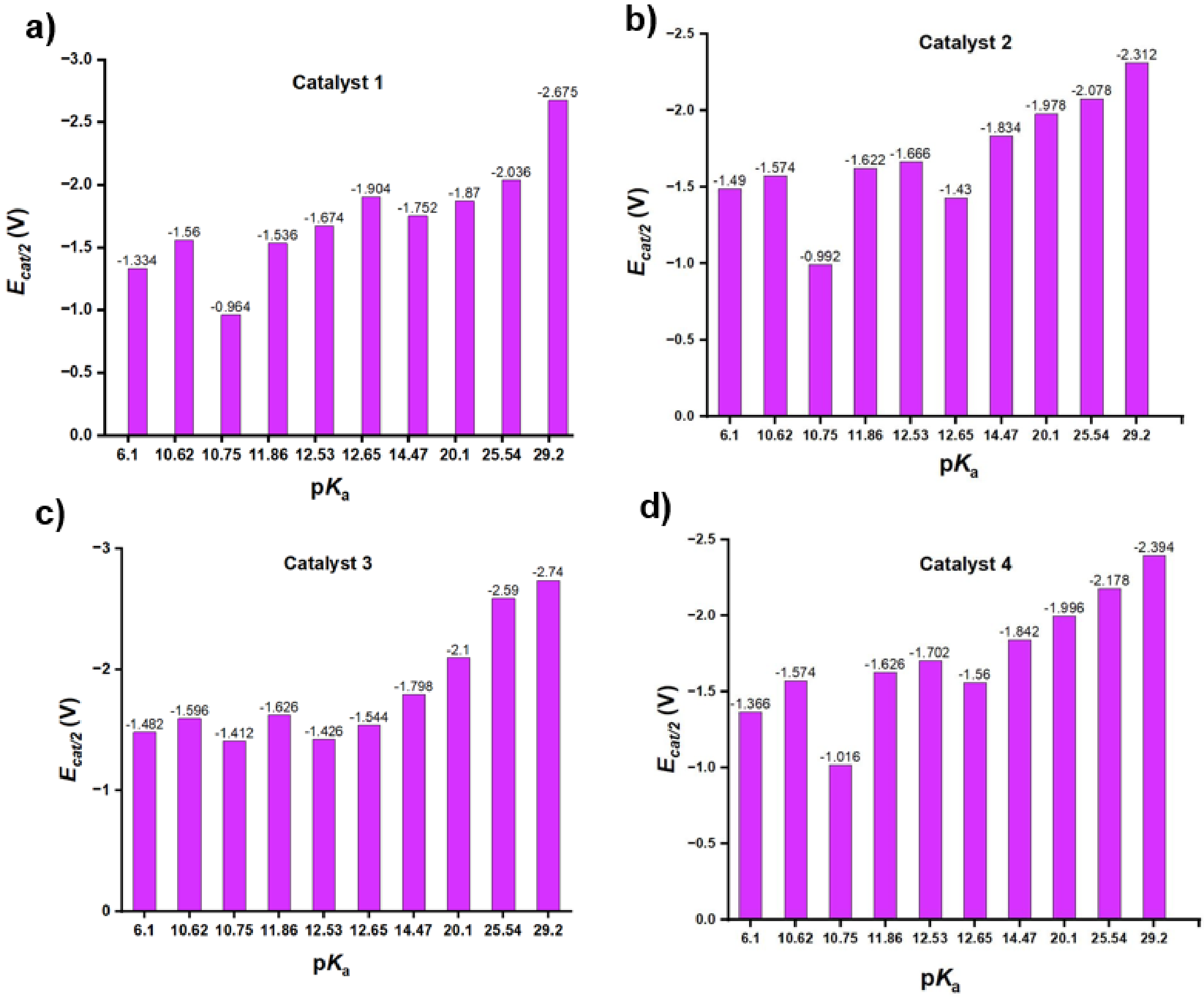

Figure S152. Plot of half-wave potential $E_{cat/2}$ (vs Fc/Fc^+) of catalytic wave for proton reduction vs pK_a (in acetonitrile) at 0.1 M concentration of the proton sources in presence of 0.5 mM of a) [(L^1)$BiCl_2$] **(1)** b) [(L^2)$BiCl_2$] **(2)** c) [(L^3)$BiCl_2$] **(3)** d) [(L^4)$BiCl_2$] **(4)**, for all 10 acids.

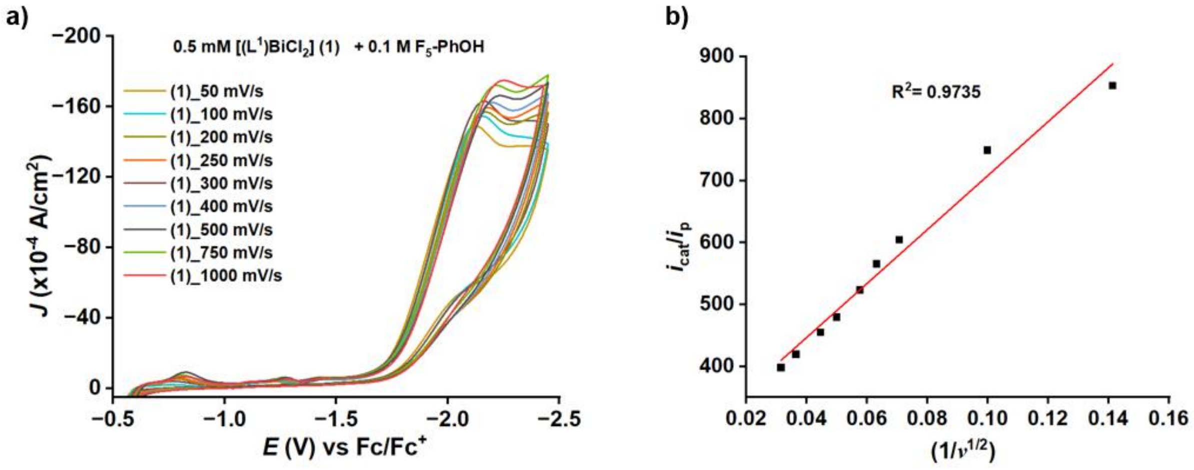

Figure S153. a) Scan rate independent Cyclic Voltammogram of 0.5 mM [(L^1)BiCl$_2$] **(1)** at 0.1 M of pentafluorophenol (pK_a 20.1 in acetonitrile) with 0.1 M TBAPF$_6$ as a supporting electrolyte in acetonitrile; varying scan rate (50 mV/s to 1000 mV/s). Condition: Working electrode glassy carbon (3 mm diameter), counter electrode Pt wire, and reference is Ag/AgCl. Data referenced to Fc/Fc$^+$ couple. **b)** Linear plot of i_{cat}/i_p vs $1/v^{1/2}$ depicts scan rate independent CVs using equation TOF = $F\upsilon n_p^3((0.4463/n_{cat})(i_{cat}/i_p))^2/RT$

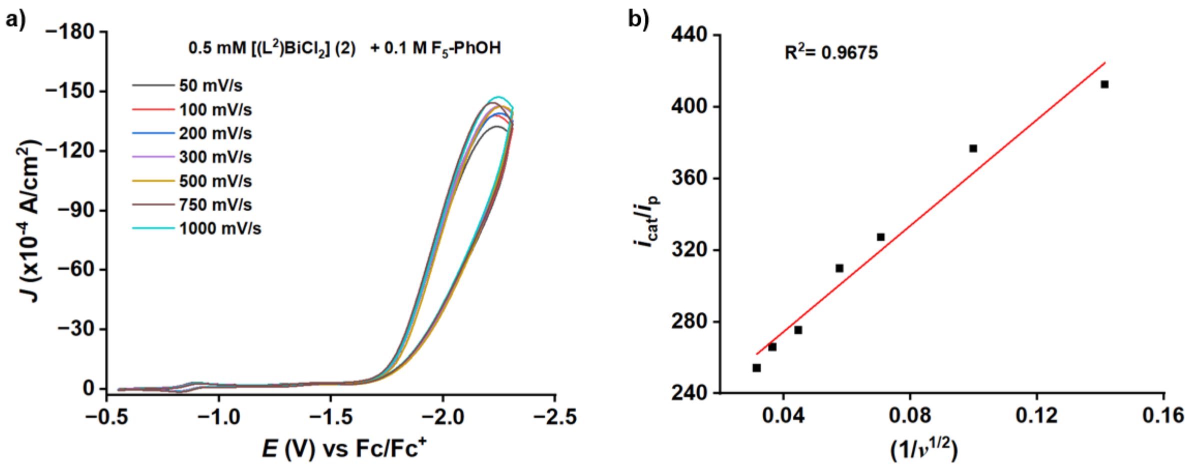

Figure S154. a) Scan rate independent Cyclic Voltammogram of 0.5 mM [(L^2)BiCl$_2$] **(2)** at 0.1 M of pentafluorophenol (pK_a 20.1 in acetonitrile) with 0.1 M TBAPF$_6$ as a supporting electrolyte in acetonitrile; varying scan rate. Condition: Working electrode glassy carbon (3 mm diameter), counter electrode Pt wire, and reference is Ag/AgCl. Data referenced to Fc/Fc$^+$ couple. **b)** Linear plot of i_{cat}/i_p vs $1/v^{1/2}$ depicts scan rate independent CVs using equation TOF = $F\upsilon n_p^3((0.4463/n_{cat})(i_{cat}/i_p))^2/RT$

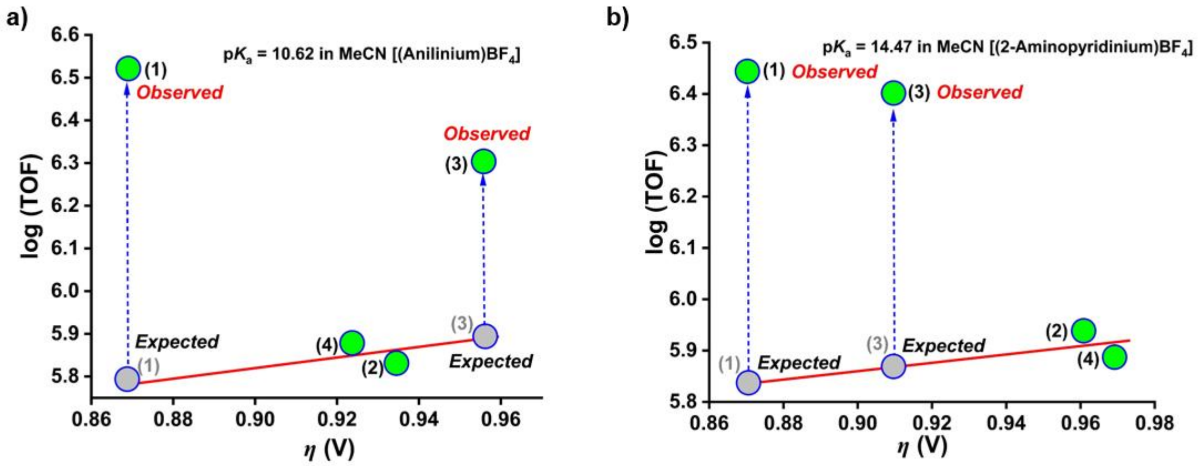

Figure S155 (a). Linear Free Energy Relationship (LFER) plot showing log(TOF) with η (V) for HER (in acetonitrile) at **(a)** 0.1 M anilinium BF_4^- (pK_a 10.62 in acetonitrile) and **(b)** 0.1 M 2-aminopyridinium BF_4^- (pK_a 14.47 in acetonitrile) with 0.1 M $TBAPF_6$ as a supporting electrolyte Condition: Scan rate = 100 mV/s, 0.5 mM of different catalyst [(L^1)$BiCl_2$] **(1) (a)**, [(L^2)$BiCl_2$] **(2),** [(L^3)$BiCl_2$] **(3)**, [(L^4)$BiCl_2$] **(4)**.

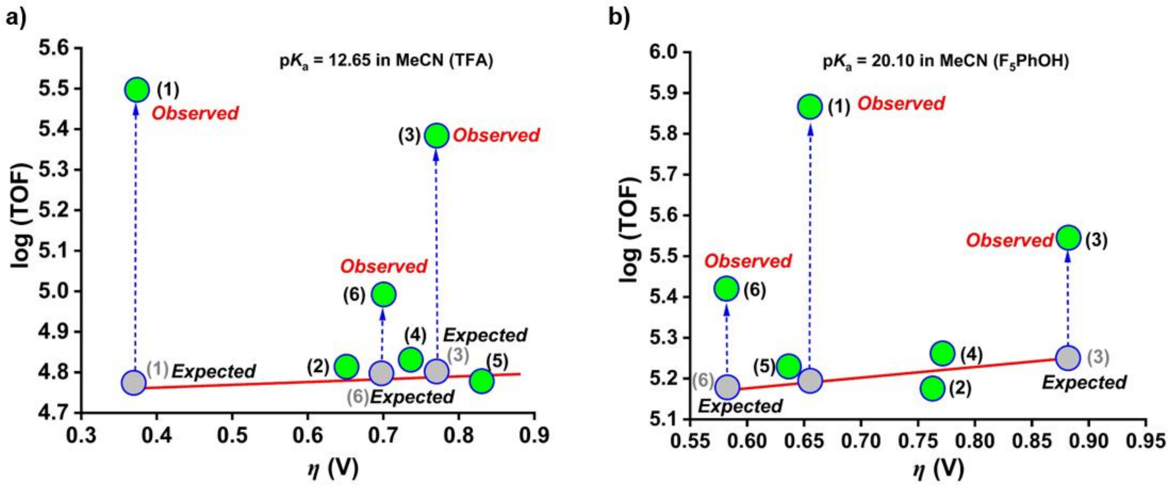

Figure S156 (a). Linear Free Energy Relationship (LFER) plot showing log(TOF) with η (V) for HER (in acetonitrile) at **(a)** 0.1 M TFA (pK_a 12.65 in acetonitrile) and **(b)** 0.1 M pentafluorophenol (pK_a 20.10 in acetonitrile) with 0.1 M $TBAPF_6$ as a supporting electrolyte Condition: Scan rate = 100 mV/s, 0.5 mM of different catalyst [(L^1)$BiCl_2$] **(1)**, [(L^2)$BiCl_2$] **(2),** [(L^3)$BiCl_2$] **(3)**, [(L^4)$BiCl_2$] **(4)**, [(L^5)$BiCl_2$] **(5),** [(L^6)$BiCl_2$] **(6).**

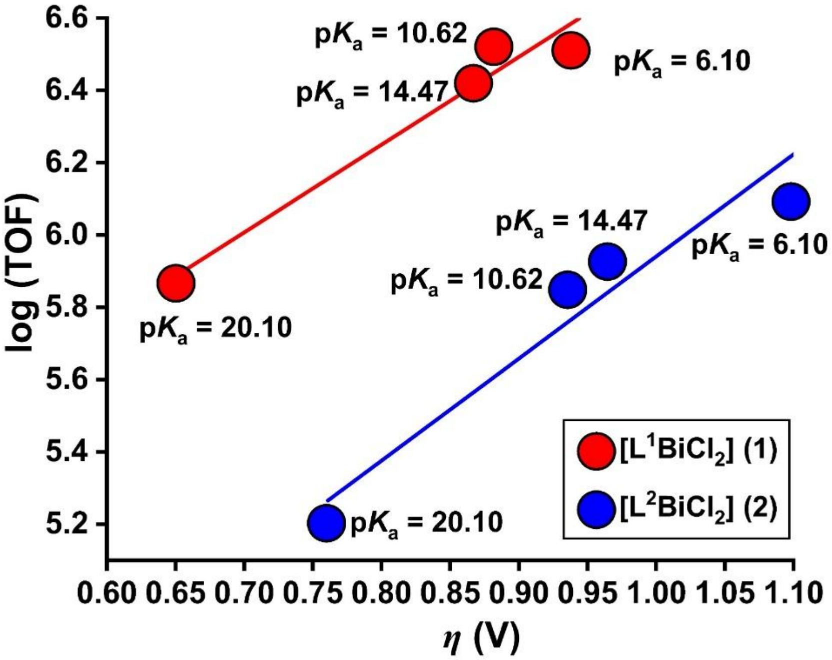

Figure S157 (a). Linear Free Energy Relationship (LFER) plot showing log(TOF) with η (V) for HER (in acetonitrile) for different acids of varying the pK_a of proton sources keeping catalyst constant. Red plots showing 0.5 mM of catalyst [(L^1)$BiCl_2$] **(1),** and blue plot showing 0.5 mM catalyst [(L^2)$BiCl_2$] **(2).** Condition: 0.1 M $TBAPF_6$ as a supporting electrolyte, scan rate = 100 mV/s.

Foot-Of-The Wave Analysis (FOWA) (XII)

TOF of electrocatalysts using the formula based on i_{cat}/i_p values, sometimes are an over interpretation. To validate the activity of the catalysts more accurately, we have taken the foot of the wave analyses (FOWA) approach which reveals the initial rate for predicting activity. FOWA for our developed catalysts ([$(L^1)BiCl_2$] **(1),** [$(L^2)BiCl_2$] **(2),** [$(L^3)BiCl_2$] **(3)**, [$(L^4)BiCl_2$] **(4)**) were performed using 0.1 M of different proton sources in acetonitrile.[10],[11],[12]

Foot-of-the-Wave Analysis (FOWA) is used to determine the kinetic parameters such as TOF_{max} of an electrocatalyst from cyclic voltammetry data by analyzing the initial rising portion ("foot") of the catalytic wave, where mass transport limitations are minimal. From CV utilising i_{cat} , i_p, $E_{cat/2}$ and E at different range a plot of $\frac{i_{cat}}{i_p}$ versus $1/\left[1+\exp\left(\left(\frac{nF}{RT}\right)\left(E-E_{\frac{cat}{2}}\right)\right)\right]$ was plotted. Points were chosen from foot region of the plot ensuring that the observed current reflects intrinsic catalytic kinetics rather than diffusion limitations, and fitted linearly to give a slope. Where slope = [2.24 × n × $\sqrt{(RT / Fv)}$ × $\sqrt{(TOF_{max})}$], putting n = 2, TOF_{max} is calculated from the slope obtained by fitting the low-potential region of the catalytic wave and putting the value in formula.

$$i_{cat}/i_p = [2.24 \times n \sqrt{(RT / Fv)} \times \sqrt{(TOF_{max})}] / [1 + \exp((nF/RT)(E - E_{cat/2}))]$$

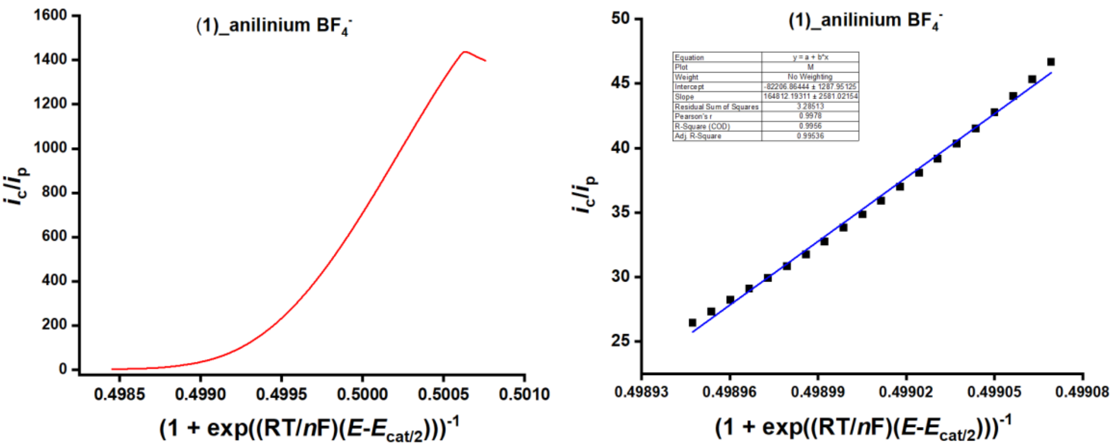

Figure S158. a) Plot for i_c/i_p vs (1 + exp((RT/*n*F)E-$E_{cat/2}$))$^{-1}$ from onset to peak position taken from CV for 0.1 M anilinium tetrafluoroborate (pK_a 10.62 in acetonitrile) in presence of 0.5 mM [$(L^1)BiCl_2$] **(1)**. b) Corresponding FOWA plot.

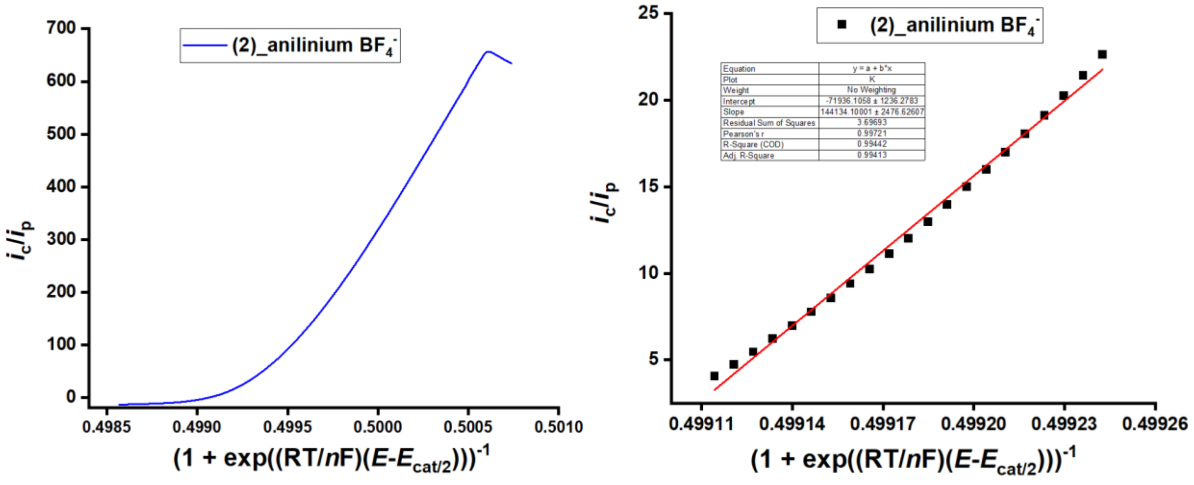

Figure S159. a) Plot for i_c/i_p vs (1 + exp((RT/nF)E-$E_{cat/2}$))$^{-1}$ from onset to peak position taken from CV for 0.1 M anilinium tetrafluoroborate (pK_a 10.62 in acetonitrile) in presence of 0.5 mM [(L^2)BiCl$_2$] **(2)**. b) Corresponding FOWA plot.

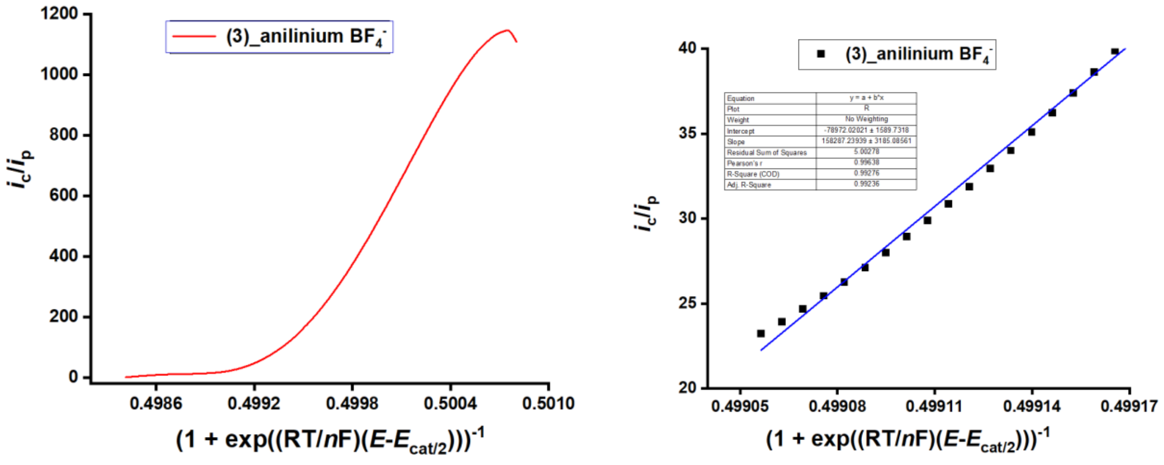

Figure S160. a) Plot for i_c/i_p vs (1 + exp((RT/nF)E-$E_{cat/2}$))$^{-1}$ from onset to peak position taken from CV for 0.1 M anilinium tetrafluoroborate (pK_a 10.62 in acetonitrile) in presence of 0.5 mM [(L^3)BiCl$_2$] **(3)**. b) Corresponding FOWA plot.

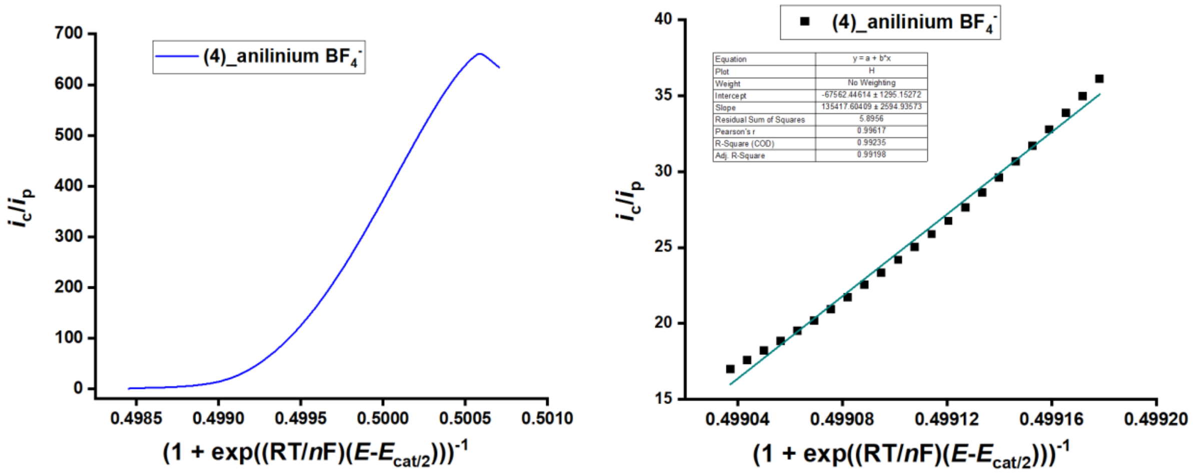

Figure S161. a) Plot for i_c/i_p vs (1 + exp((RT/nF)E-$E_{cat/2}$))$^{-1}$ from onset to peak position taken from CV for 0.1 M anilinium tetrafluoroborate (pK_a 10.62 in acetonitrile) in presence of 0.5 mM [(L^4)BiCl$_2$] **(4)**. b) Corresponding FOWA plot.

The calculated TOF_{max} from FOWA of 0.1 M anilinium tetrafluoroborate in presence of 0.5 mM catalyst were found to be 5.27 x 10^9 s^{-1}, 4.03 x 10^9 s^{-1}, 4.86 x 10^9 s^{-1}, and 3.5 x 10^9 s^{-1} for [(L^1)BiCl$_2$] **(1),** [(L^2)BiCl$_2$] **(2),** [(L^3)BiCl$_2$] **(3)**, [(L^4)BiCl$_2$] **(4)** respectively.

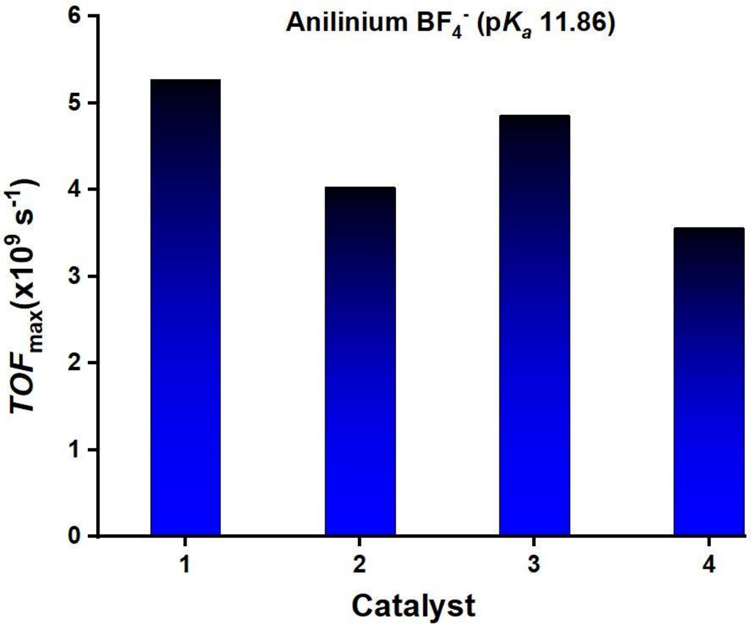

Figure S162. Plot of TOF$_{max}$ values obtained from FOWA for 0.1 M anilinium tetrafluoroborate (pK_a 10.62 in acetonitrile) as external proton source for different Bi(III) catalysts [(L^1)BiCl$_2$] **(1),** [(L^2)BiCl$_2$] **(2),** [(L^3)BiCl$_2$] **(3)**, [(L^4)BiCl$_2$] **(4)**.

FOWA for catalysts **(**[(L^1)BiCl$_2$] **(1),** [(L^2)BiCl$_2$] **(2),** [(L^3)BiCl$_2$] **(3)**, [(L^4)BiCl$_2$] **(4))** using 0.1 M TFA (pK_a =12.65 in acetonitrile).

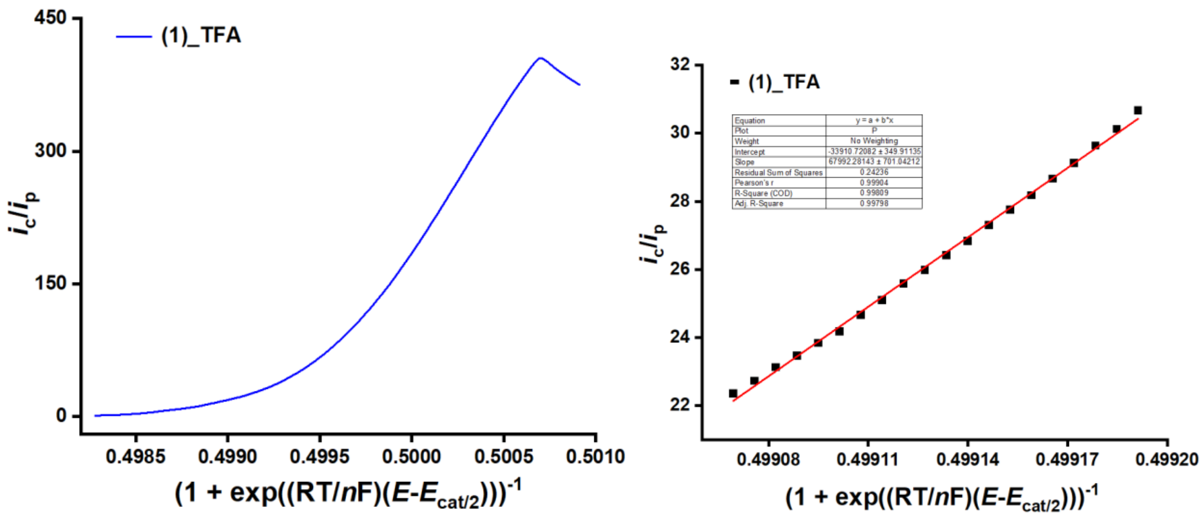

Figure S163. a) Plot for i_c/i_p vs (1 + exp((RT/nF)E-$E_{cat/2}$))$^{-1}$ from onset to peak position taken from CV for 0.1 M TFA (pK_a 12.65 in acetonitrile) in the presence of 0.5 mM [(L^1)BiCl$_2$] **(1).** b) Corresponding FOWA plot.

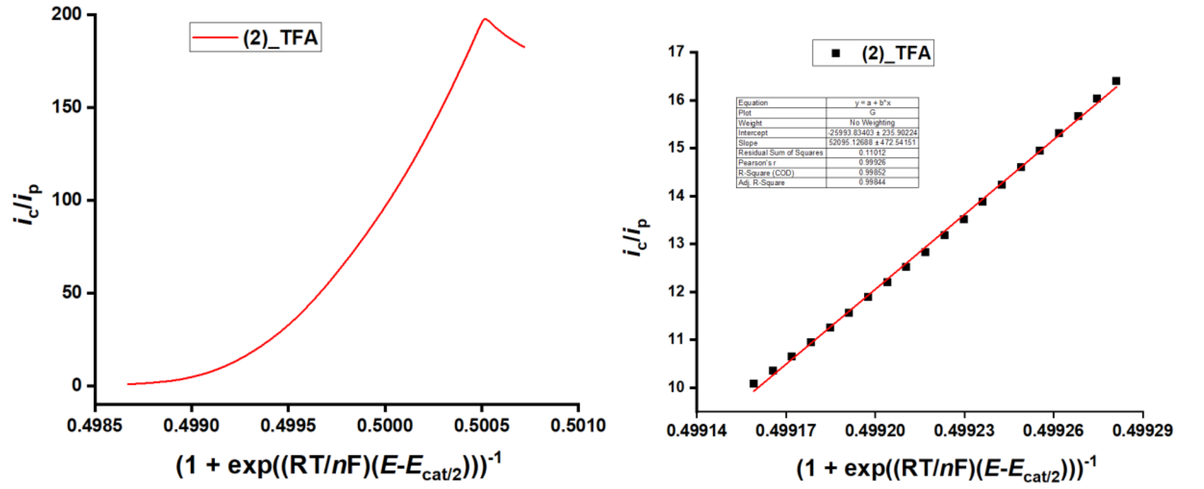

Figure S164. a) Plot for i_c/i_p vs (1 + exp((RT/nF)E-$E_{cat/2}$))$^{-1}$ from onset to peak position taken from CV for 0.1 M TFA (pK_a 12.65 in acetonitrile) in presence of 0.5 mM [(L^2)BiCl$_2$] **(2).** b) Corresponding FOWA plot.

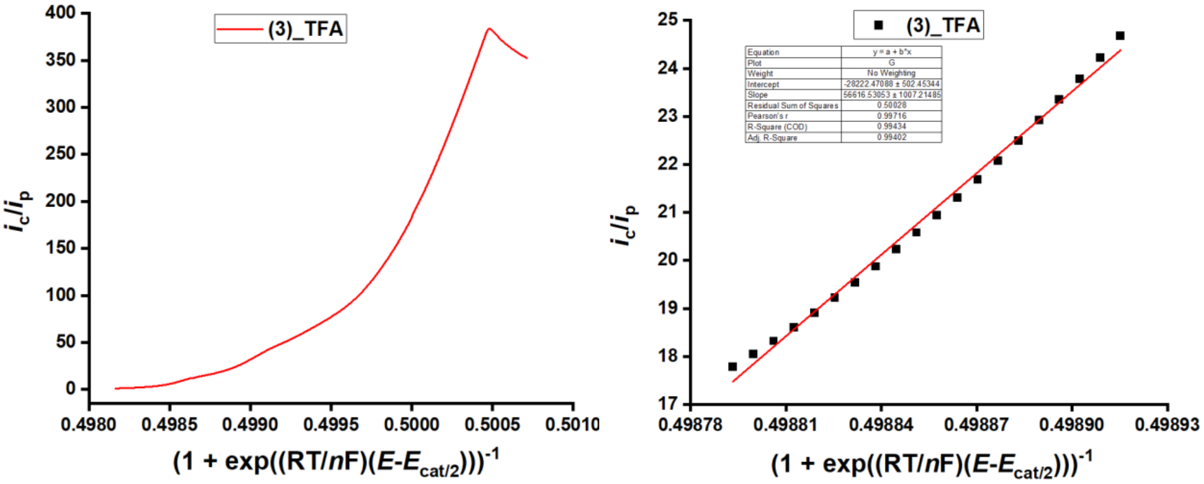

Figure S165. a) Plot for i_c/i_p vs $(1 + \exp((RT/nF)E\text{-}E_{cat/2}))^{-1}$ from onset to peak position taken from CV for 0.1 M TFA (pK_a 12.65 in acetonitrile) in presence of 0.5 mM [(L^3)BiCl$_2$] **(3).** b) Corresponding FOWA plot.

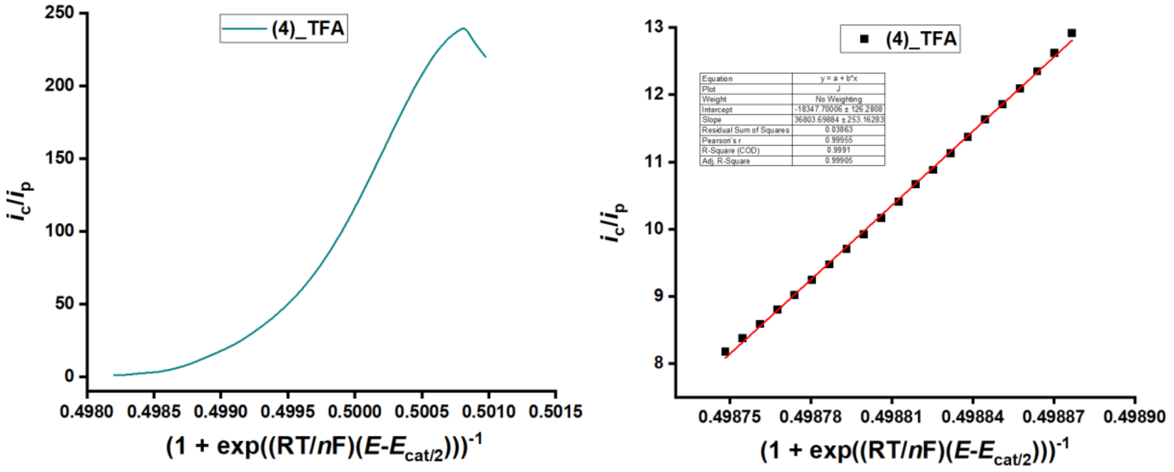

Figure S166. a) Plot for i_c/i_p vs $(1 + \exp((RT/nF)E\text{-}E_{cat/2}))^{-1}$ from onset to peak position taken from CV for 0.1 M TFA (pK_a 12.65 in acetonitrile) in presence of 0.5 mM [(L^4)BiCl$_2$] **(4).** b) Corresponding FOWA plot.

The calculated TOF_{max} from FOWA of 0.1 M TFA in presence of 0.5 mM catalyst were found to be 8.97 x 10^8 s^{-1}, 5.27 x 10^8 s^{-1}, 6.22 x 10^8 s^{-1}, and 2.63 x 10^8 s^{-1} for [(L^1)$BiCl_2$] **(1),** [(L^2)$BiCl_2$] **(2),** [(L^3)$BiCl_2$] **(3)**, [(L^4)$BiCl_2$] **(4)** respectively.

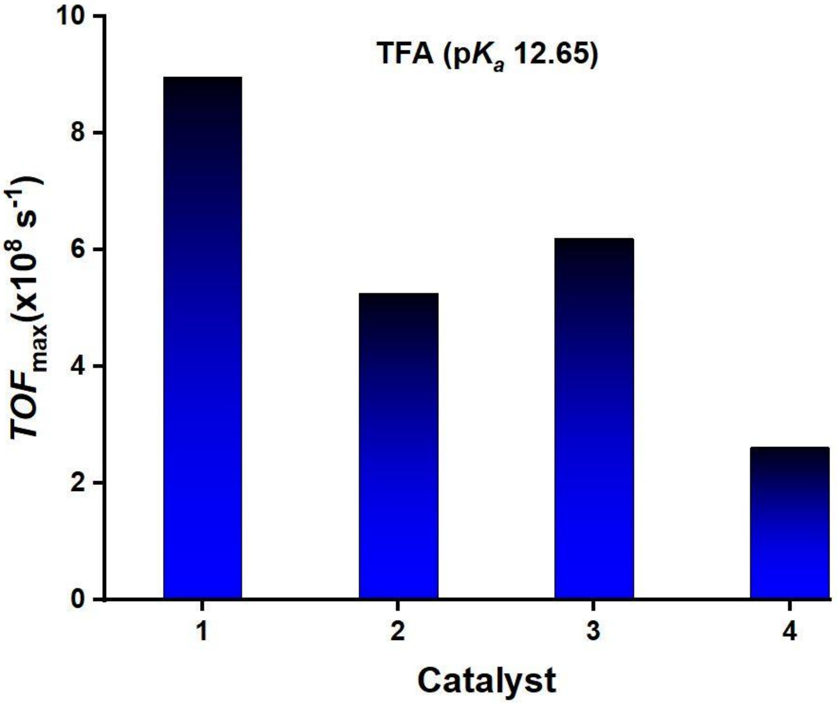

Figure S167. Plot of TOF_{max} values obtained from FOWA for 0.1 M TFA (pK_a 12.65 in acetonitrile) as an external proton source for different Bi(III) catalysts [(L^1)$BiCl_2$] **(1),** [(L^2)$BiCl_2$] **(2),** [(L^3)$BiCl_2$] **(3)**, [(L^4)$BiCl_2$] **(4)**.

FOWA for catalysts **([(L[1])BiCl$_2$] (1),** [(L[2])BiCl$_2$] **(2),** [(L[3])BiCl$_2$] **(3)**, [(L[4])BiCl$_2$] **(4))** were performed using 0.1 M 2-aminopyridinium BF_4^- (pK_a =14.47 in acetonitrile).

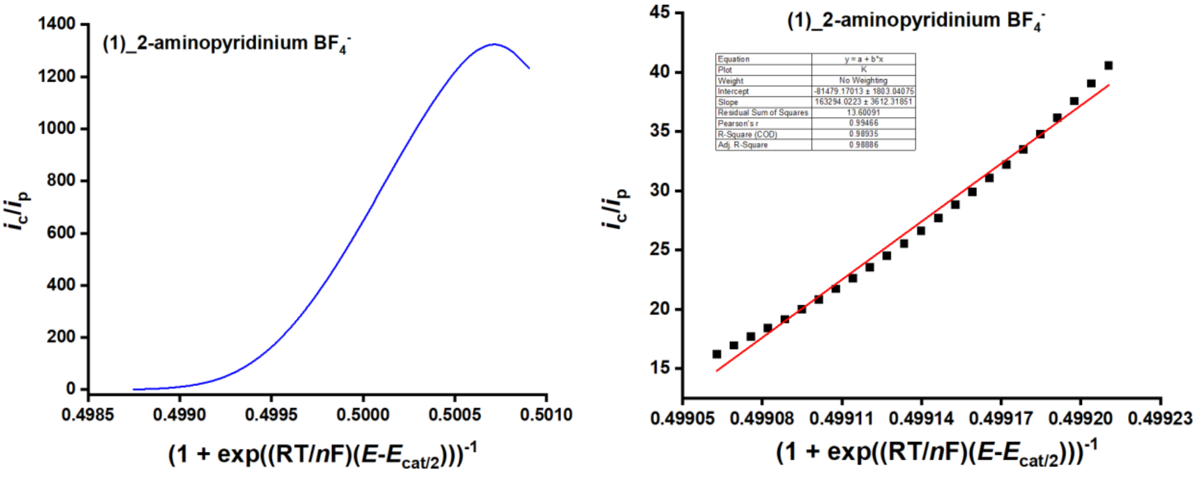

Figure S168. a) Plot for i_c/i_p vs $(1 + \exp((RT/nF)E\text{-}E_{cat/2}))^{-1}$ from onset to peak position taken from CV for 0.1 M 2-aminopyridinium tetrafluoroborate (pK_a 14.47 in acetonitrile) in presence of 0.5 mM [(L[1])BiCl$_2$] **(1)**. b) Corresponding FOWA plot.

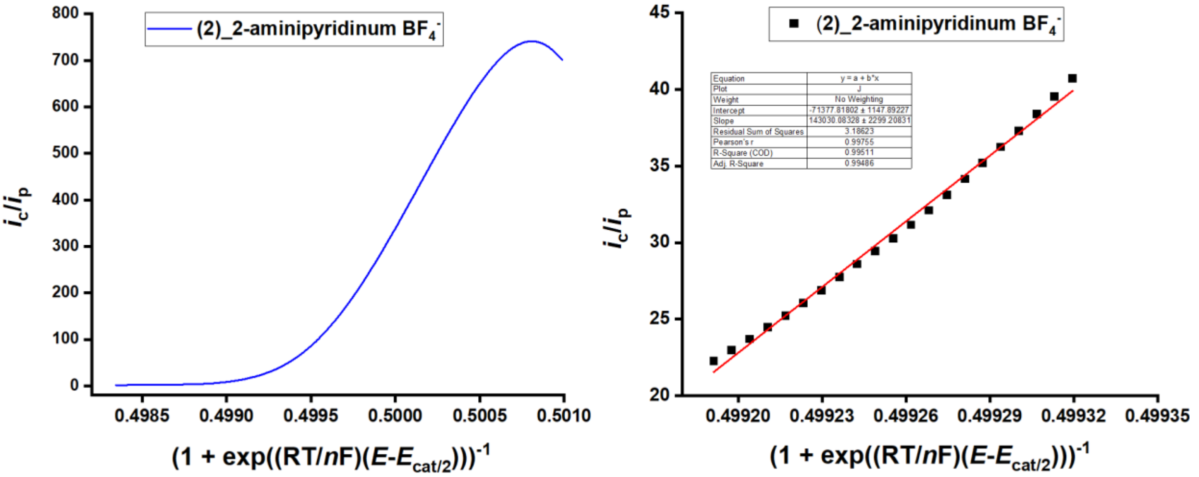

Figure S169. a) Plot for i_c/i_p vs $(1 + \exp((RT/nF)E\text{-}E_{cat/2}))^{-1}$ from onset to peak position taken from CV for 0.1 M 2-aminopyridinium tetrafluoroborate (pK_a 14.47 in acetonitrile) in presence of 0.5 mM [(L[2])BiCl$_2$] **(2)**. b) Corresponding FOWA plot.

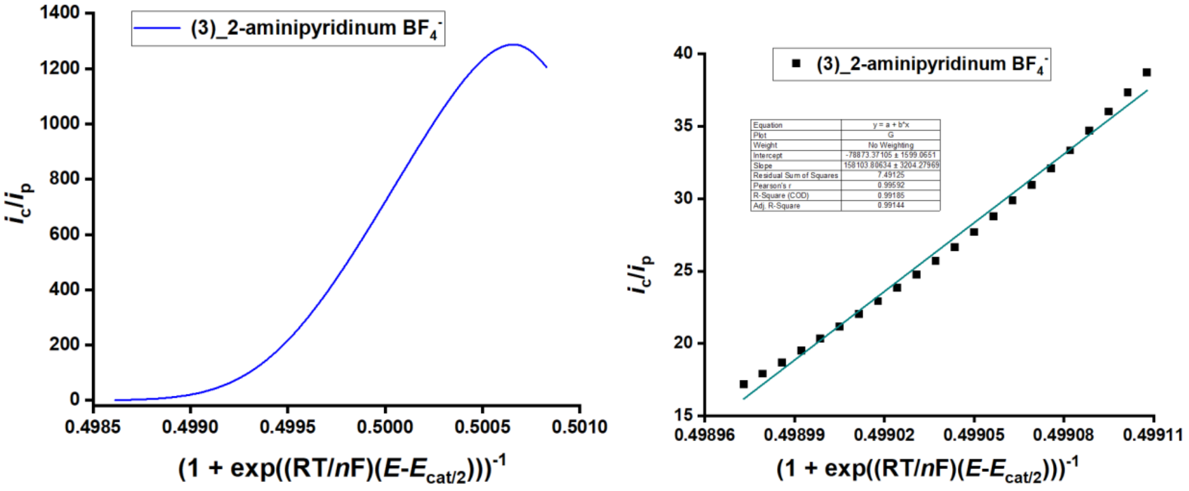

Figure S170. a) Plot for i_c/i_p vs $(1 + \exp((RT/nF)E\text{-}E_{cat/2}))^{-1}$ from onset to peak position taken from CV for 0.1 M 2-aminopyridinium tetrafluoroborate (pK_a 14.47 in acetonitrile) in presence of 0.5 mM [(L^3)BiCl$_2$] **(3)**. b) Corresponding FOWA plot.

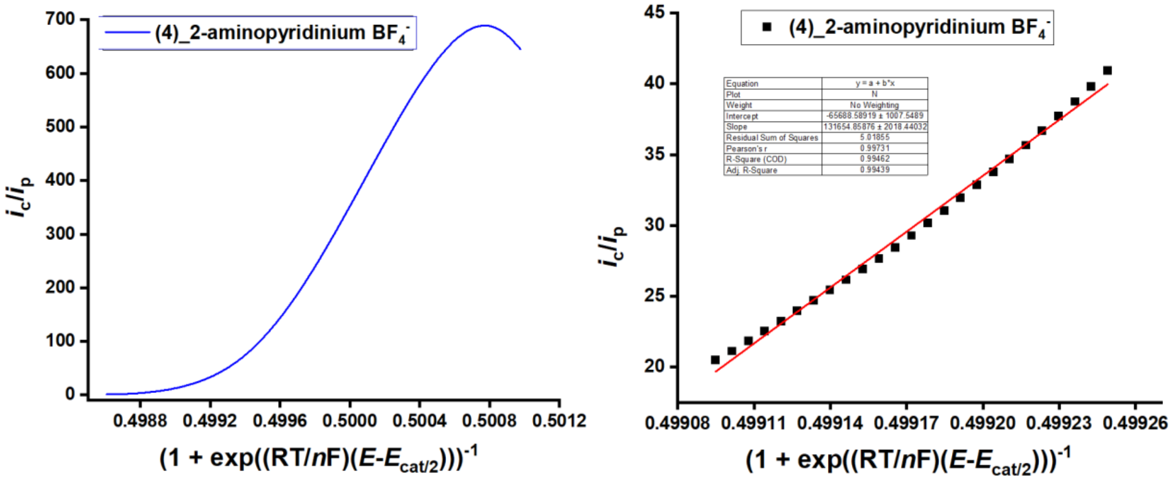

Figure S171. a) Plot for i_c/i_p vs $(1 + \exp((RT/nF)E\text{-}E_{cat/2}))^{-1}$ from onset to peak position taken from CV for 0.1 M 2-aminopyridinium tetrafluoroborate (pK_a 14.47 in acetonitrile) in presence of 0.5 mM [(L^4)BiCl$_2$] **(4)**. b) Corresponding FOWA plot.

The calculated TOF_{max} from FOWA of 0.1 M 2-aminopyridinium tetrafluoroborate in presence of 0.5 mM catalyst were found to be 5.18 x 10^9 s^{-1}, 3.97 x 10^9 s^{-1}, 4.85 x 10^9 s^{-1}, and 3.36 x 10^9 s^{-1} for [(L^1)$BiCl_2$] **(1),** [(L^2)$BiCl_2$] **(2),** [(L^3)$BiCl_2$] **(3)**, [(L^4)$BiCl_2$] **(4)** respectively.

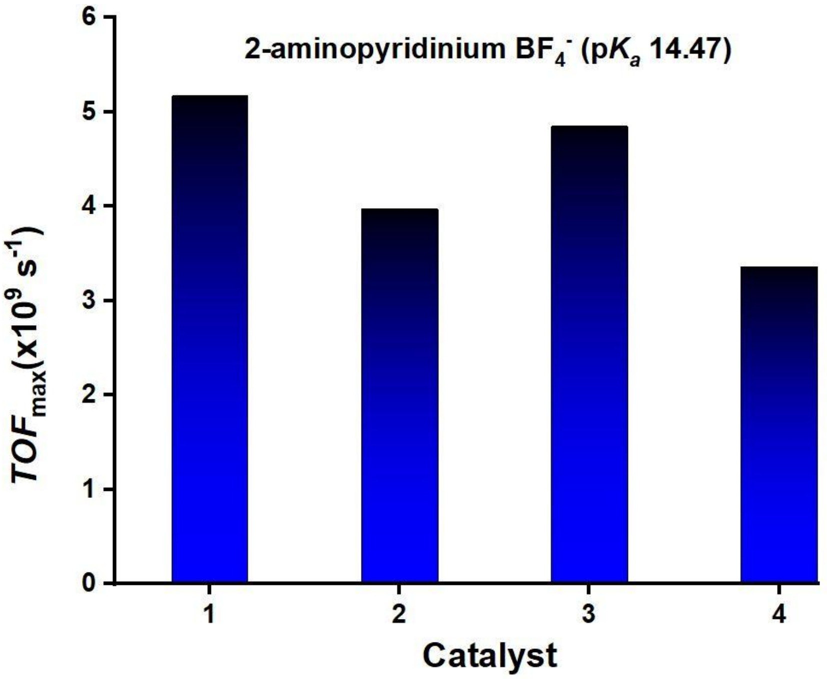

Figure 172. Plot of TOF_{max} values obtained from FOWA for 0.1 M 2-aminopyridinium tetrafluoroborate (pK_a 14.47 in acetonitrile) as an external proton source for different Bi(III) catalysts [(L^1)$BiCl_2$] **(1),** [(L^2)$BiCl_2$] **(2),** [(L^3)$BiCl_2$] **(3)**, [(L^4)$BiCl_2$] **(4)**.

FOWA for catalysts ([$(L^1)BiCl_2$] (1), [$(L^2)BiCl_2$] (2), [$(L^3)BiCl_2$] **(3)**, [$(L^4)BiCl_2$] **(4))** were performed using 0.1 M F_5-PhOH (pK_a =20.1 in acetonitrile).

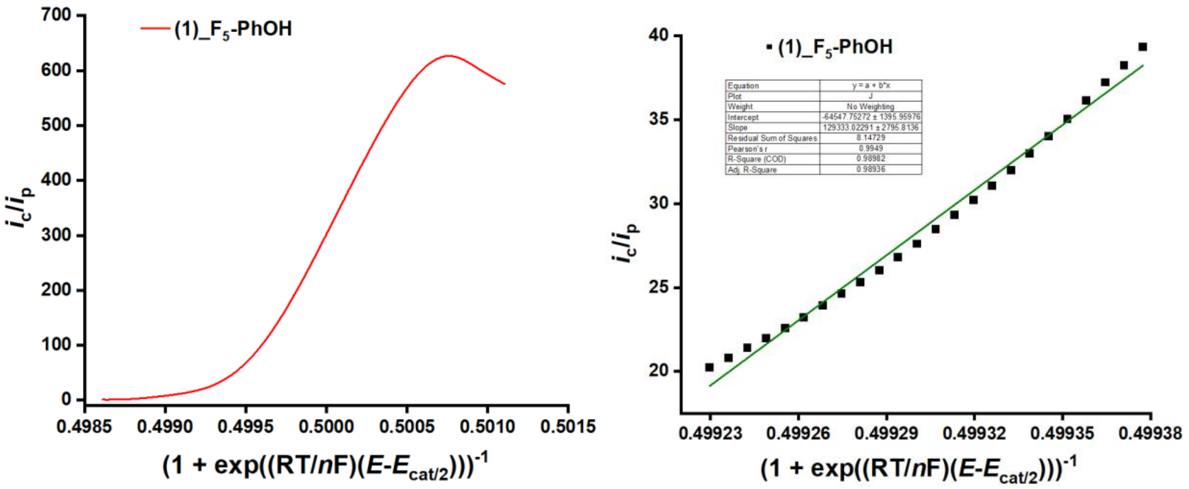

Figure S173. a) Plot for i_c/i_p vs $(1 + \exp((RT/nF)E\text{-}E_{cat/2}))^{-1}$ from onset to peak position taken from CV for 0.1 M F_5-PhOH (pK_a 20.10 in acetonitrile) in presence of 0.5 mM [$(L^1)BiCl_2$] **(1)**. b) Corresponding FOWA plot.

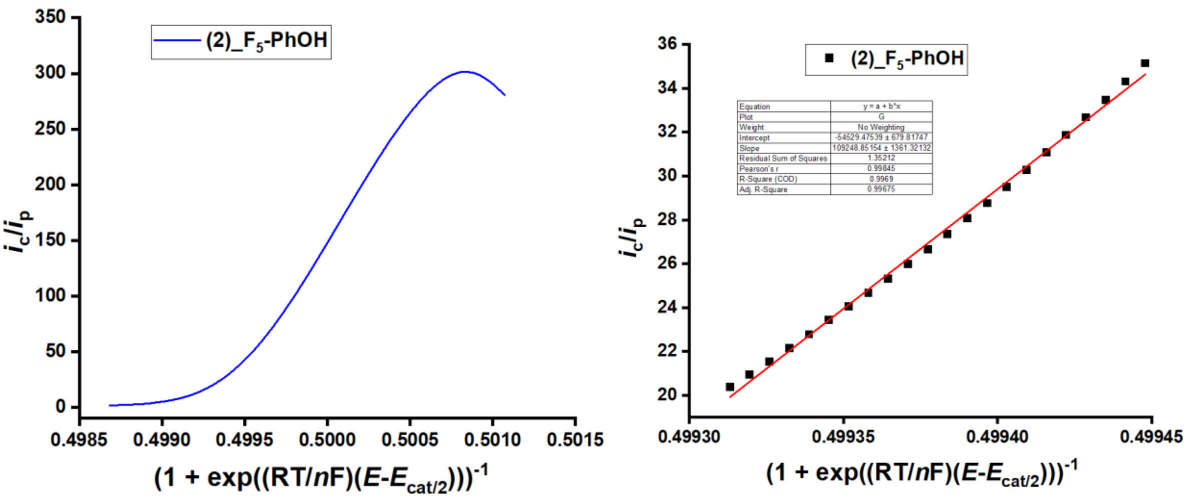

Figure S174. a) Plot for i_c/i_p vs $(1 + \exp((RT/nF)E\text{-}E_{cat/2}))^{-1}$ from onset to peak position taken from CV for 0.1 M F_5-PhOH (pK_a 20.10 in acetonitrile) in presence of 0.5 mM [$(L^2)BiCl_2$] **(2)**. b) Corresponding FOWA plot.

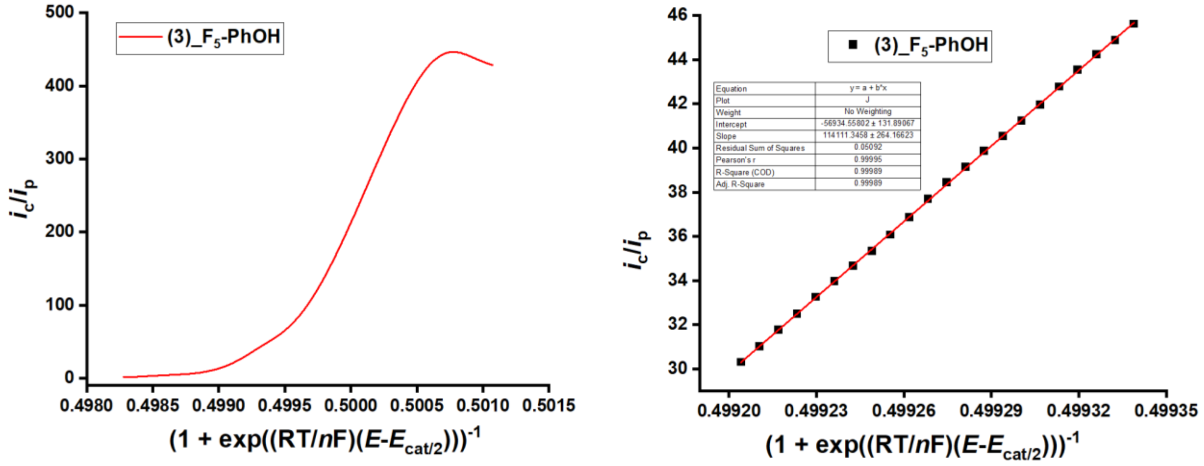

Figure S175. a) Plot for i_c/i_p vs (1 + exp((RT/*n*F)E-$E_{cat/2}$))$^{-1}$ from onset to peak position taken from CV for 0.1 M F_5-PhOH (pK_a 20.10 in acetonitrile) in presence of 0.5 mM [(L^3)BiCl_2] **(3)**. b) Corresponding FOWA plot.

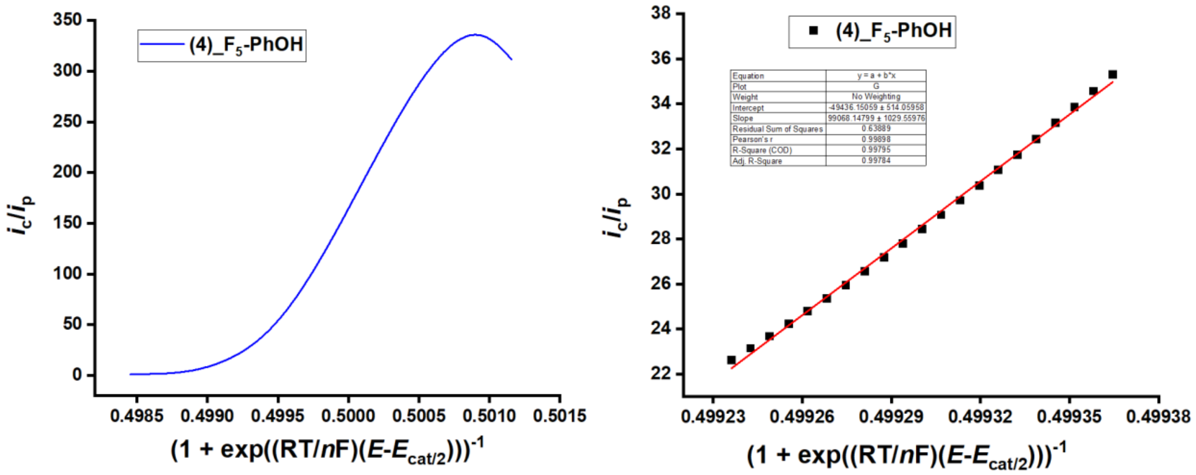

Figure S176. a) Plot for i_c/i_p vs (1 + exp((RT/*n*F)E-$E_{cat/2}$))$^{-1}$ from onset to peak position taken from CV for 0.1 M F_5-PhOH (pK_a 20.10 in acetonitrile) in presence of 0.5 mM [(L^4)BiCl_2] **(4)**. b) Corresponding FOWA plot.

The calculated TOF_{max} from FOWA of 0.1 M F_5-PhOH in presence of 0.5 mM catalyst were found to be 3.25 x 10^9 $s^{-1,}$ 2.32 x 10^9 s^{-1}, 2.53 x 10^9 s^{-1}, and 1.9 x 10^9 for [(L^1)$BiCl_2$] **(1),** [(L^2)$BiCl_2$] **(2),** [(L^3)$BiCl_2$] **(3)**, [(L^4)$BiCl_2$] **(4)** respectively.

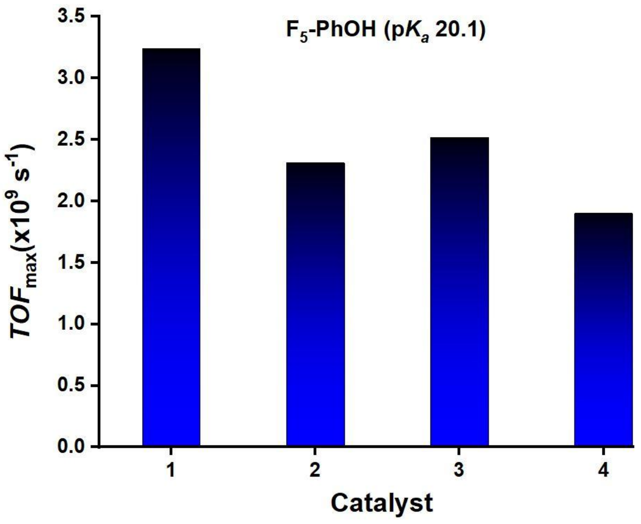

Figure S177. Plot of TOF_{max} values obtained from FOWA for 0.1 M F_5-PhOH (pK_a 20.10 in acetonitrile) as an external proton source for different Bi(III) catalysts [(L^1)$BiCl_2$] **(1),** [(L^2)$BiCl_2$] **(2),** [(L^3)$BiCl_2$] **(3)**, [(L^4)$BiCl_2$] **(4)**.

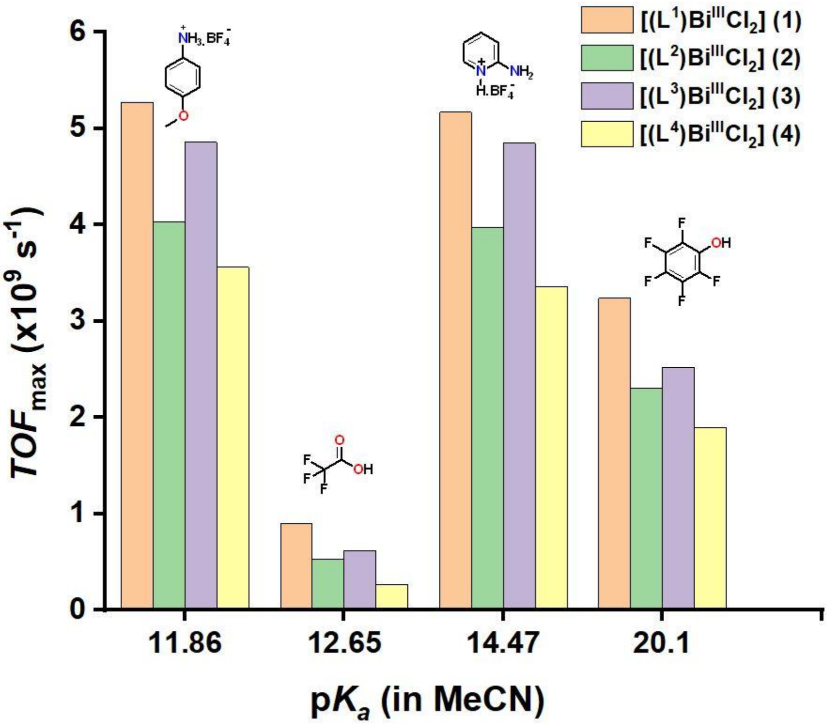

Figure S178. Complied plot of TOF_{max} values from FOWA using 0.1 M of four different acids as an external proton source in acetonitrile for catalysts [(L[1])BiCl[2]] **(1),** [(L[2])BiCl[2]] **(2),** [(L[3])BiCl[2]] **(3)**, [(L[4])BiCl[2]] **(4)**.

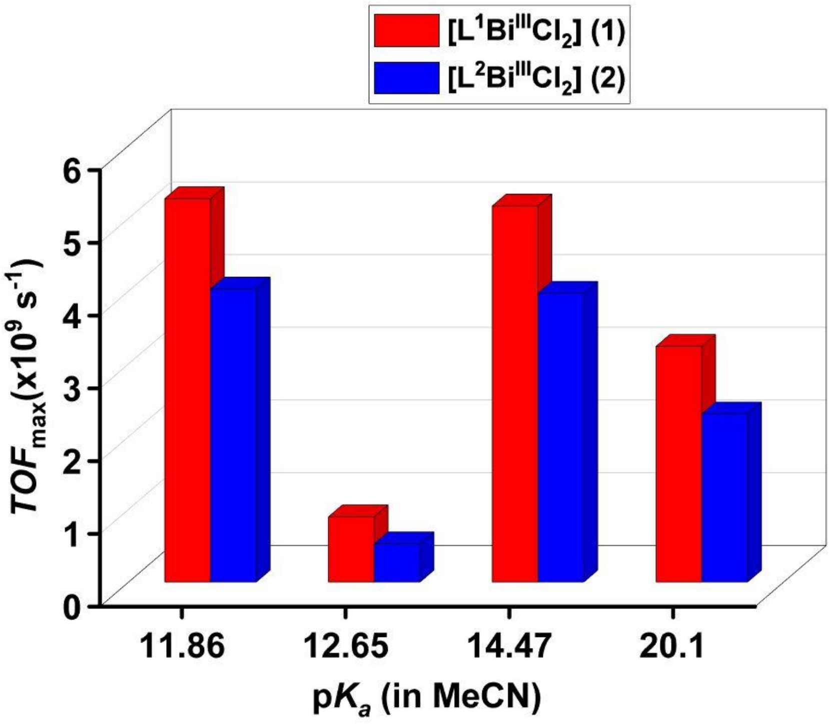

Figure S179. Complied plot of TOF_{max} (from FOWA) values for 0.1 M of four acid as an external proton source in acetonitrile for catalyst [(L[1])BiCl[2]] **(1)** and [(L[2])BiCl[2]] **(2)**.

Variable scan rate FOWA analysis

$i_{cat}/i_p = [2.24 \times n \sqrt{(RT / Fv)} \times \sqrt{(TOF_{max})}] / [1 + exp((nF/RT)(E – E_{cat/2}))]$

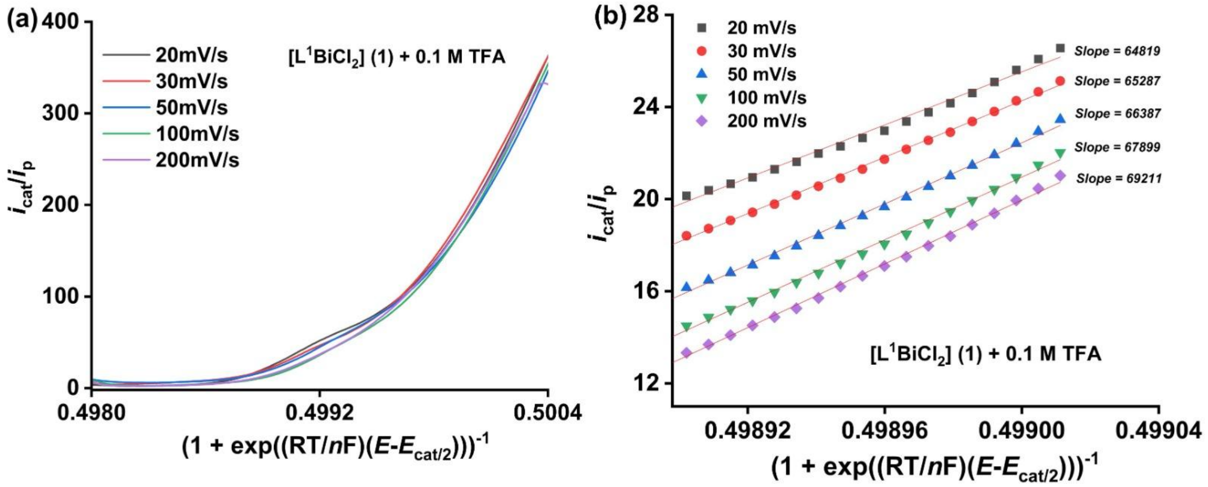

Figure S180. (a) FOWA plot of 0.5 mM catalyst **[(L¹)BiCl₂] (1)** varying scan rate with 0.1 M TFA in acetonitrile. (b) slope of the linear fit region from foot of the curve at different scan rates.

Table S21. FOWA slope with respective TOF_{max} on increasing scan rate with 0.5 mM catalyst **[(L¹)BiCl₂] (1)** at 0.1 M TFA.

Scan rate	Slope	TOF_{max} (s^{-1})
20 mV/s	64819.86	1.63×10^8
30 mV/s	65287.49	2.48×10^8
50 mV/s	66387.2	4.28×10^8
100 mV/s	67899.6	8.95×10^8
200 mV/s	69211.6	18.60×10^8

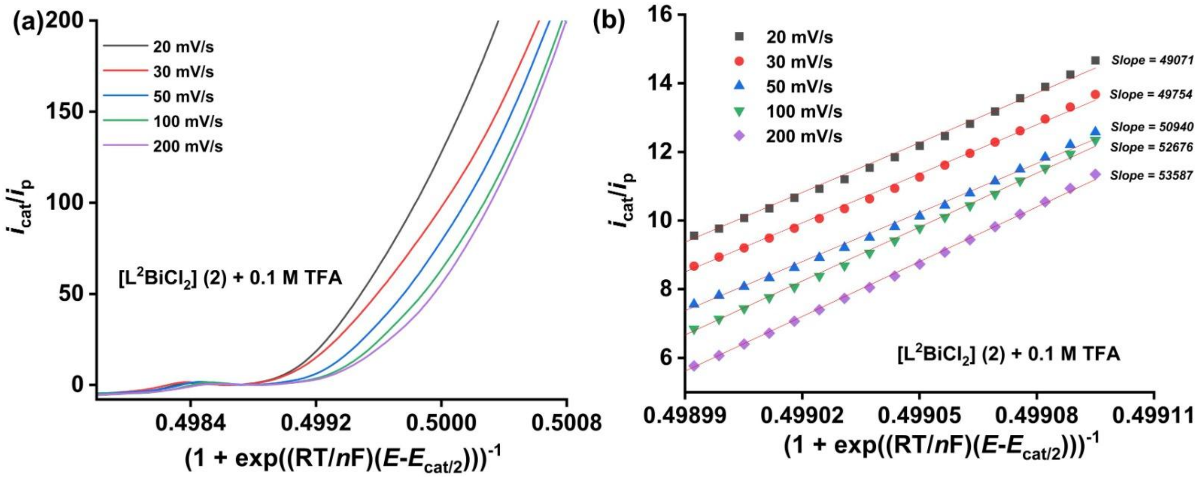

Figure S181. (a) FOWA plot of 0.5 mM catalyst **[(L²)BiCl₂] (2)** varying scan rate with 0.1 M TFA in acetonitrile. (b) slope of the linear fit region from foot of the curve at different scan rates.

Table S22. FOWA slope with respective TOF$_{max}$ on increasing scan rate with 0.5 mM catalyst **[(L^2)BiCl$_2$] (2)** at 0.1 M TFA.

**Scan rate**	**Slope**	**TOF$_{max}$ (s^{-1})**
20 mV/s	49071.01	0.93 x 10^8
30 mV/s	49754.71	1.44 x 10^8
50 mV/s	50940.17	2.52 x 10^8
100 mV/s	52376.75	5.32 x 10^8
200 mV/s	53587.35	11.1 x 10^8

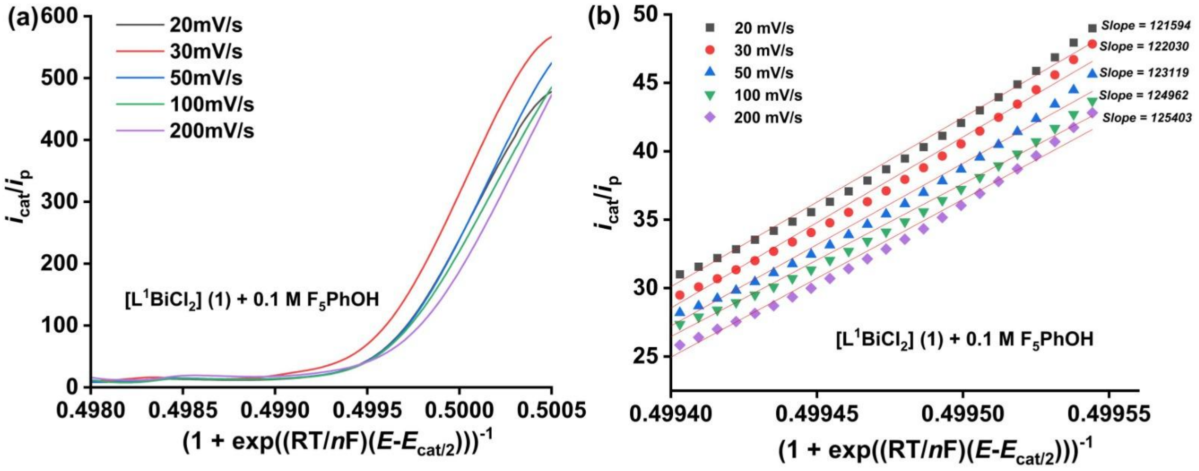

Figure S182. (a) FOWA plot of 0.5 mM catalyst **[(L¹)BiCl₂] (1)** varying scan rate with 0.1 M of F_5-PhOH in acetonitrile. (b) slope of the linear fit region from foot of the curve at different scan rates.

Table S23. FOWA slope with respective TOF_{max} on increasing scan rate with 0.5 mM catalyst **[(L¹)BiCl₂] (1)** at 0.1 M F_5-PhOH.

Scan rate	Slope	TOF_{max} (s^{-1})
20 mV/s	121594.6	0.57 x 10^9
30 mV/s	122030.5	0.86 x 10^9
50 mV/s	123119.1	1.40 x 10^9
100 mV/s	124962.2	3.00 x 10^9
200 mV/s	125403.3	6.10 x 10^9

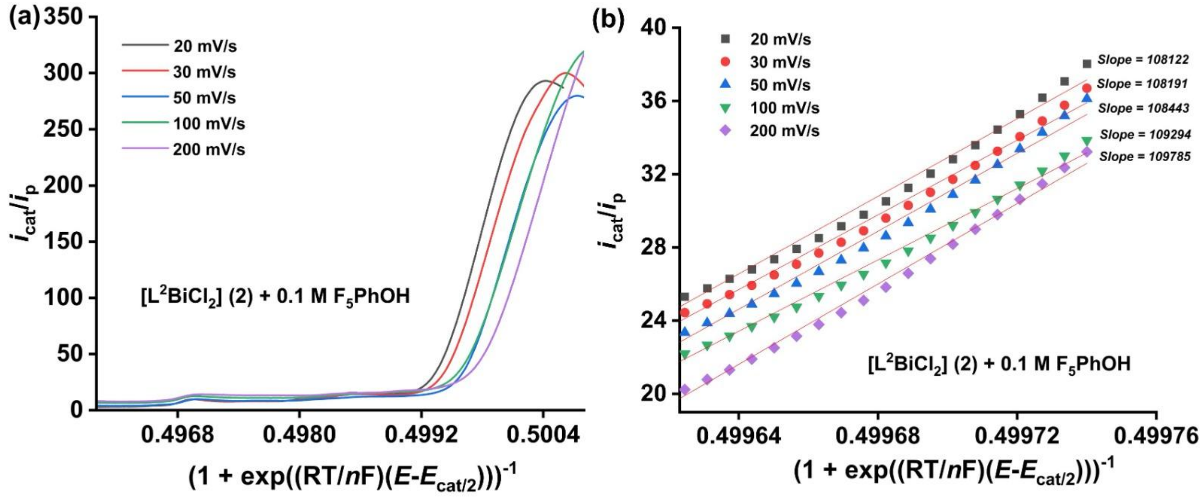

Figure S183. (a) FOWA plot of 0.5 mM catalyst **[(L²)BiCl₂] (2)** varying scan rate at 0.1 M of F_5-PhOH in acetonitrile. (b) slope of the linear fit region from foot of the curve at different scan rates.

Table S24. FOWA slope with respective TOF_{max} on increasing scan rate with 0.5 mM catalyst [**(L²)BiCl₂] (2)** at 0.1 M F_5-PhOH.

Scan rate	Slope	TOF_{max} (s^{-1})
20 mV/s	108122	0.45×10^9
30 mV/s	108191	0.68×10^9
50 mV/s	108443	1.1×10^9
100 mV/s	109294	2.3×10^9
200 mV/s	109786	4.6×10^9

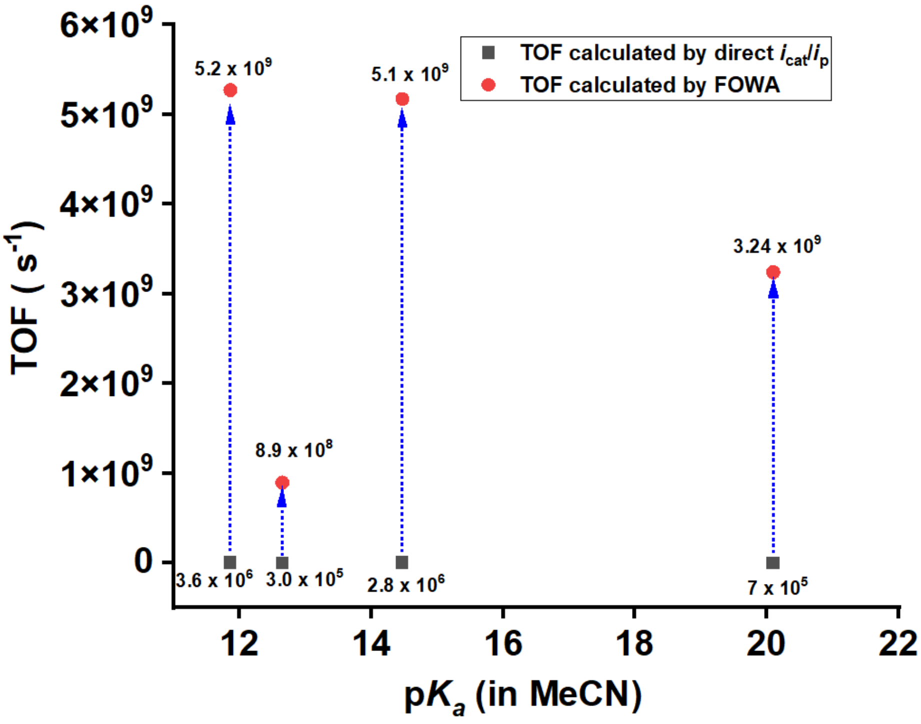

Figure S184. Complied plot of TOF values for 0.1 M of four acid as an external proton source calculated by FOWA and by direct method of i_{cat}/i_p for catalyst $[(L^1)BiCl_2]$ **(1)** and $[(L^2)BiCl_2]$ **(2).**

Electrochemical experiment for Kinetic Isotope Effect (KIE) (XIII)

1. **Proton reduction by catalyst [(L[1])BiCl$_2$] (1) with TFA and TFA-d1**

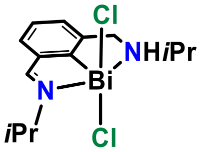

[(L[1])Bi^{III}Cl$_2$] (1)

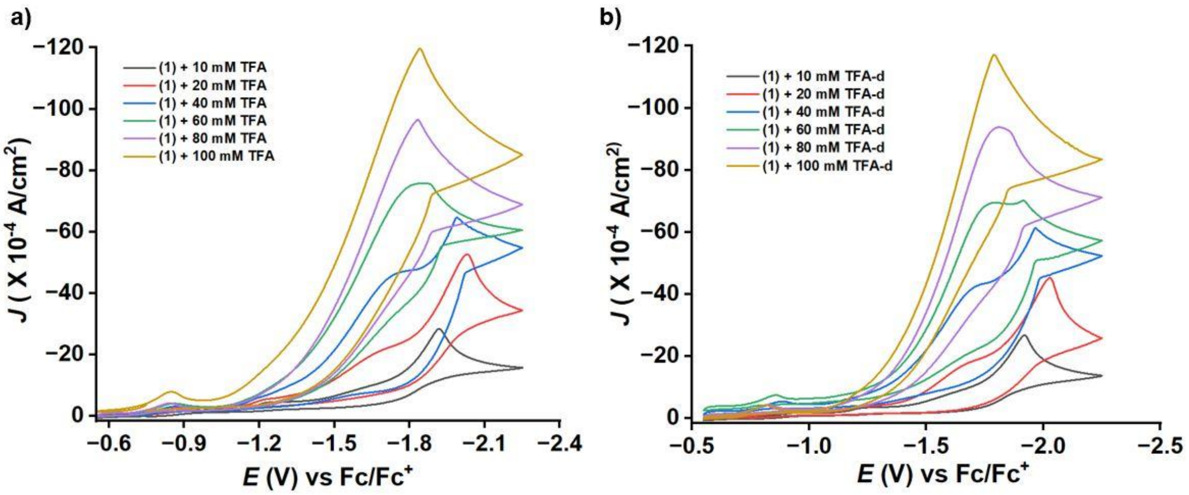

Figure S185 a) Cyclic Voltammogram of 0.5 mM [(L[1])BiCl$_2$] **(1)** with varying concentration of TFA **b)** Cyclic Voltammogram of 0.5 mM [(L[1])BiCl$_2$] **(1)** with varying concentration of TFA-d1 Condition: 0.1 M TBAPF$_6$ as a supporting electrolyte in acetonitrile; Scan rate = 100 mV/s. Working electrode glassy carbon (3 mm diameter), counter electrode Pt wire, and reference is Ag/AgCl. Data referenced to Fc/Fc$^+$ couple.

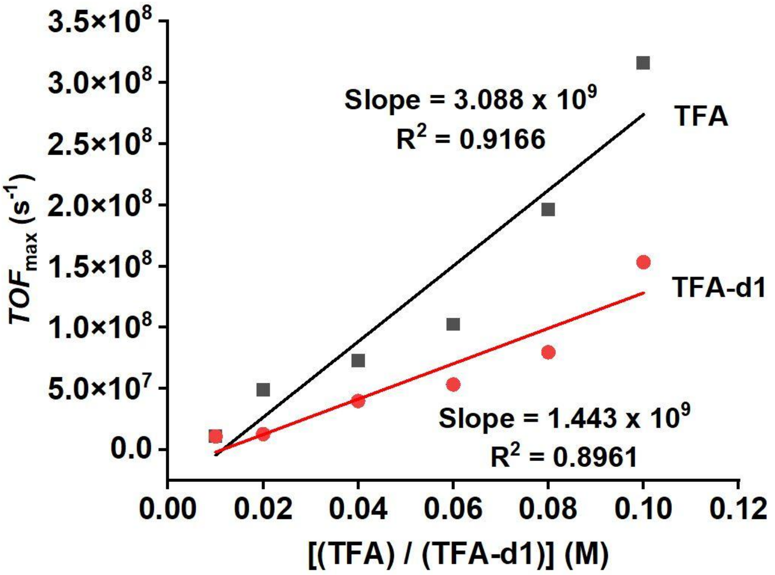

Figure S186. Plot for TOF_{max} (obtained from FOWA) vs concentration of TFA (black line) and TFA-d1 (red line) for catalyst $[(L^1)BiCl_2]$ **(1)**. Kinetic Isotope Effect, KIE = k_H/k_D = 3.08/1.44 = 2.1 and slope of black line = k_2 = 3.088 x 10^9 (M^{-1} s^{-1}) for TFA with $[(L^1)BiCl_2]$ **(1)**.

2. **Proton reduction by Catalyst $[(L^2)BiCl_2]$ (2)**, **with TFA and TFA-d1**

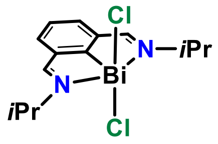

$[(L^2)Bi^{III}Cl_2]$ (2)

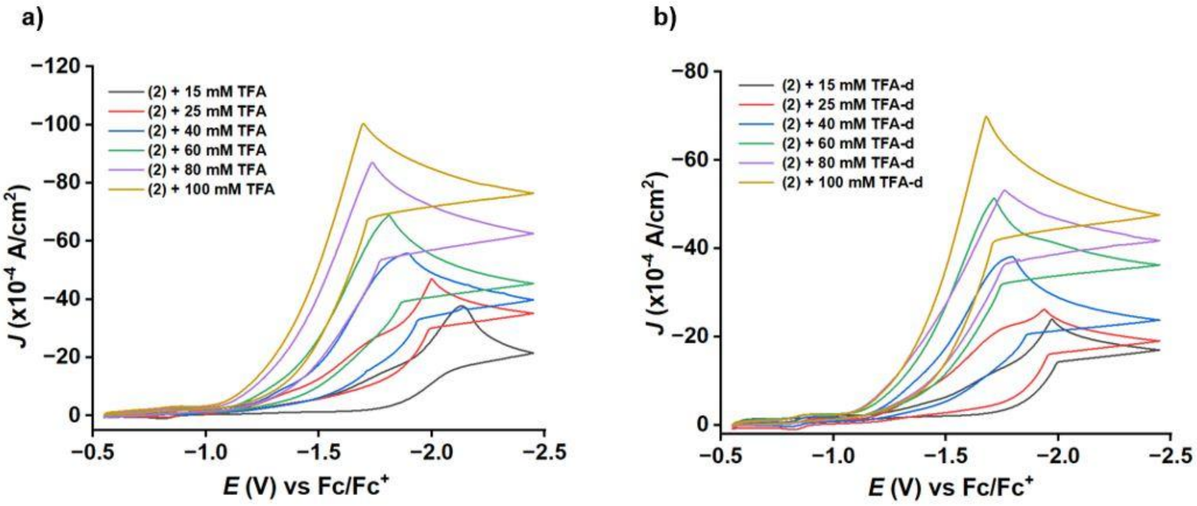

Figure S187. Cyclic Voltammogram of 0.5 mM $[(L^2)BiCl_2]$ **(2)** with varying concentration of TFA **b)** Cyclic Voltammogram of 0.5 mM $[(L^2)BiCl_2]$ **(2)** with varying concentration of TFA-d1 Condition: 0.1 M $TBAPF_6$ as a supporting electrolyte in acetonitrile; Scan rate = 100 mV/s.

Working electrode glassy carbon (3 mm diameter), counter electrode Pt wire, and reference is Ag/AgCl. Data referenced to Fc/Fc+ couple.

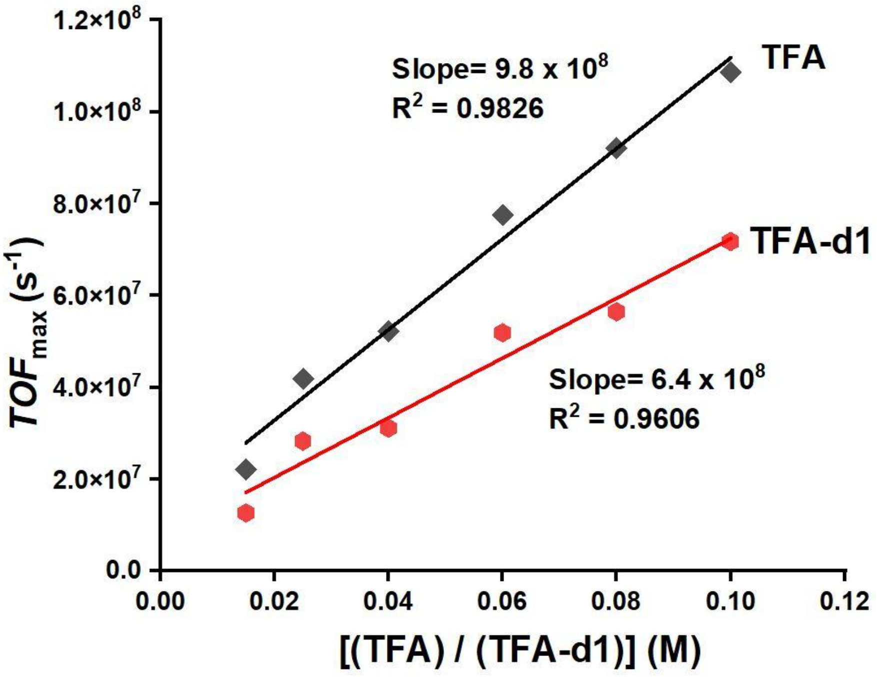

Figure S188. Plot for TOF_{max} (obtained from FOWA) vs concentration of TFA (black line) and TFA-d1 (red line) for catalyst $[(L^2)BiCl_2]$ **(2)**. Kinetic Isotope Effect, KIE = $k_H/k_D = 9.8 \times 10^8/6.4 \times 10^8 = 1.51$ and slope of black line = $k_2 = 9.8 \times 10^8$ (M^{-1} s^{-1}) for TFA with catalyst $[(L^2)BiCl_2]$ **(2)**.

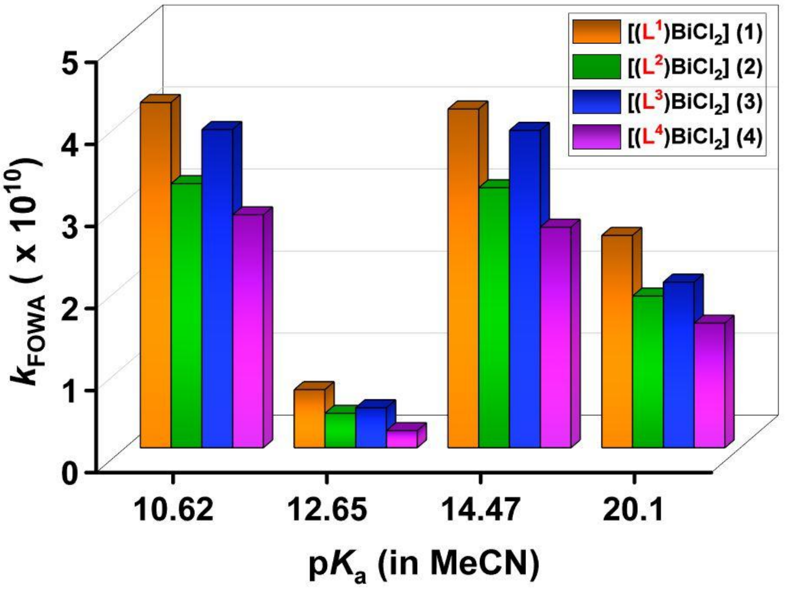

Figure S189. Plot of k_{FOWA} vs pK_a for all catalysts [(L^1)$BiCl_2$] **(1),** [(L^2)$BiCl_2$] **(2),** [(L^3)$BiCl_2$] **(3)**, [(L^4)$BiCl_2$] **(4)** at maximum conc. (0.1 M) of acid sources used in acetonitrile.

Calculation for first protonation step k_1 via foot of the wave analysis[13]

To perform foot-of-the-wave analysis on the catalytic voltammograms, the potential axis is converted to $\exp[-F/RT(E - E_{cat/2})]$ plotted as X axis. In the Y axis is plotted the current as the ratio of the catalytic current (i_c) divided by the non-catalytic current (i_p) of the Bi(III)/(I) redox couple. The equation derived for FOWA of an ECEC mechanism (Eq. S1) is divided by the Randles–Sevcik equation (Eq. S2) to obtain the i_c/i_p relationship (Eq. S3). The linear portion at the foot of the wave can be fit with a straight line to obtain the slope (m), which is represented by Eq. S4. Solving Eq. S4 for k_{FOWA} yields Eq. S5, which is used to obtain the observed rate from the FOWA analysis.

$i_c = 2FAC_{cat} \sqrt{(k_{FOWA} D_{cat})} \exp[-F/RT (E - E_{cat/2})]$ Eq. S1

$i_p = 0.4463\ nFAC_{cat} (nFvD_{cat}/RT)^{1/2}$ Eq. S2

$i_c/i_p = (4.48/n) \sqrt{(RT\, k_{FOWA} /nFv)} \exp[-F/RT (E - E_{cat/2})]$ Eq. S3

Inserting numerical values for constants;

R = 8.3145 J mol^{-1} K^{-1}, T = 298 K, F = 96,485 C mol^{-1}

y = mx

$i_c/i_p = (0.7179/n) \sqrt{(k_{FOWA} /nv)} \exp [-38.9(E - E_{cat/2})]$

The slope of the line (m) obtained from FOWA for an ECEC mechanism is:

$m = (0.7179/n) \sqrt{(k_{FOWA} /nv)}$ Eq. S4

Solving for k_{FOWA}:

$k_{FOWA} = nv\, (mn/0.7179)^2$ Eq. S5

In this work, n = 2 corresponds to the Bi(III)/(I) redox couple. v = 0.1 V/s for all FOWA datasets and thus,

$k_{FOWA} = 1.55m^2$ Eq. S6

k_{FOWA} at 0.5 mM concentration of the respective catalysts [(L^1)$BiCl_2$] **(1)** and [(L^2)$BiCl_2$] **(2)** were calculated using equation Eq S6. The k_{FOWA} values thus obtained was plotted against varying

concentration of TFA (pK_a 12.65 in acetonitrile) to obtain a straight line (Figure S190a and S190b). The slope of the linear fit gave k_1.[13]

Electrochemical working condition: 0.1 M $TBAPF_6$ as a supporting electrolyte in acetonitrile; scan rate = 100 mV/s, Working electrode glassy carbon (3 mm diameter), counter electrode Pt wire, and reference is Ag/AgCl. Data referenced to Fc/Fc^+ couple.

Plot of k_{FOWA} vs conc. of TFA for [(L^1)BiCl$_2$] (1)

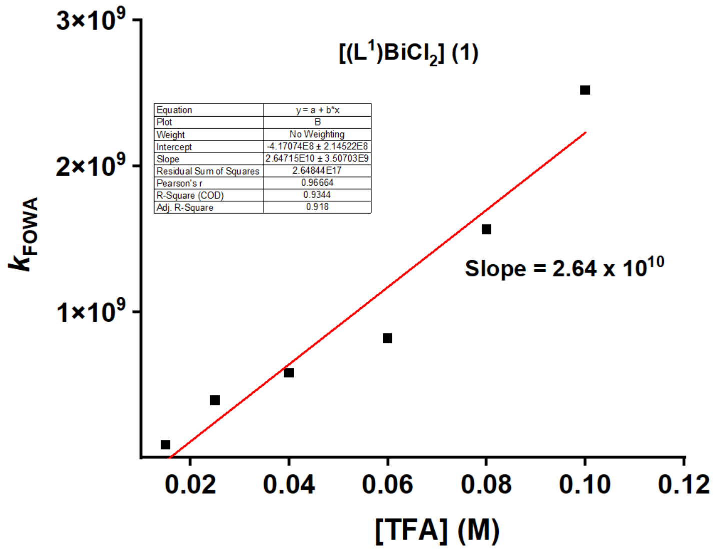

Figure S190a. Plot of k_{FOWA} vs concentration of TFA for **[(L^1)BiCl$_2$] (1).** k_{FOWA} vs various TFA concentration gives k_1 = 2.64 x 10^{10} (M^{-1} s^{-1})

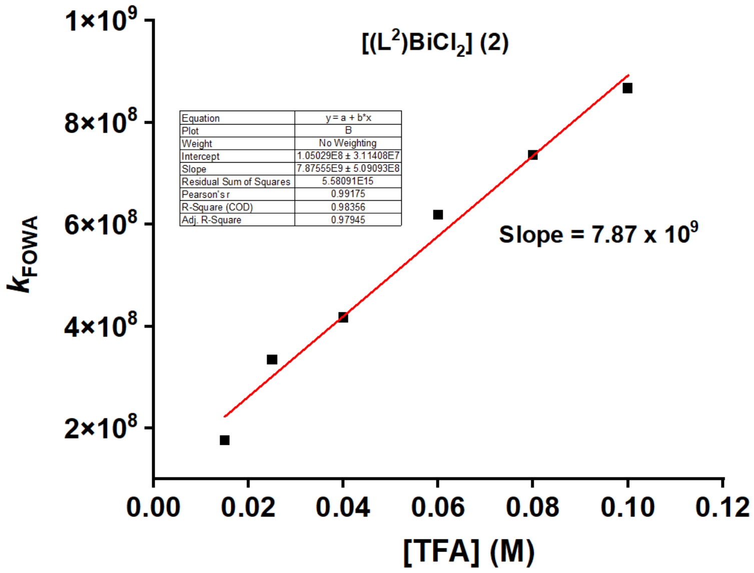

Figure S190b. Plot of k_{FOWA} vs concentration of TFA for **[(L^2)BiCl$_2$] (2)** k_{FOWA} vs various TFA concentration gives k_1 = 7.87 x 10^9 (M^{-1} s^{-1})

Calculation for second protonation step k_2 via foot of the wave analysis

For an ECEC mechanism, when $k_1[H^+] >> k_2[H^+]$ [11,14-16]

Using TOF_{max} obtained from FOWA calculation

$k_{obs} = TOF_{max} = k_2[H^+]$ [11,14-16]

Plot of TOF_{max} obtained from FOWA vs varied concentration of TFA (pK_a 12.65 in acetonitrile) using 0.5 mM catalyst gave a linear fit having slope = k_2 = 3.08 x 10^9 M^{-1} s^{-1} for **[(L^1)$BiCl_2$] (1)** (Figure S191a) and slope = k_2 = 9.8 x 10^8 M^{-1} s^{-1} for **[(L^2)$BiCl_2$] (2)** (Figure S191b).

Electrochemical working condition: 0.1 M $TBAPF_6$ as a supporting electrolyte in acetonitrile; scan rate = 100 mV/s, Working electrode glassy carbon (3 mm diameter), counter electrode Pt wire, and reference is Ag/AgCl. Data referenced to Fc/Fc^+ couple.

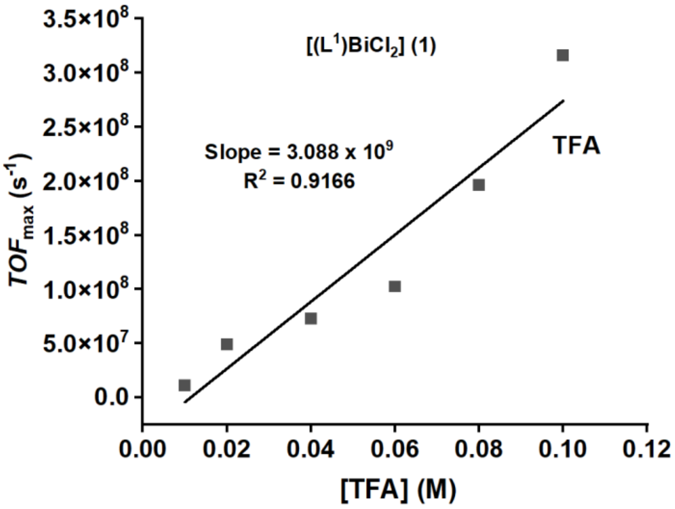

Figure S191a. Plot of TOF_{max} vs concentration of TFA for **[(L^1)$BiCl_2$] (1).** The slope of the linear fit gave k_2 = 3.08 x 10^9 (M^{-1} s^{-1})

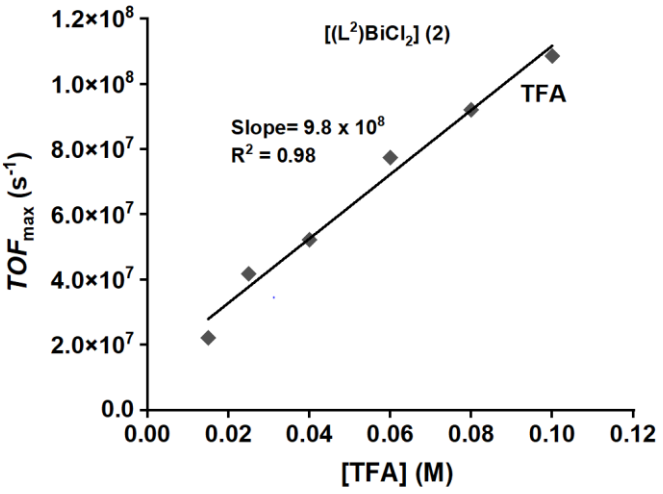

Figure S191b. Plot of TOF_{max} vs concentration of TFA for **$[(L^2)BiCl_2]$ (2).** The slope of the linear fit gave $k_2 = 9.8 \times 10^8$ (M^{-1} s^{-1})

For better clarity, k_2 was also estimated using TOF values derived from catalytic plateau currents, giving significantly smaller values of 5.0×10^6 M^{-1} s^{-1} for (**1**) and 1.1×10^6 M^{-1} s^{-1} for (**2**) as shown below (Figure S191c). However, these values should be interpreted with caution because k_1 and k_2 would then originate from kinetically distinct regimes of the voltammogram (FOWA versus plateau-current analysis), precluding a rigorous direct comparison within a single mechanistic framework. Moreover, the plateau-current region may be influenced by secondary phenomena such as substrate depletion, catalyst deactivation, uncompensated resistance effects, or product inhibition, rendering the physical significance of the derived k_2 values less definitive. These values are therefore presented only as supplementary information and are not used in assigning the rate-determining step. Furthermore, extensive electrokinetic analyses presented in this study together with catalyst stability investigations support the conclusion that the catalytic measurements were performed under kinetic control and are not significantly affected by such secondary processes.

A point to note, irrespective of the method employed, k_2 remains substantially smaller than k_1, consistently supporting the assignment of the second protonation step as the rate-determining step in the proposed HER pathway.

Calculation for second protonation step k_2 via catalytic plateau current[13]

Catalytic current: $i_{\mathrm{cat}} = n_{\mathrm{cat}}\, FADC_{\mathrm{cat}}\, (k_{\mathrm{obs}}/D)^{1/2}$

non-catalytic current (Randles-Sevcik equation):

$i_p = 0.4463\, npFAC_{\mathrm{cat}}\, (npFDv/RT)^{1/2}$

Dividing:

$i_{cat}/i_p = (n_{cat}/0.4463np)[k_{obs}RT/(npFv)]^{1/2}$

Rearranging:

$(0.4463np/n_{cat})(i_{cat}/i_p) = [k_{obs}RT/(npFv)]^{1/2}$

Squaring both sides and putting k_{obs} = TOF

$(i_{cat}/i_p)^2 = [n_{cat}^2/(0.4463^2\ np^2)]\times[(\text{TOF}\times RT)/(npFv)]$

$= [n_{cat}^2\ RT\ \text{TOF}]/[(0.4463)^2\ np^3Fv]$

Rearranging the equation

$\text{TOF} = [(0.4463)^2\ np^3Fv/(ncat^2RT)]\times(i_{cat}/i_p)^2$

or $\text{TOF} = (Fvnp^3\ /\ RT)\times[(0.4463/n_{cat})\times(i_{cat}/i_p)]^2$
Therefore,

$\text{TOF} = k_{obs} = (npFv/RT)[(0.4463np/n_{cat})(i_{cat}/i\text{p})]^2$

For the ECEC mechanism, when $k_1[H^+] >> k_2[H^+]$[11]

$k_{obs} = (\text{TOF}) = k_2[H^+]$

Electrochemical working condition: 0.1 M $TBAPF_6$ as a supporting electrolyte in acetonitrile; scan rate = 100 mV/s, Working electrode glassy carbon (3 mm diameter), counter electrode Pt wire, and reference is Ag/AgCl. Data referenced to Fc/Fc^+ couple.

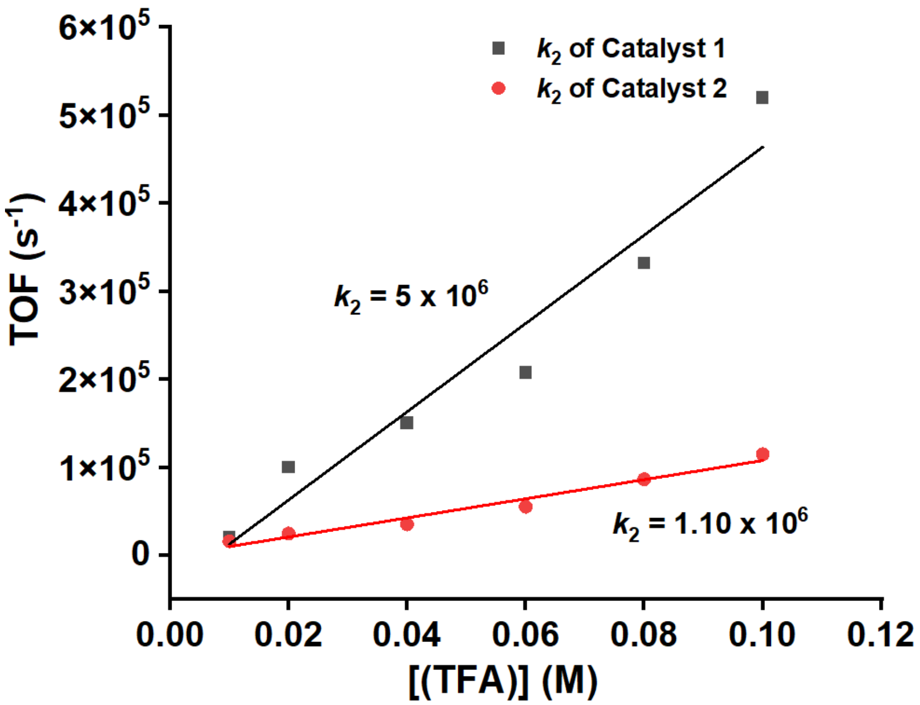

Figure S191c. Plot of TOF obtained from catalytic plateau current vs varied concentration of TFA (pK_a 12.65 in acetonitrile) using 0.5 mM catalyst gave a linear fit having slope = k_2 = 5 x 10^6 M^{-1} s^{-1} for **[(L[1])BiCl$_2$] (1)** (black) and slope = k_2 = 1.10 x $10^{86}$$M^{-1}$ s^{-1} for **[(L[2])BiCl$_2$] (2)** (red).

Tafel Analysis (XIV)

Tafel slope is the number of mV/s required to increase the current by a factor of a factor of 10, and thus it is reported in mV/dec Therefore, a low Tafel slope value is an indication of an active catalyst, as a smaller overpotential is required to reach a higher current density. Under certain conditions, kinetic information, such as the rate-determining step, can be extracted from the value of the Tafel slope.[17]

The Tafel equation describes the current's exponential dependence on overpotential in these regions:

$$\eta = a + b \log(i)$$

Where η is the overpotential, i is the current density, a is the Tafel intercept, and b is the Tafel slope.

Calculation of Tafel slope for catalyst [(L[1])BiCl$_2$] (1) with 2-aminopyridinium BF_4^-

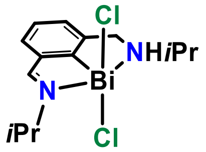

[(L^1)Bi^{III}Cl$_2$] (1)

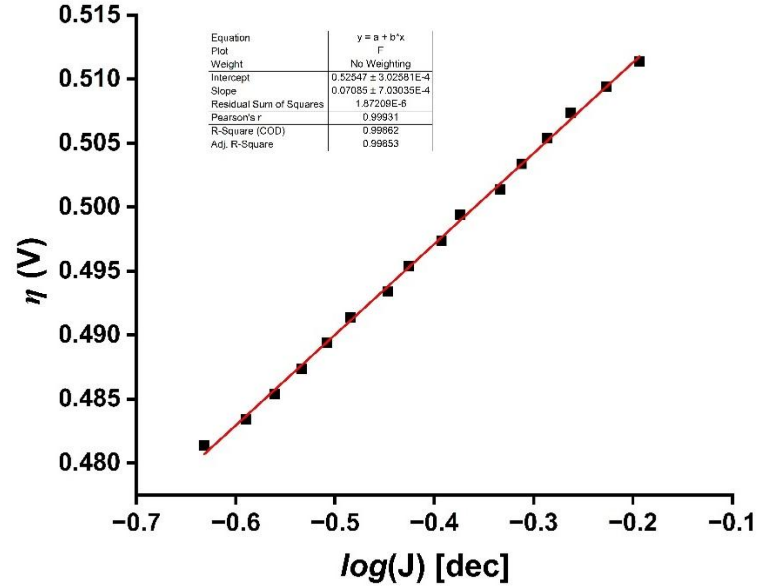

Figure S192. Tafel Slope of catalyst [(L^1)BiCl$_2$] **(1)** with 2-aminopyridinium BF_4^- (pK_a 14.47 in acetonitrile) is 70.85 mV/dec.

Calculation of Tafel slope for catalyst [(L^1)BiCl$_2$] (1) with Anilinium BF_4^-

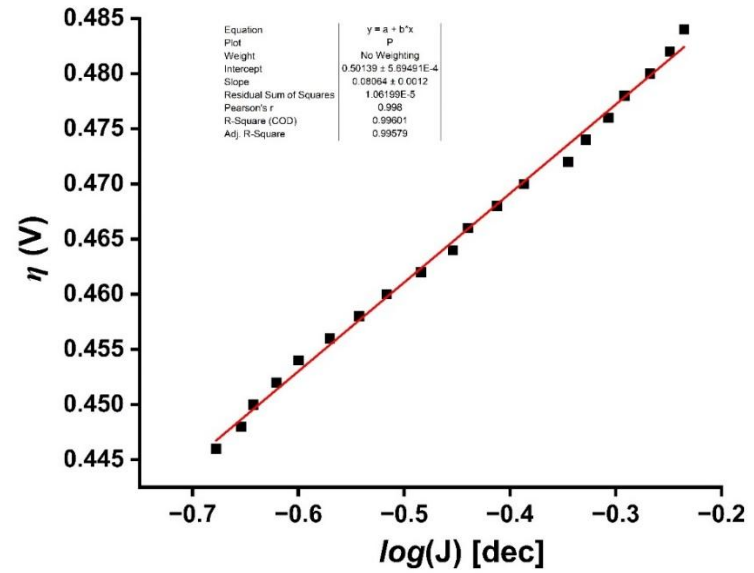

Figure S193. Tafel Slope of catalyst [(L^1)$BiCl_2$] **(1)** with Anilinium tetrafluoroborate (pK_a 10.62 in acetonitrile) is 80.64 mV/dec.

Calculation of Tafel slope for catalyst [(L^2)$BiCl_2$] (2) with 2-aminopyridinium BF_4^-

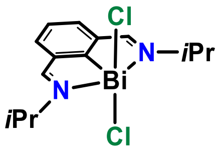

[(L^2)$Bi^{III}Cl_2$] (2)

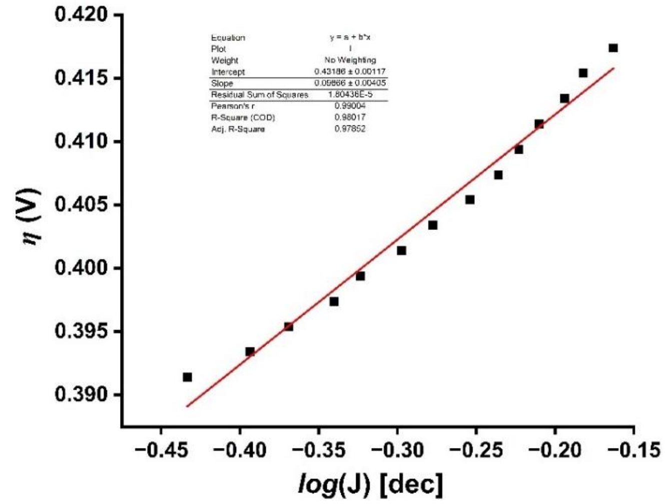

Figure S194. Tafel Slope of catalyst [L^2)$BiCl_2$] **(2)** with 2-aminopyridinium BF_4^- (pK_a 14.47 in acetonitrile) is 98.66 mV/dec.

Calculation of Tafel slope for catalyst [(L^2)$BiCl_2$] (2) with Anilinium BF_4^-

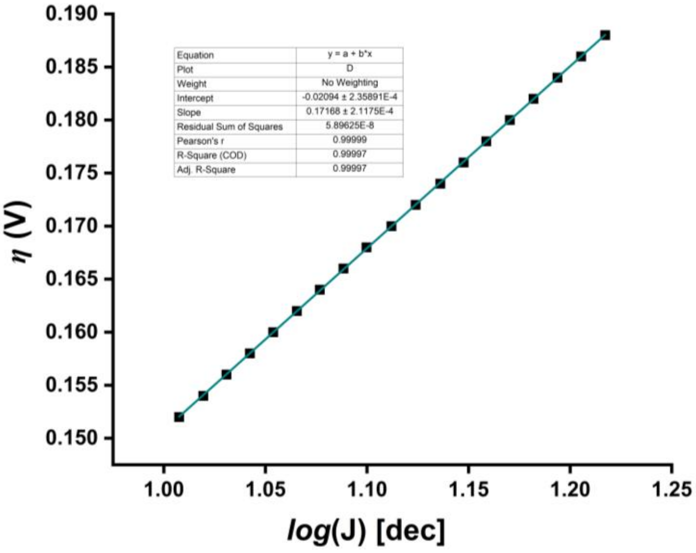

Figure S195. Tafel Slope of catalyst [(L^2)$BiCl_2$] **(2)** with Anilinium tetrafluoroborate (pK_a 10.62 in acetonitrile) is 171 mV/dec.

Calculation of Tafel slope for catalyst 3[(L^3)$BiCl_2$] (3) with 2-aminopyridinium BF_4^-

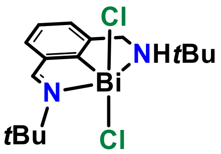

[(L^3)$Bi^{III}Cl_2$] (3)

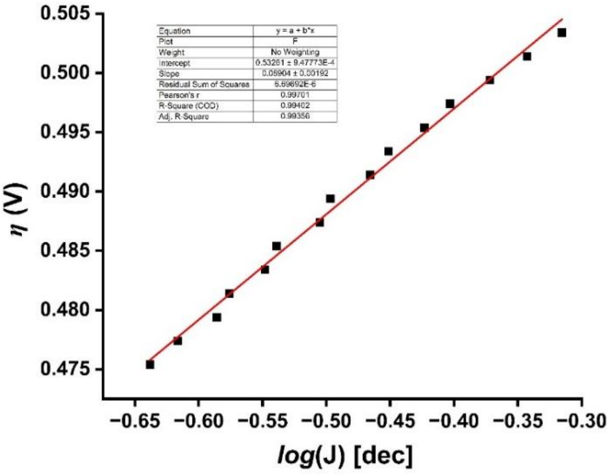

Figure S196. Tafel Slope of catalyst [(L^3)$BiCl_2$] **(3)** with 2-aminopyridinium BF_4^- (pK_a 14.47 in acetonitrile) is 89.04 mV/dec

Calculation of Tafel slope for catalyst [(L^3)$BiCl_2$] (3) with Anilinium BF_4^-

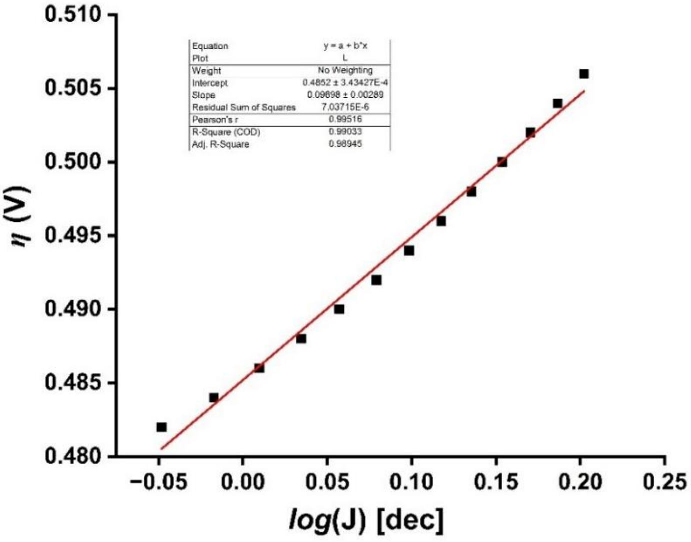

Figure S197. Tafel Slope of catalyst [(L^3)BiCl$_2$] **(3)** with anilinium tetrafluoroborate (pK_a 10.62 in acetonitrile) is 96.98 mV/dec.

Calculation of Tafel slope for catalyst [(L^4)BiCl$_2$] (4) with 2-aminopyridinium BF_4^-

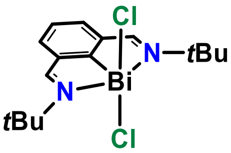

[(L^4)Bi^{III}Cl$_2$] (4)

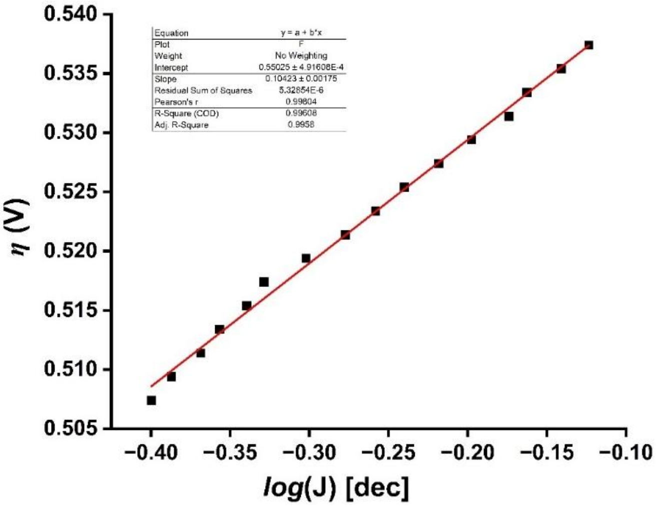

Figure S198. Tafel Slope of catalyst [(L^4)BiCl$_2$] **(4)** with 2-aminopyridinium BF_4^- (pK_a 14.47 in acetonitrile) is 104.23 mV/dec

Calculation of Tafel slope for catalyst [(L^4)BiCl$_2$] (4) with Anilinium BF_4^-

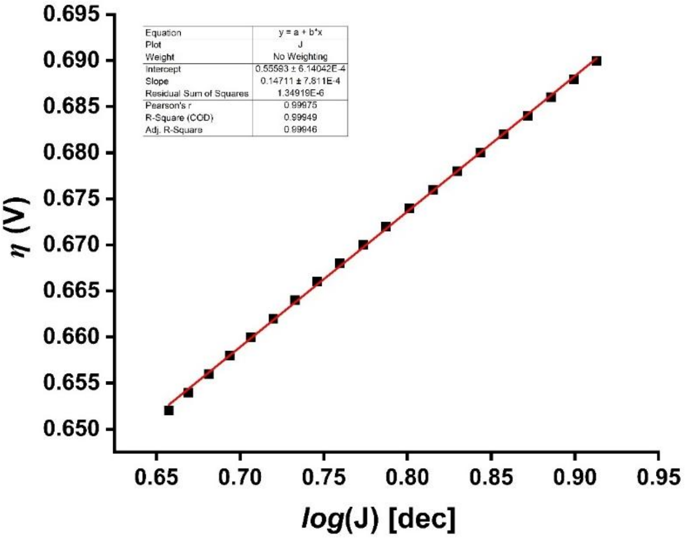

Figure S199. Tafel Slope of catalyst [(L^4)BiCl$_2$] **(4)** with Anilinium tetrafluoroborate (pK_a 10.62 in acetonitrile) is 147.11 mV/dec.

Table S25. Compiled Tafel slope data for catalyst [(L^1)BiCl$_2$] **(1),** [(L^2)BiCl$_2$] **(2),** [(L^3)BiCl$_2$] **(3)**, [(L^4)BiCl$_2$] **(4).**

CATALYST	2-aminopyridinium BF_4^- (pK_a 14.47) (Tafel Slope in mV/dec)	Anilinium BF_4^- (pK_a 10.62) (Tafel Slope in mV/dec)
**[(L^1)BiCl$_2$] (1)**	70.85	80.64
**[(L^2)BiCl$_2$] (2)**	98.66	171
**[(L^3)BiCl$_2$] (3)**	89.04	96.98
**[(L^4)BiCl$_2$] (4)**	104.23	147.11

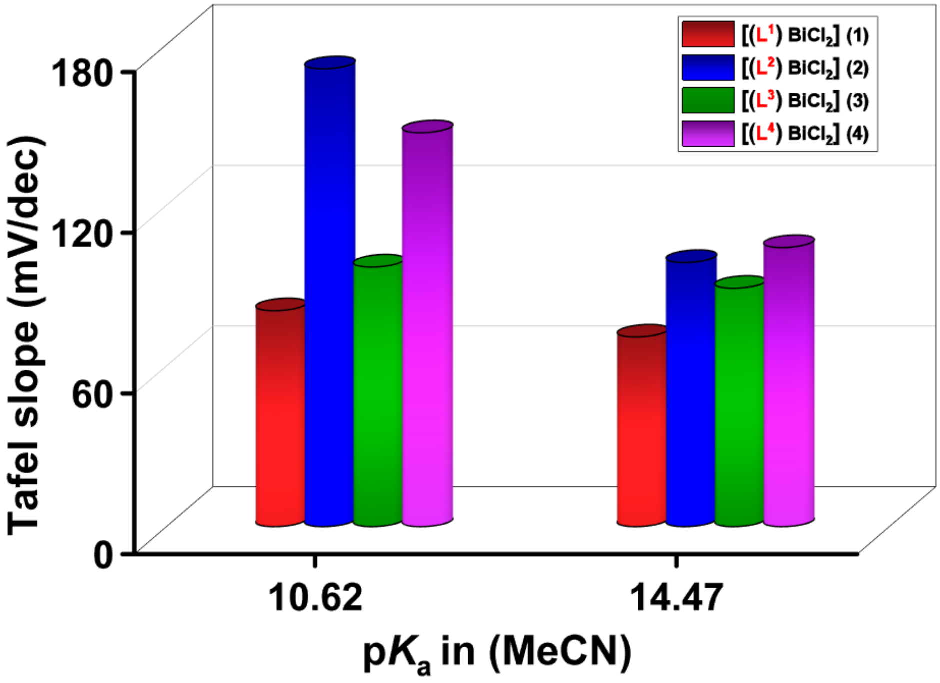

Figure S200. Tafel Slope comparison of catalyst [(L^1)BiCl$_2$] **(1)** and [(L^2)BiCl$_2$] **(2)**

Charge transfer coefficient (*α*)[18]

The charge transfer coefficient (α) is an important kinetic parameter in electrochemistry that describes how the activation energy changes with applied potential. In general (α) is taken as 0.5 ($0 < \alpha < 1$) However, experimentally α can be calculated from Tafel Plot.

From Tafel slope equation,

$$\eta = a + b \log(i)$$

b = 2.303 RT / (αnF) n = no. of electron involved in r.d.s

α = 2.303 RT / (nFb)

Table S26. Tafel slope and charge transfer coefficient (***α***) for catalyst [(L^1)$BiCl_2$] **(1),** [(L^2)$BiCl_2$] **(2),** [(L^3)$BiCl_2$] **(3)**, [(L^4)$BiCl_2$] **(4) with** 2-aminopyridinium BF_4^- (pK_a 14.47).

**Catalyst**	2-aminopyridinium BF_4^- (pK_a 14.47) (Tafel Slope in mV/dec)	Charge transfer coefficient (***α***)
**[(L^1)$BiCl_2$] (1)**	70.85	0.83
**[(L^2)$BiCl_2$] (2)**	98.66	0.60
**[(L^3)$BiCl_2$] (3)**	89.04	0.66
**[(L^4)$BiCl_2$] (4)**	104.23	0.57

UV/Vis Spectroelectrochemical Studies (XV)

To probe the in situ formed Bi(I) active species under electrochemical conditions, UV–vis spectroelectrochemical experiments were carried out using a SEC-CT thin-layer quartz spectroelectrochemical cell equipped with a platinum grid mesh working electrode, a platinum wire counter electrode, and an Ag/AgCl (Vycor frit) pseudo-reference electrode connected to a potentiostat operating in amperometric mode and coupled to a UV–vis spectrophotometer. The reduction of Bismuth (III) complex **[(L^1)$BiCl_2$] (1)** (0.5 mmol) in CH_3CN and 0.1 M of $TBAPF_6$ have been investigated by in situ UV-Vis Spectroscopy. On applying a constant potential of −1.25 V vs Ag/AgCl and simultaneously monitoring UV-Vis spectrum, shows the formation of active catalysts Bi (I) **[(L^1)Bi] (1')**. Reduction of **[(L^1)$BiCl_2$] (1)** resulted in appearance of three intense absorptions bands centered at 386, 487 and 604 nm for **[(L^1)Bi] (1')**.

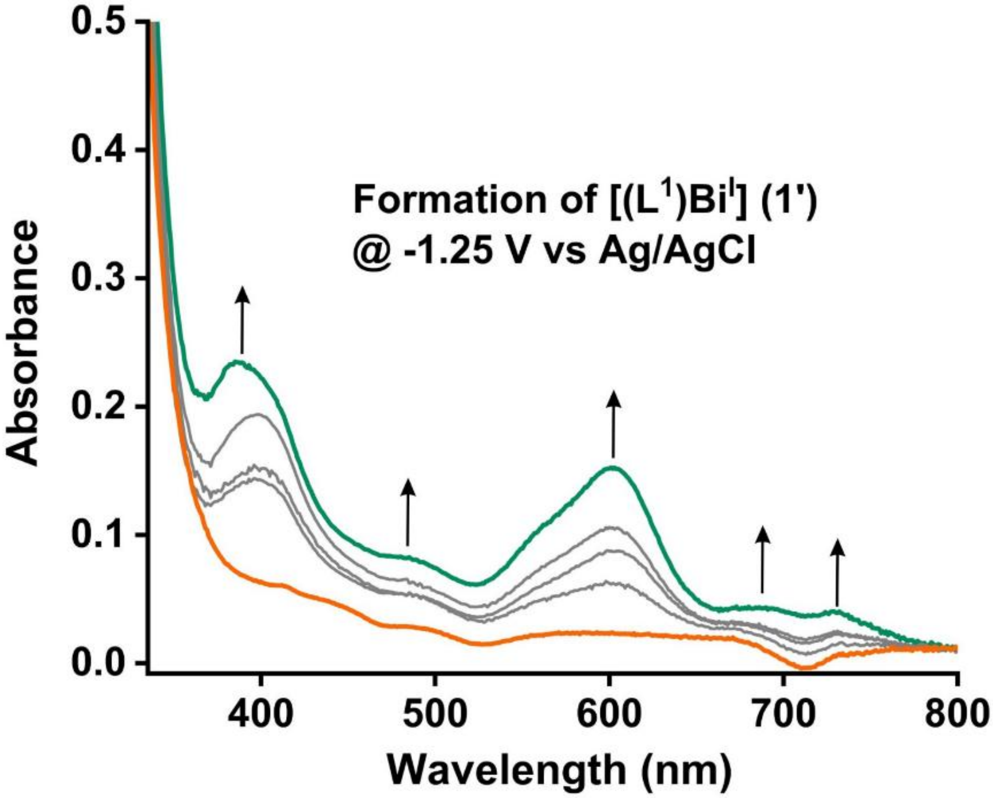

Figure S201. UV-Vis absorption spectrum for formation of bismuthinidene [L^1Bi^I] (**1′**) in acetonitrile from the corresponding [$L^1Bi^{III}Cl_2$] (**1**) catalyst under spectroelectrochemical condition with an applied potential of −1.25 V vs Ag/AgCl.

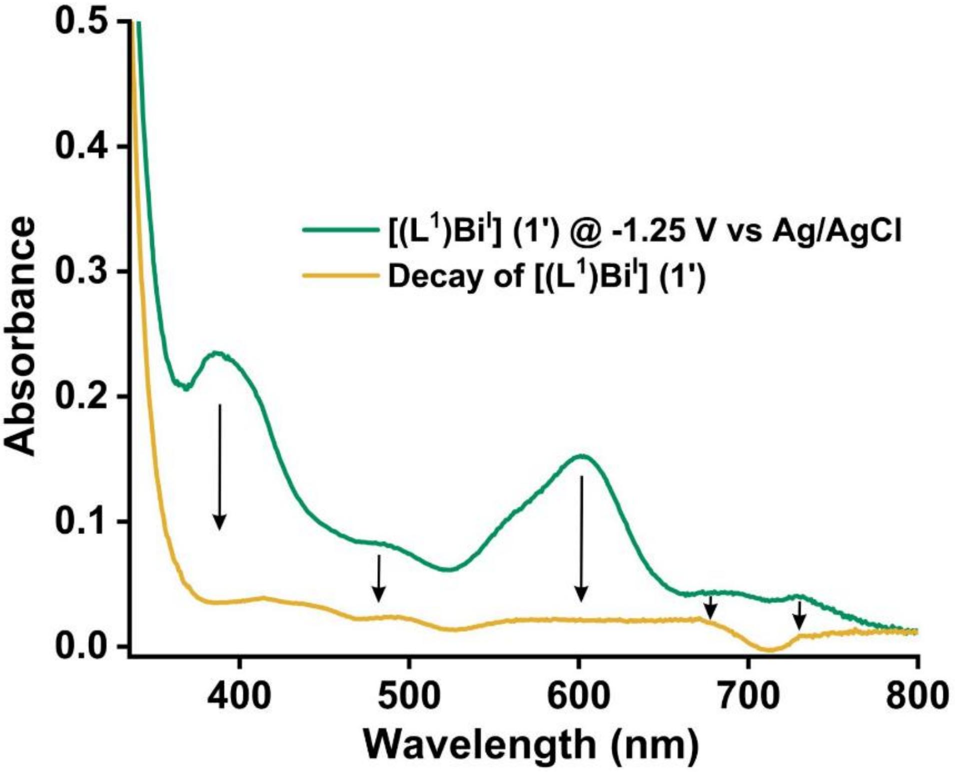

Figure S202. UV-Vis absorption spectrum for the rapid decay of bismuthinidene [(L^{1})Bi^{I}] (**1′**) in acetonitrile.

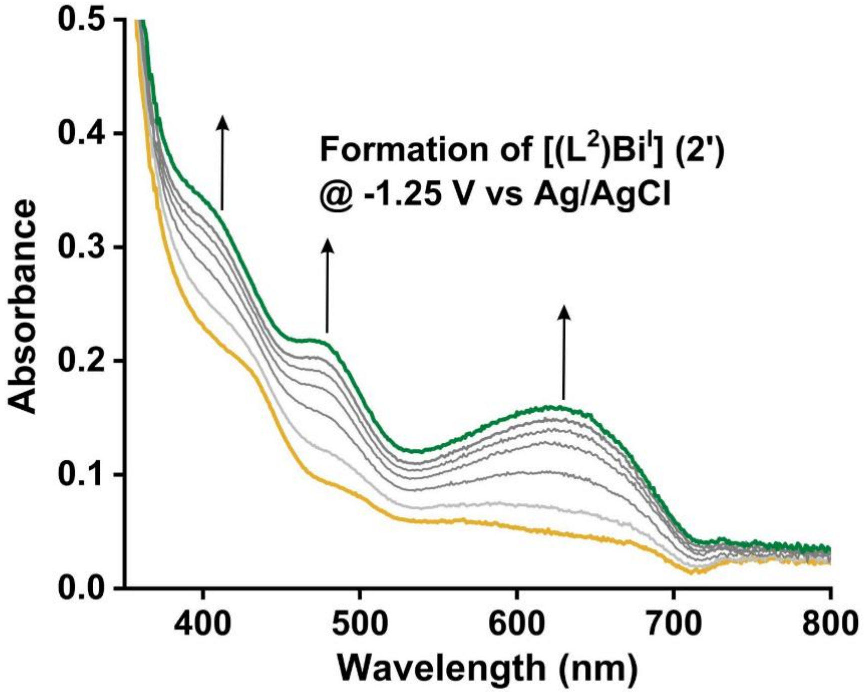

Figure S203. UV-Vis absorption spectrum for formation of bismuthinidene [L^2Bi^I] (**2′**) in acetonitrile from the corresponding [$L^2Bi^{III}Cl_2$] (**2**) catalyst under spectroelectrochemical condition with an applied potential of −1.25 V vs Ag/AgCl.

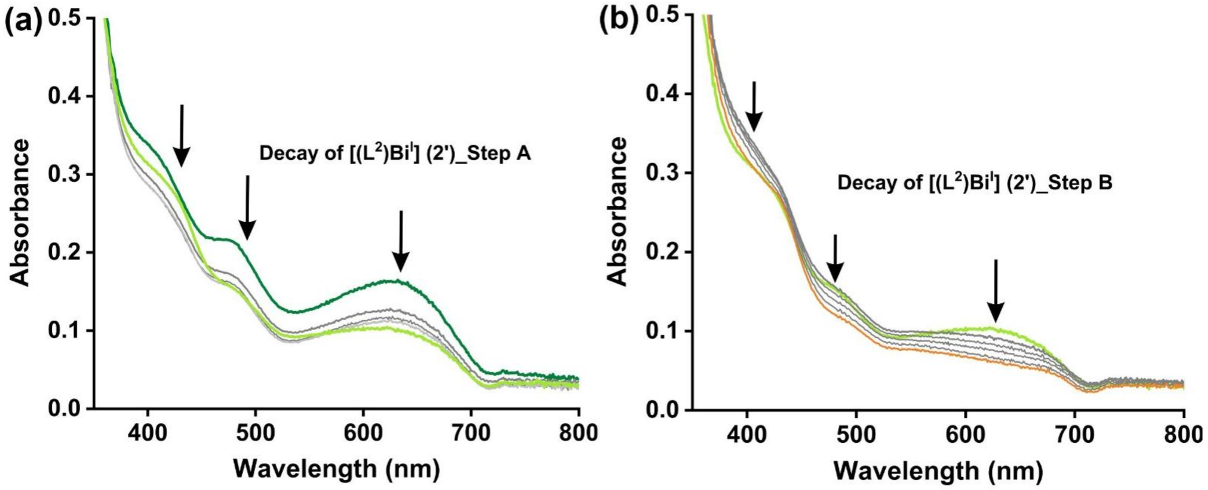

Figure S204. UV-Vis absorption spectra showing stepwise decay (a) Step (A) and (b) Step (B) of bismuthinidene [(L[2])Bi[I]] (**2′**) in acetonitrile. (−1.25 V Ag/AgCl in acetonitrile)

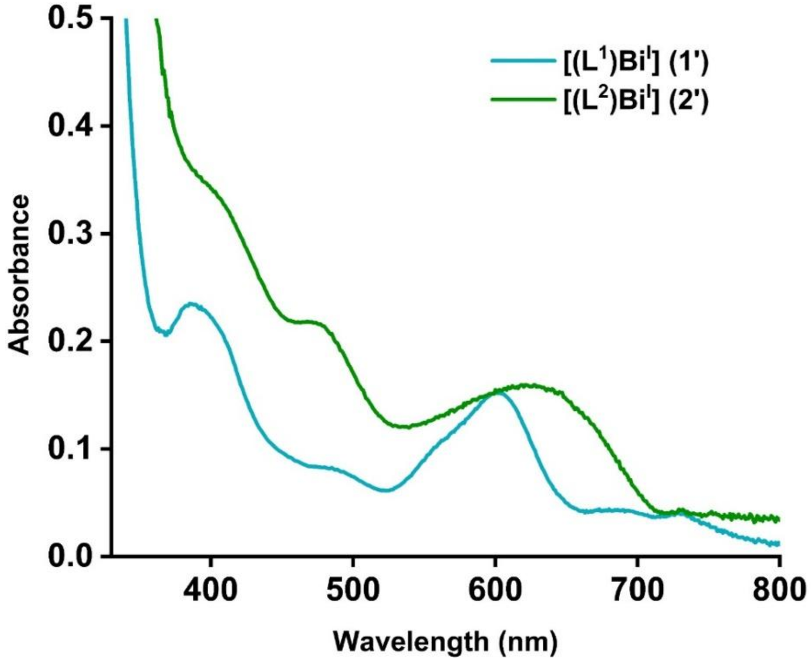

Figure S205. UV-Vis spectra of Bi(I) species [(L[1])Bi] **(1')** and [(L[2])Bi] **(2')** generated insitu from catalyst [(L[1])$BiCl_2$] **(1)** and [(L[2])$BiCl_2$] **(2**) respectively under spectroelectrochemical conditions (−1.25 V Ag/AgCl in acetonitrile).

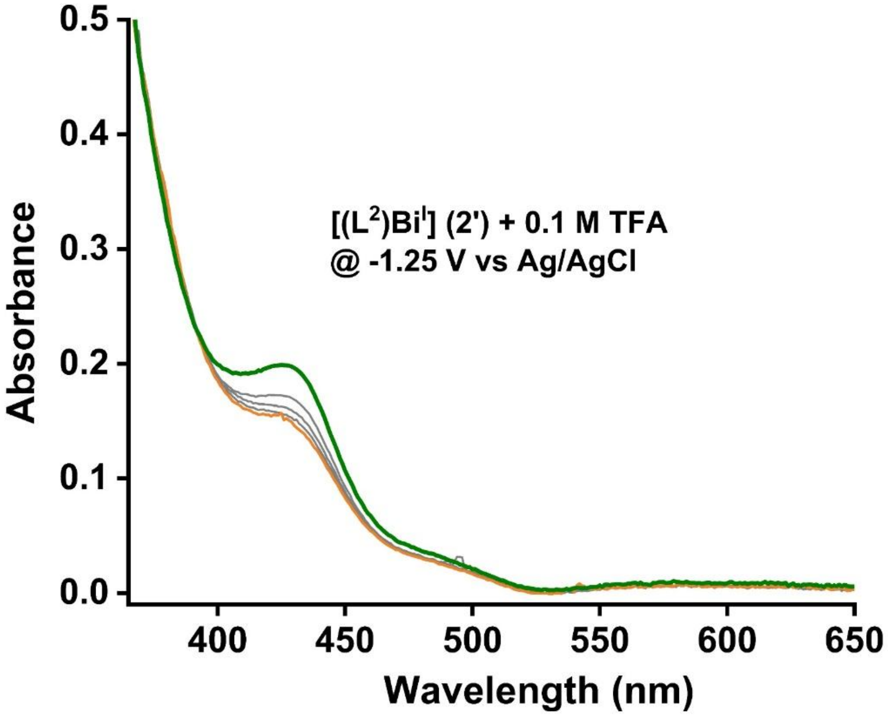

Figure S206. UV-Vis absorption change after adding 0.1 M TFA (pK_a = 12.65 in acetonitrile) to bismuthinidene [L^2Bi^I] **(2′)** generated in acetonitrile from the corresponding [$L^2Bi^{III}Cl_2$] (**2**) catalyst under electrochemical condition with an applied potential of −1.25 V vs Ag/AgCl.

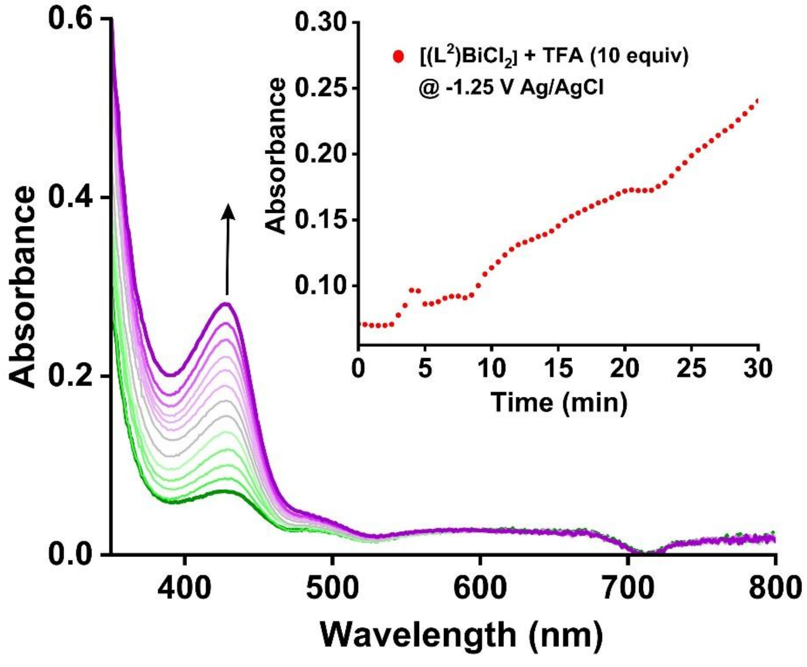

Figure S207. UV-Vis absorption spectra showing formation of peak at 430 nm when [$L^2Bi^{III}Cl_2$] (**2**) and 10 equiv. of TFA (pK_a = 12.65 in acetonitrile) was subjected to an applied potential of −1.25 V vs Ag/AgCl under spectroelectrochemical condition (inset shows the time profile of formation of the peak at 430 nm)

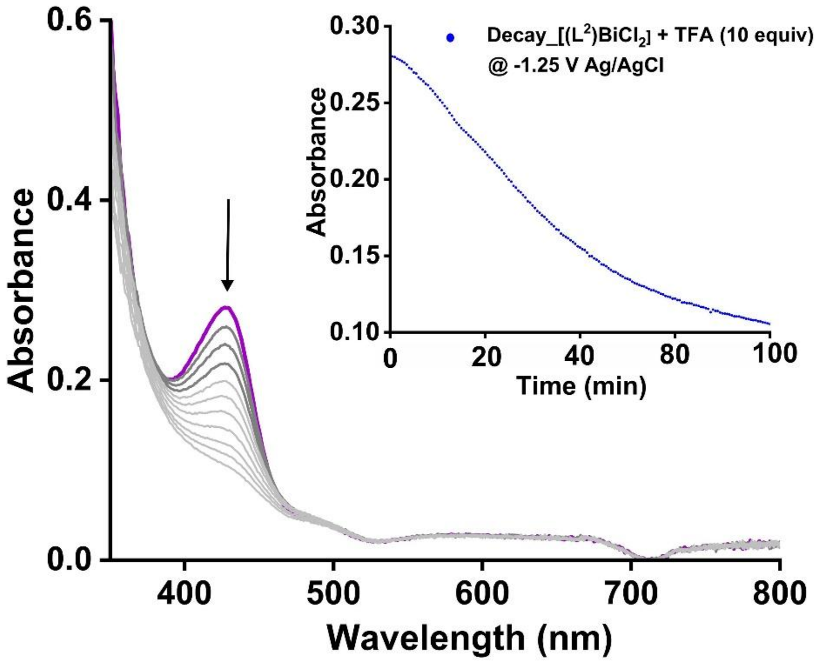

Figure S208. UV-Vis absorption spectra showing decay of peak at 430 nm when $[L^2Bi^{III}Cl_2]$ (**2**) and 10 equiv. of TFA (pK_a = 12.65 in acetonitrile) was subjected to an applied potential of −1.25 V vs Ag/AgCl under spectroelectrochemical condition (inset shows the time profile of decay of the peak at 430 nm)

Controlled Potential Electrolysis (XVI)

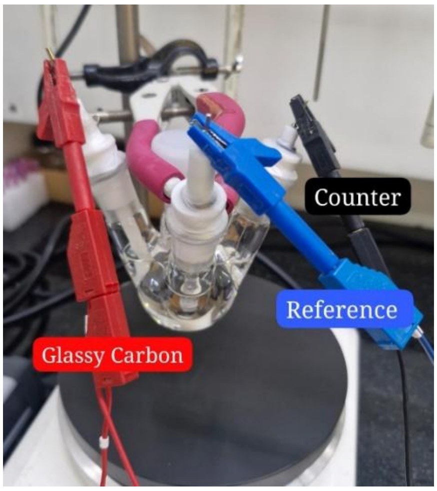

Figure S209: In house customized CPE setup.

The CPE experiment was performed in a 35 mL electrochemical cell, with acetonitrile as the solvent, 1 mM of catalyst**,** and 0.2 M of proton source, with applied potential as per proton source and time 1.5 h to 2 h (Table S33)

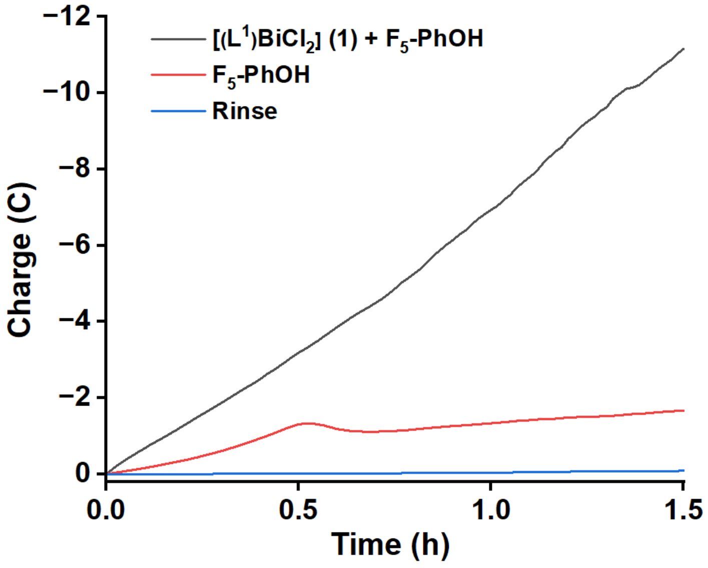

Figure S210. Charge passed vs time plot for CPE experiment of 1 mM of catalyst **[(L[1])BiCl$_2$] (1),** and 0.2 M of F_5-PhOH in 35 mL acetonitrile at potential −1.8 V (vs Ag/AgCl) for 1.5 hours. Overlay of corresponding rinse (blue) and blank acid (red) CPE without catalyst.

Table S27. Summary of GC-TCD analyses from headspace injection for CPE experiment of **[(L[1])BiCl$_2$] (1)** with Pentafluorophenol

Catalyst	Acid	Experimental mole	Theoretical mole	Experimental Charge	F.E
**[(L[1])BiCl$_2$] (1)**	F_5-PhOH	$6.01x10^{-5}$	5.77×10^{-5}	−11.15 C	104%

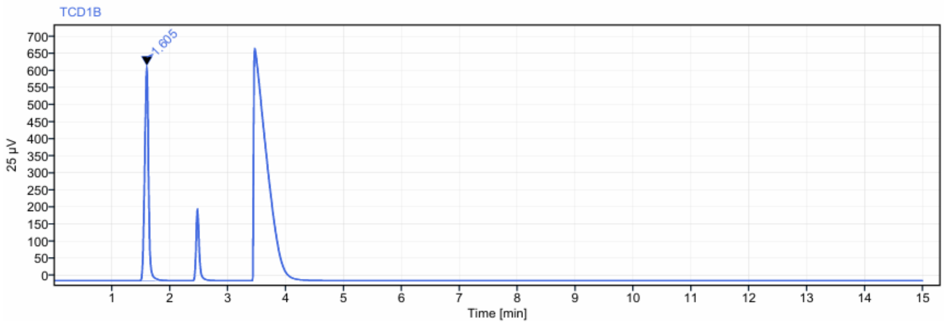

Signal: TCD1B

Name	RT [min]	RF	Area	Area %	Concentration []
H2	1.61		2876.832	100.00	

Figure S211. GC-TCD Analysis Chromatogram showing hydrogen formation for **[(L[1])BiCl$_2$] (1)** with F_5-PhOH

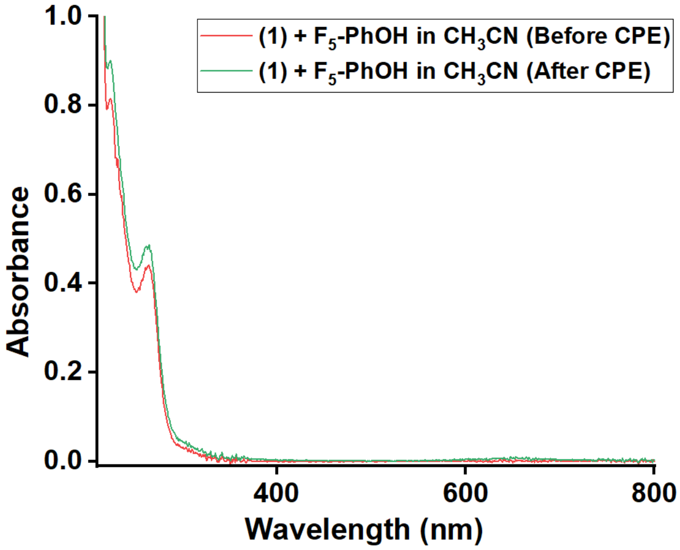

Figure S212. UV-Vis spectra of **[(L[1])BiCl$_2$] (1)** with F_5-PhOH, before (red) and after (green) CPE showing no catalyst decomposition.

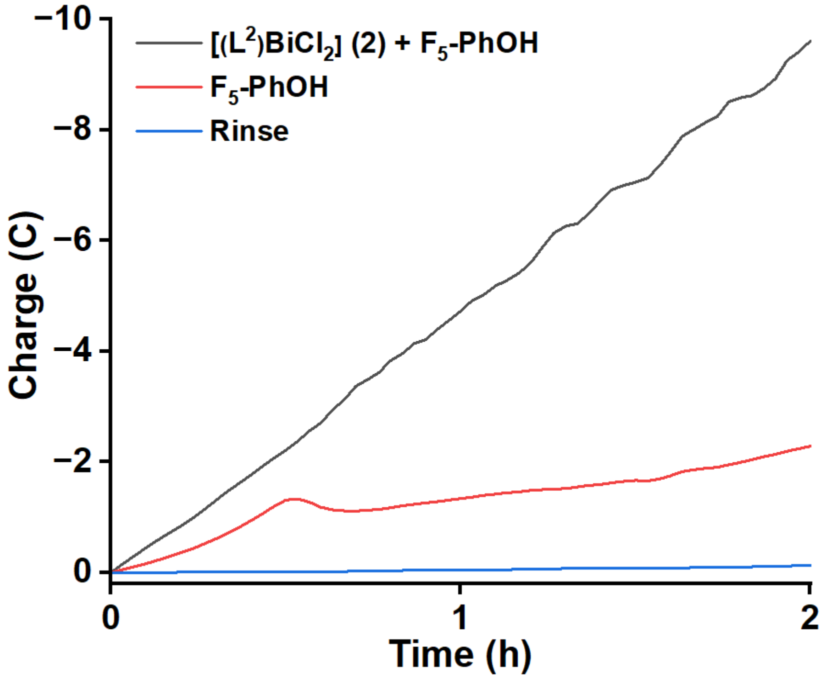

Figure S213. Charge passed vs time plot for CPE experiment of 1 mM of catalyst **[(L^2)BiCl$_2$] (2),** and 0.2 M of F_5-PhOH in 35 mL acetonitrile at potential −1.8 V (vs Ag/AgCl) for 2 hours. Overlay of corresponding rinse (blue) and blank acid (red) CPE without catalyst.

Table S28. Summary of GC-TCD analyses from headspace injection for CPE experiment of **[(L^2)BiCl$_2$] (2)** with Pentafluorophenol

Catalyst	Acid	Experimental mole	Theoretical mole	Experimental Charge	F.E
**[(L^2)BiCl$_2$] (2)**	F_5-PhOH	5.18×10^{-5}	5.53×10^{-5}	−10.69 C	104%

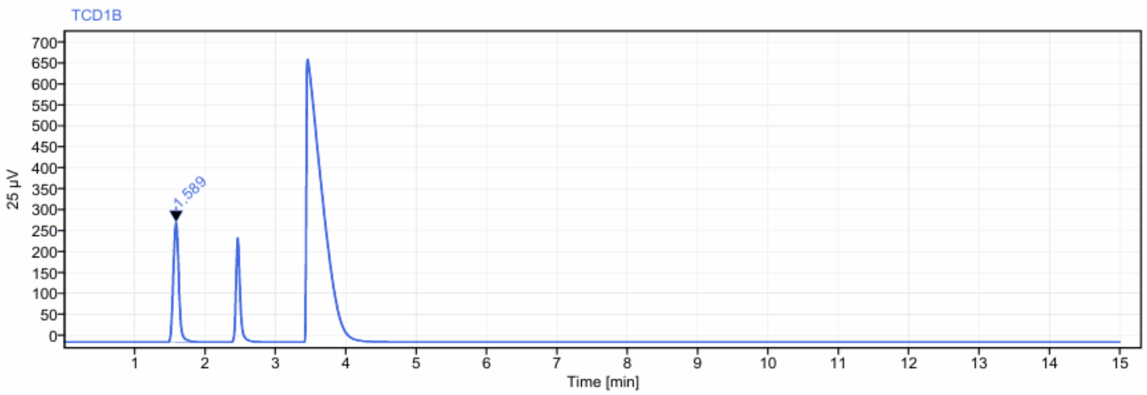

Signal: TCD1B

Name	RT [min]	RF	Area	Area %	Concentration []
H2	1.59		1551.683	100.00	

Figure S214. GC-TCD Analysis Chromatogram for **[(L^2)BiCl$_2$] (2)** with F_5-PhOH showing hydrogen formation.

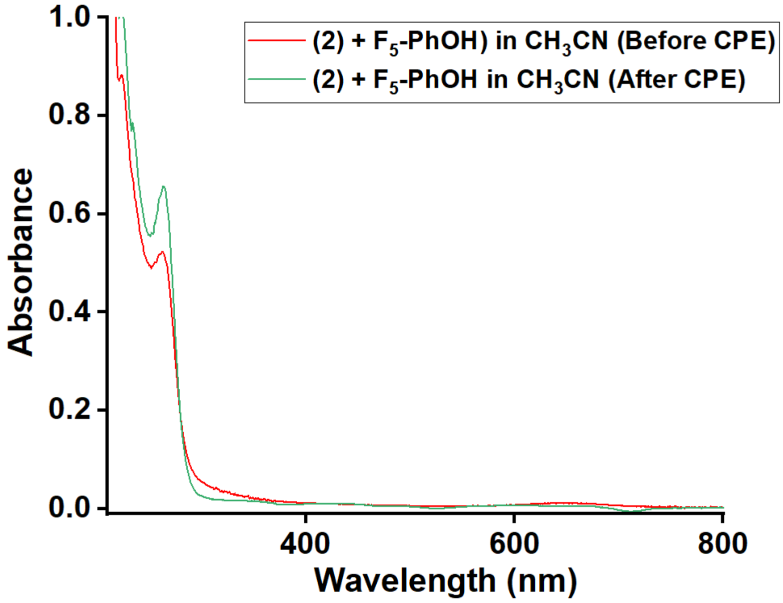

Figure S215. UV-Vis spectra of **[(L^2)BiCl$_2$] (2)** with F_5-PhOH, before (red) and after (green) CPE showing no catalyst decomposition.

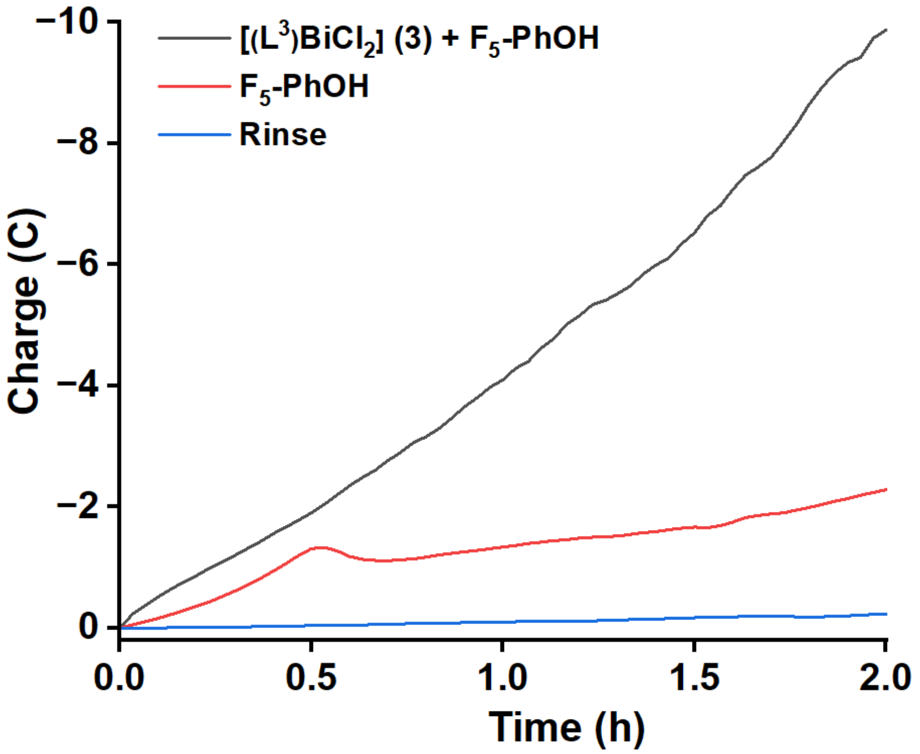

Figure S216. Charge passed vs time plot for bulk electrolysis experiments for 1 mM of catalyst **[(L^3)BiCl$_2$] (3),** and 0.2 M of F_5-PhOH in 35 mL acetonitrile at potential −1.8 V (vs Ag/AgCl) for 2 hours. Overlay of corresponding rinse (blue) and blank acid (red) CPE without catalyst.

Table S29. Summary of GC-TCD analyses from headspace injection for CPE experiment of **[(L^3)BiCl$_2$] (3)** with Pentafluorophenol

Catalyst	Acid	Experimental mole	Theoretical mole	Experimental Charge	F.E
**[(L^3)BiCl$_2$] (3)**	F_5-PhOH	$5.1x10^{-5}$	$5.1x10^{-5}$	−9.87 C	99%

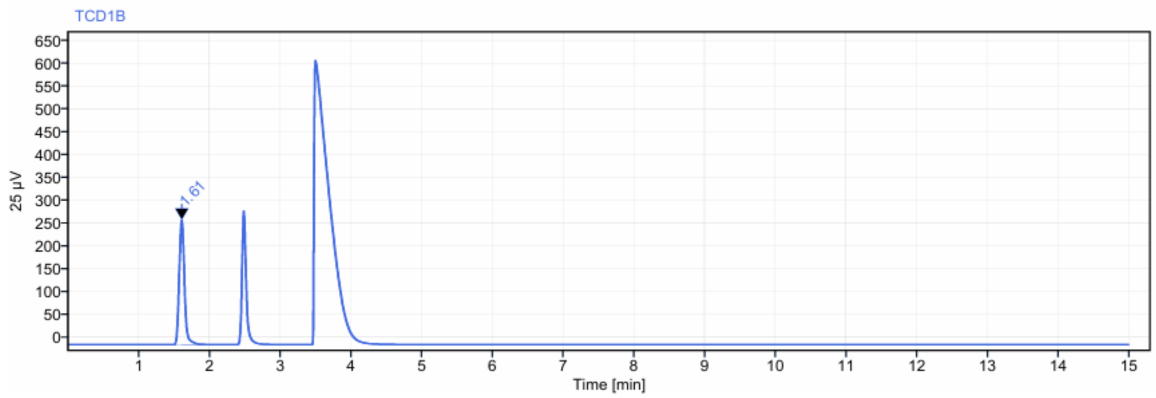

Signal: TCD1B

Name	RT [min]	RF	Area	Area %	Concentration []
H2	1.61		1408.399	100.00	

Figure S217. GC-TCD Analysis Chromatogram for **[(L^3)BiCl$_2$] (3)** with F_5-PhOH showing hydrogen formation.

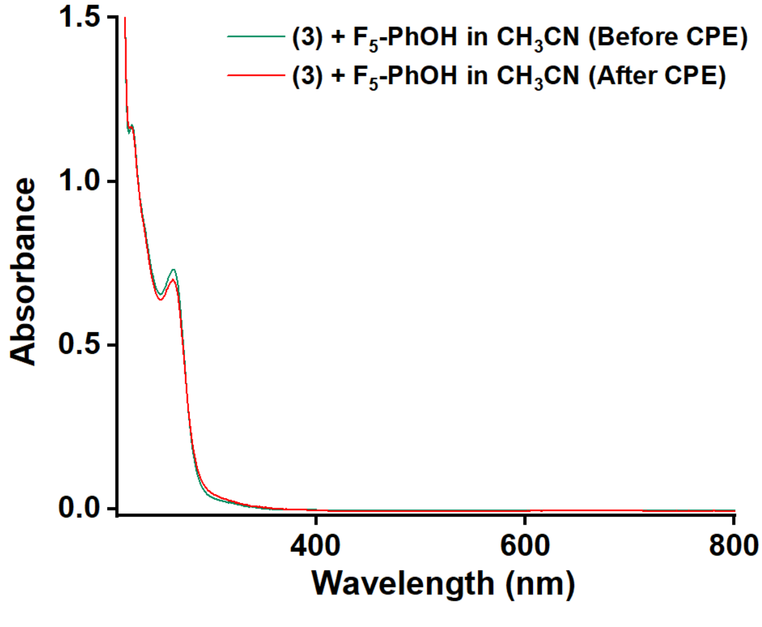

Figure S218. UV-Vis spectrum of bulk electrolysis experiment of **[(L^3)BiCl$_2$] (3)** with F_5-PhOH, before (green) and after (red) CPE showing no catalyst decomposition.

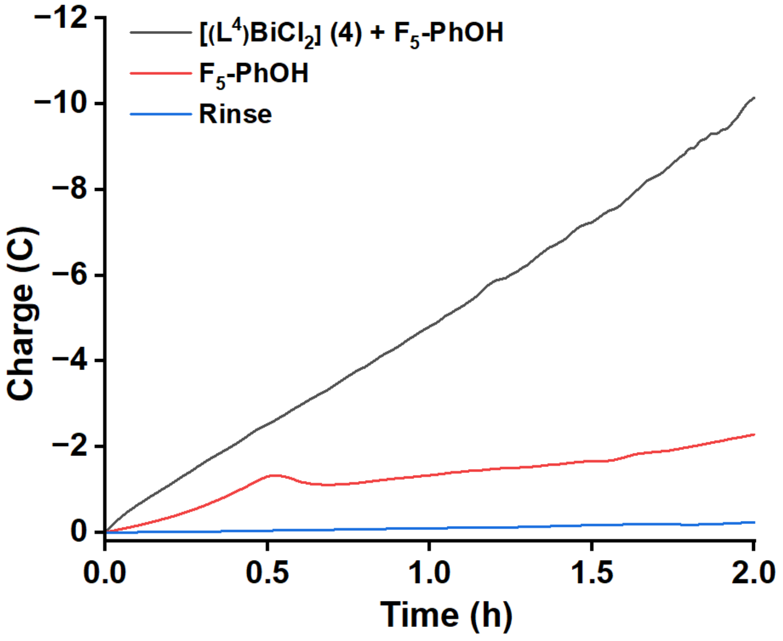

Figure S219. Charge passed vs time plot for bulk electrolysis experiments for 1 mM of catalyst **$[(L^4)BiCl_2]$ (4),** and 0.2 M of F_5-PhOH in 35 mL acetonitrile at potential −1.8 V (vs Ag/AgCl) for 2 hours. Overlay of corresponding rinse (blue) and blank acid (red) CPE without catalyst.

Table S30. Summary of GC-TCD analyses from headspace injection for CPE experiment of **$[(L^4)BiCl_2]$ (4)** with Pentafluorophenol

Catalyst	Acid	Experimental mole	Theoretical mole	Experimental Charge	F.E
**$[(L^4)BiCl_2]$ (4)**	F_5-PhOH	5.49 $\times10^{-5}$	5.2$\times10^{-5}$	−10.14 C	104%

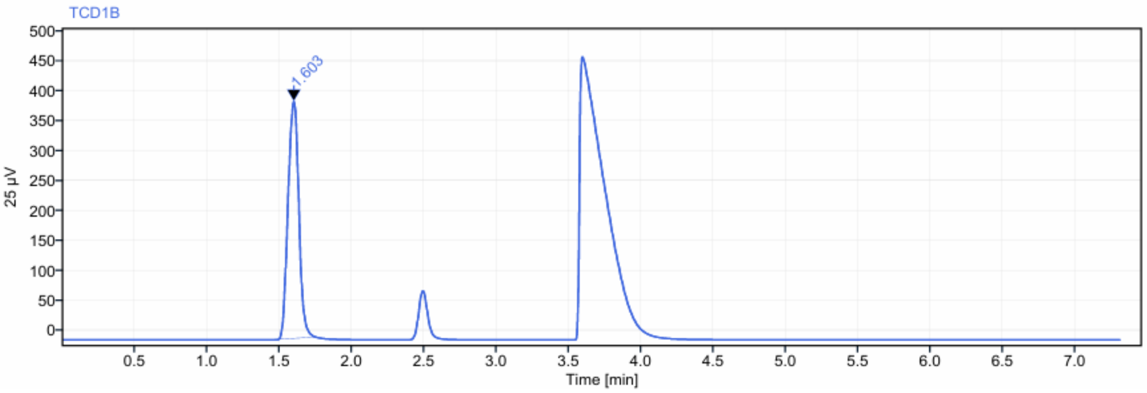

Signal: TCD1B

Name	RT [min]	RF	Area	Area %	Concentration []
H2	1.60		2054.548	100.00	

Figure S220. GC-TCD Analysis Chromatogram for **[(L⁴)BiCl₂] (4)** with F_5-PhOH showing hydrogen formation.

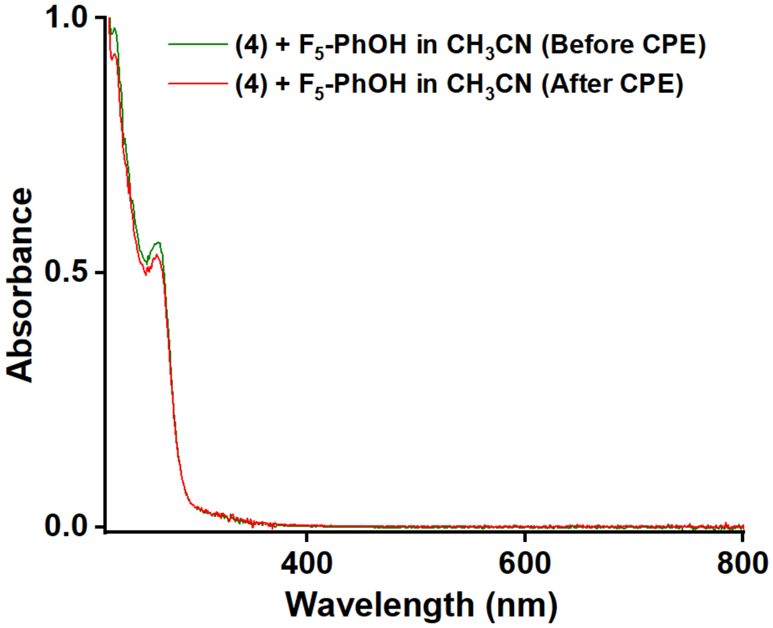

Figure S221. UV-Vis spectra of **[(L⁴)BiCl₂] (4)** with F_5-PhOH, before (green) and after (red) CPE showing no catalyst decomposition.

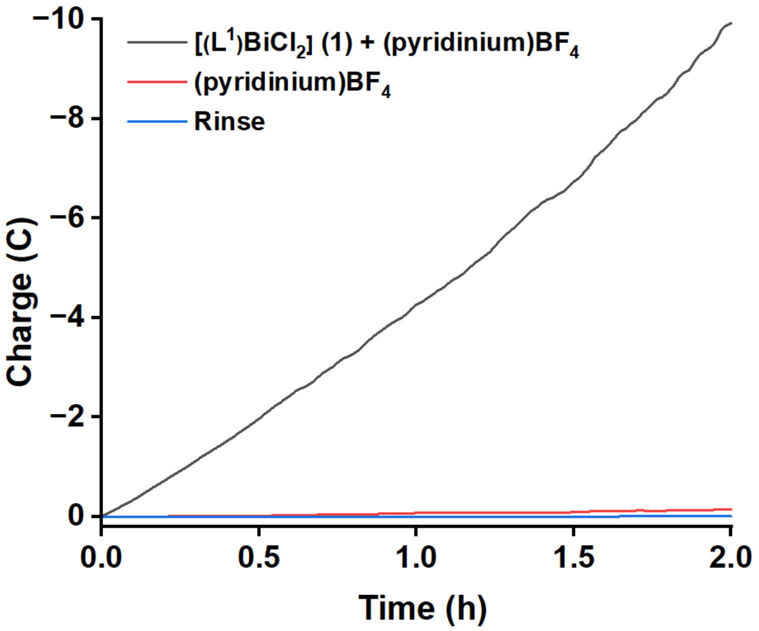

Figure S222. Charge passed vs time plot for bulk electrolysis experiments for 1 mM of catalyst **[(L[1])BiCl$_2$] (1),** and 0.2 M of pyridinium BF_4^- in 35 mL ACN at potential −1.25 V for 2 hours. Overlay of corresponding rinse (blue) and blank acid (red) CPE without catalyst.

Table S31. Summary of GC-TCD analyses from headspace injection for CPE experiment of **[(L[1])BiCl$_2$] (1)** with pyridinium tetrafluoroborate.

Catalyst	Acid	Experimental mole	Theoretical mole	Experimental Charge	F.E
**[(L[1])BiCl$_2$] (1)**	Pyridinium BF_4^-	$5.33x10^{-5}$	$5.14x10^{-5}$	−9.92 C	103%

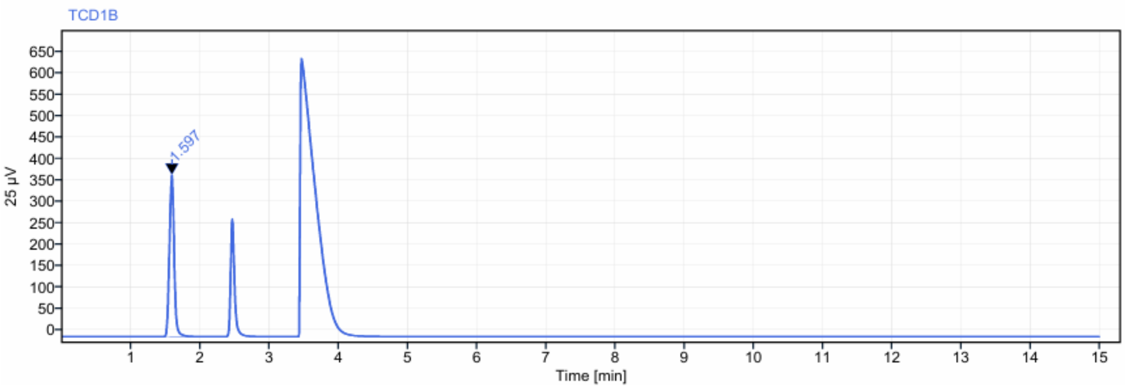

Signal: TCD1B

Name	RT [min]	RF	Area	Area %	Concentration []
H2	1.60		1794.086	100.00	

Figure S223. GC-TCD Analysis Chromatogram for **[(L^1)BiCl$_2$] (1)** with Pyridinium BF_4^- showing hydrogen formation.

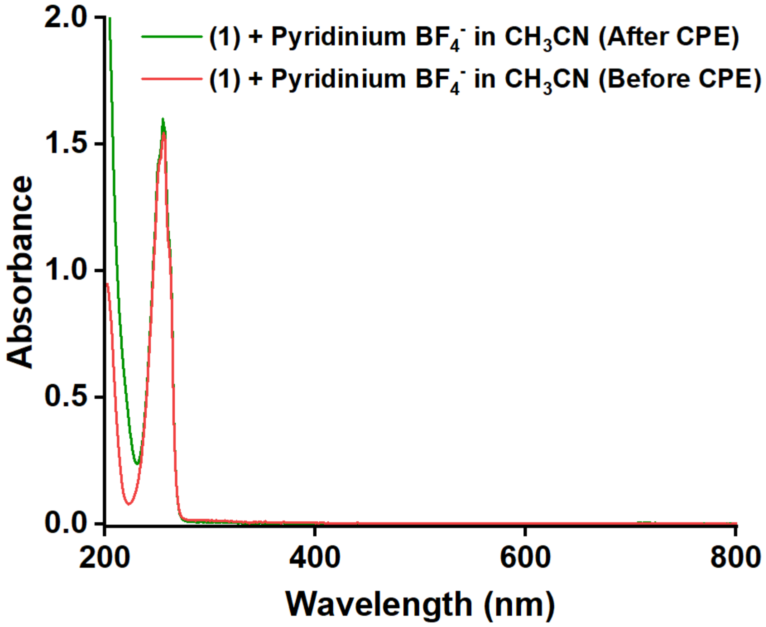

Figure S224. UV-Vis spectra of **[(L^1)BiCl$_2$] (1)** with Pyridinium BF_4^-, before (red) and after (green) CPE showing no catalyst decomposition.

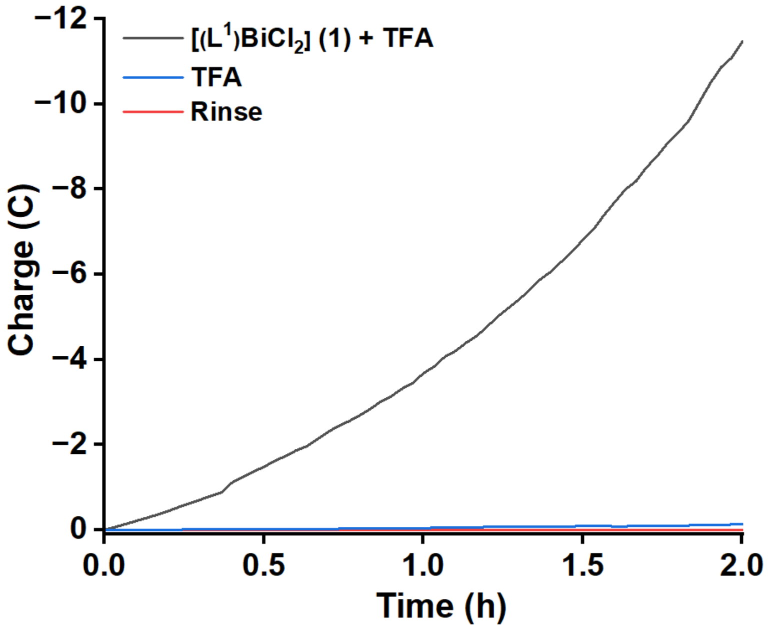

Figure S225. Charge passed vs time plot for bulk electrolysis experiments for 1 mM of catalyst **[(L[1])BiCl$_2$] (1),** and 0.2 M of TFA in 35 mL ACN at potential −1.2 V (vs Ag/AgCl) for 2 hours. Overlay of corresponding rinse (blue) and blank acid (red) CPE without catalyst.

Table S19. Summary of GC-TCD analyses from headspace injection for CPE experiment of of **[(L[1])BiCl$_2$] (1)** with TFA

Catalyst	Acid	Experimental mole	Theoretical mole	Experimental Charge	F.E
**[(L[1])BiCl$_2$] (1)**	TFA	5.90 x10^{-5}	5.94x10^{-5}	−11.47 C	99%

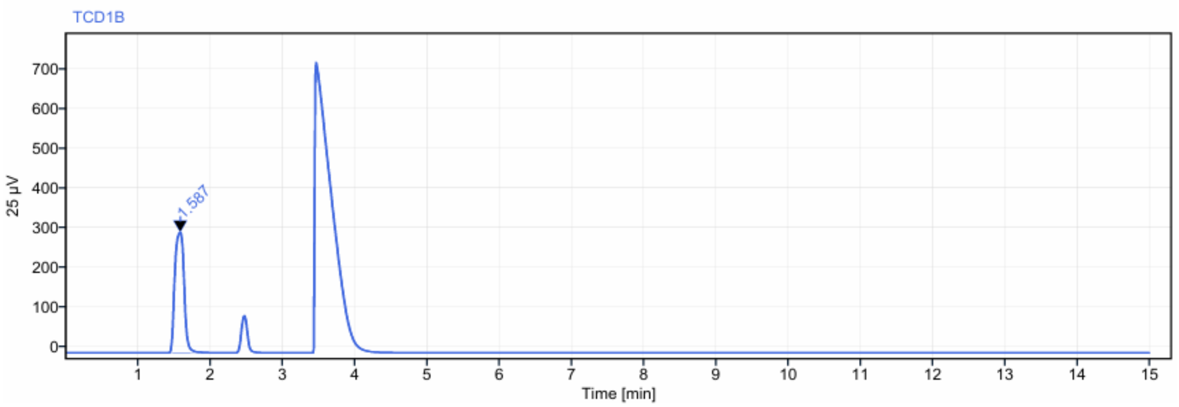

Signal: TCD1B

Name	RT [min]	RF	Area	Area %	Concentration []
H2	1.59		2700.685	100.00	

Figure S226. GC-TCD Analysis Chromatogram for **[(L[1])BiCl$_2$] (1)** with TFA showing hydrogen formation.

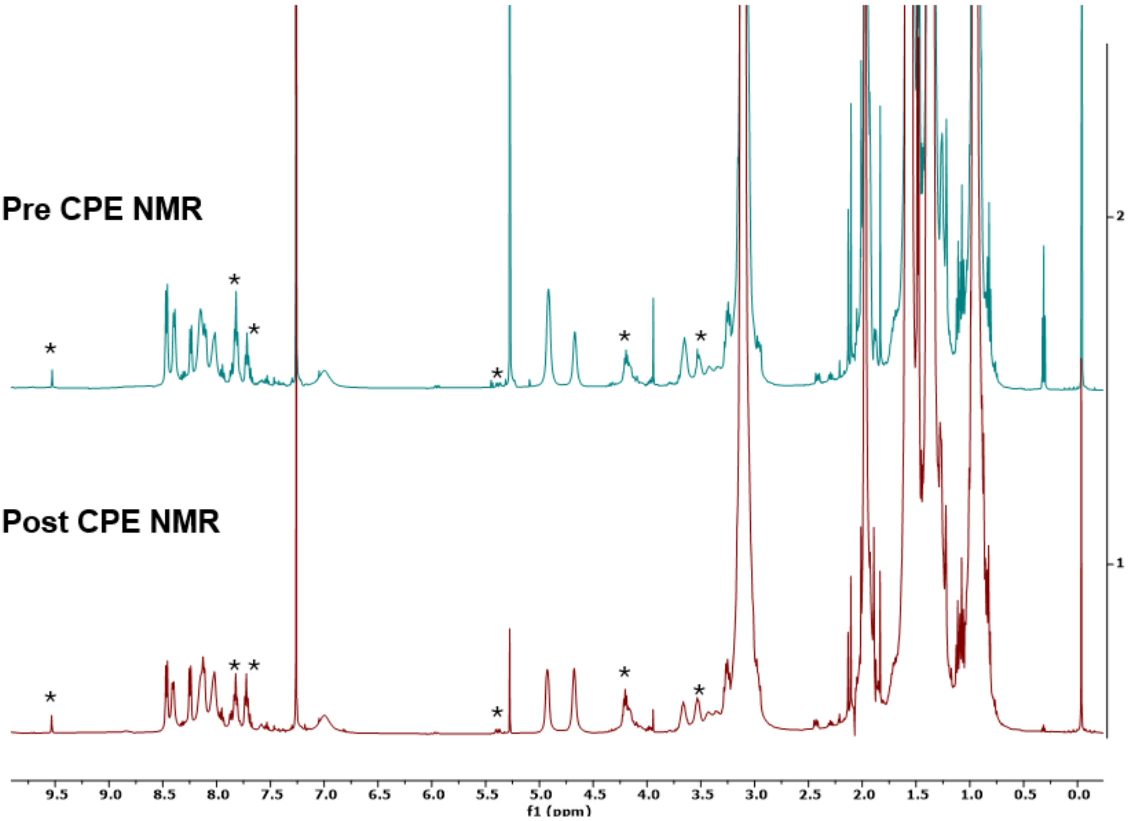

Figure S227. ^{1}H NMR of catalyst [(L[1])BiCl$_2$] **(1)** with TFA before CPE (sky blue) and after CPE (red) showing no catalyst decomposition under electrochemical condition. *Marked peaks are from the catalyst.

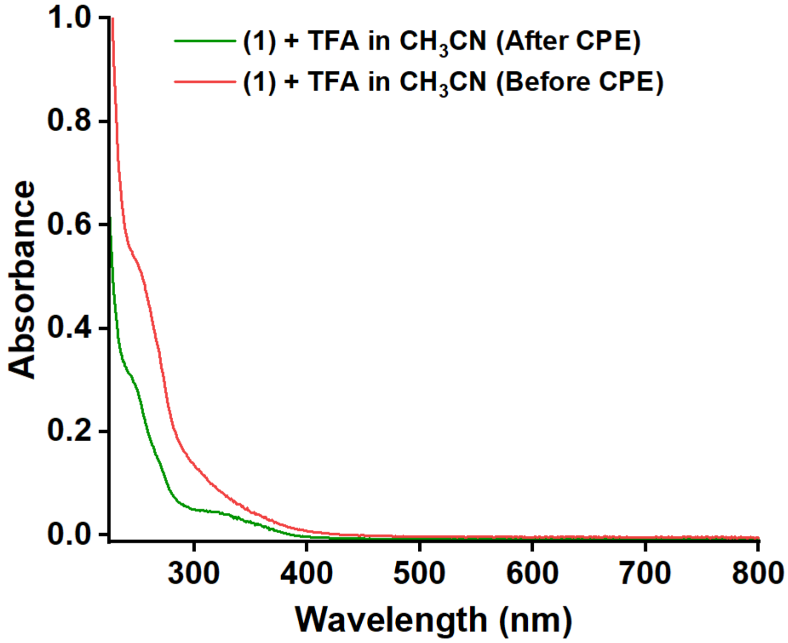

Figure S228. UV-Vis spectra of **[(L¹)BiCl₂] (1)** with TFA, before (red) and after (green) CPE showing no catalyst decomposition.

Table S33. Summary of CPE Experiments

Catalyst 1 mM	Acid (0.2 M)	Applied potential Vs Ag/AgCl	Exp. mole	Theoretical mole	Charge (C)	F.E %
**[(L^1)BiCl$_2$] (1)**	F_5-PhOH	−1.8 V	6.01x10^{-5}	5.77x10^{-5}	−11.15	104%
**[(L^2)BiCl$_2$] (2)**	F_5-PhOH	−1.8 V	5.18x10^{-5}	5.53x10^{-5}	−10.69	104%
**[(L^3)BiCl$_2$] (3)**	F_5-PhOH	−1.8 V	5.1x10^{-5}	5.1x10^{-5}	−9.87	99%
**[(L^4)BiCl$_2$] (4)**	F_5-PhOH	−1.8 V	5.49x10^{-5}	5.2x10^{-5}	−10.14	104%
**[(L^1)BiCl$_2$] (1)**	Pyridinium BF_4^-	−1.25 V	5.33x10^{-5}	5.14x10^{-5}	−9.92	103%
**[(L^1)BiCl$_2$] (1)**	TFA	−1.2 V	5.90 x10^{-5}	5.94x10^{-5}	−11.47	99%

Computational study (XVII)

1. Computational Details

All computations were carried out using the ORCA 5.0.3 quantum chemical program package.[19,20] Geometries were optimized using the hybrid-GGA PBE0[21] functional in conjunction with the def2-SVP basis sets.[22] To accelerate the calculations, the resolution-of-the-identity approximation for the Coulomb term (RIJ) together with the chain-of-spheres approximation for Hartree–Fock exchange (RIJCOSX) was applied.[23] The atom-pairwise dispersion correction with Becke–Johnson damping (D3BJ) was employed to account for noncovalent interactions.[24] During geometry optimization and frequency calculations, all atoms were fully relaxed. Subsequent numerical frequency calculations were performed for the optimized geometries to confirm that they correspond to stationary points characterized by the absence of imaginary frequencies, whereas transition state structures were verified by the presence of a single imaginary frequency. Gibbs free energies were obtained from single-point calculations at the PBE0-D3BJ/def2-TZVP[25] level of theory based on the PBE0-D3BJ/def2-SVP optimized geometries. The SMD continuum solvation model[26] was employed to account for solvent effects using acetonitrile as the implicit solvent. All reported values correspond to relative Gibbs free energies computed at the SMD (acetonitrile)-PBE0-D3BJ/def2-TZVP // PBE0-D3BJ/def2-SVP level of theory. To gain further insight into the electronic structure, quasi-restricted orbitals (QROs) were analyzed using ORCA, whereas Intrinsic Bonding Orbital (IBO) analyses were performed using the IBOview program.[27]

2. Quasi Restricted Orbital (QRO) Analysis

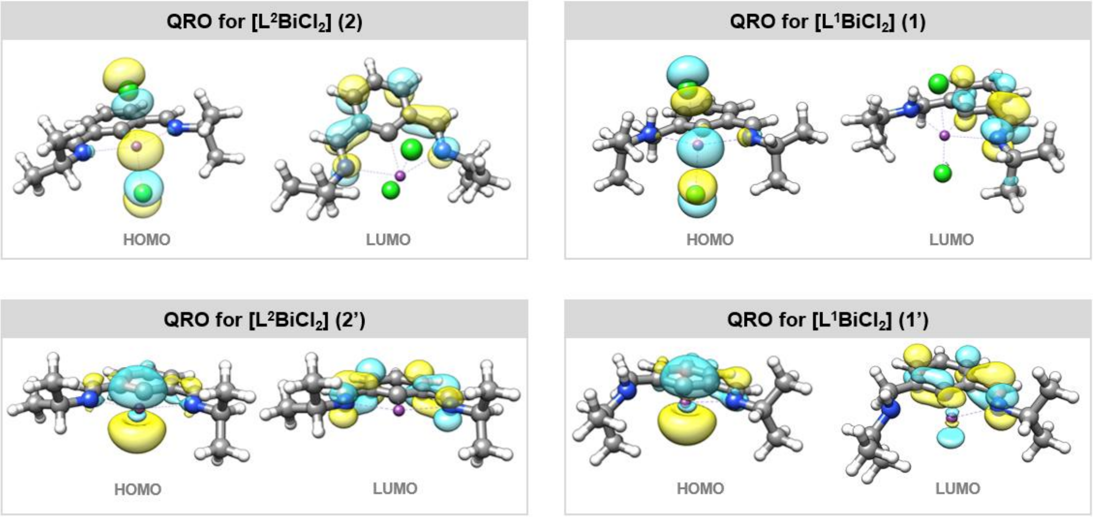

Figure S229. Quasi-Restricted Orbitals (QROs) showing HOMO-LUMO structure of **[(L^2)BiCl$_2$] (2), [(L^1)BiCl$_2$] (1), as well as reduced bismuthidine [(L^2)Bi] (2′) and [(L^1)Bi] (1′)**

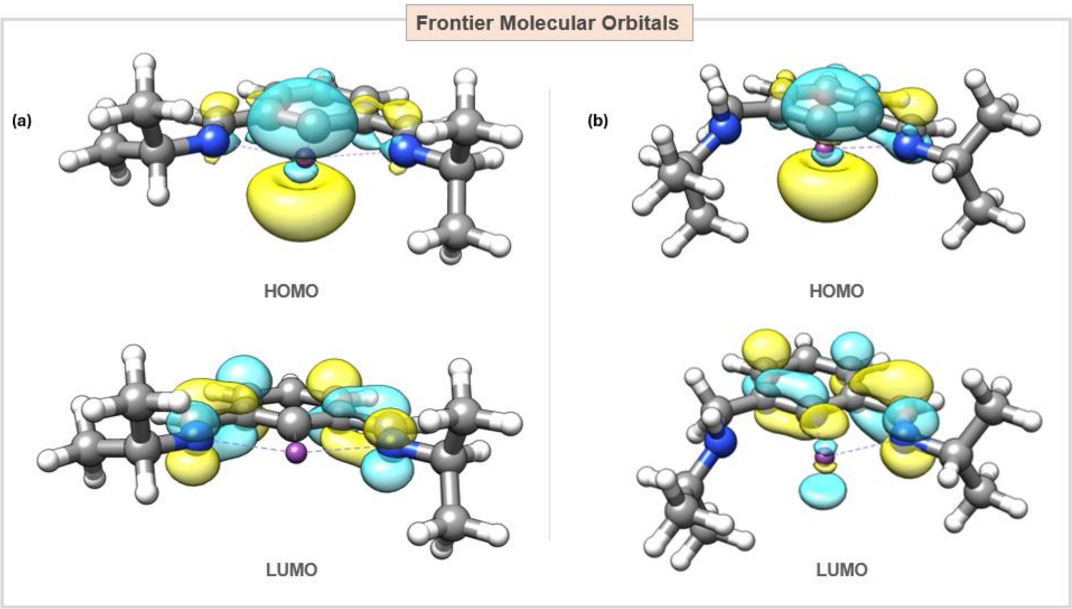

Figure S230. Frontier Molecular Orbitals (FMOs) showing HOMO-LUMO structure of **[(L^1)BiCl$_2$] (1) and [(L^2)BiCl$_2$] (2)**

Table S34. HOMO-LUMO gap of Bi(I) and Bi(III) species for catalyst **[(L^1)BiCl$_2$] (1)** and **[(L^2)BiCl$_2$] (2)**

**Species**	**HOMO (eV)**	**LUMO (eV)**	**Δ(HOMO-LUMO) (eV)**
**[(L^2)BiCl$_2$] (2′)**	−4.59	−1.61	2.98
**[(L^2)BiCl$_2$] (2)**	−6.79	−1.87	4.92
**[(L^1)BiCl$_2$] (1′)**	−4.42	−1.29	3.14
**[(L^1)BiCl$_2$] (1)**	−6.81	−1.79	5.01

3. Intrinsic Bonding Orbital (IBO) Analysis: (i) Int2→TS23→Int3, and (ii) Int4→TS45→Int5

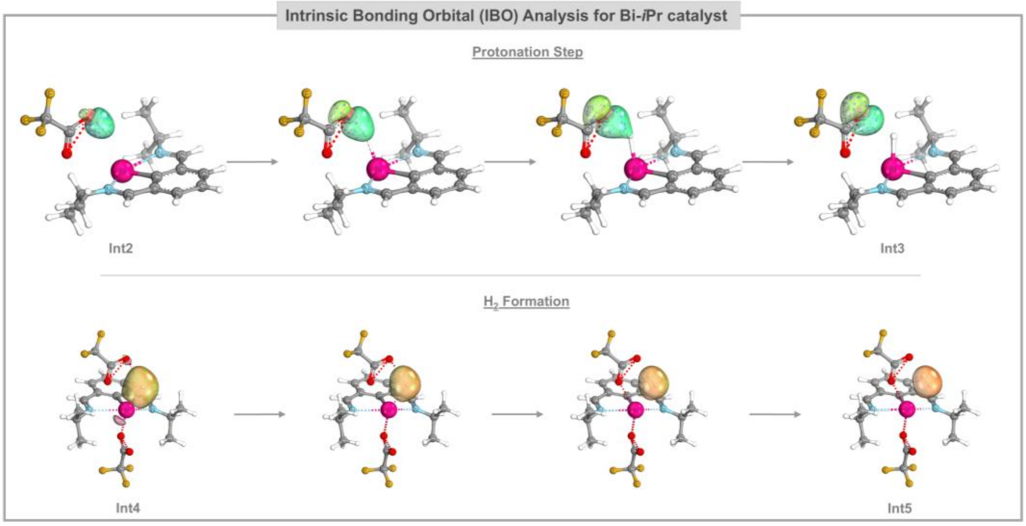

Figure S231. Intrinsic Bonding Orbital (IBO) analyses showing protonation and H_2 formation step for **[(L^2)Bi] (2′).**

Intrinsic bonding orbital analysis for H_2 formation (2nd step) for [$(L^1)BiCl_2$] (1):

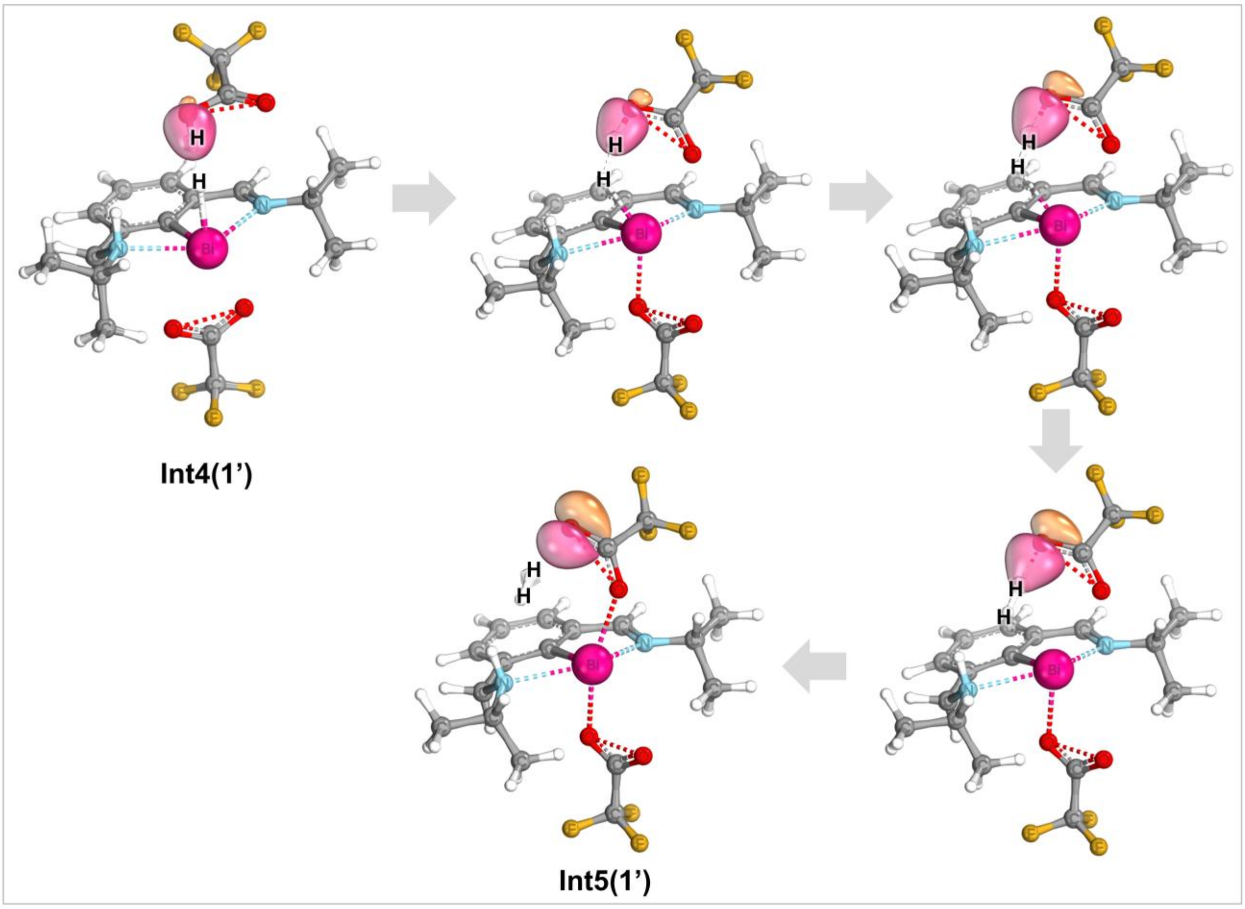

Figure S232. Intrinsic bonding orbital analysis for σ(O−H) bonding orbital along the Int4(1)→TS45(1)→Int5(1) conversion.

4. Electronic Energies and Gibbs Energies

Total electronic energies and Gibbs free energies (298.15 K) of the optimized structures computed at the SMD (acetonitrile)-PBE0-D3BJ/def2-TZVP// PBE0-D3BJ/def2-SVP level of theory are provided below.

i. [(L^1)Bi] (1′)

Table S35. Total electronic energies and Gibbs free energies (298.15 K) of the optimized structures.

Molecule	E_{tot}[au]	$G_{298.15}$[au]	Imaginary frequency
**Int1**	−868.598	−868.339	
**Int2**	−1395.13	−1394.84	
**TS23**	−1395.11	−1394.82	*i*377.55
**Int3**	−1395.12	−1394.84	
**Int4**	−1921.66	−1921.34	
**TS45**	−1921.64	−1921.33	*i*667.23
**Int5(Product)**	−1921.68	−1921.37	

ii. [(L^2)Bi] (2′)

Table S36. Total electronic energies and Gibbs free energies (298 K) of the optimized structures

Molecule	E_{tot}[au]	$G_{298.15}$[au]	Imaginary frequency
**Int1**	−869.802	−869.518	
**Int2**	−1396.33	−1396.02	
**TS23**	−1396.32	−1396.01	*i*294.94
**Int3**	−1396.34	−1396.03	
**Int4**	−1922.87	−1922.52	
**TS45**	−1922.85	−1922.51	*i798.41*
**Int5(Product)**	−1922.9	−1922.56	

5. **Lowdin plot and Comparison of Bond parameter of Experimental (via XRD) and theoretical Optimized structure**

Table S37. Löwdin charge (e) of Bi(I) and Bi(III) species for catalyst **[(L¹)BiCl₂] (1)** and **[(L²)BiCl₂] (2)**

Atom	Löwdin charge (e)	Löwdin charge (e)	Löwdin charge (e)	Löwdin charge (e)
	**$[(L^1)Bi]$ (1′) Bi (I)**	**$[(L^1)BiCl_2]$ (1) Bi (III)**	**$[(L^2)Bi]$ (2′) Bi (I)**	**$[(L^2)BiCl_2]$ (2) Bi (III)**
Bi	0.150	0.598	0.206	0.589
N1	−0.092	−0.067	−0.087	−0.047
N2	−0.053	−0.036	−0.079	−0.054
C1	−0.178	−0.151	−0.180	−0.121
C2	−0.057	−0.039	−0.081	−0.065
C6	−0.084	−0.067	−0.083	−0.066
C7	0.026	0.88	0.039	0.93
C8	0.029	0.32	0.043	0.88
Cl1	-	−0.386	-	−0.383
Cl2	-	−0.362	-	−0.389

Catalyst [(L^2)BiCl$_2$] (2)

Table S38. Comparison of Bond length of Catalyst **[(L^2)BiCl$_2$] (2)** Bi(III) species crystallographic vs optimized structure.

**Atoms**	**Bond Length in Å**<br><br>**Experimental**	**Bond Length in Å**<br><br>**Optimized Structure (DFT)**
**Bi(1)-Cl(1)**	2.69(11)	2.64
**Bi(1)-Cl(2)**	2.66(11)	2.63
**Bi(1)-N(2)**	2.49(3)	2.52
**Bi(1)-N(1)**	2.47(3)	2.51
**Bi(1)-C(1)**	2.20(3)	2.23
**N(1)-C(7)**	1.27(4)	1.27
**N(2)-C(8)**	1.28(4)	1.27
**N(2)-C(10)**	1.46(4)	1.45
**N(1)-C(9)**	1.47(4)	1.45
**C(1)-C(2)**	1.38(5)	1.39
**C(1)- C(6)**	1.38(5)	1.39
**C(7)- C(6)**	1.46(5)	1.46
**C(8)- C(2)**	1.47(5)	1.46

Catalyst 1 [(L[1])BiCl$_2$] (1)

Table S39. Comparison of Bond length of Catalyst **[(L^1)BiCl$_2$] (1)** Bi(III) species crystallographic vs optimized structure.

**Atoms**	**Bond Length in Å**<br><br>**Experimental**	**Bond Length in Å**<br><br>**Optimized Structure (DFT)**
**Bi(1)-Cl(2)**	2.783(5)	2.68
**Bi(1)-Cl(1)**	2.673(5)	2.63
**Bi(1)-N(2)**	2.468(18)	2.51
**Bi(1)-N(1)**	2.56(2)	2.53
**Bi(1)-C(1)**	2.172(18)	2.23
**N(1)-C(7)**	1.22(3)	1.27
**N(2)-C(8)**	1.48(3)	1.46
**N(1)-C(9)**	1.53(3)	1.45
**N(2)-C(10)**	1.55(3)	1.47
**C(2)-C(1)**	1.37(3)	1.39
**C(2)- C(8)**	1.46(3)	1.50
**C(1)- C(6)**	1.45(3)	1.39
**C(7)- C(6)**	1.43(3)	1.46

Optimized structure bond parameter of catalyst [(L^1)Bi] (1′) and [(L^2)Bi] (2′) Bi(I) species

Table S40. Comparison of Bond length of **[(L^1)Bi] (1′) Bi**(I) species and catalyst **[(L^2)Bi] (2′)** Bi(I) species as derived from DFT calculated optimized structure.

**Atoms**	**Bond Length of Optimized Structure (Å) [(L^1)BiCl$_2$] (1′)**	**Bond Length of Optimized Structure (Å) [(L^2)BiCl$_2$] (2′)**
**Bi(1)-N(2)**	2.72	2.52
**Bi(1)-N(1)**	2.30	2.41
**Bi(1)-C(1)**	2.18	2.15
**N(1)-C(7)**	1.30	1.3
**N(2)-C(8)**	1.46	1.29
**N(1)-C(9)**	1.46	1.46
**N(2)-C(10)**	1.46	1.46
**C(2)-C(1)**	1.40	1.41
**C(2)- C(8)**	1.51	1.45
**C(1)- C(6)**	1.42	1.41
**C(7)- C(6)**	1.43	1.44

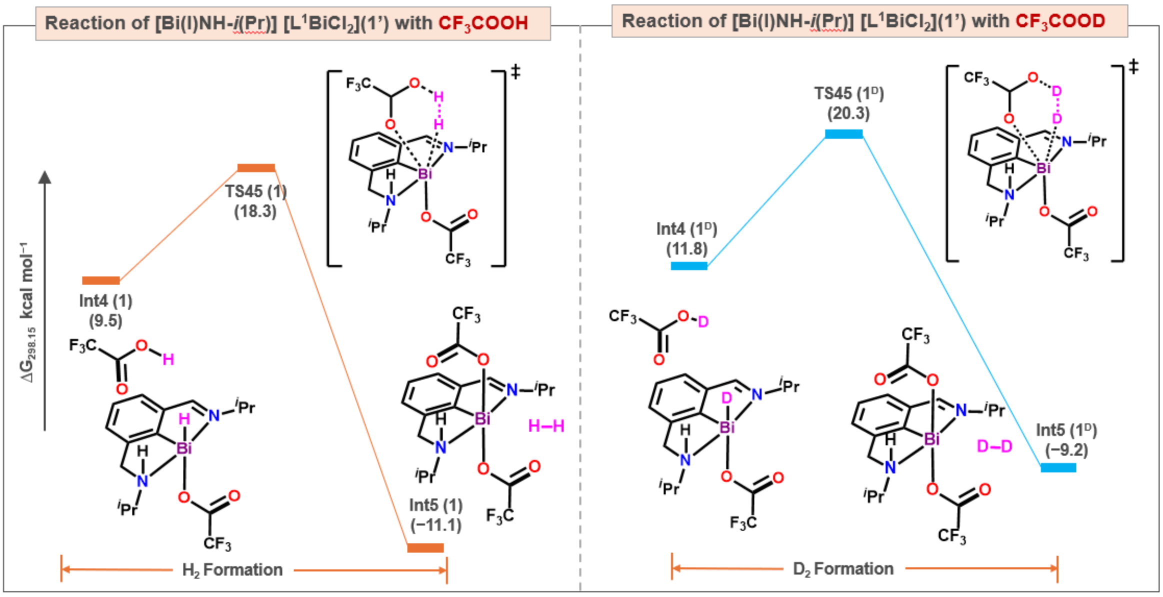

Figure S233. Computed Gibbs free-energy profiles (kcal mol^{-1}) comparing the hydrogenated and deuterated pathways for the hydrogen evolution step using trifluoroacetic acid (TFA) and deuterated TFA (TFA-d) as the proton sources for catalyst [Bi(I)NH−*i*Pr] (**1′**).

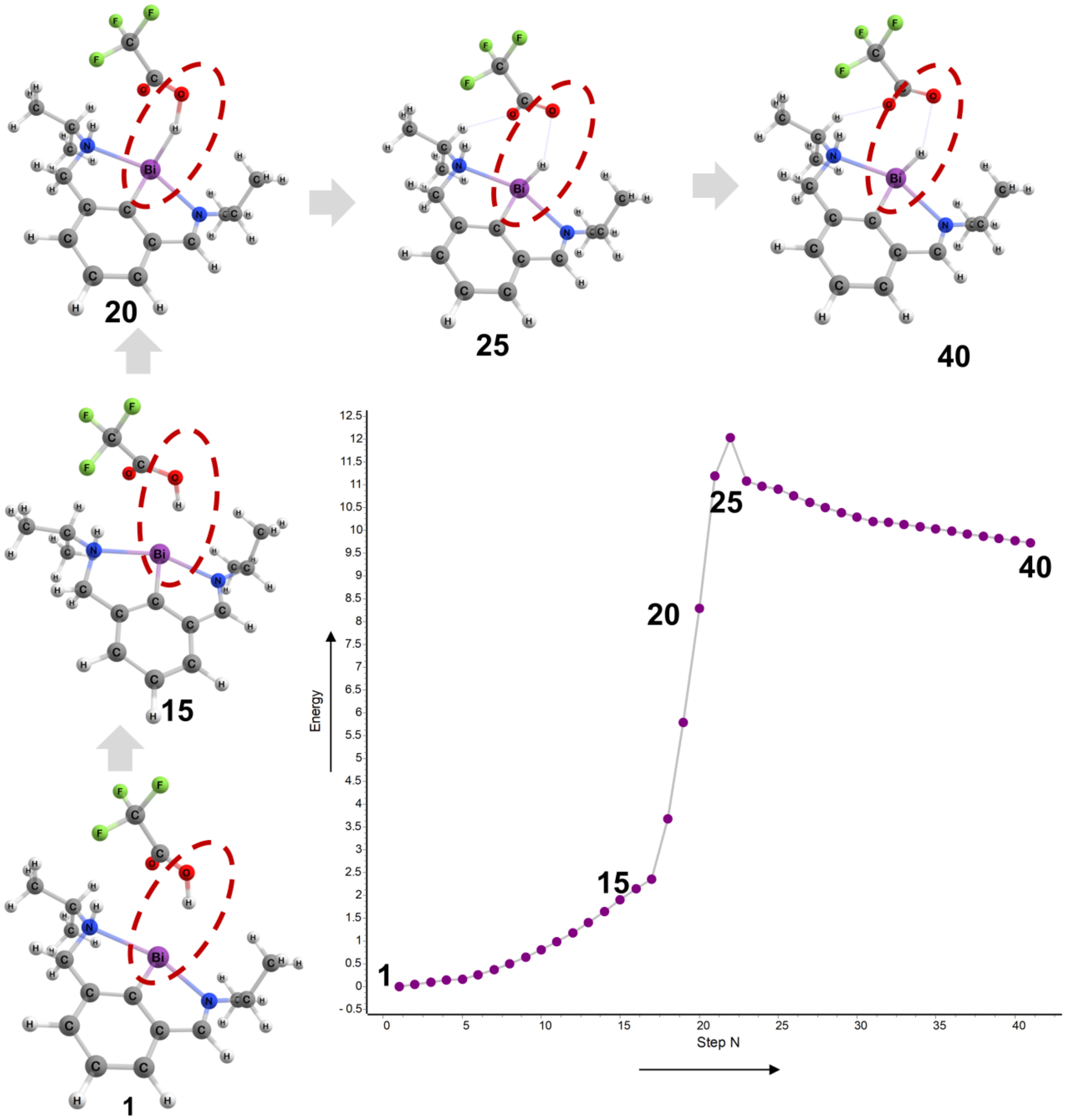

Figure S234. Intrinsic reaction coordinate (IRC) calculations performed for the transition state **TS23(1’)**, demonstrating its connectivity to the corresponding forward **Int3(1’)** and backward Int**2(1’)** intermediates.

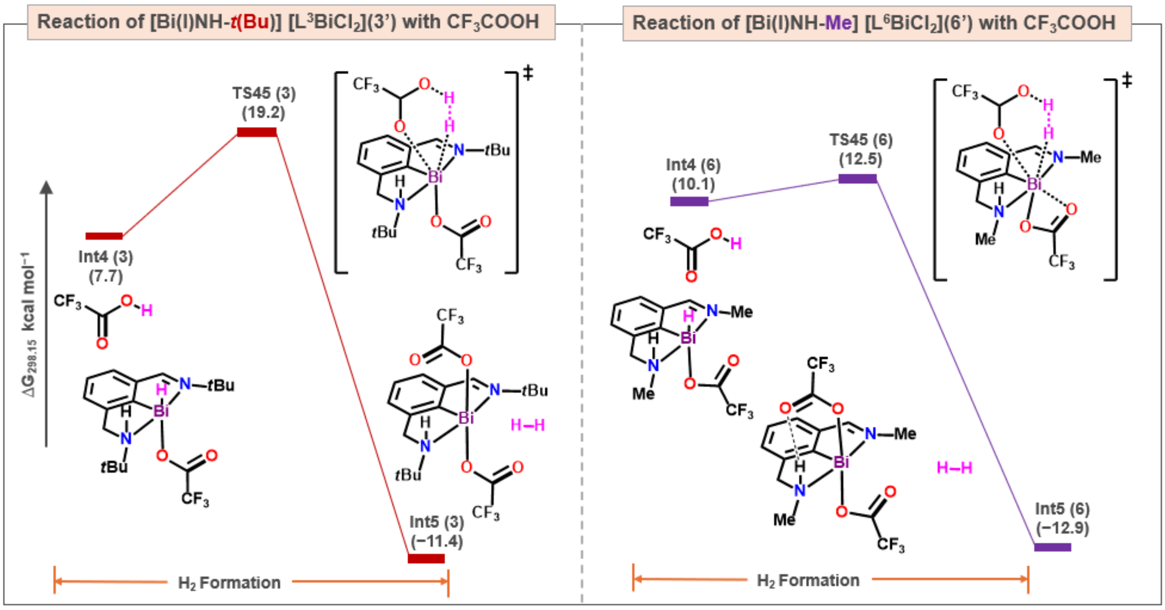

Figure S235. Computed Gibbs free energy profile for the hydrogen evolution step (in kcal mol^{-1}) catalyzed by [Bi(I)NH−*t*Bu] (**3′**) and [Bi(I)NH−Me] (**6′**) in the presence of trifluoroacetic acid (TFA).[28,29]

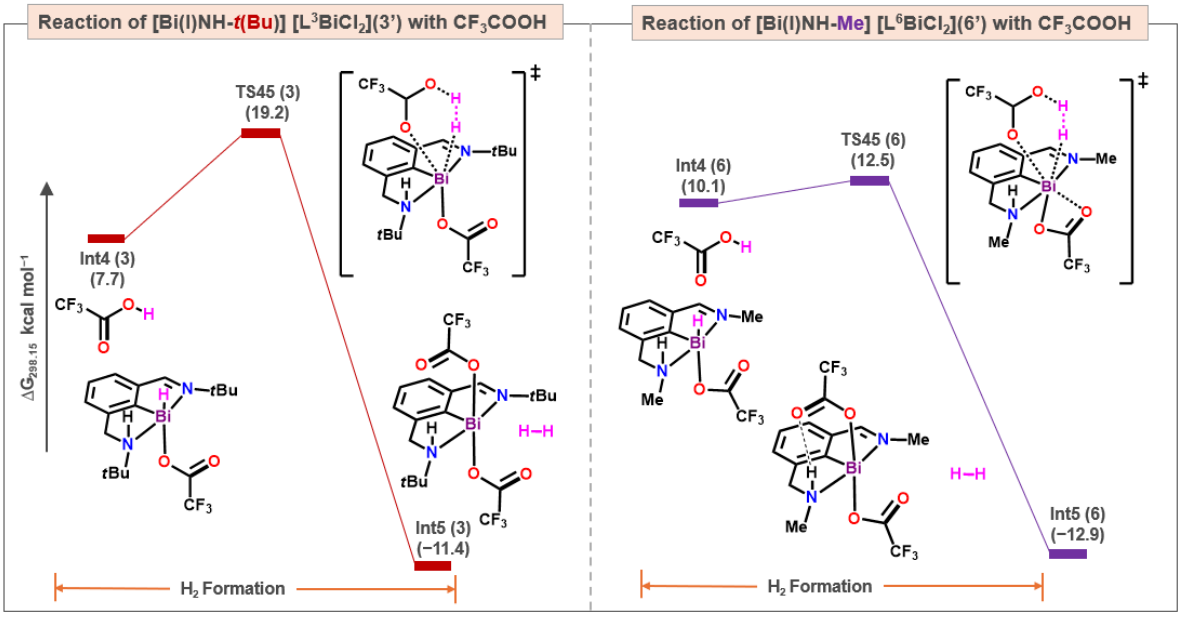

Figure S236. Computed Gibbs free energy profile for the hydrogen evolution step (in kcal mol^{-1}) catalyzed by [Bi(I)NH−*t*Bu] (**3′**) and [Bi(I)NH−Me] (**6′**) in the presence of trifluoroacetic acid (TFA).

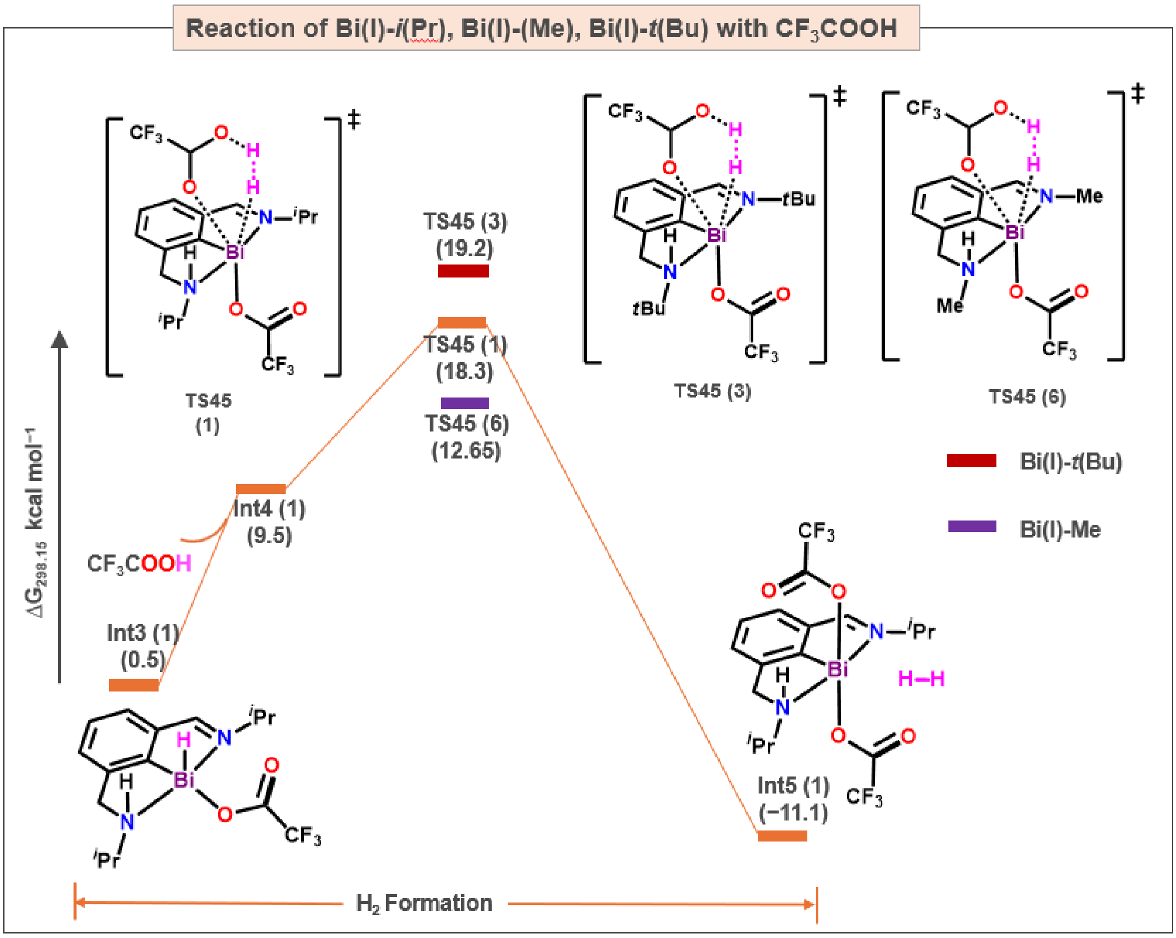

Figure S237. Computed Gibbs free energy profile for the hydrogen evolution step (in kcal mol^{-1}) catalyzed by [Bi(I)NH−*i*Pr] (**1′**), [Bi(I)NH−*t*Bu] (**3′**) and [Bi(I)NH−Me] (**6′**) in the presence of trifluoroacetic acid (TFA).

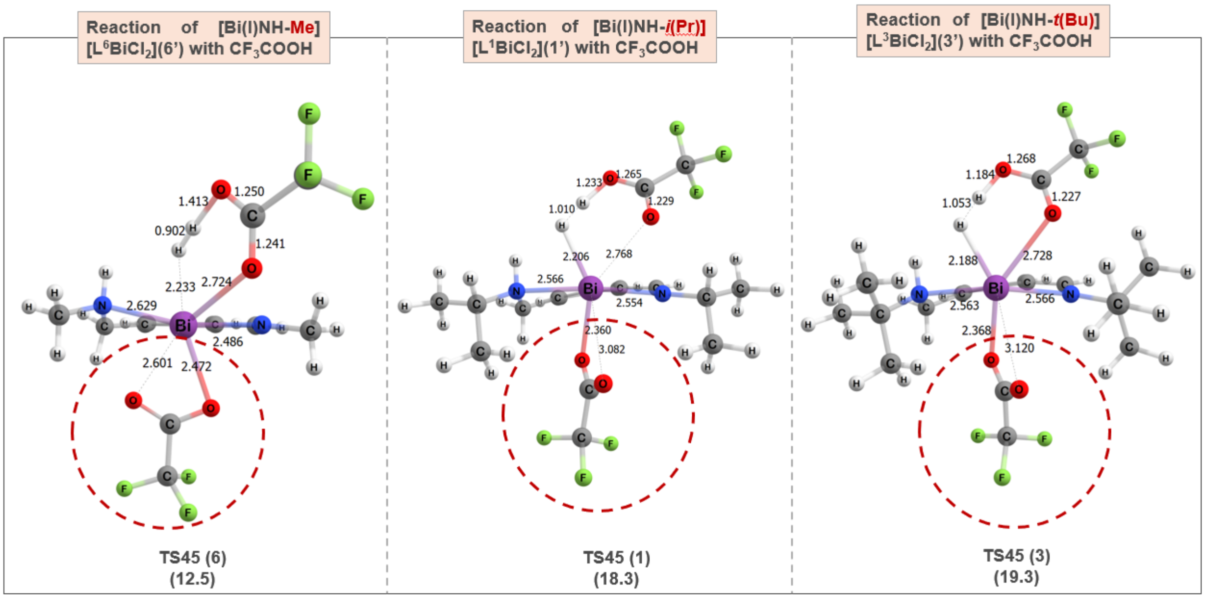

Figure S238. Comparison of transition-state structures showing TFA coordination and the steric influence of Me, *i*Pr and *t*Bu substituents.

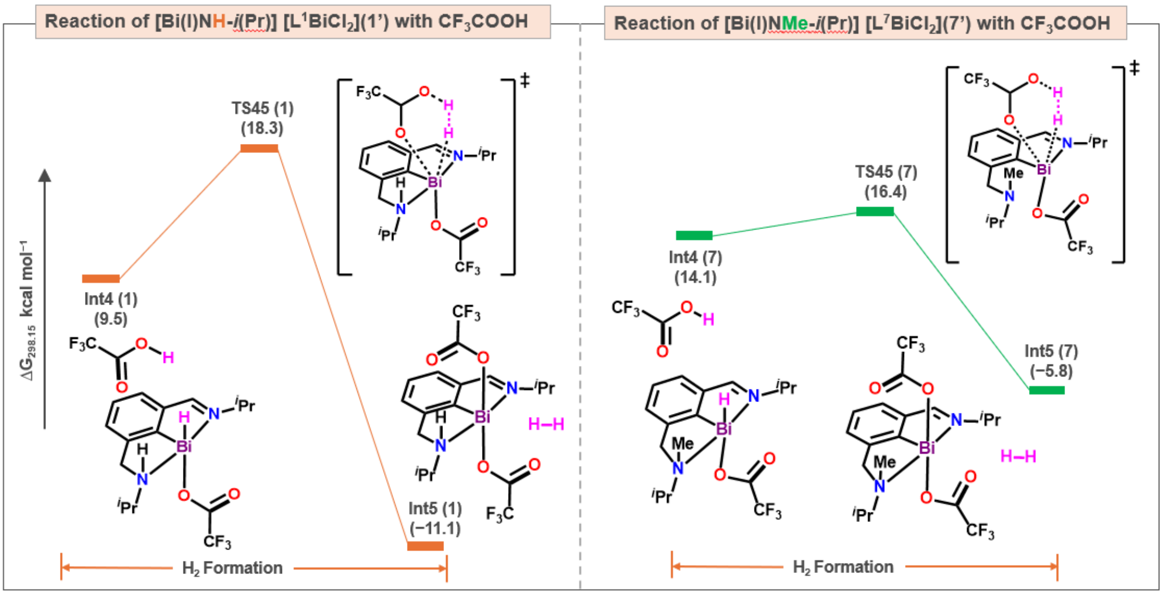

Figure S239. Computed Gibbs free energy profile for the hydrogen evolution step (in kcal mol^{-1}) catalyzed by [Bi(I)NH−*i*(Pr)] (**1′**) and [Bi(I)NMe−*i*(Pr)] (**7′**) in the presence of trifluoroacetic acid (TFA).

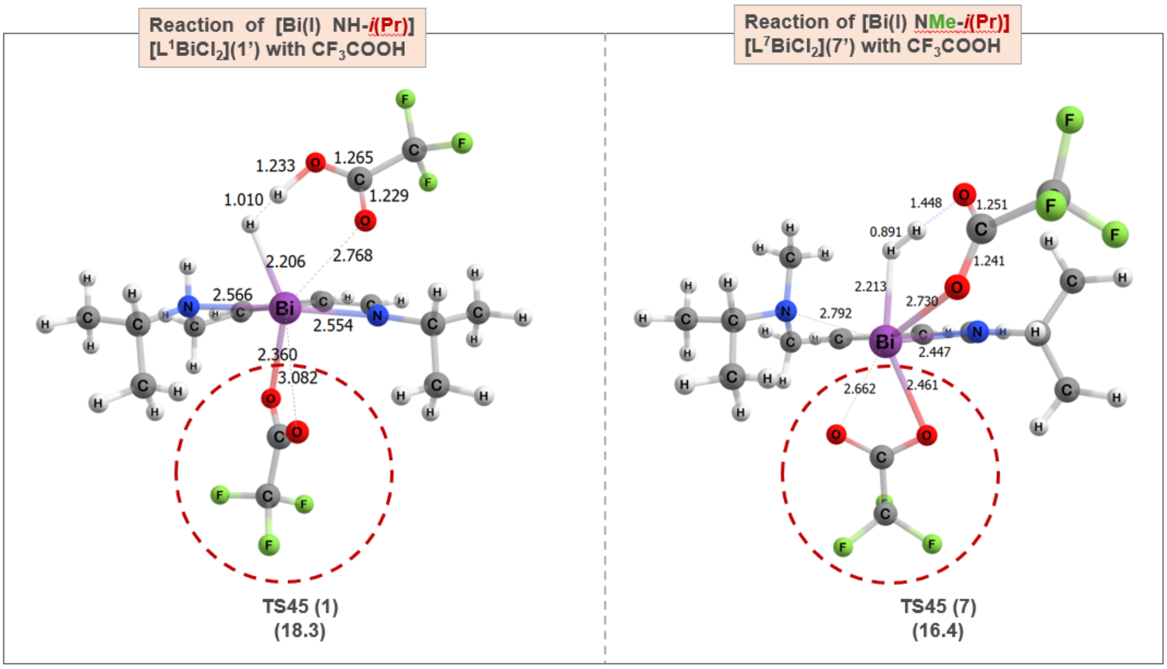

Figure S240. Comparative geometrical analysis of the transition states for [Bi(I)NH−*i*(Pr)] (**1′**) and [Bi(I)NMe−*i*(Pr)] (**7′**).

Further, non-covalent interactions (NCIs) analysis was performed using the Multiwfn program,[30] and NCI iso-surfaces were generated using the VMD 1.9.3 program.[31] NCI analysis of TS45(**1**) from [Bi(I)NH−*i*(Pr)] (**1′**) shows attractive interaction (shown in green, Figure S241) between the pendant *N−H* hydrogen and Bi−H hydrogen atoms in the hydrogen elimination step. These results are suggestive of an additional stabilizing interaction in TS45(**1**) having the pendant -*NH* arm, which likely contributes to the experimentally observed enhanced catalytic performance for hydrogen evolution for [Bi(I)NH−*i*(Pr)] (**1′**).

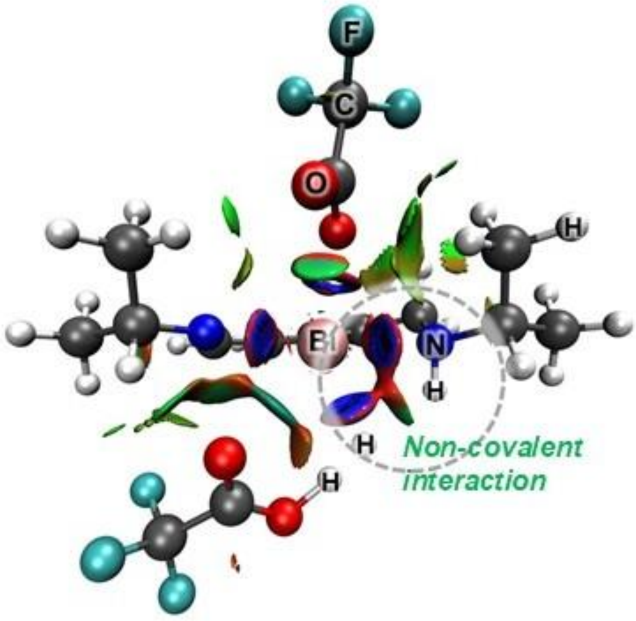

Figure S241. Non-covalent interaction (NCI) plot of TS45(**1**) derived from [Bi(I)NH−*i*(Pr)] (**1′**) showing important non-covalent interaction (in green color) between Bi-H and -*NH* pendant hydrogen in the hydrogen evolution step.

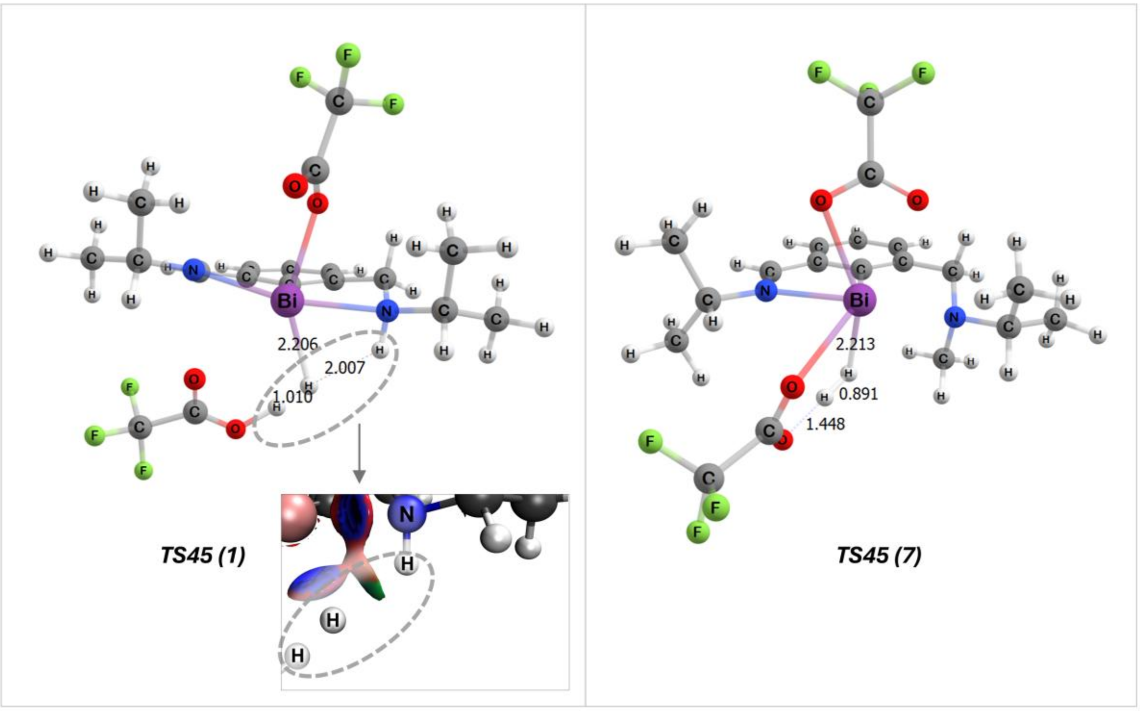

Figure S242. Comparison of the geometrical structures of the rate-determining transition states **TS45(1)** and **TS45(7)**. Distances shown in the structures are in Å. Only the relevant

fragment involved in the H_2-formation step is shown to emphasize the important non-covalent interactions (in green color) computed from NCI analysis.

6. Cartesian Coordinates of Optimized Structures

DFT optimization method: PBE0-D3BJ/def2-SVP

(i) Optimized cartesian coordinates of [(L[1])Bi] (1′)

Int1

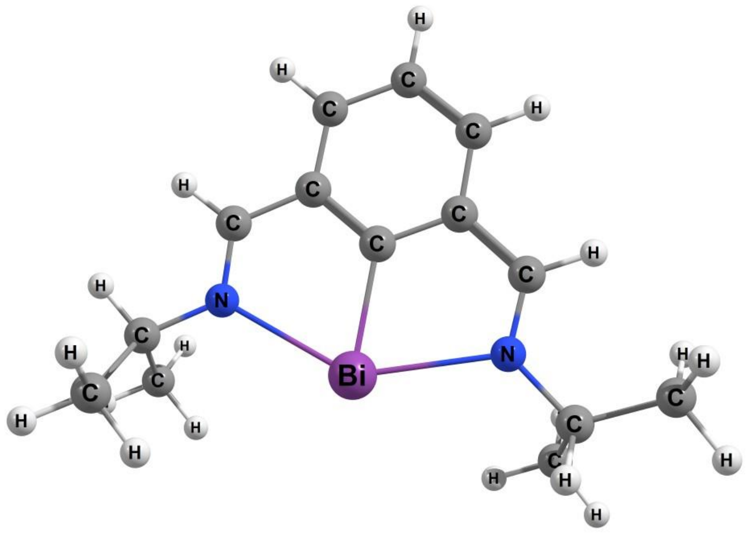

Bi	0.568877850	−0.157956776	0.822799835
N	2.786438264	0.779314198	0.240266402
N	−1.864938960	0.170360264	0.496594150
C	−1.215667897	3.123201996	−1.558565234
H	−2.228639835	3.440961219	−1.825408994
C	−1.033455377	1.994565290	−0.751024088
C	−2.124139060	1.200537003	−0.239583858
H	−3.153787812	1.489130414	−0.494194664
C	1.386293542	2.315261189	−0.885119276
C	−0.114116267	3.844709870	−2.026623059
H	−0.265890905	4.723243474	−2.656842165
C	0.274581542	1.583519347	−0.406385808

C	1.179965937	3.442181147	−1.691294090
H	2.040153621	4.008588053	−2.061887451
C	2.688500245	1.834459377	−0.501346200
H	3.592018062	2.367051600	−0.841627065
C	−2.904905962	−0.670333603	1.072727904
C	−4.303830762	−0.455602249	0.512622654
H	−4.702743341	0.536504516	0.777414026
H	−4.987931392	−1.206356555	0.934087774
H	−4.325294131	−0.558841701	−0.582815914
C	−2.883189963	−0.516972627	2.591986895
H	−1.877389476	−0.709563429	2.992330907
H	−3.588181539	−1.218100278	3.063916954
H	−3.167040254	0.508334258	2.876992115
C	4.090902934	0.288949342	0.643950140
C	4.287802274	−1.136688537	0.143568284
H	4.178943973	−1.187685707	−0.949397108
H	3.538307253	−1.810079292	0.588329708
H	5.288128726	−1.507520505	0.414146934
C	4.233270538	0.383953158	2.158523090
H	5.233125743	0.050874138	2.476245690
H	3.482704266	−0.248083442	2.658567995
H	4.083815388	1.418250132	2.500656196
H	4.868742059	0.929736370	0.182284723
H	−2.607241283	−1.708567654	0.839742598

Int2

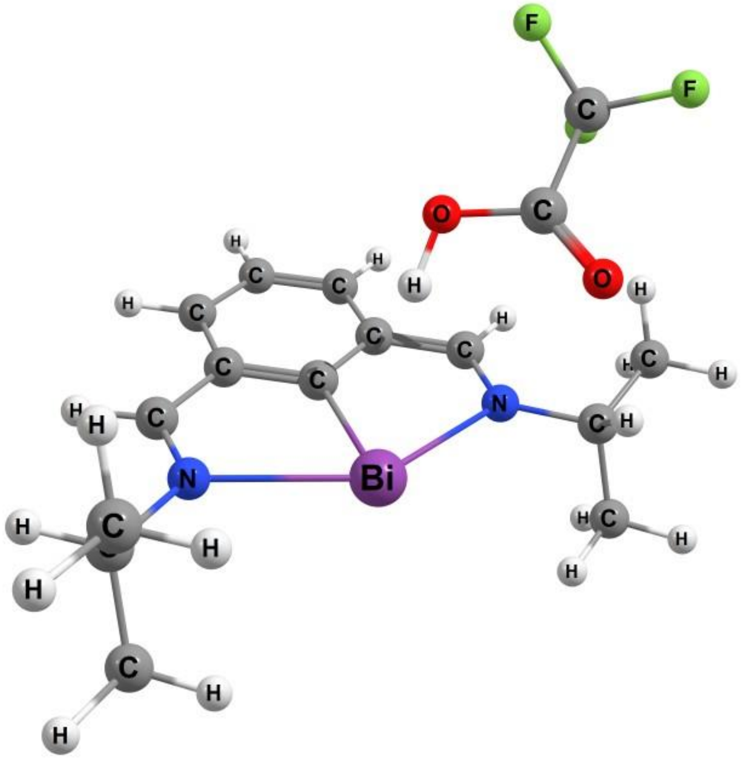

Bi	0.752525000	−0.326773000	1.165099000
N	2.886692000	0.648187000	0.338240000
N	−1.697187000	−0.247404000	0.839139000
C	−1.314888000	2.312022000	−1.745065000
H	−2.352089000	2.487293000	−2.046648000
C	−1.032491000	1.376553000	−0.742591000
C	−2.048464000	0.597108000	−0.070492000
H	−3.099426000	0.730731000	−0.362452000
C	1.348282000	1.855250000	−0.993029000
C	−0.281065000	3.019720000	−2.362626000
H	−0.510160000	3.748838000	−3.142144000
C	0.305730000	1.147250000	−0.357854000

C	1.044447000	2.791959000	−1.989985000
H	1.851946000	3.344087000	−2.480665000
C	2.692304000	1.547343000	−0.566377000
H	3.544546000	2.079418000	−1.021210000
C	−2.651395000	−1.065750000	1.578854000
C	−4.046164000	−1.130045000	0.975733000
H	−4.567044000	−0.160709000	1.031663000
H	−4.650656000	−1.860111000	1.532704000
H	−4.018904000	−1.452619000	−0.075296000
C	−2.682739000	−0.597821000	3.030860000
H	−1.673513000	−0.591393000	3.467979000
H	−3.317609000	−1.263129000	3.634826000
H	−3.086489000	0.424734000	3.099053000
C	4.231958000	0.326494000	0.777988000
C	4.535355000	−1.136412000	0.479217000
H	4.423242000	−1.349565000	−0.593735000
H	3.845669000	−1.796373000	1.028671000
H	5.563871000	−1.387272000	0.779564000
C	4.381098000	0.647987000	2.260141000
H	5.408121000	0.442915000	2.598001000
H	3.691484000	0.035250000	2.861741000
H	4.152720000	1.705313000	2.457902000
C	−1.391832000	−2.738230000	−1.431871000
O	−0.534908000	−1.834776000	−1.839933000
O	−1.443448000	−3.276012000	−0.361657000
H	0.031790000	−1.542773000	−1.075755000
C	−2.440767000	−3.022123000	−2.525771000
H	4.949363000	0.953929000	0.212245000

H	−2.232069000	−2.085282000	1.553884000
F	−1.890729000	−3.125287000	−3.728832000
F	−3.324202000	−2.015755000	−2.557454000
F	−3.098095000	−4.137206000	−2.258492000

TS23

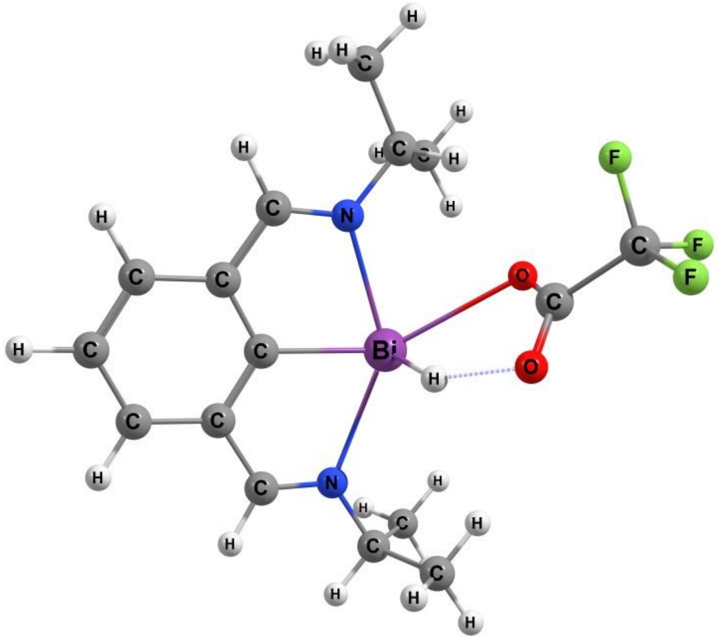

Bi	0.439692365	-0.308078298	0.734277088
N	2.719843992	0.700971086	0.148574742
N	-1.995746474	0.121811746	0.340337594
C	-1.273762590	3.349056994	-1.244199310
H	-2.281864324	3.713445681	-1.463590641
C	-1.095895721	2.118727711	-0.596137328
C	-2.211711614	1.257080326	-0.206291032
H	-3.233225132	1.612767703	-0.406042336
C	1.305256259	2.410216444	-0.701367580
C	-0.162068338	4.105673910	-1.617019835
H	-0.300533527	5.062196091	-2.124950898
C	0.191190900	1.655170944	-0.316727719

C	1.125862354	3.638897786	-1.353863295
H	1.994172494	4.229087039	-1.661582121
C	2.620290220	1.834265140	-0.433788060
H	3.521310289	2.380469417	-0.765651568
C	-3.061779769	-0.781337691	0.762023509
C	-4.459872623	-0.356902474	0.346805368
H	-4.778125087	0.573387162	0.845782392
H	-5.171812961	-1.143277742	0.634135029
H	-4.544491581	-0.219186043	-0.742135669
C	-2.950679604	-0.999114751	2.267241555
H	-1.974080035	-1.433085298	2.520485114
H	-3.723461397	-1.707736995	2.598207080
H	-3.085762608	-0.053125019	2.815881363
C	4.007117269	0.066864001	0.359634441
C	4.082110138	-1.194234620	-0.494820416
H	3.984740545	-0.952084426	-1.562936013
H	3.271875840	-1.895687197	-0.241009500
H	5.044009498	-1.704897262	-0.339689641
C	4.190512446	-0.232116517	1.841441931
H	5.177522642	-0.682739003	2.021523855
H	3.424110507	-0.940412316	2.193799709
H	4.111505462	0.684615732	2.443876659
C	-0.500120482	-3.271989954	-0.470792663
O	0.343395459	-2.894320190	-1.305006397
O	-0.798557219	-2.772276617	0.631659081
H	0.623446307	-1.360205607	-0.769775846
C	-1.342626562	-4.513882139	-0.856094481
H	4.807904095	0.761909768	0.036802129

H	-2.816100415	-1.747137276	0.292231627
F	-0.999441692	-5.027642912	-2.031029078
F	-2.642345636	-4.177710988	-0.926936109
F	-1.228454690	-5.474620344	0.063147270

Int3

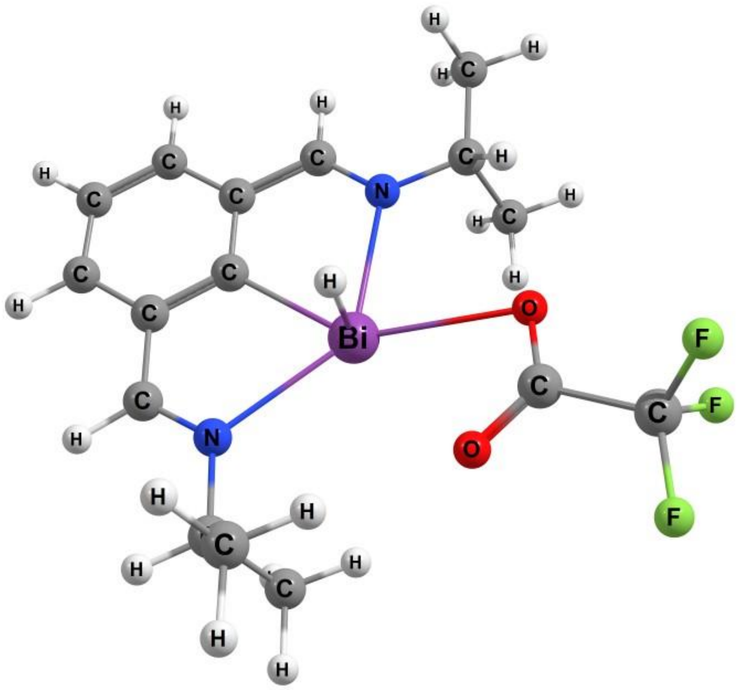

Bi	0.213260711	-0.390042501	0.384798948
N	2.539437063	0.550054893	-0.108124004
N	-2.186720596	0.345111793	0.131519998
C	-1.223141638	3.761422327	-0.816210255
H	-2.196842709	4.243916772	-0.945477745
C	-1.141672654	2.418535542	-0.417823355
C	-2.320930720	1.574915854	-0.187754577
H	-3.315903362	2.029418662	-0.314705003
C	1.271332457	2.529147316	-0.520407862
C	-0.052347036	4.482145891	-1.058040503
H	-0.113498662	5.526865323	-1.369889279

C	0.103191334	1.810412924	-0.261000558
C	1.195780227	3.873199772	-0.916686891
H	2.106640587	4.442535979	-1.125611102
C	2.534378533	1.793451923	-0.399348308
H	3.483433323	2.329889857	-0.580258566
C	-3.299809855	-0.562280287	0.380403717
C	-4.661895392	-0.033851316	-0.036046552
H	-4.981616807	0.822640654	0.579740364
H	-5.411832062	-0.826915396	0.093736185
H	-4.678362742	0.269904440	-1.094134593
C	-3.266143994	-0.981048779	1.847467521
H	-2.315616366	-1.479663884	2.081121381
H	-4.071736147	-1.701815023	2.048300359
H	-3.403041130	-0.110421084	2.508685987
C	3.774710018	-0.209032138	-0.015314465
C	3.819060494	-1.234353779	-1.143020290
H	3.736259511	-0.746116666	-2.125276903
H	3.000128980	-1.962114603	-1.031499602
H	4.772206926	-1.782800141	-1.108389101
C	3.852801732	-0.883600459	1.348825471
H	4.815986980	-1.404042988	1.455118179
H	3.052811785	-1.634409165	1.448064556
H	3.766615248	-0.149615746	2.164020750
C	0.018320738	-3.390980486	0.276961302
O	1.180688181	-2.951658483	0.252146759
O	-1.045546456	-2.736739061	0.311429495
H	0.181694721	-0.750509839	-1.364338112
C	-0.154911504	-4.927951860	0.277257828

H	4.626827263	0.491397166	-0.125510886
H	-3.058903159	-1.465052253	-0.205742024
F	1.004802077	-5.571006418	0.357751809
F	-0.764511757	-5.326196470	-0.845189312
F	-0.911526492	-5.317168514	1.307464104

Int4

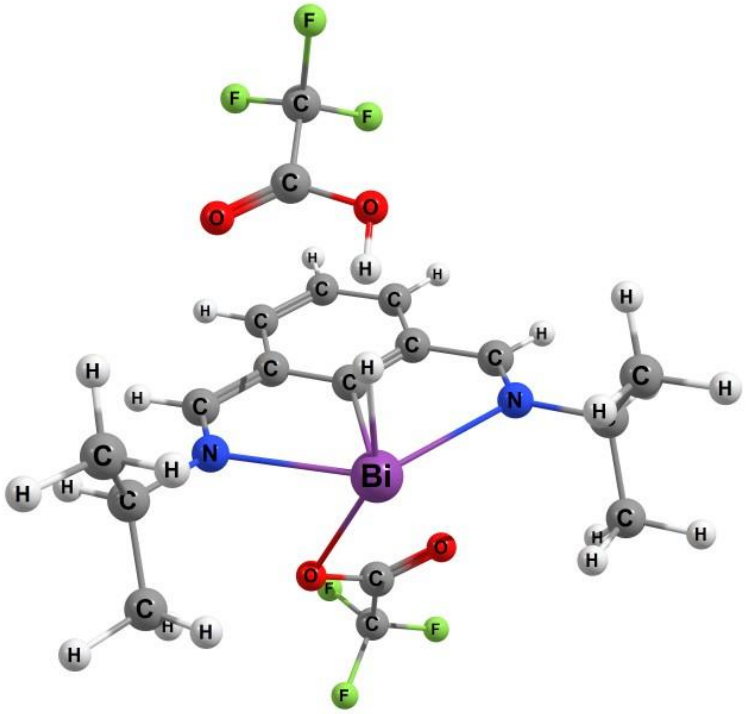

Bi	0.324648000	-1.516352000	-1.147266000
N	2.549526000	-0.510949000	-0.472475000
N	-2.098194000	-1.303044000	-0.589827000
C	-1.538739000	2.019267000	0.847430000
H	-2.556945000	2.328009000	1.098118000
C	-1.314319000	0.798172000	0.196859000
C	-2.379344000	-0.153848000	-0.118447000
H	-3.424730000	0.132502000	0.080914000
C	1.073401000	1.202617000	0.261570000
C	-0.456307000	2.825238000	1.198214000

H	-0.629926000	3.771316000	1.714393000
C	-0.012977000	0.412879000	-0.104106000
C	0.848945000	2.421824000	0.913780000
H	1.693247000	3.048044000	1.214341000
C	2.407439000	0.668187000	-0.009893000
H	3.286386000	1.298978000	0.206071000
C	-3.136032000	-2.272320000	-0.888152000
C	-3.009238000	-3.439272000	0.082662000
H	-2.043419000	-3.951719000	-0.052172000
H	-3.812311000	-4.170720000	-0.090976000
H	-3.061103000	-3.089520000	1.123870000
C	-3.030395000	-2.700431000	-2.345420000
H	-3.002577000	-1.828226000	-3.012689000
H	-3.880570000	-3.344806000	-2.613073000
H	-2.106283000	-3.276893000	-2.515310000
C	3.862390000	-1.052431000	-0.768150000
C	3.956555000	-1.360799000	-2.257197000
H	3.671754000	-0.488261000	-2.860779000
H	3.275198000	-2.183295000	-2.527995000
H	4.979941000	-1.671451000	-2.514379000
C	4.102034000	-2.281407000	0.098809000
H	5.102185000	-2.696515000	-0.093988000
H	3.358753000	-3.063909000	-0.121595000
H	4.025967000	-2.033770000	1.167688000
C	-0.476773000	-0.535713000	3.169346000
O	0.666373000	-0.973063000	2.704472000
O	-1.580865000	-0.822726000	2.798015000
H	0.409778000	-2.287419000	0.627265000

C	-0.245256000	0.439250000	4.341573000
H	4.622961000	-0.287258000	-0.517049000
H	-4.118853000	-1.787121000	-0.731895000
F	-1.343374000	1.136096000	4.585548000
F	0.743566000	1.290924000	4.077517000
F	0.077247000	-0.242351000	5.440531000
O	-0.984292000	-0.255392000	-3.093930000
C	0.038540000	0.324523000	-3.510379000
O	1.185270000	0.254145000	-3.036153000
C	-0.185279000	1.238715000	-4.736704000
F	0.948864000	1.743810000	-5.210489000
F	-0.780366000	0.570296000	-5.727526000
F	-0.980451000	2.261235000	-4.404645000
H	0.512639000	-1.540955000	1.890895000

TS45

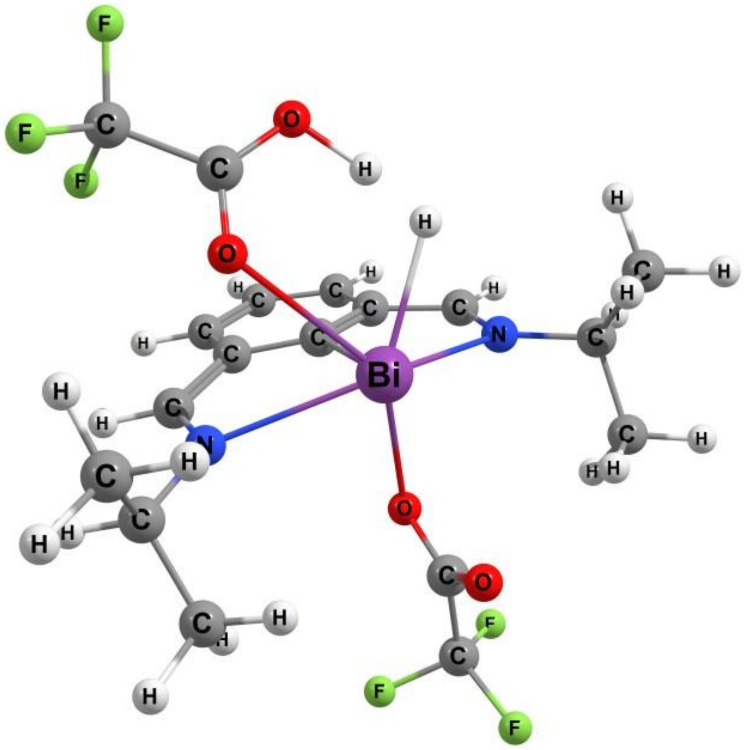

Bi	0.637783531	-1.371664675	-0.448464071
N	2.801598881	-0.139042081	-0.806499272
N	-1.887421764	-0.692639870	-0.319679685
C	-0.949896552	2.558524781	1.067034917
H	-1.930901267	2.966747534	1.325843242
C	-0.842761672	1.245779732	0.587709769
C	-2.020299196	0.428845430	0.261469248
H	-3.019569189	0.843385729	0.483830916
C	1.541678053	1.552994092	0.302329145
C	0.190197142	3.354232759	1.183762869
H	0.102794271	4.375781811	1.558943915
C	0.414167356	0.733885693	0.275137297
C	1.431389573	2.868954092	0.775859613
H	2.311497263	3.517365004	0.807497086
C	2.775762326	1.021069011	-0.278107879
H	3.676764041	1.658459882	-0.280002787
C	-3.032243575	-1.477329992	-0.736934453
C	-3.094278259	-2.774112988	0.060581251
H	-2.206247429	-3.394142479	-0.136294046
H	-3.984526796	-3.349215960	-0.233884329
H	-3.126939113	-2.580605928	1.140771974
C	-2.926637954	-1.733041007	-2.235925823
H	-2.825760149	-0.793234630	-2.797114209
H	-3.822585764	-2.261573283	-2.593616133
H	-2.048575090	-2.356485705	-2.467280099
C	4.001866888	-0.663377870	-1.433069574
C	3.713775984	-0.964664457	-2.898462789
H	3.313869490	-0.081151657	-3.416014057

H	2.975875145	-1.775734970	-2.999122198
H	4.637838485	-1.280705349	-3.404519046
C	4.479637263	-1.897795286	-0.678941399
H	5.408013126	-2.277220691	-1.130587493
H	3.728350560	-2.701548982	-0.726192880
H	4.667264799	-1.672297525	0.380267607
C	-0.218048042	-1.473028108	2.876596344
O	0.987213392	-1.183528996	3.147392242
O	-0.659942280	-1.936709814	1.829927744
H	1.983456022	-1.571648183	1.184049981
C	-1.240584355	-1.111363864	3.971425523
H	4.790144659	0.112637092	-1.374943294
H	-3.953350130	-0.894630603	-0.535286725
F	-2.209835330	-2.017486660	4.032154145
F	-1.795810279	0.065937701	3.652576986
F	-0.690455557	-0.999306556	5.169377209
O	0.326329989	-0.208707875	-2.502932876
C	0.326412667	-1.056436112	-3.445881367
O	0.481108709	-2.268023711	-3.364914826
C	0.091921083	-0.408934661	-4.829307862
F	0.238339323	-1.277349505	-5.819736317
F	-1.145767461	0.094399356	-4.900893091
F	0.950161496	0.595431494	-5.031935216
H	1.575673005	-1.382133156	2.147186523

Int5(Product)

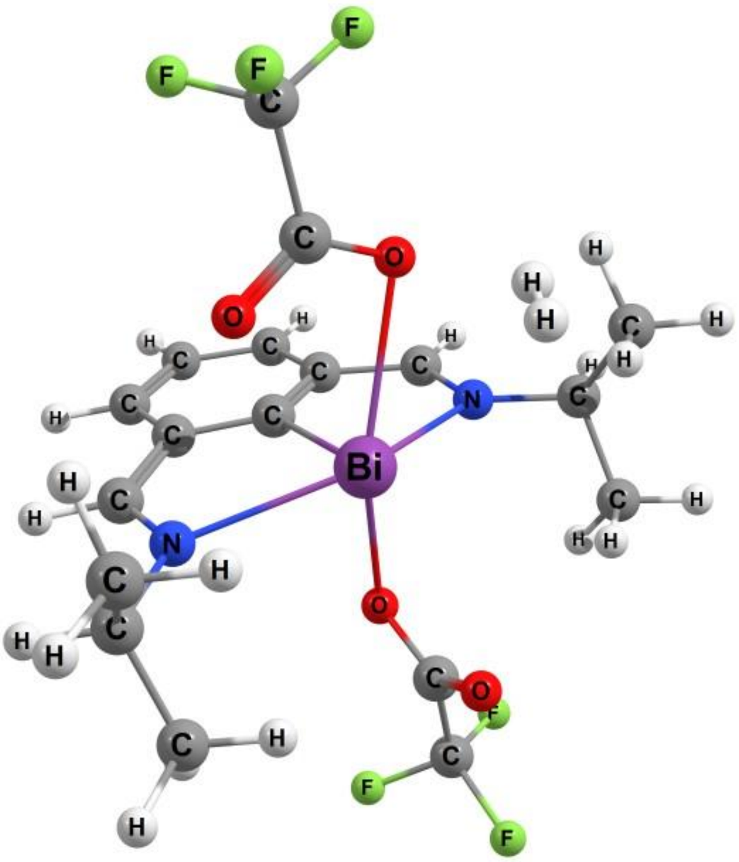

Bi	0.310440000	-1.065627000	-0.426309000
N	2.518419000	0.139167000	-0.607089000
N	-2.143659000	-0.510376000	-0.467745000
C	-1.439135000	2.927572000	0.597699000
H	-2.444936000	3.331108000	0.744590000
C	-1.261727000	1.582844000	0.243410000
C	-2.372339000	0.666744000	-0.039586000
H	-3.406719000	1.026893000	0.095404000
C	1.136819000	1.919346000	0.163184000
C	-0.325877000	3.755560000	0.747000000
H	-0.463602000	4.802450000	1.024632000
C	0.026188000	1.088200000	0.066639000
C	0.959707000	3.264684000	0.516339000
H	1.822981000	3.930906000	0.599890000

C	2.436638000	1.341883000	-0.197447000
H	3.335911000	1.978740000	-0.135615000
C	-3.209389000	-1.437803000	-0.795540000
C	-3.134967000	-2.642767000	0.133916000
H	-2.204577000	-3.207546000	-0.035690000
H	-3.979677000	-3.319461000	-0.061108000
H	-3.148039000	-2.336548000	1.188716000
C	-3.085735000	-1.834883000	-2.261798000
H	-3.094000000	-0.952050000	-2.917452000
H	-3.922221000	-2.490194000	-2.545783000
H	-2.147937000	-2.382843000	-2.445277000
C	3.780194000	-0.454852000	-1.006552000
C	3.665069000	-0.941439000	-2.445971000
H	3.348124000	-0.132875000	-3.119845000
H	2.928800000	-1.756892000	-2.529590000
H	4.635022000	-1.327667000	-2.791993000
C	4.140221000	-1.580760000	-0.044845000
H	5.129263000	-1.988274000	-0.300567000
H	3.408327000	-2.400487000	-0.112346000
H	4.149241000	-1.230097000	0.996025000
C	0.448441000	-1.274937000	2.457969000
O	1.514514000	-1.088707000	1.838251000
O	-0.683052000	-1.340332000	1.941463000
H	1.784886000	-3.666575000	1.515136000
C	0.519965000	-1.457435000	3.987921000
H	4.566017000	0.323864000	-0.951371000
H	-4.179450000	-0.926404000	-0.639584000
F	0.065930000	-2.664152000	4.327775000

F	-0.236183000	-0.544460000	4.597689000
F	1.759240000	-1.339187000	4.447046000
O	0.167604000	-0.190459000	-2.597126000
C	0.263085000	-1.142713000	-3.434995000
O	0.350098000	-2.338186000	-3.197134000
C	0.281520000	-0.646063000	-4.897716000
F	0.316750000	-1.647610000	-5.763596000
F	-0.800731000	0.090090000	-5.159644000
F	1.359319000	0.118393000	-5.110020000
H	1.456301000	-4.310511000	1.275434000

(ii) Optimized cartesian coordinates of Bi-NH-*i*Pr(I)

Int1

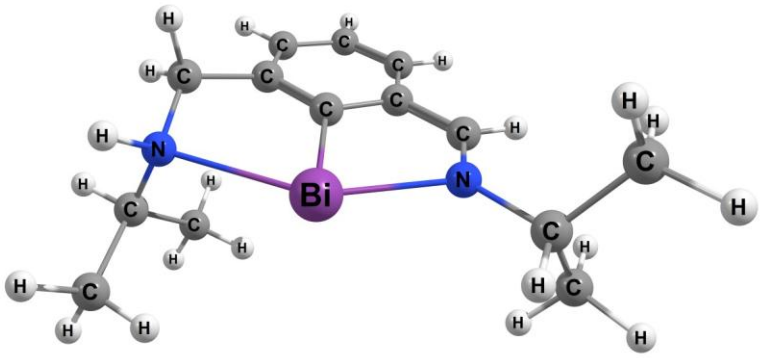

Bi	0.405273000	-0.740689000	0.051636000
N	2.919866000	0.028963000	-0.752182000
N	-1.705442000	0.043783000	0.559316000
C	-0.859823000	3.465963000	-0.476107000
H	-1.777625000	4.016694000	-0.249122000
C	-0.788414000	2.080113000	-0.202335000
C	-1.865458000	1.326501000	0.353837000
H	-2.811396000	1.824719000	0.607981000

C	1.485977000	2.032629000	-1.093651000
C	0.227016000	4.114552000	-1.027355000
H	0.180283000	5.186266000	-1.233414000
C	0.404419000	1.367315000	-0.489706000
C	1.399362000	3.394213000	-1.341721000
H	2.244127000	3.917053000	-1.801938000
C	2.665606000	1.214872000	-1.563351000
H	3.569473000	1.850042000	-1.643682000
C	-2.775082000	-0.770380000	1.129083000
C	-3.981299000	-0.831948000	0.198091000
H	-4.465812000	0.152768000	0.106903000
H	-4.730179000	-1.541368000	0.581600000
H	-3.675385000	-1.156561000	-0.807159000
C	-3.146685000	-0.313674000	2.535621000
H	-2.252806000	-0.270577000	3.174878000
H	-3.867727000	-1.010403000	2.989316000
H	-3.608275000	0.686091000	2.524029000
C	3.916018000	0.196633000	0.315164000
C	4.184831000	-1.155786000	0.953454000
H	4.508364000	-1.899421000	0.206999000
H	3.274632000	-1.534084000	1.446205000
H	4.977664000	-1.076791000	1.711222000
C	3.473663000	1.221480000	1.347036000
H	4.241140000	1.316847000	2.129394000
H	2.526257000	0.911751000	1.814118000
H	3.318389000	2.214431000	0.902395000
H	4.861955000	0.562145000	-0.141062000
H	-2.362946000	-1.790595000	1.208164000

H	3.234191000	-0.723400000	-1.359815000
H	2.433984000	0.866716000	-2.585014000

Int2

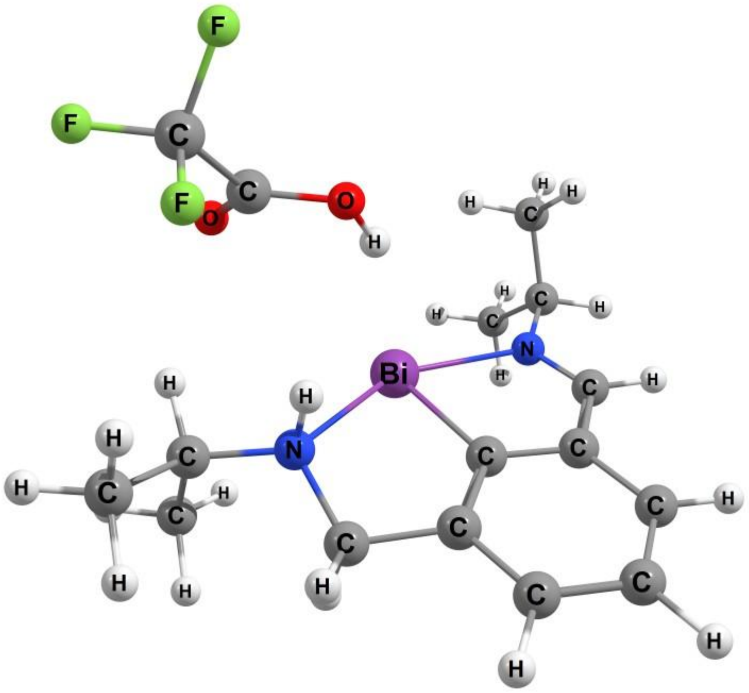

Bi	0.698619896	-0.261725446	0.945565703
N	2.771571020	0.697044678	0.283333273
N	-1.911027812	-0.204385455	0.350521010
C	-1.289055192	3.033201315	-1.356191435
H	-2.309875637	3.361898488	-1.575402855
C	-1.083945278	1.919103985	-0.550681129
C	-2.230946927	1.197717576	0.113192034
H	-3.154064163	1.328437733	-0.481306895
C	1.320296890	2.221744670	-0.806612507
C	-0.202985590	3.752843129	-1.887443884
H	-0.389037477	4.626111008	-2.516327173
C	0.227393961	1.492659650	-0.287404401
C	1.093805226	3.358140376	-1.610476428

H	1.942372942	3.918802108	-2.013453132
C	2.632285180	1.742964358	-0.471554306
H	3.522279594	2.260711504	-0.862942992
C	-2.844068929	-0.974106206	1.191420926
C	-4.234462036	-1.096776444	0.575381084
H	-4.753608278	-0.126054697	0.537346487
H	-4.857880602	-1.782297674	1.168671796
H	-4.179289962	-1.499713555	-0.448253093
C	-2.888577977	-0.432389799	2.611524846
H	-1.873178988	-0.305495938	3.017102162
H	-3.433105326	-1.133162629	3.260551309
H	-3.406002619	0.537711584	2.671621593
C	4.095485214	0.178083057	0.587164357
C	4.330182828	-1.128006408	-0.163714219
H	4.208718065	-0.984599995	-1.247258525
H	3.609864434	-1.896036948	0.158062231
H	5.346425814	-1.504829357	0.027172647
C	4.260007777	0.008633270	2.090790821
H	5.274950419	-0.341882684	2.330877906
H	3.543182632	-0.731795855	2.480271368
H	4.083253302	0.958324937	2.615834381
C	-0.926331349	-3.333820156	-0.901530311
O	-0.211397419	-2.459274790	-1.566200471
O	-0.870658278	-3.591599973	0.269230203
H	0.340662226	-1.912407202	-0.921554325
C	-1.962576310	-3.991629930	-1.834165479
H	4.841997023	0.916491114	0.235561776
H	-2.407792721	-1.984864147	1.238500106

F	-1.374082320	-4.585229299	-2.866372649
F	-2.792683838	-3.053546326	-2.307646763
F	-2.679480334	-4.885333896	-1.178239886
H	-1.837280436	-0.665395769	-0.557026756
H	-2.415526515	1.671182782	1.092520310

TS23

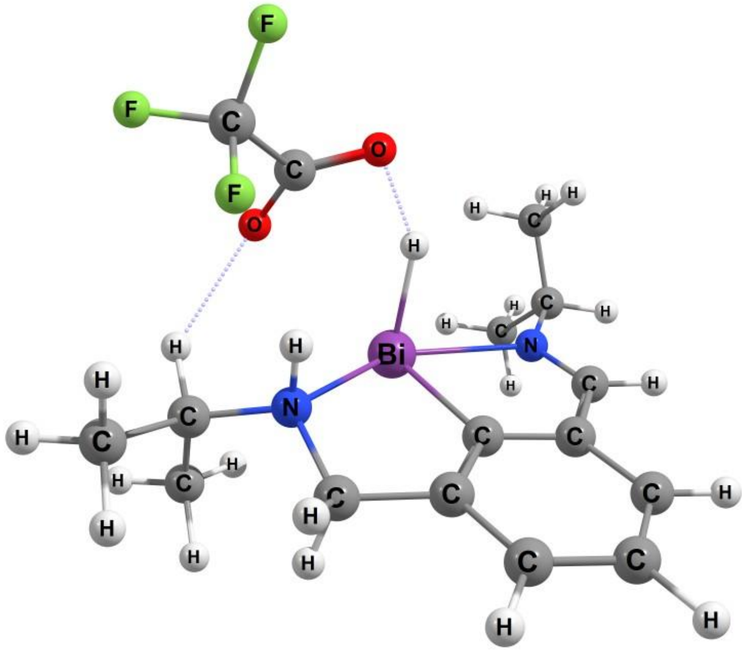

Bi	0.530486390	-0.433569368	0.838862261
N	2.714229107	0.625495000	0.250070850
N	-1.963565771	-0.313921973	0.263083155
C	-1.347143199	2.980553241	-1.418719066
H	-2.362124618	3.296504630	-1.679009487
C	-1.155585906	1.839130164	-0.633949985
C	-2.321392516	1.063630945	-0.068405945
H	-3.177115991	1.107443310	-0.764507665
C	1.239836277	2.180522542	-0.785967700
C	-0.255939177	3.723323842	-1.879020832
H	-0.424561802	4.609785393	-2.494155077

C	0.141326766	1.436456413	-0.327069881
C	1.040260668	3.330992933	-1.563379951
H	1.896801440	3.905976730	-1.926732837
C	2.567049201	1.688242234	-0.449058633
H	3.449120947	2.236668233	-0.823236534
C	-2.868098010	-1.014004931	1.205662988
C	-4.299415663	-1.084582938	0.687654270
H	-4.780944003	-0.093750373	0.660186980
H	-4.904924860	-1.726643461	1.344355301
H	-4.330010137	-1.520782672	-0.322131619
C	-2.790592798	-0.417840636	2.601791618
H	-1.752488268	-0.376527554	2.964967604
H	-3.365624966	-1.041310020	3.301317208
H	-3.212407223	0.598868636	2.652261381
C	4.031475366	0.088142841	0.540636526
C	4.201273027	-1.244894981	-0.178893530
H	4.083608334	-1.124996544	-1.265291948
H	3.448612026	-1.975917765	0.155117638
H	5.199418567	-1.661543119	0.021535428
C	4.208801573	-0.044107133	2.047013950
H	5.215818221	-0.418803461	2.282134305
H	3.476143002	-0.752262161	2.465474601
H	4.072615417	0.924917698	2.549189121
C	-0.530802340	-3.158915510	-0.873921246
O	0.471522000	-2.781452836	-1.518799532
O	-0.751174262	-3.075445542	0.342433118
H	0.825260909	-1.503693231	-0.660927282
C	-1.728283043	-3.617004727	-1.742077230

H	4.793001610	0.795143296	0.156278058
H	-2.461932621	-2.036618284	1.237942101
F	-1.377271361	-4.116015529	-2.917443315
F	-2.494887636	-2.516180208	-1.991401947
F	-2.512159528	-4.496064359	-1.129148784
H	-1.952081634	-0.861703301	-0.599254135
H	-2.650373845	1.562032595	0.858567927

Int3

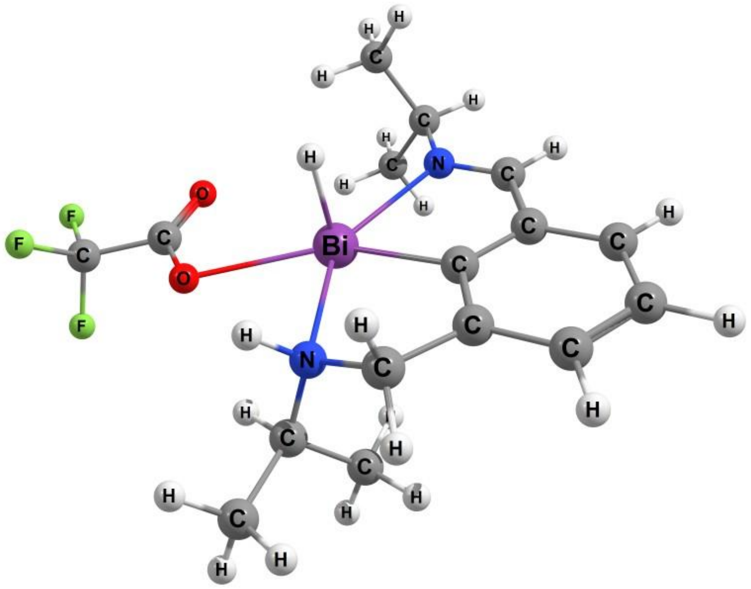

Bi	0.297958484	-0.680524873	-0.089006803
N	2.545915256	0.522880140	0.008670568
N	-2.166840597	-0.174467490	-0.411848214
C	-1.402152321	3.511064230	-0.626610598
H	-2.387745591	3.947512487	-0.818684913
C	-1.242498013	2.119634183	-0.597948397
C	-2.383766093	1.175018672	-0.925558497
H	-2.466106733	1.114267785	-2.023669577
C	1.117546192	2.426690065	-0.161732244
C	-0.308062385	4.353452946	-0.417151349

H	-0.446935340	5.436739921	-0.436886999
C	0.013437573	1.580891632	-0.347036013
C	0.959131130	3.818959898	-0.192851938
H	1.818439326	4.479729101	-0.044983430
C	2.424463838	1.794215016	0.022924758
H	3.310792768	2.440603737	0.160661780
C	-2.895051512	-0.557416252	0.816054282
C	-4.392335736	-0.696348786	0.572083458
H	-4.858567614	0.264856482	0.301665317
H	-4.894839009	-1.062440846	1.479653510
H	-4.591883500	-1.419174068	-0.233600698
C	-2.583238698	0.388822672	1.964218698
H	-1.501329463	0.467497362	2.148404310
H	-3.056107710	0.023007798	2.886760523
H	-2.963112151	1.405036251	1.775384541
C	3.828004059	-0.135152995	0.168606548
C	4.112342538	-0.976158514	-1.070325479
H	4.102090458	-0.357893078	-1.979786795
H	3.357692675	-1.771603337	-1.180851388
H	5.099635372	-1.453778994	-0.985693965
C	3.804107510	-0.992580447	1.429212676
H	4.784604516	-1.466731712	1.582750889
H	3.053412395	-1.794120982	1.338526552
H	3.568940765	-0.388367427	2.317933712
C	-0.295423024	-3.638700500	-0.463781015
O	0.921429264	-3.488543804	-0.348231947
O	-1.176014428	-2.747017055	-0.598720499
H	0.594205140	-0.796957638	-1.852534361

C	-0.869639094	-5.072889652	-0.394394600
H	4.616669436	0.636854632	0.273163211
H	-2.502887323	-1.559307479	1.055594230
F	0.077253407	-5.996581028	-0.278687017
F	-1.590911811	-5.358611071	-1.482644611
F	-1.680535591	-5.186906452	0.666588827
H	-2.352965985	-0.890039768	-1.113198572
H	-3.342429621	1.592552637	-0.573151551

Int4

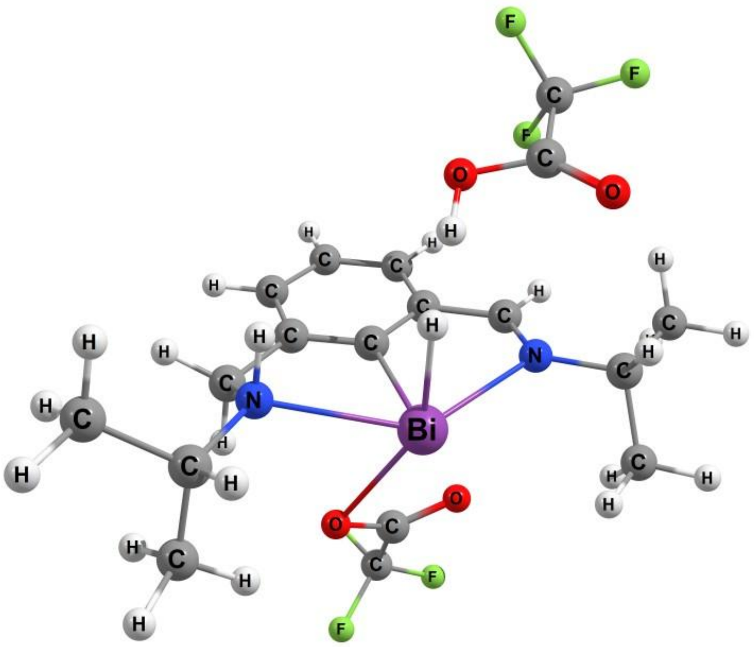

Bi	-0.427939661	0.750451275	-0.456821868
C	2.164423920	-0.366296203	1.008745594
H	3.170284858	-0.562378405	1.405502677
C	-0.248996683	-0.709871939	1.203662039
C	1.030087648	-0.997241776	1.681616772
C	-1.383148511	-1.276982873	1.768350974
N	-2.629960372	-0.541297749	-0.191416930
N	1.986321459	0.373386112	-0.016731471

C	2.832231991	2.561233635	-0.619350573
H	2.841366514	2.885066705	0.431349462
H	1.855828984	2.849580786	-1.036053462
C	1.185749858	-1.880105704	2.755639116
H	2.183537669	-2.122077725	3.130635871
C	3.062226719	1.056258609	-0.723810460
C	-1.219294270	-2.162535427	2.840802333
H	-2.095723304	-2.627895391	3.301238758
C	-2.736084447	-0.882627767	1.230302501
H	-3.469797298	-1.688134089	1.410781924
C	0.054781523	-2.456564430	3.329968376
H	0.164356990	-3.150139477	4.166275786
C	-3.842571045	-0.023182232	-0.859385631
C	-4.398504299	1.207974332	-0.162941923
H	-3.608595901	1.927610562	0.091408569
H	-4.911070109	0.942565981	0.774367527
C	4.461124310	0.656912846	-0.285949650
H	4.622463974	-0.426130142	-0.389308137
H	5.196973378	1.159983123	-0.928687645
H	4.669499388	0.957672451	0.753203347
C	-4.902191522	-1.104409939	-1.041630141
H	-5.306096374	-1.440687326	-0.073940138
H	-5.745059265	-0.715943722	-1.632222719
H	-4.499694876	-1.982046881	-1.572558004
H	3.611629001	3.100775877	-1.176608527
H	-5.138436614	1.694509371	-0.814447022
H	-0.176199323	-0.733326904	-1.696903207
C	-0.783880621	2.500001909	1.967206503

O	0.376506648	2.395276621	1.542091991
O	-1.809340550	1.965695846	1.491950922
H	0.657928818	-1.915824114	-1.464488826
C	-1.014875017	3.343459165	3.241759386
C	2.464570724	-2.503945536	-1.547292794
O	2.887547323	-1.674227315	-2.303217654
O	1.227423976	-2.687819441	-1.163231729
C	3.399479976	-3.501457216	-0.833886252
H	2.941608541	0.749698473	-1.777322878
H	-3.498765794	0.279560738	-1.864456072
F	-1.250080687	2.536948640	4.283535937
F	-2.074252888	4.141669062	3.103632632
F	0.033008670	4.101992906	3.544076001
F	2.982689153	-4.753630907	-0.972888616
F	3.435453607	-3.216647157	0.475242972
F	4.629949984	-3.403743591	-1.307330581
H	-2.330261748	-1.378227316	-0.694832493
H	-3.067126239	0.019178339	1.767169981

TS45

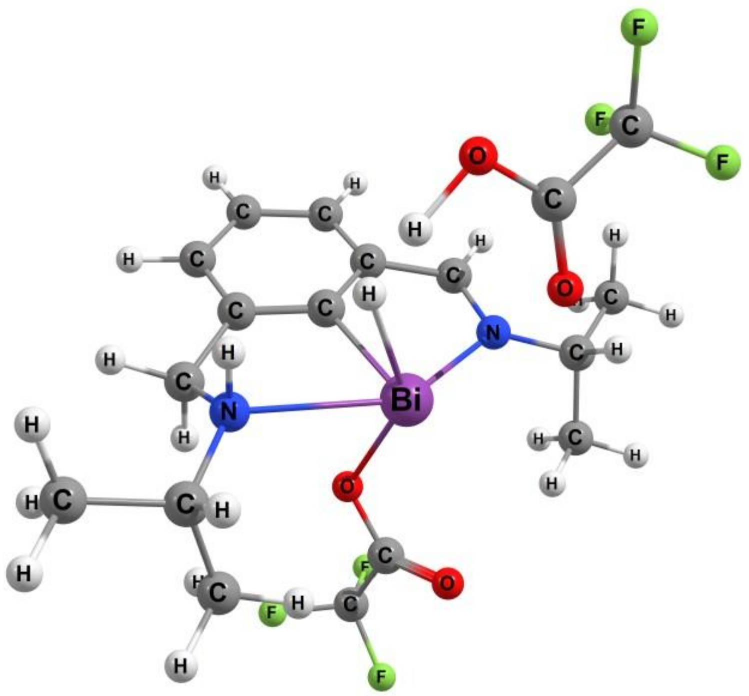

Bi	-0.214763166	0.072142091	-0.475769871
C	2.240121890	-0.809006288	1.440569687
H	3.213897629	-1.059101896	1.888046331
C	-0.175994533	-1.223765770	1.352115327
C	1.053985529	-1.507843717	1.948853726
C	-1.366907668	-1.628135299	1.943320632
N	-2.589234363	-0.779084006	-0.003087189
N	2.123383199	0.095670844	0.551320744
C	2.910104282	2.363510492	0.287603019
H	2.874917429	2.576325056	1.367973212
H	1.941317269	2.652304935	-0.146773441
C	1.107223045	-2.327628927	3.081456072
H	2.068560708	-2.565642338	3.545158272
C	3.228053865	0.892197761	0.038341402
C	-1.309087967	-2.445192591	3.080711982
H	-2.234760541	-2.789413632	3.551289862
C	-2.672216678	-1.079744485	1.424158477

H	-3.498298482	-1.775708308	1.650789069
C	-0.079871983	-2.812892288	3.627589082
H	-0.050340822	-3.459507748	4.507221606
C	-3.743699409	-0.087861192	-0.616233481
C	-3.756013589	1.398361767	-0.295449284
H	-2.887891652	1.915596410	-0.728717805
H	-3.757510249	1.591700869	0.786880854
C	4.597587686	0.496777985	0.563610955
H	4.829618205	-0.554983216	0.339607741
H	5.363584594	1.115872093	0.075767230
H	4.685947310	0.660625958	1.650095241
C	-5.072260516	-0.757996549	-0.282032665
H	-5.354325516	-0.601097992	0.770483961
H	-5.874387333	-0.333289644	-0.902816129
H	-5.039217722	-1.841975265	-0.474464005
H	3.686787652	2.997932645	-0.163488583
H	-4.658869323	1.860746217	-0.719715307
H	-0.697012615	-1.830716123	-1.481713451
C	-0.866463177	2.756145302	0.786420506
O	-0.474799817	3.138718807	-0.304824767
O	-0.915161644	1.560102132	1.216896389
H	0.097479185	-2.405770704	-1.723418635
C	-1.377007755	3.781571517	1.822201721
C	2.025153940	-2.104463691	-2.092697334
O	1.872839235	-0.887103117	-2.019164709
O	1.141414303	-3.003625380	-1.991463521
C	3.471285915	-2.626182019	-2.229578838
H	3.208850781	0.710067195	-1.049247330

H	-3.579542930	-0.195720050	-1.702104464
F	-0.533782240	3.851027267	2.853680775
F	-2.574395772	3.415691273	2.296956415
F	-1.497777974	4.993959139	1.303370551
F	3.532766206	-3.844756352	-2.741747820
F	4.016843015	-2.664791438	-1.000763357
F	4.210822909	-1.814570372	-2.974476265
H	-2.441810158	-1.657785087	-0.504081154
H	-2.874067335	-0.137064307	1.959873808

Int5(Product)

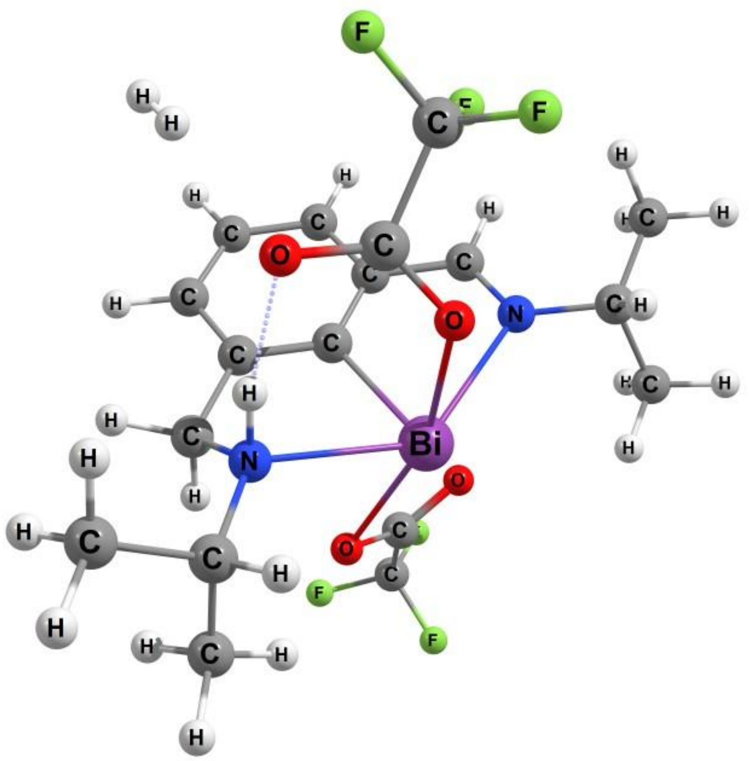

Bi	-0.111768555	0.968655661	-0.155393105
C	2.418150873	-0.340346657	1.337028031
H	3.402647974	-0.684137705	1.687860221
C	-0.015689702	-0.622746723	1.387743658
C	1.228627315	-0.977778343	1.910324402

C	-1.184758687	-1.263056315	1.773118644
N	-2.177268719	-0.452098370	-0.282260515
N	2.299322563	0.545170047	0.430283226
C	3.368042370	2.676189250	0.016425165
H	3.550177667	2.877970971	1.082654548
H	2.385707113	3.104337566	-0.229457221
C	1.301222425	-1.969553223	2.893409530
H	2.268219948	-2.266435673	3.308517551
C	3.410242089	1.175983000	-0.258897718
C	-1.103055535	-2.254170896	2.759545215
H	-2.005947817	-2.783932618	3.075725221
C	-2.473629469	-0.880263414	1.087437816
H	-3.173051751	-1.734027162	1.107252941
C	0.127977274	-2.589775296	3.322974020
H	0.176158678	-3.368792420	4.086803576
C	-3.310126489	-0.022126443	-1.122942927
C	-4.083195917	1.135787088	-0.513154586
H	-3.416131057	1.939429697	-0.172387055
H	-4.677942799	0.812292274	0.355075997
C	4.772594108	0.571378162	0.034991841
H	4.789517716	-0.505933648	-0.188622472
H	5.531781630	1.056408863	-0.594797981
H	5.070645050	0.723409915	1.084820750
C	-4.213886085	-1.194353615	-1.489007594
H	-4.754878115	-1.578718373	-0.610172844
H	-4.966890988	-0.879841704	-2.226842330
H	-3.630994915	-2.019670056	-1.925472933
H	4.132459113	3.195142486	-0.579662269

H	-4.784548874	1.544604355	-1.254827530
H	-0.301422890	-4.856173668	0.661848590
C	-0.761057496	2.655903500	2.058282055
O	0.444894873	2.542030662	1.780363460
O	-1.691174276	2.058875791	1.471369235
H	-0.331692094	-4.369413714	0.075118029
C	-1.157289724	3.652931193	3.164459774
C	0.487965053	-1.850190765	-1.712287692
O	0.689926074	-0.599469049	-1.729675251
O	-0.541539310	-2.457200005	-1.442534967
C	1.762365760	-2.661115191	-2.037551609
H	3.189374581	1.012692859	-1.330073745
H	-2.840368217	0.334234169	-2.056808148
F	-2.284455821	3.289774461	3.765910450
F	-1.348490594	4.856862132	2.618001697
F	-0.208259333	3.764228814	4.085449665
F	1.520825808	-3.956612832	-2.161174492
F	2.649061406	-2.501626152	-1.038655879
F	2.340058378	-2.233659842	-3.158265418
H	-1.713238876	-1.250219501	-0.758763543
H	-2.938954531	-0.041747801	1.629046324

Int5 (6)

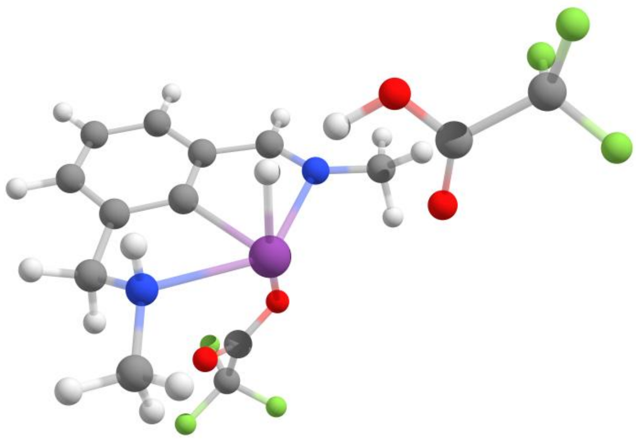

Bi	-0.195544393	-0.181867430	-0.309342480
C	2.045019851	-0.776983311	1.890693836
H	2.953922477	-0.775031651	2.516597853
C	-0.378761883	-1.038491864	1.747014863
C	0.789586910	-1.221828111	2.490664278
C	-1.620204235	-1.386460505	2.264892029
N	-2.569220351	-1.117875212	0.017521017
N	2.090998883	-0.358046066	0.686204016
H	3.084974482	1.306322210	-0.028461851
C	0.721524648	-1.770745164	3.777309047
H	1.631454331	-1.912454594	4.367065155
C	3.284880748	0.237616196	0.149881539
C	-1.681865115	-1.945380196	3.547413895
H	-2.649410997	-2.228099831	3.972714852
C	-2.850254976	-1.074351157	1.453296490
H	-3.686227595	-1.746728461	1.721492316
C	-0.518518218	-2.137349330	4.295076853
H	-0.583621052	-2.573952355	5.293985018
C	-3.591513957	-0.465672282	-0.790628336
H	-3.590163683	0.607261895	-0.552642407

H	4.140086506	0.145154943	0.840164622
H	-4.600705473	-0.874968468	-0.604290407
H	0.035203532	-2.055350467	-0.858526680
C	-0.834762241	2.308461723	1.196518507
O	0.366917764	2.010417961	1.081607148
O	-1.812614074	1.661183623	0.768463895
H	0.984400388	-2.538762235	-1.763591119
C	-1.123547301	3.622126993	1.956557011
C	2.260275234	-1.708587275	-2.874645914
O	1.927621539	-0.572499009	-2.642954966
O	1.721039916	-2.799757782	-2.414382897
C	3.506094789	-1.989948797	-3.737222552
H	3.539907521	-0.211377977	-0.818704925
H	-3.358595172	-0.590889997	-1.857628964
F	-0.630827639	3.557200197	3.196061990
F	-2.424561909	3.879328274	2.049363698
F	-0.543250228	4.654078465	1.340195967
F	3.467001597	-3.182189235	-4.313101908
F	4.580606990	-1.942286654	-2.942058604
F	3.640065371	-1.065513418	-4.673628209
H	-2.452606425	-2.089523994	-0.271137488
H	-3.159715288	-0.041838349	1.682365079

TS45 (6)

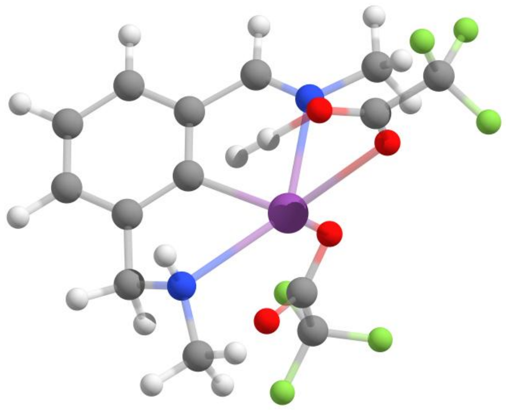

Bi	-0.042535000	-0.137355000	-0.251358000
C	2.091556000	-0.804961000	2.036207000
H	2.988212000	-0.877304000	2.674864000
C	-0.332233000	-1.032015000	1.799256000
C	0.804685000	-1.234002000	2.586380000
C	-1.593109000	-1.397397000	2.258403000
N	-2.447630000	-1.174623000	-0.024786000
N	2.174385000	-0.332408000	0.855646000
H	3.279458000	1.288989000	0.208676000
C	0.686154000	-1.803027000	3.859211000
H	1.572466000	-1.960271000	4.480088000
C	3.401864000	0.200503000	0.329287000
C	-1.706102000	-1.972807000	3.531333000
H	-2.689081000	-2.268992000	3.909191000
C	-2.793194000	-1.112617000	1.393037000
H	-3.629104000	-1.793499000	1.641148000
C	-0.575195000	-2.171638000	4.323543000
H	-0.680420000	-2.622688000	5.312561000
C	-3.458936000	-0.599793000	-0.899833000
H	-3.532087000	0.477977000	-0.697050000
H	4.256055000	0.005268000	0.998482000
H	-4.454946000	-1.056675000	-0.756097000

H	0.034018000	-2.329718000	-0.666737000
C	-0.757448000	2.312845000	1.036734000
O	0.444251000	1.986288000	0.917427000
O	-1.722300000	1.636753000	0.639575000
H	0.538638000	-2.510543000	-1.392615000
C	-1.027960000	3.655645000	1.744057000
C	2.020223000	-1.703052000	-2.686952000
O	1.865686000	-0.600538000	-2.138906000
O	1.381336000	-2.761036000	-2.498567000
C	3.159408000	-1.766850000	-3.730749000
H	3.585383000	-0.213328000	-0.670828000
H	-3.164275000	-0.738382000	-1.949748000
F	-0.544339000	3.617904000	2.985004000
F	-2.324863000	3.923606000	1.812442000
F	-0.427766000	4.650512000	1.094425000
F	3.342777000	-2.987887000	-4.213366000
F	4.309905000	-1.366032000	-3.175453000
F	2.896468000	-0.951715000	-4.752541000
H	-2.269528000	-2.146083000	-0.282379000
H	-3.134775000	-0.084183000	1.594691000

Int5 (6)

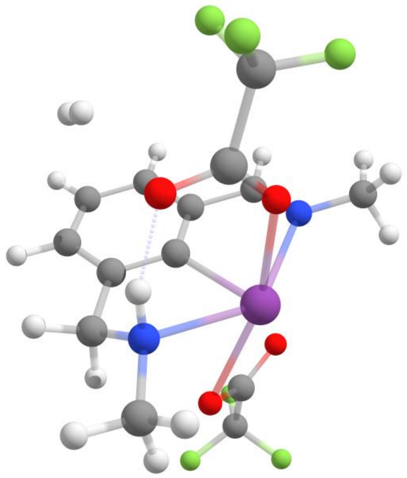

Bi	0.190508000	0.733032000	-0.345540000
C	2.383489000	-0.305632000	1.769874000
H	3.284760000	-0.527490000	2.367207000
C	0.004496000	-0.698991000	1.341937000
C	1.123109000	-0.954037000	2.138706000
C	-1.207972000	-1.335083000	1.560957000
N	-1.873683000	-0.633990000	-0.685165000
N	2.441490000	0.471879000	0.763424000
H	3.549576000	2.168546000	0.298346000
C	1.015892000	-1.843764000	3.211749000
H	1.883428000	-2.057355000	3.841966000
C	3.665273000	1.073618000	0.322472000
C	-1.308963000	-2.223210000	2.640057000
H	-2.252491000	-2.741525000	2.832512000
C	-2.358045000	-1.031194000	0.635833000
H	-3.034847000	-1.902088000	0.563610000
C	-0.208043000	-2.465537000	3.460833000
H	-0.300890000	-3.163454000	4.295685000
C	-2.902381000	-0.046610000	-1.529044000
H	-3.229748000	0.912421000	-1.101035000
H	4.522522000	0.816463000	0.967578000
H	-3.780801000	-0.708419000	-1.629396000
H	0.207912000	-4.205091000	0.884063000
C	-0.929619000	2.601103000	1.499184000
O	0.311057000	2.506902000	1.472643000
O	-1.715460000	1.925880000	0.799826000
H	0.209259000	-4.108075000	0.128546000
C	-1.534306000	3.660279000	2.442294000

C	0.910942000	-2.060379000	-1.969287000
O	1.329018000	-0.938921000	-1.560429000
O	-0.223254000	-2.525580000	-1.932500000
C	2.021920000	-2.967810000	-2.551425000
H	3.873704000	0.734114000	-0.705022000
H	-2.496884000	0.131191000	-2.535047000
F	-0.943917000	3.628556000	3.633074000
F	-2.836009000	3.474401000	2.619681000
F	-1.355331000	4.875768000	1.921137000
F	1.602638000	-3.607154000	-3.638261000
F	2.366071000	-3.884369000	-1.640122000
F	3.117151000	-2.284134000	-2.872562000
H	-1.430982000	-1.447702000	-1.157285000
H	-2.937365000	-0.188416000	1.048129000

Int 4 (3)

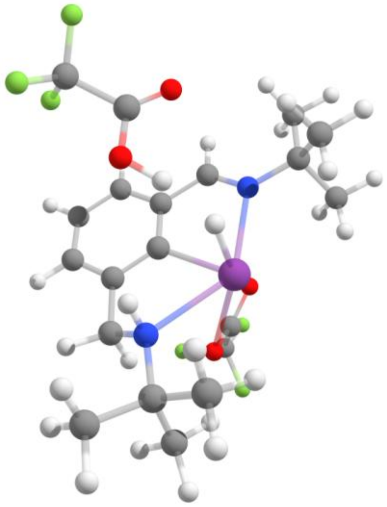

Bi	-0.270185000	0.931719000	-0.444032000
C	2.385803000	-0.136708000	0.922274000
H	3.408073000	-0.338399000	1.268783000
C	-0.007270000	-0.604568000	1.135174000
C	1.294027000	-0.862826000	1.569746000

C	-1.100390000	-1.275099000	1.667042000
N	-2.415728000	-0.536110000	-0.242531000
N	2.152098000	0.680165000	-0.028347000
C	2.867543000	2.907174000	-0.593743000
H	2.826562000	3.187745000	0.468113000
H	1.888425000	3.150242000	-1.034224000
C	1.513437000	-1.822651000	2.564154000
H	2.529052000	-2.046238000	2.901029000
C	3.189129000	1.418820000	-0.753229000
C	-0.871568000	-2.235848000	2.659702000
H	-1.714504000	-2.785513000	3.088365000
C	-2.478644000	-0.922535000	1.169637000
H	-3.170786000	-1.765571000	1.339795000
C	0.424353000	-2.503976000	3.103270000
H	0.585300000	-3.261859000	3.872678000
C	-3.684247000	-0.119921000	-0.896043000
C	-4.332475000	1.026006000	-0.125138000
H	-3.611026000	1.825617000	0.090830000
H	-4.743839000	0.688103000	0.837031000
C	4.597150000	1.129692000	-0.240500000
H	4.854255000	0.064631000	-0.345747000
H	5.322477000	1.707575000	-0.830367000
H	4.716136000	1.426073000	0.813016000
C	-4.647874000	-1.307176000	-0.993055000
H	-4.934374000	-1.683298000	0.000096000
H	-5.572311000	-1.007530000	-1.508457000
H	-4.200965000	-2.138329000	-1.562870000
H	3.628130000	3.516064000	-1.104589000

H	-5.165889000	1.434920000	-0.714585000
H	0.093435000	-0.479452000	-1.735912000
C	-0.807154000	2.496467000	2.070725000
O	0.378231000	2.481296000	1.712148000
O	-1.780982000	1.980055000	1.477676000
H	0.529413000	-1.832776000	-1.429950000
C	-1.163605000	3.187355000	3.406411000
C	1.895880000	-3.081961000	-0.949373000
O	2.839130000	-2.340100000	-1.005883000
O	0.645262000	-2.810970000	-1.214929000
C	2.075998000	-4.539959000	-0.478858000
F	-1.625559000	2.280613000	4.275940000
F	-2.121560000	4.100002000	3.228149000
F	-0.121934000	3.792732000	3.964873000
F	1.130826000	-5.344277000	-0.946923000
F	2.035182000	-4.576694000	0.854888000
F	3.255110000	-4.999714000	-0.870918000
H	-2.056033000	-1.337053000	-0.767029000
H	-2.837015000	-0.052747000	1.740658000
C	-3.315626000	0.342279000	-2.304873000
H	-2.700562000	1.255121000	-2.273387000
H	-2.754004000	-0.434214000	-2.848793000
H	-4.221011000	0.571867000	-2.884792000
C	3.079499000	0.992463000	-2.220334000
H	3.828114000	1.522911000	-2.827235000
H	3.237747000	-0.091367000	-2.313911000
H	2.083567000	1.228222000	-2.625409000

TS45 (3)

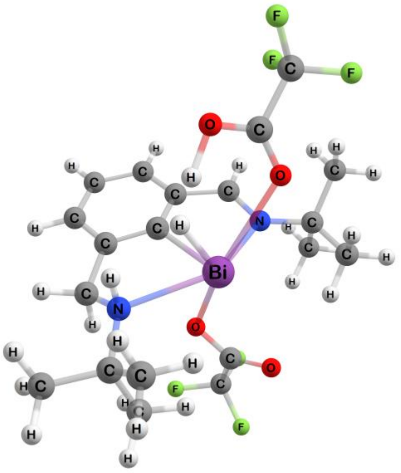

Bi	-0.205233000	-0.048129000	-0.638060000
C	2.206415000	-0.625267000	1.434841000
H	3.172243000	-0.790599000	1.939444000
C	-0.170798000	-1.214871000	1.266230000
C	1.048241000	-1.379615000	1.928404000
C	-1.363608000	-1.612465000	1.856802000
N	-2.588166000	-0.849501000	-0.138387000
N	2.069617000	0.241633000	0.512349000
C	3.311846000	2.214339000	1.143081000
H	3.622126000	1.785998000	2.108706000
H	2.360890000	2.746534000	1.292120000
C	1.097785000	-2.102454000	3.125395000
H	2.050108000	-2.245843000	3.643440000
C	3.169115000	1.121319000	0.077660000
C	-1.308820000	-2.332089000	3.058961000
H	-2.235156000	-2.670246000	3.532716000
C	-2.676868000	-1.130515000	1.290900000
H	-3.479103000	-1.853351000	1.518802000
C	-0.084733000	-2.601184000	3.669979000
H	-0.057892000	-3.170712000	4.601460000

C	-3.738728000	-0.158849000	-0.780142000
C	-3.733317000	1.326429000	-0.425866000
H	-2.877560000	1.844104000	-0.883300000
H	-3.688089000	1.497612000	0.658762000
C	4.475611000	0.345564000	-0.091442000
H	4.340299000	-0.470795000	-0.813800000
H	5.258183000	1.019371000	-0.469111000
H	4.840674000	-0.076168000	0.857113000
C	-5.063665000	-0.799995000	-0.357591000
H	-5.281927000	-0.627858000	0.706433000
H	-5.892285000	-0.368160000	-0.937336000
H	-5.056853000	-1.886517000	-0.540669000
H	4.071638000	2.945874000	0.830482000
H	-4.650468000	1.798422000	-0.807063000
H	-0.733024000	-1.960021000	-1.561429000
C	-0.764092000	2.690554000	0.601121000
O	-0.372605000	3.064529000	-0.494025000
O	-0.929211000	1.497472000	1.002811000
H	0.034386000	-2.667131000	-1.704511000
C	-1.070548000	3.748612000	1.685284000
C	1.969114000	-2.542217000	-1.878885000
O	1.938090000	-1.322483000	-1.745593000
O	0.989061000	-3.346800000	-1.870942000
C	3.357921000	-3.201804000	-2.007847000
F	0.038437000	3.991883000	2.396289000
F	-2.012036000	3.332497000	2.532293000
F	-1.474283000	4.895362000	1.154033000
F	3.298610000	-4.419505000	-2.521757000

F	3.904394000	-3.284852000	-0.788858000
F	4.160231000	-2.459212000	-2.764438000
H	-2.440141000	-1.734878000	-0.629034000
H	-2.923954000	-0.188619000	1.808036000
C	-3.558703000	-0.324079000	-2.288189000
H	-2.608825000	0.115587000	-2.630089000
H	-3.567482000	-1.385881000	-2.581614000
H	-4.371946000	0.183712000	-2.825815000
C	2.769584000	1.751870000	-1.255231000
H	3.550825000	2.450867000	-1.587761000
H	2.653232000	0.976380000	-2.025323000
H	1.830971000	2.319736000	-1.156013000

Int 5 (3)

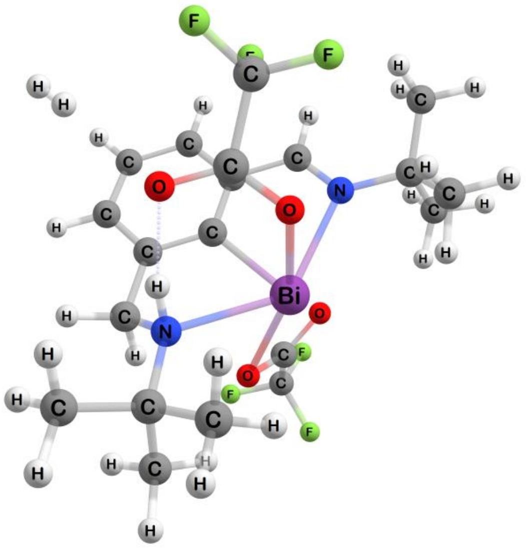

Bi	-0.119656000	0.896856000	-0.264042000
C	2.374657000	-0.399046000	1.318774000
H	3.341650000	-0.750940000	1.707948000
C	-0.068953000	-0.630127000	1.347480000
C	1.164900000	-1.003420000	1.882110000

C	-1.251835000	-1.236385000	1.747232000
N	-2.245008000	-0.482940000	-0.331023000
N	2.300513000	0.474759000	0.395907000
C	3.672436000	2.456023000	0.479722000
H	3.959348000	2.308776000	1.531882000
H	2.744061000	3.044366000	0.472905000
C	1.213592000	-1.978226000	2.883553000
H	2.173816000	-2.288013000	3.304754000
C	3.473256000	1.107752000	-0.218676000
C	-1.194397000	-2.209217000	2.753343000
H	-2.110504000	-2.709872000	3.078701000
C	-2.533107000	-0.837901000	1.060901000
H	-3.261756000	-1.663562000	1.131053000
C	0.027502000	-2.564434000	3.323533000
H	0.057325000	-3.330688000	4.101111000
C	-3.393352000	-0.095650000	-1.189327000
C	-4.170734000	1.059202000	-0.565888000
H	-3.503331000	1.884867000	-0.281833000
H	-4.715217000	0.746279000	0.337179000
C	4.731560000	0.248013000	-0.103061000
H	4.563366000	-0.752039000	-0.531485000
H	5.552488000	0.725160000	-0.656845000
H	5.068599000	0.135869000	0.938375000
C	-4.307803000	-1.306219000	-1.405139000
H	-4.758627000	-1.648877000	-0.462040000
H	-5.131544000	-1.047194000	-2.086668000
H	-3.746986000	-2.145122000	-1.845862000
H	4.466176000	3.028459000	-0.023126000

H	-4.915354000	1.432877000	-1.283877000
H	-0.594264000	-5.017867000	0.756148000
C	-0.757808000	2.710837000	1.852457000
O	0.447328000	2.569880000	1.584344000
O	-1.693651000	2.113208000	1.275673000
H	-0.587510000	-4.516005000	0.181403000
C	-1.156833000	3.719682000	2.946678000
C	0.407700000	-2.049264000	-1.610503000
O	0.623964000	-0.806705000	-1.726631000
O	-0.646689000	-2.622358000	-1.364183000
C	1.689234000	-2.907349000	-1.733300000
F	-0.140007000	4.004483000	3.748107000
F	-2.153321000	3.243607000	3.686923000
F	-1.568821000	4.855340000	2.377938000
F	1.426598000	-4.162307000	-2.061345000
F	2.317055000	-2.915196000	-0.543445000
F	2.544569000	-2.411299000	-2.627830000
H	-1.799738000	-1.312103000	-0.769018000
H	-2.958513000	0.040282000	1.570865000
C	-2.807332000	0.330854000	-2.535014000
H	-2.236483000	1.268665000	-2.442111000
H	-2.137594000	-0.443781000	-2.938623000
H	-3.609849000	0.506505000	-3.265803000
C	3.139214000	1.315785000	-1.696737000
H	3.997694000	1.755572000	-2.224582000
H	2.867410000	0.363355000	-2.172971000
H	2.290508000	2.009152000	-1.810159000

Int 4 (7)

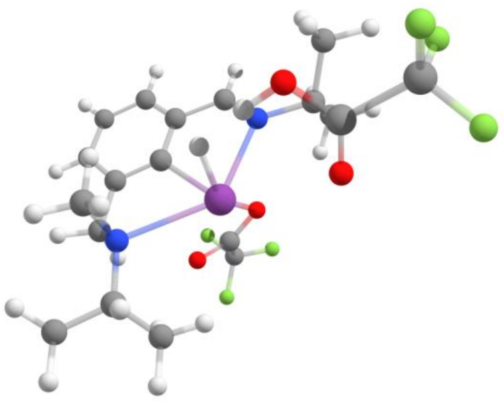

Bi	-0.259582000	0.037339000	-0.253096000
C	1.952521000	-0.519617000	1.930666000
H	2.866917000	-0.542302000	2.545188000
C	-0.465100000	-0.813155000	1.789021000
C	0.698962000	-0.940922000	2.550163000
C	-1.707670000	-1.166565000	2.297628000
N	-2.641368000	-1.206058000	0.019191000
N	1.983348000	-0.108963000	0.720717000
C	3.620803000	1.714697000	0.847093000
H	3.992561000	1.496250000	1.861251000
H	2.759686000	2.392805000	0.933811000
C	0.624291000	-1.435234000	3.859245000
H	1.528203000	-1.536795000	4.466231000
C	3.209650000	0.434689000	0.127151000
C	-1.773795000	-1.668707000	3.602437000
H	-2.740158000	-1.959277000	4.024995000
C	-2.928627000	-0.955701000	1.438280000
H	-3.754074000	-1.599641000	1.794071000
C	-0.617061000	-1.797682000	4.375856000
H	-0.689287000	-2.187320000	5.393526000
C	-3.692259000	-0.700892000	-0.906382000
C	-3.637081000	0.804905000	-1.105135000

H	-2.683921000	1.123688000	-1.551906000
H	-3.751301000	1.361888000	-0.165770000
C	4.315482000	-0.611895000	0.068929000
H	3.974797000	-1.520386000	-0.448538000
H	5.179580000	-0.212354000	-0.480874000
H	4.660247000	-0.893559000	1.076515000
C	-5.103236000	-1.140974000	-0.519908000
H	-5.459458000	-0.603761000	0.372053000
H	-5.797924000	-0.908920000	-1.339944000
H	-5.174038000	-2.221404000	-0.326238000
H	4.428079000	2.216190000	0.293427000
H	-4.439983000	1.109163000	-1.792557000
H	0.226669000	-1.753666000	-0.821110000
C	-0.841878000	2.506835000	1.421358000
O	0.339872000	2.242260000	1.131068000
O	-1.852238000	1.847300000	1.120177000
H	1.299172000	-2.060750000	-1.680312000
C	-1.085189000	3.764356000	2.285937000
C	2.261789000	-1.084116000	-2.978877000
O	1.650490000	-0.053083000	-2.857438000
O	2.065870000	-2.198960000	-2.330377000
C	3.473442000	-1.175603000	-3.927664000
H	2.941065000	0.702353000	-0.905121000
H	-3.447808000	-1.173285000	-1.872788000
F	-1.176603000	3.414436000	3.575178000
F	-2.221843000	4.373514000	1.952198000
F	-0.098175000	4.648756000	2.174155000
F	3.619370000	-2.390778000	-4.436659000

F	4.578641000	-0.873742000	-3.238143000
F	3.348803000	-0.308977000	-4.917560000
H	-3.233148000	0.096750000	1.546154000
C	-2.405364000	-2.631481000	-0.195998000
H	-1.584932000	-2.974256000	0.447828000
H	-3.301271000	-3.237272000	0.035311000
H	-2.116328000	-2.811357000	-1.240105000

TS 45 (7)

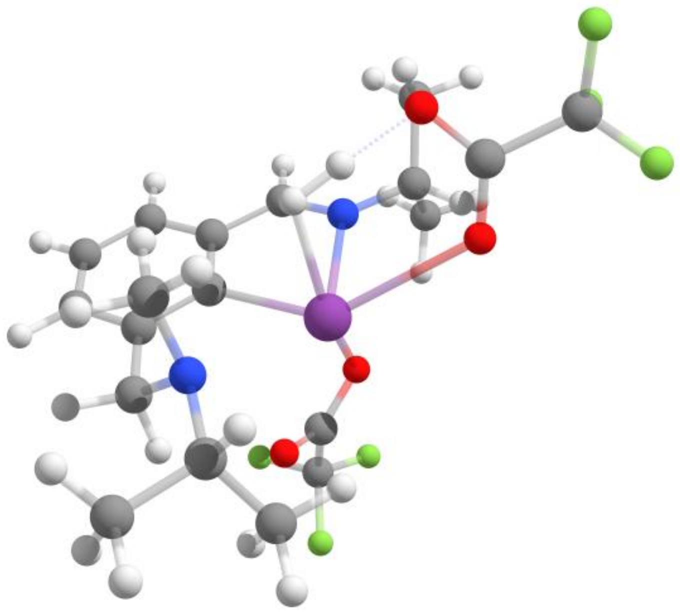

Bi	-0.215235000	0.110985000	-0.263998000
C	1.963037000	-0.566983000	1.927019000
H	2.881239000	-0.653681000	2.529342000
C	-0.465344000	-0.767879000	1.791805000
C	0.695316000	-0.947357000	2.547651000
C	-1.712489000	-1.120595000	2.294670000
N	-2.646621000	-1.231601000	0.024195000
N	2.008212000	-0.127992000	0.728772000
C	3.763923000	1.581704000	0.884532000
H	4.103774000	1.330036000	1.902380000

H	2.962183000	2.330312000	0.958835000
C	0.616563000	-1.466911000	3.845538000
H	1.521502000	-1.608776000	4.442800000
C	3.267937000	0.347870000	0.140338000
C	-1.785506000	-1.643100000	3.592388000
H	-2.755703000	-1.931193000	4.007666000
C	-2.933404000	-0.926557000	1.430114000
H	-3.761802000	-1.550976000	1.814688000
C	-0.631784000	-1.806936000	4.361890000
H	-0.709531000	-2.215570000	5.371672000
C	-3.704384000	-0.786299000	-0.920426000
C	-3.668222000	0.708233000	-1.192476000
H	-2.710545000	1.019014000	-1.635181000
H	-3.820187000	1.308577000	-0.285576000
C	4.296314000	-0.773087000	0.058803000
H	3.882718000	-1.641186000	-0.474165000
H	5.178531000	-0.424328000	-0.495839000
H	4.630580000	-1.094918000	1.058363000
C	-5.110371000	-1.225758000	-0.514215000
H	-5.474409000	-0.654136000	0.353099000
H	-5.809018000	-1.041777000	-1.343040000
H	-5.165502000	-2.297057000	-0.270911000
H	4.614885000	2.023128000	0.345649000
H	-4.459394000	0.966098000	-1.911665000
H	0.242492000	-2.011909000	-0.688362000
C	-0.786643000	2.534103000	1.196813000
O	0.380602000	2.207075000	0.880379000
O	-1.807207000	1.891023000	0.911584000

H	0.909664000	-2.081801000	-1.274401000
C	-0.956799000	3.804015000	2.054175000
C	2.101782000	-1.001189000	-2.708288000
O	1.452663000	0.016677000	-2.422588000
O	1.987970000	-2.154875000	-2.237723000
C	3.251108000	-0.794259000	-3.722804000
H	3.013471000	0.648983000	-0.883388000
H	-3.453930000	-1.298061000	-1.864668000
F	-1.085677000	3.452455000	3.336296000
F	-2.046687000	4.476623000	1.700343000
F	0.087956000	4.614005000	1.950666000
F	3.710724000	-1.935272000	-4.217498000
F	4.272318000	-0.176996000	-3.102816000
F	2.870637000	-0.021624000	-4.734194000
H	-3.241387000	0.128344000	1.498929000
C	-2.385491000	-2.659098000	-0.134963000
H	-1.564953000	-2.968116000	0.525898000
H	-3.271157000	-3.273810000	0.113567000
H	-2.088084000	-2.875225000	-1.170049000

Int 5 (7)

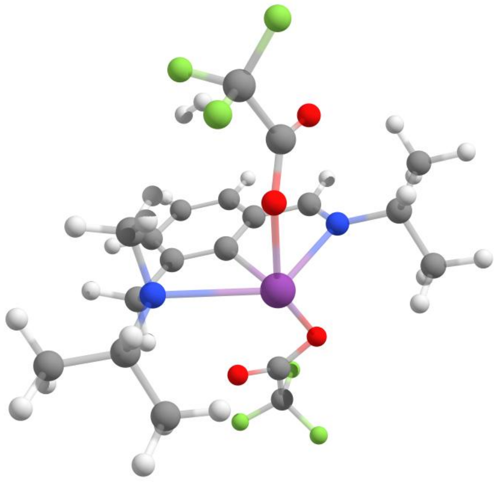

Bi	-0.015202000	0.737467000	-0.237561000
C	2.205577000	-0.415046000	1.679228000
H	3.134944000	-0.703237000	2.189424000
C	-0.210256000	-0.707552000	1.433834000
C	0.945825000	-0.980511000	2.166887000
C	-1.441010000	-1.251687000	1.770374000
N	-2.294699000	-0.749567000	-0.480655000
N	2.216767000	0.354182000	0.664204000
C	3.380669000	2.426430000	0.189714000
H	3.433080000	2.725031000	1.247188000
H	2.450626000	2.848539000	-0.217805000
C	0.869671000	-1.811584000	3.288889000
H	1.766707000	-2.041277000	3.869912000
C	3.416658000	0.907267000	0.057522000
C	-1.505924000	-2.090930000	2.889975000
H	-2.459486000	-2.546433000	3.172203000
C	-2.650574000	-0.914914000	0.932929000
H	-3.416213000	-1.702396000	1.061707000
C	-0.362823000	-2.356920000	3.646015000
H	-0.433657000	-3.011777000	4.517175000
C	-3.357488000	-0.114719000	-1.305159000
C	-3.455167000	1.388029000	-1.103521000
H	-2.516271000	1.897547000	-1.365577000
H	-3.696469000	1.662750000	-0.068025000
C	4.711735000	0.304432000	0.569741000
H	4.711204000	-0.786932000	0.433713000
H	5.555180000	0.716602000	-0.001997000
H	4.887672000	0.543599000	1.630878000

C	-4.728157000	-0.770033000	-1.140491000
H	-5.176017000	-0.527721000	-0.164692000
H	-5.411752000	-0.396102000	-1.916242000
H	-4.687066000	-1.864125000	-1.241348000
H	4.228355000	2.872714000	-0.349853000
H	-4.240546000	1.791146000	-1.759593000
H	0.788236000	-3.929574000	1.116417000
C	-0.811020000	2.505097000	1.923045000
O	0.394261000	2.380958000	1.617690000
O	-1.758818000	1.946236000	1.346993000
H	1.211102000	-3.785061000	0.499741000
C	-1.124879000	3.439188000	3.108675000
C	1.542513000	-1.775048000	-1.803051000
O	0.691158000	-0.823096000	-1.791251000
O	2.539368000	-1.937147000	-1.128819000
C	1.182909000	-2.889181000	-2.820676000
H	3.326107000	0.639919000	-1.009846000
H	-3.026587000	-0.289033000	-2.342210000
F	-2.389623000	3.332174000	3.496360000
F	-0.902321000	4.708445000	2.762494000
F	-0.346319000	3.151638000	4.149469000
F	2.263956000	-3.357519000	-3.430773000
F	0.326079000	-2.478039000	-3.756706000
F	0.601175000	-3.910760000	-2.171973000
H	-3.069043000	0.034653000	1.300570000
C	-1.916393000	-2.041589000	-1.052512000
H	-1.087237000	-2.481937000	-0.485479000
H	-2.758196000	-2.757177000	-1.028785000

H	-1.581101000	-1.908974000	-2.087276000

Int4 (1D)

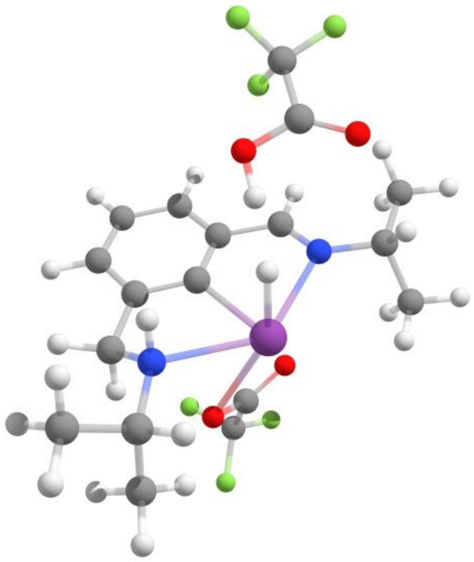

Bi	-0.427566000	0.751495000	-0.455555000
C	2.166328000	-0.365816000	1.008307000
H	3.172316000	-0.562366000	1.404589000
C	-0.247212000	-0.709838000	1.203529000
C	1.032070000	-0.996995000	1.681260000
C	-1.381158000	-1.277375000	1.768265000
N	-2.628391000	-0.540182000	-0.190847000
N	1.988070000	0.374370000	-0.016769000
C	2.834953000	2.562090000	-0.618412000
H	2.845313000	2.885232000	0.432470000
H	1.858236000	2.851147000	-1.033814000
C	1.188102000	-1.880082000	2.755095000
H	2.186024000	-2.121968000	3.129841000
C	3.063853000	1.057019000	-0.724257000
C	-1.216922000	-2.163165000	2.840518000
H	-2.093180000	-2.628941000	3.300925000
C	-2.734348000	-0.883350000	1.230479000

H	-3.467348000	-1.689796000	1.409827000
C	0.057314000	-2.456998000	3.329388000
H	0.167171000	-3.150915000	4.165413000
C	-3.841269000	-0.021735000	-0.858163000
C	-4.398235000	1.208037000	-0.160076000
H	-3.608921000	1.928282000	0.094534000
H	-4.910019000	0.941168000	0.777307000
C	4.462940000	0.656271000	-0.288339000
H	4.623579000	-0.426662000	-0.393801000
H	5.198388000	1.160058000	-0.931010000
H	4.672627000	0.955241000	0.751058000
C	-4.900034000	-1.103492000	-1.042166000
H	-5.303759000	-1.441673000	-0.075099000
H	-5.743152000	-0.714799000	-1.632259000
H	-4.496754000	-1.979903000	-1.574477000
H	3.614096000	3.101459000	-1.176173000
H	-5.139163000	1.694195000	-0.810758000
H	-0.175669000	-0.730680000	-1.697117000
C	-0.786959000	2.501445000	1.967927000
O	0.374423000	2.398890000	1.545279000
O	-1.810175000	1.963811000	1.491411000
H	0.656775000	-1.915607000	-1.466459000
C	-1.023138000	3.346317000	3.240586000
C	2.462756000	-2.506161000	-1.546832000
O	2.888107000	-1.676330000	-2.301257000
O	1.224751000	-2.688586000	-1.164769000
C	3.395060000	-3.505970000	-0.833090000
H	2.941955000	0.751177000	-1.777845000

H	-3.497537000	0.282865000	-1.862693000
F	-1.254093000	2.540499000	4.283997000
F	-2.087105000	4.137993000	3.100403000
F	0.020209000	4.111432000	3.541689000
F	2.977924000	-4.757491000	-0.976482000
F	3.427706000	-3.224546000	0.476738000
F	4.626869000	-3.407761000	-1.303119000
H	-2.328411000	-1.376609000	-0.695036000
H	-3.066290000	0.017366000	1.768641000

TS45 (1D)

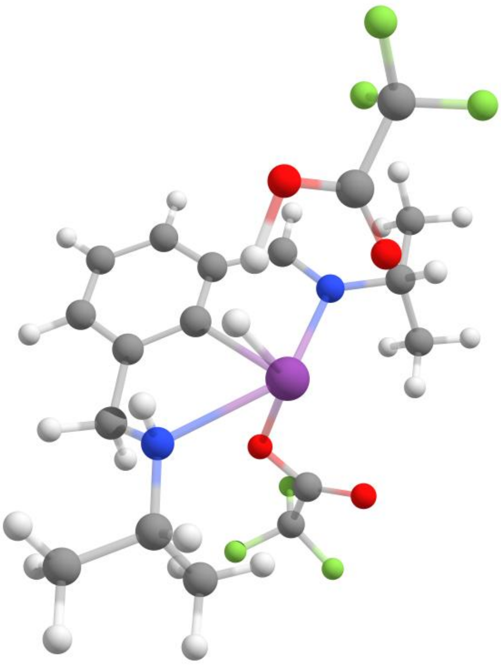

Bi	-0.214770000	0.072153000	-0.475752000
C	2.240091000	-0.809011000	1.440574000
H	3.213867000	-1.059081000	1.888065000
C	-0.176019000	-1.223772000	1.352128000
C	1.053958000	-1.507840000	1.948875000
C	-1.366933000	-1.628142000	1.943330000
N	-2.589249000	-0.779076000	-0.003081000
N	2.123350000	0.095608000	0.551266000

C	2.910048000	2.363437000	0.287390000
H	2.874837000	2.576326000	1.367745000
H	1.941266000	2.652193000	-0.147026000
C	1.107194000	-2.327605000	3.081491000
H	2.068530000	-2.565607000	3.545201000
C	3.228016000	0.892110000	0.038237000
C	-1.309116000	-2.445184000	3.080733000
H	-2.234789000	-2.789399000	3.551313000
C	-2.672241000	-1.079755000	1.424161000
H	-3.498321000	-1.775726000	1.650777000
C	-0.079901000	-2.812866000	3.627625000
H	-0.050372000	-3.459464000	4.507270000
C	-3.743707000	-0.087829000	-0.616216000
C	-3.755996000	1.398393000	-0.295418000
H	-2.887870000	1.915621000	-0.728693000
H	-3.757478000	1.591724000	0.786914000
C	4.597550000	0.496741000	0.563543000
H	4.829590000	-0.555036000	0.339620000
H	5.363544000	1.115804000	0.075658000
H	4.685902000	0.660670000	1.650016000
C	-5.072276000	-0.757948000	-0.282014000
H	-5.354334000	-0.601053000	0.770505000
H	-5.874400000	-0.333226000	-0.902790000
H	-5.039250000	-1.841926000	-0.474452000
H	3.686734000	2.997835000	-0.163731000
H	-4.658850000	1.860793000	-0.719670000
H	-0.696942000	-1.830724000	-1.481721000
C	-0.866500000	2.756130000	0.786460000

O	-0.474871000	3.138726000	-0.304789000
O	-0.915173000	1.560079000	1.216919000
H	0.097574000	-2.405706000	-1.723542000
C	-1.377027000	3.781525000	1.822280000
C	2.025293000	-2.104394000	-2.092639000
O	1.873017000	-0.887039000	-2.018940000
O	1.141516000	-3.003541000	-1.991587000
C	3.471421000	-2.626136000	-2.229484000
H	3.208822000	0.709902000	-1.049340000
H	-3.579557000	-0.195681000	-1.702089000
F	-0.533800000	3.850895000	2.853763000
F	-2.574427000	3.415649000	2.297008000
F	-1.497763000	4.993942000	1.303510000
F	3.532922000	-3.844566000	-2.741993000
F	4.016822000	-2.665109000	-1.000611000
F	4.211076000	-1.814333000	-2.974058000
H	-2.441831000	-1.657769000	-0.504090000
H	-2.874103000	-0.137083000	1.959886000

Int5 (1D)

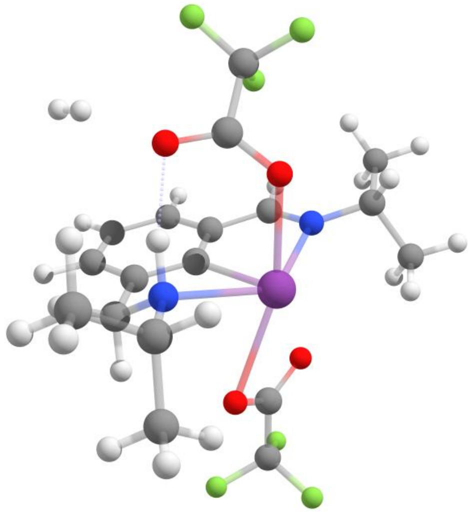

Bi	-0.111733000	0.968438000	-0.155696000
C	2.418102000	-0.340732000	1.337033000
H	3.402537000	-0.684538000	1.688038000
C	-0.015800000	-0.622904000	1.387667000
C	1.228482000	-0.977979000	1.910338000
C	-1.184952000	-1.263033000	1.773149000
N	-2.177556000	-0.452277000	-0.282300000
N	2.299462000	0.544662000	0.430143000
C	3.368778000	2.675517000	0.016967000
H	3.551423000	2.876883000	1.083194000
H	2.386400000	3.103914000	-0.228295000
C	1.300958000	-1.969612000	2.893582000
H	2.267920000	-2.266530000	3.308756000
C	3.410563000	1.175392000	-0.258864000
C	-1.103346000	-2.254007000	2.759722000
H	-2.006285000	-2.783622000	3.075982000
C	-2.473815000	-0.880188000	1.087483000
H	-3.173401000	-1.733794000	1.107565000
C	0.127648000	-2.589659000	3.323207000
H	0.175738000	-3.368580000	4.087150000
C	-3.310471000	-0.022408000	-1.122991000
C	-4.083843000	1.135207000	-0.513045000
H	-3.416969000	1.938819000	-0.171807000
H	-4.678801000	0.811303000	0.354895000
C	4.772768000	0.570333000	0.034837000
H	4.789368000	-0.506909000	-0.189116000
H	5.532077000	1.055356000	-0.594793000
H	5.070873000	0.721960000	1.084702000

C	-4.213920000	-1.194801000	-1.489271000
H	-4.754542000	-1.579709000	-0.610440000
H	-4.967242000	-0.880290000	-2.226783000
H	-3.630831000	-2.019741000	-1.926181000
H	4.133054000	3.194529000	-0.579254000
H	-4.785034000	1.544208000	-1.254745000
H	-0.299122000	-4.854418000	0.663704000
C	-0.760988000	2.656018000	2.057880000
O	0.444958000	2.542140000	1.780010000
O	-1.691082000	2.058851000	1.471043000
H	-0.330062000	-4.368134000	0.076615000
C	-1.157253000	3.653049000	3.164056000
C	0.487410000	-1.849227000	-1.712694000
O	0.688503000	-0.598377000	-1.730723000
O	-0.541607000	-2.456807000	-1.442312000
C	1.762295000	-2.659391000	-2.038045000
H	3.189669000	1.012496000	-1.330090000
H	-2.840749000	0.334212000	-2.056773000
F	-2.284405000	3.289817000	3.765503000
F	-1.348503000	4.857009000	2.617651000
F	-0.208222000	3.764363000	4.085033000
F	1.521632000	-3.955134000	-2.160807000
F	2.649335000	-2.498589000	-1.039663000
F	2.339140000	-2.232180000	-3.159281000
H	-1.713418000	-1.250330000	-0.758769000
H	-2.938880000	-0.041467000	1.628983000

References

(1) Perrin, D. D., & Armarego, W. L. F. Purification of laboratory chemicals.**1988** *Pergamon Press*.

(2) Moon, H. W.; Wang, F.; Bhattacharyya, K.; Planas, O.; Leutzsch, M.; Nöthling, N.; Auer, A. A.; Cornella, J. Mechanistic Studies on the Bismuth-Catalyzed Transfer Hydrogenation of Azoarenes. *Angew. Chemie Int. Ed.* **2023**, *62* (49), e202313578.

(3) Mato, M.; Bruzzese, P. C.; Takahashi, F.; Leutzsch, M.; Reijerse, E. J.; Schnegg, A.; Cornella, J. Oxidative Addition of Aryl Electrophiles into a Red-Light-Active Bismuthinidene. *J. Am. Chem. Soc.* **2023**, *145* (34), 18742.

(4) Wang, F.; Planas, O.; Cornella, J. Bi(I)-Catalyzed Transfer-Hydrogenation with Ammonia-Borane. *J. Am. Chem. Soc.* **2019**, *141* (10), 4235–4240.

(5) McCarthy, B. D.; Dempsey, J. L. Decoding Proton-Coupled Electron Transfer with Potential–PKa Diagrams. *Inorg. Chem.* **2017**, *56* (3), 1225–1231.

(6) Elgrishi, N.; Rountree, K. J.; McCarthy, B. D.; Rountree, E. S.; Eisenhart, T. T.; Dempsey, J. L. A Practical Beginner's Guide to Cyclic Voltammetry. *J. Chem. Educ.* **2018**, *95* (2), 197.

(7) Upadhyay, A.; Kanika; Mandhar, Y.; Batabyal, M.; Raju, S.; Jaiswal, S.; Butcher, R. J.; Kumar, S. Synthesis of Cobalt(Ii) Phenolate Selenoether Complexes to Mimic Hydrogenase-like Activity for Hydrogen Gas Production. *Dalt. Trans.* **2023**, *52* (1), 159–174.

(8) Roy, S.; Sharma, B.; Pécaut, J.; Simon, P.; Fontecave, M.; Tran, P. D.; Derat, E.; Artero, V. Molecular Cobalt Complexes with Pendant Amines for Selective Electrocatalytic Reduction of Carbon Dioxide to Formic Acid. *J. Am. Chem. Soc.* **2017**, *139* (10), 3685.

(9) Stratakes, B. M.; Dempsey, J. L.; Miller, A. J. M. Determining the Overpotential of Electrochemical Fuel Synthesis Mediated by Molecular Catalysts: Recommended Practices, Standard Reduction Potentials, and Challenges. *ChemElectroChem* **2021**, *8* (22), 4161–4180.

(10) Wang, V. C.-C.; Johnson, B. A. Interpreting the Electrocatalytic Voltammetry of Homogeneous Catalysts by the Foot of the Wave Analysis and Its Wider Implications. *ACS Catal.* **2019**, *9*, 7109.

(11) Rountree, E. S.; McCarthy, B. D.; Eisenhart, T. T.; Dempsey, J. L. Evaluation of Homogeneous Electrocatalysts by Cyclic Voltammetry. *Inorg. Chem.* **2014**, *53* (19), 9983.

(12) Mandal, S. K.; Sunil, C.; Choudhury, J. [Fe]-Hydrogenase-Inspired Proton-Shuttle Installation in a Molecular Cobalt Complex for High-Efficiency H2 Evolution Reaction. *ACS Catal.* **2024**, *14* (3), 2058–2070.

(13) Rountree, E. S.; Martin, D. J.; McCarthy, B. D.; Dempsey, J. L. Correction to "Linear Free Energy Relationships in the Hydrogen Evolution Reaction: Kinetic Analysis of a Cobaloxime Catalyst." *ACS Catal.* **2016**, *6* (9), 5684.

(14) Martin, D. J.; McCarthy, B. D.; Rountree, E. S.; Dempsey, J. L. Qualitative Extension of the EC′ Zone Diagram to a Molecular Catalyst for a Multi-Electron, Multi-Substrate Electrochemical Reaction. *Dalt. Trans.* **2016**, *45* (24), 9970–9976.

(15) Costentin, C.; Savéant, J.-M. Multielectron, Multistep Molecular Catalysis of Electrochemical Reactions: Benchmarking of Homogeneous Catalysts. *ChemElectroChem.* **2014**, *1* (7), 1226.

(16) Lee, K. J.; Elgrishi, N.; Kandemir, B.; Dempsey, J. L. Electrochemical and Spectroscopic Methods for Evaluating Molecular Electrocatalysts. *Nat. Rev. Chem.* **2017**, *1* (5), 39.

(17) van der Heijden, O.; Park, S.; Vos, R. E.; Eggebeen, J. J. J.; Koper, M. T. M. Tafel Slope Plot as a Tool to Analyze Electrocatalytic Reactions. *ACS Energy Lett.* **2024**, *9* (4), 1871–1879.

(18) Bard, A. J., & Faulkner, L. R. *Electrochemical Methods: Fundamentals and Applications*. *John Wiley & Sons* vol. 2nd ed. **2001**.

(19) Neese, F. The ORCA Program System. *WIREs Comput. Mol. Sci.* **2012**, *2* (1), 73–78.

(20) Neese, F. Software Update: The ORCA Program System—Version 5.0. *WIREs Comput. Mol. Sci.* **2022**, *12* (5), e1606.

(21) Adamo, C.; Barone, V. Toward Reliable Density Functional Methods without Adjustable Parameters: The PBE0 Model. *J. Chem. Phys.* **1999**, *110* (13), 6158–6170.

(22) Schäfer, A.; Horn, H.; Ahlrichs, R. Fully Optimized Contracted Gaussian Basis Sets for Atoms Li to Kr. *J. Chem. Phys.* **1992**, *97* (4), 2571.

(23) Neese, F.; Wennmohs, F.; Hansen, A.; Becker, U. Efficient, Approximate and Parallel Hartree–Fock and Hybrid DFT Calculations. A ‘Chain-of-Spheres’ Algorithm for the Hartree–Fock Exchange. *Chem. Phys.* **2009**, *356* (1), 98–109.

(24) Grimme, S.; Ehrlich, S.; Goerigk, L. Effect of the Damping Function in Dispersion Corrected Density Functional Theory. *J. Comput. Chem.* **2011**, *32* (7), 1456–1465.

(25) Weigend, F.; Ahlrichs, R. Balanced Basis Sets of Split Valence, Triple Zeta Valence and Quadruple Zeta Valence Quality for H to Rn: Design and Assessment of Accuracy. *Phys. Chem. Chem. Phys.* **2005**, *7* (18), 3297.

(26) Marenich, A. V; Cramer, C. J.; Truhlar, D. G. Universal Solvation Model Based on Solute Electron Density and on a Continuum Model of the Solvent Defined by the Bulk Dielectric Constant and Atomic Surface Tensions. *J. Phys. Chem. B* **2009**, *113* (18), 6378–6396.

(27) Knizia, G. Intrinsic Atomic Orbitals: An Unbiased Bridge between Quantum Theory and Chemical Concepts. *J. Chem. Theory Comput.* **2013**, *9* (11), 4834–4843.

(28) Chen, S.; Jing, H.-X.; Lin, J.; Ye, M.; Huang, Y.-Y.; Li, S.-J.; Lan, Y.; Song, Q. Copper-Catalyzed Chemodivergent and Regioselective Ring-Opening Diborylation of Vinylcyclopropanes to Access Diversified 1,5-Diboronates. *ACS Catal.* **2026**, *16* (10), 9317–9332.

(29) Maji, S.; Singh, N. K.; Manna, S.; Kabari, S.; Chauhan, R. S.; Guin, S.; Raul, A.; Gupta, P.; Maiti, D. C–H Activation Initiated Skeletal Recasting of Cyclopropane Carboxylic Acid. *ACS Catal.* **2026**, *16* (5), 4516–4527.

(30) Lu, T.; Chen, F. Multiwfn: A Multifunctional Wavefunction Analyzer. *J. Comput. Chem.* **2012**, *33* (5), 580–592.

(31) Humphrey, W.; Dalke, A.; Schulten, K. VMD: Visual Molecular Dynamics. *J. Mol. Graph.* **1996**, *14* (1), 33–38.
